# Cancer Incidence, Mortality, Years of Life Lost, Years Lived With Disability, and Disability-Adjusted Life Years for 29 Cancer Groups From 2010 to 2019: A Systematic Analysis for the Global Burden of Disease Study 2019

**DOI:** 10.1001/jamaoncol.2021.6987

**Published:** 2021-12-30

**Authors:** Global Burden of Disease 2019 Cancer Collaboration, Jonathan M. Kocarnik, Kelly Compton, Frances E. Dean, Weijia Fu, Brian L. Gaw, James D. Harvey, Hannah Jacqueline Henrikson, Dan Lu, Alyssa Pennini, Rixing Xu, Emad Ababneh, Mohsen Abbasi-Kangevari, Hedayat Abbastabar, Sherief M. Abd-Elsalam, Amir Abdoli, Aidin Abedi, Hassan Abidi, Hassan Abolhassani, Isaac Akinkunmi Adedeji, Qorinah Estiningtyas Sakilah Adnani, Shailesh M. Advani, Muhammad Sohail Afzal, Mohammad Aghaali, Bright Opoku Ahinkorah, Sajjad Ahmad, Tauseef Ahmad, Ali Ahmadi, Sepideh Ahmadi, Tarik Ahmed Rashid, Yusra Ahmed Salih, Gizachew Taddesse Akalu, Addis Aklilu, Tayyaba Akram, Chisom Joyqueenet Akunna, Hanadi Al Hamad, Fares Alahdab, Ziyad Al-Aly, Saqib Ali, Yousef Alimohamadi, Vahid Alipour, Syed Mohamed Aljunid, Motasem Alkhayyat, Amir Almasi-Hashiani, Nihad A. Almasri, Sadeq Ali Ali Al-Maweri, Sami Almustanyir, Nivaldo Alonso, Nelson Alvis-Guzman, Hubert Amu, Etsay Woldu Anbesu, Robert Ancuceanu, Fereshteh Ansari, Alireza Ansari-Moghaddam, Maxwell Hubert Antwi, Davood Anvari, Anayochukwu Edward Anyasodor, Muhammad Aqeel, Jalal Arabloo, Morteza Arab-Zozani, Olatunde Aremu, Hany Ariffin, Timur Aripov, Muhammad Arshad, Al Artaman, Judie Arulappan, Zatollah Asemi, Mohammad Asghari Jafarabadi, Tahira Ashraf, Prince Atorkey, Avinash Aujayeb, Marcel Ausloos, Atalel Fentahun Awedew, Beatriz Paulina Ayala Quintanilla, Temesgen Ayenew, Mohammed A. Azab, Sina Azadnajafabad, Amirhossein Azari Jafari, Ghasem Azarian, Ahmed Y. Azzam, Ashish D. Badiye, Saeed Bahadory, Atif Amin Baig, Jennifer L. Baker, Senthilkumar Balakrishnan, Maciej Banach, Till Winfried Bärnighausen, Francesco Barone-Adesi, Fabio Barra, Amadou Barrow, Masoud Behzadifar, Uzma Iqbal Belgaumi, Woldesellassie M. Mequanint Bezabhe, Yihienew Mequanint Bezabih, Devidas S. Bhagat, Akshaya Srikanth Bhagavathula, Nikha Bhardwaj, Pankaj Bhardwaj, Sonu Bhaskar, Krittika Bhattacharyya, Vijayalakshmi S. Bhojaraja, Sadia Bibi, Ali Bijani, Antonio Biondi, Catherine Bisignano, Tone Bjørge, Archie Bleyer, Oleg Blyuss, Obasanjo Afolabi Bolarinwa, Srinivasa Rao Bolla, Dejana Braithwaite, Amanpreet Brar, Hermann Brenner, Maria Teresa Bustamante-Teixeira, Nadeem Shafique Butt, Zahid A. Butt, Florentino Luciano Caetano dos Santos, Yin Cao, Giulia Carreras, Ferrán Catalá-López, Francieli Cembranel, Ester Cerin, Achille Cernigliaro, Raja Chandra Chakinala, Soosanna Kumary Chattu, Vijay Kumar Chattu, Pankaj Chaturvedi, Odgerel Chimed-Ochir, Daniel Youngwhan Cho, Devasahayam J. Christopher, Dinh-Toi Chu, Michael T. Chung, Joao Conde, Sanda Cortés, Paolo Angelo Cortesi, Vera Marisa Costa, Amanda Ramos Cunha, Omid Dadras, Amare Belachew Dagnew, Saad M. A. Dahlawi, Xiaochen Dai, Lalit Dandona, Rakhi Dandona, Aso Mohammad Darwesh, José das Neves, Fernando Pio De la Hoz, Asmamaw Bizuneh Demis, Edgar Denova-Gutiérrez, Deepak Dhamnetiya, Mandira Lamichhane Dhimal, Meghnath Dhimal, Mostafa Dianatinasab, Daniel Diaz, Shirin Djalalinia, Huyen Phuc Do, Saeid Doaei, Fariba Dorostkar, Francisco Winter dos Santos Figueiredo, Tim Robert Driscoll, Hedyeh Ebrahimi, Sahar Eftekharzadeh, Maha El Tantawi, Hassan El-Abid, Iffat Elbarazi, Hala Rashad Elhabashy, Muhammed Elhadi, Shaimaa I. El-Jaafary, Babak Eshrati, Sharareh Eskandarieh, Firooz Esmaeilzadeh, Arash Etemadi, Sayeh Ezzikouri, Mohammed Faisaluddin, Emerito Jose A. Faraon, Jawad Fares, Farshad Farzadfar, Abdullah Hamid Feroze, Simone Ferrero, Lorenzo Ferro Desideri, Irina Filip, Florian Fischer, James L. Fisher, Masoud Foroutan, Takeshi Fukumoto, Peter Andras Gaal, Mohamed M. Gad, Muktar A. Gadanya, Silvano Gallus, Mariana Gaspar Fonseca, Abera Getachew Obsa, Mansour Ghafourifard, Ahmad Ghashghaee, Nermin Ghith, Maryam Gholamalizadeh, Syed Amir Gilani, Themba G. Ginindza, Abraham Tamirat T. Gizaw, James C. Glasbey, Mahaveer Golechha, Pouya Goleij, Ricardo Santiago Gomez, Sameer Vali Gopalani, Giuseppe Gorini, Houman Goudarzi, Giuseppe Grosso, Mohammed Ibrahim Mohialdeen Gubari, Maximiliano Ribeiro Guerra, Avirup Guha, D. Sanjeeva Gunasekera, Bhawna Gupta, Veer Bala Gupta, Vivek Kumar Gupta, Reyna Alma Gutiérrez, Nima Hafezi-Nejad, Mohammad Rifat Haider, Arvin Haj-Mirzaian, Rabih Halwani, Randah R. Hamadeh, Sajid Hameed, Samer Hamidi, Asif Hanif, Shafiul Haque, Netanja I. Harlianto, Josep Maria Haro, Ahmed I. Hasaballah, Soheil Hassanipour, Roderick J. Hay, Simon I. Hay, Khezar Hayat, Golnaz Heidari, Mohammad Heidari, Brenda Yuliana Herrera-Serna, Claudiu Herteliu, Kamal Hezam, Ramesh Holla, Md Mahbub Hossain, Mohammad Bellal Hossain Hossain, Mohammad-Salar Hosseini, Mostafa Hosseini, Mehdi Hosseinzadeh, Mihaela Hostiuc, Sorin Hostiuc, Mowafa Househ, Mohamed Hsairi, Junjie Huang, Fernando N. Hugo, Rabia Hussain, Nawfal R. Hussein, Bing-Fang Hwang, Ivo Iavicoli, Segun Emmanuel Ibitoye, Fidelia Ida, Kevin S. Ikuta, Olayinka Stephen Ilesanmi, Irena M. Ilic, Milena D. Ilic, Lalu Muhammad Irham, Jessica Y. Islam, Rakibul M. Islam, Sheikh Mohammed Shariful Islam, Nahlah Elkudssiah Ismail, Gaetano Isola, Masao Iwagami, Louis Jacob, Vardhmaan Jain, Mihajlo B. Jakovljevic, Tahereh Javaheri, Shubha Jayaram, Seyed Behzad Jazayeri, Ravi Prakash Jha, Jost B. Jonas, Tamas Joo, Nitin Joseph, Farahnaz Joukar, Mikk Jürisson, Ali Kabir, Danial Kahrizi, Leila R. Kalankesh, Rohollah Kalhor, Feroze Kaliyadan, Yogeshwar Kalkonde, Ashwin Kamath, Nawzad Kameran Al-Salihi, Himal Kandel, Neeti Kapoor, André Karch, Ayele Semachew Kasa, Srinivasa Vittal Katikireddi, Joonas H. Kauppila, Taras Kavetskyy, Sewnet Adem Kebede, Pedram Keshavarz, Mohammad Keykhaei, Yousef Saleh Khader, Rovshan Khalilov, Gulfaraz Khan, Maseer Khan, Md Nuruzzaman Khan, Moien A. B. Khan, Young-Ho Khang, Amir M. Khater, Maryam Khayamzadeh, Gyu Ri Kim, Yun Jin Kim, Adnan Kisa, Sezer Kisa, Katarzyna Kissimova-Skarbek, Jacek A. Kopec, Rajasekaran Koteeswaran, Parvaiz A. Koul, Sindhura Lakshmi Koulmane Laxminarayana, Ai Koyanagi, Burcu Kucuk Bicer, Nuworza Kugbey, G. Anil Kumar, Narinder Kumar, Nithin Kumar, Om P. Kurmi, Tezer Kutluk, Carlo La Vecchia, Faris Hasan Lami, Iván Landires, Paolo Lauriola, Sang-woong Lee, Shaun Wen Huey Lee, Wei-Chen Lee, Yo Han Lee, James Leigh, Elvynna Leong, Jiarui Li, Ming-Chieh Li, Xuefeng Liu, Joana A. Loureiro, Raimundas Lunevicius, Muhammed Magdy Abd El Razek, Azeem Majeed, Alaa Makki, Shilpa Male, Ahmad Azam Malik, Mohammad Ali Mansournia, Santi Martini, Seyedeh Zahra Masoumi, Prashant Mathur, Martin McKee, Ravi Mehrotra, Walter Mendoza, Ritesh G. Menezes, Endalkachew Worku Mengesha, Mohamed Kamal Mesregah, Tomislav Mestrovic, Junmei Miao Jonasson, Bartosz Miazgowski, Tomasz Miazgowski, Irmina Maria Michalek, Ted R. Miller, Hamed Mirzaei, Hamid Reza Mirzaei, Sanjeev Misra, Prasanna Mithra, Masoud Moghadaszadeh, Karzan Abdulmuhsin Mohammad, Yousef Mohammad, Mokhtar Mohammadi, Seyyede Momeneh Mohammadi, Abdollah Mohammadian-Hafshejani, Shafiu Mohammed, Nagabhishek Moka, Ali H. Mokdad, Mariam Molokhia, Lorenzo Monasta, Mohammad Ali Moni, Mohammad Amin Moosavi, Yousef Moradi, Paula Moraga, Joana Morgado-da-Costa, Shane Douglas Morrison, Abbas Mosapour, Sumaira Mubarik, Lillian Mwanri, Ahamarshan Jayaraman Nagarajan, Shankar Prasad Nagaraju, Chie Nagata, Mukhammad David Naimzada, Vinay Nangia, Atta Abbas Naqvi, Sreenivas Narasimha Swamy, Rawlance Ndejjo, Sabina O. Nduaguba, Ionut Negoi, Serban Mircea Negru, Sandhya Neupane Kandel, Cuong Tat Nguyen, Huong Lan Thi Nguyen, Robina Khan Niazi, Chukwudi A. Nnaji, Nurulamin M. Noor, Virginia Nuñez-Samudio, Chimezie Igwegbe Nzoputam, Bogdan Oancea, Chimedsuren Ochir, Oluwakemi Ololade Odukoya, Felix Akpojene Ogbo, Andrew T. Olagunju, Babayemi Oluwaseun Olakunde, Emad Omar, Ahmed Omar Bali, Abidemi E. Emmanuel Omonisi, Sokking Ong, Obinna E. Onwujekwe, Hans Orru, Doris V. Ortega-Altamirano, Nikita Otstavnov, Stanislav S. Otstavnov, Mayowa O. Owolabi, Mahesh P A, Jagadish Rao Padubidri, Keyvan Pakshir, Adrian Pana, Demosthenes Panagiotakos, Songhomitra Panda-Jonas, Shahina Pardhan, Eun-Cheol Park, Eun-Kee Park, Fatemeh Pashazadeh Kan, Harsh K. Patel, Jenil R. Patel, Siddhartha Pati, Sanjay M. Pattanshetty, Uttam Paudel, David M. Pereira, Renato B. Pereira, Arokiasamy Perianayagam, Julian David Pillay, Saeed Pirouzpanah, Farhad Pishgar, Indrashis Podder, Maarten J. Postma, Hadi Pourjafar, Akila Prashant, Liliana Preotescu, Mohammad Rabiee, Navid Rabiee, Amir Radfar, Raghu Anekal Radhakrishnan, Venkatraman Radhakrishnan, Ata Rafiee, Fakher Rahim, Shadi Rahimzadeh, Mosiur Rahman, Muhammad Aziz Rahman, Amir Masoud Rahmani, Nazanin Rajai, Aashish Rajesh, Ivo Rakovac, Pradhum Ram, Kiana Ramezanzadeh, Kamal Ranabhat, Priyanga Ranasinghe, Chythra R. Rao, Sowmya J. Rao, Reza Rawassizadeh, Mohammad Sadegh Razeghinia, Andre M. N. Renzaho, Negar Rezaei, Nima Rezaei, Aziz Rezapour, Thomas J. Roberts, Jefferson Antonio Buendia Rodriguez, Peter Rohloff, Michele Romoli, Luca Ronfani, Gholamreza Roshandel, Godfrey M. Rwegerera, Manjula S, Siamak Sabour, Basema Saddik, Umar Saeed, Amirhossein Sahebkar, Harihar Sahoo, Sana Salehi, Marwa Rashad Salem, Hamideh Salimzadeh, Mehrnoosh Samaei, Abdallah M. Samy, Juan Sanabria, Senthilkumar Sankararaman, Milena M. Santric-Milicevic, Yaeesh Sardiwalla, Arash Sarveazad, Brijesh Sathian, Monika Sawhney, Mete Saylan, Ione Jayce Ceola Schneider, Mario Sekerija, Allen Seylani, Omid Shafaat, Zahra Shaghaghi, Masood Ali Shaikh, Erfan Shamsoddin, Mohammed Shannawaz, Rajesh Sharma, Aziz Sheikh, Sara Sheikhbahaei, Adithi Shetty, Jeevan K. Shetty, Pavanchand H. Shetty, Kenji Shibuya, Reza Shirkoohi, K. M. Shivakumar, Velizar Shivarov, Soraya Siabani, Sudeep K. Siddappa Malleshappa, Diego Augusto Santos Silva, Jasvinder A. Singh, Yitagesu Sintayehu, Valentin Yurievich Skryabin, Anna Aleksandrovna Skryabina, Matthew J. Soeberg, Ahmad Sofi-Mahmudi, Houman Sotoudeh, Paschalis Steiropoulos, Kurt Straif, Ranjeeta Subedi, Mu'awiyyah Babale Sufiyan, Iyad Sultan, Saima Sultana, Daniel Sur, Viktória Szerencsés, Miklós Szócska, Rafael Tabarés-Seisdedos, Takahiro Tabuchi, Hooman Tadbiri, Amir Taherkhani, Ken Takahashi, Iman M. Talaat, Ker-Kan Tan, Vivian Y. Tat, Bemnet Amare A. Tedla, Yonas Getaye Tefera, Arash Tehrani-Banihashemi, Mohamad-Hani Temsah, Fisaha Haile Tesfay, Gizachew Assefa Tessema, Rekha Thapar, Aravind Thavamani, Viveksandeep Thoguluva Chandrasekar, Nihal Thomas, Hamid Reza Tohidinik, Mathilde Touvier, Marcos Roberto Tovani-Palone, Eugenio Traini, Bach Xuan Tran, Khanh Bao Tran, Mai Thi Ngoc Tran, Jaya Prasad Tripathy, Biruk Shalmeno Tusa, Irfan Ullah, Saif Ullah, Krishna Kishore Umapathi, Bhaskaran Unnikrishnan, Era Upadhyay, Marco Vacante, Maryam Vaezi, Sahel Valadan Tahbaz, Diana Zuleika Velazquez, Massimiliano Veroux, Francesco S. Violante, Vasily Vlassov, Bay Vo, Victor Volovici, Giang Thu Vu, Yasir Waheed, Richard G. Wamai, Paul Ward, Yi Feng Wen, Ronny Westerman, Andrea Sylvia Winkler, Lalit Yadav, Seyed Hossein Yahyazadeh Jabbari, Lin Yang, Sanni Yaya, Taklo Simeneh Yazie Yazie, Yigizie Yeshaw, Naohiro Yonemoto, Mustafa Z. Younis, Zabihollah Yousefi, Chuanhua Yu, Deniz Yuce, Ismaeel Yunusa, Vesna Zadnik, Fariba Zare, Mikhail Sergeevich Zastrozhin, Anasthasia Zastrozhina, Jianrong Zhang, Chenwen Zhong, Linghui Zhou, Cong Zhu, Arash Ziapour, Ivan R. Zimmermann, Christina Fitzmaurice, Christopher J. L. Murray, Lisa M. Force

**Affiliations:** 1 Institute for Health Metrics and Evaluation, University of Washington, Seattle; 2 Department of Global Health, Boston University, Boston, Massachusetts; 3 Department of Medicine, Brigham and Women's Hospital, Boston, Massachusetts; 4 Pathology and Laboratory Medicine Institute, Cleveland Clinic, Cleveland, Ohio; 5 Social Determinants of Health Research Center, Shahid Beheshti University of Medical Sciences, Tehran, Iran; 6 Advanced Diagnostic and Interventional Radiology Research Center, Tehran University of Medical Sciences, Tehran, Iran; 7 Tropical Medicine Department, Tanta University, Tanta, Egypt; 8 Zoonoses Research Center, Jahrom University of Medical Sciences, Jahrom, Iran; 9 Department of Orthopaedic Surgery, University of Southern California, Los Angeles; 10 Laboratory Technology Sciences Department, Yasouj University, Yasuj, Iran; 11 Department of Laboratory Medicine, Karolinska University Hospital, Huddinge, Sweden; 12 Research Center for Immunodeficiencies, Tehran University of Medical Sciences, Tehran, Iran; 13 Department of Sociology, Olabisi Onabanjo University, Ago-Iwoye, Nigeria; 14 Department of Midwifery, Karya Husada Institute of Health Sciences, Kediri, Indonesia; 15 Department of Midwifery, Auckland University of Technology, Auckland, New Zealand; 16 Terasaki Institute for Biomedical Innovation, Los Angeles, California; 17 School of Medicine, Georgetown University, Washington, DC; 18 Department of Life Sciences, University of Management and Technology, Lahore, Pakistan; 19 Department of Epidemiology and Biostatistics, Qom University of Medical Sciences, Qom, Iran; 20 The Australian Centre for Public and Population Health Research, University of Technology Sydney, Sydney, New South Wales, Australia; 21 Foundation University Medical College, Foundation University Islamabad, Islamabad, Pakistan; 22 Department of Epidemiology and Health Statistics, Southeast University, Nanjing, China; 23 Department of Epidemiology and Biostatistics, Shahrekord University of Medical Sciences, Shahrekord, Iran; 24 Department of Epidemiology, Shahid Beheshti University of Medical Sciences, Tehran, Iran; 25 School of Advanced Technologies in Medicine, Shahid Beheshti University of Medical Sciences, Tehran, Iran; 26 Department of Computer Science and Engineering, University of Kurdistan Hewler, Erbil, Iraq; 27 Database Technology Department, Sulaimani Polytechnic University, Sulaymaniyah, Iraq; 28 College of Informatics, Sulaimani Polytechnic University, Sulaymaniyah, Iraq; 29 Microbiology, Immunology and Parasitology Department, St Paul's Hospital Millennium Medical College, Addis Ababa, Ethiopia; 30 Microbial, Cellular and Molecular Biology, Addis Ababa University, Addis Ababa, Ethiopia; 31 Department of Medical Laboratory Sciences, Arba Minch University, Arba Minch, Ethiopia; 32 School of Mathematical Sciences, University of Science Malaysia, Penang, Malaysia; 33 Department of Public Health, Intercountry Centre for Oral Health for Africa, Jos, Nigeria; 34 Department of Public Health, Federal Ministry of Health, Garki, Nigeria; 35 Geriatric and Long-Term Care Department, Hamad Medical Corporation, Doha, Qatar; 36 Rumailah Hospital, Hamad Medical Corporation, Doha, Qatar; 37 Mayo Evidence-Based Practice Center, Mayo Clinic Foundation for Medical Education and Research, Rochester, Minnesota; 38 John T. Milliken Department of Internal Medicine, Washington University in St Louis, St Louis, Missouri; 39 Clinical Epidemiology Center, Department of Veterans Affairs, St Louis, Missouri; 40 Department of Information Systems, Sultan Qaboos University, Muscat, Oman; 41 Pars Hospital, Iran University of Medical Sciences, Tehran, Iran; 42 Department of Epidemiology and Biostatistics, Tehran University of Medical Sciences, Tehran, Iran; 43 Health Management and Economics Research Center, Iran University of Medical Sciences, Tehran, Iran; 44 Department of Health Economics, Iran University of Medical Sciences, Tehran, Iran; 45 Department of Health Policy and Management, Kuwait University, Safat, Kuwait; 46 International Centre for Casemix and Clinical Coding, National University of Malaysia, Bandar Tun Razak, Malaysia; 47 Department of Internal Medicine, Cleveland Clinic, Cleveland, Ohio; 48 Department of Epidemiology, Arak University of Medical Sciences, Arak, Iran; 49 Physiotherapy Department, University of Jordan, Amman, Jordan; 50 College of Dental Medicine, Qatar University, Doha, Qatar; 51 Faculty of Dentistry, Sana'a University, Sana’a, Yemen; 52 College of Medicine, Alfaisal University, Riyadh, Saudi Arabia; 53 Ministry of Health, Riyadh, Saudi Arabia; 54 Department of Surgery, University of São Paulo, São Paulo, Brazil; 55 Research Group in Hospital Management and Health Policies, Universidad de la Costa, Barranquilla, Colombia; 56 Research Group in Health Economics, University of Cartagena, Cartagena, Colombia; 57 Department of Population and Behavioural Sciences, University of Health and Allied Sciences, Ho, Ghana; 58 Department of Public Health, Samara University, Samara, Ethiopia; 59 Pharmacy Department, Carol Davila University of Medicine and Pharmacy, Bucharest, Romania; 60 Research Center for Evidence Based Medicine, Tabriz University of Medical Sciences, Tabriz, Iran; 61 Razi Vaccine and Serum Research Institute, Agricultural Research, Education, and Extension Organization, Tehran, Iran; 62 Department of Epidemiology and Biostatistics, Zahedan University of Medical Sciences, Zahedan, Iran; 63 Clinical Laboratory Department, Ghana Health Service, Kumasi, Ghana; 64 Department of Molecular Medicine, Kwame Nkrumah University of Science and Technology, Kumasi, Ghana; 65 Department of Parasitology, Mazandaran University of Medical Sciences, Sari, Iran; 66 Department of Parasitology, Iranshahr University of Medical Sciences, Iranshahr, Iran; 67 School of Community Health, Charles Sturt University, Orange, New South Wales, Australia; 68 Department of Psychology, Foundation University Islamabad, Rawalpandi, Pakistan; 69 Social Determinants of Health Research Center, Birjand University of Medical Sciences, Birjand, Iran; 70 Department of Public Health, Birmingham City University, Birmingham, England; 71 Department of Paediatrics, University of Malaya, Kuala Lumpur, Malaysia; 72 University of Malaya Medical Centre, University of Malaya, Kuala Lumpur, Malaysia; 73 Public Health and Healthcare Management, Tashkent Institute of Postgraduate Medical Education, Tashkent, Uzbekistan; 74 Boston Children's Hospital, Boston, Massachusetts; 75 Allied Health Sciences, Khyber Medical University, Timergara, Lower Dir, Pakistan; 76 Zayed University, Abu Dhabi, United Arab Emirates; 77 Department of Maternal and Child Health, Sultan Qaboos University, Muscat, Oman; 78 Research Center for Biochemistry and Nutrition in Metabolic Diseases, Kashan University of Medical Sciences, Kashan, Iran; 79 Department of Biostatistics and Epidemiology, Tabriz University of Medical Sciences, Tabriz, Iran; 80 Department of Biostatistics and Epidemiology, Zanjan University of Medical Sciences, Zanjan, Iran; 81 Institute of Radiological Sciences and Medical Imaging Technology, University of Lahore, Lahore, Pakistan; 82 School of Medicine and Public Health, University of Newcastle, Newcastle, New South Wales, Australia; 83 Hunter New England Population Health, Wallsend, New South Wales, Australia; 84 Northumbria HealthCare National Health Service (NHS) Foundation Trust, NHS England, Newcastle upon Tyne, England; 85 School of Business, University of Leicester, Leicester, England; 86 Department of Statistics and Econometrics, Bucharest University of Economic Studies, Bucharest, Romania; 87 Department of Surgery, Addis Ababa University, Addis Ababa, Ethiopia; 88 The Judith Lumley Centre, La Trobe University, Melbourne, Victoria, Australia; 89 San Martin de Porres University, Lima, Peru; 90 Department of Nursing, Debre Markos University, Debre Markos, Ethiopia; 91 Department of Neurosurgery, Cairo University, Cairo, Egypt; 92 Noncommunicable Diseases Research Center, Tehran, Iran; 93 School of Medicine, Shahroud University of Medical Sciences, Shahroud, Iran; 94 Department of Environmental Health Engineering, Hamadan University of Medical Sciences, Hamadan, Iran; 95 Faculty of Medicine, October 6 University, 6th of October City, Egypt; 96 Department of Forensic Science, Government Institute of Forensic Science, Nagpur, India; 97 Department of Parasitology, Tarbiat Modares University, Tehran, Iran; 98 Department of Parasitology, Alborz University of Medical Sciences, Karaj, Iran; 99 Unit of Biochemistry, Universiti Sultan Zainal Abidin, Kuala Terengganu, Malaysia; 100 Center for Clinical Research and Prevention, Bispebjerg University Hospital, Frederiksberg, Denmark; 101 Department of Medical Microbiology, Haramaya University, Harar, Ethiopia; 102 Department of Hypertension, Medical University of Lodz, Lodz, Poland; 103 Polish Mothers' Memorial Hospital Research Institute, Lodz, Poland; 104 Heidelberg Institute of Global Health, Heidelberg University, Heidelberg, Germany; 105 T.H. Chan School of Public Health, Harvard University, Boston, Massachusetts; 106 Department of Translational Medicine, University of Eastern Piedmont, Novara, Italy; 107 Academic Unit of Obstetrics and Gynecology, University of Genoa, Genoa, Italy; 108 Department of Public and Environmental Health, University of the Gambia, Brikama, The Gambia; 109 Epidemiology and Disease Control Unit, Ministry of Health, Kotu, The Gambia; 110 Social Determinants of Health Research Center, Lorestan University of Medical Sciences, Khorramabad, Iran; 111 Department of Oral Pathology and Microbiology, Krishna Institute of Medical Sciences, Karad, India; 112 University of Tasmania, Tasmania, Victoria, Australia; 113 Bahir Dar University, Bahir Dar, Ethiopia; 114 Department of Internal Medicine, Bahir Dar University, Bahir Dar, Ethiopia; 115 One Health, University of Nantes, Nantes, France; 116 Department of Forensic Chemistry, Government Institute of Forensic Science, Aurangabad, India; 117 Department of Social and Clinical Pharmacy, Charles University, Hradec Kralova, Czech Republic; 118 Institute of Public Health, United Arab Emirates University, Al Ain, United Arab Emirates; 119 Department of Anatomy, Government Medical College Pali, Pali, India; 120 Department of Community Medicine and Family Medicine, All India Institute of Medical Sciences, Jodhpur, India; 121 School of Public Health, All India Institute of Medical Sciences, Jodhpur, India; 122 Neurovascular Imaging Laboratory, New South Wales Brain Clot Bank, Sydney, New South Wales, Australia; 123 Department of Neurology and Neurophysiology, South West Sydney Local Heath District and Liverpool Hospital, Sydney, New South Wales, Australia; 124 Department of Statistical and Computational Genomics, National Institute of Biomedical Genomics, Kalyani, India; 125 Department of Statistics, University of Calcutta, Kolkata, India; 126 Department of Anatomy, Manipal University College Melaka, Melaka, Malaysia; 127 Institute of Soil and Environmental Sciences, University of Agriculture, Faisalabad, Faisalabad, Pakistan; 128 Social Determinants of Health Research Center, Babol University of Medical Sciences, Babol, Iran; 129 Department of General Surgery and Medical-Surgical Specialties, University of Catania, Catania, Italy; 130 Department of Global Public Health and Primary Care, University of Bergen, Bergen, Norway; 131 Cancer Registry of Norway, Oslo, Norway; 132 Department of Radiation Medicine, Oregon Health and Science University, Portland; 133 McGovern Medical School, University of Texas, Houston; 134 School of Physics, Engineering and Computer Science, University of Hertfordshire, Hatfield, England; 135 Institute for Women's Health, University College London, London, England; 136 Department of Public Health Medicine, University of KwaZulu-Natal, Durban, South Africa; 137 Department of Biomedical Sciences, Nazarbayev University, Nur-Sultan City, Kazakhstan; 138 Department of Epidemiology, University of Florida, Gainesville; 139 Cancer Population Sciences Program, University of Florida Health Cancer Center, Gainesville; 140 Department of Surgery, University of Toronto, Toronto, Ontario, Canada; 141 Division of Clinical Epidemiology and Aging Research, German Cancer Research Center, Heidelberg, Germany; 142 Department of Public Health, Federal University of Juiz de Fora, Juiz de Fora, Brazil; 143 Department of Family and Community Medicine, King Abdulaziz University, Jeddah, Saudi Arabia; 144 School of Public Health and Health Systems, University of Waterloo, Waterloo, Ontario, Canada; 145 Al Shifa School of Public Health, Al Shifa Trust Eye Hospital, Rawalpindi, Pakistan; 146 Institute of Microengineering, Federal Polytechnic School of Lausanne, Lausanne, Switzerland; 147 Department of Surgery, Washington University in St Louis, St Louis, Missouri; 148 Institute for Cancer Research, Prevention and Clinical Network, Florence, Italy; 149 National School of Public Health, Institute of Health Carlos III, Madrid, Spain; 150 Clinical Epidemiology Program, Ottawa Hospital Research Institute, Ottawa, Ontario, Canada; 151 Department of Nutrition, Federal University of Santa Catarina, Florianópolis, Brazil; 152 Mary MacKillop Institute for Health Research, Australian Catholic University, Melbourne, Victoria, Australia; 153 School of Public Health, University of Hong Kong, Hong Kong, China; 154 Regional Epidemiological Observatory Department, Sicilian Regional Health Authority, Palermo, Italy; 155 Hospitalist Department, Geisinger Health System, Danville, Pennsylvania; 156 Department of Public Health, Texila American University, Georgetown, Guyana; 157 Department of Medicine, University of Toronto, Toronto, Ontario, Canada; 158 Saveetha Medical College, Saveetha University, Chennai, India; 159 Center for Cancer Epidemiology, Tata Memorial Hospital, Navi Mumbai, India; 160 Department of Head Neck Surgery, Tata Memorial Hospital, Mumbai, India; 161 Department of Public Health and Health Policy, Hiroshima University, Hiroshima, Japan; 162 Division of Plastic Surgery, University of Washington, Seattle; 163 Department of Pulmonary Medicine, Christian Medical College and Hospital, Vellore, India; 164 Center for Biomedicine and Community Health, VNU International School, Hanoi, Vietnam; 165 Department of Otolaryngology, Wayne State University, Detroit, Michigan; 166 Nova Medical School, Nova University of Lisbon, Lisbon, Portugal; 167 Department of Public Health, Pontifical Catholic University of Chile, Santiago, Chile; 168 Research Line in Environmental Exposures and Health Effects at Population Level, Centro de Desarrollo Urbano Sustentable, Santiago, Chile; 169 School of Medicine and Surgery, University of Milan Bicocca, Monza, Italy; 170 Research Unit on Applied Molecular Biosciences, University of Porto, Porto, Portugal; 171 Faculty of Dentistry, Federal University of Rio Grande do Sul, Porto Alegre, Brazil; 172 School of Public Health, Walailak University, Nakhon Si Thammarat, Thailand; 173 Graduate School of Medicine, Kyoto University, Kyoto, Japan; 174 Department of Nursing, Bahir Dar University, Bahir Dar, Ethiopia; 175 Environmental Health Department, Imam Abdulrahman Bin Faisal University, Dammam, Saudi Arabia; 176 Department of Health Metrics Sciences, School of Medicine, University of Washington, Seattle; 177 Public Health Foundation of India, Gurugram, India; 178 Indian Council of Medical Research, New Delhi, India; 179 Department of Information Technology, University of Human Development, Sulaymaniyah, Iraq; 180 Institute for Research and Innovation in Health, University of Porto, Porto, Portugal; 181 Institute of Biomedical Engineering, University of Porto, Porto, Portugal; 182 Department of Public Health, National University of Colombia, Bogota, Colombia; 183 Department of Nursing, Woldia University, Woldia, Ethiopia; 184 School of Nursing, Jimma University, Jimma, Ethiopia; 185 Center for Nutrition and Health Research, National Institute of Public Health, Cuernavaca, Mexico; 186 Department of Community Medicine, Dr Baba Saheb Ambedkar Medical College and Hospital, Delhi, India; 187 Policy Research Institute, Kathmandu, Nepal; 188 Global Institute for Interdisciplinary Studies, Kathmandu, Nepal; 189 Health Research Section, Nepal Health Research Council, Kathmandu, Nepal; 190 Department of Epidemiology and Biostatistics, Shahroud University of Medical Sciences, Shahroud, Iran; 191 Department of Epidemiology, Shiraz University of Medical Sciences, Shiraz, Iran; 192 Center of Complexity Sciences, National Autonomous University of Mexico, Mexico City, Mexico; 193 Faculty of Veterinary Medicine and Zootechnics, Autonomous University of Sinaloa, Culiacán Rosales, Mexico; 194 Development of Research and Technology Center, Ministry of Health and Medical Education, Tehran, Iran; 195 Center of Excellence in Behavioral Medicine, Nguyen Tat Thanh University, Ho Chi Minh City, Vietnam; 196 School of Health, Guilan University of Medical Sciences, Rasht, Iran; 197 Department of Community Nutrition, Shahid Beheshti University of Medical Sciences, Tehran, Iran; 198 Department of Medical Laboratory Sciences, Iran University of Medical Sciences, Tehran, Iran; 199 Epidemiology and Data Analysis Laboratory, University Center Faculdade de Medicina do ABC, Santo André, Brazil; 200 Sydney School of Public Health, University of Sydney, Sydney, New South Wales, Australia; 201 Noncommunicable Diseases Research Center, Tehran University of Medical Sciences, Tehran, Iran; 202 Liver and Pancreaticobilliary Disease Research Center, Tehran University of Medical Sciences, Tehran, Iran; 203 Division of Urology, Children's Hospital of Philadelphia, Philadelphia, Pennsylvania; 204 Pediatric Dentistry and Dental Public Health Department, Alexandria University, Alexandria, Egypt; 205 Direction de L'épidémiologie et la Lutte Contre les Maladies, Ministry of Health, Rabat, Morocco; 206 Neurophysiology Department, Cairo University, Cairo, Egypt; 207 Faculty of Medicine, University of Tripoli, Tripoli, Libya; 208 Department of Neurology, Cairo University, Cairo, Egypt; 209 Preventive Medicine and Public Health Research Center, Iran University of Medical Sciences, Tehran, Iran; 210 Multiple Sclerosis Research Center, Tehran University of Medical Sciences, Tehran, Iran; 211 Department of Public Health, Maragheh University of Medical Sciences, Maragheh, Iran; 212 Division of Cancer Epidemiology and Genetics, National Cancer Institute, Bethesda, Maryland; 213 Department of Virology, Pasteur Institute of Morocco, Casablanca, Morocco; 214 Department of Internal Medicine, Rochester General Hospital, Rochester, New York; 215 Department of Health Policy and Administration, University of the Philippines Manila, Manila, Philippines; 216 Department of Neurological Surgery, Northwestern University, Chicago, Illinois; 217 Department of Neurological Surgery, University of Washington, Seattle; 218 Human Biology Division, Fred Hutchinson Cancer Research Center, Seattle, Washington; 219 Department of Neurosciences, Rehabilitation, Ophthalmology, Genetics, Maternal and Child Health, University of Genoa, Genoa, Italy; 220 University Eye Clinic, University of Genoa, Genoa, Italy; 221 Psychiatry Department, Kaiser Permanente, Fontana, California; 222 School of Health Sciences, A.T. Still University, Mesa, Arizona; 223 Institute of Gerontological Health Services and Nursing Research, Ravensburg-Weingarten University of Applied Sciences, Weingarten, Germany; 224 James Cancer Hospital, Ohio State University, Columbus; 225 Department of Medical Parasitology, Abadan University of Medical Sciences, Abadan, Iran; 226 Faculty of Medicine, Abadan University of Medical Sciences, Abadan, Iran; 227 Department of Dermatology, Kobe University, Kobe, Japan; 228 Health Services Management Training Centre, Semmelweis University, Budapest, Hungary; 229 Department of Applied Social Sciences, Sapientia Hungarian University of Transylvania, Târgu-Mureş, Romania; 230 Department of Cardiovascular Medicine, Cleveland Clinic, Cleveland, Ohio; 231 Gillings School of Global Public Health, University of North Carolina Chapel Hill, Chapel Hill; 232 Community Medicine Department, Bayero University, Kano, Kano, Nigeria; 233 Department of Community Medicine, Aminu Kano Teaching Hospital, Kano, Nigeria; 234 Department of Environmental Health Sciences, Mario Negri Institute for Pharmacological Research, Milan, Italy; 235 National Health Service, London, England; 236 School of Psychology, Addis Ababa University, Addis Ababa, Ethiopia; 237 Department of Medical Surgical Nursing, Tabriz University of Medical Sciences, Tabriz, Iran; 238 Student Research Committee, Iran University of Medical Sciences, Tehran, Iran; 239 Research Group for Genomic Epidemiology, Technical University of Denmark, Copenhagen, Denmark; 240 Cancer Research Center, Shahid Beheshti University of Medical Sciences, Tehran, Iran; 241 Faculty of Allied Health Sciences, University of Lahore, Lahore, Pakistan; 242 Afro-Asian Institute, Lahore, Pakistan; 243 Discipline of Public Health Medicine, University of KwaZulu-Natal, Durban, South Africa; 244 Health, Behavior and Society, Jimma University, Jimma, Ethiopia; 245 National Institute for Health Research Global Health Research Unit on Global Surgery, University of Birmingham, Birmingham, England; 246 Health Systems and Policy Research, Indian Institute of Public Health, Gandhinagar, India; 247 Department of Genetics, Sana Institute of Higher Education, Sari, Iran; 248 Department of Oral Surgery and Pathology, Federal University of Minas Gerais, Belo Horizonte, Brazil; 249 Hudson College of Public Health, University of Oklahoma Health Sciences Center, Oklahoma City; 250 Department of Health and Social Affairs, Government of the Federated States of Micronesia, Palikir, Federated States of Micronesia; 251 Oncological Network, Prevention and Research Institute, Institute for Cancer Research, Prevention and Clinical Network, Florence, Italy; 252 Department of Respiratory Medicine, Hokkaido University, Sapporo, Japan; 253 Center for Environmental and Health Sciences, Hokkaido University, Sapporo, Japan; 254 Department of Biomedical and Biotechnological Sciences, University of Catania, Catania, Italy; 255 Department of Family and Community Medicine, University of Sulaimani, Sulaimani, Iraq; 256 Harrington Heart and Vascular Institute, Case Western Reserve University, Cleveland, Ohio; 257 Division of Cardiovascular Medicine, Ohio State University, Columbus; 258 Department of Paediatrics, National Cancer Institute, Maharagama, Sri Lanka; 259 Department of Public Health, Torrens University, Melbourne, Victoria, Australia; 260 School of Medicine, Deakin University, Geelong, Victoria, Australia; 261 Department of Clinical Medicine, Macquarie University, Sydney, New South Wales, Australia; 262 Department of Epidemiology and Psychosocial Research, Ramón de la Fuente Muñiz National Institute of Psychiatry, Mexico City, Mexico; 263 Department of Radiology and Radiological Science, Johns Hopkins University, Baltimore, Maryland; 264 School of Medicine, Tehran University of Medical Sciences, Tehran, Iran; 265 Department of Social and Public Health, Ohio University, Athens; 266 Department of Pharmacology, Shahid Beheshti University of Medical Sciences, Tehran, Iran; 267 Obesity Research Center, Shahid Beheshti University of Medical Sciences, Tehran, Iran; 268 Clinical Sciences Department, University of Sharjah, Sharjah, United Arab Emirates; 269 College of Medicine, University of Sharjah, Sharjah, United Arab Emirates; 270 Department of Family and Community Medicine, Arabian Gulf University, Manama, Bahrain; 271 University Institute of Public Health, University of Lahore, Lahore, Pakistan; 272 School of Health and Environmental Studies, Hamdan Bin Mohammed Smart University, Dubai, United Arab Emirates; 273 Research & Scientific Studies Unit, Jazan University, Jazan, Saudi Arabia; 274 Faculty of Medicine, Utrecht University, Utrecht, Netherlands; 275 Department of Radiology, University Medical Center Utrecht, Utrecht, Netherlands; 276 Research Unit, University of Barcelona, Barcelona, Spain; 277 Biomedical Research Networking Center for Mental Health Network, Barcelona, Spain; 278 Department of Zoology and Entomology, Al Azhar University, Cairo, Egypt; 279 Gastrointestinal and Liver Diseases Research Center, Guilan University of Medical Sciences, Rasht, Iran; 280 Caspian Digestive Disease Research Center, Guilan University of Medical Sciences, Rasht, Iran; 281 International Foundation for Dermatology, London, England; 282 St John's Institute of Dermatology, King's College London, London, England; 283 Institute of Pharmaceutical Sciences, University of Veterinary and Animal Sciences, Lahore, Pakistan; 284 Department of Pharmacy Administration and Clinical Pharmacy, Xian Jiaotong University, Xian, China; 285 Independent Consultant, Santa Clara, California; 286 Community-Oriented Nursing Midwifery Research Center, Shahrekord University of Medical Sciences, Shahrekord, Iran; 287 Departamento de Salud Oral, Universidad Autónoma de Manizales, Manizales, Colombia; 288 School of Business, London South Bank University, London, England; 289 Department of Applied Microbiology, Taiz University, Taiz, Yemen; 290 Department of Microbiology, Nankai University, Tianjin, China; 291 Kasturba Medical College, Mangalore, Manipal Academy of Higher Education, Manipal, India; 292 Social and Environmental Health Research, Nature Study Society of Bangladesh, Khulna, Bangladesh; 293 Department of Health Promotion and Community Health Sciences, Texas A&M University, College Station; 294 Department of Population Sciences, University of Dhaka, Dhaka, Bangladesh; 295 Student Research Committee, Tabriz University of Medical Sciences, Tabriz, Iran; 296 Pediatric Chronic Kidney Disease Research Center, Tehran University of Medical Sciences, Tehran, Iran; 297 Institute of Research and Development, Duy Tan University, Da Nang, Vietnam; 298 Department of Computer Science, University of Human Development, Sulaymaniyah, Iraq; 299 Internal Medicine Department, Carol Davila University of Medicine and Pharmacy, Bucharest, Romania; 300 Department of Legal Medicine and Bioethics, Carol Davila University of Medicine and Pharmacy, Bucharest, Romania; 301 Clinical Legal Medicine Department, National Institute of Legal Medicine Mina Minovici, Bucharest, Romania; 302 College of Science and Engineering, Hamad Bin Khalifa University, Doha, Qatar; 303 Faculty of Medicine of Tunis, University Tunis El Manar, Tunis, Tunisia; 304 Jockey Club School of Public Health and Primary Care, Chinese University of Hong Kong, Hong Kong, China; 305 Department of Preventive and Social Dentistry, Federal University of Rio Grande do Sul, Porto Alegre, Brazil; 306 School of Pharmaceutical Sciences, University of Science Malaysia, Penang, Malaysia; 307 Department of Biomolecular Sciences, University of Zakho, Zakho, Iraq; 308 Department of Occupational Safety and Health, China Medical University, Taichung, Taiwan; 309 Department of Public Health, University of Naples Federico II, Naples, Italy; 310 Department of Health Promotion and Education, University of Ibadan, Ibadan, Nigeria; 311 Pharmacoepidemiology Department, Sanofi, Cambridge, Massachusetts; 312 Division of Allergy and Infectious Diseases, University of Washington, Seattle; 313 Department of Community Medicine, University of Ibadan, Ibadan, Nigeria; 314 Department of Community Medicine, University College Hospital, Ibadan, Ibadan, Nigeria; 315 Faculty of Medicine, University of Belgrade, Belgrade, Serbia; 316 Department of Epidemiology, University of Kragujevac, Kragujevac, Serbia; 317 School of Pharmacy, Taipei Medical University, Taipei, Taiwan; 318 Faculty of Pharmacy, Ahmad Dahlan University, Yogyakarta, Indonesia; 319 H. Lee Moffitt Cancer Center and Research Institute, Tampa, Florida; 320 Department of Epidemiology and Preventive Medicine, Monash University, Melbourne, Victoria, Australia; 321 Institute for Physical Activity and Nutrition, Deakin University, Burwood, Victoria, Australia; 322 Sydney Medical School, University of Sydney, Sydney, New South Wales, Australia; 323 Department of Clinical Pharmacy, MAHSA University, Bandar Saujana Putra, Malaysia; 324 Department of General Surgery and Surgical-Medical Specialties, University of Catania, Catania, Italy; 325 Department of Health Services Research, University of Tsukuba, Tsukuba, Japan; 326 Department of Non-Communicable Disease Epidemiology, London School of Hygiene and Tropical Medicine, London, England; 327 Research and Development Unit, Biomedical Research Networking Center for Mental Health Network, Sant Boi de Llobregat, Spain; 328 Faculty of Medicine, University of Versailles Saint-Quentin-en-Yvelines, Montigny-le-Bretonneux, France; 329 Institute of Comparative Economic Studies, Hosei University, Tokyo, Japan; 330 Department of Global Health, Economics and Policy, University of Kragujevac, Kragujevac, Serbia; 331 Health Informatic Lab, Boston University, Boston, Massachusetts; 332 Department of Biochemistry, Government Medical College, Mysuru, India; 333 Urology Department, University of Florida, Jacksonville; 334 Department of Community Medicine, Banaras Hindu University, Varanasi, India; 335 Department of Ophthalmology, Heidelberg University, Heidelberg, Germany; 336 Beijing Institute of Ophthalmology, Beijing Tongren Hospital, Beijing, China; 337 Department of Community Medicine, Manipal Academy of Higher Education, Mangalore, India; 338 Institute of Family Medicine and Public Health, University of Tartu, Tartu, Estonia; 339 Minimally Invasive Surgery Research Center, Iran University of Medical Sciences, Tehran, Iran; 340 Department of Genetics, Kermanshah University of Medical Sciences, Kermanshah, Iran; 341 School of Management and Medical Informatics, Tabriz University of Medical Sciences, Tabriz, Iran; 342 Institute for Prevention of Non-communicable Diseases, Qazvin University of Medical Sciences, Qazvin, Iran; 343 Health Services Management Department, Qazvin University of Medical Sciences, Qazvin, Iran; 344 Dermatology Department, King Faisal University, Hofuf, Saudi Arabia; 345 Public Health Division, Society for Education, Action and Research in Community Health, Gadchiroli, India; 346 Manipal Academy of Higher Education, Manipal, India; 347 Department of Computer Science and Engineering, University of Kurdistan Hewler, Hewler, Iraq; 348 Save Sight Institute, University of Sydney, Sydney, New South Wales, Australia; 349 Sydney Eye Hospital, South Eastern Sydney Local Health District, Sydney, New South Wales, Australia; 350 Institute for Epidemiology and Social Medicine, University of Münster, Münster, Germany; 351 Department of Adult Health Nursing, Bahir Dar University, Bahir Dar, Ethiopia; 352 Medical Research Council/Chief Scientist Office Social and Public Health Sciences Unit, University of Glasgow, Glasgow, Scotland; 353 Surgery Research Unit, University of Oulu, Oulu, Finland; 354 Department of Molecular Medicine and Surgery, Karolinska Institute, Stockholm, Sweden; 355 Department of Surface Engineering, John Paul II Catholic University of Lublin, Lublin, Poland; 356 Drohobych Ivan Franko State Pedagogical University, Drohobych, Ukraine; 357 Department of Epidemiology and Biostatistics, University of Gondar, Gondar, Faroe Islands; 358 School of Science and Technology, University of Georgia, Tbilisi, Georgia; 359 Department of Diagnostic and Interventional Radiology, New Hospitals LTD, Tbilisi, Georgia; 360 Students Scientific Research Center, Tehran University of Medical Sciences, Tehran, Iran; 361 Department of Public Health, Jordan University of Science and Technology, Irbid, Jordan; 362 Department of Biophysics and Biochemistry, Baku State University, Baku, Azerbaijan; 363 Russian Institute for Advanced Study, Moscow State Pedagogical University, Moscow, Russia; 364 Department of Medical Microbiology and Immunology, United Arab Emirates University, Al Ain, United Arab Emirates; 365 Epidemiology Department, Jazan University, Jazan, Saudi Arabia; 366 Department of Population Science, Jatiya Kabi Kazi Nazrul Islam University, Mymensingh, Bangladesh; 367 Family Medicine Department, United Arab Emirates University, Al Ain, United Arab Emirates; 368 Primary Care Department, NHS North West London, London, England; 369 Department of Health Policy and Management, Seoul National University, Seoul, South Korea; 370 Institute of Health Policy and Management, Seoul National University, Seoul, South Korea; 371 National Hepatology and Tropical Medicine Research Institute, Cairo University, Cairo, Egypt; 372 Shahid Beheshti University of Medical Sciences, Tehran, Iran; 373 The Iranian Academy of Medical Sciences, Tehran, Iran; 374 Department of Preventive Medicine, Yonsei University, Seoul, South Korea; 375 School of Traditional Chinese Medicine, Xiamen University Malaysia, Sepang, Malaysia; 376 School of Health Sciences, Kristiania University College, Oslo, Norway; 377 Department of Global Community Health and Behavioral Sciences, Tulane University, New Orleans, Louisiana; 378 Department of Nursing and Health Promotion, Oslo Metropolitan University, Oslo, Norway; 379 Department of Health Economics and Social Security, Jagiellonian University Medical College, Krakow, Poland; 380 School of Population and Public Health, University of British Columbia, Vancouver, British Columbia, Canada; 381 Arthritis Research Canada, Richmond, British Columbia, Canada; 382 Microbiology & Molecular Cell Biology Department, Eastern Virginia Medical School, Norfolk; 383 Department of Internal and Pulmonary Medicine, Sheri Kashmir Institute of Medical Sciences, Srinagar, India; 384 Kasturba Medical College, Udupi, India; 385 Biomedical Research Networking Center for Mental Health Network, San Juan de Dios Sanitary Park, Sant Boi de Llobregat, Spain; 386 Catalan Institution for Research and Advanced Studies, Barcelona, Spain; 387 Faculty of Medicine, Gazi University, Ankara, Turkey; 388 University of Environment and Sustainable Development, Somanya, Ghana; 389 Department of Orthopaedics, Medanta Hospital, Lucknow, India; 390 Faculty of Health and Life Sciences, Coventry University, Coventry, England; 391 Department of Medicine, McMaster University, Hamilton, Ontario, Canada; 392 Department of Pediatric Oncology, Hacettepe University, Ankara, Turkey; 393 Department of Clinical Sciences and Community Health, University of Milan, Milan, Italy; 394 Department of Community and Family Medicine, University of Baghdad, Baghdad, Iraq; 395 Unit of Genetics and Public Health, Institute of Medical Sciences, Las Tablas, Panama; 396 Ministry of Health, Herrera, Panama; 397 Institute of Clinical Physiology, National Research Council, Pisa, Italy; 398 Pattern Recognition and Machine Learning Lab, Gachon University, Seongnam, South Korea; 399 School of Pharmacy, Monash University, Bandar Sunway, Malaysia; 400 School of Pharmacy, Taylor's University Lakeside Campus, Subang Jaya, Malaysia; 401 Office of Health Policy & Legislative Affairs, University of Texas, Galveston; 402 Graduate School of Public Health, Ajou University, Suwon-si, South Korea; 403 Asbestos Diseases Research Institute, University of Sydney, Sydney, New South Wales, Australia; 404 Faculty of Science, Universiti Brunei Darussalam, Bandar Seri Begawan, Brunei; 405 Department of Medical Oncology, Peking Union Medical College, Beijing, China; 406 Department of Health Promotion and Health Education, National Taiwan Normal University, Taipei, Taiwan; 407 Lerner Research Institute, Cleveland Clinic, Cleveland, Ohio; 408 Department of Quantitative Health Science, Case Western Reserve University, Cleveland, Ohio; 409 Laboratory for Process Engineering, Environment, Biotechnology and Energy, University of Porto, Porto, Portugal; 410 School of Health, Polytechnic Institute of Porto, Portugal; 411 Department of General Surgery, Liverpool University Hospitals NHS Foundation Trust, Liverpool, England; 412 Department of Surgery, University of Liverpool, Liverpool, England; 413 Ophthalmology Department, Ministry of Health and Population, Aswan, Egypt; 414 Department of Primary Care and Public Health, Imperial College London, London, England; 415 Mass Communication Department, University of Sharjah, Sharjah, United Arab Emirates; 416 Department of Ophthalmology, M M Joshi Eye Institute, Hubli, India; 417 Rabigh Faculty of Medicine, King Abdulaziz University, Jeddah, Saudi Arabia; 418 Faculty of Public Health, Universitas Airlangga, Surabaya, Indonesia; 419 Indonesian Public Health Association, Surabaya, Indonesia; 420 Department of Midwifery, Hamadan University of Medical Sciences, Hamadan, Iran; 421 National Centre for Disease Informatics and Research, Indian Council of Medical Research, Bengaluru, India; 422 Department of Health Services Research and Policy, London School of Hygiene and Tropical Medicine, London, England; 423 India Cancer Research Consortium, Indian Council of Medical Research, New Delhi, India; 424 Peru Country Office, United Nations Population Fund, Lima, Peru; 425 Forensic Medicine Division, Imam Abdulrahman Bin Faisal University, Dammam, Saudi Arabia; 426 Department of Reproductive Health and Population Studies, Bahir Dar University, Bahir Dar, Ethiopia; 427 Department of Orthopaedic Surgery, Menoufia University Faculty of Medicine, Shebin El-Kom, Egypt; 428 Clinical Microbiology and Parasitology Unit, Polyclinic “Dr. Zora Profozic”, Zagreb, Croatia; 429 University Centre Varazdin, University North, Varazdin, Croatia; 430 School of Public Health and Community Medicine, University of Gothenburg, Gothenburg, Sweden; 431 Center for Innovation in Medical Education, Pomeranian Medical University, Szczecin, Poland; 432 Pomeranian Medical University, Szczecin, Poland; 433 Department of Propedeutics of Internal Diseases and Arterial Hypertension, Pomeranian Medical University, Szczecin, Poland; 434 Woman-Mother-Child Department, Lausanne University Hospital, Lausanne, Switzerland; 435 Pacific Institute for Research and Evaluation, Calverton, Maryland; 436 School of Public Health, Curtin University, Perth, Western Australia, Australia; 437 Department of Medical Immunology, Tehran University of Medical Sciences, Tehran, Iran; 438 Department of Surgical Oncology, All India Institute of Medical Sciences, Jodhpur, India; 439 Biotechnology Research Center, Tabriz University of Medical Sciences, Tabriz, Iran; 440 Molecular Medicine Research Center, Tabriz University of Medical Sciences, Tabriz, Iran; 441 Department of Biology, Salahaddin University-Erbil, Erbil, Iraq; 442 Internal Medicine Department, King Saud University, Riyadh, Saudi Arabia; 443 Department of Information Technology, Lebanese French University, Erbil, Iraq; 444 Department of Anatomical Sciences, Zanjan University of Medical Sciences, Zanjan, Iran; 445 Health Systems and Policy Research Unit, Ahmadu Bello University, Zaria, Nigeria; 446 Department of Health Care Management, Technical University of Berlin, Berlin, Germany; 447 Oncology Department, Appalachian Regional Healthcare, Hazard, Kentucky; 448 Internal Medicine, University of Kentucky, Lexington; 449 Faculty of Life Sciences and Medicine, King's College London, London, England; 450 Clinical Epidemiology and Public Health Research Unit, Burlo Garofolo Institute for Maternal and Child Health, Trieste, Italy; 451 Department of Computer Science and Engineering, Pabna University of Science and Technology, Pabna, Bangladesh; 452 Department of Molecular Medicine, National Institute of Genetic Engineering and Biotechnology, Tehran, Iran; 453 Social Determinants of Health Research Center, Kurdistan University of Medical Sciences, Sanandaj, Iran; 454 Computer, Electrical, and Mathematical Sciences and Engineering Division, King Abdullah University of Science and Technology, Thuwal, Saudi Arabia; 455 University Hospital Center of Porto, University of Porto, Porto, Portugal; 456 Section of Plastic Surgery, University of Michigan School of Medicine, Ann Arbor; 457 Department of Clinical Biochemistry, Babol University of Medical Sciences, Babol, Iran; 458 Department of Clinical Biochemistry, Tarbiat Modares University, Tehran, Iran; 459 Department of Epidemiology and Biostatistics, Wuhan University, Wuhan, China; 460 College of Medicine and Public Health, Flinders University, Adeaide, South Australia, Australia; 461 Research and Analytics Department, Initiative for Financing Health and Human Development, Chennai, India; 462 Department of Research and Analytics, Bioinsilico Technologies, Chennai, India; 463 Department of Nephrology, Manipal Academy of Higher Education, Manipal, India; 464 Department of Education for Clinical Research, National Center for Child Health and Development, Tokyo, Japan; 465 Laboratory of Public Health Indicators Analysis and Health Digitalization, Moscow Institute of Physics and Technology, Dolgoprudny, Russia; 466 Experimental Surgery and Oncology Laboratory, Kursk State Medical University, Kursk, Russia; 467 Suraj Eye Institute, Nagpur, India; 468 Department of Pharmacy Practice, Imam Abdulrahman Bin Faisal University, Dammam, Saudi Arabia; 469 Discipline of Social and Administrative Pharmacy, University of Science, Malaysia, Penang, Malaysia; 470 Mysore Medical College and Research Institute, Government Medical College, Mysore, India; 471 Department of Disease Control and Environmental Health, Makerere University, Kampala, Uganda; 472 Pharmaceutical Outcomes and Policy Department, University of Florida, Gainesville; 473 Department of General Surgery, Carol Davila University of Medicine and Pharmacy, Bucharest, Romania; 474 Department of General Surgery, Emergency Hospital of Bucharest, Bucharest, Romania; 475 Department of Oncology, Victor Babes University of Medicine and Pharmacy, Timisoara, Romania; 476 Estia Health Blakehurst, Estia Health, Sydney, New South Wales, Australia; 477 Institute for Global Health Innovations, Duy Tan University, Hanoi, Vietnam; 478 International Islamic University Islamabad, Islamabad, Pakistan; 479 South African Medical Research Council, Cape Town, South Africa; 480 School of Public Health and Family Medicine, University of Cape Town, Cape Town, South Africa; 481 Medical Research Council Clinical Trials Unit, University College London, London, England; 482 Department of Gastroenterology, Cambridge University Hospitals, Cambridge, England; 483 Unit of Microbiology and Public Health, Institute of Medical Sciences, Las Tablas, Panama; 484 Department of Public Health, Ministry of Health, Herrera, Panama; 485 Center of Excellence in Reproductive Health Innovation, University of Benin, Benin City, Nigeria; 486 Administrative and Economic Sciences Department, University of Bucharest, Bucharest, Romania; 487 Department of International Cyber Education, Mongolian National University of Medical Sciences, Ulaanbaatar, Mongolia; 488 Advisory Board, Ministry of Health, Ulaanbaatar, Mongolia; 489 Department of Community Health and Primary Care, University of Lagos, Idi Araba, Nigeria; 490 Department of Family and Preventive Medicine, University of Utah, Salt Lake City; 491 Translational Health Research Institute, Western Sydney University, Sydney, New South Wales, Australia; 492 Department of Psychiatry and Behavioural Neurosciences, McMaster University, Hamilton, Ontario, Canada; 493 Department of Psychiatry, University of Lagos, Lagos, Nigeria; 494 Community Prevention and Care Services, National AIDS Control Committee, Abuja, Nigeria; 495 Mass Communication Department, Ajman University, Dubai, United Arab Emirates; 496 Diplomacy and Public Relations Department, University of Human Development, Sulaymaniyah, Iraq; 497 Department of Anatomic Pathology, Ekiti State University, Ado-Ekiti, Nigeria; 498 Department of Anatomic Pathology, Ekiti State University Teaching Hospital, Ado-Ekiti, Nigeria; 499 Noncommunicable Disease Prevention Unit, Ministry of Health, Bandar Seri Begawan, Brunei; 500 Early Detection and Cancer Prevention Services, Pantai Jerudong Specialist Centre, Bandar Seri Begawan, Brunei; 501 Department of Pharmacology and Therapeutics, University of Nigeria Nsukka, Enugu, Nigeria; 502 Section of Sustainable Health, Umeå University, Umea, Sweden; 503 Health Systems Research Center, National Institute of Public Health, Cuernavaca, Mexico; 504 Department of Project Management, National Research University Higher School of Economics, Moscow, Russia; 505 Department of Medicine, University of Ibadan, Ibadan, Nigeria; 506 Department of Medicine, University College Hospital, Ibadan, Ibadan, Nigeria; 507 Department of Respiratory Medicine, Jagadguru Sri Shivarathreeswara Academy of Health Education and Research, Mysore, India; 508 Department of Medical Mycology and Parasitology, Shiraz University of Medical Sciences, Shiraz, Iran; 509 Department of Health Metrics, Center for Health Outcomes and Evaluation, Bucharest, Romania; 510 Department of Nutrition and Dietetics, Harokopio University, Athens, Greece; 511 Vision and Eye Research Institute, Anglia Ruskin University, Cambridge, England; 512 Institute of Health Services Research, Yonsei University, Seoul, South Korea; 513 Department of Medical Humanities and Social Medicine, Kosin University, Busan, South Korea; 514 Iran University of Medical Sciences, Tehran, Iran; 515 Department of Internal Medicine, Ochsner Medical Center, New Orleans, Louisiana; 516 Department of Epidemiology, Human Genetics and Environmental Sciences, The University of Texas Health Science Center at Houston School of Public Health, Dallas; 517 Department of Epidemiology, University of Arkansas for Medical Sciences, Little Rock; 518 Centre of Excellence, Khallikote University, Berhampur, India; 519 Research Division, Association for Biodiversity Conservation and Research, Balasore, India; 520 Department of Health Policy, Manipal Academy of Higher Education, Manipal, India; 521 Research Section, Nepal Health Research Council, Kathmandu, Nepal; 522 Faculty of Humanities and Social Sciences, Tribhuvan University, Kathmandu, Nepal; 523 Associated Laboratory for Green Chemistry, University of Porto, Porto, Portugal; 524 Department of Chemistry, University of Porto, Porto, Portugal; 525 Department of Development Studies, International Institute for Population Sciences, Mumbai, India; 526 Basic Medical Sciences Department, Durban University of Technology, Durban, South Africa; 527 Department of Biochemistry and Dietetics, Tabriz University of Medical Sciences, Tabriz, Iran; 528 Urooncology Research Center, Tehran University of Medical Sciences, Tehran, Iran; 529 Department of Dermatology, College of Medicine and Sagore Dutta Hospital, Kolkata, India; 530 University Medical Center Groningen, University of Groningen, Groningen, Netherlands; 531 School of Economics and Business, University of Groningen, Groningen, Netherlands; 532 Department of Nutrition and Food Sciences, Maragheh University of Medical Sciences, Maragheh, Iran; 533 Dietary Supplements and Probiotic Research Center, Alborz University of Medical Sciences, Karaj, Iran; 534 Department of Biochemistry, Jagadguru Sri Shivarathreeswara University, Mysuru, India; 535 National Institute of Infectious Diseases, Bucuresti, Romania; 536 Department of Infectious Diseases, Carol Davila University of Medicine and Pharmacy, Bucharest, Romania; 537 Biomedical Engineering Department, Amirkabir University of Technology, Tehran, Iran; 538 Department of Physics, Sharif University of Technology, Tehran, Iran; 539 College of Medicine, University of Central Florida, Orlando; 540 Manipal College of Dental Sciences, Manipal Academy of Higher Education, Manipal, India; 541 Department of Medical Oncology, Cancer Institute, Chennai, India; 542 Department of Medicine, University of Alberta, Edmonton, Alberta, Canada; 543 Metabolomics and Genomics Research Center, Tehran University of Medical Sciences, Tehran, Iran; 544 Department of Natural Science, Middlesex University, London, England; 545 Department of Population Science and Human Resource Development, University of Rajshahi, Rajshahi, Bangladesh; 546 School of Nursing and Healthcare Professions, Federation University Australia, Berwick, Victoria, Australia; 547 School of Nursing and Midwifery, La Trobe University, Melbourne, Victoria, Australia; 548 Future Technology Research Center, National Yunlin University of Science and Technology, Yunlin, Taiwan; 549 Department of Internal Medicine, Harvard University, Boston, Massachusetts; 550 Department of Surgery, University of Texas Health Science Center at San Antonio, San Antonio; 551 European Office for the Prevention and Control of Noncommunicable Diseases, World Health Organization, Moscow, Russia; 552 Department of Cardiology, Emory University, Atlanta, Georgia; 553 Health Emergency Operation Center, Ministry of Health and Population, Kathmandu, Nepal; 554 Central Department of Public Health, Institute of Medicine, Kathmandu, Nepal; 555 Department of Pharmacology, University of Colombo, Colombo, Sri Lanka; 556 Department of Community Medicine, Manipal Academy of Higher Education, Manipal, India; 557 Department of Oral Pathology, Srinivas Institute of Dental Sciences, Mangalore, India; 558 Department of Computer Science, Boston University, Boston, Massachusetts; 559 Department of Immunology and Laboratory Sciences, Sirjan School of Medical Sciences, Sirjan, Iran; 560 Department of Immunology, Kerman University of Medical Sciences, Kerman, Iran; 561 School of Medicine, Western Sydney University, Campbelltown, New South Wales, Australia; 562 Translational Health Research Institute, Western Sydney University, Campbelltown, New South Wales, Australia; 563 Endocrinology and Metabolism Research Center, Tehran University of Medical Sciences, Tehran, Iran; 564 Network of Immunity in Infection, Malignancy and Autoimmunity, Universal Scientific Education and Research Network, Tehran, Iran; 565 Dana-Farber Cancer Institute, Boston, Massachusetts; 566 Deparment of Pharmacology and Toxicology, University of Antioquia, Medellin, Colombia; 567 Department of Global Health and Population, Harvard University, Boston, Massachusetts; 568 Center for Indigenous Health Research, Wuqu' Kawoq Maya Health Alliance, Tecpan, Guatemala; 569 Maurizio Bufalini Hospital, Cesena, Italy; 570 Golestan Research Center of Gastroenterology and Hepatology, Golestan University of Medical Sciences, Gorgan, Iran; 571 Department of Internal Medicine, University of Botswana, Gaborone, Botswana; 572 Oral and Maxillofacial Surgery, Jagadguru Sri Shivarathreeswara University, Mysore, India; 573 Sharjah Institute for Medical Research, University of Sharjah, Sharjah, United Arab Emirates; 574 Research and Development, Islamabad Diagnostic Center Pakistan, Islamabad, Pakistan; 575 Biological Production Development, National Institute of Health, Islamabad, Pakistan; 576 Applied Biomedical Research Center, Mashhad University of Medical Sciences, Mashhad, Iran; 577 Biotechnology Research Center, Mashhad University of Medical Sciences, Mashhad, Iran; 578 Department of Radiology, University of Southern California, Los Angeles; 579 Public Health and Community Medicine Department, Cairo University, Giza, Egypt; 580 Digestive Diseases Research Institute, Tehran University of Medical Sciences, Tehran, Iran; 581 Emergency Department, Brown University, Providence, Rhode Island; 582 Department of Entomology, Ain Shams University, Cairo, Egypt; 583 Department of Surgery, Marshall University, Huntington, West Virginia; 584 Department of Nutrition and Preventive Medicine, Case Western Reserve University, Cleveland, Ohio; 585 Department of Pediatrics, University Hospitals Rainbow Babies & Children's Hospital, Cleveland, Ohio; 586 Department of Pediatrics, Case Western Reserve University, Cleveland, Ohio; 587 School of Public Health and Health Management, University of Belgrade, Belgrade, Serbia; 588 Division of Plastic and Reconstructive Surgery, McMaster University, Hamilton, Ontario, Canada; 589 Colorectal Research Center, Iran University of Medical Sciences, Tehran, Iran; 590 Faculty of Health & Social Sciences, Bournemouth University, Bournemouth, England; 591 Department of Public Health Sciences, University of North Carolina at Charlotte, Charlotte; 592 Market Access, Bayer, Istanbul, Turkey; 593 Department of Health Sciences, Federal University of Santa Catarina, Araranguá, Brazil; 594 Department of Medical Statistics, Epidemiology and Medical Informatics, University of Zagreb, Zagreb, Croatia; 595 Department of Epidemiology and Prevention of Chronic Noncommunicable Diseases, Croatian Institute of Public Health, Zagreb, Croatia; 596 National Heart, Lung, and Blood Institute, National Institutes of Health, Rockville, Maryland; 597 Department of Radiology and Interventional Neuroradiology, Isfahan University of Medical Sciences, Isfahan, Iran; 598 Clinical Research Development Unit of Farshchian Heart Center, Hamedan University of Medical Sciences, Hamadan, Iran; 599 Independent Consultant, Karachi, Pakistan; 600 Department of Oral Health, Non-Communicable Diseases Research Center, Tehran, Iran; 601 Noncommunicable Diseases Committee, National Institute for Medical Research Developmen, Tehran, Iran; 602 Symbiosis Medical College for Women, Symbiosis International University, Pune, India; 603 University School of Management and Entrepreneurship, Delhi Technological University, Delhi, India; 604 Centre for Medical Informatics, University of Edinburgh, Edinburgh, Scotland; 605 Division of General Internal Medicine, Harvard University, Boston, Massachusetts; 606 Department of Obstetrics and Gynaecology, Manipal Academy of Higher Education, Mangalore, India; 607 Department of Biochemistry, Manipal University College Melaka, Melaka, Malaysia; 608 Department of Forensic Medicine, Manipal Academy of Higher Education, Mangalore, India; 609 University of Tokyo, Tokyo, Japan; 610 Cancer Research Center, Tehran University of Medical Sciences, Tehran, Iran; 611 Cancer Biology Research Center, Tehran University of Medical Sciences, Tehran, Iran; 612 Public Health Dentistry Department, Krishna Institute of Medical Sciences, Karad, India; 613 Clinical Immunology and Hematology, Sofiamed University Hospital, Sofia, Bulgaria; 614 Department of Genetics, Sofia University St. Kliment Ohridiski, Sofia, Bulgaria; 615 Department of Health Education and Health Promotion, Kermanshah University of Medical Sciences, Kermanshah, Iran; 616 School of Health, University of Technology Sydney, Sydney, New South Wales, Australia; 617 Department of Hematology-Oncology, Baystate Medical Center, Springfield, Massachusetts; 618 Department of Physical Education, Federal University of Santa Catarina, Florianópolis, Brazil; 619 School of Medicine, University of Alabama at Birmingham, Birmingham; 620 Medicine Service Department of Veterans Affairs, Birmingham, Alabama; 621 Department of Midwifery, Haramaya University, Harar, Ethiopia; 622 Department No. 16, Moscow Research and Practical Centre on Addictions, Moscow, Russia; 623 Therapeutic Department, Balashiha Central Hospital, Balashikha, Russia; 624 Asbestos Diseases Research Institute, Sydney, New South Wales, Australia; 625 Cochrane Iran Associate Centre, National Institute for Medical Research Development, Iranian Ministry of Health and Medical Education, Tehran, Iran; 626 Department of Radiology, University of Alabama at Birmingham, Birmingham; 627 Department of Medicine, Democritus University of Thrace, Alexandroupolis, Greece; 628 Schiller Institute, Boston College, Boston, Massachusetts; 629 Barcelona Institute for Global Health, Barcelona, Spain; 630 Nepal Health Research Council, Kathmandu, Nepal; 631 Department of Community Medicine, Ahmadu Bello University, Zaria, Nigeria; 632 Pediatric Services, King Hussein Cancer Center, Amman, Jordan; 633 Pediatrics, University of Jordan, Amman, Jordan; 634 Maternal and Child Health, Projahnmo Research Foundation, Dhaka, Bangladesh; 635 Department of Medical Oncology, The Oncology Institute “Prof Dr Ion Chiricuţă” Cluj-Napoca, Cluj-Napoca, Romania; 636 Department of Medical Oncology, Iuliu Hatieganu University of Medicine and Pharmacy, Cluj-Napoca, Romania; 637 Faculty of Health and Public Administration, Semmelweis University, Budapest, Hungary; 638 Department of Medicine, University of Valencia, Valencia, Spain; 639 Carlos III Health Institute, Biomedical Research Networking Center for Mental Health Network, Madrid, Spain; 640 Cancer Control Center, Osaka International Cancer Institute, Osaka, Japan; 641 Johns Hopkins University, Baltimore, Maryland; 642 Research Center for Molecular Medicine, Hamadan University of Medical Sciences, Hamadan, Iran; 643 Pathology Department, Alexandria University, Alexandria, Egypt; 644 Department of Surgery, National University of Singapore, Singapore, Singapore; 645 Department of Pathology, University of Texas, Galveston; 646 College of Public Health, Medical and Veterinary Sciences, James Cook University, Cairns, Queensland, Australia; 647 University of Gondar, Gondar, Ethiopia; 648 Department of Clinical Pharmacy, University of Gondar, Gondar, Ethiopia; 649 Department of Community and Family Medicine, Iran University of Medical Sciences, Tehran, Iran; 650 Pediatric Intensive Care Unit, King Saud University, Riyadh, Saudi Arabia; 651 School of Public Health, Mekelle University, Mekelle, Ethiopia; 652 Southgate Institute for Health and Society, Flinders University, Adelaide, South Australia, Australia; 653 School of Public Health, University of Adelaide, Adelaide, South Australia, Australia; 654 Division of Pediatric Gastroenterology, Case Western Reserve University, Cleveland, Ohio; 655 Department of Gastroenterology and Hepatology, Mayo Clinic, Scottsdale, Arizona; 656 Department of Endocrinology, Diabetes and Metabolism, Christian Medical College and Hospital, Vellore, India; 657 HIV/STI Surveillance Research Center and World Health Organization Collaborating Center for HIV Surveillance, Kerman University of Medical Sciences, Kerman, Iran; 658 Nutritional Epidemiology Research Team, National Institute for Health and Medical Research Institut national de la santé et de la recherche médicale, Paris, France; 659 Department of Health, Medicine and Human Biology, Sorbonne Paris Nord University, Bobigny, France; 660 Department of Pathology and Legal Medicine, University of São Paulo, Ribeirão Preto, Brazil; 661 Modestum LTD, London, England; 662 Institute for Risk Assessment Sciences, Utrecht University, Utrecht, Netherlands; 663 Department of Health Economics, Hanoi Medical University, Hanoi, Vietnam; 664 Department of Molecular Medicine and Pathology, University of Auckland, Auckland, New Zealand; 665 Clinical Hematology and Toxicology, Maurice Wilkins Centre, Auckland, New Zealand; 666 School of Public Health and Social Work, Queensland University of Technology, Brisbane, Queensland, Australia; 667 Health Informatics Department, Nursing and Midwifery Faculty, Hanoi Medical University, Ha Noi, Vietnam; 668 Department of Community Medicine, All India Institute of Medical Sciences, Nagpur, India; 669 Department of Epidemiology and Biostatistics, Haramaya University, Haramaya, Ethiopia; 670 Department of Allied Health Sciences, Iqra National University, Peshawar, Pakistan; 671 Pakistan Council for Science and Technology, Ministry of Science and Technology, Islamabad, Pakistan; 672 Department of Pediatric Cardiology, Rush University, Chicago, Illinois; 673 Kasturba Medical College, Manipal Academy of Higher Education, Mangalore, India; 674 Amity Institute of Biotechnology, Amity University Rajasthan, Jaipur, India; 675 Alzahra Teaching Hospital, Tabriz University of Medical Sciences, Tabriz, Iran; 676 Women's Reproductive Health Research Center, Tabriz University of Medical Sciences, Tabriz, Iran; 677 Clinical Cancer Research Center, Milad General Hospital, Tehran, Iran; 678 Department of Microbiology, Islamic Azad University, Tehran, Iran; 679 Department of Medical and Surgical Sciences and Advanced Technologies, University of Catania, Catania, Italy; 680 Department of Medical and Surgical Sciences, University of Bologna, Bologna, Italy; 681 Occupational Health Unit, Sant'Orsola Malpighi Hospital, Bologna, Italy; 682 Department of Health Care Administration and Economics, National Research University Higher School of Economics, Moscow, Russia; 683 Faculty of Information Technology, Ho Chi Minh City University of Technology, Ho Chi Minh City, Vietnam; 684 Department of Neurosurgery, Erasmus University Medical Center, Rotterdam, Netherlands; 685 Center for Experimental Microsurgery, Iuliu Hațieganu University of Medicine and Pharmacy, Cluj-Napoca, Romania; 686 Cultures, Societies and Global Studies, Integrated Initiative for Global Health, Northeastern University, Boston, Massachusetts; 687 School of Public Health, University of Nairobi, Nairobi, Kenya; 688 College of Medicine and Public Health, Flinders University, Adelaide, South Australia, Australia; 689 Key Laboratory of Shaanxi Province for Craniofacial Precision Medicine Research, Stomatological Hospital (College) of Xi'an Jiaotong University, Xi'an, China; 690 Competence Center of Mortality-Follow-Up of the German National Cohort, Federal Institute for Population Research, Wiesbaden, Germany; 691 Institute of Health and Society, University of Oslo, Oslo, Norway; 692 Department of Neurology, Technical University of Munich, Munich, Germany; 693 Adelaide Medical School, University of Adelaide, Adelaide, South Australia, Australia; 694 Research and Development Division, The George Institute for Global Health, New Delhi, India; 695 Cancer Epidemiology and Prevention Research, Alberta Health Services, Calgary, British Columbia, Canada; 696 Department of Oncology, University of Calgary, Calgary, Alberta, Canada; 697 School of International Development and Global Studies, University of Ottawa, Ottawa, Ontario, Canada; 698 George Institute for Global Health, University of Oxford, Oxford, England; 699 Department of Pharmacy, Debre Tabor University, Debre Tabor, Ethiopia; 700 Department of Epidemiology and Biostatistics, University of Gondar, Gondar, Ethiopia; 701 Department of Neuropsychopharmacology, National Center of Neurology and Psychiatry, Kodaira, Japan; 702 Department of Public Health, Juntendo University, Tokyo, Japan; 703 Department of Health Policy and Management, Jackson State University, Jackson, Mississippi; 704 School of Medicine, Tsinghua University, Beijing, China; 705 Department of Environmental Health, Mazandaran University of Medical Sciences, Sari, Iran; 706 Cancer Institute, Hacettepe University, Ankara, Turkey; 707 Department of Clinical Pharmacy and Outcomes Sciences, University of South Carolina, Columbia; 708 Epidemiology and Cancer Registry Sector, Institute of Oncology Ljubljana, Ljubljana, Slovenia; 709 Shahroud University of Medical Sciences, Shahroud, Iran; 710 Laboratory of Genetics and Genomics, Moscow Research and Practical Centre on Addictions, Moscow, Russia; 711 Addictology Department, Russian Medical Academy of Continuous Professional Education, Moscow, Russia; 712 Pediatrics Department, Russian Medical Academy of Continuous Professional Education, Moscow, Russia; 713 Department of General Practice, University of Melbourne, Melbourne, Victoria, Australia; 714 Victorian Comprehensive Cancer Centre, Melbourne, Victoria, Australia; 715 Bone Marrow Transplantation Center, Zhejiang University, Hangzhou, China; 716 Department of Epidemiology, Human Genetics, and Environmental Sciences, University of Texas Health Science Center at Houston, Houston; 717 Departamento de Saúde Coletiva, Brasília University, Brasilia, Brazil; 718 Division of Hematology, University of Washington, Seattle; 719 Division of Pediatric Hematology-Oncology, University of Washington, Seattle

## Abstract

**Importance:**

The Global Burden of Diseases, Injuries, and Risk Factors Study 2019 (GBD 2019) provided systematic estimates of incidence, morbidity, and mortality to inform local and international efforts toward reducing cancer burden.

**Objective:**

To estimate cancer burden and trends globally for 204 countries and territories and by Sociodemographic Index (SDI) quintiles from 2010 to 2019.

**Evidence Review:**

The GBD 2019 estimation methods were used to describe cancer incidence, mortality, years lived with disability, years of life lost, and disability-adjusted life years (DALYs) in 2019 and over the past decade. Estimates are also provided by quintiles of the SDI, a composite measure of educational attainment, income per capita, and total fertility rate for those younger than 25 years. Estimates include 95% uncertainty intervals (UIs).

**Findings:**

In 2019, there were an estimated 23.6 million (95% UI, 22.2-24.9 million) new cancer cases (17.2 million when excluding nonmelanoma skin cancer) and 10.0 million (95% UI, 9.36-10.6 million) cancer deaths globally, with an estimated 250 million (235-264 million) DALYs due to cancer. Since 2010, these represented a 26.3% (95% UI, 20.3%-32.3%) increase in new cases, a 20.9% (95% UI, 14.2%-27.6%) increase in deaths, and a 16.0% (95% UI, 9.3%-22.8%) increase in DALYs. Among 22 groups of diseases and injuries in the GBD 2019 study, cancer was second only to cardiovascular diseases for the number of deaths, years of life lost, and DALYs globally in 2019. Cancer burden differed across SDI quintiles. The proportion of years lived with disability that contributed to DALYs increased with SDI, ranging from 1.4% (1.1%-1.8%) in the low SDI quintile to 5.7% (4.2%-7.1%) in the high SDI quintile. While the high SDI quintile had the highest number of new cases in 2019, the middle SDI quintile had the highest number of cancer deaths and DALYs. From 2010 to 2019, the largest percentage increase in the numbers of cases and deaths occurred in the low and low-middle SDI quintiles.

**Conclusions and Relevance:**

The results of this systematic analysis suggest that the global burden of cancer is substantial and growing, with burden differing by SDI. These results provide comprehensive and comparable estimates that can potentially inform efforts toward equitable cancer control around the world.

## Key Points

**Question:**

What was the burden of cancer globally and across Sociodemographic Index (SDI) groupings in 2019, and how has incidence, morbidity, and mortality changed since 2010?

**Findings:**

In this systematic analysis, there were 23.6 million new global cancer cases in 2019 (17.2 million when excluding those with nonmelanoma skin cancer), 10.0 million cancer deaths, and an estimated 250 million disability-adjusted life years estimated to be due to cancer; since 2010, these represent increases of 26.3%, 20.9%, and 16.0%, respectively. Absolute cancer burden increased in all SDI quintiles since 2010, but the largest percentage increases occurred in the low and low-middle SDI quintiles.

**Meanings:**

The study results suggest that increased cancer prevention and control efforts are needed to equitably address the evolving and increasing burden of cancer across the SDI spectrum.

## Teaser

The Global Burden of Diseases, Injuries, and Risk Factors Study 2019 examines cancer burden and trends globally for 204 countries and territories and by Socio-demographic Index quintiles from 2010 to 2019.

## Introduction

Cancers are a major contributor to disease burden worldwide, and projections forecast that global cancer burden will continue to grow for at least the next 2 decades.^1,2,3,4^ The United Nations (UN) Sustainable Development Goals (SDGs) recognize the need for reducing cancer burden as part of target 3.4, stating “By 2030, reduce by one third premature mortality from noncommunicable diseases [NCDs] through prevention and treatment and promote mental health and well-being.”^5^ Most countries will need to accelerate their efforts to reduce NCD burden, including cancer, to meet this SDG target.^6,7^ Increasing the pace of progress will be particularly critical given the ongoing COVID-19 pandemic, which has led to delays and disruptions in cancer screenings, diagnosis, and treatment around the world.^8,9,10,11,12^

The importance of prevention and control of NCDs, including cancer, was emphasized by the third UN High-Level Meeting on NCDs in 2018^13^ and the UN High-Level Meeting on Universal Health Coverage in 2019.^14,15^ World Health Organization initiatives that are focused on breast cancer,^16^ cervical cancer,^17^ and childhood cancer^18^ are valuable efforts toward reducing global cancer burden in combination with national-level cancer control planning and implementation. Global and local efforts require comprehensive assessments of cancer burden, information that may be sparse or unavailable in some countries.^19^

The Global Burden of Diseases (GBD), Injuries, and Risk Factors Study 2019 (GBD 2019) framework enables the comparable assessment of cancer burden across locations and time in terms of cancer incidence, mortality, years of life lost (YLLs), years lived with disability (YLDs), and disability-adjusted life years (DALYs).^20^ Estimates of YLLs, YLDs, and DALYs complement incidence and mortality estimates by incorporating morbidity and mortality contributions to total cancer burden over the lifetime. Because GBD 2019 estimated disease burden across a mutually exclusive and collectively exhaustive hierarchy of diseases and injuries, cancer burden can also be systematically compared with and ranked against other causes of disease burden. Together, these qualities help GBD 2019 provide a comprehensive picture of variation in cancer burden that can potentially inform cancer control planning.

In this article, we present results for 29 cancer groups from the GBD 2019 study, globally and for 204 countries and territories, from 2010 through 2019. Results are also provided by quintiles of the Sociodemographic Index (SDI), a summary indicator of social and economic development that allows for analyses of disease burden patterns across different resource contexts.^20,21^ These estimates update results from the GBD 2017 study^22^ and supersede published estimates from previous GBD iterations.^22,23,24,25^

## Methods

This section provides an overview of GBD 2019 cancer estimation methods. Additional detail is provided in the GBD 2019 summary publications,^20,21,26^ as well as in the eAppendix, eFigures 1 to 15, and eTables 1 to 18 in the Supplement. This study is compliant with the Guidelines for Accurate and Transparent Health Estimates Reporting (GATHER) statement (eTable 13 in the Supplement).^27^ The University of Washington institutional review board committee approved GBD 2019, and informed consent was waived because of the use of deidentified data. This article was produced as part of the GBD Collaborator Network and in accordance with the GBD Protocol (http://www.healthdata.org/gbd/about/protocol).

### Study Design

Disease and injuries in GBD 2019 were organized into a comprehensive hierarchy of nested levels, with neoplasms as 1 of 22 level 2 groups.^20^ Cancers were classified into 30 level 3 cancer groups (eg, leukemia), 4 of which were further subdivided into level 4 groups (eg, chronic myeloid leukemia). While the GBD study estimates benign and in situ neoplasms as an important component of total health burden from all neoplasms broadly, this level 3 cancer group was not included in the estimates reported in this article to focus on malignant cancers (eAppendix in the Supplement). Similarly, because nonmelanoma skin cancer (NMSC) has relatively high incidence and low mortality compared with other cancers, this article presents estimates with and without NMSC.

There are 5 major ways that this iteration of the GBD study improved on the data and methods used to estimate cancer burden in GBD 2017^22^ (eAppendix in the Supplement). First, GBD 2019 incorporated an additional 104 076 new cancer-, location-, and year-specific sources of data compared with GBD 2017 (eTable 1 in the Supplement). Second, data processing methods were improved for several cancers, particularly liver cancer, as described later. Third, the youngest age group estimated was increased or decreased for several cancers to align with cancer registry age patterns. Fourth, modeling parameters were updated to perform additional smoothing of mortality-to-incidence ratio (MIR) estimates across age and time, reducing improbable variation from sparse data. Fifth, cancer survival estimation methods were updated to improve uncertainty estimations and estimate age-specific instead of all-ages survival curves.

Results are presented by SDI, a composite indicator of income per capita, mean years of education, and fertility rate for those younger than 25 years.^21^ The SDI is the geometric mean of these 3 independently estimated and scaled components, with lower values representing lower development. While SDI values may change over time, for consistency of comparison, countries were grouped into quintiles according to their SDI values in 2019 (eTable 2 and eFigure 1 in the Supplement). These quintiles were termed *low*, *low-middle*, *middle*, *high-middle*, and *high*. More details are provided in the eAppendix in the Supplement, including the population and SDI bounds for each quintile.

### Data Sources and Processing

Cancer estimation in GBD 2019 used 929 193 cancer-, location-, and year-specific sources of data, of which 767 514 (82.6%) were from vital registration systems, 155 542 (16.7%) from cancer registries, and 6137 (0.7%) from verbal autopsy reports (eTable 1 in the Supplement). The cancers presented in this analysis include *malignant neoplasms* or *cancer* as defined by the *International Statistical Classification of Diseases and Related Health Problems, Ninth Revision *(*ICD-9*) codes 140 to 209,^28^ or *Tenth Revision *(*ICD-10*) codes C00 to C96.^29^ Incidence and mortality data with these *ICD* codes are mapped to GBD cancer causes^20^ (eAppendix and eTables 3-5 in the Supplement). One processing update for GBD 2019 was the remapping of deaths coded to *ICD-10* code C22.9; because this code includes unspecified primary or secondary liver cancer, a subset of these deaths were redistributed to various other cancers that metastasize to the liver.^20,30,31^ Kaposi sarcoma was not estimated because deaths were primarily redistributed to be of HIV/AIDS (eAppendix in the Supplement).^20^ The GBD NMSC estimates included squamous cell carcinoma and basal cell carcinoma. Because NMSC reporting was incomplete in many cancer registries,^32^ GBD 2019 additionally incorporated data from the literature and clinical sources to estimate NMSC burden (eAppendix in the Supplement).

### Modeling Process

The GBD cancer mortality and YLL estimation process included 2 primary steps (eFigure 2 in the Supplement), beginning with the estimation of cancer MIRs, which provide an association between mortality and incidence estimation, maximizing data availability. The MIRs were modeled using a space-time Gaussian process regression approach^26^ (MIR methods are described in the eAppendix in the Supplement) using matched incidence and mortality data from cancer registries (eTable 6 in the Supplement) and the GBD-estimated health care access and quality index^33^ as a covariate. These estimated MIRs were then used to convert cancer registry incidence data into inputs for mortality modeling.

Estimating cancer mortality was the second step. The GBD 2019 study used a Cause of Death Ensemble model (CODEm) approach that combined data from vital registration systems, cancer registries, and verbal autopsy reports to estimate mortality across several submodels.^34^ Covariates provided for potential inclusion in the submodels of the ensemble, such as smoking prevalence or alcohol use, can be found in the eAppendix and eTables 7 and 8 in the Supplement. Ensemble model construction and performance was evaluated through out-of-sample predictive validity tests (eTable 9 in the Supplement). For each cancer, sex-specific CODEm models generated mortality estimates across locations, years, and age groups. These cancer mortality estimates were then scaled to align with the total mortality for all causes of death, which was separately estimated in GBD 2019 (eTable 10 in the Supplement).^21^ To estimate YLLs, a standard age-specific GBD life expectancy was applied to mortality estimates by age group (eAppendix in the Supplement).^20^

The GBD cancer incidence and YLD estimation process included 2 additional steps (eFigure 3 in the Supplement), starting with estimating incidence. Incidence was estimated by taking mortality estimates from the second step described previously and dividing by MIR estimates from the first step described previously for each cancer type, sex, location, year, and 5-year age group. Additional information can be found in the eAppendix in the Supplement.

Next, YLDs were estimated by combining prevalence estimates with disability weights associated with various phases of cancer survival. To estimate 10-year cancer prevalence, survival curves estimated from MIRs were combined with GBD-estimated background mortality and applied to incidence estimates. Additional information regarding survival and prevalence estimation can be found in the eAppendix and eFigure 3 in the Supplement. These 10-year prevalence estimates were then partitioned into 4 sequelae according to the expected person-time spent in these 4 phases of cancer survival: (1) diagnosis/treatment, (2) remission, (3) metastatic/disseminated, and (4) terminal (eTable 11 in the Supplement). Each sequela prevalence was multiplied by a sequela-specific disability weight that represented the magnitude of health loss (eTable 12 in the Supplement).^20^ For 5 cancer types (bladder, breast, colorectal, larynx, and prostate cancer), the total prevalence additionally included lifetime prevalence of procedure-related disability (eg, laryngectomy due to larynx cancer). These procedure-related prevalence estimates were modeled in the Bayesian meta-regression tool DisMod-MR, version 2.1,^20^ using medical records data on the proportion of patients with cancer who underwent these procedures and the estimated number of 10-year survivors (eAppendix in the Supplement). These procedure-related prevalence estimates were then multiplied by procedure-specific disability weights (eTable 12 in the Supplement). Total cancer-specific YLDs were estimated by summing across these sequelae. Finally, DALYs were estimated as the sum of YLDs and YLLs.^20^

### Reporting Standards

All rates are reported per 100 000 person-years. Annualized rates of change from 2010 to 2019 represent the mean percentage change per year during this period (eAppendix in the Supplement). The GBD world population standard was used to calculate age-standardized rates (eAppendix in the Supplement).^21^ For all estimates, 95% uncertainty intervals (UIs) are reported. Uncertainty was propagated through each step of the cancer estimation process, with UIs representing the 2.5th and 97.5th percentiles of the distribution of 1000 draws at each step (eAppendix in the Supplement).^20^

Results are reported for 29 cancer groups, 204 countries and territories, and 5 SDI quintiles from 2010 to 2019. These estimates, as well as extended years (1990-2019), additional cancer groups, national and subnational locations, sex-specific estimates, and additional age groups are available from online resources (https://vizhub.healthdata.org/gbd-compare/ and http://ghdx.healthdata.org/gbd-results-tool).

Data processing and analyses were conducted using Python, version 3.7.0 (Python Software Foundation); Stata, version 15.1 (StataCorp); and R, version 3.4.1 (R Foundation). Code is available at https://ghdx.healthdata.org/gbd-2019/code.

## Results

### Global Estimates of Total Cancers and Cancer-Specific Burden in 2019

Across 204 countries and territories, there were 23.6 million (95% UI, 22.2-24.9 million) incident cancer cases and 10.0 million (95% UI, 9.36-10.6 million) deaths in 2019 (Table 1). Excluding NMSC, there were an estimated 17.2 million (95% UI, 15.9-18.5 million) incident cancer cases and 9.97 million (95% CI, 9.31-10.5 million) deaths (Table 1).

**Table 1.** Global Incidence and Deaths in 2019 for Total Cancers and 29 Cancer Groups

Cancer type^a^	Deaths, thousands (95% UI)			ASMR per 100 000 (95% UI)			Incident cases, thousands (95% UI)			ASIR per 100 000 (95% UI)		
	Total	Male	Female	Total	Male	Female	Total	Male	Female	Total	Male	Female
Total	10 000 (9360-10 600)	5690 (5250-6100)	4340 (3970-4660)	124.7 (116.4-132.0)	156.1 (143.9-167.2)	99.9 (91.5-107.3)	23 600 (22 200-24 900)	12 900 (12 100-13 800)	10 600 (9920-11 400)	290.5 (274.0-307.1)	348.7 (327.3-370.8)	246.1 (229.8-263.1)
Excluding NMSC	9970 (9310-10 500)	5650 (5220-6 070)	4310 (3950-4 640)	123.9 (115.7-131.2)	155.1 (142.9-166.1)	99.4 (91.0-106.8)	17 200 (15 900-18 500)	9260 (8470-10 000)	7960 (7280-8610)	211.4 (195.4-226.8)	245.9 (225.3-266.5)	185.0 (169.4-200.2)
Tracheal, bronchus, and lung	2040 (1880-2190)	1390 (1260-1510)	657 (590-719)	25.2 (23.2-27.0)	37.4 (34.1-40.7)	15.0 (13.5-16.4)	2260 (2070-2450)	1520 (1370-1680)	737 (658-814)	27.7 (25.3-30.0)	40.4 (36.5-44.4)	16.8 (15.0-18.6)
Colon and rectum	1090 (1000-1150)	594 (551-638)	492 (438-532)	13.7 (12.6-14.5)	16.6 (15.4-17.9)	11.2 (10.0-12.2)	2170 (2000-2 340)	1240 (1130-1360)	926 (832-1 010)	26.7 (24.6-28.9)	33.1 (30.2-36.2)	21.2 (19.0-23.2)
Stomach	957 (871-1030)	612 (544-678)	346 (308-382)	11.9 (10.8-12.8)	16.6 (14.8-18.3)	7.9 (7.1-8.8)	1270 (1150-1400)	847 (748-963)	423 (377-467)	15.6 (14.1-17.2)	22.4 (19.8-25.3)	9.7 (8.7-10.7)
Breast	701 (647-752)	12.1 (10.7-13.3)	689 (635-740)	8.6 (7.9-9.2)	0.3 (0.3-0.4)	15.9 (14.7-17.1)	2000 (1830-2170)	25.1 (22.2-27.8)	1980 (1810-2150)	24.2 (22.1-26.2)	0.7(0.6-0.7)	45.9 (41.9-49.8)
Pancreatic	531 (492-567)	278 (258-299)	253 (226-274)	6.6 (6.1-7.1)	7.5 (7.0-8.1)	5.8 (5.1-6.2)	530 (486-574)	280 (256-303)	250 (224-275)	6.6 (6.0-7.1)	7.5 (6.8-8.1)	5.7 (5.1-6.3)
Esophageal	498 (438-551)	366 (315-415)	133 (110-150)	6.1 (5.4-6.8)	9.7 (8.3-11.0)	3.0 (2.5-3.4)	535 (467-595)	389 (336-444)	146 (120-165)	6.5 (5.7-7.2)	10.1 (8.7-11.6)	3.3 (2.7-3.8)
Prostate	487 (420-594)	487 (420-594)	NA	6.3 (5.4-7.7)	15.3 (13.0-18.6)	NA	1410 (1230-1830)	1410 (1230-1830)	NA	17.4 (15.1-22.5)	38.6 (33.6-49.8)	NA
Liver	485 (444-526)	334 (300-368)	151 (134-167)	5.9 (5.4-6.4)	8.7 (7.9-9.6)	3.5 (3.1-3.8)	534 (487-589)	376 (335-422)	158 (140-176)	6.5 (5.9-7.2)	9.7 (8.7-10.8)	3.6 (3.2-4.0)
Other malignant neoplasms	408 (355-444)	220 (180-249)	188 (169-204)	5.1 (4.5-5.6)	5.9 (4.8-6.7)	4.5 (4.0-4.8)	831 (741-906)	451 (381-504)	381 (347-415)	10.4 (9.3-11.4)	11.9 (10.0-13.3)	9.2 (8.4-10.1)
Leukemia	335 (307-360)	188 (165-208)	146 (132-158)	4.3 (3.9-4.6)	5.2 (4.6-5.7)	3.5 (3.2-3.8)	644 (587-700)	351 (308-390)	293 (263-322)	8.2 (7.5-8.9)	9.4 (8.3-10.5)	7.2 (6.5-8.0)
Cervical	280 (239-314)	NA	280 (239-314)	3.4 (2.9-3.8)	NA	6.5 (5.5-7.3)	566 (482-636)	NA	566 (482-636)	6.8 (5.8-7.7)	NA	13.4 (11.4-15.0)
Non-Hodgkin lymphoma	255 (238-270)	146 (136-155)	109 (98.9-117)	3.2 (3.0-3.4)	4.0 (3.7-4.2)	2.5 (2.3-2.7)	457 (417-499)	266 (241-291)	191 (169-211)	5.7 (5.2-6.3)	7.2 (6.5-7.9)	4.5 (4.0-4.9)
Brain and central nervous system	246 (186-271)	139 (99.6-157)	108 (76.4-122)	3.0 (2.3-3.4)	3.6 (2.6-4.1)	2.6 (1.8-2.9)	348 (262-389)	187 (135-215)	161 (114-184)	4.3 (3.3-4.9)	4.8 (3.5-5.6)	3.9 (2.8-4.5)
Bladder	229 (211-243)	169 (157-181)	59.5 (52.3-64.6)	2.9 (2.7-3.1)	5.1 (4.7-5.4)	1.4 (1.2-1.5)	524 (476-569)	408 (371-444)	116 (104-128)	6.5 (5.9-7.1)	11.3 (10.2-12.3)	2.7 (2.4-2.9)
Lip and oral cavity	199 (182-218)	132 (118-145)	67.8 (60.8-75.7)	2.4 (2.2-2.7)	3.4 (3.1-3.8)	1.6 (1.4-1.7)	373 (341-404)	243 (219-268)	130 (117-143)	4.5 (4.1-4.9)	6.2 (5.6-6.8)	3.0 (2.7-3.3)
Ovarian	198 (175-218)	NA	198 (175-218)	2.4 (2.1-2.7)	NA	4.6 (4.0-5.0)	294 (261-330)	NA	294 (261-330)	3.9 (3.2-4.0)	NA	6.9 (6.1-7.7)
Gallbladder and biliary tract	172 (145-189)	73.0 (59.5-80.4)	99.5 (81.7-114.0)	2.2 (1.8-2.4)	2.1 (1.7-2.3)	2.3 (1.9-2.6)	199 (167-220)	86.4 (69.4-95.9)	113 (91.6-130)	2.5 (2.1-2.7)	2.4 (1.9-2.7)	2.6 (2.1-3.0)
Kidney	166 (155-176)	109 (101-116)	57.7 (52.2-61.9)	2.1 (1.9-2.2)	3.0 (2.8-3.2)	1.3 (1.2-1.4)	372 (345-402)	241 (221-262)	131 (120-142)	4.6 (4.2-4.9)	6.2 (5.7-6.8)	3.1 (2.8-3.3)
Larynx	123 (115-133)	106 (97.8-115)	17.8 (16.2-19.7)	1.5 (1.4-1.6)	2.7 (2.5-3.0)	0.4 (0.4-0.5)	209 (194-225)	181 (166-196)	28.5 (26.1-31.3)	2.5 (2.3-2.7)	4.6 (4.2-5.0)	0.7 (0.6-0.7)
Other pharynx	114 (103-126)	88.0 (78.0-98.7)	26.2 (22.5-30.5)	1.4 (1.2-1.5)	2.2 (2.0-2.5)	0.6 (0.5-0.7)	167 (153-180)	129 (116-142)	37.6 (33.1-42.3)	2.0 (1.8-2.2)	3.2 (2.9-3.5)	0.9 (0.8-1.0)
Multiple myeloma	113 (99.5-122)	60.4 (50.7-67.1)	53.0 (45.1-58.3)	1.4 (1.2-1.5)	1.7 (1.4-1.8)	1.2 (1.0-1.3)	156 (137-173)	84.5 (70.9-94.9)	71.2 (60.3-80.1)	1.9 (1.7-2.1)	2.3 (1.9-2.6)	1.6 (1.4-1.8)
Uterine	91.6 (82.4-101.5)	NA	91.6 (82.4-101.5)	1.1 (1.0-1.3)	NA	2.1 (1.9-2.3)	435 (397-480)	NA	435 (397-480)	5.2 (4.8-5.7)	NA	10.0 (9.1-11.0)
Nasopharynx	71.6 (65.4-77.6)	51.2 (46.0-57.0)	20.4 (18.2-22.8)	0.9 (0.8-0.9)	1.3 (1.2-1.4)	0.5 (0.4-0.5)	177 (156-200)	127 (108-149)	49.2 (42.6-57.0)	2.1 (1.9-2.4)	3.1 (2.7-3.7)	1.2 (1.0-1.3)
Malignant skin melanoma	62.8 (46.3-71.0)	35.4 (22.0-42.7)	27.4 (19.0-31.9)	0.8 (0.6-0.9)	1.0 (0.6-1.2)	0.6 (0.4-0.7)	290 (214-342)	153 (89.8-193)	137 (92.7-167)	3.6 (2.6-4.2)	4.0 (2.3-5.1)	3.2 (2.2-3.9)
Nonmelanoma skin	56.1 (50.4-59.8)	33.2 (30.3-35.6)	22.8 (19.3-25.2)	0.7 (0.7-0.8)	1.0 (0.9-1.1)	0.5 (0.4-0.6)	6350 (5810-6950)	3680 (3350-4060)	2670 (2430-2910)	79.1 (72.3-86.6)	102.8 (93.9-112.9)	61.1 (55.8-66.7)
Thyroid	45.6 (41.3-48.8)	18.6 (16.8-20.2)	26.9 (23.7-29.3)	0.6 (0.5-0.6)	0.5 (0.5-0.6)	0.6 (0.5-0.7)	234 (212-253)	76.0 (68.2-82.9)	158 (140-173)	2.8 (2.6-3.1)	1.9 (1.7-2.1)	3.7 (3.3-4.1)
Mesothelioma	29.3 (26.7-31.0)	21.2 (20.0-22.5)	8.03 (5.88-8.92)	0.4 (0.3-0.4)	0.6 (0.6-0.6)	0.2 (0.1-0.2)	34.5 (31.2-37.8)	25.2 (22.9-27.6)	9.34 (6.84-10.7)	0.4 (0.4-0.5)	0.7 (0.6-0.8)	0.2 (0.2-0.2)
Hodgkin lymphoma	27.6 (23.7-31.8)	17.2 (13.9-21.0)	10.4 (8.23-12.6)	0.3 (0.3-0.4)	0.4 (0.4-0.5)	0.3 (0.2-0.3)	87.5 (77.9-101.4)	51.3 (43.6-58.7)	36.2 (30.2-46.1)	1.1 (1.0-1.3)	1.3 (1.1-1.5)	0.9 (0.7-1.1)
Testicular	10.8 (9.96-11.9)	10.8 (9.96-11.9)	NA	0.1 (0.1-0.2)	0.3 (0.3-0.3)	NA	109.3 (93.4-129.5)	109.3 (93.4-129.5)	NA	1.4 (1.2-1.7)	2.8 (2.4-3.3)	NA

Abbreviations: ASIR, age-standardized incidence rate; ASMR, age-standardized mortality rate; NA, not applicable; NMSC, nonmelanoma skin cancer; UI, uncertainty interval.

^a^ Rows are ordered by decreasing number of total deaths. Cancer groups are defined based on* International Classification of Diseases, Ninth Revision* (*ICD-9*) and *International Classification of Diseases and Related Health Problems, Tenth Revision* (*ICD-10*) codes and include all codes pertaining to malignant neoplasms (*ICD-9* codes 140-208 and *ICD-10* codes C00-C96) except for Kaposi sarcoma (C46; eAppendix in the Supplement). eTables 3 and 4 in the Supplement detail how the original ICD codes were mapped to the Global Burden of Disease cancer cause list. Visual comparisons of cancer-specific incidence and mortality are provided in eFigures 14 and 15 in the Supplement. Detailed results for incidence and mortality by Sociodemographic Index quintile, region, and country can be accessed in eTables 16 and 17 in the Supplement and at https://vizhub.healthdata.org/gbd-compare/.

**Figure 1.**
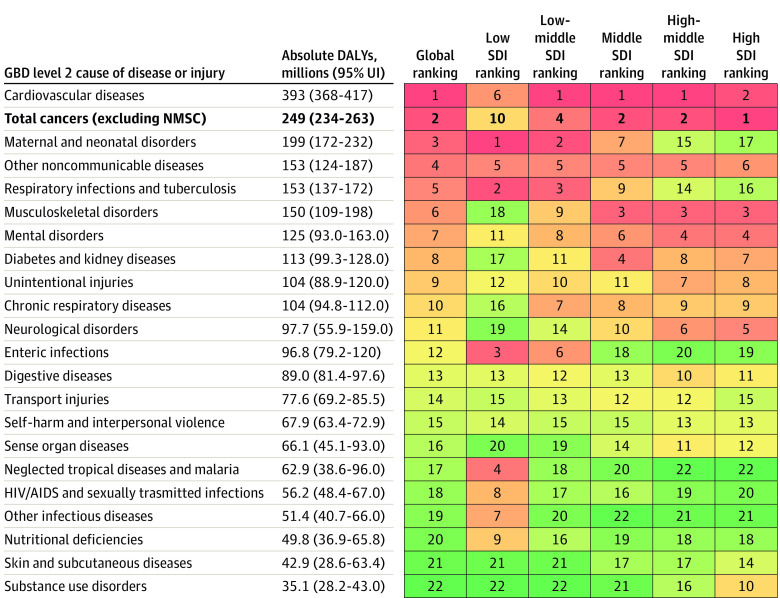
Ranking of Total Cancer Absolute Disability-Adjusted Life Years (DALYs) in 2019 Among the 22 Level 2 Categories of Disease in the Global Burden of Disease (GBD) Study by Quintile of Sociodemographic Index (SDI) Total cancers excludes nonmelanoma skin cancer. The GBD study organized diseases and injuries into a hierarchy that was mutually exclusive and collectively exhaustive. More details of this hierarchy were previously published.^22^ Colors represent the ranking of the cause within a given location group (eg, high SDI quintile) from red (highest ranking) to green (lowest ranking). The other noncommunicable diseases include congenital birth defects; urinary diseases and male infertility; gynecological diseases; hemoglobinopathies and hemolytic anemias; endocrine, metabolic, blood, and immune disorders; oral disorders; and sudden infant death syndrome. The other infectious diseases include meningitis; encephalitis; diphtheria; whooping cough; tetanus; measles; varicella and herpes zoster; acute hepatitis; and other unspecified infectious diseases. NMSC indicates nonmelanoma skin cancer; UI, uncertainty interval.

Globally, cancers were estimated to cause 250 million (95% UI, 235-264 million) DALYs in 2019 (eTable 15 in the Supplement). Of the total global DALYs, 96.9% (95% UI, 96.0%-97.7%) came from YLLs, whereas 3.1% (95% UI, 2.3%-4.0%) came from YLDs (eTable 14 and eFigure 4 in the Supplement). Among the 22 groups of diseases and injuries in level 2 of the GBD cause hierarchy (Figure 1^22^), total cancer was the second-highest cause of DALYs, deaths, and YLLs behind cardiovascular diseases (Table 2; eTable 15 in the Supplement). As such, cancer had greater overall and fatal burden globally in 2019 than other major groups of diseases in the GBD, such as maternal and neonatal disorders, musculoskeletal disorders, and respiratory infections and tuberculosis (Figure 1).

**Table 2.** Global Cancer Estimates in 2019 and Ranking Among 22 Level 2 Categories of Diseases and Injuries in the Global Burden of Disease Study Overall and by Quintile of Sociodemographic Index

Location^a^	DALYs		Deaths		YLLs		Incident cases		YLDs	
	No. millions (95% UI)	Cancer rank	No. millions (95% UI)	Cancer rank	No. millions (95% UI)	Cancer rank	No. millions (95% UI)	Cancer rank	No. millions (95% UI)	Cancer rank
Global	249.0 (233.6-263.2)	2	9.97 (9.31-10.5)	2	241.3 (226.5-255.3)	2	17.2 (15.9-18.5)	21	7.72 (5.68-9.96)	20
SDI										
Low	18.0 (15.9-20.2)	10	0.54 (0.48-0.60)	5	17.7 (15.7-19.8)	9	0.68 (0.60-0.76)	21	0.26 (0.18-0.34)	22
Low-middle	40.2 (36.8-43.7)	4	1.37 (1.26-1.49)	2	39.5 (36.1-43.0)	4	1.81 (1.67-1.96)	21	0.70 (0.52-0.92)	22
Middle	76.3 (69.7-83.2)	2	2.88 (2.62-3.15)	2	74.5 (68.0-81.4)	2	4.47 (4.05-4.89)	21	1.85 (1.36-2.44)	20
High-middle	63.5 (58.6-68.2)	2	2.65 (2.42-2.85)	2	61.4 (56.6-66.0)	2	4.69 (4.29-5.09)	21	2.11 (1.54-2.75)	16
High	50.9 (48.1-52.9)	1	2.53 (2.31-2.64)	2	48.1 (45.5-49.7)	1	5.56 (5.02-6.09)	19	2.79 (2.03-3.61)	12

Abbreviations: DALYs, disability-adjusted life years; SDI, Sociodemographic Index; UI, uncertainty interval; YLDs, years lived with disability; YLLs, years of life lost.

^a^ Total numbers and rankings exclude nonmelanoma skin cancer. All estimates refer to estimates in 2019. Rank refers to the relative ranking of the total cancer estimate for a given measure (eg, DALYs) and SDI quintile (eg, high SDI) compared among the 22 level 2 categories of diseases and injuries in the Global Burden of Disease Study 2019. More details on SDI quintiles, including population, are in the eAppendix in the Supplement. A version of this table using age-standardized rates is provided in eTable 18 in the Supplement.

**Figure 2.**
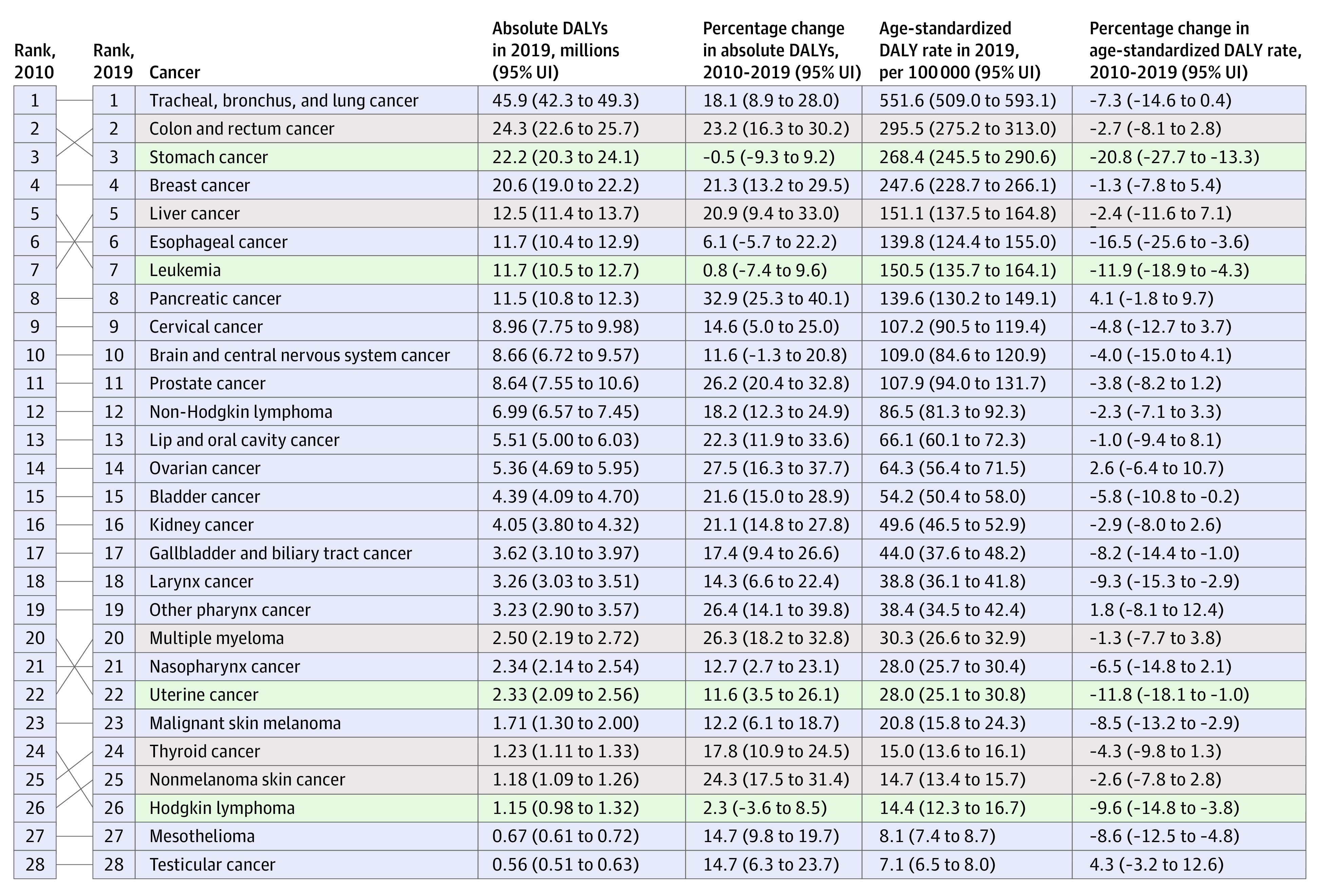
Cancer Group Rankings by Disability-Adjusted Life Years (DALYs) in 2019 and Percentage Change From 2010 to 2019 Rankings are by absolute DALYs and exclude the other malignant neoplasms cancer group. Cancers are ordered by rank in 2019, with lines connecting to their rank in 2010. Absolute DALYs and age-standardized DALY rates for 2010 can be found online at https://vizhub.healthdata.org/gbd-compare/. Colors refer to the directional change in cancer rank from 2010 to 2019: red signifies an increase in rank, blue signifies no change in rank, and green signifies a decrease in rank. UI indicates uncertainty interval.

The 5 leading causes of cancer-related DALYs for both sexes combined (Figure 2), excluding other malignant neoplasms, were tracheal, bronchus, and lung (TBL) cancer, with 18.3% (95% UI, 17.5%-19.1%) of total cancer-related DALYs; colon and rectum cancer (CRC), with 9.7% (95% UI, 9.4%-10.0%); stomach cancer, with 8.9% (8.6%-9.3%); breast cancer, with 8.2% (7.8%-8.7%); and liver cancer, with 5.0% (4.8%-5.3%).

Tracheal, bronchus, and lung cancer were estimated to cause 45.9 million (95% UI, 42.3-49.3 million) DALYs in 2019; of these, 98.8% (95% UI, 98.5%-99.1%) came from YLLs and just 1.2% (95% UI, 0.9%-1.5%) from YLDs (eTable 14 and eFigure 4 in the Supplement). In 2019, there were 2.04 million (95% UI, 1.88-2.19 million) deaths due to TBL cancer and 2.26 million (95% UI, 2.07-2.45 million) incident TBL cases (Table 1). Tracheal, bronchus, and lung cancer was the leading cause of cancer incidence and mortality in 58 and 119 countries and territories, respectively, for males (eFigures 5 and 6 in the Supplement), and 1 and 27 countries, respectively, for females (eFigures 7 and 8 in the Supplement).

Colon and rectum cancer were estimated to cause 24.3 million (95% UI, 22.6-25.7 million) DALYs in 2019; of these, 95.6% (95% UI, 94.4%-96.8%) came from YLLs and 4.4% (95% UI, 3.2%-5.6%) from YLDs (eTable 14 and eFigure 4 in the Supplement). In 2019, there were 1.09 million (95% UI, 1.00-1.15 million) deaths due to CRC and 2.17 million (95% UI, 2.00-2.34 million) incident CRC cases (Table 1). Colon and rectum cancer was the leading cause of cancer incidence and mortality in 1 country and 9 countries, respectively, for females (eFigures 7 and 8 in the Supplement) and of cancer incidence for 11 countries in males (eFigure 5 in the Supplement).

Stomach cancer was estimated to cause an estimated 22.2 million (95% UI, 20.3-24.1 million) DALYs in 2019; of these, 98.4% (95% UI, 98.0%-98.9%) came from YLLs and 1.6% (95% UI, 1.1%-2.0%) from YLDs (eTable 14 and eFigure 4 in the Supplement). There were also 957 000 (95% UI, 871 000-1 030 000) deaths and 1.27 million (95% UI, 1.15-1.40 million) incident cases of stomach cancer in 2019 (Table 1). Stomach cancer was the leading cause of cancer incidence and mortality in 5 and 11 countries, respectively, for males (eFigures 5 and 6 in the Supplement) and of cancer mortality in 6 countries for females (eFigure 8 in the Supplement).

Breast cancer was the leading cause of cancer-related DALYs, deaths, and YLLs among females globally in 2019. Most of the global breast cancer burden occurred for females, with 20.3 million (95% UI, 18.7-21.9 million) of 20.6 million (95% UI, 19.0-22.2 million) total breast cancer–related DALYs in 2019 occurring in females, of which 93.3% (95% UI, 91.1%-95.2%) came from YLLs and 6.7% (95% UI, 4.8%-8.9%) from YLDs (eTable 14 and eFigure 4 in the Supplement). Likewise, 689 000 (95% UI, 635 000-740 000) of 701 000 (95% UI, 647 000-752 000) breast cancer deaths occurred in females, and 1.98 million (95% UI, 1.81–2.15 million) of 2.00 million (95% UI, 1.83–2.17 million) incident cases of breast cancer (Table 1). For females, breast cancer was the leading cause of cancer incidence in 157 countries and deaths in 119 countries (eFigures 7 and 8 in the Supplement).

Liver cancer was estimated to cause 12.5 million (95% UI, 11.4-13.7 million) DALYs in 2019; of these, 99.0% (95% UI, 98.6%-99.3%) came from YLLs and 1.0% (95% UI, 0.7%-1.4%) from YLDs (eTable 14 and eFigure 4 in the Supplement). There were also 485 000 (95% UI, 444 000-526 000) deaths and 534 000 (95% UI, 487 000-589 000) incident cases of liver cancer in 2019 (Table 1). Liver cancer was the leading cause of cancer incidence and mortality in 6 and 8 countries, respectively, in males (eFigures 5 and 6 in the Supplement) and 1 and 2 countries, respectively, in females (eFigures 7 and 8 in the Supplement).

Sex-specific DALY rankings differed slightly from those previously described because of the higher prominence of several sex-specific cancers. Among males, TBL cancer remained the leading cause of cancer-related DALYs globally, followed by stomach, CRC, liver, and esophageal cancer, with prostate cancer sixth (eFigure 9 in the Supplement). Among females, the leading cause of cancer-related DALYs globally was breast cancer, followed by TBL, CRC, cervical, and stomach cancer, with ovarian cancer sixth (eFigure 10 in the Supplement).

### Global Trends in Cancer Burden From 2010 to 2019

Globally, the number of new cancer cases increased from 18.7 million (95% UI, 18.0-19.3 million) in 2010 to 23.6 million (95% UI, 22.2-24.9 million) in 2019, an increase of 26.3% (95% UI, 20.3%-32.3%). Age-standardized incidence rates remained generally the same during this period, with a difference of −1.1% (95% UI, −5.8% to 3.5%) and an annualized rate of change of −0.1% (95% UI, −0.7% to 0.4%). Excluding NMSC, the number of incident cases increased from 13.8 million (95% UI, 13.3-14.3 million) in 2010 to 17.2 million (95% UI, 15.9-18.5 million) in 2019, a 24.6% (95% UI, 16.8%-32.6%) increase, while the age-standardized incidence rates remained the same during this period, with a difference of −1.6% (95% UI, −7.7% to 4.6%) and an annualized rate of change of −0.2% (95% UI, −0.9% to 0.5%).

Similarly, the number of global total cancer deaths increased by 20.9% (95% UI, 14.2%-27.6%) from 8.29 million (95% UI, 7.89-8.57 million) in 2010 to 10.0 million (95% UI, 9.36-10.6 million) in 2019. Cancer deaths also increased as a proportion of total deaths of all causes, rising from 15.7% (95% UI, 15.0%-16.2%) in 2010 to 17.7% (95% UI, 16.8%-18.4%) in 2019. By contrast, age-standardized mortality rates declined by −5.9% (95% UI, −11.0% to −0.9%) during this 10-year period, with an annualized rate of change of −0.7% (95% UI, −1.3% to −0.1%). During this decade, the absolute number of global cancer-related DALYs increased by 16.0% (95% UI, 9.3%-22.8%) from 216 million (95% UI, 208-223 million) in 2010 to 250 million (95% UI, 235-264 million) in 2019. The proportion of estimated total global DALYs that were due to cancer increased from 8.4% (95% UI, 7.7%-9.0%) of total DALYs from all causes in 2010 to 9.9% (95% UI, 8.9%-10.9%) in 2019. A decline is also evident in the age-standardized rates, as age-standardized cancer-related DALYs rates decreased by −6.6% (95% UI, −11.9% to −1.1%) during this period.

**Figure 3.**
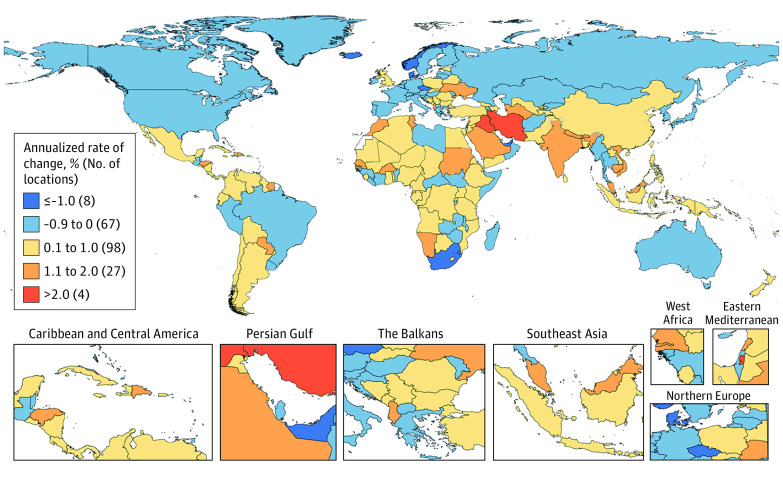
Annualized Rate of Change in Age-Standardized Total Cancer Incidence Rate From 2010 to 2019 in Both Sexes Total cancer excludes nonmelanoma skin cancers. Annualized rate of change from 2010 to 2019 represents the average percentage change per year during this period, with negative values indicating decreasing incidence rates and positive values indicating increasing incidence rates. There were several geographic locations where estimates were not available (eg, Western Sahara and French Guiana), as they were not modeled locations in the Global Burden of Disease 2019 Study.

**Figure 4.**
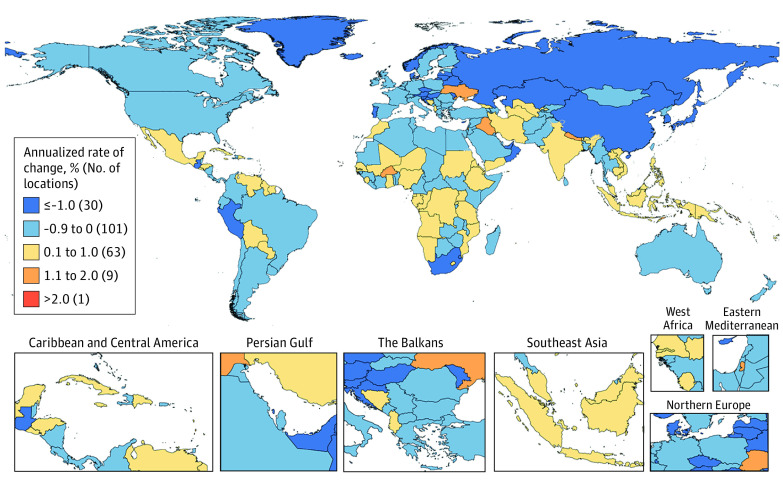
Annualized Rate of Change in Age-Standardized Total Cancer Mortality Rate From 2010 to 2019 in Both Sexes Total cancer excludes nonmelanoma skin cancers. Annualized rate of change from 2010 to 2019 represents the mean percentage change per year during this period, with negative values indicating decreasing mortality rates and positive values indicating increasing mortality rates. There were several geographic locations where estimates were not available (eg, Western Sahara and French Guiana) as they were not modeled locations in the Global Burden of Disease 2019 Study.

Location-specific annualized rates of change in age-standardized mortality and incidence rates from 2010 to 2019 for total cancers, excluding NMSC, varied by location. During this period, age-standardized mortality rates decreased in 131 of 204 countries and territories (64.2%; Figure 3), and age-standardized incidence rates decreased in 75 of 204 countries and territories (36.8%; Figure 4).

Trends during the last decade varied by type of cancer, including several shifts in cancer group rankings by absolute DALYs (Figure 2). For example, CRC and liver cancer rose from the third and seventh leading causes of cancer-related DALYs in 2010 to second and fifth in 2019 because of large increases in the number of DALYs and small decreases in age-standardized DALY rates. In contrast, stomach cancer and leukemia dropped from second and fifth to third and seventh during the same period because of large decreases in age-standardized DALY rates and minimal changes in the number of DALYs (Figure 2).

### Cancer Burden by SDI

**Figure 5.**
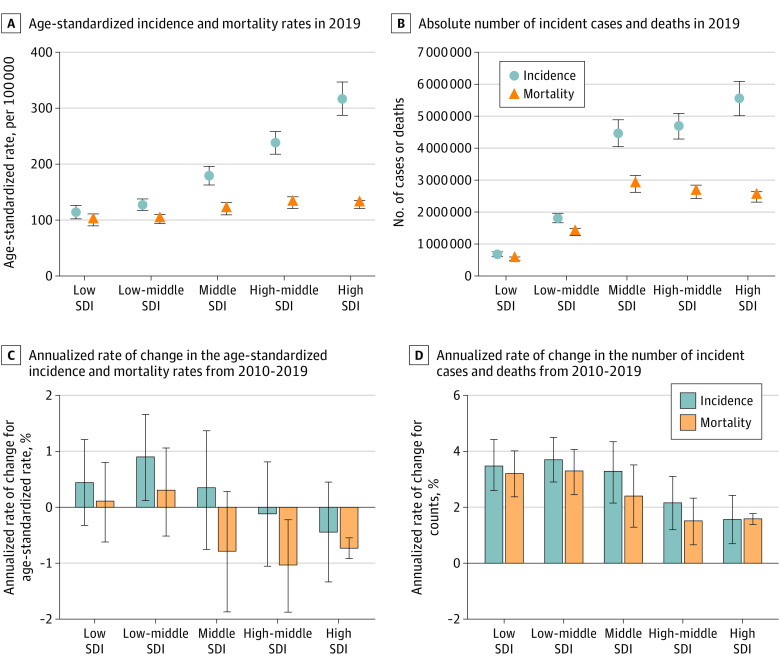
Total Cancer Incidence and Mortality Age-Standardized Rates and Absolute Counts in 2019 and Annualized Rate of Change for Incidence and Mortality in Age-Standardized Rates and Absolute Counts From 2010 to 2019 by Sociodemographic Index (SDI) Quintile Panels provide global estimates for total cancers, except nonmelanoma skin cancer, stratified by SDI quintile. Annualized rate of change from 2010 to 2019 represents the mean percentage change per year during this period. Black bars represent 95% uncertainty intervals.

Cancer burden varied considerably across SDI quintiles in 2019 levels and rankings (Table 2 and Figure 4) and trends during the 2010 to 2019 study period (Figure 5; eTables 16 and 17 in the Supplement). The following results exclude NMSC.

In the high SDI quintile in 2019, there were 50.9 million (95% UI, 48.1-52.9 million) DALYs estimated to be caused by cancer, of which 94.5% (95% UI, 93.1%-95.9%) were from YLLs and 5.5% (95% UI, 4.1%-6.9%) from YLDs. The most cases and the highest age-standardized incidence rates were in the high SDI quintile (Table 2; Figure 5). Compared with GBD level 2 groups of diseases and injuries, cancer was the leading cause of YLLs and DALYs in the high SDI quintile and was the leading or second leading cause of deaths by age-standardized rate or absolute number, respectively. In the high-middle SDI quintile, there were 63.5 million (95% UI, 58.6-68.2 million) DALYs estimated to be caused by cancer, of which 96.7% (95% UI, 95.7%-97.6%) were from YLLs and 3.3% (95% UI, 2.4%-4.3%) from YLDs. The high-middle SDI had the highest age-standardized rates of deaths and DALYs of all SDI quintiles and the second highest age-standardized incidence rate (Table 2; Figure 5).

The middle SDI quintile had the highest number of cancer-related DALYs and deaths of any SDI quintile in 2019, with 76.3 million (95% UI, 69.7-83.2 million) DALYs and 2.88 million (95% UI, 2.62-3.15 million) deaths (Table 2, Figure 5). Of the SDI quintiles, the middle SDI quintile had the largest total population (eAppendix in the Supplement). For DALYs, 97.6% (95% UI, 96.8%-98.3%) came from YLLs and 2.4% (95% UI, 1.7%-3.2%) from YLDs. In the low-middle SDI quintile, there were 40.2 million (95% UI, 36.8-43.7 million) DALYs estimated to be caused by cancer in 2019; of these, 98.2% (95% UI, 97.7%-98.7%) were from YLLs and 1.8% (95% UI, 1.3%-2.3%) from YLDs.

In the low SDI quintile, there were 18.0 million (95% UI, 15.9-20.2 million) DALYs estimated to be caused by cancer in 2019; of these, 98.6% (95% UI, 98.1%-98.9%) were from YLLs and 1.4% (95% UI, 1.1%-1.9%) from YLDs. The low SDI quintile had the lowest numbers and age-standardized rates of cancer cases and deaths (Table 2; Figure 5). In contrast to the higher rankings in other quintiles, cancer was the fifth leading cause of death in the low SDI quintile in 2019, ninth for YLLs, and tenth for DALYs.

Alongside these differences, some patterns held across most SDI quintiles. In 2019, TBL cancer had the highest number of cancer deaths and DALYs in both sexes combined in all but the low SDI quintile, in which it was breast cancer (eFigure 11 in the Supplement). Excluding NMSC, the most incident cases occurred for CRC in the high SDI quintile, TBL in the high-middle and middle SDI quintiles, and breast cancer in the low-middle and low SDI quintiles (eFigure 12 in the Supplement).

While in 2019 the largest absolute numbers of cases and deaths occurred in the middle to high SDI quintiles, from 2010 to 2019, the largest increasing annualized rates of change in the absolute numbers of cases and deaths occurred in the low-middle SDI quintile and then the low SDI quintile (Figure 5; eTables 16 and 17 in the Supplement). Changes in age-standardized rates from 2010 to 2019 also varied by SDI quintile. For mortality, age-standardized rates increased from 2010 to 2019 in the low and low-middle SDI quintiles but decreased in the middle to high SDI quintiles. For incidence, age-standardized rates increased during this period for the low to middle SDI quintiles but decreased in the high-middle and high SDI quintiles, with the largest decrease in the high SDI quintile. While there was substantial heterogeneity between countries and territories within the same SDI quintile, country-specific estimates showed similar overall trends between SDI and age-standardized incidence and mortality rates (eFigure 13 in the Supplement).

## Discussion

The results of this systematic analysis demonstrate the substantial and growing global burden of cancer, with patterns of burden differing by SDI quintile. In 2019, cancer-related DALYs were second only to cardiovascular diseases in their contribution to global disease burden, and in the high SDI quintile, cancer overtook cardiovascular disease to become the leading cause of DALYs. Between 2010 and 2019, the number of new global cancer cases and deaths increased by 26.3% and 20.9%, respectively. However, the largest percentage increases in cancer incidence and mortality during the last decade occurred in the lower SDI quintiles, likely reflecting ongoing epidemiologic transitions, demographic shifts, and disparities in cancer prevention, care, and control. Together, these results provide comprehensive and comparable estimates that can potentially inform efforts for equitably reducing the evolving burden of cancer globally.

While the absolute burden of cancer grew from 2010 to 2019, global age-standardized incidence rates remained similar at −1.1% (95% UI, −5.8% to 3.5%) and mortality rates decreased by −5.9% (95% UI, −11.0% to −0.9%). These age-standardized mortality results suggest cautious optimism that some progress may have been made in early diagnosis and cancer treatment globally during the last decade. However, inequities in the distribution and growth of cancer burden around the world diminish this potential advancement and suggest that an acceleration of efforts to effectively address cancer burden are needed. Of particular concern, recent progress in reducing age-standardized incidence and mortality rates seems concentrated in higher SDI locations, while both rates are still trending upward in lower SDI locations. The increasing age-standardized incidence and mortality rates in lower SDI quintiles may reflect several factors, including shifting population age structures, increasing capacity for diagnosis and registration of cancer cases and deaths, and changes in cancer risk factors, such as metabolic, behavioral, environmental, and occupational exposures. For example, changing patterns of smoking prevalence by SDI quintile may be particularly relevant to cancer burden,^35^ with a need for further smoking reduction and tobacco control initiatives in many countries.^36,37^ These differences in cancer burden across the SDI spectrum suggest a need to tailor cancer control efforts to specific resource contexts and health system needs, incorporating local cultural and cancer context-specific knowledge.

Low and low-middle SDI locations had a higher rate of growth in the number of cases and deaths than high SDI locations during the last decade. Consistent with this trend, forecasts of cancer incidence^38^ and mortality^1^ suggest a growing burden in these locations, predicting that by 2040 more than two-thirds of the world’s cancers will occur in low-income and middle-income countries.^38^ Increasing cancer burden in already overburdened and underresourced settings is concerning given existing disparities in health care access and coverage.^2,3,39^ As many in countries within the lower SDI quintiles have insufficient access to cancer prevention services, timely diagnosis, and comprehensive treatment, efforts to strengthen cancer control infrastructure, expand workforce capacity, and increase access to universal health coverage and sufficient financial security will be crucial.^3,40^ The grouping of countries by SDI quintile is not meant to imply that all countries within an SDI quintile have equivalent capacity to prevent, diagnose, or treat cancers; each country has unique strengths and needs that should be considered. Further, the growing absolute number of cases and deaths in all SDI quintiles suggests that even as progress has been made in reducing age-standardized rates in some settings, globally there is an expanding need for health care infrastructure that is capable of supporting the provision of effective diagnoses and treatments for a growing number of patients with cancer.

While the traditional cancer metrics of incidence and mortality are crucial, DALY estimates provide perspective on the healthy years of life lost because of cancer morbidity and mortality globally. The GBD 2019 study found that on a global level, most cancer-related DALYs (96.9%; 95% UI, 96.0%-97.7%) in 2019 came from YLLs, suggesting that the total health loss from cancer was primarily associated with premature death. This finding is a valuable reminder of the lives that prematurely ended because of cancer globally and the importance of working toward improved global survival outcomes. While YLDs contribute less to global DALYs, the percentage of DALYs estimated to be caused by YLDs increased with increasing SDI quintiles, ranging from 1.4% (95% UI, 1.1%-1.8%) in the low SDI quintile to 5.7% (95% UI, 4.2%-7.1%) in the high SDI quintile. This greater comparative contribution of YLDs in higher SDI settings is consistent with likely improved survival,^41^ given generally more available access to cancer screening,^42,43^ diagnosis,^44,45^ and treatment^46,47^ as SDI increases. Consequently, the contribution of YLDs to health loss due to cancer would be expected to be increasingly relevant to global health planning as cancer survival improves globally, and the support needs of survivors of cancer should be considered as part of comprehensive cancer control planning efforts.^48,49^

The contribution of cancers to total global DALYs estimated to be caused by disease and injury has increased during the past decade, rising from third place in 2010 to second place in 2019, remaining behind only cardiovascular diseases. However, in high SDI settings, cancer-related DALYs have surpassed cardiovascular disease–related DALYs to become the leading cause of total disease burden in 2019. Other studies have described cancer’s emerging prominence as the leading cause of premature death in countries with high income^50^ or a high Human Development Index,^4^ some of which is attributed to relative decreases in cardiovascular disease deaths.^4,50,51^ The GBD 2019 study builds on this evolving global landscape of cancer burden by demonstrating the comparative importance of cancer in high SDI settings not just for mortality, but also when comparing the nonfatal burden of cancer and other diseases.

Together, these results suggest the need for increased cancer prevention and control efforts to reduce current burden,^52^ as well as the need to accelerate progress in lower SDI locations to reduce the effect of growing burden.^1,2^ One important step is bolstering national cancer control plans (NCCPs)^53,54,55,56^ that identify, plan, and evaluate a framework of cost-effective and feasible interventions, such as the World Health Organization’s best buys proposals for cancer prevention, diagnosis, and management.^38^ The increasing global uptake of NCCPs has demonstrated the utility of this approach in addressing cancer burden in several settings.^57,58,59^ However, creating and implementing effective NCCPs requires detailed knowledge about the local burden of cancer and associated risk factors, in addition to awareness of sociocultural circumstances and previous cancer control implementation efforts. Lack of information about local cancer epidemiology can be a substantial barrier in some data-sparse, and often resource-limited, locations.^60,61^ Cancer burden estimates, such as those in the GBD 2019 study, can potentially be helpful as part of context-specific cancer resource planning and prioritization efforts.

### Limitations

Several limitations provide opportunities for improvement in future GBD iterations. An ongoing challenge is a lack of high-quality data in many locations. This includes time lags in data availability, nonspecific cause of death data from vital registration systems, and ascertainment limitations of verbal autopsy reports. The GBD addresses these data limitations through data-seeking efforts, data processing corrections, and modeling approaches that incorporate geospatial and temporal smoothing. These approaches allow the estimation of comprehensive results with appropriate uncertainty bounds. However, in years or locations where data were not available, estimates relied on covariates and modeling parameters, which may overestimate or underestimate true cancer burden. As data can be less available or reliable in locations within the lower SDI quintiles,^19^ estimates should be interpreted with some caution. These data limitations reinforce the need for enhancing cancer surveillance globally.^61,62^

Similarly, scarcity of age-specific and year-specific survival data requires using MIRs to estimate survival, which may not approximate location-specific survival trends well. Years lived with disability are currently estimated based on 10-year prevalence, which may underestimate the lifelong health loss and disability that some survivors of cancer experience, particularly for survivors of pediatric cancer.^63^ While the lifelong disability from treatment-related surgical procedures is currently estimated for 5 cancers, other sources of long-term disability in survivors of cancer have not yet been captured in these analyses. Finally, this study only estimated global cancer burden through 2019, and as such did not incorporate any associations of the COVID-19 pandemic with global cancer morbidity and mortality. Assessing these associations will be critical for future work on cancer burden, as the ongoing pandemic is likely to delay progress in efforts to reduce health loss from cancer globally through delays and reductions in screening, diagnosis, and treatment.^8,9,10,11,12^

## Conclusions

This systematic analysis of the GBD 2019 study provides comprehensive and comparable estimates of cancer burden worldwide, which were updated and improved from previous GBD cycles. These estimates varied substantially by SDI quintile, highlighting global inequities in cancer burden. While the high SDI quintile had the highest estimated number of incident cases in 2019, the middle SDI quintile had the highest estimated number of deaths and DALYs. During the last decade, cancer burden has grown the fastest in the low and low-middle SDI quintiles. Such estimates are vital for improving equity in global cancer outcomes and meeting key SDG targets for reducing cancer and other noncommunicable disease burden.

## Notes

Supplement. eAppendix. eTable 1. Number of site-years for cancer mortality data by source type, for GBD 2019 compared to GBD 2017 eTable 2. Socio-demographic Index groupings by geography, based on 2019 values eTable 3. List of International Classification of Diseases (ICD) codes mapped to the Global Burden of Disease cause list for cancer incidence data eTable 4. List of International Classification of Diseases (ICD) codes mapped to the Global Burden of Disease cause list for cancer mortality data eTable 5. Undefined cancer code categories (ICD-10) and respective target codes for cancer registry incidence data eTable 6. Cancer registry sources for cancer incidence and mortality-to-incidence ratio data by country, year, and registry eTable 7. Covariates selected for CODEm for each GBD cancer group and expected direction of covariate eTable 8. Comparison of GBD 2017 and GBD 2019 covariates used and level of covariates eTable 9. Results for CODEm model performance testing eTable 10. Percent change before and after CoDCorrect by cancer for all ages, both sexes combined, 2019 eTable 11. Duration of four prevalence phases by cancer eTable 12. Disability weights eTable 13. GATHER5 guidelines checklist eTable 14. Contribution of YLDs and YLLs to DALYs by cancer, global, both sexes, 2019 eTable 15. Global number of incidence, prevalence, YLDs, deaths, YLLs, DALYs for both sexes, 2010 and 2019 for all level 2 GBD causes eTable 16. Trends in incidence globally, and by SDI quintile, both sexes, 2010 to 2019 eTable 17. Trends in mortality globally, and by SDI quintile, both sexes, 2010 to 2019 eTable 18. Global age-standardized cancer estimates in 2019 and ranking among 22 level 2 categories of diseases and injuries in the Global Burden of Disease Study, overall and by quintile of socio-demographic index eFigure 1. Socio-demographic index quintiles for the Global Burden of Disease Study 2019 eFigure 2. Flowchart GBD cancer mortality, YLL estimation eFigure 3. Flowchart GBD cancer incidence, prevalence, YLD estimation eFigure 4. Contribution of YLDs and YLLs to DALYs by cancer, global, both sexes, 2019 eFigure 5. Top-ranked cancers by absolute number of incident cases for all ages in males, 2019 eFigure 6. Top-ranked cancers by absolute number of deaths for all ages in males, 2019 eFigure 7. Top-ranked cancers by absolute number of incident cases for all ages in females, 2019 eFigure 8. Top-ranked cancers by absolute number of deaths for all ages in females, 2019 eFigure 9. Cancers ranked by disability-adjusted life years (DALYs) for males between 2010 and 2019 eFigure 10. Cancers ranked by disability-adjusted life years (DALYs) for females between 2010 and 2019 eFigure 11. Ranking of 29 cancer groups by mortality in 2019, by country and location group eFigure 12. Ranking of 29 cancer groups by incidence in 2019, by country and location group eFigure 13. Age-standardized rates and annualized rate of change for age-standardized rates for total cancers excluding non-melanoma skin cancer, all ages, both sexes eFigure 14. Global counts of cancer-specific incidence and deaths in 2019, overall and by sex eFigure 15. Global age-standardized cancer-specific incidence and mortality rates in 2019, overall and by sex
