# Supplementary material for: Cancer Incidence, Mortality, Years of Life Lost, Years Lived With Disability, and Disability-Adjusted Life Years for 29 Cancer Groups From 2010 to 2019: A Systematic Analysis for the Global Burden of Disease Study 2019
Source: JAMA Oncol. 2021 Dec 30;8(3):420–44. doi: 10.1001/jamaoncol.2021.6987 (PMC8719276; doi:10.1001/jamaoncol.2021.6987)
Supplement: Supplement. — eAppendix. eTable 1. Number of site-years for cancer mortality data by source type, for GBD 2019 compared to GBD 2017 eTable 2. Socio-demographic Index groupings by geography, based on 2019 values eTable 3. List of International Classification of Diseases (ICD) codes mapped to the Global Burden of Disease cause list for cancer incidence data eTable 4. List of International Classification of Diseases (ICD) codes mapped to the Global Burden of Disease cause list for cancer mortality data eTable 5. Undefined cancer code categories (ICD-10) and respective target codes for cancer registry incidence data eTable 6. Cancer registry sources for cancer incidence and mortality-to-incidence ratio data by country, year, and registry eTable 7. Covariates selected for CODEm for each GBD cancer group and expected direction of covariate eTable 8. Comparison of GBD 2017 and GBD 2019 covariates used and level of covariates eTable 9. Results for CODEm model performance testing eTable 10. Percent change before and after CoDCorrect by cancer for all ages, both sexes combined, 2019 eTable 11. Duration of four prevalence phases by cancer eTable 12. Disability weights eTable 13. GATHER5 guidelines checklist eTable 14. Contribution of YLDs and YLLs to DALYs by cancer, global, both sexes, 2019 eTable 15. Global number of incidence, prevalence, YLDs, deaths, YLLs, DALYs for both sexes, 2010 and 2019 for all level 2 GBD causes eTable 16. Trends in incidence globally, and by SDI quintile, both sexes, 2010 to 2019 eTable 17. Trends in mortality globally, and by SDI quintile, both sexes, 2010 to 2019 eTable 18. Global age-standardized cancer estimates in 2019 and ranking among 22 level 2 categories of diseases and injuries in the Global Burden of Disease Study, overall and by quintile of socio-demographic index eFigure 1. Socio-demographic index quintiles for the Global Burden of Disease Study 2019 eFigure 2. Flowchart GBD cancer mortality, YLL estimation eFigure 3. Flowchart GBD cancer incidence, [file jamaoncol-e216987-s001.pdf]

Supplemental Online Content

Kocarnik JM, Compton K, Dean FE, et al; Global Burden of Disease 2019 Cancer Collaboration. Cancer incidence, mortality, years of life lost, years lived with disability, and disability-adjusted life years for 29 cancer groups from 2010 to 2019: a systematic analysis for the Global Burden of Disease Study 2019. *JAMA Oncol*. Published online December 30, 2021. doi:10.1001/jamaoncol.2021.6987

eAppendix.
eTable 1. Number of site-years for cancer mortality data by source type, for GBD 2019 compared to GBD 2017
eTable 2. Socio-demographic Index groupings by geography, based on 2019 values
eTable 3. List of International Classification of Diseases (ICD) codes mapped to the Global Burden of Disease cause list for cancer incidence data
eTable 4. List of International Classification of Diseases (ICD) codes mapped to the Global Burden of Disease cause list for cancer mortality data
eTable 5. Undefined cancer code categories (ICD-10) and respective target codes for cancer registry incidence data
eTable 6. Cancer registry sources for cancer incidence and mortality-to-incidence ratio data by country, year, and registry
eTable 7. Covariates selected for CODEm for each GBD cancer group and expected direction of covariate
eTable 8. Comparison of GBD 2017 and GBD 2019 covariates used and level of covariates
eTable 9. Results for CODEm model performance testing
eTable 10. Percent change before and after CoDCorrect by cancer for all ages, both sexes combined, 2019
eTable 11. Duration of four prevalence phases by cancer
eTable 12. Disability weights
eTable 13. GATHER5 guidelines checklist
eTable 14. Contribution of YLDs and YLLs to DALYs by cancer, global, both sexes, 2019
eTable 15. Global number of incidence, prevalence, YLDs, deaths, YLLs, DALYs for both sexes, 2010 and 2019 for all level 2 GBD causes
eTable 16. Trends in incidence globally, and by SDI quintile, both sexes, 2010 to 2019
eTable 17. Trends in mortality globally, and by SDI quintile, both sexes, 2010 to 2019
eTable 18. Global age-standardized cancer estimates in 2019 and ranking among 22 level 2 categories of diseases and injuries in the Global Burden of Disease Study, overall and by quintile of socio-demographic index
eFigure 1. Socio-demographic index quintiles for the Global Burden of Disease Study 2019
eFigure 2. Flowchart GBD cancer mortality, YLL estimation
eFigure 3. Flowchart GBD cancer incidence, prevalence, YLD estimation
eFigure 4. Contribution of YLDs and YLLs to DALYs by cancer, global, both sexes, 2019
eFigure 5. Top-ranked cancers by absolute number of incident cases for all ages in males, 2019
eFigure 6. Top-ranked cancers by absolute number of deaths for all ages in males, 2019
eFigure 7. Top-ranked cancers by absolute number of incident cases for all ages in females, 2019
eFigure 8. Top-ranked cancers by absolute number of deaths for all ages in females, 2019
eFigure 9. Cancers ranked by disability-adjusted life years (DALYs) for males between 2010 and 2019
eFigure 10. Cancers ranked by disability-adjusted life years (DALYs) for females between 2010 and 2019
eFigure 11. Ranking of 29 cancer groups by mortality in 2019, by country and location group
eFigure 12. Ranking of 29 cancer groups by incidence in 2019, by country and location group
eFigure 13. Age-standardized rates and annualized rate of change for age-standardized rates for total cancers excluding non-melanoma skin cancer, all ages, both sexes
eFigure 14. Global counts of cancer-specific incidence and deaths in 2019, overall and by sex
eFigure 15. Global age-standardized cancer-specific incidence and mortality rates in 2019, overall and by sex

This supplemental material has been provided by the authors to give readers additional information about their work.

© 2021 Kocarnik JM et al; Global Burden of Disease 2019 Cancer Collaboration. *JAMA Oncology*.

eAppendix.

Updates to this appendix were only made if methods were updated for GBD 2019; other parts remain the same as in the appendix to the GBD 2017 manuscript "Global Burden of Disease Cancer Collaboration. Global, Regional, and National Cancer Incidence, Mortality, Years of Life Lost, Years Lived With Disability, and Disability-Adjusted Life-Years for 29 Cancer Groups, 1990 to 2017: A Systematic Analysis for the Global Burden of Disease Study. *JAMA Oncol.* 2019;5(12):1749–1768. doi:10.1001/jamaoncol.2019.2996."[1] Certain parts of this appendix were published in appendices of other GBD 2019 publications and are cited when used.[2–4]

Additional method summaries for all GBD malignant neoplasms except for NMSC

Cancers in the GBD Cause Hierarchy

The Global Burden of Disease (GBD) cause list is organized in a hierarchy. Levels 1 and 2 represent general groupings of causes, while levels 3 and 4 represent increasingly specific causes. The general Level 1 group "Non-communicable diseases" includes the broad Level 2 group "Neoplasms," which includes all malignant and benign neoplasms. Level 3 includes 30 cancer groups, and Level 4 includes 42 groups since the Level 3 parent causes "Leukemia", "Liver cancer", "Non-melanoma skin cancer", and "Other neoplasms" are further subdivided into Level 4 subtypes. In this publication, estimates for the GBD cancer groups are presented as totals combined across age groups, sexes, and locations for the time period from 2010 to 2019. More specific results are available via the GBD 2019 Results Tool (http://ghdx.healthdata.org/gbd-results-tool), which provides estimates for each of the 5-year GBD age groups (0-5; 5-9; etc. until 95+, plus more granular reporting in ages under 5 years), for each year 1990 to 2019, by sex, and in 204 countries or territories. All *International Classification of Disease*[6] (ICD) codes pertaining to cancer from ICD-9 (codes 140-209) and ICD-10 (codes C00-C96) are included in the estimates for "malignant neoplasms," except for Kaposi sarcoma (ICD-9: 176, ICD-10: C46). Kaposi sarcoma deaths are primarily redistributed to HIV/AIDS according to age (100% for ages 0-15 years, 95% for 15-50 years, and 90% for ≥50 years), with the remaining deaths assigned to "other malignant neoplasms".[2] All ICD-9 and ICD-10 codes pertaining to neoplasms (ICD-9 140-239, ICD-10 C00-D49) are included in the estimates for "Neoplasms." eTable 4 and eTable list all ICD codes and their respective GBD causes.

Benign neoplasms are accounted for in the Level 3 GBD cause "Other neoplasms," (Level 3; ICD-9 codes 210-239, ICD-10 codes D00-D49), which is further subdivided into the Level 4 causes "Myelodysplastic, myeloproliferative, and other hematopoietic neoplasms," "Benign and in situ cervical and uterine neoplasms," "Benign and in situ intestinal neoplasms," and "Other benign and in situ neoplasms."

An important change from the GBD 2017 cancer cause hierarchy is that in GBD 2017, the Level 3 cause "Other neoplasms" and its underlying Level 4 causes were not counted in the total incidence for the Level 2 "Neoplasms" cause; this is no longer the case in the GBD 2019 analysis. In this current iteration of GBD results reporting (GBD 2019), incidence, prevalence, mortality, years lived with disability (YLDs), years of life lost (YLLs), and disability-adjusted life years (DALYs) estimates for total neoplasms are true aggregates of all underlying causes, including Level 3 "Other neoplasms." For convenience to those primarily interested in estimates for malignant neoplasms, the GBD 2019 Results Tool (http://ghdx.healthdata.org/gbd-results-tool) now also includes a new custom Level 2 cancer grouping, "D. Total Cancers." This "Total Cancers" grouping provides estimates for the subset of the total neoplasms that exclude benign and in situ neoplasms. Of note, this manuscript focuses on reporting results for "Total Cancers", not "Neoplasms", but estimates for "Neoplasms" are available via the GBD 2019 Results Tool as well.

Data sources

The GBD cause of death (CoD) database contains cancer mortality data originating from multiple sources, including vital registration (VR), verbal autopsy (VA), and cancer registry (CR) data. The cancer registry mortality estimates that are uploaded into the CoD database stem from cancer registry

incidence data that have been transformed to mortality estimates through the use of mortality-to-incidence ratios (MIRs).

A summary of cancer-, location-, and year-specific data sources used for GBD 2019 can be found in eTable 3. These included 767,514 sources from vital registration systems (111,084 new for GBD 2019), 155,542 sources from cancer registries (25,901 new for GBD 2019), and 6,137 sources from verbal autopsy reports.

Cancer registry data sources

Cancer incidence and mortality data were sought from individual cancer registries, such as the Surveillance, Epidemiology, and End Results (SEER) Program[7]; provided by collaborators; or downloaded from aggregated databases of cancer registry data such as "Cancer Incidence In Five Continents" (CI5),"[8–18] EUREG,[19] or NORDCAN.[20] Only population-based cancer registries were included, with inclusion criteria that they included all cancers (i.e. were not specialty registries), reported data for all age groups (except for pediatric cancer registries), and reported data for both sexes. Pathology-based cancer registries were included if they had a defined population. Hospital-based cancer registries were excluded. Redundant cancer registry data were excluded from either the final incidence data input or the MIR model input if a more detailed source (e.g., providing more detailed age or diagnostic groups) was available for the same population. Preference was given to registries with national coverage over those with only local coverage, except those from countries where the GBD study provides subnational estimates. Data were excluded if the coverage population was unknown, except for in high SDI quintile locations with full geographic coverage where the GBD population could be substituted.

A list of the cancer registries included in our analysis and the years covered can be found in eTable . We used all data from GBD 2017 and added registry data from Argentina, Australia, Austria, Bermuda, Canada, Chile, China, Colombia, Germany, Netherlands, Switzerland, United Kingdom, Uruguay, and Yemen. Additional metadata for each source are available in the online GBD citation tool, http://ghdx.healthdata.org/gbd-2019.

Mortality-to-incidence ratio data sources

Most cancer registries only report cancer incidence. However, if a cancer registry also reported cancer mortality, mortality data were also extracted. CR sources with matching incidence and mortality data were used in the mortality to incidence ratio estimation. eTable 2 lists the registries used for the estimation of mortality-to-incidence ratios.

Cancer mortality data in the cause of death database other than cancer registry data

In addition to cancer registry data, the GBD cause of death (CoD) database also contains cancer mortality data originating from multiple sources, including vital registration (VR) and verbal autopsy (VA) data. In countries without VR systems, VA studies are a viable data source to inform CoD. VA data are obtained by trained interviewers who use a standardised questionnaire to ask relatives about the signs, symptoms, and demographic characteristics of recently deceased family members. CoD is assigned based on the answers to the questionnaires. A detailed description of the data sources and processing steps for the cause of death database can be found in the appendix to the GBD 2019 paper "Global burden of 369 diseases and injuries in 204 countries and territories, 1990–2019: a systematic analysis for the Global Burden of Disease Study 2019," as well as in the online GBD citation tool http://ghdx.healthdata.org/gbd-2019.[2]

Bias of categories of input data

Potential biases of the input data included for the CoD database can also be found in the appendix to the GBD 2019 paper "Global burden of 369 diseases and injuries in 204 countries and territories, 1990–2019: a systematic analysis for the Global Burden of Disease Study 2019."[2] Cancer registry data can be biased in multiple ways. A high proportion of ill-defined cancer cases in the cancer registry data requires redistribution of these cases to other cancers, which introduces a potential for bias. Changes between coding systems can lead to artificial differences in disease estimates; however, we adjust for this bias by mapping the different coding systems to GBD cancer causes. Underreporting of cancers that require advanced diagnostic techniques (e.g., leukemia, brain, pancreatic, and liver cancer) can be an issue in cancer registries from low-income countries. On the other hand, misclassification of metastatic sites as primary cancer can lead to overestimation of cancer sites that are common sites for metastases (e.g., brain or liver cancer). Since many cancer registries are located in urban areas, the representativeness of the registry for the general non-urban population can also be problematic. The accuracy of mortality data reported in cancer registries usually depends on the quality of the vital registration system. If the vital registration system is incomplete or of poor quality, the mortality-to-incidence ratio can be biased to lower ratios.

Data analysis

Flowcharts describing the conceptual overview of the processing of cancer data are available in eFigure 1 and eFigure 2.

Cancer registry data processing

Cancer registry data goes through multiple processing steps before entering the CoD database.

1. Formatting incidence and mortality data. First, the original data are transformed into standardized files, which included standardization of format, categorization, and registry names (#1 in eFigure 1).

2. Subtotal recalculation. Some cancer registries report individual codes as well as aggregated totals. An example of this would be where the registry data reports C18, C19, and C20 individually, and also the aggregated group of C18–C20 (colon and rectum cancer). The data processing step, "subtotal recalculation" (#2 in eFigure 1), verifies these totals and subtracts the values of any individual codes from the aggregates.

3. Mapping data to GBD causes. In the third step (#3 in in eFigure 1), cancer registry incidence data and cancer registry mortality data are mapped to GBD causes. A different map is used for incidence and for mortality data because of the assumption that there are no deaths for certain cancers. One example is basal cell carcinoma of the skin. In the cancer registry incidence data, basal cell carcinoma is mapped to non-melanoma skin cancer (basal cell carcinoma). However, if basal cell skin cancer is recorded in the cancer registry mortality data, the deaths are instead mapped to non-melanoma skin cancer (squamous cell carcinoma) under the assumption that they were actually misclassified squamous cell skin cancers. Another example is benign or in situ neoplasms. Because cancer registries do not collect non-malignant neoplasms in a standardized way, any benign or in situ neoplasms reported in a cancer registry incidence dataset are dropped from that dataset. The same neoplasms reported in a cancer registry mortality dataset are instead mapped to the respective invasive cancer. For example, cases of "ductal carcinoma in situ" in a cancer registry incidence dataset are dropped from the dataset, while deaths

from “ductal carcinoma in situ” in a cancer registry mortality dataset are mapped to breast cancer. Maps of ICD- codes to GBD causes for incidence and mortality data can be found in eTable 4 and eTable 5.

4. Age/sex splitting. In the fourth data processing step (#4 in in eFigure 1), cancer registry data are standardized to the GBD age groups. The GBD age groups reported in the GBD 2019 study include four age categories under 5 years of age: early neonatal (0-6 days), late neonatal (7-27 days), post-neonatal (28-364 days), and 1-4 years. These are followed by 19 adolescent and adult age categories binned by five years: 5-9 years, 10-14 years, etc., up to the terminal age group of 95 years and older. For each cancer, the minimum age group estimated was determined as the youngest age-group where SEER reported at least 50 cases over the period 1990 to 2015.[7] The modeled starting and ending age groups for each cancer are reported in eTable 7 and 8. Reference global age-specific incidence rates were generated using hospital inpatient data as described in Section 4.3 of the appendix to the GBD 2019 paper “Global burden of 369 diseases and injuries in 204 countries and territories, 1990–2019: a systematic analysis for the Global Burden of Disease Study 2019.”[2]. Reference age-specific mortality rates were generated using aggregated deaths from processed VR data, using the approach described in Section 2.5 of the appendix to the aforementioned GBD 2019 paper.

For incidence or mortality datasets that require age-splitting, age-specific proportions are then generated by applying the reference age-specific rates to the registry population to produce the expected number of cases (or deaths for a mortality dataset) for that registry by age. The expected number of cases (or deaths) for each sex, age, and cancer were normalized to 1, creating final, age-specific proportions. These proportions were then applied to the total number of cases (or deaths) by sex and cancer to get the GBD age group-specific number of cases (or deaths) related to that dataset.

In the rare case that the cancer registry only contains data for both sexes combined, the age-specific cases or deaths are split and reassigned to separate sexes using the same weights that are used for the age-splitting process. Starting from the expected number of deaths, global proportions are generated by sex for each age. For example, if for ages 15-19 years old there are 6 expected deaths for males from cause of death data and 4 expected deaths for females, then 60% of the combined-sex deaths for ages 15-19 years would be assigned to males and the remaining 40% would be assigned to females.

5. Cause disaggregation. In the fifth step (#5 in eFigure 1), data for cause entries that are aggregates of GBD causes were redistributed across those GBD causes. Examples of these aggregated causes include some cancer registries reporting ICD-10 codes C00-C14 together as “lip, oral cavity, and pharyngeal cancer.” These groups are broken down into subcauses that can be individually mapped to single GBD causes. In this example, the more specific ICD-10 codes within C00-C14 are “lip and oral cavity cancer” (C00-C08), “nasopharynx cancer” (C11), “cancer of other parts of the pharynx” (C09-C10, C12-C13), and “Malignant neoplasm of other and ill-defined sites in the lip, oral cavity, and pharynx” (C14). To redistribute the data, weights were created using the same “rate-applied-to-population” method employed in age-sex splitting (see step four above). For the undefined code (C14 in the example) an “average all cancer” weight was used, calculated on the high-quality cancer registry data from SEER/NORDCAN/CI5 by dividing the sum of the cases across these registries by the combined population across these registries. Then, proportions were generated by subcause for each aggregate cause as in the sex-splitting example above (see step four). The total number of cases from the aggregated group

(C00-C14) was recalculated for each subgroup and the undefined code (C14). C14 was then redistributed as a “garbage code” in step six. For two exceptions, C44 (non-melanoma skin cancer) and C46 (Kaposi’s sarcoma), fixed proportions were used to redistribute into GBD causes. Non-melanoma skin cancer processing is described under section “Input data and methodological summary for non-melanoma skin cancer (squamous-cell carcinoma).” C46 entries were primarily redistributed to HIV according to age (100% for age <15 years, 95% for age 15-49 years, and 90% for age ≥50 years), with the remainder redistributed to the GBD cancer cause “Other malignant neoplasms.”

6. Redistribution. In the sixth step (#6 in in eFigure 1), unspecified ICD codes (“garbage codes”) such as “ill-defined cancer site” (for example, C76 or C80) are redistributed across relevant causes estimated within the GBD hierarchy. Redistribution of cancer registry incidence and mortality data mirrored the process of the redistribution used in the cause of death database and utilized the same redistribution maps as specified in Section 2.4 of the appendix to the GBD 2019 Diseases and Injuries capstone, “Global burden of 369 diseases and injuries in 204 countries and territories, 1990–2019: a systematic analysis for the Global Burden of Disease Study 2019”.[2] Sources and targets of garbage codes can be found in eTable 6.

7. Removal of duplicates. In the seventh step (#7 in in eFigure 1), duplicate or redundant data sources were removed from the processed cancer registry dataset. Duplicate sources were present if, for example, a cancer registry was part of the CI5 database but we also had data from that registry directly. Redundancies occurred and were removed as described in “Cancer Incidence Data Sources,” where more detailed data were available, or when national registry data could replace regionally representative data. From here, two parallel selection processes were run; one to generate input data for the mortality-to-incidence ratio (MIR) models, and one to generate incidence for final mortality estimation. When creating the final incidence input, higher priority was given to registry data from the most standardized source; whereas for the MIR model input, only sources that reported both incidence and mortality were used.

8. Combine matching incidence and mortality data and model MIRs. In the eighth step (#8 in in eFigure 1), the processed incidence and mortality data from cancer registries were matched by cancer cause, age, sex, year, and location to generate MIRs (eTable 6). The resulting MIRs were used as input for a three-step modeling approach using the general GBD spatiotemporal Gaussian process regression (ST-GPR)[3] approach, with the Healthcare Access and Quality (HAQ) Index as a covariate in the linear mixed effects model using logit-transformed MIR as the outcome.[21]

$$\text{logit}\left(MIR_{c,a,s,t}\right) = \alpha + \beta_1 HAQIndex_{c,t} + \sum_{a}^{A} \beta_2 I_a + \beta_3 I_s + \epsilon_{c,a,s,t}$$

MIR: mortality-to-incidence ratio
c: country (or subnational for subnationally modeled locations), a: age group, t: time (years); s: sex
HAQIndex: Healthcare Access and Quality Index
I: indicator variable
$\epsilon_{c,a,s,t}$: error term

Predictions were made without the random effects. The ST-GPR model has three main hyper-parameters that control for smoothing across time, age, and geography.[2] These hyper-parameters were adjusted for GBD 2019 in order to improve model performance in locations with sparse data. The time adjustment parameter lambda (λ) aims to borrow strength from neighboring time points (i.e., the value in this year is highly correlated with the value in the previous year but less so further back in time). For GBD 2019, lambda was lowered from 2 to 0.05, increasing the weight of more distant years. The age adjustment parameter omega (ω) borrows strength from data in neighboring age groups and was lowered from 1.0 to 0.5, increasing the weight of more distant age groups. The space adjustment parameter zeta (ξ) aims to borrow strength across the hierarchy of geographical locations. Zeta was lowered from 0.95 to 0.01, reducing the weight of more distant geographical data at the region or super region level. For the remaining parameters in the Gaussian process regression, we lowered the amplitude from 2 to 1 (reducing fluctuation from the mean function) and reduced the scale value from 15 to 10 (reducing the time distance over which points are correlated). Compared to GBD 2017 models, these model specification changes generally led to more smoothing of the MI ratio estimates across age and time, and less geographic smoothing at the region or super region level.

Data-cleaning steps for MIR estimation were similar to those for GBD 2017. For each cancer, MIRs from locations in HAQ Index quintiles 1-4 were dropped if they were below the median of MIRs from locations in HAQ Index quintile 5. We also dropped MIRs from locations in HAQ Index quintiles 1-4 if the MIRs were above an outlier threshold calculated as the third quartile + 1.5 * IQR (inter-quartile range). We dropped all MIR data that were based on fewer than 15 incident cases to avoid excessive variation in the ratio due to small numbers (this threshold was 25 cases in GBD 2017, but was lowered in GBD 2019 in order to include additional data). An exception to this threshold was made for mesothelioma and acute myeloid leukemia, where instead we dropped MIRs that were based on fewer than ten cases because of lower data availability for these two cancers. For the lower end of the age spectrum where cancers are generally rarer, we also aggregated incidence and mortality to the youngest five-year age bin where SEER reported at least 50 cases from 1990 to 2015, to avoid unstable MIR predictions in young age groups because of too few data. The MIR estimates in this SEER-based minimum age-bin were then copied down to all younger GBD age groups estimated for that cancer.

Since MIRs can be above 1, especially in older age groups and cancers with low cure rates, we used the 95th percentile (by age group) of the cleaned dataset (detailed above) to cap the MIR input data. The addition of new data for GBD 2019 led to slightly different upper caps compared to GBD 2017:

**Age group (years)**	**Maximum MIR, GBD 2017**	**Maximum MIR, GBD 2019**
0 to 4	0.56	0.71
5 to 9	0.71	0.73
10 to 14	0.84	0.80
15 to 19	0.86	0.65
20 to 24	0.65	0.72
25 to 29	0.59	0.78
30 to 34	0.63	0.87
35 to 39	0.73	0.91
40 to 44	0.83	0.97

45 to 49	0.86	0.93
50 to 54	0.89	0.93
55 to 59	0.91	0.95
60 to 64	0.96	0.99
65 to 69	1.01	1.03
70 to 74	1.09	1.10
75 to 79	1.22	1.19
80 to 84	1.36	1.27
85 to 89	1.39	1.35
90 to 94	1.45	1.53
95+	1.87	1.84

These "upper cap" values were used to allow MIRs over 1 in some age groups but to constrain the MIRs to a maximum level. Any MIR values over this cap were Winsorized to the cap value. To run the logit model, the input data were first divided by the upper caps to get proportional data ranging from 0 to 1. Model predictions from ST-GPR were then rescaled back by multiplying them by the upper caps. To constrain the MIRs at the lower end, we used the fifth percentile of the cancer and age-specific cleaned MIR input data to Winsorize all model predictions below this lower cap.

9. Generate mortality estimates from incidence and MIRs
Final estimated MIRs were matched with the cleaned cancer registry incidence dataset finalized in the ninth step (#9 in eFigure 1) to generate mortality estimates (#10 in eFigure 1):

$$MIR_{estimates} * \ incidence_{registry} = mortality_{CR\ inputs}$$

These mortality estimates are then smoothed by a Bayesian noise-reduction algorithm (to deal with zero counts; this is also applied to the VR and VA data), as specified in Section 2.14 of the appendix to the GBD 2019 paper "Global burden of 369 diseases and injuries in 204 countries and territories, 1990–2019: a systematic analysis for the Global Burden of Disease Study 2019."[2] These data were uploaded into the CoD database as CR data (#11 in the flowchart). Cancer-specific mortality modelling then followed the general CODEm process using the totality of VA, VR, and CR data.

Cause of death database formatting

Formatting of data sources for the cause of death (CoD) database, including VR and VA data, is similar to many of the steps outlined above for CR data (#11 in eFigure 1) and is described in Section 2 of the appendix to the GBD 2019 paper "Global burden of 369 diseases and injuries in 204 countries and territories, 1990–2019: a systematic analysis for the Global Burden of Disease Study 2019."[2]

VA data may not capture cancer deaths as accurately or comprehensively as cancer registries or vital registration systems, but provides a useful contribution to cancer models in locations without VR or CR data. Additional processing and restrictions are performed on VA data to ensure quality standards and feasible inputs. More details on VA data processing are provided in the appendix noted above, particularly Sections 2.2 (VA overview), 2.10 (VA cause restrictions), 2.14 (noise reduction), 2.15 (outlier identification), and 2.16 (data quality ratings).

CODEm models

Mortality estimates for each cancer were generated using the GBD Cause of Death Ensemble model (CODEm, #12 in eFigure 1) approach, the methods of which have been described in previous publications.[2,22] Additional details are specified in Section 3.1 of the appendix to the GBD 2019 paper "Global burden of 369 diseases and injuries in 204 countries and territories, 1990–2019: a systematic analysis for the Global Burden of Disease Study 2019."[2]

In brief, the CODEm approach is based on several principles: that all types of available data should be used, even if data quality varies; that a diverse set of plausible models with different combinations of covariates should be evaluated; that both individual models and the overall ensemble models should be tested for their predictive validity; and that the best model or sets of models should be chosen based on the out–of-sample predictive validity.

Covariates are provided for potential use in the ensemble based on a possible predictive relationship between the covariate and the specific cancer mortality, with an expected level and direction of association. Generally, Level 1 covariates have a proven strong relationship with the outcome, such as etiological or biological roles. Level 2 covariates have a strong relationship but not a known direct biological link. Level 3 covariates have a relationship that may be more distal in the causal chain, or are mediated through Level 1 or 2 covariates.[22] The covariates provided to CODEm, as well as their level and direction, differ by cause and sex. The covariates used for 2019 can be found in eTable 7, and differences between GBD 2017 and GBD 2019 covariates can be found in eTable 8.

To generate an ensemble model, CODEm generates submodels that evaluate all plausible relationships between covariates and the response variable. Three additive components of data variance are used in CODEm: sampling variance, non-sampling variance, and garbage code redistribution variance. Model performance of all models is evaluated through out-of-sample predictive validity tests. Ensemble models are constructed from the individual models, with the contribution of individual models to the ensemble weighted by the basis of their predictive validity ranking. The final ensemble contains 1000 draws from these individual component models, from which a mean estimate and a 95% uncertainty interval are calculated. The 95% uncertainty interval represents the 0.025 and 0.975 quantiles of the draws.

Liver cancer etiology split models

The level 3 cause "liver cancer" is estimated as above, while additional methods are utilized for the underlying level 4 causes. For the level 4 causes of liver cancer, the level 3 total deaths are proportionally split into the five etiology groups included in GBD 2019: 1) Liver cancer due to hepatitis B, 2) Liver cancer due to hepatitis C, 3) Liver cancer due to alcohol, 4) Liver cancer due to nonalcoholic steatohepatitis (NASH), and 5) Liver cancer due to other causes. To find data to inform these proportions, a systematic literature search was performed in PubMed on 10/24/2016 using the following search string:

> ("liver neoplasms"[All Fields] OR "HCC"[All Fields] OR "liver cancer"[All Fields] OR "Carcinoma, Hepatocellular"[Mesh]) AND (("hepatitis B"[All Fields] OR "Hepatitis B"[Mesh] OR "Hepatitis B virus"[Mesh] OR "Hepatitis B Antibodies"[Mesh] OR "Hepatitis B Antigens"[Mesh]) OR ("hepatitis C"[All Fields] OR "Hepatitis C"[Mesh] OR "hepatitis C antibodies"[MESH] OR "Hepatitis C Antigens"[Mesh] OR "Hepacivirus"[Mesh]) OR ("alcohol"[All Fields] OR "Alcohol

Drinking"[Mesh] OR "Alcohol-Related Disorders"[Mesh] OR "Alcoholism"[Mesh] OR "Alcohol-Induced Disorders"[Mesh])) NOT (animals[MeSH] NOT humans[MeSH])".

Studies were included if the study population was representative of the liver cancer population for the respective location. Several studies not initially found through this search but that were included in the meta-analysis by de Martel et al, were added.[23] We also included the study by Hong et al, after the authors provided us with additional data on the overlap in etiologies.[24]

For each study, the proportions of liver cancer due to the five specific etiologies were calculated. Cases were considered to be due to NASH when the manuscript explicitly listed the etiology to be NASH or non-alcoholic fatty liver disease (NAFLD). Cases where the etiology was listed as "cryptogenic," "idiopathic," or "unknown" were included within the "other causes" category. In manuscripts where the etiology for a case was not known but major categories could not be ruled out (for example, if the study tested for hepatitis B and C, but did not assess alcohol use), only the explicitly defined proportions were included (in this example, including proportions for hepatitis B and C, and excluding any remainder). Remaining etiologies were included under a combined "other" group (for example, hemochromatosis, autoimmune hepatitis, Wilson's disease, etc.). If multiple etiologies were reported for an individual patient, these were apportioned proportionally to the individual etiologies.

The proportion data found through the systematic literature review were used as input for five separate DisMod-MR 2.1[2] models to determine the proportion of liver cancers due to the five subgroups for all locations, both sexes, and all GBD age groups (step #16 in eFigure 1). For liver cancer due to hepatitis C and hepatitis B, a prior value of 0 percent was set between age 0 and 0.01 years. For liver cancer due to alcohol, a prior value of 0 percent was set for ages 0 to 5 years. Covariates differed by model and direction. The liver cancer due to alcohol model included positive covariates for the liters of alcohol consumed *per capita* and the proportion of cirrhosis due to alcohol, and a negative covariate for the proportion of alcohol abstainers. The liver cancer due to hepatitis B model included positive covariates for hepatitis B seroprevalence (HBsAg) and the proportion of cirrhosis due to hepatitis B, and a negative covariate for 10-year lagged hepatitis B 3-dose vaccine coverage. The liver cancer due to hepatitis C model included positive covariates for hepatitis C seroprevalence (anti-HCV) and the proportion of cirrhosis due to hepatitis C. The liver cancer due to NASH model included positive covariates for mean body mass index, the prevalence of obesity, the prevalence of NASH and non-alcoholic fatty liver disease, and the proportion of cirrhosis due to NASH. The liver cancer due to other causes model included a positive covariate for the proportion of cirrhosis due to other causes.To ensure consistency between cirrhosis and liver cancer estimates and to take advantage of the data for the related causes (e.g., "liver cancer due to hepatitis C" and the related cause "cirrhosis due to hepatitis C"), we generated covariates from initial liver cancer proportion models that were used in the cirrhosis etiology proportion models. Estimates from the cirrhosis etiology proportion models were then used to make covariates for use in the final liver cancer etiology models. More information about cirrhosis modeling can be found in the "Cirrhosis" section of the appendix to the GBD 2019 paper "Global burden of 369 diseases and injuries in 204 countries and territories, 1990–2019: a systematic analysis for the Global Burden of Disease Study 2019."[2]

Since the proportion models were run independently of each other, the final proportion estimates were scaled to sum to 100% within each age, sex, year, and location, by dividing each proportion by the sum of the five (step # 17 in eFigure 1). For the liver cancer subtype mortality estimates, we multiplied the

parent cause "liver cancer" deaths by the corresponding scaled proportions (step # 18 in eFigure 1). Single cause estimates were later adjusted to fit into the separately modeled all-cause mortality in the process CoDCorrect.

CoDCorrect

CODEm models estimate the individual cause-level mortality without taking into account the independently modeled all-cause mortality (#13 in eFigure 1). To ensure that all single causes add up to the all-cause mortality and that all child-causes add up to the parent cause, an algorithm called "CoDCorrect" is used (#14 and #15 in eFigure 1). Further details on the CoDCorrect algorithm can be found in Section 3.3.2 of the appendix to the GBD 2019 paper "Global burden of 369 diseases and injuries in 204 countries and territories, 1990–2019: a systematic analysis for the Global Burden of Disease Study 2019."[2] Final mortality estimates at the 1000-draw level provide an estimated mean mortality with 95% uncertainty interval.

Calculating YLLs

To calculate years of life lost (YLLs), final death estimates after CoDCorrect adjustment are multiplied by the standard GBD life expectancy given the age at death, sex, and location. Further details on GBD life expectancy values can be found in the GBD 2019 paper "Global age-sex-specific fertility, mortality, health life expectancy (HALE), and population estimates in 2014 countries and territories, 1950-2019: a comprehensive demographic analysis for the Global Burden of Disease Study 2019."[4] Uncertainty is propagated from the CoDCorrect mortality estimates, calculating YLLs for each of the 1000 CoDCorrect draws to provide estimated mean YLLs with corresponding 95% uncertainty intervals.

Incidence estimation

The final GBD cancer mortality estimates (after CoDCorrect adjustment) were transformed to incidence estimates by using the MIRs specific to that cancer cause (#1 in eFigure). Final mortality estimates at the 1000-draw level were divided by the modeled MIR estimates (also at the 1000-draw level) to generate 1000 draws of incidence estimates (which provides an estimated mean incidence with 95% uncertainty interval). It was assumed that uncertainty in the MIRs is independent of uncertainty in the estimated mortality.

Prevalence estimation

After transforming the final GBD cancer mortality estimates to incidence estimates (step 1 in eFigure 2), incidence was combined with annual relative survival estimates from 1 to 10 years after diagnosis (step 7 in the flowchart). Prior to GBD 2017 we used an access to care metric to scale survival for each location between two survival curves representing presumed "best case" and "worst case" survival.[25,26] For GBD 2017, we updated this method in order to more directly utilize MIRs to estimate yearly cancer relative survival.[1] For GBD 2019 we updated these methods to estimate age-specific rather than all-ages survival curves.

Previous reports suggest that the value of (1 – MIR) may serve as a proxy for 5-year relative survival, with the exact correlation varying slightly by cancer type.[27] Because this correlation varies, we trained cancer-specific prediction models to estimate 5-year survival from MIRs, using data from SEER[7]. We used SEER*Stat[28] to obtain mortality, incidence, and relative survival statistics from the nine SEER registries reporting from 1980-2014 (step 2), by cancer type, sex, 5-year blocks (i.e., 1980-84, 1985-1989, etc.), and 5-year age groups (except combining 80+). For each cancer, we modelled SEER 5-year relative

survival using MIRs calculated from SEER mortality and incidence. For GBD 2019 we updated this model from the Poisson regression used in GBD 2017[1] to using a generalized linear model with a quasibinomial family and logit link, weighted by the number of index cases (step 3 in eFigure 2). To reduce variability due to small samples, we only included MIRs based on at least 25 incident cases (except for the cancers mesothelioma, nasopharynx cancer, and acute lymphoid leukemia, where MIRs based on at least 10 cases were included). These models were then applied to the GBD MIR estimates to predict an estimated 5-year survival for each age/sex/year/location (step 4). To prevent unrealistic values, predicted 5-year survival values were Winsorized to be between 0% and 100% survival. Unlike GBD 2017, we did not require the estimated age-specific 5-year survival to be greater than the all-ages worst-case survival scenario from SurvCan and US 1950 survival data[29,30], since age-specific survival could be plausibly lower than for these all-ages scenarios.

To generate yearly survival estimates up to 10 years, in GBD 2019 we downloaded SEER sex- and age-specific annual 1- through 10-year relative survival data from persons diagnosed between 2001 and 2010 (2001 through 2010 so that all cases had at least 5 years of follow-up, with half having the full 10 years of follow-up). This is updated from GBD 2017, where we downloaded all-ages survival data from persons diagnosed in 2004 (2004 so that all cases had the full 10 years of follow-up).[31] A proportional scalar was calculated as the predicted GBD 5-year survival estimate divided by the SEER 5-year survival statistic, and was then used to generate yearly survival estimates by scaling the 1-10 year SEER curve to the GBD survival predictions under the proportional hazard assumption (step 5). This change from GBD 2017 (where we used SEER all-ages data from 2004 as the scalar and survival curve) impacts prevalence and YLD estimation, generally leading to survival estimates that are higher for younger ages and lower for older ages compared to estimates using the all-ages curve.

The estimated relative survival is next transformed into absolute survival estimates (steps 6 and 7 in eFigure 2). To account for background mortality in the relative survival estimates, GBD 2019 lifetables were used to calculate lambda (λ) values:

$$\lambda = \frac{\ln\left(\frac{nLx_n}{nLx_{n+1}}\right)}{5}$$

nLx = person years lived between ages x and x+n (from GBD lifetable).

Absolute survival was then calculated using these lambda values in an exponential survival function:

$$absolute\ survival = relative\ survival * e^{\lambda * t}$$

t = time (in years)

Absolute survival is combined with incidence to estimate the prevalence at each year 1 through 10 after diagnosis, which is then split into the four sequelae (step 8 in the flowchart). For the purposes of calculating disability due to cancer, survivors beyond 10 years were considered cured. For this group, the survivor population prevalence was divided into two sequelae: (1) diagnosis and primary therapy phase; and (2) controlled phase. For the population that did not survive beyond 10 years, the yearly prevalence was divided into the four sequelae by assigning the fixed durations for each of the (1) diagnosis and primary therapy phase, (3) metastatic phase, and (4) terminal phase, and assigning the remaining prevalence to the (2) controlled phase (step 8 in the flowchart). Duration of these four

sequelae remained the same as for GBD 2013,[32] GBD 2015,[25] GBD 2016,[26] and GBD 2017.[1] eTable 12 lists the duration of each, along with the sources used to determine their length.[33–38]

For cancer-specific procedure sequelae, hospital data were used to estimate the number of cancer patients undergoing mastectomy, laryngectomy, stoma, prostatectomy, and cystectomy (step 9 in the flowchart). Proportions were generated by dividing the rate of procedures generated from the diagnostic codes in the hospital dataset and the coverage population by the GBD age-, and sex-specific disease incidence rates for that country.

To estimate procedure-related disability for each of these five cancers, the procedure proportions (proportion of each cancer population that undergo these procedures) from hospital data were used as input for a proportion model in DisMod-MR 2.1[2] to estimate the proportions for all locations, by age, year, and sex. Details of clinical and claims data processing are available in section 4.3.4 of the appendix to the GBD 2019 paper "Global burden of 369 diseases and injuries in 204 countries and territories, 1990–2019: a systematic analysis for the Global Burden of Disease Study 2019."[2]

Since colostomy or ileostomy procedures are done for reasons other than cancer, a literature review was conducted to determine the proportion of ostomies due to colon and rectum cancer. Based on the results of the literature review that an average of 58% of ostomies are done for colon and rectum cancer, the "all cause" colostomy proportions were multiplied by 0.58.[39–41]

The final procedure proportions were applied to the incident cases of the respective cancers and multiplied with the proportion of the incident population surviving for 10 years to determine the incident cases of the cancer population that underwent procedures and that survived beyond 10 years. These incident cases were used again as an input for DisMod-MR 2.1, with a remission specification of zero and an excess mortality rate prior of 0 to 0.1, as well as with increasing both the age of the population and the year by 10 years to reflect prevalence after that population has survived 10 years. The results from this model are incidence and lifetime prevalent cases of persons with these cancer-related sequelae who have survived beyond 10 years.

Since disability associated with prostatectomy comes from impotence and incontinence, and not from the prostatectomy itself, 18% of the prostatectomy prevalence was assumed to have incontinence and 55% was assumed to have impotence, based on a literature review done for GBD 2013.[42–49] Cases were assigned disability for either impotence or incontinence, but no cases were assigned disability from both.

We assumed that for the population surviving up to 10 years, only the prevalence population being in remission experiences additional disability due to procedures (e.g. women suffering from metastatic breast cancer do not experience additional disability due to a mastectomy during this phase). To estimate the prevalence of the cancer population in remission during the first 10 years after diagnosis with and without procedure-related disability, we multiplied the prevalence of the population in the remission phase with the proportion of the population undergoing a procedure. This step allowed us to estimate disability during the remission phase for both the population experiencing disability due to the remission phase alone, as well as the population experiencing disability from the remission phase and the additional procedure-related disability.

Lastly, the procedure sequelae prevalence and general sequelae prevalence were multiplied with their respective disability weights (eTable 12) to obtain the number of YLDs (steps 11 and 12 in the flowchart). The methods used to generate disability weights are described in Section 4.8, "Disability weights" in the appendix to the paper "Global burden of 369 diseases and injuries in 204 countries and territories, 1990-2019: a systematic analysis for the Global Burden of Disease Study 2019."[2] In brief, disability weights are created from survey data to represent the magnitude of health loss associated with an outcome. These disability weights range from 0, implying a state equivalent to full health, to 1, a state equivalent to death. The sum of these YLDs is the final YLD estimate associated with each cancer cause.

Reporting Standards

Countries and territories reported can be found in eTable . All rates are reported per 100 000 person-years. Annualized rates of change (ARC) from 2010 to 2019 represent the average percentage change per year over this period, and are calculated as:

$$ARC = \frac{\ln\left(\frac{X\_y2}{X\_y1}\right)}{y2 - y1}$$

X_yn = value of measure (e.g. incidence) at year $\_y_n$

y1 = starting year (e.g. 2010)

y2 = ending year (e.g. 2019)

The GBD world population age standard was used to calculate age-standardized rates presented throughout GBD. In GBD 2017 and 2019, we used the non-weighted mean of the GBD year's age-specific proportional distributions for national locations with populations greater than 5 million in the GBD year to update the world population age standard.[4] The final values used for the age standard are specified in Appendix Table 13 of the GBD 2019 paper "Global age-sex-specific fertility, mortality, health life expectancy (HALE), and population estimates in 204 countries and territories, 1950-2019: a comprehensive demographic analysis for the Global Burden of Disease Study 2019."[4]

Socio-demographic Index (SDI)

Methods for the calculation of SDI and SDI quintiles are described in appendix section 2.10 of the GBD 2019 paper "Global burden of 369 diseases and injuries in 204 countries and territories, 1990-2019: a systematic analysis for the Global Burden of Disease Study 2019."[2] In brief, SDI is a summary indicator to represent background levels of social and economic conditions that can influence health outcomes in a given location. This summary indicator comprises three indices: lag-distributed income *per capita*, mean education for those aged 15 years or older, and total fertility rate for those younger than 25 years of age. Possible values for each of these three indices range from 0 to 1, representing the bounds with which lower or higher values of the level of development for that index would no longer worsen or improve health outcomes, respectively. The composite SDI is the geometric mean of these three indices for a given location-year. For reporting purposes, values were multiplied by 100 to obtain SDI on a scale of 0 to 100. The SDI cutoffs for determining SDI quintiles for analysis were computed by using the country-level estimates of SDI for the year 2019, excluding countries with populations less than 1 million. For GBD 2019 analyses, all locations are assigned to these quintiles according to their SDI value

in the year 2019.

	Low SDI	Low-middle SDI	Middle SDI	High-middle SDI	High SDI
SDI value range in 2019	0 - 45.98	45.99 - 60.96	60.97 - 70.19	70.20 - 80.71	80.72 - 100
Number of countries and territories	35	42	41	46	40
Total population in 2019, millions (95% UI)	1 130 (1 080 - 1 170)	1 760 (1 670 - 1 860)	2 400 (2 280 - 2 510)	1 430 (1 350 - 1 510)	1 010 (966 - 1 060)
Total population in 2010, millions (95% UI)	956 (931 - 979)	1 360 (1 320 - 1 390)	2 200 (2 140 - 2 270)	1 570 (1 510 - 1 620)	901 (873 - 929)
Female population in 2019, millions (95% UI)	563 (540 - 585)	878 (833 - 923)	1 190 (1 130 - 1 250)	716 (676 - 753)	508 (484 - 531)
Female population in 2010, millions (95% UI)	481 (469 - 493)	681 (662 - 699)	1 090 (1 060 - 1 120)	775 (747 - 800)	447 (434 - 461)
Male population in 2019, millions (95% UI)	566 (543 - 589)	886 (840 - 934)	1 210 (1 150 - 1 260)	715 (674 - 753)	506 (482 - 528)
Male population in 2010, millions (95% UI)	475 (463 - 486)	675 (655 - 695)	1 110 (1 080 - 1 150)	792 (763 - 819)	454 (440 - 468)

Interpretation of results

Changes in GBD 2019 methods and results from GBD 2017

Cancer mortality estimates for GBD 2019 can differ from the GBD 2017 results for multiple reasons. Updated cancer mortality data were added from vital registration systems and updated incidence and mortality data from cancer registries. Previously all deaths with ICD-10 code C22.9 ("Malignant neoplasm of liver, not specified as primary or secondary") were mapped to liver cancer, but included deaths from liver metastases rather than primary liver cancer alone; for GBD 2019, these deaths were instead mapped as garbage codes and redistributed to liver and 13 other cancers (colon and rectum, pancreas, prostate, breast, esophagus, bladder, kidney, stomach, ovary, uterus, cervix, gallbladder, testes). The mortality-to-incidence ratio estimation was updated with lower case inclusion criteria and different model hyperparameters compared to GBD 2017, leading to more training data and less smoothing across age and time. Covariates and model parameters used in CODEm models were updated for GBD 2019. This included removing or replacing covariates that had been updated by other GBD teams (primarily dietary covariates), assigning a prior for direction of association to all covariates (previously covariates such as income and Socio-demographic Index had been allowed to have agnostic direction priors), and changing the minimum age ranges for which the models estimated mortality. For GBD 2019, the minimum age group estimated for each cancer was set as the youngest age group where at least 50 incident cancers were reported in SEER over the 25-year period 1990-2015. Though based solely on incidence data from the United States, this represents a data-driven improvement over prior GBD cycles, where age restriction was based on expert opinion. An area of future development will be to evaluate this cutoff using data from additional locations.

Comparison to GLOBOCAN

Another major group producing country-level cancer mortality estimates is the International Agency for Research on Cancer (IARC) with their GLOBOCAN database.[50] Significantly different methods between the GBD study and GLOBOCAN can lead to differences in results. For GLOBOCAN, estimates are produced separately at the national level, using several different regression or imputation models differentially by country depending on the data available.[51] For the GBD, cancer estimation occurs globally across all locations following a consistent, well-documented ensemble modeling approach that includes relevant covariate data, which allows cross-validation of models as well as determination of uncertainty. Another major difference is the ability in the GBD study to adjust individual causes of death to the all-cause mortality envelope, which is estimated separately, allowing GBD to correct for the under-diagnosis of cancer in countries with inadequate diagnostic resources. Redistribution of a fraction of undefined causes of death to certain cancers is another methodological advantage of the GBD study, and estimates for cancer mortality can therefore differ substantially in countries with a large proportion of undefined causes of deaths in their vital registration data or a large proportion of undefined cancer

cases in their cancer registry data. There are also differences in the inclusion and categorization of cancer types reported: basal cell carcinoma cases are included in GBD total cancer incidence estimates, but excluded in GLOBOCAN; some cancers are individually reported in GLOBOCAN (e.g., penis cancer), which in the GBD are included within the "other malignant neoplasms" group.

Limitations

There are certain limitations to consider when interpreting the GBD mortality cancer estimates. First, even though every effort is made to include the most recently available data for each country, data seeking resources are not limitless and new data cannot always be accessed as soon as they are made available. It is therefore possible that the GBD study does not include all available data sources for cancer incidence or cancer mortality. Second, different redistribution methods can potentially change the cancer estimates substantially if the data sources used for the estimated location contain a large number of undefined causes; however, neglecting to account for these undefined deaths would likely introduce an even greater bias in the disease estimates. Third, using mortality-to-incidence ratios to transform cancer registry incidence data to mortality estimates requires accurate MIRs. For GBD 2019 we have made further refinements to the estimation of MIRs, but the method remains sensitive to under-diagnosis of cancer cases or under-ascertainment of cancer deaths. However, given that the majority of data used for the cancer mortality estimation come from vital registration data and not cancer registry data, this is not a major limitation. There are several geographic locations where estimates are not available (e.g. Western Sahara, French Guiana) as they were not modelled locations in the GBD 2019.[2] These countries are colored white in the global map figures included in this paper.

Additional method summaries for Non-melanoma skin cancer (squamous and basal cell carcinoma)

Case definition

The Level 3 cause "non-melanoma skin cancer" (NMSC) is comprised of two Level 4 causes, "basal cell carcinoma" and "squamous cell carcinoma." NMSC in the GBD analysis does not include other types of skin cancer (e.g., melanoma, Merkel cell carcinoma).

Input data

Since mortality estimates for non-melanoma skin cancer are only produced for squamous-cell carcinoma under the assumption that basal-cell carcinoma causes almost no deaths, all mortalities reported as "C44" or "173" were mapped to the "squamous-cell carcinoma" GBD cause. We estimated squamous cell and basal cell skin cancer incidence by using data from cancer registries, primary literature, clinical data, and insurance claims. Only cancer registries that were listed in CI5 as registering squamous cell carcinoma or basal cell carcinoma, respectively, were included in the analysis. Details of clinical and claims data processing are available in section 4.3.4 of the appendix to the GBD 2019 paper "Global burden of 369 diseases and injuries in 204 countries and territories, 1990–2019: a systematic analysis for the Global Burden of Disease Study 2019."[2] Clinical informatics sources include the MarketScan administrative claims database in the United States.[52] For 2019, the clinical data were adjusted to account for differences in healthcare access and quality across time and space, and corrected to account for outpatient encounters that did not warrant an inpatient hospital admission. This is a change from GBD 2017, where these data only included non-primary diagnoses in inpatient admissions. This change in GBD 2019 led to higher values in the input clinical informatics data compared to GBD 2017, as it now includes diagnoses from outpatient procedures that did not require hospital admission (whereas

previously these data approximated the rate of inpatient admissions for NMSC cases who had access to hospitals).

Modeling strategy

Non-melanoma skin cancer deaths are modeled in CODEm using vital registration data, as detailed above. All non-melanoma skin cancer deaths are modeled as squamous cell carcinoma deaths, as we make an assumption that there are no deaths from basal cell carcinoma.

For cancer registry data reported at the three-digit level (i.e., C44: Other and unspecified malignant neoplasm of skin), proportions from Karagas et al were used to split C44 into squamous cell carcinoma and basal cell carcinoma.[53] The only new data compared to GBD 2017 was additional data from hospital and outpatient sources. For both basal cell carcinoma and squamous cell carcinoma, DisMod-MR 2.1[2] models were used to model incidence and prevalence. Prevalence was calculated as a function of two extreme scenarios (duration 1 versus 5 years). Country-, age-, sex-, and year-specific duration was estimated using a country-age-sex-year-specific relative access-to-care-score.

The access to care score was based on the melanoma mortality-to-incidence ratio:

$$Access\ to\ care = 1 - \frac{Age\ standardized\ MIR_{cys} - Age\ standardized\ MIR_{min}}{Age\ standardized\ MIR_{max} - Age\ standardized\ MIR_{min}}$$

c=country; y=year; s=sex; Age-standardized MIR_{min}=lowest MIR for all countries and years; Age standardized MIR_{max}=highest MIR for all countries and years

Remission was calculated as the inverse of the duration estimates and used as additional input for DisMod-MR 2.1.

To reflect differing degrees of disability due to squamous cell carcinoma, we used three levels of severity that were derived from the Medical Expenditure Panel Survey (MEPS),[54] resulting in proportions of 80% mild, 15% moderate, and 5% severe disfigurement. For basal cell carcinoma, disability severity was split into 60% asymptomatic (without disability) and 40% with mild disfigurement. Prevalence was multiplied by distinct disability weights (eTable 13) to generate YLDs.

Interpretation of results

Non-melanoma skin cancer mortality estimates are generally not available from other sources. Even though the data availability for non-melanoma skin cancer is poor, the fact that it is the most common incident cancer, with rates expected to rise globally, makes it important to include the disease in the GBD framework.

Limitations

Cancer registry data for non-melanoma skin cancer incidence have to be interpreted with caution due to a substantial amount of under-reporting or rules that only the first non-melanoma skin cancer has to be registered. Many cancer registries therefore do not include non-melanoma skin cancers at all. However, the information regarding whether registries capture NMSC or not is not consistently available. Therefore, no cancer registry data were used to estimate deaths due to squamous-cell carcinoma of the skin. For vital registration data, we make the assumption that there are no deaths due to basal-cell non-

melanoma skin cancer, and therefore all deaths attributed to basal-cell carcinoma were included instead as squamous-cell carcinoma.

References

1. Fitzmaurice C, Abate D, Abbasi N, et al. Global, Regional, and National Cancer Incidence, Mortality, Years of Life Lost, Years Lived With Disability, and Disability-Adjusted Life-Years for 29 Cancer Groups, 1990 to 2017: A Systematic Analysis for the Global Burden of Disease Study. *JAMA Oncol*. 2019;5(12):1749-1768. doi:10.1001/jamaoncol.2019.2996

2. Vos T, Lim SS, Abbafati C. Global burden of 369 diseases and injuries in 204 countries and territories, 1990–2019: a systematic analysis for the global burden of disease study 2019. *The Lancet*. 2020;396(10258):1204-1222.

3. Murray CJL, Aravkin AY, Zheng P, et al. Global burden of 87 risk factors in 204 countries and territories, 1990–2019: a systematic analysis for the Global Burden of Disease Study 2019. *The Lancet*. 2020;396(10258):1223-1249.

4. Wang H, Abbas KM, Abbasifard M, et al. Global age-sex-specific fertility, mortality, healthy life expectancy (HALE), and population estimates in 204 countries and territories, 1950-2019: a comprehensive demographic analysis for the Global Burden of Disease Study 2019. *Lancet Lond Engl*. 2020;396(10258):1160-1203. doi:10.1016/S0140-6736(20)30977-6

5. Stevens GA, Alkema L, Black RE, et al. Guidelines for Accurate and Transparent Health Estimates Reporting: the GATHER statement. *The Lancet*. 2016;388(10062):e19-e23. doi:10.1016/S0140-6736(16)30388-9

6. World Health Organization. International Classification of Diseases (ICD). Published 2018. Accessed February 25, 2020. http://www.who.int/classifications/icd/en/

7. Surveillance, Epidemiology, and End Results (SEER) Program (www.Seer.Cancer.Gov) SEER*Stat Database: Incidence - SEER 18.

8. Doll R, Payne P, Waterhouse J, eds. *Cancer Incidence in Five Continents, Vol. I*. Union Internationale Contre le Cancer; 1966. Accessed February 24, 2021. https://publications.iarc.fr/Non-Series-Publications/Other-Non-Series-Publications/Cancer-Incidence-In-Five-Continents-Volume-I-1966

9. Doll R, Muir CS, Waterhouse JA. *Cancer Incidence in Five Continents, Vol. II*. Union Internationale Contre le Cancer; 1970.

10. Waterhouse J, Muir C, Correa P, Powell J. Cancer Incidence in Five Continents III. Lyon: IARC; 1976.

11. Waterhouse J, Muir C, Shanmugaratnam K, Powell J. Cancer Incidence in Five Continents IV. Lyon: IARC; 1982.

12. Muir C, Mack T, Powell J, Whelan S. Cancer Incidence in Five Continents V. Lyon: IARC; 1987.

13. Parkin D, Muir C, Whelan S, Gao Y, Ferlay J, Powell J. Cancer Incidence in Five Continents VI. Lyon: IARC; 1992.

14. Parkin D, Whelan S, Ferlay J, Raymond L, Young J. Cancer Incidence in Five Continents VII. Lyon: IARC; 1997.

15. Parkin D, Whelan S, Ferlay J, Teppo L, Thomas D. Cancer Incidence in Five Continents VIII. Lyon: IARC; 2002.

16. Curado M, Edwards B, Shin H, et al. Cancer Incidence in Five Continents IX. Lyon: IARC; 2007. http://www.iarc.fr/en/publ.

17. Forman D, Bray F, Brewster D, et al. Cancer Incidence in Five Continents X. http://ci5.iarc.fr. Published 2013.

18. Bray F, Colombet M, Mery L, et al., eds. *Cancer Incidence in Five Continents*. Vol XI (electronic version). International Agency for Research on Cancer; 2017. https://ci5.iarc.fr

19. Steliarova-Foucher E, O'Callaghan M, Ferlay J, et al. The European Cancer Observatory: A new data resource. *Eur J Cancer Oxf Engl 1990*. 2015;51(9):1131-1143. doi:10.1016/j.ejca.2014.01.027

20. Engholm G, Ferlay J, Christensen N, et al. NORDCAN--a Nordic tool for cancer information, planning, quality control and research. *Acta Oncol Stockh Swed*. 2010;49(5):725-736. doi:10.3109/02841861003782017

21. Barber RM, Fullman N, Sorensen RJD, et al. Healthcare Access and Quality Index based on mortality from causes amenable to personal health care in 195 countries and territories, 1990-2015: a novel analysis from the Global Burden of Disease Study 2015. *Lancet Lond Engl*. 2017;390(10091):231-266. doi:10.1016/S0140-6736(17)30818-8

22. Foreman KJ, Lozano R, Lopez AD, Murray CJ. Modeling causes of death: an integrated approach using CODEm. *Popul Health Metr*. 2012;10:1. doi:10.1186/1478-7954-10-1

23. de Martel C, Maucort-Boulch D, Plummer M, Franceschi S. World-wide relative contribution of hepatitis B and C viruses in hepatocellular carcinoma. *Hepatol Baltim Md*. 2015;62(4):1190-1200. doi:10.1002/hep.27969

24. Hong TP, Gow P, Fink M, et al. Novel population-based study finding higher than reported hepatocellular carcinoma incidence suggests an updated approach is needed. *Hepatol Baltim Md*. 2016;63(4):1205-1212. doi:10.1002/hep.28267

25. Fitzmaurice C, Allen C, Barber RM, et al. Global, Regional, and National Cancer Incidence, Mortality, Years of Life Lost, Years Lived With Disability, and Disability-Adjusted Life-years for 32 Cancer Groups, 1990 to 2015: A Systematic Analysis for the Global Burden of Disease Study. *JAMA Oncol*. 2016;3(4):524-548. doi:10.1001/jamaoncol.2016.5688

26. Fitzmaurice C, Akinyemiju TF, Al Lami FH, et al. Global, Regional, and National Cancer Incidence, Mortality, Years of Life Lost, Years Lived With Disability, and Disability-Adjusted Life-Years for 29 Cancer Groups, 1990 to 2016: A Systematic Analysis for the Global Burden of Disease Study. *JAMA Oncol*. 2018;4(11):1553-1568. doi:10.1001/jamaoncol.2018.2706

27. Asadzadeh Vostakolaei F, Karim-Kos HE, Janssen-Heijnen MLG, Visser O, Verbeek ALM, Kiemeney LALM. The validity of the mortality to incidence ratio as a proxy for site-specific cancer survival. *Eur J Public Health*. 2011;21(5):573-577. doi:10.1093/eurpub/ckq120

28. *SEER*Stat Software*.; 2014. http://seer.cancer.gov/seerstat/

29. Sankaranarayanan R, Swaminathan R, Lucas E. *Cancer Survival in Africa, Asia, the Caribbean and Central America (SurvCan)*.

30. National Center for Health Statistics, Centers for Disease Control and, Prevention. US Mortality Files. 61-Year Trends in U.S. Cancer Death Rates. http://seer.cancer.gov/archive/csr/1975_2010/results_merged/topic_historical_mort_trends.pdf

31. SEER Cancer Statistics Review 1975-2011. http://seer.cancer.gov/csr/1975_2011/results_merged/topic_survival_by_year_dx.pdf

32. Fitzmaurice C, Dicker D, Pain A, et al. The Global Burden of Cancer 2013. *JAMA Oncol*. 2015;1(4):505-527. doi:10.1001/jamaoncol.2015.0735

33. Neal RD, Din NU, Hamilton W, et al. Comparison of cancer diagnostic intervals before and after implementation of NICE guidelines: analysis of data from the UK General Practice Research Database. *Br J Cancer*. 2014;110(3):584-592. doi:10.1038/bjc.2013.791

34. *Surveillance, Epidemiology, and End Results (SEER) Program (Www.Seer.Cancer.Gov) SEER*Stat Database: Incidence - SEER 18 Regs Research Data + Hurricane Katrina Impacted Louisiana Cases, Nov 2012 Sub (1973-2010 Varying) - Linked To County Attributes - Total U.S., 1969-2011 Counties, National Cancer Institute, DCCPS, Surveillance Research Program, Surveillance Systems Branch, Released April 2013, Based on the November 2012 Submission.*

35. Allgar VL, Neal RD. Delays in the diagnosis of six cancers: analysis of data from the National Survey of NHS Patients: Cancer. *Br J Cancer*. 2005;92(11):1959-1970. doi:10.1038/sj.bjc.6602587

36. Neal RD, Cannings-John R, Hood K, et al. Excision of malignant melanomas in North Wales: effect of location and surgeon on time to diagnosis and quality of excision. *Fam Pract*. 2008;25(4):221-227. doi:10.1093/fampra/cmn036

37. Kewalramani T, Nimer SD, Zelenetz AD, et al. Progressive disease following autologous transplantation in patients with chemosensitive relapsed or primary refractory Hodgkin's disease or aggressive non-Hodgkin's lymphoma. *Bone Marrow Transplant*. 2003;32(7):673-679. doi:10.1038/sj.bmt.1704214

38. Esteban D, Tovar N, Jiménez R, et al. Patients with relapsed/refractory chronic lymphocytic leukaemia may benefit from inclusion in clinical trials irrespective of the therapy received: a case-control retrospective analsysis. *Blood Cancer J*. 2015;5:e356. doi:10.1038/bcj.2015.78

39. Canova C, Giorato E, Roveron G, Turrini P, Zanotti R. Validation of a stoma-specific quality of life questionnaire in a sample of patients with colostomy or ileostomy. *Colorectal Dis Off J Assoc Coloproctology G B Irel*. 2013;15(11):e692-698. doi:10.1111/codi.12324

40. Caricato M, Ausania F, Ripetti V, Bartolozzi F, Campoli G, Coppola R. Retrospective analysis of long-term defunctioning stoma complications after colorectal surgery. *Colorectal Dis Off J Assoc Coloproctology G B Irel*. 2007;9(6):559-561. doi:10.1111/j.1463-1318.2006.01187.x

41. Erwin-Toth P, Thompson SJ, Davis JS. Factors impacting the quality of life of people with an ostomy in North America: results from the Dialogue Study. *J Wound Ostomy Cont Nurs Off Publ Wound Ostomy Cont Nurses Soc WOCN*. 2012;39(4):417-422; quiz 423-424. doi:10.1097/WON.0b013e318259c441

42. Catalona WJ, Carvalhal GF, Mager DE, Smith DS. Potency, continence and complication rates in 1,870 consecutive radical retropubic prostatectomies. *J Urol*. 1999;162(2):433-438.

43. Donnellan SM, Duncan HJ, MacGregor RJ, Russell JM. Prospective assessment of incontinence after radical retropubic prostatectomy: objective and subjective analysis. *Urology*. 1997;49(2):225-230. doi:10.1016/S0090-4295(96)00451-7

44. Eastham JA, Kattan MW, Rogers E, et al. Risk factors for urinary incontinence after radical prostatectomy. *J Urol*. 1996;156(5):1707-1713.

45. Kundu SD, Roehl KA, Eggener SE, Antenor JAV, Han M, Catalona WJ. Potency, continence and complications in 3,477 consecutive radical retropubic prostatectomies. *J Urol*. 2004;172(6 Pt 1):2227-2231.

46. Potosky AL, Davis WW, Hoffman RM, et al. Five-Year Outcomes After Prostatectomy or Radiotherapy for Prostate Cancer: The Prostate Cancer Outcomes Study. *JNCI J Natl Cancer Inst*. 2004;96(18):1358-1367. doi:10.1093/jnci/djh259

47. Sacco E, Prayer-Galetti T, Pinto F, et al. Urinary incontinence after radical prostatectomy: incidence by definition, risk factors and temporal trend in a large series with a long-term follow-up. *BJU Int*. 2006;97(6):1234-1241. doi:10.1111/j.1464-410X.2006.06185.x

48. Stanford JL, Feng Z, Hamilton AS, et al. Urinary and sexual function after radical prostatectomy for clinically localized prostate cancer: the Prostate Cancer Outcomes Study. *JAMA*. 2000;283(3):354-360.

49. Walsh PC, Marschke P, Ricker D, Burnett AL. Patient-reported urinary continence and sexual function after anatomic radical prostatectomy. *Urology*. 2000;55(1):58-61.

50. Ferlay J, Ervik M, Lam F, et al. Global Cancer Observatory: Cancer Today. Lyon, France: International Agency for Research on Cancer. Published 2020. Accessed March 10, 2021. https://gco.iarc.fr/today

51. Ferlay J, Colombet M, Soerjomataram I, et al. Estimating the global cancer incidence and mortality in 2018: GLOBOCAN sources and methods. *Int J Cancer*. 2019;144(8):1941-1953. doi:https://doi.org/10.1002/ijc.31937

52. Truven Health Analytics. United States MarketScan Commercial Claims and Encounters Database. Ann Arbor, United States.

53. Karagas MR, Greenberg ER, Spencer SK, Stukel TA, Mott LA. Increase in incidence rates of basal cell and squamous cell skin cancer in New Hampshire, USA. New Hampshire Skin Cancer Study Group. *Int J Cancer J Int Cancer*. 1999;81(4):555-559.

54. Medical Expenditure Panel Survey Home. Accessed November 15, 2019. https://meps.ahrq.gov/mepsweb/

Authors' Contributions

Managing the estimation or publications process

Jonathan M Kocarnik, Kelly Compton, Christina Fitzmaurice, and Lisa M Force.

Writing the first draft of the manuscript

Jonathan M Kocarnik, Kelly Compton, and Lisa M Force.

Primary responsibility for applying analytical methods to produce estimates

Jonathan M Kocarnik, Frances E Dean, Weijia Fu, James D Harvey, Rixing Xu, and Lisa M Force.

Primary responsibility for seeking, cataloguing, extracting, or cleaning data; designing or coding figures and tables

Jonathan M Kocarnik, Frances E Dean, Weijia Fu, Brian L Gaw, James D Harvey, Hannah Jacqueline Henrikson, Dan Lu, Alyssa Pennini, Rixing Xu, Lisa M Force.

Providing data or critical feedback on data sources

Jonathan M Kocarnik, Hedayat Abbastabar, Amir Abdoli, Hassan Abidi, Hassan Abolhassani, Isaac Akinkunmi Adedeji, Qorinah Estiningtyas Sakilah Adnani, Shailesh M Advani, Muhammad Sohail Afzal, Mohammad Aghaali, Bright Opoku Ahinkorah, Sajjad Ahmad Ahmad, Tauseef Ahmad, Ali Ahmadi, Sepideh Ahmadi, Tarik Ahmed Rashid, Yusra Ahmed Salih, Gizachew Taddesse Akalu, Addis Aklilu, Chisom Joyqueenet Akunna, Hanadi Al Hamad, Fares Alahdab, Saqib Ali, Syed Mohamed Aljunid, Motasem Alkhayyat, Amir Almasi-Hashiani, Sami Almustanyir, Nelson Alvis-Guzman, Hubert Amu, Fereshteh Ansari, Maxwell Hubert Antwi, Davood Anvari, Jalal Arabloo, Morteza Arab-Zozani, Olatunde Aremu, Hany Ariffin, Timur Aripov, Al Artaman, Judie Arulappan, Avinash Aujayeb, Marcel Ausloos, Beatriz Paulina Ayala Quintanilla, Mohammed A Mohammed Azab, Amirhossein Azari Jafari, Ahmed Y Azzam, Saeed Bahadory, Atif Amin Baig, Senthilkumar Balakrishnan, Maciej Banach, Till Winfried Bärnighausen, Amadou Barrow, Devidas S Bhagat, Sonu Bhaskar, Vijayalakshmi S Bhojaraja, Sadia Bibi, Archie Bleyer, Obasanjo Afolabi Bolarinwa, Dejana Braithwaite, Florentino Luciano Caetano dos Santos, Ferrán Catalá-López, Achille Cernigliaro, Raja Chandra Chakinala, Soosanna Kumary Chattu, Vijay Kumar Chattu, Dinh-Toi Chu, Michael T Chung, Joao Conde, Paolo Angelo Cortesi, Vera Marisa Costa, Saad M A Dahlawi, Xiaochen Dai, Lalit Dandona, Rakhi Dandona, Aso Mohammad Darwesh, Fernando Pio De la Hoz, Mandira Lamichhane Dhimal, Meghnath Dhimal, Mostafa Dianatinasab, Huyen Phuc Do, Saeid Doaei, Tim Robert Driscoll, Sahar Eftekharzadeh, Arash Etemadi, Mohammed Faisaluddin, Jawad Fares, Farshad Farzadfar, Takeshi Fukumoto, Peter Andras Gaal, Mohamed M Gad, Muktar A Gadanya, Ahmad Ghashghaee, Syed Amir Gilani, Themba G Ginindza, Abraham Tamirat T Gizaw, Mahaveer Golechha, Pouya Goleij, Ricardo Santiago Gomez, Houman Goudarzi, Giuseppe Grosso, Avirup Guha, D Sanjeeva Gunasekera, Bhawna Gupta, Veer Bala Gupta, Vivek Kumar Gupta, Nima Hafezi-Nejad, Arvin Haj-Mirzaian, Rabih Halwani, Shafiul Haque, Josep Maria Haro, Ahmed I Hasaballah, Soheil Hassanipour, Roderick J Hay, Mohammad Heidari, Claudiu Herteliu, Mehdi Hosseinzadeh, Mowafa Househ, Segun Emmanuel Ibitoye, Sheikh Mohammed Shariful Islam, Nahlah Elkudssiah Ismail, Gaetano Isola, Vardhmaan Jain, Mihajlo Jakovljevic, Tahereh Javaheri, Shubha Jayaram, Seyed Behzad Jazayeri, Jost B Jonas, Tamas Joo, Nitin Joseph, Farahnaz Joukar, Mikk Jürisson, Danial Kahrizi, Leila R Kalankesh, Rohollah Kalhor, Feroze Kaliyadan, Nawzad Kameran Al-Salihi, André Karch, Ayele Semachew Kasa, Srinivasa Vittal Katikireddi, Joonas H Kauppila, Taras Kavetskyy, Yousef Saleh Khader, Rovshan Khalilov, Maseer Khan, Md Nuruzzaman Khan, Young-Ho Khang, Amir M Khater, Gyu Ri Kim, Yun Jin Kim, Adnan Kisa, Sezer Kisa, Parvaiz A Koul, Sindhura Lakshmi Koulmane Laxminarayana, Burcu Kucuk Bicer, Nuworza Kugbey, G Anil Kumar, Om P Kurmi, Paolo Lauriola, Sang-woong Lee, Shaun Wen Huey Lee, Yo Han Lee,

James Leigh, Elvynna Leong, Xuefeng Liu, Muhammed Magdy Abd El Razek, Alaa Makki, Mohammad Ali Mansournia, Walter Mendoza, Ritesh G Menezes, Endalkachew Worku Mengesha, Bartosz Miazgowski, Irmina Maria Michalek, Ted R Miller, Prasanna Mithra, Masoud Moghadaszadeh, Karzan Abdulmuhsin Mohammad, Mokhtar Mohammadi, Abdollah Mohammadian-Hafshejani, Shafiu Mohammed, Ali H Mokdad, Mariam Molokhia, Lorenzo Monasta, Sumaira Mubarik, Ahamarshan Jayaraman Nagarajan, Mukhammad David Naimzada, Sreenivas Narasimha Swamy, Ionut Negoi, Cuong Tat Nguyen, Huong Lan Thi Nguyen, Robina Khan Niazi, Chukwudi A Nnaji, Chimezie Igwegbe Nzoputam, Bogdan Oancea, Felix Akpojene Ogbo, Andrew T Olagunju, Emad Omar, Ahmed Omar Bali, Abidemi E Emmanuel Omonisi, Sokking Ong, Obinna E Onwujekwe, Hans Orru, Doris V Ortega-Altamirano, Nikita Otstavnov, Stanislav S Otstavnov, Mayowa O Owolabi, Mahesh P A, Jagadish Rao Padubidri, Keyvan Pakshir, Adrian Pana, Demosthenes Panagiotakos, Songhomitra Panda-Jonas, Shahina Pardhan, Eun-Cheol Park, Fatemeh Pashazadeh Kan, Harsh K Patel, Jenil R Patel, Sanjay M Pattanshetty, David M Pereira, Renato B Pereira Pereira, Arokiasamy Perianayagam, Julian David Pillay, Maarten J Postma, Hadi Pourjafar, Liliana Preotescu, Mohammad Rabiee, Navid Rabiee, Raghu Anekal Radhakrishnan, Fakher Rahim, Amir Masoud Rahmani, Ivo Rakovac, Kiana Ramezanzadeh, Chythra R Rao, Sowmya J Rao, Reza Rawassizadeh, Andre M N Renzaho, Nima Rezaei, Jefferson Antonio Buendia Rodriguez, Luca Ronfani, Gholamreza Roshandel, Godfrey M Rwegerera, Manjula S, Siamak Sabour, Basema Saddik, Umar Saeed, Abdallah M Samy, Juan Sanabria, Senthilkumar Sankararaman, Milena M Santric-Milicevic, Brijesh Sathian, Monika Sawhney, Mete Saylan, Mario Sekerija, Allen Seylani, Masood Ali Shaikh, Mohammed Shannawaz, Sara Sheikhbahaei, Adithi Shetty, Jeevan K Shetty, Pavanchand H Shetty, K M Shivakumar, Soraya Siabani, Jasvinder A Singh, Valentin Yurievich Skryabin, Anna Aleksandrovna Skryabina, Matthew J Soeberg, Houman Sotoudeh, Daniel Sur, Viktória Szerencsés, Miklós Szócska, Rafael Tabarés-Seisdedos, Takahiro Tabuchi, Amir Taherkhani, Ker-Kan Tan, Mohamad-Hani Temsah, Gizachew Assefa Tessema, Viveksandeep Thoguluva Chandrasekar, Nihal Thomas, Mathilde Touvier, Marcos Roberto Tovani-Palone, Bach Xuan Tran, Irfan Ullah, Saif Ullah, Krishna Kishore Umapathi, Bhaskaran Unnikrishnan, Sahel Valadan Tahbaz, Vasily Vlassov, Bay Vo, Victor Volovici, Giang Thu Vu, Yasir Waheed, Ronny Westerman, Seyed Hossein Yahyazadeh Jabbari, Sanni Yaya, Naohiro Yonemoto, Mustafa Z Younis, Chuanhua Yu, Deniz Yuce, Ismaeel Yunusa, Vesna Zadnik, Mikhail Sergeevich Zastrozhin, Anasthasia Zastrozhina, Arash Ziapour, and Christopher J L Murray.

Developing methods or computational machinery

Jonathan M Kocarnik, Frances E Dean, Weijia Fu, Alyssa Pennini, Rixing Xu, Ali Ahmadi, Tarik Ahmed Rashid, Yusra Ahmed Salih, Tayyaba Akram, Saqib Ali, Amir Almasi-Hashiani, Hubert Amu, Davood Anvari, Avinash Aujayeb, Mohammed A Mohammed Azab, Ahmed Y Azzam, Devasahayam J Christopher, Xiaochen Dai, Aso Mohammad Darwesh, Mostafa Dianatinasab, Saeid Doaei, Firooz Esmaeilzadeh, Mahaveer Golechha, Bhawna Gupta, Mohammad Rifat Haider, Mohammad Heidari, Mehdi Hosseinzadeh, Mowafa Househ, Rabia Hussain, Tahereh Javaheri, Danial Kahrizi, Nawzad Kameran Al-Salihi, Taras Kavetskyy, Rovshan Khalilov, Adnan Kisa, Sezer Kisa, Sang-woong Lee, James Leigh, Alaa Makki, Masoud Moghadaszadeh, Mokhtar Mohammadi, Shafiu Mohammed, Ali H Mokdad, Abbas Mosapour, Emad Omar, Ahmed Omar Bali, Saeed Pirouzpanah, Amir Masoud Rahmani, Reza Rawassizadeh, Umar Saeed, Abdallah M Samy, Bay Vo, Ronny Westerman, Arash Ziapour, and Christopher J L Murray.

Providing critical feedback on methods or results

Jonathan M Kocarnik, Frances E Dean, Emad Ababneh, Hedayat Abbastabar, Sherief M Abd-Elsalam, Aidin Abedi, Hassan Abidi, Hassan Abolhassani, Isaac Akinkunmi Adedeji, Qorinah Estiningtyas Sakilah

Adnani, Shailesh M Advani, Muhammad Sohail Afzal, Mohammad Aghaali, Bright Opoku Ahinkorah, Sajjad Ahmad Ahmad, Tauseef Ahmad, Ali Ahmadi, Tarik Ahmed Rashid, Yusra Ahmed Salih, Gizachew Taddesse Akalu, Addis Aklilu, Tayyaba Akram, Chisom Joyqueenet Akunna, Hanadi Al Hamad, Fares Alahdab, Ziyad Al-Aly, Saqib Ali, Yousef Alimohamadi, Vahid Alipour, Syed Mohamed Aljunid, Motasem Alkhayyat, Amir Almasi-Hashiani, Nihad A Almasri, Sami Almustanyir, Nivaldo Alonso, Nelson Alvis-Guzman, Hubert Amu, Etsay Woldu Anbesu, Robert Ancuceanu, Fereshteh Ansari, Alireza Ansari-Moghaddam, Maxwell Hubert Antwi, Davood Anvari, Anayochukwu Edward Anyasodor, Muhammad Aqeel, Jalal Arabloo, Morteza Arab-Zozani, Olatunde Aremu, Hany Ariffin, Timur Aripov, Muhammad Arshad, Al Artaman, Judie Arulappan, Zatollah Asemi, Mohammad Asghari Jafarabadi, Tahira Ashraf, Prince Atorkey, Avinash Aujayeb, Marcel Ausloos, Atalel Fentahun Awedew, Beatriz Paulina Ayala Quintanilla, Temesgen Ayenew, Mohammed A Mohammed Azab, Amirhossein Azari Jafari, Ghasem Azarian, Ahmed Y Azzam, Ashish D Badiye, Atif Amin Baig, Jennifer L Baker, Senthilkumar Balakrishnan, Maciej Banach, Till Winfried Bärnighausen, Fabio Barra, Amadou Barrow, Masoud Behzadifar, Woldesellassie M Mequanint Bezabhe, Yihienew Mequanint Bezabih, Akshaya Srikanth Bhagavathula, Nikha Bhardwaj, Pankaj Bhardwaj, Sonu Bhaskar, Krittika Bhattacharyya, Vijayalakshmi S Bhojaraja, Sadia Bibi, Ali Bijani, Antonio Biondi, Archie Bleyer, Oleg Blyuss, Obasanjo Afolabi Bolarinwa, Srinivasa Rao Bolla, Dejana Braithwaite, Hermann Brenner, Nadeem Shafique Butt, Zahid A Butt, Florentino Luciano Caetano dos Santos, Yin Cao, Giulia Carreras, Ferrán Catalá-López, Francieli Cembranel, Ester Cerin, Achille Cernigliaro, Raja Chandra Chakinala, Soosanna Kumary Chattu, Vijay Kumar Chattu, Odgerel Chimed-Ochir, Daniel Youngwhan Cho, Devasahayam J Christopher, Dinh-Toi Chu, Michael T Chung, Joao Conde, Paolo Angelo Cortesi, Vera Marisa Costa, Omid Dadras, Amare Belachew Dagnew, Saad M A Dahlawi, Xiaochen Dai, Lalit Dandona, Rakhi Dandona, Aso Mohammad Darwesh, José das Neves, Fernando Pio De la Hoz, Asmamaw Bizuneh Demis, Deepak Dhamnetiya, Mandira Lamichhane Dhimal, Meghnath Dhimal, Mostafa Dianatinasab, Daniel Diaz, Shirin Djalalinia, Huyen Phuc Do, Saeid Doaei, Fariba Dorostkar, Francisco Winter dos Santos Figueiredo, Tim Robert Driscoll, Hedyeh Ebrahimi, Hassan El-Abid, Hala Rashad Elhabashy, Muhammed Elhadi, Babak Eshrati, Sharareh Eskandarieh, Firooz Esmaeilzadeh, Arash Etemadi, Mohammed Faisaluddin, Emerito Jose A Faraon, Jawad Fares, Farshad Farzadfar, Abdullah Hamid Feroze, Simone Ferrero, Lorenzo Ferro Desideri, Irina Filip, Florian Fischer, James L Fisher, Masoud Foroutan, Takeshi Fukumoto, Peter Andras Gaal, Mohamed M Gad, Muktar A Gadanya, Mariana Gaspar Fonseca, Mansour Ghafourifard, Nermin Ghith, Themba G Ginindza, Abraham Tamirat T Gizaw, James C Glasbey, Mahaveer Golechha, Ricardo Santiago Gomez, Sameer Vali Gopalani, Houman Goudarzi, Giuseppe Grosso, Mohammed Ibrahim Mohialdeen Gubari, Avirup Guha, D Sanjeeva Gunasekera, Bhawna Gupta, Veer Bala Gupta, Vivek Kumar Gupta, Reyna Alma Gutiérrez, Nima Hafezi-Nejad, Mohammad Rifat Haider, Rabih Halwani, Randah R Hamadeh, Sajid Hameed, Samer Hamidi, Asif Hanif, Shafiul Haque, Netanja I Harlianto, Josep Maria Haro, Ahmed I Hasaballah, Soheil Hassanipour, Roderick J Hay, Simon I Hay, Khezar Hayat, Golnaz Heidari, Mohammad Heidari, Brenda Yuliana Herrera-Serna, Claudiu Herteliu, Kamal Hezam, Ramesh Holla, Md Mahbub Hossain, Mohammad-Salar Hosseini, Mehdi Hosseinzadeh, Mihaela Hostiuc, Mowafa Househ, Mohamed Hsairi, Fernando N Hugo, Rabia Hussain, Nawfal R Hussein, Bing-Fang Hwang, Segun Emmanuel Ibitoye, Fidelia Ida, Olayinka Stephen Ilesanmi, Irena M Ilic, Milena D Ilic, Lalu Muhammad Irham, Jessica Y Islam, Sheikh Mohammed Shariful Islam, Nahlah Elkudssiah Ismail, Gaetano Isola, Masao Iwagami, Louis Jacob, Vardhmaan Jain, Mihajlo Jakovljevic, Tahereh Javaheri, Shubha Jayaram, Seyed Behzad Jazayeri, Ravi Prakash Jha, Jost B Jonas, Tamas Joo, Nitin Joseph, Farahnaz Joukar, Mikk Jürisson, Ali Kabir, Danial Kahrizi, Leila R Kalankesh, Rohollah Kalhor, Feroze Kaliyadan, Yogeshwar Kalkonde, Nawzad Kameran Al-Salihi, Himal Kandel, Neeti Kapoor, André Karch, Srinivasa Vittal Katikireddi, Joonas H Kauppila, Taras Kavetskyy, Sewnet Adem Kebede, Pedram Keshavarz, Yousef Saleh Khader, Rovshan Khalilov, Gulfaraz Khan, Maseer Khan, Md

Nuruzzaman Khan, Moien AB Khan, Young-Ho Khang, Amir M Khater, Maryam Khayamzadeh, Gyu Ri Kim, Yun Jin Kim, Adnan Kisa, Sezer Kisa, Katarzyna Kissimova-Skarbek, Jacek A Kopec, Rajasekaran Koteeswaran, Sindhura Lakshmi Koulmane Laxminarayana, Ai Koyanagi, Burcu Kucuk Bicer, Nuworza Kugbey, G Anil Kumar, Narinder Kumar, Nithin Kumar, Om P Kurmi, Tezer Kutluk, Carlo La Vecchia, Faris Hasan Lami, Paolo Lauriola, Sang-woong Lee, Shaun Wen Huey Lee, Wei-Chen Lee, Yo Han Lee, James Leigh, Elvynna Leong, Jiarui Li, Ming-Chieh Li, Xuefeng Liu, Joana A Loureiro, Muhammed Magdy Abd El Razek, Azeem Majeed, Alaa Makki, Ahmad Azam Malik, Mohammad Ali Mansournia, Santi Martini, Seyedeh Zahra Masoumi, Prashant Mathur, Martin McKee, Walter Mendoza, Ritesh G Menezes, Endalkachew Worku Mengesha, Mohamed Kamal Mesregah, Tomislav Mestrovic, Junmei Miao Jonasson, Tomasz Miazgowski, Irmina Maria Michalek, Ted R Miller, Hamed Mirzaei, Hamid Reza Mirzaei, Sanjeev Misra, Prasanna Mithra, Masoud Moghadaszadeh, Yousef Mohammad, Mokhtar Mohammadi, Seyyede Momeneh Mohammadi, Abdollah Mohammadian-Hafshejani, Shafiu Mohammed, Nagabhishek Moka, Ali H Mokdad, Mariam Molokhia, Mohammad Ali Moni, Mohammad Amin Moosavi, Yousef Moradi, Joana Morgado-da-Costa, Sumaira Mubarik, Ahamarshan Jayaraman Nagarajan, Shankar Prasad Nagaraju, Mukhammad David Naimzada, Vinay Nangia, Atta Abbas Naqvi, Sreenivas Narasimha Swamy, Rawlance Ndejjo, Ionut Negoi, Serban Mircea Negru, Sandhya Neupane Kandel, Cuong Tat Nguyen, Huong Lan Thi Nguyen, Robina Khan Niazi, Chukwudi A Nnaji, Nurulamin M Noor, Chimezie Igwegbe Nzoputam, Bogdan Oancea, Chimedsuren Ochir, Oluwakemi Ololade Odukoya, Felix Akpojene Ogbo, Andrew T Olagunju, Babayemi Oluwaseun Olakunde, Emad Omar, Ahmed Omar Bali, Abidemi E Emmanuel Omonisi, Sokking Ong, Obinna E Onwujekwe, Hans Orru, Doris V Ortega-Altamirano, Nikita Otstavnov, Stanislav S Otstavnov, Mayowa O Owolabi, Mahesh P A, Jagadish Rao Padubidri, Adrian Pana, Demosthenes Panagiotakos, Songhomitra Panda-Jonas, Shahina Pardhan, Eun-Cheol Park, Eun-Kee Park, Harsh K Patel, Jenil R Patel, Siddhartha Pati, Sanjay M Pattanshetty, Uttam Paudel, David M Pereira, Renato B Pereira Pereira, Arokiasamy Perianayagam, Julian David Pillay, Saeed Pirouzpanah, Indrashis Podder, Maarten J Postma, Hadi Pourjafar, Akila Prashant, Liliana Preotescu, Mohammad Rabiee, Navid Rabiee, Amir Radfar, Venkatraman Radhakrishnan, Ata Rafiee, Fakher Rahim, Shadi Rahimzadeh, Mosiur Rahman, Muhammad Aziz Rahman, Amir Masoud Rahmani, Nazanin Rajai, Aashish Rajesh, Ivo Rakovac, Pradhum Ram, Kamal Ranabhat, Priyanga Ranasinghe, Chythra R Rao, Sowmya J Rao, Reza Rawassizadeh, Mohammad Sadegh Razeghinia, Andre M N Renzaho, Negar Rezaei, Nima Rezaei, Aziz Rezapour, Thomas J Roberts, Jefferson Antonio Buendia Rodriguez, Peter Rohloff, Gholamreza Roshandel, Godfrey M Rwegerera, Manjula S, Siamak Sabour, Basema Saddik, Umar Saeed, Harihar Sahoo, Sana Salehi, Marwa Rashad Salem, Hamideh Salimzadeh, Mehrnoosh Samaei, Abdallah M Samy, Juan Sanabria, Senthilkumar Sankararaman, Milena M Santric-Milicevic, Yaeesh Sardiwalla, Arash Sarveazad, Brijesh Sathian, Monika Sawhney, Mete Saylan, Ione Jayce Ceola Schneider, Mario Sekerija, Allen Seylani, Omid Shafaat, Zahra Shaghaghi, Masood Ali Shaikh, Erfan Shamsoddin, Mohammed Shannawaz, Rajesh Sharma, Aziz Sheikh, Sara Sheikhbahaei, Jeevan K Shetty, Pavanchand H Shetty, Kenji Shibuya, K M Shivakumar, Velizar Shivarov, Soraya Siabani, Diego Augusto Santos Silva, Jasvinder A Singh, Yitagesu Sintayehu, Valentin Yurievich Skryabin, Anna Aleksandrovna Skryabina, Ahmad Sofi-Mahmudi, Houman Sotoudeh, Paschalis Steiropoulos, Kurt Straif, Ranjeeta Subedi, Mu'awiyyah Babale Sufiyan, Iyad Sultan, Saima Sultana, Daniel Sur, Viktória Szerencsés, Miklós Szócska, Rafael Tabarés-Seisdedos, Hooman Tadbiri, Ken Takahashi, Iman M Talaat, Ker-Kan Tan, Vivian Y Tat, Bemnet Amare A Tedla, Yonas Getaye Tefera, Arash Tehrani-Banihashemi, Fisaha Haile Tesfay, Gizachew Assefa Tessema, Rekha Thapar, Aravind Thavamani, Viveksandeep Thoguluva Chandrasekar, Nihal Thomas, Hamid Reza Tohidinik, Mathilde Touvier, Marcos Roberto Tovani-Palone, Eugenio Traini, Bach Xuan Tran, Khanh Bao Tran, Mai Thi Ngoc Tran, Jaya Prasad Tripathy, Biruk Shalmeno Tusa, Irfan Ullah, Saif Ullah, Krishna Kishore Umapathi, Bhaskaran Unnikrishnan, Era Upadhyay, Marco Vacante, Maryam

Vaezi, Sahel Valadan Tahbaz, Diana Zuleika Velazquez, Francesco S Violante, Vasily Vlassov, Bay Vo, Victor Volovici, Giang Thu Vu, Yasir Waheed, Richard G Wamai, Paul Ward, Yi Feng Wen, Ronny Westerman, Andrea Sylvia Winkler, Seyed Hossein Yahyazadeh Jabbari, Lin Yang, Sanni Yaya, Taklo Simeneh Yazie Yazie, Yigizie Yeshaw, Naohiro Yonemoto, Mustafa Z Younis, Zabihollah Yousefi, Chuanhua Yu, Deniz Yuce, Ismaeel Yunusa, Vesna Zadnik, Fariba Zare, Mikhail Sergeevich Zastrozhin, Anasthasia Zastrozhina, Jianrong Zhang, Chenwen Zhong, Linghui Zhou, Cong Zhu, Arash Ziapour, Ivan R Zimmermann, and Christopher J L Murray.

Drafting the work or revising is critically for important intellectual content

Jonathan M Kocarnik, Kelly Compton, Weijia Fu, Mohsen Abbasi-Kangevari, Hedayat Abbastabar, Sherief M Abd-Elsalam, Hassan Abidi, Hassan Abolhassani, Isaac Akinkunmi Adedeji, Shailesh M Advani, Muhammad Sohail Afzal, Mohammad Aghaali, Bright Opoku Ahinkorah, Sajjad Ahmad Ahmad, Ali Ahmadi, Sepideh Ahmadi, Gizachew Taddesse Akalu, Tayyaba Akram, Fares Alahdab, Motasem Alkhayyat, Amir Almasi-Hashiani, Nihad A Almasri, Sadeq Ali Ali Al-Maweri, Sami Almustanyir, Nivaldo Alonso, Nelson Alvis-Guzman, Hubert Amu, Robert Ancuceanu, Maxwell Hubert Antwi, Anayochukwu Edward Anyasodor, Jalal Arabloo, Morteza Arab-Zozani, Olatunde Aremu, Hany Ariffin, Timur Aripov, Judie Arulappan, Prince Atorkey, Avinash Aujayeb, Marcel Ausloos, Beatriz Paulina Ayala Quintanilla, Mohammed A Mohammed Azab, Sina Azadnajafabad, Amirhossein Azari Jafari, Ahmed Y Azzam, Atif Amin Baig, Jennifer L Baker, Senthilkumar Balakrishnan, Maciej Banach, Till Winfried Bärnighausen, Francesco Barone-Adesi, Fabio Barra, Amadou Barrow, Masoud Behzadifar, Uzma Iqbal Belgaumi, Woldesellassie M Mequanint Bezabhe, Yihienew Mequanint Bezabih, Akshaya Srikanth Bhagavathula, Sonu Bhaskar, Krittika Bhattacharyya, Vijayalakshmi S Bhojaraja, Sadia Bibi, Antonio Biondi, Catherine Bisignano, Tone Bjørge, Dejana Braithwaite, Amanpreet Brar, Hermann Brenner, Maria Teresa Bustamante-Teixeira, Nadeem Shafique Butt, Florentino Luciano Caetano dos Santos, Yin Cao, Ferrán Catalá-López, Francieli Cembranel, Ester Cerin, Soosanna Kumary Chattu, Vijay Kumar Chattu, Daniel Youngwhan Cho, Devasahayam J Christopher, Michael T Chung, Joao Conde, Paolo Angelo Cortesi, Vera Marisa Costa, Amanda Ramos Cunha, José das Neves, Fernando Pio De la Hoz, Asmamaw Bizuneh Demis, Edgar Denova-Gutiérrez, Deepak Dhamnetiya, Mandira Lamichhane Dhimal, Meghnath Dhimal, Mostafa Dianatinasab, Daniel Diaz, Huyen Phuc Do, Saeid Doaei, Francisco Winter dos Santos Figueiredo, Tim Robert Driscoll, Sahar Eftekharzadeh, Maha El Tantawi, Iffat Elbarazi, Muhammed Elhadi, Shaimaa I El-Jaafary, Sharareh Eskandarieh, Firooz Esmaeilzadeh, Arash Etemadi, Sayeh Ezzikouri, Mohammed Faisaluddin, Emerito Jose A Faraon, Jawad Fares, Abdullah Hamid Feroze, Simone Ferrero, Lorenzo Ferro Desideri, Irina Filip, Florian Fischer, James L Fisher, Masoud Foroutan, Takeshi Fukumoto, Mohamed M Gad, Muktar A Gadanya, Silvano Gallus, Abera Getachew Obsa, Mansour Ghafourifard, Nermin Ghith, Maryam Gholamalizadeh, Abraham Tamirat T Gizaw, James C Glasbey, Sameer Vali Gopalani, Giuseppe Gorini, Maximiliano Ribeiro Guerra, Avirup Guha, D Sanjeeva Gunasekera, Bhawna Gupta, Veer Bala Gupta, Vivek Kumar Gupta, Reyna Alma Gutiérrez, Nima Hafezi-Nejad, Rabih Halwani, Randah R Hamadeh, Sajid Hameed, Netanja I Harlianto, Josep Maria Haro, Ahmed I Hasaballah, Simon I Hay, Golnaz Heidari, Mohammad Heidari, Claudiu Herteliu, Kamal Hezam, Ramesh Holla, Md Mahbub Hossain, Mohammad Bellal Hossain Hossain, Mohammad-Salar Hosseini, Mostafa Hosseini, Sorin Hostiuc, Mowafa Househ, Junjie Huang, Fernando N Hugo, Rabia Hussain, Nawfal R Hussein, Ivo Iavicoli, Segun Emmanuel Ibitoye, Olayinka Stephen Ilesanmi, Irena M Ilic, Milena D Ilic, Jessica Y Islam, Rakibul M Islam, Sheikh Mohammed Shariful Islam, Nahlah Elkudssiah Ismail, Gaetano Isola, Masao Iwagami, Louis Jacob, Vardhmaan Jain, Mihajlo Jakovljevic, Shubha Jayaram, Ravi Prakash Jha, Jost B Jonas, Nitin Joseph, Mikk Jürisson, Ali Kabir, Danial Kahrizi, Feroze Kaliyadan, Ashwin Kamath, Himal Kandel, André Karch, Srinivasa Vittal Katikireddi, Joonas H Kauppila, Taras Kavetskyy, Sewnet Adem Kebede, Pedram

Keshavarz, Mohammad Keykhaei, Yousef Saleh Khader, Rovshan Khalilov, Maseer Khan, Md Nuruzzaman Khan, Moien AB Khan, Amir M Khater, Gyu Ri Kim, Yun Jin Kim, Adnan Kisa, Sezer Kisa, Parvaiz A Koul, Sindhura Lakshmi Koulmane Laxminarayana, Ai Koyanagi, Nuworza Kugbey, Narinder Kumar, Om P Kurmi, Carlo La Vecchia, Iván Landires, Paolo Lauriola, Elvynna Leong, Joana A Loureiro, Raimundas Lunevicius, Muhammed Magdy Abd El Razek, Azeem Majeed, Shilpa Male, Ahmad Azam Malik, Ravi Mehrotra, Walter Mendoza, Ritesh G Menezes, Endalkachew Worku Mengesha, Mohamed Kamal Mesregah, Tomislav Mestrovic, Tomasz Miazgowski, Irmina Maria Michalek, Ted R Miller, Masoud Moghadaszadeh, Karzan Abdulmuhsin Mohammad, Yousef Mohammad, Shafiu Mohammed, Nagabhishek Moka, Ali H Mokdad, Mariam Molokhia, Lorenzo Monasta, Mohammad Ali Moni, Paula Moraga, Joana Morgado-da-Costa, Shane Douglas Morrison, Abbas Mosapour, Lillian Mwanri, Ahamarshan Jayaraman Nagarajan, Shankar Prasad Nagaraju, Chie Nagata, Mukhammad David Naimzada, Sreenivas Narasimha Swamy, Sabina O Nduaguba, Ionut Negoi, Serban Mircea Negru, Sandhya Neupane Kandel, Cuong Tat Nguyen, Huong Lan Thi Nguyen, Robina Khan Niazi, Nurulamin M Noor, Virginia Nuñez-Samudio, Chimezie Igwegbe Nzoputam, Oluwakemi Ololade Odukoya, Felix Akpojene Ogbo, Andrew T Olagunju, Abidemi E Emmanuel Omonisi, Obinna E Onwujekwe, Hans Orru, Doris V Ortega-Altamirano, Nikita Otstavnov, Stanislav S Otstavnov, Mayowa O Owolabi, Mahesh P A, Jagadish Rao Padubidri, Adrian Pana, Songhomitra Panda-Jonas, Shahina Pardhan, Eun-Cheol Park, Harsh K Patel, Jenil R Patel, Siddhartha Pati, Uttam Paudel, David M Pereira, Renato B Pereira Pereira, Julian David Pillay, Saeed Pirouzpanah, Farhad Pishgar, Indrashis Podder, Maarten J Postma, Liliana Preotescu, Mohammad Rabiee, Navid Rabiee, Amir Radfar, Venkatraman Radhakrishnan, Ata Rafiee, Fakher Rahim, Nazanin Rajai, Ivo Rakovac, Pradhum Ram, Kamal Ranabhat, Priyanga Ranasinghe, Chythra R Rao, Sowmya J Rao, Mohammad Sadegh Razeghinia, Andre M N Renzaho, Nima Rezaei, Thomas J Roberts, Jefferson Antonio Buendia Rodriguez, Peter Rohloff, Michele Romoli, Luca Ronfani, Godfrey M Rwegerera, Manjula S, Basema Saddik, Umar Saeed, Amirhossein Sahebkar, Marwa Rashad Salem, Hamideh Salimzadeh, Mehrnoosh Samaei, Abdallah M Samy, Juan Sanabria, Milena M Santric-Milicevic, Yaeesh Sardiwalla, Mete Saylan, Ione Jayce Ceola Schneider, Mario Sekerija, Allen Seylani, Omid Shafaat, Erfan Shamsoddin, Mohammed Shannawaz, Rajesh Sharma, Aziz Sheikh, Sara Sheikhbahaei, Jeevan K Shetty, Pavanchand H Shetty, Kenji Shibuya, Reza Shirkoohi, K M Shivakumar, Sudeep K Siddappa Malleshappa, Diego Augusto Santos Silva, Jasvinder A Singh, Valentin Yurievich Skryabin, Anna Aleksandrovna Skryabina, Ahmad Sofi-Mahmudi, Paschalis Steiropoulos, Ranjeeta Subedi, Mu'awiyyah Babale Sufiyan, Iyad Sultan, Saima Sultana, Takahiro Tabuchi, Hooman Tadbiri, Iman M Talaat, Ker-Kan Tan, Vivian Y Tat, Yonas Getaye Tefera, Mohamad-Hani Temsah, Gizachew Assefa Tessema, Viveksandeep Thoguluva Chandrasekar, Nihal Thomas, Hamid Reza Tohidinik, Mathilde Touvier, Marcos Roberto Tovani-Palone, Bach Xuan Tran, Jaya Prasad Tripathy, Biruk Shalmeno Tusa, Irfan Ullah, Saif Ullah, Krishna Kishore Umapathi, Bhaskaran Unnikrishnan, Era Upadhyay, Marco Vacante, Sahel Valadan Tahbaz, Diana Zuleika Velazquez, Massimiliano Veroux, Francesco S Violante, Vasily Vlassov, Victor Volovici, Giang Thu Vu, Yasir Waheed, Richard G Wamai, Paul Ward, Ronny Westerman, Andrea Sylvia Winkler, Lalit Yadav, Seyed Hossein Yahyazadeh Jabbari, Lin Yang, Sanni Yaya, Yigizie Yeshaw, Naohiro Yonemoto, Mustafa Z Younis, Deniz Yuce, Mikhail Sergeevich Zastrozhin, Anasthasia Zastrozhina, Jianrong Zhang, Chenwen Zhong, Arash Ziapour, Ivan R Zimmermann, and Christopher J L Murray.

Extracting, cleaning, or cataloging data; designing or coding figures and tables

Jonathan M Kocarnik, Hassan Abidi, Ali Ahmadi, Hubert Amu, Amirhossein Azari Jafari, Mostafa Dianatinasab, Saeid Doaei, Takeshi Fukumoto, Mansour Ghafourifard, Ahmed I Hasaballah, Mohammad Heidari, Mowafa Househ, Danial Kahrizi, Taras Kavetskyy, Rovshan Khalilov, Masoud Moghadaszadeh, Ali H Mokdad, Abdallah M Samy, Saif Ullah, and Arash Ziapour.

Managing the overall research enterprise

Jonathan M Kocarnik, Kelly Compton, Lalit Dandona, Simon I Hay, Ali H Mokdad, and Christopher J L Murray.

Supplementary Tables and Figures

eTable 1: Number of site-years for cancer mortality data by source type, for GBD 2019 compared to GBD 2017

Cause	VR GBD 2017	VR GBD 2019	VR change GBD 2017 to GBD 2019	VA GBD 2017	VA GBD 2019	VA change GBD 2017 to GBD 2019	CR GBD 2017	CR GBD 2019	CR change GBD 2017 to GBD 2019	Total GBD 2017	Total GBD 2019	Total Change GBD 2017 to GBD 2019
All malignant neoplasms (total)	656 430	767 514	16.9	6 900	6 137	-11.1	129 641	155 542	20.0	819 127	929 193	13.4
All malignant neoplasms (unique)	20 120	23 378	16.2	491	541	10.2	5 545	5 385	-2.9	26 156	29 304	12.0
Acute lymphoid leukemia	16 763	19 427	15.9	NA	NA	NA	1 672	2 329	39.3	18 435	21 756	18.0
Acute myeloid leukemia	16 763	19 430	15.9	NA	NA	NA	2 490	3 122	25.4	19 253	22 552	17.1
Bladder cancer	19 320	22 559	16.8	NA	NA	NA	4 279	5 146	20.3	23 599	27 705	17.4
Brain and central nervous system cancer	19 321	22 560	16.8	427	535	25.3	4 478	5 311	18.6	24 226	28 406	17.3
Breast cancer	19 618	23 378	19.2	678	515	-24.0	4 458	5 333	19.6	24 754	29 226	18.1
Cervical cancer	19 618	22 849	16.5	370	182	-50.8	4 378	4 971	13.5	24 366	28 002	14.9
Chronic lymphoid leukemia	16 763	19 105	14.0	NA	NA	NA	1 651	1 062	-35.7	18 414	20 167	9.5
Chronic myeloid leukemia	16 763	19 247	14.8	NA	NA	NA	1 664	2 291	37.7	18 427	21 538	16.9
Colon and rectum cancer	19 618	22 849	16.5	660	516	-21.8	4 474	5 349	19.6	24 752	28 714	16.0
Esophageal cancer	19 614	22 845	16.5	648	514	-20.7	4 453	5 288	18.8	24 715	28 647	15.9
Gallbladder and biliary tract cancer	18 771	22 023	17.3	NA	NA	NA	4 400	5 230	18.9	23 171	27 253	17.6
Hodgkin lymphoma	19 319	22 558	16.8	NA	NA	NA	4 439	5 295	19.3	23 758	27 853	17.2
Kidney cancer	19 318	22 557	16.8	NA	NA	NA	4 342	5 182	19.3	23 660	27 739	17.2
Larynx cancer	19 614	22 845	16.5	374	183	-51.1	4 456	5 292	18.8	24 444	28 320	15.9
Leukemia	19 617	23 377	19.2	584	187	-68.0	4 462	5 319	19.2	24 663	28 883	17.1
Lip and oral cavity cancer	19 322	22 559	16.8	638	514	-19.4	4 315	4 498	4.2	24 275	27 571	13.6
Liver cancer	19 334	22 846	18.2	374	539	44.1	4 464	5 320	19.2	24 172	28 705	18.8
Malignant skin melanoma	19 322	22 559	16.8	NA	NA	NA	4 351	5 266	21.0	23 673	27 825	17.5
Mesothelioma	10 944	13 387	22.3	NA	NA	NA	0	3 362	100.0	10 944	16 749	53.0
Multiple myeloma	19 318	21 929	13.5	NA	NA	NA	4 426	5 227	18.1	23 744	27 156	14.4

Nasopharynx cancer	19 320	22 557	16.8	NA	NA	NA	4 411	5 247	19.0	23 731	27 804	17.2
Non-Hodgkin lymphoma	19 321	22 846	18.2	NA	539	100.0	4 468	5 335	19.4	23 789	28 720	20.7
Non-melanoma skin cancer	19 329	22 557	16.7	NA	NA	NA	NA	NA	NA	19 329	22 557	16.7
Non-melanoma skin cancer (squamous-cell carcinoma)	18 772	22 024	17.3	NA	NA	NA	NA	NA	NA	18 772	22 024	17.3
Other leukemia	16 765	19 776	18.0	210	0	-100.0	3 935	3 096	-21.3	20 910	22 872	9.4
Other malignant neoplasms	19 376	22 848	17.9	NA	540	100.0	3 140	5 368	71.0	22 516	28 756	27.7
Other pharynx cancer	19 320	22 557	16.8	374	186	-50.3	4 404	5 169	17.4	24 098	27 912	15.8
Ovarian cancer	19 318	22 557	16.8	NA	NA	NA	4 455	5 007	12.4	23 773	27 564	15.9
Pancreatic cancer	19 321	22 560	16.8	NA	NA	NA	4 472	5 295	18.4	23 793	27 855	17.1
Prostate cancer	19 615	22 846	16.5	NA	NA	NA	4 455	4 965	11.4	24 070	27 811	15.5
Stomach cancer	19 618	22 849	16.5	374	187	-50.0	4 474	5 312	18.7	24 466	28 348	15.9
Testicular cancer	18 774	21 866	16.5	160	301	88.1	4 425	4 959	12.1	23 359	27 126	16.1
Thyroid cancer	19 319	22 558	16.8	NA	NA	NA	4 457	5 328	19.5	23 776	27 886	17.3
Tracheal, bronchus, and lung cancer	19 618	23 378	19.2	655	516	-21.2	4 459	5 318	19.3	24 732	29 212	18.1
Uterine cancer	19 604	22 846	16.5	374	183	-51.1	4 434	4 950	11.6	24 412	27 979	14.6

Abbreviations: CR, cancer registry; GBD, Global Burden of Disease Study; NA, not applicable; VA, verbal autopsy; VR, vital registration.

Footnote: NA indicates that either no data were available, or that data are not considered reliable for the cancer type. For “All malignant neoplasms,” the “total” row provides the sum of all cancer-specific rows, while the “unique” row is the number of unique site-years of data regardless of cancer type.

eTable 2: Socio-demographic Index groupings by geography, based on 2019 values

Country Name	SDI Quintile
Afghanistan	Low SDI
Albania	Middle SDI
Algeria	Middle SDI
American Samoa	High-middle SDI
Andorra	High SDI
Angola	Low-middle SDI
Antigua and Barbuda	High-middle SDI
Argentina	High-middle SDI
Armenia	Middle SDI
Australia	High SDI
Austria	High SDI
Azerbaijan	Middle SDI
Bahamas	High-middle SDI
Bahrain	High-middle SDI
Bangladesh	Low-middle SDI
Barbados	High-middle SDI
Belarus	High-middle SDI
Belgium	High SDI
Belize	Low-middle SDI
Benin	Low SDI
Bermuda	High SDI
Bhutan	Low-middle SDI
Bolivia (Plurinational State of)	Low-middle SDI
Bosnia and Herzegovina	High-middle SDI
Botswana	Middle SDI
Brazil	Middle SDI
Brunei Darussalam	High SDI
Bulgaria	High-middle SDI
Burkina Faso	Low SDI
Burundi	Low SDI
Cabo Verde	Low-middle SDI
Cambodia	Low-middle SDI
Cameroon	Low-middle SDI
Canada	High SDI
Central African Republic	Low SDI
Chad	Low SDI
Chile	High-middle SDI
China	Middle SDI
Colombia	Middle SDI
Comoros	Low SDI
Congo	Low-middle SDI

Cook Islands	High-middle SDI
Costa Rica	Middle SDI
Croatia	High-middle SDI
Cuba	Middle SDI
Cyprus	High SDI
Czechia	High SDI
Côte d'Ivoire	Low SDI
Democratic People's Republic of Korea	Low-middle SDI
Democratic Republic of the Congo	Low SDI
Denmark	High SDI
Djibouti	Low-middle SDI
Dominica	High-middle SDI
Dominican Republic	Low-middle SDI
Ecuador	Middle SDI
Egypt	Middle SDI
El Salvador	Low-middle SDI
Equatorial Guinea	Middle SDI
Eritrea	Low SDI
Estonia	High SDI
Eswatini	Low-middle SDI
Ethiopia	Low SDI
Fiji	Middle SDI
Finland	High SDI
France	High SDI
Gabon	Middle SDI
Gambia	Low SDI
Georgia	High-middle SDI
Germany	High SDI
Ghana	Low-middle SDI
Greece	High-middle SDI
Greenland	High-middle SDI
Grenada	Middle SDI
Guam	High SDI
Guatemala	Low-middle SDI
Guinea	Low SDI
Guinea-Bissau	Low SDI
Guyana	Middle SDI
Haiti	Low SDI
Honduras	Low-middle SDI
Hungary	High-middle SDI
Iceland	High SDI
India	Low-middle SDI
Indonesia	Middle SDI

Iran (Islamic Republic of)	Middle SDI
Iraq	Middle SDI
Ireland	High SDI
Israel	High-middle SDI
Italy	High-middle SDI
Jamaica	Middle SDI
Japan	High SDI
Jordan	High-middle SDI
Kazakhstan	High-middle SDI
Kenya	Low-middle SDI
Kiribati	Low-middle SDI
Kuwait	High SDI
Kyrgyzstan	Low-middle SDI
Lao People's Democratic Republic	Low-middle SDI
Latvia	High SDI
Lebanon	High-middle SDI
Lesotho	Low-middle SDI
Liberia	Low SDI
Libya	High-middle SDI
Lithuania	High SDI
Luxembourg	High SDI
Madagascar	Low SDI
Malawi	Low SDI
Malaysia	High-middle SDI
Maldives	Low-middle SDI
Mali	Low SDI
Malta	High-middle SDI
Marshall Islands	Low-middle SDI
Mauritania	Low-middle SDI
Mauritius	High-middle SDI
Mexico	Middle SDI
Micronesia (Federated States of)	Low-middle SDI
Monaco	High SDI
Mongolia	Low-middle SDI
Montenegro	High-middle SDI
Morocco	Low-middle SDI
Mozambique	Low SDI
Myanmar	Low-middle SDI
Namibia	Middle SDI
Nauru	Middle SDI
Nepal	Low SDI
Netherlands	High SDI
New Zealand	High SDI

Nicaragua	Low-middle SDI
Niger	Low SDI
Nigeria	Low-middle SDI
Niue	High-middle SDI
North Macedonia	High-middle SDI
Northern Mariana Islands	High-middle SDI
Norway	High SDI
Oman	High-middle SDI
Pakistan	Low SDI
Palau	High-middle SDI
Palestine	Low-middle SDI
Panama	Middle SDI
Papua New Guinea	Low SDI
Paraguay	Middle SDI
Peru	Middle SDI
Philippines	Middle SDI
Poland	High-middle SDI
Portugal	High-middle SDI
Puerto Rico	High SDI
Qatar	High SDI
Republic of Korea	High SDI
Republic of Moldova	High-middle SDI
Romania	High-middle SDI
Russian Federation	High-middle SDI
Rwanda	Low SDI
Saint Kitts and Nevis	High-middle SDI
Saint Lucia	Middle SDI
Saint Vincent and the Grenadines	Middle SDI
Samoa	Middle SDI
San Marino	High SDI
São Tomé and Príncipe	Low-middle SDI
Saudi Arabia	High SDI
Senegal	Low SDI
Serbia	High-middle SDI
Seychelles	High-middle SDI
Sierra Leone	Low SDI
Singapore	High SDI
Slovakia	High SDI
Slovenia	High SDI
Solomon Islands	Low SDI
Somalia	Low SDI
South Africa	Middle SDI
South Sudan	Low SDI

Spain	High-middle SDI
Sri Lanka	High-middle SDI
Sudan	Low-middle SDI
Suriname	Middle SDI
Sweden	High SDI
Switzerland	High SDI
Syrian Arab Republic	Middle SDI
Taiwan (Province of China)	High SDI
Tajikistan	Low-middle SDI
Thailand	Middle SDI
Timor-Leste	Low-middle SDI
Togo	Low SDI
Tokelau	Middle SDI
Tonga	Middle SDI
Trinidad and Tobago	High-middle SDI
Tunisia	Middle SDI
Turkey	High-middle SDI
Turkmenistan	Middle SDI
Tuvalu	Low-middle SDI
Uganda	Low SDI
Ukraine	High-middle SDI
United Arab Emirates	High SDI
United Kingdom	High SDI
United Republic of Tanzania	Low SDI
United States Virgin Islands	High-middle SDI
United States of America	High SDI
Uruguay	High-middle SDI
Uzbekistan	Middle SDI
Vanuatu	Low-middle SDI
Venezuela (Bolivarian Republic of)	Low-middle SDI
Viet Nam	Middle SDI
Yemen	Low SDI
Zambia	Low-middle SDI
Zimbabwe	Low-middle SDI

eTable 3: List of International Classification of Diseases (ICD) codes mapped to the Global Burden of Disease cause list for cancer incidence data

Cause	ICCC3	ICD-10	ICD-9
Lip and oral cavity cancer	XIf1	C00, C00.0, C00.1, C00.2, C00.3, C00.4, C00.5, C00.6, C00.8, C00.9, C01, C01.9, C02, C02.0, C02.1, C02.2, C02.3, C02.4, C02.8, C02.9, C03, C03.0, C03.1, C03.9, C04, C04.0, C04.1, C04.8, C04.9, C05, C05.0, C05.1, C05.2, C05.8, C05.9, C06, C06.0, C06.1, C06.2, C06.8, C06.80, C06.89, C06.9, C07, C07.0, C07.9, C08, C08.0, C08.1, C08.8, C08.9	140, 140.0, 140.1, 140.2, 140.3, 140.4, 140.5, 140.6, 140.7, 140.8, 140.9, 141, 141.0, 141.1, 141.2, 141.3, 141.4, 141.5, 141.6, 141.8, 141.9, 142, 142.0, 142.1, 142.2, 142.3, 142.8, 142.9, 143, 143.0, 143.1, 143.8, 143.9, 144, 144.0, 144.1, 144.4, 144.8, 144.9, 145, 145.0, 145.1, 145.2, 145.3, 145.4, 145.5, 145.6, 145.8, 145.9
Nasopharynx cancer	XIc	C11, C11.0, C11.1, C11.2, C11.3, C11.8, C11.9	147, 147.0, 147.1, 147.2, 147.3, 147.8, 147.9
Other pharynx cancer	NA	C09, C09.0, C09.1, C09.8, C09.9, C1, C10, C10.0, C10.1, C10.2, C10.3, C10.4, C10.8, C10.9, C12, C12.0, C12.9, C13, C13.0, C13.1, C13.2, C13.8, C13.9	146, 146.0, 146.1, 146.2, 146.3, 146.4, 146.5, 146.6, 146.7, 146.8, 146.9, 148, 148.0, 148.1, 148.2, 148.3, 148.4, 148.5, 148.8, 148.9
Esophageal cancer	NA	C15, C15.0, C15.1, C15.2, C15.3, C15.4, C15.5, C15.8, C15.9	150, 150.0, 150.1, 150.2, 150.3, 150.4, 150.5, 150.6, 150.7, 150.8, 150.9
Stomach cancer	NA	C16, C16.0, C16.1, C16.2, C16.3, C16.4, C16.5, C16.6, C16.7, C16.8, C16.9	151, 151.0, 151.1, 151.2, 151.3, 151.4, 151.5, 151.6, 151.8, 151.9, 209.23
Colon and rectum cancer	XIf2, XIf3	C18, C18.0, C18.1, C18.2, C18.3, C18.4, C18.5, C18.6, C18.7, C18.8, C18.9, C19, C19.0, C19.9, C2, C20, C20.0, C20.8, C20.9, C21, C21.0, C21.1, C21.2, C21.8, C21.9	153, 153.0, 153.1, 153.2, 153.3, 153.4, 153.5, 153.6, 153.7, 153.8, 153.9, 154, 154.0, 154.1, 154.2, 154.3, 154.4, 154.8, 154.9, 209.1, 209.10, 209.11, 209.12, 209.13, 209.14, 209.15, 209.16, 209.17, 569.0, 569.43, 569.44, 569.84, 569.85
Liver cancer	VIIb, VIIc	C22, C22.0, C22.1, C22.3, C22.4, C22.5, C22.7, C22.8	155, 155.0, 155.1, 155.3, 155.5, 155.9
Gallbladder and biliary tract cancer	NA	C23, C23.0, C23.9, C24, C24.0, C24.1, C24.4, C24.8, C24.9	156, 156.0, 156.1, 156.2, 156.3, 156.8, 156.9
Pancreatic cancer	XIIa2	C25, C25.0, C25.1, C25.2, C25.3, C25.4, C25.7, C25.8, C25.9	157, 157.0, 157.1, 157.2, 157.3, 157.4, 157.5, 157.7, 157.8, 157.9
Larynx cancer	NA	C32, C32.0, C32.1, C32.2, C32.3, C32.8, C32.9	161, 161.0, 161.1, 161.2, 161.3, 161.8, 161.9
Tracheal, bronchus, and lung cancer	XIIa3, XIf4	C33, C33.0, C33.2, C33.9, C34, C34.0, C34.00, C34.01, C34.02, C34.1, C34.10, C34.11, C34.12, C34.2, C34.3, C34.30, C34.31, C34.32, C34.4, C34.7, C34.8, C34.80, C34.81, C34.82, C34.9, C34.90, C34.91, C34.92	162, 162.0, 162.1, 162.2, 162.3, 162.4, 162.5, 162.8, 162.9, 209.21

Malignant skin melanoma	XId	C43, C43.0, C43.1, C43.10, C43.11, C43.12, C43.2, C43.20, C43.21, C43.22, C43.3, C43.30, C43.31, C43.39, C43.4, C43.5, C43.51, C43.52, C43.59, C43.6, C43.60, C43.61, C43.62, C43.7, C43.70, C43.71, C43.72, C43.8, C43.9	172, 172.0, 172.1, 172.2, 172.3, 172.4, 172.5, 172.6, 172.7, 172.8, 172.9
Non-melanoma skin cancer	XIe	C44.0, C44.00, C44.09, C44.1, C44.10, C44.101, C44.102, C44.109, C44.19, C44.191, C44.192, C44.199, C44.2, C44.20, C44.201, C44.202, C44.209, C44.29, C44.291, C44.292, C44.299, C44.3, C44.30, C44.300, C44.301, C44.309, C44.39, C44.390, C44.391, C44.399, C44.4, C44.40, C44.41, C44.42, C44.49, C44.5, C44.50, C44.500, C44.501, C44.509, C44.590, C44.591, C44.599, C44.6, C44.60, C44.601, C44.602, C44.609, C44.69, C44.691, C44.692, C44.699, C44.7, C44.70, C44.701, C44.702, C44.709, C44.79, C44.791, C44.792, C44.799, C44.8, C44.80, C44.89, C44.9, C44.99	173, 173.0, 173.00, 173.09, 173.1, 173.10, 173.19, 173.2, 173.20, 173.29, 173.3, 173.30, 173.39, 173.4, 173.40, 173.49, 173.5, 173.50, 173.59, 173.6, 173.60, 173.69, 173.7, 173.70, 173.79, 173.8, 173.80, 173.89, 173.9, 173.90, 173.99, 232, 232.0, 232.1, 232.2, 232.3, 232.4, 232.5, 232.6, 232.7, 232.8, 232.9
Breast cancer	XIf6	C50, C50.0, C50.01, C50.011, C50.012, C50.019, C50.02, C50.021, C50.022, C50.029, C50.1, C50.11, C50.111, C50.112, C50.119, C50.12, C50.121, C50.122, C50.129, C50.2, C50.21, C50.211, C50.212, C50.219, C50.22, C50.221, C50.222, C50.229, C50.3, C50.31, C50.311, C50.312, C50.319, C50.32, C50.321, C50.322, C50.329, C50.4, C50.41, C50.411, C50.412, C50.419, C50.42, C50.421, C50.422, C50.429, C50.5, C50.51, C50.511, C50.512, C50.519, C50.52, C50.521, C50.522, C50.529, C50.6, C50.61, C50.611, C50.612, C50.619, C50.62, C50.621, C50.622, C50.629, C50.7, C50.8, C50.81, C50.811, C50.812, C50.819, C50.82, C50.821, C50.822, C50.829, C50.9, C50.91, C50.911, C50.912, C50.919, C50.92, C50.921, C50.922, C50.929	174, 174.0, 174.1, 174.2, 174.3, 174.4, 174.5, 174.6, 174.8, 174.9, 175, 175.0, 175.3, 175.9, 610, 610.0, 610.1, 610.2, 610.3, 610.4, 610.8, 610.9
Cervical cancer	XIf7	C53, C53.0, C53.1, C53.3, C53.4, C53.8, C53.9	180, 180.0, 180.1, 180.2, 180.3, 180.4, 180.5, 180.6, 180.8, 180.9, 622.1, 622.10, 622.11, 622.12, 622.2, 622.7
Uterine cancer	NA	C54, C54.0, C54.1, C54.2, C54.3, C54.4, C54.8, C54.9	182, 182.0, 182.1, 182.8, 182.9
Ovarian cancer	NA	C56, C56.0, C56.1, C56.2, C56.4, C56.9	183, 183.0
Prostate cancer	NA	C61, C61.0, C61.9	185, 185.0, 185.9
Testicular cancer	NA	C62, C62.0, C62.00, C62.01, C62.02, C62.1, C62.10, C62.11, C62.12, C62.9, C62.90, C62.91, C62.92	186, 186.0, 186.9
Kidney cancer	VI, VIa, VIa1, VIa2, VIa3, VIa4, VIb, VIc	C64, C64.0, C64.1, C64.2, C64.4, C64.5, C64.6, C64.8, C64.9, C65, C65.0, C65.1, C65.2, C65.9	189.0, 189.1, 189.5, 189.6, 209.24

Bladder cancer	XIf8	C67, C67.0, C67.1, C67.2, C67.3, C67.4, C67.5, C67.6, C67.7, C67.8, C67.9	188, 188.0, 188.1, 188.2, 188.3, 188.4, 188.5, 188.6, 188.7, 188.8, 188.9
Brain and nervous system cancer	III, IIIa, IIIa1, IIIa2, IIIb, IIIc, IIIc1, IIIc2, IIIc3, IIIc4, IIId, IIId1, IIId2, IIId3, IIIe, IIIe1, IIIe2, IIIe3, IIIe4, IIIe5, IIIf, Xa, Xa1, Xa2, Xa3, Xa4, Xa5, Xa6	C70, C70.0, C70.1, C70.5, C70.6, C70.9, C71, C71.0, C71.1, C71.2, C71.3, C71.4, C71.5, C71.6, C71.7, C71.8, C71.9, C72, C72.0, C72.1, C72.2, C72.20, C72.21, C72.22, C72.3, C72.30, C72.31, C72.32, C72.4, C72.40, C72.41, C72.42, C72.5, C72.50, C72.59, C72.8, C72.9	191, 191.0, 191.1, 191.2, 191.3, 191.4, 191.5, 191.6, 191.7, 191.8, 191.9, 192, 192.0, 192.1, 192.2, 192.3, 192.4, 192.8, 192.9
Thyroid cancer	XIb	C73, C73.0, C73.1, C73.2, C73.3, C73.4, C73.5, C73.8, C73.9	193, 193.0, 193.2, 193.9
Mesothelioma	XIIa5	C45, C45.0, C45.1, C45.2, C45.3, C45.4, C45.5, C45.6, C45.7, C45.8, C45.9	NA
Hodgkin lymphoma	IIa	C81, C81.0, C81.00, C81.01, C81.02, C81.03, C81.04, C81.05, C81.06, C81.07, C81.08, C81.09, C81.1, C81.10, C81.11, C81.12, C81.13, C81.14, C81.15, C81.16, C81.17, C81.18, C81.19, C81.2, C81.20, C81.21, C81.22, C81.23, C81.24, C81.25, C81.26, C81.27, C81.28, C81.29, C81.3, C81.30, C81.31, C81.32, C81.33, C81.34, C81.35, C81.36, C81.37, C81.38, C81.39, C81.4, C81.40, C81.41, C81.42, C81.43, C81.44, C81.45, C81.46, C81.47, C81.48, C81.49, C81.5, C81.6, C81.7, C81.70, C81.71, C81.72, C81.73, C81.74, C81.75, C81.76, C81.77, C81.78, C81.79, C81.8, C81.9, C81.90, C81.91, C81.92, C81.93, C81.94, C81.95, C81.96, C81.97, C81.98, C81.99	201, 201.0, 201.00, 201.01, 201.02, 201.03, 201.04, 201.05, 201.06, 201.07, 201.08, 201.1, 201.10, 201.11, 201.12, 201.13, 201.14, 201.15, 201.16, 201.17, 201.18, 201.2, 201.20, 201.21, 201.22, 201.23, 201.24, 201.25, 201.26, 201.27, 201.28, 201.4, 201.40, 201.41, 201.42, 201.43, 201.44, 201.45, 201.46, 201.47, 201.48, 201.5, 201.50, 201.51, 201.52, 201.53, 201.54, 201.55, 201.56, 201.57, 201.58, 201.6, 201.60, 201.61, 201.62, 201.63, 201.64, 201.65, 201.66, 201.67, 201.68, 201.7, 201.70, 201.71, 201.72, 201.73, 201.74, 201.75, 201.76, 201.77, 201.78, 201.9, 201.90, 201.91, 201.92, 201.93, 201.94, 201.95, 201.96, 201.97, 201.98
Non-Hodgkin lymphoma	IIb, IIb1, IIb2, IIb3, IIb4, IIc, IId, IIe	C83.7, C83.70, C83.71, C83.72, C83.73, C83.74, C83.75, C83.76, C83.77, C83.78, C83.79, C83.8, C82, C82.0, C82.00, C82.01, C82.02, C82.03, C82.04, C82.05, C82.06, C82.07, C82.08, C82.09, C82.1, C82.10, C82.11, C82.12, C82.13, C82.14, C82.15, C82.16, C82.17, C82.18, C82.19, C82.2, C82.20, C82.21, C82.22, C82.23, C82.24, C82.25, C82.26, C82.27, C82.28, C82.29, C82.3, C82.30, C82.31, C82.32, C82.33, C82.34, C82.35, C82.36, C82.37, C82.38, C82.39, C82.4, C82.40, C82.41, C82.42, C82.43, C82.44, C82.45, C82.46, C82.47, C82.48, C82.49, C82.5, C82.50, C82.51, C82.52, C82.53,	200.2, 200.20, 200.21, 200.22, 200.23, 200.24, 200.25, 200.26, 200.27, 200.28, 200, 200.0, 200.00, 200.01, 200.02, 200.03, 200.04, 200.05, 200.06, 200.07, 200.08, 200.1, 200.10, 200.11, 200.12, 200.13, 200.14, 200.15, 200.16, 200.17, 200.18, 200.3, 200.30, 200.31, 200.32, 200.33, 200.34, 200.35, 200.36, 200.37, 200.38, 200.4, 200.40, 200.41, 200.42, 200.43, 200.44, 200.45, 200.46, 200.47, 200.48, 200.5,

		C82.54, C82.55, C82.56, C82.57, C82.58, C82.59, C82.6, C82.60, C82.61, C82.62, C82.63, C82.64, C82.65, C82.66, C82.67, C82.68, C82.69, C82.7, C82.8, C82.80, C82.81, C82.82, C82.83, C82.84, C82.85, C82.86, C82.87, C82.88, C82.89, C82.9, C82.90, C82.91, C82.92, C82.93, C82.94, C82.95, C82.96, C82.97, C82.98, C82.99, C83, C83.0, C83.00, C83.01, C83.02, C83.03, C83.04, C83.05, C83.06, C83.07, C83.08, C83.09, C83.1, C83.10, C83.11, C83.12, C83.13, C83.14, C83.15, C83.16, C83.17, C83.18, C83.19, C83.2, C83.3, C83.30, C83.31, C83.32, C83.33, C83.34, C83.35, C83.36, C83.37, C83.38, C83.39, C83.4, C83.5, C83.50, C83.51, C83.52, C83.53, C83.54, C83.55, C83.56, C83.57, C83.58, C83.59, C83.6, C83.80, C83.81, C83.82, C83.83, C83.84, C83.85, C83.86, C83.87, C83.88, C83.89, C83.9, C83.90, C83.91, C83.92, C83.93, C83.94, C83.95, C83.96, C83.97, C83.98, C83.99, C84, C84.0, C84.00, C84.01, C84.02, C84.03, C84.04, C84.05, C84.06, C84.07, C84.08, C84.09, C84.1, C84.10, C84.11, C84.12, C84.13, C84.14, C84.15, C84.16, C84.17, C84.18, C84.19, C84.2, C84.3, C84.4, C84.40, C84.41, C84.42, C84.43, C84.44, C84.45, C84.46, C84.47, C84.48, C84.49, C84.5, C84.6, C84.60, C84.61, C84.62, C84.63, C84.64, C84.65, C84.66, C84.67, C84.68, C84.69, C84.7, C84.70, C84.71, C84.72, C84.73, C84.74, C84.75, C84.76, C84.77, C84.78, C84.79, C84.8, C84.9, C84.90, C84.91, C84.92, C84.93, C84.94, C84.95, C84.96, C84.97, C84.98, C84.99, C85, C85.0, C85.1, C85.10, C85.11, C85.12, C85.13, C85.14, C85.15, C85.16, C85.17, C85.18, C85.19, C85.2, C85.20, C85.21, C85.22, C85.23, C85.24, C85.25, C85.26, C85.27, C85.28, C85.29, C85.3, C85.4, C85.5, C85.6, C85.7, C85.8, C85.80, C85.81, C85.82, C85.83, C85.84, C85.85, C85.86, C85.87, C85.88, C85.89, C85.9, C85.90, C85.91, C85.92, C85.93, C85.94, C85.95, C85.96, C85.97, C85.98, C85.99, C86, C86.0, C86.1, C86.2, C86.3, C86.4, C86.5, C86.6, C96, C96.0, C96.1, C96.2, C96.3, C96.4, C96.5, C96.6, C96.7, C96.8, C96.9	200.50, 200.51, 200.52, 200.53, 200.54, 200.55, 200.56, 200.57, 200.58, 200.6, 200.60, 200.61, 200.62, 200.63, 200.64, 200.65, 200.66, 200.67, 200.68, 200.7, 200.70, 200.71, 200.72, 200.73, 200.74, 200.75, 200.76, 200.77, 200.78, 200.8, 200.80, 200.81, 200.82, 200.83, 200.84, 200.85, 200.86, 200.87, 200.88, 200.9, 202, 202.0, 202.00, 202.01, 202.02, 202.03, 202.04, 202.05, 202.06, 202.07, 202.08, 202.1, 202.10, 202.11, 202.12, 202.13, 202.14, 202.15, 202.16, 202.17, 202.18, 202.2, 202.20, 202.21, 202.22, 202.23, 202.24, 202.25, 202.26, 202.27, 202.28, 202.3, 202.30, 202.31, 202.32, 202.33, 202.34, 202.35, 202.36, 202.37, 202.38, 202.4, 202.40, 202.41, 202.42, 202.43, 202.44, 202.45, 202.46, 202.47, 202.48, 202.5, 202.50, 202.51, 202.52, 202.53, 202.54, 202.55, 202.56, 202.57, 202.58, 202.6, 202.60, 202.61, 202.62, 202.63, 202.64, 202.65, 202.66, 202.67, 202.68, 202.7, 202.70, 202.71, 202.72, 202.73, 202.74, 202.75, 202.76, 202.77, 202.78, 202.8, 202.80, 202.81, 202.82, 202.83, 202.84, 202.85, 202.86, 202.87, 202.88, 202.9, 202.90, 202.91, 202.92, 202.93, 202.94, 202.95, 202.96, 202.97, 202.98
Multiple myeloma	NA	C88, C88.0, C88.00, C88.01, C88.1, C88.2, C88.20, C88.3, C88.4, C88.40, C88.7, C88.70, C88.71, C88.8, C88.9, C89, C90, C90.0, C90.00, C90.01, C90.02, C90.1, C90.10, C90.11, C90.12, C90.2, C90.20, C90.21, C90.22, C90.3, C90.30,	203, 203.0, 203.00, 203.01, 203.02, 203.1, 203.10, 203.11, 203.12, 203.8, 203.80, 203.81, 203.82, 203.9

		C90.31, C90.32, C90.4, C90.5, C90.6, C90.7, C90.8, C90.9	
Acute lymphoid leukemia	Ia, Ia1, Ia2, Ia3, Ia4	C91.0, C91.00, C91.01, C91.02, C91.2, C91.3, C91.30, C91.31, C91.32, C91.6, C91.60, C91.61, C91.62	204.0, 204.00, 204.01, 204.02, 204.2, 204.20, 204.21, 204.22
Acute myeloid leukemia	Ib	C92.0, C92.00, C92.01, C92.02, C92.3, C92.30, C92.31, C92.32, C92.4, C92.40, C92.41, C92.42, C92.5, C92.50, C92.51, C92.52, C92.6, C92.60, C92.61, C92.62, C93.0, C93.00, C93.01, C93.02, C94.0, C94.00, C94.01, C94.02, C94.2, C94.20, C94.21, C94.22, C94.4, C94.40, C94.41, C94.42, C94.5	205.0, 205.00, 205.01, 205.02, 205.2, 205.20, 205.21, 205.22, 205.3, 205.30, 205.31, 205.32, 206.0, 206.00, 206.01, 206.02, 207.0, 207.00, 207.01, 207.02, 207.20, 207.8, 207.80, 207.81, 207.82
Chronic lymphoid leukemia	Custom mapping (see footnote)	Custom mapping (see footnote)	Custom mapping (see footnote)
Chronic myeloid leukemia	Ic	C92.1, C92.10, C92.11, C92.12, C92.2, C92.20, C92.21, C92.22	205.1, 205.10, 205.11, 205.12
Other leukemia	Ie	C91.20, C91.70, C92.70, C92.80, C93, C93.1, C93.10, C93.11, C93.12, C93.3, C93.30, C93.31, C93.32, C93.8, C94, C94.1, C94.3, C94.30, C94.31, C94.32, C94.50, C94.6, C94.60, C94.7, C94.70, C94.8, C94.80, C94.81, C94.82, C95, C95.0, C95.00, C95.01, C95.02, C95.1, C95.10, C95.11, C95.12, C95.2, C95.4, C95.6, C95.7, C95.70, C95.9, C95.90, C95.91, C95.92	205.92, 206.1, 206.10, 206.11, 206.12, 207, 207.1, 207.10, 207.11, 207.12, 207.2, 207.21, 207.22, 207.9, 208, 208.0, 208.00, 208.01, 208.02, 208.1, 208.10, 208.11, 208.12, 208.2, 208.20, 208.21, 208.22, 208.4, 208.7, 208.8, 208.80, 208.81, 208.82, 208.9, 208.90, 208.91, 208.92
Other malignant neoplasms	VIII, VIIIa, VIIIb, VIIIc, VIIIc1, VIIIc2, VIIId, VIIId1, VIIId2, VIIId3, VIIId4, VIIIe, XIf9, V, IVa, IVb, XIIa1, XIIa4, XIIa6, XIIb, XIa, XIf10, XIf11, XIf5, Xb, Xb1, Xb2, Xb3, Xb4, Xb5, Xb6, IX, IXa, IXb, IXb1, IXb2, IXb3, IXd, IXd1, IXd10, IXd11, IXd2, IXd3, IXd4, IXd5, IXd6, IXd7, IXd8, IXd9, IXe	C40, C40.0, C40.00, C40.01, C40.02, C40.1, C40.10, C40.11, C40.12, C40.2, C40.20, C40.21, C40.22, C40.3, C40.30, C40.31, C40.32, C40.8, C40.80, C40.81, C40.82, C40.9, C40.90, C40.91, C40.92, C41, C41.0, C41.01, C41.02, C41.1, C41.2, C41.3, C41.4, C41.5, C41.6, C41.7, C41.8, C41.9, C42.0, C42.1, C42.2, C42.3, C42.4, C69.0, C69.00, C69.01, C69.02, C69.1, C69.10, C69.11, C69.12, C69.3, C69.30, C69.31, C69.32, C69.4, C69.40, C69.41, C69.42, C69.5, C69.50, C69.51, C69.52, C69.6, C69.60, C69.61, C69.62, C69.7, C69.8, C69.80, C69.81, C69.82, C69.2, C69.20, C69.21, C69.22, C47, C47.0, C47.1, C47.10, C47.11, C47.12, C47.2, C47.20, C47.21, C47.22, C47.3, C47.4, C47.5, C47.6, C47.8, C47.9, C74.90, C17, C17.0, C17.1, C17.2, C17.3, C17.8, C17.9, C3, C30, C30.0, C30.1, C30.2, C30.3, C30.5, C30.8, C30.9, C31, C31.0, C31.1, C31.2, C31.3, C31.8, C31.9, C37, C37.0, C37.1, C37.2, C37.3, C37.9, C38, C38.0, C38.1, C38.2, C38.3, C38.4, C38.8, C4, C48, C48.0, C48.1, C48.2, C48.8, C48.9, C4A, C5, C51,	170, 170.0, 170.1, 170.2, 170.3, 170.4, 170.5, 170.6, 170.7, 170.8, 170.9, 190, 190.0, 190.1, 190.2, 190.3, 190.4, 190.6, 190.7, 190.8, 190.5, 152, 152.0, 152.1, 152.2, 152.3, 152.4, 152.6, 152.8, 152.9, 158, 158.0, 158.3, 158.4, 158.5, 158.6, 158.8, 158.9, 160, 160.0, 160.1, 160.2, 160.3, 160.4, 160.5, 160.6, 160.8, 160.9, 163, 163.0, 163.1, 163.3, 163.5, 163.8, 163.9, 164, 164.0, 164.1, 164.2, 164.3, 164.8, 164.9, 181, 181.0, 181.9, 183.2, 183.3, 183.4, 183.5, 183.8, 184.0, 184.1, 184.2, 184.3, 184.4, 184.8, 187.1, 187.2, 187.3, 187.4, 187.5, 187.6, 187.7, 187.8, 189.2, 189.3, 189.4, 189.8, 194.1, 194.3, 194.4, 194.5, 194.6, 194.8, 209.0, 209.00,

		C51.0, C51.1, C51.2, C51.8, C51.9, C52, C52.0, C52.9, C57, C57.0, C57.00, C57.01, C57.02, C57.1, C57.10, C57.11, C57.12, C57.2, C57.20, C57.21, C57.22, C57.3, C57.4, C57.7, C57.8, C58, C58.0, C58.9, C60, C60.0, C60.1, C60.2, C60.8, C60.9, C63, C63.0, C63.00, C63.01, C63.02, C63.1, C63.10, C63.11, C63.12, C63.2, C63.7, C63.8, C66, C66.0, C66.1, C66.2, C66.9, C68.0, C68.1, C68.8, C7, C75, C75.0, C75.1, C75.2, C75.3, C75.4, C75.5, C75.6, C75.8, C49, C49.0, C49.1, C49.10, C49.11, C49.12, C49.2, C49.20, C49.21, C49.22, C49.3, C49.4, C49.5, C49.6, C49.8, C49.9	209.01, 209.02, 209.03, 209.22, 209.25, 209.26, 209.27, 209.31, 209.32, 209.33, 209.34, 209.35, 209.36, 171, 171.0, 171.2, 171.3, 171.4, 171.5, 171.6, 171.7, 171.8, 171.9

Abbreviations: ICCC3, International Classification of Childhood Cancer, Third Edition; ICD-9, International Classification of Diseases, Ninth Revision; ICD-10, International Classification of Diseases, Tenth Revision; NA, not applicable.

Footnote: Cancers with NA do not have any relevant codes mapped from ICCC3. Chronic lymphoid leukemia is only modeled for ages 20 years and above in GBD. ICD codes (ICD-9: 204.1, 204.10, 204.11, and 204.12; ICD-10: C91.1, C91.10, C91.11, and C91.12) under 20 years are redistributed (see Section “6. Redistribution” in this appendix for more information) to “Acute lymphoid leukemia,” while these ICD codes over 20 years old are mapped directly to “Chronic lymphoid leukemia.”

eTable 4: List of International Classification of Diseases (ICD) codes mapped to the Global Burden of Disease cause list for cancer mortality data

Cause	ICCC3	ICD10	ICD9
Lip and oral cavity cancer	XIf1	C00, C00.0, C00.1, C00.2, C00.3, C00.4, C00.5, C00.6, C00.8, C00.9, C01, C01.9, C02, C02.0, C02.1, C02.2, C02.3, C02.4, C02.8, C02.9, C03, C03.0, C03.1, C03.9, C04, C04.0, C04.1, C04.8, C04.9, C05, C05.0, C05.1, C05.2, C05.8, C05.9, C06, C06.0, C06.1, C06.2, C06.8, C06.80, C06.89, C06.9, C07, C07.0, C07.9, C08, C08.0, C08.1, C08.8, C08.9, D00.00, D00.01, D00.02, D00.03, D00.04, D00.05, D00.06, D00.07, D10.0, D10.1, D10.2, D10.3, D10.30, D10.39, D10.4, D10.5, D11, D11.0, D11.7, D11.9, D37.01, D37.02, D37.03, D37.030, D37.031, D37.032, D37.039, D37.04, D37.09	140, 140.0, 140.1, 140.2, 140.3, 140.4, 140.5, 140.6, 140.7, 140.8, 140.9, 141, 141.0, 141.1, 141.2, 141.3, 141.4, 141.5, 141.6, 141.8, 141.9, 142, 142.0, 142.1, 142.2, 142.3, 142.8, 142.9, 143, 143.0, 143.1, 143.8, 143.9, 144, 144.0, 144.1, 144.4, 144.8, 144.9, 145, 145.0, 145.1, 145.2, 145.3, 145.4, 145.5, 145.6, 145.8, 145.9, 210, 210.0, 210.1, 210.2, 210.3, 210.4, 210.5, 210.6, 235, 235.0
Nasopharynx cancer	XIc	C11, C11.0, C11.1, C11.2, C11.3, C11.8, C11.9, D00.08, D10.6, D37.05	147, 147.0, 147.1, 147.2, 147.3, 147.8, 147.9, 210.7, 210.8, 210.9
Other pharynx cancer	NA	C09, C09.0, C09.1, C09.8, C09.9, C1, C10, C10.0, C10.1, C10.2, C10.3, C10.4, C10.8, C10.9, C12, C12.0, C12.9, C13, C13.0, C13.1, C13.2, C13.8, C13.9, D10.7	146, 146.0, 146.1, 146.2, 146.3, 146.4, 146.5, 146.6, 146.7, 146.8, 146.9, 148, 148.0, 148.1, 148.2, 148.3, 148.4, 148.5, 148.8, 148.9
Esophageal cancer	NA	C15, C15.0, C15.1, C15.2, C15.3, C15.4, C15.5, C15.8, C15.9, D00.1, D13.0	150, 150.0, 150.1, 150.2, 150.3, 150.4, 150.5, 150.6, 150.7, 150.8, 150.9, 211, 211.0, 230.1
Stomach cancer	NA	C16, C16.0, C16.1, C16.2, C16.3, C16.4, C16.5, C16.6, C16.7, C16.8, C16.9, D00.2, D13.1, D37.1	151, 151.0, 151.1, 151.2, 151.3, 151.4, 151.5, 151.6, 151.8, 151.9, 209.23, 209.63, 211.1, 230.2
Colon and rectum cancer	XIf2, XIf3	C18, C18.0, C18.1, C18.2, C18.3, C18.4, C18.5, C18.6, C18.7, C18.8, C18.9, C19, C19.0, C19.9, C2, C20, C20.0, C20.8, C20.9, C21, C21.0, C21.1, C21.2, C21.8, C21.9, D01.0, D01.1, D01.2, D01.3, D12, D12.0, D12.1, D12.2, D12.3, D12.4, D12.5, D12.6, D12.7, D12.8, D12.9, D37.3, D37.4, D37.5	153, 153.0, 153.1, 153.2, 153.3, 153.4, 153.5, 153.6, 153.7, 153.8, 153.9, 154, 154.0, 154.1, 154.2, 154.3, 154.4, 154.8, 154.9, 209.1, 209.10, 209.11, 209.12, 209.13, 209.14, 209.15, 209.16, 209.17, 209.5, 209.50, 209.51, 209.52, 209.53, 209.54, 209.55, 209.56, 209.57, 211.3, 211.4, 230.3, 230.4, 230.5, 230.6, 569.0, 569.43, 569.44, 569.84, 569.85
Liver cancer	VIIb, VIIc	C22, C22.0, C22.1, C22.3, C22.4, C22.5, C22.7, C22.8, D13.4	155, 155.0, 155.1, 155.3, 155.5, 155.9, 211.5
Gallbladder and biliary tract cancer	NA	C23, C23.0, C23.9, C24, C24.0, C24.1, C24.4, C24.8, C24.9, D13.5	156, 156.0, 156.1, 156.2, 156.3, 156.8, 156.9, 209.65, 209.66, 209.67
Pancreatic cancer	XIIa2	C25, C25.0, C25.1, C25.2, C25.3, C25.4, C25.7, C25.8, C25.9, D13.6, D13.7	157, 157.0, 157.1, 157.2, 157.3, 157.4, 157.5, 157.7, 157.8, 157.9, 211.6, 211.7
Larynx cancer	NA	C32, C32.0, C32.1, C32.2, C32.3, C32.8, C32.9, D02.0, D14.1, D38.0	161, 161.0, 161.1, 161.2, 161.3, 161.8, 161.9, 212.1, 231, 231.0, 235.6

Tracheal, bronchus, and lung cancer	XIIa3, XIf4	C33, C33.0, C33.2, C33.9, C34, C34.0, C34.00, C34.01, C34.02, C34.1, C34.10, C34.11, C34.12, C34.2, C34.3, C34.30, C34.31, C34.32, C34.4, C34.7, C34.8, C34.80, C34.81, C34.82, C34.9, C34.90, C34.91, C34.92, D02.1, D02.2, D02.20, D02.21, D02.22, D02.3, D14.2, D14.3, D14.30, D14.31, D14.32, D38.1	162, 162.0, 162.1, 162.2, 162.3, 162.4, 162.5, 162.8, 162.9, 209.21, 209.61, 212.2, 212.3, 231.1, 231.2, 235.7
Malignant skin melanoma	XId	C43, C43.0, C43.1, C43.10, C43.11, C43.12, C43.2, C43.20, C43.21, C43.22, C43.3, C43.30, C43.31, C43.39, C43.4, C43.5, C43.51, C43.52, C43.59, C43.6, C43.60, C43.61, C43.62, C43.7, C43.70, C43.71, C43.72, C43.8, C43.9, D03, D03.0, D03.1, D03.10, D03.11, D03.12, D03.2, D03.20, D03.21, D03.22, D03.3, D03.30, D03.39, D03.4, D03.5, D03.51, D03.52, D03.59, D03.6, D03.60, D03.61, D03.62, D03.7, D03.70, D03.71, D03.72, D03.8, D03.9, D22, D22.0, D22.1, D22.10, D22.11, D22.12, D22.2, D22.20, D22.21, D22.22, D22.3, D22.30, D22.39, D22.4, D22.5, D22.6, D22.60, D22.61, D22.62, D22.7, D22.70, D22.71, D22.72, D22.9, D23, D23.0, D23.1, D23.10, D23.11, D23.12, D23.2, D23.20, D23.21, D23.22, D23.3, D23.30, D23.39, D23.4, D23.5, D23.6, D23.60, D23.61, D23.62, D23.7, D23.70, D23.71, D23.72, D23.9	172, 172.0, 172.1, 172.2, 172.3, 172.4, 172.5, 172.6, 172.7, 172.8, 172.9
Non-melanoma skin cancer	XIe	D48.5	222.4
Non-melanoma skin cancer (squamous-cell carcinoma)	NA	C44, C44.0, C44.00, C44.01, C44.02, C44.09, C44.1, C44.10, C44.101, C44.102, C44.109, C44.11, C44.111, C44.112, C44.119, C44.12, C44.121, C44.122, C44.129, C44.19, C44.191, C44.192, C44.199, C44.2, C44.20, C44.201, C44.202, C44.209, C44.21, C44.211, C44.212, C44.219, C44.22, C44.221, C44.222, C44.229, C44.29, C44.291, C44.292, C44.299, C44.3, C44.30, C44.300, C44.301, C44.309, C44.31, C44.310, C44.311, C44.319, C44.32, C44.320, C44.321, C44.329, C44.39, C44.390, C44.391, C44.399, C44.4, C44.40, C44.41, C44.42, C44.49, C44.5, C44.50, C44.500, C44.501, C44.509, C44.51, C44.510, C44.511, C44.519, C44.52, C44.520, C44.521, C44.529, C44.59, C44.590, C44.591, C44.599, C44.6, C44.60, C44.601, C44.602, C44.609, C44.61, C44.611, C44.612, C44.619, C44.62, C44.621, C44.622, C44.629, C44.69, C44.691, C44.692, C44.699, C44.7, C44.70, C44.701, C44.702, C44.709, C44.71, C44.711, C44.712, C44.719, C44.72, C44.721, C44.722, C44.729, C44.79, C44.791, C44.792, C44.799, C44.8, C44.80, C44.81, C44.82, C44.89, C44.9, C44.90, C44.91, C44.92, C44.99, D04, D04.0, D04.1, D04.10, D04.11, D04.12, D04.2, D04.20, D04.21,	173, 173.0, 173.00, 173.01, 173.02, 173.09, 173.1, 173.10, 173.11, 173.12, 173.19, 173.2, 173.20, 173.21, 173.22, 173.29, 173.3, 173.30, 173.31, 173.32, 173.39, 173.4, 173.40, 173.41, 173.42, 173.49, 173.5, 173.50, 173.51, 173.52, 173.59, 173.6, 173.60, 173.61, 173.62, 173.69, 173.7, 173.70, 173.71, 173.72, 173.79, 173.8, 173.80, 173.81, 173.82, 173.89, 173.9, 173.90, 173.91, 173.92, 173.99, 232, 232.0, 232.1, 232.2, 232.3, 232.4, 232.5, 232.6, 232.7, 232.8, 232.9, 238.2

		D04.22, D04.3, D04.30, D04.39, D04.4, D04.5, D04.6, D04.60, D04.61, D04.62, D04.7, D04.70, D04.71, D04.72, D04.8, D04.9, D49.2	
Breast cancer	XIf6	C50, C50.0, C50.01, C50.011, C50.012, C50.019, C50.02, C50.021, C50.022, C50.029, C50.1, C50.11, C50.111, C50.112, C50.119, C50.12, C50.121, C50.122, C50.129, C50.2, C50.21, C50.211, C50.212, C50.219, C50.22, C50.221, C50.222, C50.229, C50.3, C50.31, C50.311, C50.312, C50.319, C50.32, C50.321, C50.322, C50.329, C50.4, C50.41, C50.411, C50.412, C50.419, C50.42, C50.421, C50.422, C50.429, C50.5, C50.51, C50.511, C50.512, C50.519, C50.52, C50.521, C50.522, C50.529, C50.6, C50.61, C50.611, C50.612, C50.619, C50.62, C50.621, C50.622, C50.629, C50.7, C50.8, C50.81, C50.811, C50.812, C50.819, C50.82, C50.821, C50.822, C50.829, C50.9, C50.91, C50.911, C50.912, C50.919, C50.92, C50.921, C50.922, C50.929, D05, D05.0, D05.00, D05.01, D05.02, D05.1, D05.10, D05.11, D05.12, D05.7, D05.8, D05.80, D05.81, D05.82, D05.9, D05.90, D05.91, D05.92, D24, D24.0, D24.1, D24.2, D24.9, D48.6, D48.60, D48.61, D48.62, D49.3	174, 174.0, 174.1, 174.2, 174.3, 174.4, 174.5, 174.6, 174.8, 174.9, 175, 175.0, 175.3, 175.9, 217, 217.0, 217.8, 233, 233.0, 238.3, 239.3, 610, 610.0, 610.1, 610.2, 610.3, 610.4, 610.8, 610.9
Cervical cancer	XIf7	C53, C53.0, C53.1, C53.3, C53.4, C53.8, C53.9, D06, D06.0, D06.1, D06.7, D06.9, D26.0	180, 180.0, 180.1, 180.2, 180.3, 180.4, 180.5, 180.6, 180.8, 180.9, 219, 219.0, 233.1, 622.1, 622.10, 622.11, 622.12, 622.2, 622.7
Uterine cancer	NA	C54, C54.0, C54.1, C54.2, C54.3, C54.4, C54.8, C54.9, D07.0, D07.1, D07.2, D26.1, D26.7, D26.9	182, 182.0, 182.1, 182.8, 182.9, 233.2
Ovarian cancer	NA	C56, C56.0, C56.1, C56.2, C56.4, C56.9, D27, D27.0, D27.1, D27.9, D39.1, D39.10, D39.11, D39.12	183, 183.0, 220, 220.0, 220.9, 236.2
Prostate cancer	NA	C61, C61.0, C61.9, D07.5, D29.1, D40.0	185, 185.0, 185.9, 222.2, 236.5
Testicular cancer	NA	C62, C62.0, C62.00, C62.01, C62.02, C62.1, C62.10, C62.11, C62.12, C62.9, C62.90, C62.91, C62.92, D29.2, D29.20, D29.21, D29.22, D29.3, D29.30, D29.31, D29.32, D29.4, D29.7, D29.8, D40.1, D40.10, D40.11, D40.12, D40.7, D40.8	186, 186.0, 186.9, 222, 222.0, 222.3, 236.4
Kidney cancer	VI, VIa, VIa1, VIa2, VIa3, VIa4, VIb, VIc	C64, C64.0, C64.1, C64.2, C64.4, C64.5, C64.6, C64.8, C64.9, C65, C65.0, C65.1, C65.2, C65.9, D30.0, D30.00, D30.01, D30.02, D30.1, D30.10, D30.11, D30.12, D41.0, D41.00, D41.01, D41.02, D41.1, D41.10, D41.11, D41.12	189.0, 189.1, 189.5, 189.6, 209.24, 209.64, 223, 223.0, 223.1, 236.91
Bladder cancer	XIf8	C67, C67.0, C67.1, C67.2, C67.3, C67.4, C67.5, C67.6, C67.7, C67.8, C67.9, D09.0, D30.3, D41.4, D41.7, D41.8, D49.4	188, 188.0, 188.1, 188.2, 188.3, 188.4, 188.5, 188.6, 188.7, 188.8, 188.9, 223.3, 233.7, 236.7, 239.4
Brain and central nervous system cancer	III, IIIa, IIIa1, IIIa2, IIIb, IIIc, IIIc1, IIIc2, IIIc3, IIIc4, IIId, IIId1, IIId2, IIId3,	C70, C70.0, C70.1, C70.5, C70.6, C70.9, C71, C71.0, C71.1, C71.2, C71.3, C71.4, C71.5, C71.6, C71.7, C71.8, C71.9, C72, C72.0, C72.1, C72.2, C72.20, C72.21, C72.22, C72.3, C72.30, C72.31, C72.32, C72.4, C72.40, C72.41, C72.42, C72.5, C72.50, C72.59, C72.8, C72.9	191, 191.0, 191.1, 191.2, 191.3, 191.4, 191.5, 191.6, 191.7, 191.8, 191.9, 192, 192.0, 192.1, 192.2, 192.3, 192.4, 192.8, 192.9

	IIIe, IIIe1, IIIe2, IIIe3, IIIe4, IIIe5, IIIf, Xa, Xa1, Xa2, Xa3, Xa4, Xa5, Xa6		
Thyroid cancer	XIb	C73, C73.0, C73.1, C73.2, C73.3, C73.4, C73.5, C73.8, C73.9, D09.3, D09.8, D34, D34.0, D34.9, D44.0	193, 193.0, 193.2, 193.9, 226, 226.0, 226.9
Mesothelioma	XIIa5	C45, C45.0, C45.1, C45.2, C45.3, C45.4, C45.5, C45.6, C45.7, C45.8, C45.9	NA
Hodgkin lymphoma	IIa	C81, C81.0, C81.00, C81.01, C81.02, C81.03, C81.04, C81.05, C81.06, C81.07, C81.08, C81.09, C81.1, C81.10, C81.11, C81.12, C81.13, C81.14, C81.15, C81.16, C81.17, C81.18, C81.19, C81.2, C81.20, C81.21, C81.22, C81.23, C81.24, C81.25, C81.26, C81.27, C81.28, C81.29, C81.3, C81.30, C81.31, C81.32, C81.33, C81.34, C81.35, C81.36, C81.37, C81.38, C81.39, C81.4, C81.40, C81.41, C81.42, C81.43, C81.44, C81.45, C81.46, C81.47, C81.48, C81.49, C81.5, C81.6, C81.7, C81.70, C81.71, C81.72, C81.73, C81.74, C81.75, C81.76, C81.77, C81.78, C81.79, C81.8, C81.9, C81.90, C81.91, C81.92, C81.93, C81.94, C81.95, C81.96, C81.97, C81.98, C81.99	201, 201.0, 201.00, 201.01, 201.02, 201.03, 201.04, 201.05, 201.06, 201.07, 201.08, 201.1, 201.10, 201.11, 201.12, 201.13, 201.14, 201.15, 201.16, 201.17, 201.18, 201.2, 201.20, 201.21, 201.22, 201.23, 201.24, 201.25, 201.26, 201.27, 201.28, 201.4, 201.40, 201.41, 201.42, 201.43, 201.44, 201.45, 201.46, 201.47, 201.48, 201.5, 201.50, 201.51, 201.52, 201.53, 201.54, 201.55, 201.56, 201.57, 201.58, 201.6, 201.60, 201.61, 201.62, 201.63, 201.64, 201.65, 201.66, 201.67, 201.68, 201.7, 201.70, 201.71, 201.72, 201.73, 201.74, 201.75, 201.76, 201.77, 201.78, 201.9, 201.90, 201.91, 201.92, 201.93, 201.94, 201.95, 201.96, 201.97, 201.98
Non-Hodgkin lymphoma	IIb, IIb1, IIb2, IIb3, IIb4, IIc, IId, IIe	C83.7, C83.70, C83.71, C83.72, C83.73, C83.74, C83.75, C83.76, C83.77, C83.78, C83.79, C83.8, C82, C82.0, C82.00, C82.01, C82.02, C82.03, C82.04, C82.05, C82.06, C82.07, C82.08, C82.09, C82.1, C82.10, C82.11, C82.12, C82.13, C82.14, C82.15, C82.16, C82.17, C82.18, C82.19, C82.2, C82.20, C82.21, C82.22, C82.23, C82.24, C82.25, C82.26, C82.27, C82.28, C82.29, C82.3, C82.30, C82.31, C82.32, C82.33, C82.34, C82.35, C82.36, C82.37, C82.38, C82.39, C82.4, C82.40, C82.41, C82.42, C82.43, C82.44, C82.45, C82.46, C82.47, C82.48, C82.49, C82.5, C82.50, C82.51, C82.52, C82.53, C82.54, C82.55, C82.56, C82.57, C82.58, C82.59, C82.6, C82.60, C82.61, C82.62, C82.63, C82.64, C82.65, C82.66, C82.67, C82.68, C82.69, C82.7, C82.8, C82.80, C82.81, C82.82, C82.83, C82.84, C82.85, C82.86, C82.87, C82.88, C82.89, C82.9, C82.90, C82.91, C82.92, C82.93, C82.94, C82.95, C82.96, C82.97, C82.98, C82.99, C83,	200.2, 200.20, 200.21, 200.22, 200.23, 200.24, 200.25, 200.26, 200.27, 200.28, 200, 200.0, 200.00, 200.01, 200.02, 200.03, 200.04, 200.05, 200.06, 200.07, 200.08, 200.1, 200.10, 200.11, 200.12, 200.13, 200.14, 200.15, 200.16, 200.17, 200.18, 200.3, 200.30, 200.31, 200.32, 200.33, 200.34, 200.35, 200.36, 200.37, 200.38, 200.4, 200.40, 200.41, 200.42, 200.43, 200.44, 200.45, 200.46, 200.47, 200.48, 200.5, 200.50, 200.51, 200.52, 200.53, 200.54, 200.55, 200.56, 200.57, 200.58, 200.6, 200.60, 200.61, 200.62, 200.63, 200.64, 200.65, 200.66, 200.67, 200.68, 200.7, 200.70, 200.71, 200.72, 200.73,

		C83.0, C83.00, C83.01, C83.02, C83.03, C83.04, C83.05, C83.06, C83.07, C83.08, C83.09, C83.1, C83.10, C83.11, C83.12, C83.13, C83.14, C83.15, C83.16, C83.17, C83.18, C83.19, C83.2, C83.3, C83.30, C83.31, C83.32, C83.33, C83.34, C83.35, C83.36, C83.37, C83.38, C83.39, C83.4, C83.5, C83.50, C83.51, C83.52, C83.53, C83.54, C83.55, C83.56, C83.57, C83.58, C83.59, C83.6, C83.80, C83.81, C83.82, C83.83, C83.84, C83.85, C83.86, C83.87, C83.88, C83.89, C83.9, C83.90, C83.91, C83.92, C83.93, C83.94, C83.95, C83.96, C83.97, C83.98, C83.99, C84, C84.0, C84.00, C84.01, C84.02, C84.03, C84.04, C84.05, C84.06, C84.07, C84.08, C84.09, C84.1, C84.10, C84.11, C84.12, C84.13, C84.14, C84.15, C84.16, C84.17, C84.18, C84.19, C84.2, C84.3, C84.4, C84.40, C84.41, C84.42, C84.43, C84.44, C84.45, C84.46, C84.47, C84.48, C84.49, C84.5, C84.6, C84.60, C84.61, C84.62, C84.63, C84.64, C84.65, C84.66, C84.67, C84.68, C84.69, C84.7, C84.70, C84.71, C84.72, C84.73, C84.74, C84.75, C84.76, C84.77, C84.78, C84.79, C84.8, C84.9, C84.90, C84.91, C84.92, C84.93, C84.94, C84.95, C84.96, C84.97, C84.98, C84.99, C85, C85.0, C85.1, C85.10, C85.11, C85.12, C85.13, C85.14, C85.15, C85.16, C85.17, C85.18, C85.19, C85.2, C85.20, C85.21, C85.22, C85.23, C85.24, C85.25, C85.26, C85.27, C85.28, C85.29, C85.3, C85.4, C85.5, C85.6, C85.7, C85.8, C85.80, C85.81, C85.82, C85.83, C85.84, C85.85, C85.86, C85.87, C85.88, C85.89, C85.9, C85.90, C85.91, C85.92, C85.93, C85.94, C85.95, C85.96, C85.97, C85.98, C85.99, C86, C86.0, C86.1, C86.2, C86.3, C86.4, C86.5, C86.6, C96, C96.0, C96.1, C96.2, C96.3, C96.4, C96.5, C96.6, C96.7, C96.8, C96.9	200.74, 200.75, 200.76, 200.77, 200.78, 200.8, 200.80, 200.81, 200.82, 200.83, 200.84, 200.85, 200.86, 200.87, 200.88, 200.9, 202, 202.0, 202.00, 202.01, 202.02, 202.03, 202.04, 202.05, 202.06, 202.07, 202.08, 202.1, 202.10, 202.11, 202.12, 202.13, 202.14, 202.15, 202.16, 202.17, 202.18, 202.2, 202.20, 202.21, 202.22, 202.23, 202.24, 202.25, 202.26, 202.27, 202.28, 202.3, 202.30, 202.31, 202.32, 202.33, 202.34, 202.35, 202.36, 202.37, 202.38, 202.4, 202.40, 202.41, 202.42, 202.43, 202.44, 202.45, 202.46, 202.47, 202.48, 202.5, 202.50, 202.51, 202.52, 202.53, 202.54, 202.55, 202.56, 202.57, 202.58, 202.6, 202.60, 202.61, 202.62, 202.63, 202.64, 202.65, 202.66, 202.67, 202.68, 202.7, 202.70, 202.71, 202.72, 202.73, 202.74, 202.75, 202.76, 202.77, 202.78, 202.8, 202.80, 202.81, 202.82, 202.83, 202.84, 202.85, 202.86, 202.87, 202.88, 202.9, 202.90, 202.91, 202.92, 202.93, 202.94, 202.95, 202.96, 202.97, 202.98
Multiple myeloma	NA	C88, C88.0, C88.00, C88.01, C88.1, C88.2, C88.20, C88.3, C88.4, C88.40, C88.7, C88.70, C88.71, C88.8, C88.9, C89, C90, C90.0, C90.00, C90.01, C90.02, C90.1, C90.10, C90.11, C90.12, C90.2, C90.20, C90.21, C90.22, C90.3, C90.30, C90.31, C90.32, C90.4, C90.5, C90.6, C90.7, C90.8, C90.9	203, 203.0, 203.00, 203.01, 203.02, 203.1, 203.10, 203.11, 203.12, 203.8, 203.80, 203.81, 203.82, 203.9
Leukemia	NA	C91, C92	204, 205, 206
Acute lymphoid leukemia	Ia, Ia1, Ia2, Ia3, Ia4	C91.0, C91.00, C91.01, C91.02, C91.2, C91.3, C91.30, C91.31, C91.32, C91.6, C91.60, C91.61, C91.62	204.0, 204.00, 204.01, 204.02, 204.2, 204.20, 204.21, 204.22
Acute myeloid leukemia	Ib	C92.0, C92.00, C92.01, C92.02, C92.3, C92.30, C92.31, C92.32, C92.4, C92.40, C92.41, C92.42, C92.5, C92.50, C92.51, C92.52, C92.6, C92.60, C92.61, C92.62, C93.0, C93.00, C93.01, C93.02, C94.0, C94.00, C94.01, C94.02, C94.2, C94.20, C94.21, C94.22, C94.4, C94.40, C94.41, C94.42, C94.5	205.0, 205.00, 205.01, 205.02, 205.2, 205.20, 205.21, 205.22, 205.3, 205.30, 205.31, 205.32, 206.0, 206.00, 206.01, 206.02, 207.0, 207.00, 207.01, 207.02, 207.20, 207.8, 207.80, 207.81, 207.82

Chronic lymphoid leukemia	Custom mapping (see footnote)	Custom mapping (see footnote)	Custom mapping (see footnote)
Chronic myeloid leukemia	Ic	C92.1, C92.10, C92.11, C92.12, C92.2, C92.20, C92.21, C92.22	205.1, 205.10, 205.11, 205.12
Other leukemia	Ie	C91.20, C91.70, C92.70, C92.80, C93, C93.1, C93.10, C93.11, C93.12, C93.3, C93.30, C93.31, C93.32, C93.8, C94, C94.1, C94.3, C94.30, C94.31, C94.32, C94.50, C94.6, C94.60, C94.7, C94.70, C94.8, C94.80, C94.81, C94.82, C95, C95.0, C95.00, C95.01, C95.02, C95.1, C95.10, C95.11, C95.12, C95.2, C95.4, C95.6, C95.7, C95.70, C95.9, C95.90, C95.91, C95.92	205.92, 206.1, 206.10, 206.11, 206.12, 207, 207.1, 207.10, 207.11, 207.12, 207.2, 207.21, 207.22, 207.9, 208, 208.0, 208.00, 208.01, 208.02, 208.1, 208.10, 208.11, 208.12, 208.2, 208.20, 208.21, 208.22, 208.4, 208.7, 208.8, 208.80, 208.81, 208.82, 208.9, 208.90, 208.91, 208.92
Other malignant neoplasms	VIII, VIIIa, VIIIb, VIIIc, VIIIc1, VIIIc2, VIIId, VIIId1, VIIId2, VIIId3, VIIId4, VIIIe, XIf9, V, IVa, IVb, XIIa1, XIIa4, XIIa6, XIIb, XIa, XIf10, XIf11, XIf5, Xb, Xb1, Xb2, Xb3, Xb4, Xb5, Xb6, IX, IXa, IXb, IXb1, IXb2, IXb3, IXd, IXd1, IXd10, IXd11, IXd2, IXd3, IXd4, IXd5, IXd6, IXd7, IXd8, IXd9, IXe	C40, C40.0, C40.00, C40.01, C40.02, C40.1, C40.10, C40.11, C40.12, C40.2, C40.20, C40.21, C40.22, C40.3, C40.30, C40.31, C40.32, C40.8, C40.80, C40.81, C40.82, C40.9, C40.90, C40.91, C40.92, C41, C41.0, C41.01, C41.02, C41.1, C41.2, C41.3, C41.4, C41.5, C41.6, C41.7, C41.8, C41.9, C42.0, C42.1, C42.2, C42.3, C42.4, C69.0, C69.00, C69.01, C69.02, C69.1, C69.10, C69.11, C69.12, C69.3, C69.30, C69.31, C69.32, C69.4, C69.40, C69.41, C69.42, C69.5, C69.50, C69.51, C69.52, C69.6, C69.60, C69.61, C69.62, C69.7, C69.8, C69.80, C69.81, C69.82, C69.2, C69.20, C69.21, C69.22, C47, C47.0, C47.1, C47.10, C47.11, C47.12, C47.2, C47.20, C47.21, C47.22, C47.3, C47.4, C47.5, C47.6, C47.8, C47.9, C74.90, C17, C17.0, C17.1, C17.2, C17.3, C17.8, C17.9, C3, C30, C30.0, C30.1, C30.2, C30.3, C30.5, C30.8, C30.9, C31, C31.0, C31.1, C31.2, C31.3, C31.8, C31.9, C37, C37.0, C37.1, C37.2, C37.3, C37.9, C38, C38.0, C38.1, C38.2, C38.3, C38.4, C38.8, C4, C48, C48.0, C48.1, C48.2, C48.8, C48.9, C4A, C5, C51, C51.0, C51.1, C51.2, C51.8, C51.9, C52, C52.0, C52.9, C57, C57.0, C57.00, C57.01, C57.02, C57.1, C57.10, C57.11, C57.12, C57.2, C57.20, C57.21, C57.22, C57.3, C57.4, C57.7, C57.8, C58, C58.0, C58.9, C60, C60.0, C60.1, C60.2, C60.8, C60.9, C63, C63.0, C63.00, C63.01, C63.02, C63.1, C63.10, C63.11, C63.12, C63.2, C63.7, C63.8, C66, C66.0, C66.1, C66.2, C66.9, C68.0, C68.1, C68.8, C7, C75, C75.0, C75.1, C75.2, C75.3, C75.4, C75.5, C75.6, C75.8, D07.4, D09.2, D09.20, D09.21, D09.22, D13.2, D13.3, D13.30, D13.39, D14.0, D15, D15.0, D15.1, D15.2, D15.7, D15.9, D16, D16.0, D16.00, D16.01, D16.02, D16.1, D16.10, D16.11, D16.12, D16.2, D16.20,	170, 170.0, 170.1, 170.2, 170.3, 170.4, 170.5, 170.6, 170.7, 170.8, 170.9, 190, 190.0, 190.1, 190.2, 190.3, 190.4, 190.6, 190.7, 190.8, 190.5, 152, 152.0, 152.1, 152.2, 152.3, 152.4, 152.6, 152.8, 152.9, 158, 158.0, 158.3, 158.4, 158.5, 158.6, 158.8, 158.9, 160, 160.0, 160.1, 160.2, 160.3, 160.4, 160.5, 160.6, 160.8, 160.9, 163, 163.0, 163.1, 163.3, 163.5, 163.8, 163.9, 164, 164.0, 164.1, 164.2, 164.3, 164.8, 164.9, 181, 181.0, 181.9, 183.2, 183.3, 183.4, 183.5, 183.8, 184.0, 184.1, 184.2, 184.3, 184.4, 184.8, 187.1, 187.2, 187.3, 187.4, 187.5, 187.6, 187.7, 187.8, 189.2, 189.3, 189.4, 189.8, 194.1, 194.3, 194.4, 194.5, 194.6, 194.8, 209.0, 209.00, 209.01, 209.02, 209.03, 209.22, 209.25, 209.26, 209.27, 209.31, 209.32, 209.33, 209.34, 209.35, 209.36, 209.4, 209.40, 209.41, 209.42, 209.43, 211.2, 211.8, 212.0, 212.4, 212.5, 212.6, 212.7, 212.8, 213, 213.0, 213.1, 213.2, 213.3, 213.4, 213.5, 213.6, 213.7, 213.8, 213.9, 221.0, 221.1, 221.2, 221.8, 222.1, 222.8, 223.2, 223.8, 223.81, 223.89, 224, 224.0, 224.1, 224.2, 224.3, 224.4, 224.5, 224.6, 224.7, 224.8, 224.9, 225, 225.0, 225.1, 225.2, 225.3, 225.4, 225.8, 225.9, 227, 227.0,

		D16.21, D16.22, D16.3, D16.30, D16.31, D16.32, D16.4, D16.5, D16.6, D16.7, D16.8, D16.9, D28.0, D28.1, D28.7, D29.0, D30.2, D30.20, D30.21, D30.22, D30.4, D30.7, D30.8, D31, D31.0, D31.00, D31.01, D31.02, D31.1, D31.10, D31.11, D31.12, D31.2, D31.20, D31.21, D31.22, D31.3, D31.30, D31.31, D31.32, D31.4, D31.40, D31.41, D31.42, D31.5, D31.50, D31.51, D31.52, D31.6, D31.60, D31.61, D31.62, D31.9, D31.90, D31.91, D31.92, D32, D32.0, D32.1, D32.9, D33, D33.0, D33.1, D33.2, D33.3, D33.4, D33.7, D33.9, D35, D35.0, D35.00, D35.01, D35.02, D35.1, D35.2, D35.3, D35.4, D35.5, D35.6, D35.7, D35.8, D35.9, D36, D36.1, D36.10, D36.11, D36.12, D36.13, D36.14, D36.15, D36.16, D36.17, D36.7, D37.2, D38.2, D38.3, D38.4, D38.5, D39.2, D39.8, D41.2, D41.20, D41.21, D41.22, D41.3, D42, D42.0, D42.1, D42.9, D43, D43.0, D43.1, D43.2, D43.3, D43.4, D43.7, D43.8, D43.9, D44.1, D44.10, D44.11, D44.12, D44.2, D44.3, D44.4, D44.5, D44.6, D44.7, D44.8, D48.0, D48.1, D48.2, D48.3, D48.4, D49.6, D49.81, C49, C49.0, C49.1, C49.10, C49.11, C49.12, C49.2, C49.20, C49.21, C49.22, C49.3, C49.4, C49.5, C49.6, C49.8, C49.9`	227.1, 227.3, 227.4, 227.5, 227.6, 227.8, 227.9, 228, 228.0, 228.00, 228.01, 228.02, 228.03, 228.04, 228.09, 228.1, 228.9, 229.0, 229.8, 230.7, 230.8, 233.31, 233.32, 233.4, 233.5, 234.0, 234.5, 234.8, 235.4, 235.8, 236.1, 236.99, 237, 237.0, 237.1, 237.2, 237.3, 237.5, 237.6, 237.7, 237.70, 237.71, 237.72, 237.73, 237.79, 237.9, 238.0, 238.1, 239.2, 239.6, 171, 171.0, 171.2, 171.3, 171.4, 171.5, 171.6, 171.7, 171.8, 171.9

Abbreviations: ICCC3, International Classification of Childhood Cancer, Third Edition; ICD-9, International Classification of Diseases, Ninth Revision; ICD-10, International Classification of Diseases, Tenth Revision; NA, not applicable.

Footnote: Cancers with NA do not have any relevant codes mapped from ICCC3. Chronic lymphoid leukemia is only modeled for ages 20 years and above in GBD. ICD codes (ICD-9: 204.1, 204.10, 204.11, and 204.12; ICD-10: C91.1, C91.10, C91.11, and C91.12) under 20 years are redistributed (see Section "6. Redistribution" in this appendix for more information) to "Acute lymphoid leukemia," while these ICD codes over 20 years old are mapped directly to "Chronic lymphoid leukemia."

eTable 5: Undefined cancer code categories (ICD-10) and respective target codes for cancer registry incidence data

Unspecified site cancer codes	Target codes for redistribution of these unspecified site cancer
C14, C14.0-14.3, C14.8-14.9	C00-13, C00.0-00.6, C00.8-01.0, C01.9-02.4, C02.8-03.1, C03.9-04.1, C04.8-05.2, C05.8-06.2, C06.8-07.0, C07.9-08.1, C08.8-09.1, C09.8-10.4, C10.8-11.3, C11.8-12.0, C12.9-13.2, C13.8-13.9, D10.0-10.7, D11, D11.0, D11.7, D11.9
C26-29, C26.0-26.2, C26.8-26.9, C35-36	C02.0, C03.0, C04.0, C05.0, C07.0, C15-25, C15.0-15.5, C15.8-17.3, C17.8-19.0, C19.9-20.0, C20.8-21.2, C21.8-22.5, C22.7-22.8, C23.0, C23.9-24.1, C24.4, C24.8-25.4, C25.7-25.9, C30-31, C30.0-30.3, C30.5, C30.8-31.3, C31.8-31.9, C37-38, C37.0-37.3, C38.0-38.4, C38.8, C45, C45.0-45.9, C48, C48.0-48.2, C48.8-48.9, C51-52, C51.0-51.2, C51.8-52.0, C52.9, C57, C57.0-57.4, C57.7-57.8, C60, C60.0-60.2, C60.8-60.9, C63, C63.0-63.2, C63.7-63.8, C66, C66.0-66.2, C66.9, C68.0-68.1, C68.8, C75, C75.0-75.6, C75.8, D00.1-00.2, D01.0-01.3, D07.4, D09.2, D12, D12.0-13.7, D14.0, D15-16, D15.0-15.2, D15.7, D15.9-16.9, D28.0-28.1, D28.7, D29.0, D30.2, D30.4, D30.7-30.8, D31, D31.0-31.6, D31.9, D35-36, D35.0-35.2, D35.5-35.9, D36.1, D36.7, D37.1-37.5, D38.2-38.5, D39.2, D39.8, D41.2-41.3, D44.1-44.8, D48.0-48.4
C39, C39.0, C39.8-39.9	C03.0, C04.0, C05.0, C07.0, C17, C17.0-17.3, C17.8-17.9, C30-34, C30.0-30.3, C30.5, C30.8-31.3, C31.8-32.3, C32.8-33.0, C33.2, C33.9-34.4, C34.7-34.9, C37-38, C37.0-37.3, C38.0-38.4, C38.8, C45, C45.0-45.9, C48, C48.0-48.2, C48.8-48.9, C51-52, C51.0-51.2, C51.8-52.0, C52.9, C57, C57.0-57.4, C57.7-57.8, C60, C60.0-60.2, C60.8-60.9, C63, C63.0-63.2, C63.7-63.8, C66, C66.0-66.2, C66.9, C68.0-68.1, C68.8, C75, C75.0-75.6, C75.8, D02.0-02.3, D07.4, D09.2, D13.2-13.3, D14.0-14.3, D15-16, D15.0-15.2, D15.7, D15.9-16.9, D28.0-28.1, D28.7, D29.0, D30.2, D30.4, D30.7-30.8, D31, D31.0-31.6, D31.9, D35-36, D35.0-35.2, D35.5-35.9, D36.1, D36.7, D37.2, D38.0-38.5, D39.2, D39.8, D41.2-41.3, D44.1-44.8, D48.0-48.4
C42, C76-80, C76.4-76.5, C76.7-76.9, C77.3-77.4, C77.8-77.9, C79.2-80.2, C80.9, C87, C97-99, C97.0, C97.9	C00-13, C00.0-00.6, C00.8-01.0, C01.9-02.4, C02.8-03.1, C03.9-04.1, C04.8-05.2, C05.8-06.2, C06.8-07.0, C07.9-08.1, C08.8-09.1, C09.8-10.4, C10.8-11.3, C11.8-12.0, C12.9-13.2, C13.8-13.9, C15-21, C15.0-15.5, C15.8-17.3, C17.8-19.0, C19.9-20.0, C20.8-21.2, C21.8-21.9, C22.2, C23-25, C23.0, C23.9-24.1, C24.4, C24.8-25.4, C25.7-25.9, C30-34, C30.0-30.3, C30.5, C30.8-31.3, C31.8-32.3, C32.8-33.0, C33.2, C33.9-34.4, C34.7-34.9, C37-38, C37.0-37.3, C38.0-38.4, C38.8, C40-41, C40.0-40.3, C40.8-41.9, C43-44, C43.0-44.9, C47-54, C47.0-47.6, C47.8-48.2, C48.8-49.6, C49.8-51.2, C51.8-52.0, C52.9-53.1, C53.3-53.4, C53.8-54.4, C54.8-54.9, C56-57, C56.0-56.2, C56.4, C56.9-57.4, C57.7-57.8, C60-67, C60.0-60.2, C60.8-61.0, C61.9-62.1, C62.9-63.2, C63.7-63.8, C64.0-64.2, C64.4-64.6, C64.8-65.2, C65.9-66.2, C66.9-68.1, C68.8, C69.0-69.8, C70-73, C70.0-70.1, C70.5-70.6, C70.9-72.5, C72.8-73.5, C73.8-73.9, C75, C75.0-75.6, C75.8, C81-86, C81.0-86.6, C88, C88.0-88.4, C88.7-88.9, C90-96, C90.0-91.0, C91.2-91.3, C91.6, C92.0-92.6, C93.0-93.1, C93.3, C93.8, C94.0-94.8, C95.0-95.2, C95.4, C95.6-95.7, C95.9-96.9, D00.1-00.2, D01.0-01.3, D02.0-02.3, D03-06, D03.0-05.1, D05.7-06.1, D06.7, D06.9-07.2, D07.4-07.5, D09.0, D09.2-09.3, D09.8, D10.0-10.7, D11-12, D11.0, D11.7, D11.9-13.3, D13.5-13.7, D14.0-14.3, D15-16, D15.0-15.2, D15.7, D15.9-16.9, D22-24, D22.0-22.7, D22.9-23.7, D23.9-24.2, D24.9, D26.0-26.1, D26.7, D26.9-27.1, D27, D27.9-28.1, D28.7, D29.0-29.4, D29.7-29.8, D30.0-30.4, D30.7-30.8, D31, D31.0-31.6, D31.9, D34-36, D34.0, D34.9-35.2, D35.5-35.9, D36.1, D36.7, D37.1-37.5, D38.0-38.5, D39.1-39.2, D39.8, D40.0-40.1, D40.7-40.8, D41.0-41.4, D41.7-41.8, D44.0-44.8, D48.0-48.6, D49.2-49.4
C55, C55.0-55.1, C55.9-55.9	C03.0, C04.0, C05.0, C07.0, C17, C17.0-17.3, C17.8-17.9, C30-31, C30.0-30.3, C30.5, C30.8-31.3, C31.8-31.9, C37-38, C37.0-37.3, C38.0-38.4, C38.8, C48, C48.0-48.2, C48.8-48.9, C51-54, C51.0-51.2, C51.8-52.0, C52.9-53.1, C53.3-53.4, C53.8-54.4, C54.8-54.9, C57, C57.0-57.4, C57.7-57.8, C60, C60.0-60.2, C60.8-60.9, C63, C63.0-63.2, C63.7-63.8, C66, C66.0-66.2, C66.9,

	C68.0-68.1, C68.8, C75, C75.0-75.6, C75.8, D06, D06.0-06.1, D06.7, D06.9-07.2, D07.4, D09.2, D13.2-13.3, D14.0, D15-16, D15.0-15.2, D15.7, D15.9-16.9, D26.0-26.1, D26.7, D26.9, D28.0-28.1, D28.7, D29.0, D30.2, D30.4, D30.7-30.8, D31, D31.0-31.6, D31.9, D35-36, D35.0-35.2, D35.5-35.9, D36.1, D36.7, D37.2, D38.2-38.5, D39.2, D39.8, D41.2-41.3, D44.1-44.8, D48.0-48.4
C57.9, C59	C03.0, C04.0, C05.0, C07.0, C17, C17.0-17.3, C17.8-17.9, C30-31, C30.0-30.3, C30.5, C30.8-31.3, C31.8-31.9, C37-38, C37.0-37.3, C38.0-38.4, C38.8, C48, C48.0-48.2, C48.8-48.9, C51-54, C51.0-51.2, C51.8-52.0, C52.9-53.1, C53.3-53.4, C53.8-54.4, C54.8-54.9, C56-57, C56.0-56.2, C56.4, C56.9-57.4, C57.7-57.8, C60, C60.0-60.2, C60.8-60.9, C63, C63.0-63.2, C63.7-63.8, C66, C66.0-66.2, C66.9, C68.0-68.1, C68.8, C75, C75.0-75.6, C75.8, D06, D06.0-06.1, D06.7, D06.9-07.2, D07.4, D09.2, D13.2-13.3, D14.0, D15-16, D15.0-15.2, D15.7, D15.9-16.9, D26.0-26.1, D26.7, D26.9-27.1, D27, D27.9-28.1, D28.7, D29.0, D30.2, D30.4, D30.7-30.8, D31, D31.0-31.6, D31.9, D35-36, D35.0-35.2, D35.5-35.9, D36.1, D36.7, D37.2, D38.2-38.5, D39.1-39.2, D39.8, D41.2-41.3, D44.1-44.8, D48.0-48.4
C63.9	C03.0, C04.0, C05.0, C07.0, C17, C17.0-17.3, C17.8-17.9, C30-31, C30.0-30.3, C30.5, C30.8-31.3, C31.8-31.9, C37-38, C37.0-37.3, C38.0-38.4, C38.8, C48, C48.0-48.2, C48.8-48.9, C51-52, C51.0-51.2, C51.8-52.0, C52.9, C57, C57.0-57.4, C57.7-57.8, C60-63, C60.0-60.2, C60.8-61.0, C61.9-62.1, C62.9-63.2, C63.7-63.8, C66, C66.0-66.2, C66.9, C68.0-68.1, C68.8, C75, C75.0-75.6, C75.8, D07.4-07.5, D09.2, D13.2-13.3, D14.0, D15-16, D15.0-15.2, D15.7, D15.9-16.9, D28.0-28.1, D28.7, D29.0-29.4, D29.7-29.8, D30.2, D30.4, D30.7-30.8, D31, D31.0-31.6, D31.9, D35-36, D35.0-35.2, D35.5-35.9, D36.1, D36.7, D37.2, D38.2-38.5, D39.2, D39.8, D40.0-40.1, D40.7-40.8, D41.2-41.3, D44.1-44.8, D48.0-48.4
C68, C68.9	C03.0, C04.0, C05.0, C07.0, C17, C17.0-17.3, C17.8-17.9, C30-31, C30.0-30.3, C30.5, C30.8-31.3, C31.8-31.9, C37-38, C37.0-37.3, C38.0-38.4, C38.8, C48, C48.0-48.2, C48.8-48.9, C51-52, C51.0-51.2, C51.8-52.0, C52.9, C57, C57.0-57.4, C57.7-57.8, C60, C60.0-60.2, C60.8-60.9, C63-67, C63.0-63.2, C63.7-63.8, C64.0-64.2, C64.4-64.6, C64.8-65.2, C65.9-66.2, C66.9-68.1, C68.8, C75, C75.0-75.6, C75.8, D07.4, D09.0, D09.2, D13.2-13.3, D14.0, D15-16, D15.0-15.2, D15.7, D15.9-16.9, D28.0-28.1, D28.7, D29.0, D30.0-30.4, D30.7-30.8, D31, D31.0-31.6, D31.9, D35-36, D35.0-35.2, D35.5-35.9, D36.1, D36.7, D37.2, D38.2-38.5, D39.2, D39.8, D41.0-41.4, D41.7-41.8, D44.1-44.8, D48.0-48.4, D49.4
C75.9	C03.0, C04.0, C05.0, C07.0, C17, C17.0-17.3, C17.8-17.9, C30-31, C30.0-30.3, C30.5, C30.8-31.3, C31.8-31.9, C37-38, C37.0-37.3, C38.0-38.4, C38.8, C48, C48.0-48.2, C48.8-48.9, C51-52, C51.0-51.2, C51.8-52.0, C52.9, C57, C57.0-57.4, C57.7-57.8, C60, C60.0-60.2, C60.8-60.9, C63, C63.0-63.2, C63.7-63.8, C66, C66.0-66.2, C66.9, C68.0-68.1, C68.8, C73, C73.0-73.5, C73.8-73.9, C75, C75.0-75.6, C75.8, D07.4, D09.2-09.3, D09.8, D13.2-13.3, D14.0, D15-16, D15.0-15.2, D15.7, D15.9-16.9, D28.0-28.1, D28.7, D29.0, D30.2, D30.4, D30.7-30.8, D31, D31.0-31.6, D31.9, D34-36, D34.0, D34.9-35.2, D35.5-35.9, D36.1, D36.7, D37.2, D38.2-38.5, D39.2, D39.8, D41.2-41.3, D44.0-44.8, D48.0-48.4
C76.0-76.1, C77.0-77.1, C78.0-78.3	C00-13, C00.0-00.6, C00.8-01.0, C01.9-02.4, C02.8-03.1, C03.9-04.1, C04.8-05.2, C05.8-06.2, C06.8-07.0, C07.9-08.1, C08.8-09.1, C09.8-10.4, C10.8-11.3, C11.8-12.0, C12.9-13.2, C13.8-13.9, C15, C15.0-15.5, C15.8-15.9, C17, C17.0-17.3, C17.8-17.9, C30-34, C30.0-30.3, C30.5, C30.8-31.3, C31.8-32.3, C32.8-33.0, C33.2, C33.9-34.4, C34.7-34.9, C37-38, C37.0-37.3, C38.0-38.4, C38.8, C43-45, C43.0-45.9, C48, C48.0-48.2, C48.8-48.9, C50-52, C50.0-51.2, C51.8-52.0, C52.9, C57, C57.0-57.4, C57.7-57.8, C60, C60.0-60.2, C60.8-60.9, C63, C63.0-63.2, C63.7-63.8, C66, C66.0-66.2, C66.9, C68.0-68.1, C68.8, C70-73, C70.0-70.1, C70.5-70.6, C70.9-72.5, C72.8-73.5, C73.8-73.9, C75, C75.0-75.6, C75.8, D00.1, D02.0-02.3, D03-05, D03.0-05.1, D05.7-05.9, D07.4, D09.2-09.3, D09.8, D10.0-10.7, D11, D11.0, D11.7, D11.9, D13.0, D13.2-

	13.3, D14.0-14.3, D15-16, D15.0-15.2, D15.7, D15.9-16.9, D22-24, D22.0-22.7, D22.9-23.7, D23.9-24.2, D24.9, D28.0-28.1, D28.7, D29.0, D30.2, D30.4, D30.7-30.8, D31, D31.0-31.6, D31.9, D34-36, D34.0, D34.9-35.2, D35.5-35.9, D36.1, D36.7, D37.2, D38.0-38.5, D39.2, D39.8, D41.2-41.3, D44.0-44.8, D48.0-48.6, D49.2-49.3
C76.2-76.3, C77.2, C77.5, C78.4-78.8, C79.0-79.1	C02.0, C03.0, C04.0, C05.0, C07.0, C15-25, C15.0-15.5, C15.8-17.3, C17.8-19.0, C19.9-20.0, C20.8-21.2, C21.8-22.5, C22.7-22.8, C23.0, C23.9-24.1, C24.4, C24.8-25.4, C25.7-25.9, C30-31, C30.0-30.3, C30.5, C30.8-31.3, C31.8-31.9, C37-38, C37.0-37.3, C38.0-38.4, C38.8, C45, C45.0-45.9, C48, C48.0-48.2, C48.8-48.9, C51-54, C51.0-51.2, C51.8-52.0, C52.9-53.1, C53.3-53.4, C53.8-54.4, C54.8-54.9, C56-57, C56.0-56.2, C56.4, C56.9-57.4, C57.7-57.8, C60-61, C60.0-60.2, C60.8-61.0, C61.9, C63-67, C63.0-63.2, C63.7-63.8, C64.0-64.2, C64.4-64.6, C64.8-65.2, C65.9-66.2, C66.9-68.1, C68.8, C75, C75.0-75.6, C75.8, C82-86, C82.0-86.6, C88, C88.0-88.4, C88.7-88.9, C90, C90.0-90.9, C96, C96.0-96.9, D00.1-00.2, D01.0-01.3, D06, D06.0-06.1, D06.7, D06.9-07.2, D07.4-07.5, D09.0, D09.2, D12, D12.0-13.7, D14.0, D15-16, D15.0-15.2, D15.7, D15.9-16.9, D26.0-26.1, D26.7, D26.9-27.1, D27, D27.9-28.1, D28.7, D29.0-29.1, D30.0-30.4, D30.7-30.8, D31, D31.0-31.6, D31.9, D35-36, D35.0-35.2, D35.5-35.9, D36.1, D36.7, D37.1-37.5, D38.2-38.5, D39.1-39.2, D39.8, D40.0, D41.0-41.4, D41.7-41.8, D44.1-44.8, D48.0-48.4, D49.4

eTable 6: Cancer registry sources for cancer incidence and mortality-to-incidence ratio data by country, year, and registry

Location	Registry	Years available from registry	Years used for incidence	Years available for MIR	Years used for MIR
Algeria	Algiers	1993-1997	5	1993-1997	0
Algeria	Batna	2000-2006, 2008-2012	12	2000-2006, 2008-2012	0
Algeria	Oran	2005-2006	2	2005-2006	0
Algeria	Setif	1986-1993, 1998-2011	22	1986-1993, 1998-2011	0
Argentina	6 Registries Combined (Bahia Blanca, Chaco, Cordoba, Entre Rios, Mendoza, Neuquen)	1991-2013	23	1991-2013	0
Argentina	Concordia	1990-1997	8	1990-1997	0
Argentina	Cordoba	2004-2012	9	2004-2012	0
Argentina	Mendoza	2003-2014	10	2003-2012	0
Argentina	Tierra del Fuego	2003-2012	10	2003-2012	0
Argentina	Chaco	2008-2012	5	2008-2012	0
Argentina	Entre Rios Province	2008-2011	4	2008-2011	0
Argentina	Bahia Blanca	1993-2007	15	1993-2007	0
Australia	Victoria	1983-2012	30	1983-2012	0
Australia	Western Australia	1983-2012	30	1983-2012	0
Australia	Queensland	1982-2015	20	1993-2012	0
Australia	National Registry	1982-2014	33	1968-2014	26
Australia	New South Wales	1983-2012	30	1983-2012	0
Australia	Capital Territory	1983-2007	25	1983-2007	0
Australia	Tasmania	1978-2012	35	1978-2012	0
Australia	Northern Territory	1998-2012	15	1998-2012	0
Australia	South Australia	1977-2012	36	1977-2012	0
Austria	Carinthia	2008-2012	5	2008-2012	0
Austria	Vorarlberg	1993-2012	20	1993-2012	0
Austria	Tyrol	1988-2012	25	1988-2012	0
Austria	National Registry	1983-2012	30	1983-2012	7
Bahrain	National Registry	1998-2012	15	1998-2012	0
Belarus	National Registry	1983-2012	30	1983-2012	0
Belarus	Belarus Paediatric	1990-2014	25	1990-2014	0
Belgium	Antwerp	1998-2002	5	1998-2002	0
Belgium	Flanders	1998-2001	4	1998-2001	0
Belgium	Flanders except Limburg	1997-1998	2	1997-1998	0
Belgium	Limburg	1997-1998	2	1997-1998	0
Belgium	National Registry	2003-2013	11	2003-2013	0

Bermuda	Bermuda	1975-2018	44	1975-2018	1
Botswana	National Registry	1999-2008	10	1999-2008	0
Brazil	Belem	1989-1991, 1996-2012	20	1989-1991, 1996-2012	0
Brazil	Santos	2008-2009	2	2008-2009	0
Brazil	Sao Paulo	1969, 1973, 1978, 1997-2013	20	1969, 1973, 1978, 1997-2013	0
Brazil	Barretos	2008-2013	6	2008-2013	0
Brazil	Palmas	2000-2012	13	2000-2012	0
Brazil	Aracaju	1996-2013	18	1996-2013	0
Brazil	Florianopolis	2008-2012	5	2008-2012	0
Brazil	Porto Alegre	1979-1982, 1987, 1990-2006	22	1979-1982, 1987, 1990-2006	0
Brazil	Roraima	2003-2010	8	2003-2010	0
Brazil	Natal	1999-2005	7	1999-2005	0
Brazil	Angra Dos Reis	2007-2008	2	2007-2008	0
Brazil	Teresina	2000-2006	7	2000-2006	0
Brazil	Recife	1968-1971, 1980, 1995-2012	23	1968-1971, 1980, 1995-2012	0
Brazil	Curitiba	1998-2012	15	1998-2012	0
Brazil	Joao Pessoa	1999-2012	14	1999-2012	0
Brazil	Cuiaba	2000-2009	10	2000-2009	0
Brazil	Mato Grosso Interior	2001-2005	5	2001-2005	0
Brazil	Campo Grande	2000-2005, 2008-2010	7	2000-2003, 2008-2010	0
Brazil	Pocos de Caldas	2007-2011	5	2007-2011	0
Brazil	Belo Horizonte	2000-2011	12	2000-2011	0
Brazil	Goiania	1988-2012	25	1988-2012	0
Brazil	Espirito Santo	1997-2012	16	1997-2012	0
Brazil	Brasilia	1998-2001	4	1998-2001	0
Brazil	Distrito Federal	1999-2002	4	1999-2002	0
Brazil	Fortaleza	1978-1982, 1990-2009	25	1978-1982, 1990-2009	0
Brazil	Salvador	1996-2005	10	1996-2005	0
Brazil	Manaus	1999-2009	11	1999-2009	0
Brazil	Jahu	1996-2015	20	1996-2015	0
Brazil	Campinas	1991-2005	15	1991-2005	0
Brunei Darussalam	National Registry	2010-2012	3	2010-2012	0
Bulgaria	National Registry	1993-2012	20	1993-2012	12
Cameroon	Yaounde	2004-2006	3	2004-2006	0

Canada	Nunavut	2008-2012	5	2008-2012	0
Canada	Yukon	1983-1997, 2003-2012	25	1983-1997, 2003-2012	0
Canada	Saskatchewan	1960-1966, 1968-2014	54	1960-1966, 1968-2014	7
Canada	Quebec	1963-1966, 1969-1997, 2003-2007	38	1963-1966, 1969-1997, 2003-2007	0
Canada	Prince Edward Island	1978-2012	35	1978-2012	0
Canada	Ontario	1969-1971, 1978-2012	38	1969-1971, 1978-2012	0
Canada	Newfoundland	1969-2002	34	1969-2002	0
Canada	Nova Scotia	1978-2012	35	1978-2012	0
Canada	Northwest Territories	1983-2012	30	1983-2012	0
Canada	National Registry	1978-2007	30	1978-2007	0
Canada	Maritime	1969-1987	19	1969-1987	0
Canada	New Brunswick	1962-1966, 1978-2012	40	1962-1966, 1978-2012	0
Canada	Newfoundland and Labrador	1960-1966, 1969-2012	51	1960-1966, 1969-2012	0
Canada	Manitoba	1958-2012	55	1958-2012	0
Canada	Northwest Territories and Yukon	1973-1987	15	1973-1987	0
Canada	British Columbia	1969-2013	45	1969-2013	0
Canada	Alberta	1960-1966, 1969-2012	51	1960-1966, 1969-2012	0
Chile	Los Rios	2003-2007	5	2003-2007	0
Chile	Bio Bio	2003-2012	10	2003-2012	0
Chile	Antofagasta	2003-2010	8	2003-2010	0
Chile	National Registry	1959-1961, 2007-2011	8	1959-1961, 2007-2011	1
Chile	Concepcion	2008-2010	3	2008-2010	0
Chile	Valdivia	1998-2012	15	1998-2012	0
China	Zhongshan County	2004, 2006-2007	3	2004, 2006-2007	0
China	Beijing	1990-2013	23	1990-2012	20
China	Chuzhou District, Huai'an	2004, 2006-2007	3	2004, 2006-2007	0
China	Hefei	2010-2013	3	2010-2012	0
China	Ci County	1990-2012	23	1990-2012	12
China	Qianxi County	2009, 2011, 2013	1	2009	1
China	Shexian County	2003-2013	10	2003-2012	6
China	Tongling	2008-2013	5	2008-2012	2

China	Ma'anshan	2003-2013	10	2003-2012	7
China	Jiulongpo District, Chongqing	2004, 2007, 2009, 2011, 2013	3	2004, 2007, 2009	3
China	Changle	1990-2009, 2011, 2013	20	1990-2009	19
China	Sihui	1998-2009, 2011, 2013	12	1998-2009	12
China	Xiamen City	2009, 2011	1	2009	1
China	Jingtai County	2009, 2011, 2013	1	2009	0
China	Liangzhou District	2008-2009, 2011, 2013	2	2008-2009	2
China	Wuwei	2004	1	2004	0
China	Guangzhou City	2000-2013	13	2000-2012	10
China	Hong Kong Special Administrative Region of China	1974-2013	40	1974-2013	11
China	Baoding	2009, 2011, 2013	1	2009	1
China	Liuzhou	2009-2013	4	2009-2012	1
China	Fusui County	1990-1997, 2003-2009, 2011, 2013	15	1990-1997, 2003-2009	7
China	Zhuhai	2010-2012	3	2010-2012	0
China	Jiangmen	2010-2013	3	2010-2012	0
China	Zhongshan	1998-2013	15	1998-2012	12
China	Shenzen City	2004, 2011, 2013	1	2004	0
China	Feixi County	2009, 2011, 2013	1	2009	1
China	Daoli District, Harbin City	2005-2009, 2011, 2013	5	2005-2009	5
China	Hai'an County	2009, 2011, 2013	1	2009	1
China	Haimen	2003-2013	10	2003-2012	7
China	Huai'an District, Huai'an	1998-2009, 2013	12	1998-2009	12
China	Huaiyin District, Huai'an	2009-2013	4	2009-2012	1
China	Zhanggong District	2009	1	2009	1
China	Dehui	2009, 2011, 2013	1	2009	1
China	Yanji	2009, 2011, 2013	1	2009	1
China	Hengdong County	2009-2012	4	2009-2012	1

China	Anshan	1998-2013	15	1998-2012	12
China	Dalian City	1998-2009, 2011	12	1998-2009	12
China	Dandong	2008-2009, 2011, 2013	2	2008-2009	2
China	Donggang	2009, 2011	1	2009	1
China	Shenyang City	2003-2012	10	2003-2012	7
China	Zhuanghe	2009, 2011, 2013	1	2009	1
China	Yunmeng County	2009, 2011, 2013	1	2009	1
China	Benxi	2003-2011, 2013	9	2003-2011	7
China	Xining	2009, 2011, 2013	1	2009	1
China	Guanyun County	2004, 2007-2013	7	2004, 2007-2012	2
China	Donghai County	2004, 2009, 2011, 2013	2	2004, 2009	1
China	Suzhou	2006-2009, 2011, 2013	4	2006-2009	4
China	Sheyang County	2008-2013	5	2008-2012	2
China	Qidong County	1990-2009, 2011	20	1990-2009	20
China	Lianyungang	2004, 2007-2013	7	2004, 2007-2012	3
China	Jintan District	2003-2007, 2009, 2011, 2013	6	2003-2007, 2009	6
China	Jinhu County	2007-2009, 2011, 2013	3	2007-2009	3
China	Ganyu	2004, 2011, 2013	1	2004	0
China	Jianhu County	2003-2013	10	2003-2012	7
China	Yueyanglou	2009-2012	4	2009-2012	0
China	Qidong County	1983-1997, 2003-2012	25	1983-1997, 2003-2012	0
China	Wuxi	2006, 2010-2013	4	2006, 2010-2012	1
China	Dafeng	2003-2009, 2011, 2013	7	2003-2009	7
China	Xuyi County	2009, 2011, 2013	1	2009	1
China	Yangzhong	1998-2009, 2011, 2013	12	1998-2009	12

China	Chifeng	2009, 2011	1	2009	1
China	Feicheng	1998-2009, 2011, 2013	12	1998-2009	12
China	Changle	2004, 2006-2007	3	2004, 2006-2007	0
China	Wuhan City	1990-2012	23	1990-2012	17
China	Nangang District, Harbin City	1992-2013	21	1992-2012	13
China	Xianju County	2009-2012	4	2009-2012	1
China	Shangyu	2009, 2011	1	2009	1
China	Jiaxing	2000-2013	13	2000-2012	10
China	Jiashan County	1990-2013	23	1990-2012	20
China	Hangzhou City	2000-2013	13	2000-2012	10
China	Haining	1998-2009, 2011, 2013	12	1998-2009	12
China	Shangzhi	2009, 2011, 2013	1	2009	1
China	Linzhou	1990-2013	23	1990-2012	20
China	Xiping County	2009-2013	4	2009-2012	1
China	Yanshi	2009-2013	4	2009-2012	1
China	Linqu County	1998-2009, 2011	12	1998-2009	12
China	Taixing	2004-2005, 2007-2009, 2011, 2013	5	2004-2005, 2007-2009	5
China	Tianjin	1981-2004, 2011, 2013	24	1981-2004	15
China	Qingyang District, Chengdu	2009, 2011, 2013	1	2009	1
China	Shanghai	1975, 1978-2013	36	1975, 1978-2012	20
China	Yanting County	1998-2013	15	1998-2012	12
China	Wenshang County	2009, 2011	1	2009	1
China	Ziliujing District	2009	1	2009	1
China	Yangquan	2009, 2011, 2013	1	2009	1
China	Yangcheng County	2003-2009, 2011, 2013	7	2003-2009	7
China	Gejiu	2004, 2008, 2011, 2013	2	2004, 2008	1
China	Kunes County	2009	1	2009	1
Colombia	4 Registries Combined (Bucaramanga, Cali, Manizales, Pasto)	1992-2013	22	1992-2013	0
Colombia	Pasto	2003-2012	10	2003-2012	0

Colombia	National Registry	2003-2010	8	2003-2010	0
Colombia	Bucaramanga	2003-2012	10	2003-2012	0
Colombia	Cali	1962-2012	51	1962-2012	0
Colombia	Manizales	2003-2012	10	2003-2012	0
Costa Rica	National Registry	1980-2012	33	1980-2013	0
Croatia	National Registry	1988-1991, 1993-2014	26	1988-1991, 1993-2014	8
Cuba	National Registry	1968-1977, 1986, 2000-2012	24	1968-1977, 1986, 2000-2012	0
Cuba	Villa Clara	1995-1997, 2004-2007	7	1995-1997, 2004-2007	0
Cyprus	National Registry	1998-2012	15	1998-2012	4
Cyprus	South West	1998-2012	15	1998-2012	0
Czechia	National Registry	1980-2012	30	1983-2012	5
Denmark	National Registry	1953-2014	62	1953-2014	35
Ecuador	Cuenca	2003-2007	5	2003-2007	0
Ecuador	Quito	1985-2012	28	1985-2012	0
Ecuador	Guayaquil	2008-2012	5	2008-2012	0
Ecuador	Loja	2008-2010	3	2008-2010	0
Ecuador	Manabi	2008-2012	5	2008-2012	0
Ecuador	5 Registries Combined (Cuenca, Guayaquil, Loja, Manabi, Quito)	1993-2013	21	1993-2013	0
Egypt	Aswan	2008	1	2008	0
Egypt	Damietta	2009	1	2009	0
Egypt	Gharbiah	1999-2007	9	1999-2007	0
Egypt	Minia	2009	1	2009	0
Estonia	National Registry	1968-2012	45	1968-2012	5
Ethiopia	Addis Ababa	2011-2016	6	2011-2016	0
Faroe Islands	Faroe Islands	1960-2005	46	1960-2006	22
Fiji	National Registry	1998-2008	11	1998-2008	11
Finland	National Registry	1953-2014	62	1953-2014	35
France	Herault	1986-2012	26	1987-2012	0
France	Somme	1982-2012	31	1982-2012	0
France	Doubs	1977-2012	36	1977-2012	0
France	Calvados	1978-2012	35	1978-2012	0
France	France Paediatric	2000-2012	13	2000-2012	0
France	13 Registries Combined (Bas-Rhin, Calvados, Doubs, Gironde, Haut-Rhin, Herault, Isere, Lille, Limousin, Loire-	1993-2012	20	1993-2012	0

	Atlantique and Vendee, Manche, Somme, Tarn)				
France	Territoire de Belfort	2008-2012	5	2008-2012	0
France	Limousin	2009-2012	4	2009-2012	0
France	Bas Rhin	1975-2011	37	1975-2011	0
France	Isere	1979-2012	34	1979-2012	0
France	Tarn	1982-2012	31	1982-2012	0
France	Vendee	1998-2012	15	1998-2012	0
France	Lille	2008-2012	5	2008-2012	0
France	Manche	1994-2011	18	1994-2011	0
France	Nord	2005, 2008-2009	3	2005, 2008-2009	0
France	Gironde	2008-2012	5	2008-2012	0
France	Haut Rhin	1988-2012	25	1988-2012	0
France	Loire Atlantique	1997-2012	16	1997-2012	0
French Guiana	National Registry	2008-2012	5	2008-2012	0
French Polynesia	French Polynesia	1998-2002	5	1998-2002	0
Gambia	National Registry	1987-1998, 2002-2011	22	1987-1998, 2002-2011	0
Germany	Saxony-Anhalt	1998-2007	10	1998-2007	0
Germany	Rhineland Palatinate	2000-2012	13	2000-2012	0
Germany	Saarland	1968-2012	45	1968-2012	30
Germany	Schleswig Holstein	1998-2012	15	1998-2012	2
Germany	Thuringen	1998-2007	10	1998-2007	0
Germany	Bavaria	2002-2012	11	2002-2012	0
Germany	Westphalia	1998-2012	15	1998-2012	0
Germany	Mecklenburg	1998-2007	10	1998-2007	0
Germany	Munich	1998-2012	15	1998-2012	0
Germany	North Rhine Westphalia	1998-2007	10	1994-2007	2
Germany	5 Western Registries Combined (Bavaria, Lower Saxony, Rhine-Pfalz, Saarland, Schleswig-Holstein)	1994-2012	19	1994-2012	0
Germany	Mecklenburg-West Pomerania	1998-2007	10	1998-2007	0
Germany	Germany Paediatric	1980-1990, 1996-2012	17	1996-2012	0
Germany	Berlin	1998-2007	10	1998-2007	0
Germany	Brandenburg	1998-2007	10	1998-2007	0
Germany	Bremen	2000-2012	13	2000-2012	0
Germany	Eastern States (former GDR)	1964-1966, 1968-1989	25	1964-1966, 1968-1989	0
Germany	Free State of Saxony	1998-2007	10	1998-2007	0

Germany	Hamburg	1969-1979, 1995-2012	29	1969-1979, 1995-2012	2
Germany	Lower Saxony	2003-2012	10	2003-2012	0
Germany	National Registry	1991-2014	11	2000-2010	11
Greenland	Greenland	1980-2014	35	1980-2014	32
Grenada	St. George's Central Hospital	1996-2000	5	1996-2000	0
Guinea	Conakry	1992-1995, 2001-2010	14	1992-1995, 2001-2010	0
Hungary	National Registry	1977-1999, 2001-2011	11	2001-2011	0
Hungary	County Szabolcs-Szatmar	1962-1966, 1969-1971, 1973-1987	23	1962-1966, 1969-1971, 1973-1987	0
Hungary	County Vas	1962-1966, 1968-1987	25	1962-1966, 1968-1987	0
Hungary	Miskolc	1962-1966	5	1962-1966	0
Iceland	National Registry	1955-2014	60	1955-2014	35
India	Wardha	2010-2014	5		0
India	Karunagappally	1991-2007	24		0
India	Kollam	2006-2014	9		0
India	Tamil Nadu	2012-2013	2		0
India	Tripura	2010-2014	5		0
India	Kamrup Urban	2005-2014	10		0
India	Silchar	2005-2006	5		0
India	Delhi	1990-1996, 1998-2009, 2012	17		0
India	Ahmedabad Urban	2009-2010, 2012-2013	18		0
India	Bangalore	1982-2012	31		0
India	Kerala, Urban		22		0
India	Bhopal	1990-1996, 2004-2013	10		0
India	Barshi Rural	1988-1992, 2003-2014	17		0
India	Barshi Expanded	1990-1996, 2009, 2012	2		0
India	Mumbai	1964-1966, 1968-1975, 1978-2012	43		0
India	Nagpur	1980-1982, 1993-2002, 2005-2009, 2012-2013	20		0

India	Pune	1973-1982, 1993-2013	31		0
India	Manipur Excl Imphal West	2009-2010, 2012-2014	7		0
India	Aurangabad	2005-2010, 2012-2014	9		0
India	S.A.S Nagar District	2013-2014	2		0
India	Patiala District	2012-2014	3		0
India	Ahmedabad Rural	2009-2010	16		0
India	Kolkata	2005-2009, 2012	6		0
India	Chennai	1982-2013	32		0
India	Chandigarh Union Territory	2013-2014	2		0
India	Pasighat	2012-2014	3		0
India	Naharlagun Excl Papum Pare	2012-2014	3		0
India	Papum Pare	2012-2014	3		0
India	Cachar	2007-2014	8		0
India	Sikkim	2003-2014	12		0
India	Ahmedabad	1983-1987, 1993-1997, 2004-2005, 2008-2011	16		0
India	Trivandrum	1991-2002, 2005-2014	22		0
India	Dibrugarh	2005-2014	10		0
India	Imphal	2005-2006, 2009-2010, 2012-2014	5		0
India	Aizawl	2005-2008, 2012-2014	6		0
India	Sangrur District	2013-2014	2		0
India	Manipur	2006-2010	5		0
India	Mizoram Excl Aizawl	2005-2006, 2012-2014	5		0
India	Mansa District	2013-2014	2		0
India	Nagaland	2010, 2012-2014	4		0
India	Mizoram	2003-2012	10		0
India	Meghalaya	2010-2014	5		0
India	Dindigul Ambilikkai	2003-2013	11		0
Iran (Islamic Republic of)	Bushehr	2001-2010	10	2001-2010	0
Iran (Islamic Republic of)	Alborz	2002-2010	9	2002-2010	0

Iran (Islamic Republic of)	Khorasan-e-Razavi	2000-2010	11	2000-2010	0
Iran (Islamic Republic of)	West Azarbayejan	2000-2010	11	2000-2010	0
Iran (Islamic Republic of)	East Azarbayejan	2000-2010	11	2000-2010	0
Iran (Islamic Republic of)	Ardebil	2000-2010	11	2000-2010	0
Iran (Islamic Republic of)	Golestan	1996-2000, 2004-2011	12	1996-2000, 2005-2011	0
Iran (Islamic Republic of)	North Khorasan	2000, 2002-2010	10	2000, 2002-2010	0
Iran (Islamic Republic of)	National Registry	2003-2007	5	2003-2007	0
Iran (Islamic Republic of)	South Khorasan	2001-2010	10	2001-2010	0
Iran (Islamic Republic of)	Khuzestan	2000-2010	11	2000-2010	0
Iran (Islamic Republic of)	Kohgiluyeh and Boyer-Ahmad	2000-2010	11	2000-2010	0
Iran (Islamic Republic of)	Kurdistan	2000-2010	11	2000-2010	0
Iran (Islamic Republic of)	Lorestan	2000-2010	11	2000-2010	0
Iran (Islamic Republic of)	Markazi	2001-2010	10	2001-2010	0
Iran (Islamic Republic of)	Mazandaran	2000-2010	11	2000-2010	0
Iran (Islamic Republic of)	Ardabil	1985, 1996-1999, 2006-2008	8	1985, 1996-1999, 2006-2008	0
Iran (Islamic Republic of)	Qom	2000-2010	11	2000-2010	0
Iran (Islamic Republic of)	Semnan	2000-2010	11	2000-2010	0
Iran (Islamic Republic of)	Sistan and Baluchistan	2001-2010	10	2001-2010	0
Iran (Islamic Republic of)	Tehran	2000-2010	11	2000-2010	0
Iran (Islamic Republic of)	Yazd	2000-2010	11	2000-2010	0
Iran (Islamic Republic of)	Zanjan	2000-2010	11	2000-2010	0
Iran (Islamic Republic of)	Qazvin	2000-2010	11	2000-2010	0
Iran (Islamic Republic of)	Kermanshah	2000-2010	11	2000-2010	0
Iran (Islamic Republic of)	Kerman	2000-2010	11	2000-2010	0
Iran (Islamic Republic of)	Isfahan	2000-2010	11	2000-2010	0
Iran (Islamic Republic of)	Ilam	2000-2010	11	2000-2010	0
Iran (Islamic Republic of)	Hormozgan	2001-2010	10	2001-2010	0
Iran (Islamic Republic of)	Hamadan	2000-2010	11	2000-2010	0
Iran (Islamic Republic of)	Golestan	2000-2010	11	2000-2010	0
Iran (Islamic Republic of)	Gilan	2000-2010	11	2000-2010	0
Iran (Islamic Republic of)	Fars	2000-2010	11	2000-2010	0
Iran (Islamic Republic of)	Chahar Mahaal and Bakhtiari	2000-2010	11	2000-2010	0
Iraq	National Registry	2007, 2009, 2011	3	2007, 2009, 2011	0
Ireland	National Registry	1994-2012	19	1994-2012	0
Ireland	Ireland (Southern)	1980-1986, 1988-1992	12	1980-1986, 1988-1992	0

Israel	National Registry	1960-1963, 1990-2012	27	1960-1963, 1990-2012	0
Italy	Romagna	1986-2002	17	1986-2002	0
Italy	Ragusa	1981-2002	22	1981-2002	0
Italy	Syracuse	1999-2002	4	1999-2002	0
Italy	Salerno	1998-2001	4	1998-2001	0
Italy	Naples	1998-2002	5	1998-2002	0
Italy	Latina	1983-1985, 1988-1991	7	1983-1985, 1988-1991	0
Italy	Macerata	1991-2000	10	1991-2000	0
Italy	Umbria	1994-1996, 1998-2006	12	1994-1996, 1998-2006	1
Italy	Florence	1985-2002	18	1985-2002	0
Italy	Florence and Prato	1985-2002	18	1985-2002	0
Italy	Ferrara	1991-2002	12	1991-2002	0
Italy	Sassari	1993-2002	10	1993-2002	0
Italy	Reggio Emilia	1998-2002	5	1998-2002	0
Italy	North East Italy	1995-2002	8	1995-2002	0
Italy	Modena	1988-2007	20	1988-2007	0
Italy	Trieste	1984-1985, 1989-1992	6	1984-1985, 1989-1992	0
Italy	Veneto	1988-1996, 1998-2001	13	1988-1996, 1998-2001	0
Italy	Brescia	1999-2001	3	1999-2001	0
Italy	Varese	1976-2000	25	1976-2000	0
Italy	Sondrio	1998-2007	10	1998-2007	0
Italy	Milan	1999-2002	4	1999-2002	0
Italy	Genoa	1986-1996, 1998-2000	14	1986-1996, 1998-2000	0
Italy	Torino	1985-2002	18	1985-2002	0
Italy	Biella	1995-2002	8	1995-2002	0
Italy	National Registry	1986-2013	28	1986-2013	4
Italy	Parma	1978-2002	25	1978-2002	0
Jamaica	National Registry	1978-1982, 1988-1997	15	1978-1982, 1988-1997	0
Jamaica	Kingston and St Andrew	1958-1977, 2003-2011	29	1958-1977, 2003-2011	0
Japan	Tochigi	2008-2012	5	2008-2012	0
Japan	Nagasaki	1973-2012	40	1973-2012	0
Japan	Saga	1984-1986, 1988-1997, 2003-2007	18	1984-1986, 1988-1997, 2003-2007	0
Japan	Fukuoka	1974-1975	2	1974-1975	0

Japan	Hiroshima	1978-2000, 2003-2012	33	1978-2000, 2003-2012	0
Japan	Okayama	1966, 1969	2	1966, 1969	0
Japan	Osaka	1963-2002, 2008-2012	45	1963-2002, 2008-2012	0
Japan	Aichi	1998-2012	15	1998-2012	1
Japan	Fukui	1998-2012	15	1998-2012	0
Japan	Niigata	2003-2012	10	2003-2012	0
Japan	Miyagi	1959-1960, 1962-1964, 1968-1971, 1973-2010	47	1959-1960, 1962-1964, 1968-1971, 1973-2010	0
Japan	Yamagata	1983-2002, 2008-2012	25	1983-2002, 2008-2012	0
Japan	National Registry	1975-2010	36	1958-2013	36
Jordan	National Registry	1996-1998, 2001-2008	8	2001-2008	0
Kenya	Eldoret	2000-2011	12	2000-2011	0
Kenya	Nairobi	2000-2002, 2004-2012	12	2000-2002, 2004-2012	0
Kuwait	National Registry	1979-2012	34	1979-2012	0
Kyrgyzstan	National Registry	1986-1987	2	1986-1987	0
La Martinique	La Martinique	1981-1987, 1993-2002, 2008-2012	22	1981-1987, 1993-2002, 2008-2012	0
La Reunion	La Reunion	1988-1994, 2002-2011	17	1988-1994, 2002-2011	0
Latvia	National Registry	1983-2007, 2010-2012	28	1983-2007, 2010-2012	5
Lebanon	National Registry	1998, 2005-2007	4	1998, 2005-2007	0
Libya	Benghazi	2003-2005	3	2003-2005	0
Lithuania	National Registry	1978-2012	35	1978-2012	3
Malawi	Blantyre	1994-1998, 2003-2010	13	1994-1998, 2003-2010	0
Malaysia	National Registry	2003	1	2003	0
Malaysia	Penang	1998-2002, 2004-2010	12	1998-2002, 2004-2010	0
Malaysia	Sarawak	1998-2002	5	1998-2002	0
Mali	Bamako	1987-1992, 1994-1996, 2005-2014	19	1987-1992, 1994-1996, 2005-2014	0
Malta	National Registry	1969-1972, 1992-2012	25	1969-1972, 1992-2012	16
Mauritius	National Registry	2003-2012	10	2003-2012	0

Mongolia	National Registry	2003-2007	5	2003-2007	0
Morocco	2 Registries Combined (Casablanca, Rabat)	2005-2012	8	2005-2012	0
Morocco	Greater Casablanca	2004	1	2004	0
Mozambique	Lourenco Marques	1956-1960	5	1956-1960	0
Namibia	National Registry	2000-2014	15	2000-2014	0
Netherland Antilles excluding Aruba	Antilles except Aruba	1973-1982	10	1973-1982	0
Netherlands	Eindhoven	1973-2007	35	1973-2007	0
Netherlands	Maastricht	1986-2002	17	1986-2002	0
Netherlands	Three Provinces Combined (Eindhoven, Maastricht, Antilles)	1960-1962	3	1960-1962	0
Netherlands	National Registry	1989-2018	25	1989-2013	0
New Zealand	New Zealand non-Maori population	1993-2014	22	1993-2014	19
New Zealand	New Zealand Maori population	1995-2014	20	1995-2014	19
New Zealand	National Registry	1983-2015	33	1983-2015	5
Niger	Niamey	2001-2009	9	2001-2009	0
Nigeria	Calabar	2009-2013	5	2009-2013	0
Nigeria	Ibadan	1960-1969, 2003-2012	20	1960-1969, 2003-2012	0
Norway	Buskerud	1953-2015	63	1953-2015	36
Norway	Nordland	1953-2015	63	1953-2015	36
Norway	Troms	1953-2015	63	1953-2015	36
Norway	Finmark	1953-2015	63	1953-2015	36
Norway	Trondelag	1953-2015	63	1953-2015	36
Norway	Ostfold	1953-2015	63	1953-2015	36
Norway	Oppland	1953-2015	63	1953-2015	36
Norway	Vestfold	1953-2015	63	1953-2015	36
Norway	Hordaland	1953-2015	63	1953-2015	36
Norway	Rogaland	1953-2015	63	1953-2015	36
Norway	Hedemark	1953-2015	63	1953-2015	36
Norway	Akershus	1953-2015	63	1953-2015	36
Norway	Oslo	1953-2015	63	1953-2015	36
Norway	Vest-Agder	1953-2015	63	1953-2015	36
Norway	Aust-Agder	1953-2015	63	1953-2015	36
Norway	National Registry	1953-2014	62	1953-2014	35
Norway	Telemark	1953-2015	63	1953-2015	36
Norway	More og Romsdal	1953-2015	63	1953-2015	36
Norway	Sogn og Fjordane	1953-2015	63	1953-2015	36
Oman	National Registry	1993-2013	21	1993-2013	0

Pakistan	Karachi	1995-2002	8	1995-2002	0
Pakistan	South Karachi	1995-2002	8	1995-2002	0
Palestine	West Bank	2010-2011	2	2010-2011	0
Panama	National Registry	1988-1999, 2001-2011	23	1988-2011	12
Paraguay	Asuncion Region	1988-1989	2	1988-1989	0
Peru	Trujillo	1984-1990, 1996-2002	14	1984-1990, 1996-2002	1
Peru	Lima	1990-1991, 2010-2012	5	1990-1991, 2010-2012	0
Philippines	Rizal	1978-1987, 1993-1997	15	1978-1987, 1993-1997	0
Philippines	Manila	1983-2002	20	1983-2002	0
Poland	Cieszyn and Nowy Sacz	1968-1972	5	1968-1972	0
Poland	Katowice	1965-1966, 1970-1974	7	1965-1966, 1970-1974	0
Poland	Cieszyn	1973-1977	5	1973-1977	0
Poland	Opole	1985-1987	3	1985-1987	0
Poland	Warsaw Urban	1965-1966, 1968-1977, 1980-2002	35	1965-1966, 1968-1977, 1980-2002	0
Poland	Warsaw Rural	1968-1977, 1983-1987	15	1968-1977, 1983-1987	0
Poland	Warsaw	1988-2002	15	1988-2002	0
Poland	Cracow	1973-2002	30	1973-2002	0
Poland	Nowy Sacz	1973-1981, 1983-1986	13	1973-1981, 1983-1986	0
Poland	Four Rural Areas	1965-1966	2	1965-1966	0
Poland	Cracow City and District	1965-1966, 1968-1972	7	1965-1966, 1968-1972	0
Poland	National Registry	1999-2011	13	1999-2011	13
Poland	Kielce	1988-1996, 1998-2002	14	1988-1996, 1998-2002	0
Poland	Lower Silesia	1984-1997	14	1984-1997	0
Portugal	South Portugal	1998-2007	10	1998-2007	0
Portugal	Vila Nova de Gaia	1983-1987, 1993-1997	10	1983-1987, 1993-1997	0
Portugal	Porto	1998-2002	5	1998-2002	0
Portugal	North Portugal	2000-2006	7	2000-2006	7
Portugal	Centre	2003-2007	5	2003-2007	5
Portugal	Azores	1997-2011	15	1981-2012	15
Portugal	National Registry	1991-2012	22	1991-2012	0
Qatar	National Registry	2003-2012	10	2003-2012	0
Republic of Korea	Daejeon	1998-2012	15	1998-2012	0

Republic of Korea	Busan	1996-2012	17	1996-2012	0
Republic of Korea	Daegu	1997-2012	16	1997-2012	0
Republic of Korea	Gwangju	1998-2012	15	1998-2012	0
Republic of Korea	Incheon	1998-2012	15	1998-2012	0
Republic of Korea	Jejudo	2000-2002, 2004-2012	12	2000-2002, 2004-2012	0
Republic of Korea	National Registry	1999-2012	14	1999-2012	0
Republic of Korea	Seoul	1993-2012	20	1993-2012	0
Republic of Korea	Ulsan	1999-2012	14	1999-2012	0
Republic of Korea	Kangwha County	1986-1997	12	1986-1997	0
Romania	Timisoara	2008	1	2008	0
Romania	County Cluj	1974-1987	14	1974-1987	0
Romania	Cluj	2007	1	2007	1
Romania	County Timis	1970-1972	3	1970-1972	0
Romania	Banat Region	1967	1	1967	0
Russian Federation	Chukchi autonomous area	2007-2008, 2010-2015	8	2007-2016	7
Russian Federation	Ulyanovsk oblast	2007-2016	10	2007-2016	10
Russian Federation	Saratov oblast	2007-2016	10	2007-2016	10
Russian Federation	Samara oblast	2007-2016	10	2007-2016	10
Russian Federation	Penza oblast	2007-2016	10	2007-2016	10
Russian Federation	Orenburg oblast	2007-2016	10	2007-2016	10
Russian Federation	Nizhny Novgorod oblast	2007-2016	10	2007-2016	10
Russian Federation	Kurgan oblast	2007-2016	10	2007-2016	10
Russian Federation	Kirov oblast	2007-2016	10	2007-2016	10
Russian Federation	Kemerovo oblast	2007-2016	10	2007-2016	10
Russian Federation	Irkutsk oblast	2007-2016	10	2007-2016	10
Russian Federation	Krasnoyarsk kray	2007-2016	10	2007-2016	10
Russian Federation	Zabaikalsk kray	2007-2016	10	2007-2016	10
Russian Federation	Perm kray	2007-2016	10	2007-2016	10
Russian Federation	Chuvash Republic	2007-2016	10	2007-2016	10
Russian Federation	Novosibirsk oblast	2007-2016	10	2007-2016	10
Russian Federation	Sverdlovsk oblast	2007-2016	10	2007-2016	10
Russian Federation	Khanty-Mansi autonomous area	2011-2016	6	2011-2016	6
Russian Federation	Yamalo-Nenets autonomous area	2011-2016	6	2011-2016	6
Russian Federation	Republic of Tuva	2007-2016	10	2007-2016	10
Russian Federation	Sakhalin oblast	2007-2016	10	2007-2016	10
Russian Federation	Magadan oblast	2007-2015	9	2007-2016	9
Russian Federation	Amur oblast	2007-2016	10	2007-2016	10
Russian Federation	Khabarovsk kray	2007-2016	10	2007-2016	10
Russian Federation	Udmurt Republic	2007-2016	10	2007-2016	10

Russian Federation	Primorsky kray	2007-2016	10	2007-2016	10
Russian Federation	Kamchatka kray	2007-2016	10	2007-2016	10
Russian Federation	Republic of Sakha (Yakutia)	2007-2016	10	2007-2016	10
Russian Federation	Tomsk oblast	2007-2016	10	2007-2016	10
Russian Federation	Omsk oblast	2007-2016	10	2007-2016	10
Russian Federation	Republic of Buryatia	2007-2016	10	2007-2016	10
Russian Federation	Republic of Altai	2007-2015	9	2007-2016	9
Russian Federation	Chelyabinsk oblast	2007-2016	10	2007-2016	10
Russian Federation	Tyumen oblast without autonomous areas	2011-2015	5	2011-2015	5
Russian Federation	Jewish autonomous oblast	2007-2016	10	2007-2016	10
Russian Federation	Republic of Tatarstan	2007-2016	10	2007-2016	10
Russian Federation	Altai kray	2007-2016	10	2007-2016	10
Russian Federation	Republic of Mariy El	2007-2016	10	2007-2016	10
Russian Federation	Tambov oblast	2007-2016	10	2007-2016	10
Russian Federation	Astrakhan oblast	2007-2016	10	2007-2016	10
Russian Federation	Krasnodar kray	2007-2016	10	2007-2016	10
Russian Federation	Tver oblast	2007-2016	10	2007-2016	10
Russian Federation	Tula oblast	2007-2016	10	2007-2016	10
Russian Federation	Yaroslavl oblast	2007-2016	10	2007-2016	10
Russian Federation	Moscow City	2007-2016	10	2007-2016	10
Russian Federation	Republic of Karelia	2007-2016	10	2007-2016	10
Russian Federation	Komi Republic	2007-2016	10	2007-2016	10
Russian Federation	Smolensk oblast	2007-2016	10	2007-2016	10
Russian Federation	Arkhangelsk oblast without Nenets autonomous district	2007-2015	9	2007-2015	9
Russian Federation	Republic of Kalmykia	2007-2015	9	2007-2016	9
Russian Federation	Republic of Adygeya	2007-2016	10	2007-2016	10
Russian Federation	Sankt-Petersburg	2007-2016	10	2007-2016	10
Russian Federation	Pskov oblast	2007-2016	10	2007-2016	10
Russian Federation	Novgorod oblast	2007-2016	10	2007-2016	10
Russian Federation	Murmansk oblast	2007-2016	10	2007-2016	10
Russian Federation	Leningrad oblast	2007-2016	10	2007-2016	10
Russian Federation	Kaliningrad oblast	2007-2016	10	2007-2016	10
Russian Federation	Republic of Khakasia	2007-2016	10	2007-2016	10
Russian Federation	Vologda oblast	2007-2016	10	2007-2016	10
Russian Federation	Republic of Mordovia	2007-2016	10	2007-2016	10
Russian Federation	Ryazan oblast	2007-2016	10	2007-2016	10
Russian Federation	Moscow oblast	2007-2016	10	2007-2016	10

Russian Federation	Republic of Bashkortostan	2007-2016	10	2007-2016	10
Russian Federation	Stavropol kray	2007-2016	10	2007-2016	10
Russian Federation	Chechen Republic	2007-2016	10	2007-2016	10
Russian Federation	Republic of North Ossetia-Alania	2007-2016	10	2007-2016	10
Russian Federation	Karachaev-Chercassian Republic	2007-2016	10	2007-2016	10
Russian Federation	Kabardian-Balkar Republic	2007-2016	10	2007-2016	10
Russian Federation	Republic of Ingushetia	2007-2016	10	2007-2016	10
Russian Federation	Republic of Dagestan	2007-2016	10	2007-2016	10
Russian Federation	Rostov oblast	2007-2016	10	2007-2016	10
Russian Federation	Oryol oblast	2007-2016	10	2007-2016	10
Russian Federation	Volgograd oblast	2007-2016	10	2007-2016	10
Russian Federation	Belgorod oblast	2007-2016	10	2007-2016	10
Russian Federation	Bryansk oblast	2007-2016	10	2007-2016	10
Russian Federation	Vladimir oblast	2007-2016	10	2007-2016	10
Russian Federation	Voronezh oblast	2007-2016	10	2007-2016	10
Russian Federation	Ivanovo oblast	2007-2016	10	2007-2016	10
Russian Federation	Kaluga oblast	2007-2016	10	2007-2016	10
Russian Federation	Kostroma oblast	2007-2016	10	2007-2016	10
Russian Federation	Kursk oblast	2007-2016	10	2007-2016	10
Russian Federation	Lipetzk oblast	2007-2016	10	2007-2016	10
Russian Federation	St Petersburg	1983-1987, 1994-2002	14	1983-1987, 1994-2002	0
Samoa	National Registry	1980-1988	9	1980-1988	0
Saudi Arabia	Riyadh	1994-2012	19	1994-2012	0
Saudi Arabia	National Registry	1994-1996, 2006-2012	10	1994-1996, 2006-2012	7
Senegal	Dakar	1969-1974	6	1969-1974	0
Serbia	Central Serbia	2003-2007	5	2003-2007	5
Serbia	Vojvodina	1988-1997	10	1988-1997	0
Serbia	National Registry	1999-2002	4	1999-2002	0
Seychelles	National Registry	2009-2012	4	2009-2012	0
Singapore	National Registry	1950-1961, 1968-2015	60	1950-1961, 1968-2015	0
Slovakia	National Registry	1968-2010	43	1968-2010	30
Slovenia	National Registry	1956-2014	59	1956-2014	12
South Africa	Johannesburg, Bantu	1953-1955	3	1953-1955	0
South Africa	PROMEC	1998-2007	10	1998-2007	0
South Africa	Eastern Cape	2008-2012	5	2008-2012	0
South Africa	South Africa Paediatric	1998-2012	15	1998-2012	0

South Africa	National Registry	2003-2005, 2008-2012	8	2003-2005, 2008-2012	0
Spain	Cuenca	1993-2012	20	1993-2012	5
Spain	Ciudad Real	2004-2011	8	2004-2011	0
Spain	Canary Islands	1993-1995, 1997-2001, 2003-2006, 2008-2011	16	1993-1995, 1997-2001, 2003-2006, 2008-2011	0
Spain	Basque Country	1986-2012	27	1986-2012	4
Spain	Girona	1980-2012	33	1980-2012	23
Spain	La Rioja	1993-2012	20	1993-2012	13
Spain	Murcia	1983-2010	28	1983-2010	25
Spain	Navarra	1973-2010	38	1973-2010	31
Spain	Tarragona	1980-2012	33	1980-2012	24
Spain	Zaragoza	1968-2000	33	1968-2000	0
Spain	11 Registries Combined (Albacete, Asturias, Balearic Islands, Basque Country, Canary Islands, Cuenca, Girona, Granada, Murcia, Navarra, Tarragona)	1990-2013	24	1990-2013	0
Spain	Albacete	1991-2010	20	1991-2010	6
Spain	Granada	1985-2012	28	1985-2012	23
Spain	Mallorca	1988-1996, 2003-2011	18	1988-1996, 2003-2011	0
Spain	Balears	1988-2005	18	1988-2005	0
Spain	Asturias	1982-2000, 2003-2010	21	1988-2000, 2003-2010	5
Sri Lanka	National Registry	2001-2005	5	2001-2005	0
Sweden	National Registry	1958-2014	57	1958-2014	35
Sweden	Sweden except Stockholm	2002-2016	15	2002-2016	0
Sweden	Stockholm	2002-2016	15	2002-2016	0
Switzerland	Zurich	1980-2012	33	1980-2012	0
Switzerland	Fribourg	2008-2012	5	2008-2012	0
Switzerland	7 Registries Combined (Fribourg, Geneva, Neuchatel, Ticino, Valais, Vaud, Zurich)	1990-2013	24	1990-2013	0
Switzerland	Switzerland Paediatric	1990-2013	24	1990-2013	0
Switzerland	National Registry	1989-2013	25	1985-2017	2
Switzerland	Graubunden and Glarus	1989-2012	24	1980-2012	20
Switzerland	Graubunden	1989-1997	9	1989-1997	0

Switzerland	Neuchatel	1974-1976, 1978-1996, 1998-2012	37	1974-1976, 1978-2012	5
Switzerland	St Gallen - Appenzell	1980-2012	33	1980-2012	29
Switzerland	Ticino	1996-2012	17	1996-2012	0
Switzerland	Valais	1989-2012	24	1989-2012	0
Switzerland	Vaud	1975-1996, 1998-2012	37	1975-2012	5
Switzerland	Basel	1981-1997, 2003-2007	22	1981-1997, 2003-2007	0
Switzerland	Geneva	1970-2012	43	1970-2012	29
Taiwan (Province of China)	National Registry	1980-2009	30	1980-2009	28
Thailand	Udon Thani	2001-2003	3	2001-2003	0
Thailand	Songkhla	1993-1996, 1998-2012	19	1993-1996, 1998-2012	0
Thailand	Lopburi Province	2009-2012	4	2009-2012	0
Thailand	Bangkok	1995-1997, 2001-2012	15	1995-1997, 2001-2012	0
Thailand	Chiang Mai	1983-2012	30	1983-2012	0
Thailand	Chonburi	2001-2011	11	2001-2011	0
Thailand	Surat Thani	2001-2003	3	2001-2003	0
Thailand	Khon Kaen	1985-2012	22	1988-1997, 2001-2012	0
Thailand	Lop Buri	2001-2003	3	2001-2003	0
Thailand	Nakhon Phanom	2001-2003	3	2001-2003	0
Thailand	Prachuap Khiri	2001-2003	3	2001-2003	0
Thailand	Rayong	2001-2003	3	2001-2003	0
Thailand	Lampang	1993-2013	21	1993-2013	0
Thailand	Ubon Ratchathani	2001-2003	3	2001-2003	0
Trinidad and Tobago	National Registry	1995-2006	12	1995-2006	12
Tunisia	North Tunisia	1994-2006	13	1994-2006	0
Tunisia	Central Tunisia	1993-2007	15	1993-2007	0
Tunisia	Centre Sousse	1998-2002	5	1998-2002	0
Turkey	Nine Provinces Combined (Ankara, Antalya, Bursa, Edirne, Erzurum, Eskisehir, Izmir, Samsun, Trabzon)	2009-2014	6	2009-2014	0
Turkey	Izmir	1993-1996, 1998-2012	15	1998-2012	0
Turkey	Eskisehir	2002-2005, 2008-2012	9	2002-2005, 2008-2012	0
Turkey	Erzurum	2002-2005, 2010-2012	7	2002-2005, 2010-2012	0

Turkey	Edirne	2002-2012	11	2002-2012	0
Turkey	Antalya	1998-2012	15	1998-2012	0
Turkey	Ankara	2002-2005	4	2002-2005	0
Turkey	Bursa	2008-2012	5	2008-2012	0
Turkey	Trabzon	2002-2003, 2005-2012	10	2002-2003, 2005-2012	0
Turkey	Samsun	2002-2005, 2008-2012	9	2002-2005, 2008-2012	0
Turkey	8 Registries Combined (Ankara, Antalya, Bursa, Edirne, Erzurum, Izmir, Samsun, Trabzon)	1992-2012	21	1992-2012	0
Uganda	Kampala	1954-1960, 1990-2013	31	1954-1960, 1990-2013	0
Ukraine	Sevastopol	2014-2015	2	2014-2016	1
Ukraine	National Registry	2002-2012	11	2002-2012	0
Ukraine	Republic of Crimea	2014-2016	3	2014-2016	2
Ukraine	National Registry (without Crimea & Sevastopol)	2000-2012	13	2000-2012	2
United Kingdom	Bury	2015-2017	3	2015-2017	0
United Kingdom	Barnsley	2015-2017	3	2015-2017	0
United Kingdom	Yorkshire	1983-2002	20	1983-2002	0
United Kingdom	Northern England and Yorkshire	1998-2002, 2008-2012	10	1998-2002, 2008-2012	0
United Kingdom	York	2015-2017	3	2015-2017	0
United Kingdom	Wakefield	2015-2017	3	2015-2017	0
United Kingdom	Sheffield	2015-2017	3	2015-2017	0
United Kingdom	Rotherham	2015-2017	3	2015-2017	0
United Kingdom	North Lincolnshire	2015-2017	3	2015-2017	0
United Kingdom	North East Lincolnshire	2015-2017	3	2015-2017	0
United Kingdom	Leeds	2015-2017	3	2015-2017	0
United Kingdom	Kirklees	2015-2017	3	2015-2017	0
United Kingdom	Yorkshire and the Humber	1990-2014	25	1981-2014	17
United Kingdom	Cumbria	2015-2017	3	2015-2017	0
United Kingdom	Cheshire West and Chester	2015-2017	3	2015-2017	0
United Kingdom	Cheshire East	2015-2017	3	2015-2017	0
United Kingdom	Bradford	2015-2017	3	2015-2017	0
United Kingdom	North Yorkshire	2015-2017	3	2015-2017	0
United Kingdom	Calderdale	2015-2017	3	2015-2017	0
United Kingdom	Croydon	2015-2017	3	2015-2017	0
United Kingdom	East Riding of Yorkshire	2015-2017	3	2015-2017	0

United Kingdom	Bolton	2015-2017	3	2015-2017	0
United Kingdom	Camden	2015-2017	3	2015-2017	0
United Kingdom	Walsall	2015-2017	3	2015-2017	0
United Kingdom	Telford and Wrekin	2015-2017	3	2015-2017	0
United Kingdom	Stoke-on-Trent	2015-2017	3	2015-2017	0
United Kingdom	West Midlands	1960-1966, 1968-1976, 1979-2014	52	1960-1966, 1968-1976, 1979, 1981-2009, 2011-2014	25
United Kingdom	Nottinghamshire	2015-2017	3	2015-2017	0
United Kingdom	Nottingham	2015-2017	3	2015-2017	0
United Kingdom	Northamptonshire	2015-2017	3	2015-2017	0
United Kingdom	Lincolnshire	2015-2017	3	2015-2017	0
United Kingdom	Leicestershire	2015-2017	3	2015-2017	0
United Kingdom	Leicester	2015-2017	3	2015-2017	0
United Kingdom	Derbyshire	2015-2017	3	2015-2017	0
United Kingdom	Derby	2015-2017	3	2015-2017	0
United Kingdom	Rutland	2015-2017	3	2015-2017	0
United Kingdom	East Midlands	1990-2014	25	1981-2009, 2011-2014	16
United Kingdom	Kingston upon Hull, City of	2015-2017	3	2015-2017	0
United Kingdom	Doncaster	2015-2017	3	2015-2017	0
United Kingdom	Blackpool	2015-2017	3	2015-2017	0
United Kingdom	National Registry	2008-2012	5	2008-2012	0
United Kingdom	Wirral	2015-2017	3	2015-2017	0
United Kingdom	Darlington	2015-2017	3	2015-2017	0
United Kingdom	County Durham	2015-2017	3	2015-2017	0
United Kingdom	Sunderland	2015-2017	3	2015-2017	0
United Kingdom	Stockton-on-Tees	2015-2017	3	2015-2017	0
United Kingdom	South Tyneside	2015-2017	3	2015-2017	0
United Kingdom	Redcar and Cleveland	2015-2017	3	2015-2017	0
United Kingdom	North East England	1990-2014	25	1981-2009, 2011-2014	16
United Kingdom	West Scotland	1975-1992	18	1975-1992	0
United Kingdom	South East Scotland	1973-1987	15	1973-1987	0
United Kingdom	North Scotland	1973-1987	15	1973-1987	0
United Kingdom	North East Scotland	1973-1987	15	1973-1987	0
United Kingdom	East Scotland	1973-1987	15	1973-1987	0
United Kingdom	Aryshire	1970-1972	3	1970-1972	0
United Kingdom	Scotland	1963-1966, 1975-2015	45	1963-1966, 1974-2015	25

United Kingdom	Northern Ireland	1993-2012	20		0
United Kingdom	England and Wales	1979-1990	12	1979-1990	0
United Kingdom	Wolverhampton	2015-2017	3	2015-2017	0
United Kingdom	Gateshead	2015-2017	3	2015-2017	0
United Kingdom	Hartlepool	2015-2017	3	2015-2017	0
United Kingdom	Middlesbrough	2015-2017	3	2015-2017	0
United Kingdom	Newcastle upon Tyne	2015-2017	3	2015-2017	0
United Kingdom	Wigan	2015-2017	3	2015-2017	0
United Kingdom	Warrington	2015-2017	3	2015-2017	0
United Kingdom	Trafford	2015-2017	3	2015-2017	0
United Kingdom	Tameside	2015-2017	3	2015-2017	0
United Kingdom	Stockport	2015-2017	3	2015-2017	0
United Kingdom	Merseyside and Cheshire	1959-1966, 1968-1972, 1975-2002	33	1959-1966, 1968-1972, 1975-1979, 1988-2002	0
United Kingdom	St. Helens	2015-2017	3	2015-2017	0
United Kingdom	Sefton	2015-2017	3	2015-2017	0
United Kingdom	Blackburn with Darwen	2015-2017	3	2015-2017	0
United Kingdom	Salford	2015-2017	3	2015-2017	0
United Kingdom	Oldham	2015-2017	3	2015-2017	0
United Kingdom	Manchester	2015-2017	3	2015-2017	0
United Kingdom	Liverpool	2015-2017	3	2015-2017	0
United Kingdom	Lancashire	2015-2017	3	2015-2017	0
United Kingdom	Knowsley	2015-2017	3	2015-2017	0
United Kingdom	Halton	2015-2017	3	2015-2017	0
United Kingdom	North West England	1973-1977, 1979-2014	41	1973-1977, 1979, 1988-2009, 2011-2014	16
United Kingdom	Northumberland	2015-2017	3	2015-2017	0
United Kingdom	Rochdale	2015-2017	3	2015-2017	0
United Kingdom	Worcestershire	2015-2017	3	2015-2017	0
United Kingdom	Warwickshire	2015-2017	3	2015-2017	0
United Kingdom	Coventry	2015-2017	3	2015-2017	0
United Kingdom	Southwark	2015-2017	3	2015-2017	0
United Kingdom	Barking and Dagenham	2015-2017	3	2015-2017	0
United Kingdom	Sutton	2015-2017	3	2015-2017	0
United Kingdom	Tower Hamlets	2015-2017	3	2015-2017	0
United Kingdom	Waltham Forest	2015-2017	3	2015-2017	0
United Kingdom	Wandsworth	2015-2017	3	2015-2017	0
United Kingdom	Richmond upon Thames	2015-2017	3	2015-2017	0
United Kingdom	Westminster	2015-2017	3	2015-2017	0

United Kingdom	Bexley	2015-2017	3	2015-2017	0
United Kingdom	Brent	2015-2017	3	2015-2017	0
United Kingdom	Bromley	2015-2017	3	2015-2017	0
United Kingdom	England	1971-2013, 2015-2016	26	1990-2013, 2015-2016	0
United Kingdom	Wales	2003-2012	10	1981-2012	2
United Kingdom	Dorset	2015-2017	3	2015-2017	0
United Kingdom	Barnet	2015-2017	3	2015-2017	0
United Kingdom	Redbridge	2015-2017	3	2015-2017	0
United Kingdom	Newham	2015-2017	3	2015-2017	0
United Kingdom	Merton	2015-2017	3	2015-2017	0
United Kingdom	Enfield	2015-2017	3	2015-2017	0
United Kingdom	Greenwich	2015-2017	3	2015-2017	0
United Kingdom	Hackney & City of London	2015-2017	3	2015-2017	0
United Kingdom	Hammersmith and Fulham	2015-2017	3	2015-2017	0
United Kingdom	Haringey	2015-2017	3	2015-2017	0
United Kingdom	Harrow	2015-2017	3	2015-2017	0
United Kingdom	Havering	2015-2017	3	2015-2017	0
United Kingdom	Hillingdon	2015-2017	3	2015-2017	0
United Kingdom	Hounslow	2015-2017	3	2015-2017	0
United Kingdom	Thames	1998-2007	5	1991-1999, 2001-2007	0
United Kingdom	Islington	2015-2017	3	2015-2017	0
United Kingdom	Kensington and Chelsea	2015-2017	3	2015-2017	0
United Kingdom	Kingston upon Thames	2015-2017	3	2015-2017	0
United Kingdom	Lambeth	2015-2017	3	2015-2017	0
United Kingdom	Lewisham	2015-2017	3	2015-2017	0
United Kingdom	Devon	2015-2017	3	2015-2017	0
United Kingdom	Cornwall	2015-2017	3	2015-2017	0
United Kingdom	Bristol, City of	2015-2017	3	2015-2017	0
United Kingdom	Bournemouth	2015-2017	3	2015-2017	0
United Kingdom	Wokingham	2015-2017	3	2015-2017	0
United Kingdom	Windsor and Maidenhead	2015-2017	3	2015-2017	0
United Kingdom	West Sussex	2015-2017	3	2015-2017	0
United Kingdom	Oxford	1963-1966, 1968-1972, 1974-1977, 1979-2007	42	1963-1966, 1968-1972, 1974-1977, 1979-2007	0
United Kingdom	West Berkshire	2015-2017	3	2015-2017	0
United Kingdom	Surrey	2015-2017	3	2015-2017	0

United Kingdom	Southampton	2015-2017	3	2015-2017	0
United Kingdom	Slough	2015-2017	3	2015-2017	0
United Kingdom	Reading	2015-2017	3	2015-2017	0
United Kingdom	Portsmouth	2015-2017	3	2015-2017	0
United Kingdom	Oxfordshire	2015-2017	3	2015-2017	0
United Kingdom	Milton Keynes	2015-2017	3	2015-2017	0
United Kingdom	Medway	2015-2017	3	2015-2017	0
United Kingdom	Kent	2015-2017	3	2015-2017	0
United Kingdom	South East England	1990-2014	25	1981-2009, 2011-2014	16
United Kingdom	South Thames	1960-1997	38	1960-1997	0
United Kingdom	Ealing	2015-2017	3	2015-2017	0
United Kingdom	Bracknell Forest	2015-2017	3	2015-2017	0
United Kingdom	Buckinghamshire	2015-2017	3	2015-2017	0
United Kingdom	Bath and North East Somerset	2015-2017	3	2015-2017	0
United Kingdom	Trent	1963-1970, 1974-1976, 1979-1987, 1993-2002, 2005-2007	24	1963-1970, 1974-1976, 1991-2007	0
United Kingdom	Wiltshire	2015-2017	3	2015-2017	0
United Kingdom	Torbay	2015-2017	3	2015-2017	0
United Kingdom	Swindon	2015-2017	3	2015-2017	0
United Kingdom	South Gloucestershire	2015-2017	3	2015-2017	0
United Kingdom	Somerset	2015-2017	3	2015-2017	0
United Kingdom	Poole	2015-2017	3	2015-2017	0
United Kingdom	Plymouth	2015-2017	3	2015-2017	0
United Kingdom	North Somerset	2015-2017	3	2015-2017	0
United Kingdom	Gloucestershire	2015-2017	3	2015-2017	0
United Kingdom	South West England	1960-1970, 1979-2014	45	1960-1970, 1981-1984, 1986-1989, 1991-2009, 2011-2014	15
United Kingdom	Isle of Wight	2015-2017	3	2015-2017	0
United Kingdom	Hampshire	2015-2017	3	2015-2017	0
United Kingdom	East Sussex	2015-2017	3	2015-2017	0
United Kingdom	Brighton and Hove	2015-2017	3	2015-2017	0
United Kingdom	Birmingham	2015-2017	3	2015-2017	0
United Kingdom	North Tyneside	2015-2017	3	2015-2017	0
United Kingdom	Central Bedfordshire	2015-2017	3	2015-2017	0

United Kingdom	Greater London	1990-2014	25	1981-1999, 2001-2009, 2011-2014	16
United Kingdom	Norfolk	2015-2017	3	2015-2017	0
United Kingdom	Luton	2015-2017	3	2015-2017	0
United Kingdom	Hertfordshire	2015-2017	3	2015-2017	0
United Kingdom	Essex	2015-2017	3	2015-2017	0
United Kingdom	Cambridgeshire	2015-2017	3	2015-2017	0
United Kingdom	Bedford	2015-2017	3	2015-2017	0
United Kingdom	East Anglia	1988-1997	10	1988-1997	0
United Kingdom	Suffolk	2015-2017	3	2015-2017	0
United Kingdom	Thurrock	2015-2017	3	2015-2017	0
United Kingdom	Peterborough	2015-2017	3	2015-2017	0
United Kingdom	East of England	1990-2014	25	1981-2009, 2011-2014	16
United Kingdom	Staffordshire	2015-2017	3	2015-2017	0
United Kingdom	Solihull	2015-2017	3	2015-2017	0
United Kingdom	Shropshire	2015-2017	3	2015-2017	0
United Kingdom	Sandwell	2015-2017	3	2015-2017	0
United Kingdom	Herefordshire, County of	2015-2017	3	2015-2017	0
United Kingdom	Dudley	2015-2017	3	2015-2017	0
United Kingdom	Southend-on-Sea	2015-2017	3	2015-2017	0
United States of America	Oregon	1998-2012	15	1998-2012	0
United States of America	Rhode Island	1998-2012	15	1998-2012	0
United States of America	Pennsylvania	1998-2012	15	1998-2012	0
United States of America	Oklahoma	1998-2012	15	1998-2012	0
United States of America	San Francisco	1973-2012	40	1973-2012	0
United States of America	North Dakota	2003-2012	10	2003-2012	0
United States of America	North Carolina	2003-2012	10	2003-2012	0
United States of America	San Jose Monterey	1973-2013	41	1973-2013	24
United States of America	South Carolina	1998-2012	15	1998-2012	0
United States of America	Ohio	1998-2012	15	1998-2012	0
United States of America	South Dakota	2003-2012	10	2003-2012	0
United States of America	Wyoming	2003-2012	10	2003-2012	0
United States of America	Texas	1998-2012	15	1998-2012	0
United States of America	El Paso	1960-1966, 1968-1970	10	1960-1966, 1968-1970	0
United States of America	Utah	1973-2013	41	1973-2013	37
United States of America	Vermont	1998-2012	15	1998-2012	0
United States of America	Virginia	1998-2012	15	1998-2012	0
United States of America	Washington	1998-2012	15	1998-2012	0
United States of America	Seattle	1973-2013	41	1973-2013	36
United States of America	West Virginia	1998-2012	15	1998-2012	0

United States of America	Wisconsin	1998-2012	15	1998-2012	0
United States of America	New York	1993-2012	20	1993-2012	0
United States of America	Tennessee	1999-2012	14	1999-2012	0
United States of America	New Mexico	1969-2013	45	1969-2013	37
United States of America	New York except New York City	1959-1961	3	1959-1961	0
United States of America	New Hampshire	2003-2012	10	2003-2012	0
United States of America	Rural Georgia	1973-2013	41	1973-2013	24
United States of America	Greater Georgia	1973-2013	41	1973-2013	13
United States of America	Atlanta	1973-2013	41	1973-2013	36
United States of America	Georgia	1998-2012	15	1998-2012	0
United States of America	Florida	1998-2012	15	1998-2012	0
United States of America	District of Columbia	1998-2002	5	1998-2002	0
United States of America	Delaware	1998-2012	15	1998-2012	0
United States of America	Connecticut	1960-1962, 1973-2013	44	1960-1962, 1973-2013	37
United States of America	NPCR	2008-2012	5	2008-2012	0
United States of America	New Jersey	1973-2013	41	1973-2013	19
United States of America	National Registry	1962-1966, 1968-1977, 1983-2012	45	1962-1966, 1968-1977, 1983-2012	0
United States of America	Colorado	1998-2012	15	1998-2012	0
United States of America	Arizona	1998-2012	15	1998-2012	0
United States of America	Arkansas	2003-2012	10	2003-2012	0
United States of America	California	1998-2012	15	1998-2012	0
United States of America	Alameda County	1983-1987	5	1983-1987	0
United States of America	California except SF, SJ-M, & LA	2000-2013	14	2000-2013	9
United States of America	Central California	1988-1992	5	1988-1992	0
United States of America	Los Angeles	1973-2013	41	1973-2013	24
United States of America	San Francisco, Oakland, San Mateo, and Surrounding Area	1973-2013	41	1973-2013	36
United States of America	Alabama	1998-2012	15	1998-2012	0
United States of America	Idaho	1998-2012	15	1998-2012	0
United States of America	Hawaii	1960-1964, 1968-2013	51	1960-1964, 1968-2013	37
United States of America	Massachusetts	1998-2012	15	1998-2012	0
United States of America	Nevada	1959-1966, 2008-2012	13	1959-1966, 2008-2012	0
United States of America	Nebraska	1998-2012	15	1998-2012	0
United States of America	Montana	1998-2012	15	1998-2012	0
United States of America	Missouri	1996-2012	17	1996-2012	0

United States of America	Mississippi	2003-2012	10	2003-2012	0
United States of America	Minnesota	1998-2012	15	1998-2012	0
United States of America	Detroit	1973-2013	41	1973-2013	37
United States of America	Michigan	1998-2012	15	1998-2012	0
United States of America	Maryland	1998-2012	15	1998-2012	0
United States of America	Alaska	1992-2013	22	1992-2013	17
United States of America	Maine	1998-2012	15	1998-2012	0
United States of America	New Orleans	1983-2012	30	1983-2012	0
United States of America	Louisiana	1973-2013	41	1973-2013	18
United States of America	Kentucky	1973-2013	41	1973-2013	19
United States of America	Iowa	1973-2013	41	1973-2013	37
United States of America	Indiana	1998-2012	15	1998-2012	0
United States of America	Illinois	1998-2012	15	1998-2012	0
Uruguay	Montevideo	1990-1995	6	1990-1995	0
Uruguay	National Registry	2002-2014	13	2002-2014	0
Viet Nam	Ho Chi Minh	1995-1998, 2009-2012	8	1995-1998, 2009-2012	0
Viet Nam	Hanoi	1991-1997	7	1991-1997	0
Yemen	Aden	1997-2011	5	2007-2011	0
Zimbabwe	National Registry	2005-2006	2	2005-2006	0
Zimbabwe	Bulawayo	1963-1972	10	1963-1972	0
Zimbabwe	Harare	1990-2013	24	1990-2013	0

Abbreviations: MIR, mortality to incidence ratio.

Footnote: Cancer registry incidence data is only used for estimating MIRs (column “Years used for MIR”) if both incidence and mortality data are available. While this manuscript only reports estimates from 2010-2019, the columns reporting the years of data used can include values greater than 10 because earlier years were used to estimate the entire GBD time series.

eTable 7: Covariates selected for CODEm for each GBD cancer group and expected direction of covariate

Cause	Sex	Age start	Age end	Level	Direction	Covariate
Acute lymphoid leukemia	Male	0-6 days	95+ years	2	1	Tobacco (cigarettes per capita)
Acute lymphoid leukemia	Male	0-6 days	95+ years	2	1	Cumulative Cigarettes (10 Years)
Acute lymphoid leukemia	Male	0-6 days	95+ years	2	1	Cumulative Cigarettes (20 Years)
Acute lymphoid leukemia	Male	0-6 days	95+ years	3	-1	Education (years per capita)
Acute lymphoid leukemia	Male	0-6 days	95+ years	3	-1	LDI (I$ per capita)
Acute lymphoid leukemia	Male	0-6 days	95+ years	2	1	Mean BMI
Acute lymphoid leukemia	Male	0-6 days	95+ years	1	1	Log-transformed SEV scalar: Leukemia
Acute lymphoid leukemia	Male	0-6 days	95+ years	1	1	Log-transformed age-standardized SEV scalar: Leukemia
Acute lymphoid leukemia	Male	0-6 days	95+ years	3	1	Socio-demographic Index
Acute lymphoid leukemia	Male	0-6 days	95+ years	2	-1	Healthcare access and quality index
Acute lymphoid leukemia	Male	0-6 days	95+ years	2	1	Liters of alcohol consumed per capita
Acute lymphoid leukemia	Female	0-6 days	95+ years	2	1	Tobacco (cigarettes per capita)
Acute lymphoid leukemia	Female	0-6 days	95+ years	2	1	Cumulative Cigarettes (10 Years)
Acute lymphoid leukemia	Female	0-6 days	95+ years	2	1	Cumulative Cigarettes (20 Years)
Acute lymphoid leukemia	Female	0-6 days	95+ years	3	-1	Education (years per capita)
Acute lymphoid leukemia	Female	0-6 days	95+ years	3	-1	LDI (I$ per capita)
Acute lymphoid leukemia	Female	0-6 days	95+ years	2	1	Mean BMI
Acute lymphoid leukemia	Female	0-6 days	95+ years	1	1	Log-transformed SEV scalar: Leukemia
Acute lymphoid leukemia	Female	0-6 days	95+ years	1	1	Log-transformed age-standardized SEV scalar: Leukemia
Acute lymphoid leukemia	Female	0-6 days	95+ years	3	1	Socio-demographic Index
Acute lymphoid leukemia	Female	0-6 days	95+ years	2	-1	Healthcare access and quality index
Acute lymphoid leukemia	Female	0-6 days	95+ years	2	1	Liters of alcohol consumed per capita
Acute myeloid leukemia	Male	0-6 days	95+ years	2	1	Cumulative Cigarettes (10 Years)
Acute myeloid leukemia	Male	0-6 days	95+ years	2	1	Cumulative Cigarettes (20 Years)
Acute myeloid leukemia	Male	0-6 days	95+ years	3	-1	Education (years per capita)
Acute myeloid leukemia	Male	0-6 days	95+ years	3	1	LDI (I$ per capita)
Acute myeloid leukemia	Male	0-6 days	95+ years	2	1	Mean BMI
Acute myeloid leukemia	Male	0-6 days	95+ years	2	1	Smoking Prevalence

Acute myeloid leukemia	Male	0-6 days	95+ years	1	1	Log-transformed SEV scalar: Leukemia
Acute myeloid leukemia	Male	0-6 days	95+ years	1	1	Log-transformed age-standardized SEV scalar: Leukemia
Acute myeloid leukemia	Male	0-6 days	95+ years	3	1	Socio-demographic Index
Acute myeloid leukemia	Male	0-6 days	95+ years	2	-1	Healthcare access and quality index
Acute myeloid leukemia	Male	0-6 days	95+ years	2	1	Liters of alcohol consumed per capita
Acute myeloid leukemia	Female	0-6 days	95+ years	2	1	Cumulative Cigarettes (10 Years)
Acute myeloid leukemia	Female	0-6 days	95+ years	2	1	Cumulative Cigarettes (20 Years)
Acute myeloid leukemia	Female	0-6 days	95+ years	3	-1	Education (years per capita)
Acute myeloid leukemia	Female	0-6 days	95+ years	3	1	LDI (I$ per capita)
Acute myeloid leukemia	Female	0-6 days	95+ years	2	1	Mean BMI
Acute myeloid leukemia	Female	0-6 days	95+ years	2	1	Smoking Prevalence
Acute myeloid leukemia	Female	0-6 days	95+ years	1	1	Log-transformed SEV scalar: Leukemia
Acute myeloid leukemia	Female	0-6 days	95+ years	1	1	Log-transformed age-standardized SEV scalar: Leukemia
Acute myeloid leukemia	Female	0-6 days	95+ years	3	1	Socio-demographic Index
Acute myeloid leukemia	Female	0-6 days	95+ years	2	-1	Healthcare access and quality index
Acute myeloid leukemia	Female	0-6 days	95+ years	2	1	Liters of alcohol consumed per capita
Bladder cancer	Male	15-19 years	95+ years	2	1	Cumulative Cigarettes (10 Years)
Bladder cancer	Male	15-19 years	95+ years	2	1	Diabetes Fasting Plasma Glucose (mmol/L), age-standardized 25+
Bladder cancer	Male	15-19 years	95+ years	3	1	LDI (I$ per capita)
Bladder cancer	Male	15-19 years	95+ years	1	1	Smoking Prevalence
Bladder cancer	Male	15-19 years	95+ years	1	1	Schistosomiasis Prevalence Results
Bladder cancer	Male	15-19 years	95+ years	1	1	Log-transformed SEV scalar: Bladder C
Bladder cancer	Male	15-19 years	95+ years	3	1	Socio-demographic Index
Bladder cancer	Male	15-19 years	95+ years	2	-1	Healthcare access and quality index
Bladder cancer	Male	15-19 years	95+ years	3	1	Age- and sex-specific SEV for Low fruit
Bladder cancer	Male	15-19 years	95+ years	2	1	Age- and sex-specific SEV for Low vegetables
Bladder cancer	Male	15-19 years	95+ years	2	1	Liters of alcohol consumed per capita
Bladder cancer	Female	15-19 years	95+ years	2	1	Cumulative Cigarettes (10 Years)

Bladder cancer	Female	15-19 years	95+ years	2	1	Diabetes Fasting Plasma Glucose (mmol/L), age-standardized 25+
Bladder cancer	Female	15-19 years	95+ years	3	1	LDI (I$ per capita)
Bladder cancer	Female	15-19 years	95+ years	1	1	Smoking Prevalence
Bladder cancer	Female	15-19 years	95+ years	1	1	Schistosomiasis Prevalence Results
Bladder cancer	Female	15-19 years	95+ years	1	1	Log-transformed SEV scalar: Bladder C
Bladder cancer	Female	15-19 years	95+ years	3	1	Socio-demographic Index
Bladder cancer	Female	15-19 years	95+ years	2	-1	Healthcare access and quality index
Bladder cancer	Female	15-19 years	95+ years	3	1	Age- and sex-specific SEV for Low fruit
Bladder cancer	Female	15-19 years	95+ years	2	1	Age- and sex-specific SEV for Low vegetables
Bladder cancer	Female	15-19 years	95+ years	2	1	Liters of alcohol consumed per capita
Brain and nervous system cancer	Male	0-6 days	95+ years	1	1	Cumulative Cigarettes (10 Years)
Brain and nervous system cancer	Male	0-6 days	95+ years	3	-1	Education (years per capita)
Brain and nervous system cancer	Male	0-6 days	95+ years	3	-1	LDI (I$ per capita)
Brain and nervous system cancer	Male	0-6 days	95+ years	2	1	Cholesterol (total, mean per capita)
Brain and nervous system cancer	Male	0-6 days	95+ years	2	1	Systolic Blood Pressure (mmHg)
Brain and nervous system cancer	Male	0-6 days	95+ years	1	1	Smoking Prevalence
Brain and nervous system cancer	Male	0-6 days	95+ years	3	1	Socio-demographic index
Brain and nervous system cancer	Male	0-6 days	95+ years	2	-1	Healthcare access and quality index
Brain and nervous system cancer	Male	0-6 days	95+ years	2	1	Age- and sex- specific SEV for low fruit
Brain and nervous system cancer	Male	0-6 days	95+ years	2	1	Age- and sex- specific SEV for Low vegetables
Brain and nervous system cancer	Male	0-6 days	95+ years	2	1	Age- and sex-specific SEV for High red meat
Brain and nervous system cancer	Male	0-6 days	95+ years	1	1	Liters of alcohol consumed per capita
Brain and nervous system cancer	Female	0-6 days	95+ years	1	1	Cumulative Cigarettes (10 Years)
Brain and nervous system cancer	Female	0-6 days	95+ years	3	-1	Education (years per capita)
Brain and nervous system cancer	Female	0-6 days	95+ years	3	-1	LDI (I$ per capita)
Brain and nervous system cancer	Female	0-6 days	95+ years	2	1	Cholesterol (total, mean per capita)
Brain and nervous system cancer	Female	0-6 days	95+ years	2	1	Systolic Blood Pressure (mmHg)
Brain and nervous system cancer	Female	0-6 days	95+ years	1	1	Smoking Prevalence
Brain and nervous system cancer	Female	0-6 days	95+ years	3	1	Socio-demographic index

Brain and nervous system cancer	Female	0-6 days	95+ years	2	-1	Healthcare access and quality index
Brain and nervous system cancer	Female	0-6 days	95+ years	2	1	Age- and sex- specific SEV for low fruit
Brain and nervous system cancer	Female	0-6 days	95+ years	2	1	Age- and sex- specific SEV for Low vegetables
Brain and nervous system cancer	Female	0-6 days	95+ years	2	1	Age- and sex-specific SEV for High red meat
Brain and nervous system cancer	Female	0-6 days	95+ years	1	1	Liters of alcohol consumed per capita
Breast cancer	Male	15-19 years	95+ years	2	1	Cumulative cigarettes (10 years)
Breast cancer	Male	15-19 years	95+ years	2	1	Cumulative cigarettes (20 years)
Breast cancer	Male	15-19 years	95+ years	2	1	Diabetes Fasting Plasma Glucose (mmol/L), age-standardized 25+
Breast cancer	Male	15-19 years	95+ years	1	1	Mean BMI
Breast cancer	Male	15-19 years	95+ years	1	1	Total Fertility Rate
Breast cancer	Male	15-19 years	95+ years	2	1	Socio-demographic index
Breast cancer	Male	15-19 years	95+ years	1	1	Age- and sex- specific SEV for low fruit
Breast cancer	Male	15-19 years	95+ years	1	1	Liters of alcohol consumed per capita
Breast cancer	Male	15-19 years	95+ years	2	-1	Healthcare access and quality index
Breast cancer	Male	15-19 years	95+ years	2	1	Age- and sex- specific SEV for low fruit
Breast cancer	Male	15-19 years	95+ years	2	1	Age- and sex- specific SEV for Low vegetables
Breast cancer	Male	15-19 years	95+ years	1	1	Liters of alcohol consumed per capita
Breast cancer	Female	15-19 years	95+ years	2	-1	Age-specific fertility rate
Breast cancer	Female	15-19 years	95+ years	2	1	Cumulative cigarettes (10 years)
Breast cancer	Female	15-19 years	95+ years	2	1	Cumulative cigarettes (20 years)
Breast cancer	Female	15-19 years	95+ years	2	1	Diabetes Fasting Plasma Glucose (mmol/L), age-standardized 25+
Breast cancer	Female	15-19 years	95+ years	3	-1	LDI (I$ per capita)
Breast cancer	Female	15-19 years	95+ years	1	1	Mean BMI
Breast cancer	Female	15-19 years	95+ years	2	1	Smoking Prevalence
Breast cancer	Female	15-19 years	95+ years	2	-1	Total Fertility Rate
Breast cancer	Female	15-19 years	95+ years	1	1	Log-transformed SEV scalar: Breast C
Breast cancer	Female	15-19 years	95+ years	3	1	Socio-demographic index
Breast cancer	Female	15-19 years	95+ years	2	-1	Healthcare access and quality index

Breast cancer	Female	15-19 years	95+ years	2	1	Age- and sex- specific SEV for low fruit
Breast cancer	Female	15-19 years	95+ years	2	1	Age- and sex- specific SEV for Low vegetables
Breast cancer	Female	15-19 years	95+ years	1	1	Liters of alcohol consumed per capita
Cervical cancer	Female	15-19 years	95+ years	2	1	Age-specific fertility rate
Cervical cancer	Female	15-19 years	95+ years	1	1	Cumulative Cigarettes (5 Years)
Cervical cancer	Female	15-19 years	95+ years	3	-1	Education (years per capita)
Cervical cancer	Female	15-19 years	95+ years	3	-1	LDI (I$ per capita)
Cervical cancer	Female	15-19 years	95+ years	2	1	Smoking Prevalence
Cervical cancer	Female	15-19 years	95+ years	2	1	Total Fertility Rate
Cervical cancer	Female	15-19 years	95+ years	3	-1	Socio-demographic index
Cervical cancer	Female	15-19 years	95+ years	1	1	HIV age-standardized prevalence
Cervical cancer	Female	15-19 years	95+ years	2	-1	Healthcare access and quality index
Cervical cancer	Female	15-19 years	95+ years	2	1	Age- and sex- specific SEV for low fruit
Cervical cancer	Female	15-19 years	95+ years	2	1	Age- and sex- specific SEV for Low vegetables
Chronic lymphoid leukemia	Male	20-24 years	95+ years	2	1	Tobacco (cigarettes per capita)
Chronic lymphoid leukemia	Male	20-24 years	95+ years	2	1	Cumulative cigarettes (10 years)
Chronic lymphoid leukemia	Male	20-24 years	95+ years	2	1	Cumulative cigarettes (15 years)
Chronic lymphoid leukemia	Male	20-24 years	95+ years	2	1	Cumulative cigarettes (20 years)
Chronic lymphoid leukemia	Male	20-24 years	95+ years	2	1	Cumulative cigarettes (5 years)
Chronic lymphoid leukemia	Male	20-24 years	95+ years	3	-1	Education (years per capita)
Chronic lymphoid leukemia	Male	20-24 years	95+ years	3	-1	LDI (I$ per capita)
Chronic lymphoid leukemia	Male	20-24 years	95+ years	2	1	Mean BMI
Chronic lymphoid leukemia	Male	20-24 years	95+ years	2	1	Smoking Prevalence
Chronic lymphoid leukemia	Male	20-24 years	95+ years	1	1	Log-transformed SEV scalar: Leukemia
Chronic lymphoid leukemia	Male	20-24 years	95+ years	1	1	Log-transformed age-standardized SEV scalar: Leukemia
Chronic lymphoid leukemia	Male	20-24 years	95+ years	3	1	Socio-demographic Index
Chronic lymphoid leukemia	Male	20-24 years	95+ years	2	-1	Healthcare access and quality index
Chronic lymphoid leukemia	Male	20-24 years	95+ years	2	1	Liters of alcohol consumed per capita
Chronic lymphoid leukemia	Female	20-24 years	95+ years	2	1	Tobacco (cigarettes per capita)
Chronic lymphoid leukemia	Female	20-24 years	95+ years	2	1	Cumulative cigarettes (10 years)

Chronic lymphoid leukemia	Female	20-24 years	95+ years	2	1	Cumulative cigarettes (15 years)
Chronic lymphoid leukemia	Female	20-24 years	95+ years	2	1	Cumulative cigarettes (20 years)
Chronic lymphoid leukemia	Female	20-24 years	95+ years	2	1	Cumulative cigarettes (5 years)
Chronic lymphoid leukemia	Female	20-24 years	95+ years	3	-1	Education (years per capita)
Chronic lymphoid leukemia	Female	20-24 years	95+ years	3	-1	LDI (I$ per capita)
Chronic lymphoid leukemia	Female	20-24 years	95+ years	2	1	Mean BMI
Chronic lymphoid leukemia	Female	20-24 years	95+ years	2	1	Smoking Prevalence
Chronic lymphoid leukemia	Female	20-24 years	95+ years	1	1	Log-transformed SEV scalar: Leukemia
Chronic lymphoid leukemia	Female	20-24 years	95+ years	1	1	Log-transformed age-standardized SEV scalar: Leukemia
Chronic lymphoid leukemia	Female	20-24 years	95+ years	3	1	Socio-demographic Index
Chronic lymphoid leukemia (DR)	Female	20-24 years	95+ years	2	-1	Healthcare access and quality index
Chronic lymphoid leukemia (GLB)	Female	20-24 years	95+ years	1	1	Healthcare access and quality index
Chronic lymphoid leukemia	Female	20-24 years	95+ years	2	1	Liters of alcohol consumed per capita
Chronic myeloid leukemia	Male	0-6 days	95+ years	2	1	Tobacco (cigarettes per capita)
Chronic myeloid leukemia	Male	0-6 days	95+ years	2	1	Cumulative cigarettes (10 years)
Chronic myeloid leukemia	Male	0-6 days	95+ years	2	1	Cumulative cigarettes (15 years)
Chronic myeloid leukemia	Male	0-6 days	95+ years	2	1	Cumulative cigarettes (20 years)
Chronic myeloid leukemia	Male	0-6 days	95+ years	2	1	Cumulative cigarettes (5 years)
Chronic myeloid leukemia	Male	0-6 days	95+ years	3	-1	Education (years per capita)
Chronic myeloid leukemia	Male	0-6 days	95+ years	3	1	LDI (I$ per capita)
Chronic myeloid leukemia	Male	0-6 days	95+ years	2	1	Mean BMI
Chronic myeloid leukemia	Male	0-6 days	95+ years	2	1	Smoking Prevalence
Chronic myeloid leukemia	Male	0-6 days	95+ years	1	1	Log-transformed SEV scalar: Leukemia
Chronic myeloid leukemia	Male	0-6 days	95+ years	1	1	Log-transformed age-standardized SEV scalar: Leukemia
Chronic myeloid leukemia	Male	0-6 days	95+ years	3	-1	Socio-demographic Index
Chronic myeloid leukemia	Male	0-6 days	95+ years	2	-1	Healthcare access and quality index
Chronic myeloid leukemia	Male	0-6 days	95+ years	2	1	Liters of alcohol consumed per capita
Chronic myeloid leukemia	Female	0-6 days	95+ years	2	1	Tobacco (cigarettes per capita)
Chronic myeloid leukemia	Female	0-6 days	95+ years	2	1	Cumulative cigarettes (10 years)
Chronic myeloid leukemia	Female	0-6 days	95+ years	2	1	Cumulative cigarettes (15 years)

Chronic myeloid leukemia	Female	0-6 days	95+ years	2	1	Cumulative cigarettes (20 years)
Chronic myeloid leukemia	Female	0-6 days	95+ years	2	1	Cumulative cigarettes (5 years)
Chronic myeloid leukemia	Female	0-6 days	95+ years	3	-1	Education (years per capita)
Chronic myeloid leukemia	Female	0-6 days	95+ years	3	1	LDI (I$ per capita)
Chronic myeloid leukemia	Female	0-6 days	95+ years	2	1	Mean BMI
Chronic myeloid leukemia	Female	0-6 days	95+ years	2	1	Smoking Prevalence
Chronic myeloid leukemia	Female	0-6 days	95+ years	1	1	Log-transformed SEV scalar: Leukemia
Chronic myeloid leukemia	Female	0-6 days	95+ years	1	1	Log-transformed age-standardized SEV scalar: Leukemia
Chronic myeloid leukemia	Female	0-6 days	95+ years	3	-1	Socio-demographic Index
Chronic myeloid leukemia	Female	0-6 days	95+ years	2	-1	Healthcare access and quality index
Chronic myeloid leukemia	Female	0-6 days	95+ years	2	1	Liters of alcohol consumed per capita
Colon and rectum cancer	Male	5-9 years	95+ years	1	1	Tobacco (cigarettes per capita)
Colon and rectum cancer	Male	5-9 years	95+ years	2	1	Cumulative cigarettes (20 years)
Colon and rectum cancer	Male	5-9 years	95+ years	2	1	Diabetes Fasting Plasma Glucose (mmol/L), age-standardized 25+
Colon and rectum cancer	Male	5-9 years	95+ years	3	-1	Education (years per capita)
Colon and rectum cancer	Male	5-9 years	95+ years	3	1	LDI (I$ per capita)
Colon and rectum cancer	Male	5-9 years	95+ years	1	1	Mean BMI
Colon and rectum cancer	Male	5-9 years	95+ years	1	1	Log-transformed SEV scalar: Colorect C
Colon and rectum cancer	Male	5-9 years	95+ years	3	1	Socio-demographic index
Colon and rectum cancer	Male	5-9 years	95+ years	2	-1	PUFA adjusted(percent)
Colon and rectum cancer	Male	5-9 years	95+ years	3	-1	Healthcare access and quality index
Colon and rectum cancer	Male	5-9 years	95+ years	1	-1	Total Physical Activity (MET-min/week), Age-specific
Colon and rectum cancer	Male	5-9 years	95+ years	3	1	Age- and sex- specific SEV for low fruit
Colon and rectum cancer	Male	5-9 years	95+ years	2	1	Age- and sex- specific SEV for Low vegetables
Colon and rectum cancer	Male	5-9 years	95+ years	3	1	Age- and sex-specific SEV for Low nuts and seeds
Colon and rectum cancer	Male	5-9 years	95+ years	3	1	Age- and sex-specific SEV for Low milk
Colon and rectum cancer	Male	5-9 years	95+ years	1	1	Age- and sex-specific SEV for High red meat
Colon and rectum cancer	Male	5-9 years	95+ years	2	1	Age- and sex-specific SEV for Low fiber
Colon and rectum cancer	Male	5-9 years	95+ years	2	1	Age- and sex-specific SEV for Low calcium

Colon and rectum cancer	Male	5-9 years	95+ years	2	1	Liters of alcohol consumed per capita
Colon and rectum cancer	Female	5-9 years	95+ years	1	1	Tobacco (cigarettes per capita)
Colon and rectum cancer	Female	5-9 years	95+ years	2	1	Cumulative cigarettes (5 years)
Colon and rectum cancer	Female	5-9 years	95+ years	2	1	Diabetes Fasting Plasma Glucose (mmol/L), age-standardized 25+
Colon and rectum cancer	Female	5-9 years	95+ years	3	-1	Education (years per capita)
Colon and rectum cancer	Female	5-9 years	95+ years	3	1	LDI (I$ per capita)
Colon and rectum cancer	Female	5-9 years	95+ years	1	1	Mean BMI
Colon and rectum cancer	Female	5-9 years	95+ years	1	1	Log-transformed SEV scalar: Colorect C
Colon and rectum cancer	Female	5-9 years	95+ years	3	1	Socio-demographic Index
Colon and rectum cancer	Female	5-9 years	95+ years	2	-1	PUFA adjusted(percent)
Colon and rectum cancer	Female	5-9 years	95+ years	3	-1	Healthcare access and quality index
Colon and rectum cancer	Female	5-9 years	95+ years	1	-1	Total Physical Activity (MET-min/week), Age-specific
Colon and rectum cancer	Female	5-9 years	95+ years	3	1	Age- and sex- specific SEV for low fruit
Colon and rectum cancer	Female	5-9 years	95+ years	2	1	Age- and sex- specific SEV for Low vegetables
Colon and rectum cancer	Female	5-9 years	95+ years	3	1	Age- and sex-specific SEV for Low nuts and seeds
Colon and rectum cancer	Female	5-9 years	95+ years	3	1	Age- and sex-specific SEV for Low milk
Colon and rectum cancer	Female	5-9 years	95+ years	1	1	Age- and sex-specific SEV for High red meat
Colon and rectum cancer	Female	5-9 years	95+ years	2	1	Age- and sex-specific SEV for Low fiber
Colon and rectum cancer	Female	5-9 years	95+ years	2	1	Age- and sex-specific SEV for Low calcium
Colon and rectum cancer	Female	5-9 years	95+ years	2	1	Liters of alcohol consumed per capita
Esophageal cancer	Male	20-24 years	95+ years	2	1	Tobacco (cigarettes per capita)
Esophageal cancer	Male	20-24 years	95+ years	3	-1	Education (years per capita)
Esophageal cancer	Male	20-24 years	95+ years	3	1	LDI (I$ per capita)
Esophageal cancer	Male	20-24 years	95+ years	1	1	Mean BMI
Esophageal cancer	Male	20-24 years	95+ years	2	1	Indoor Air Pollution (All Cooking Fuels)
Esophageal cancer	Male	20-24 years	95+ years	3	-1	Sanitation (proportion with access)
Esophageal cancer	Male	20-24 years	95+ years	1	1	Smoking Prevalence
Esophageal cancer	Male	20-24 years	95+ years	3	-1	Improved Water Source (proportion with access)
Esophageal cancer	Male	20-24 years	95+ years	1	1	Log-transformed age-standardized SEV scalar: Esophag C

Esophageal cancer	Male	20-24 years	95+ years	3	1	Socio-demographic Index
Esophageal cancer	Male	20-24 years	95+ years	2	-1	Healthcare access and quality index
Esophageal cancer	Male	20-24 years	95+ years	2	1	Age- and sex- specific SEV for low fruit
Esophageal cancer	Male	20-24 years	95+ years	2	1	Age- and sex- specific SEV for Low vegetables
Esophageal cancer	Male	20-24 years	95+ years	1	1	Liters of alcohol consumed per capita
Esophageal cancer	Female	20-24 years	95+ years	2	1	Tobacco (cigarettes per capita)
Esophageal cancer	Female	20-24 years	95+ years	3	-1	Education (years per capita)
Esophageal cancer	Female	20-24 years	95+ years	3	1	LDI (I$ per capita)
Esophageal cancer	Female	20-24 years	95+ years	1	1	Mean BMI
Esophageal cancer	Female	20-24 years	95+ years	2	1	Indoor Air Pollution (All Cooking Fuels)
Esophageal cancer	Female	20-24 years	95+ years	3	-1	Sanitation (proportion with access)
Esophageal cancer	Female	20-24 years	95+ years	1	1	Smoking Prevalence
Esophageal cancer	Female	20-24 years	95+ years	3	-1	Improved Water Source (proportion with access)
Esophageal cancer	Female	20-24 years	95+ years	1	1	Log-transformed age-standardized SEV scalar: Esophag C
Esophageal cancer	Female	20-24 years	95+ years	3	1	Socio-demographic Index
Esophageal cancer	Female	20-24 years	95+ years	2	-1	Healthcare access and quality index
Esophageal cancer	Female	20-24 years	95+ years	2	1	Age- and sex- specific SEV for low fruit
Esophageal cancer	Female	20-24 years	95+ years	2	1	Age- and sex- specific SEV for Low vegetables
Esophageal cancer	Female	20-24 years	95+ years	1	1	Liters of alcohol consumed per capita
Gallbladder and biliary tract cancer	Male	20-24 years	95+ years	2	1	Tobacco (cigarettes per capita)
Gallbladder and biliary tract cancer	Male	20-24 years	95+ years	2	1	Cumulative cigarettes (10 years)
Gallbladder and biliary tract cancer	Male	20-24 years	95+ years	2	1	Cumulative Cigarettes (5 Years)
Gallbladder and biliary tract cancer	Male	20-24 years	95+ years	2	1	Diabetes Age-Standardized Prevalence (proportion)
Gallbladder and biliary tract cancer	Male	20-24 years	95+ years	3	-1	Education (years per capita)
Gallbladder and biliary tract cancer	Male	20-24 years	95+ years	3	1	LDI (I$ per capita)
Gallbladder and biliary tract cancer	Male	20-24 years	95+ years	1	1	Mean BMI
Gallbladder and biliary tract cancer	Male	20-24 years	95+ years	2	1	Smoking Prevalence
Gallbladder and biliary tract cancer	Male	20-24 years	95+ years	1	1	Log-transformed SEV scalar: Gallblad C
Gallbladder and biliary tract cancer	Male	20-24 years	95+ years	3	-1	Socio-demographic Index
Gallbladder and biliary tract cancer	Male	20-24 years	95+ years	2	-1	Healthcare access and quality index

Gallbladder and biliary tract cancer	Male	20-24 years	95+ years	2	1	Age- and sex- specific SEV for low fruit
Gallbladder and biliary tract cancer	Male	20-24 years	95+ years	2	1	Age- and sex- specific SEV for Low vegetables
Gallbladder and biliary tract cancer	Male	20-24 years	95+ years	2	1	Liters of alcohol consumed per capita
Gallbladder and biliary tract cancer	Female	20-24 years	95+ years	2	1	Tobacco (cigarettes per capita)
Gallbladder and biliary tract cancer	Female	20-24 years	95+ years	2	1	Cumulative cigarettes (10 years)
Gallbladder and biliary tract cancer	Female	20-24 years	95+ years	2	1	Cumulative Cigarettes (5 Years)
Gallbladder and biliary tract cancer	Female	20-24 years	95+ years	2	1	Diabetes Age-Standardized Prevalence (proportion)
Gallbladder and biliary tract cancer	Female	20-24 years	95+ years	3	-1	Education (years per capita)
Gallbladder and biliary tract cancer	Female	20-24 years	95+ years	3	1	LDI (I$ per capita)
Gallbladder and biliary tract cancer	Female	20-24 years	95+ years	1	1	Mean BMI
Gallbladder and biliary tract cancer	Female	20-24 years	95+ years	2	1	Smoking Prevalence
Gallbladder and biliary tract cancer	Female	20-24 years	95+ years	1	1	Log-transformed SEV scalar: Gallblad C
Gallbladder and biliary tract cancer	Female	20-24 years	95+ years	3	-1	Socio-demographic Index
Gallbladder and biliary tract cancer	Female	20-24 years	95+ years	2	-1	Healthcare access and quality index
Gallbladder and biliary tract cancer	Female	20-24 years	95+ years	2	1	Age- and sex- specific SEV for low fruit
Gallbladder and biliary tract cancer	Female	20-24 years	95+ years	2	1	Age- and sex- specific SEV for Low vegetables
Gallbladder and biliary tract cancer	Female	20-24 years	95+ years	2	1	Liters of alcohol consumed per capita
Hodgkin lymphoma	Male	1-4 years	95+ years	3	-1	Education (years per capita)
Hodgkin lymphoma	Male	1-4 years	95+ years	3	-1	LDI (I$ per capita)
Hodgkin lymphoma	Male	1-4 years	95+ years	3	-1	Socio-demographic Index
Hodgkin lymphoma	Male	1-4 years	95+ years	2	-1	Healthcare access and quality index
Hodgkin lymphoma	Female	1-4 years	95+ years	3	-1	Education (years per capita)
Hodgkin lymphoma	Female	1-4 years	95+ years	3	-1	LDI (I$ per capita)
Hodgkin lymphoma	Female	1-4 years	95+ years	3	-1	Socio-demographic Index
Hodgkin lymphoma	Female	1-4 years	95+ years	2	-1	Healthcare access and quality index
Kidney cancer	Male	0-6 days	95+ years	1	1	Tobacco (cigarettes per capita)
Kidney cancer	Male	0-6 days	95+ years	1	1	Cumulative cigarettes (10 years)
Kidney cancer	Male	0-6 days	95+ years	2	1	Diabetes Age-Standardized Prevalence (proportion)
Kidney cancer	Male	0-6 days	95+ years	3	-1	Education (years per capita)
Kidney cancer	Male	0-6 days	95+ years	3	1	LDI (I$ per capita)

Kidney cancer	Male	0-6 days	95+ years	1	1	Mean BMI
Kidney cancer	Male	0-6 days	95+ years	2	1	Systolic Blood Pressure (mmHg)
Kidney cancer	Male	0-6 days	95+ years	1	1	Log-transformed SEV scalar: Kidney C
Kidney cancer	Male	0-6 days	95+ years	3	1	Socio-demographic Index
Kidney cancer	Male	0-6 days	95+ years	2	-1	Healthcare access and quality index
Kidney cancer	Male	0-6 days	95+ years	2	1	Liters of alcohol consumed per capita
Kidney cancer	Female	0-6 days	95+ years	1	1	Tobacco (cigarettes per capita)
Kidney cancer	Female	0-6 days	95+ years	1	1	Cumulative cigarettes (10 years)
Kidney cancer	Female	0-6 days	95+ years	2	1	Diabetes Age-Standardized Prevalence (proportion)
Kidney cancer	Female	0-6 days	95+ years	3	-1	Education (years per capita)
Kidney cancer	Female	0-6 days	95+ years	3	1	LDI (I$ per capita)
Kidney cancer	Female	0-6 days	95+ years	1	1	Mean BMI
Kidney cancer	Female	0-6 days	95+ years	2	1	Systolic Blood Pressure (mmHg)
Kidney cancer	Female	0-6 days	95+ years	1	1	Log-transformed SEV scalar: Kidney C
Kidney cancer	Female	0-6 days	95+ years	3	1	Socio-demographic Index
Kidney cancer	Female	0-6 days	95+ years	2	-1	Healthcare access and quality index
Kidney cancer	Female	0-6 days	95+ years	2	1	Liters of alcohol consumed per capita
Larynx cancer	Male	20-24 years	95+ years	2	1	Cumulative cigarettes (10 years)
Larynx cancer	Male	20-24 years	95+ years	2	1	Cumulative cigarettes (20 years)
Larynx cancer	Male	20-24 years	95+ years	3	1	LDI (I$ per capita)
Larynx cancer	Male	20-24 years	95+ years	2	1	Population Density (over 1000 ppl/sqkm, proportion)
Larynx cancer	Male	20-24 years	95+ years	2	1	Smoking Prevalence
Larynx cancer	Male	20-24 years	95+ years	1	1	Log-transformed SEV scalar: Larynx C
Larynx cancer	Male	20-24 years	95+ years	3	1	Socio-demographic Index
Larynx cancer	Male	20-24 years	95+ years	2	-1	Healthcare access and quality index
Larynx cancer	Male	20-24 years	95+ years	2	1	Asbestos consumption (metric tons per year per capita)
Larynx cancer	Male	20-24 years	95+ years	2	1	Age- and sex- specific SEV for low fruit
Larynx cancer	Male	20-24 years	95+ years	3	1	Age- and sex- specific SEV for Low vegetables
Larynx cancer	Male	20-24 years	95+ years	1	1	Liters of alcohol consumed per capita
Larynx cancer	Female	20-24 years	95+ years	2	1	Cumulative cigarettes (10 years)

Larynx cancer	Female	20-24 years	95+ years	2	1	Cumulative cigarettes (20 years)
Larynx cancer	Female	20-24 years	95+ years	3	1	LDI (I$ per capita)
Larynx cancer	Female	20-24 years	95+ years	2	1	Population Density (over 1000 ppl/sqkm, proportion)
Larynx cancer	Female	20-24 years	95+ years	2	1	Smoking Prevalence
Larynx cancer	Female	20-24 years	95+ years	1	1	Log-transformed SEV scalar: Larynx C
Larynx cancer	Female	20-24 years	95+ years	3	1	Socio-demographic Index
Larynx cancer	Female	20-24 years	95+ years	2	-1	Healthcare access and quality index
Larynx cancer	Female	20-24 years	95+ years	2	1	Asbestos consumption (metric tons per year per capita)
Larynx cancer	Female	20-24 years	95+ years	3	1	Age- and sex- specific SEV for low fruit
Larynx cancer	Female	20-24 years	95+ years	2	1	Age- and sex- specific SEV for Low vegetables
Larynx cancer	Female	20-24 years	95+ years	1	1	Liters of alcohol consumed per capita
Leukemia	Male	0-6 days	95+ years	2	1	Tobacco (cigarettes per capita)
Leukemia	Male	0-6 days	95+ years	2	1	Cumulative cigarettes (10 years)
Leukemia	Male	0-6 days	95+ years	2	1	Cumulative cigarettes (20 years)
Leukemia	Male	0-6 days	95+ years	3	-1	Education (years per capita)
Leukemia	Male	0-6 days	95+ years	3	1	LDI (I$ per capita)
Leukemia	Male	0-6 days	95+ years	2	1	Mean BMI
Leukemia	Male	0-6 days	95+ years	1	1	Log-transformed SEV scalar: Leukemia
Leukemia	Male	0-6 days	95+ years	1	1	Log-transformed age-standardized SEV scalar: Leukemia
Leukemia	Male	0-6 days	95+ years	3	-1	Socio-demographic Index
Leukemia	Male	0-6 days	95+ years	2	-1	Healthcare access and quality index
Leukemia	Male	0-6 days	95+ years	2	1	Liters of alcohol consumed per capita
Leukemia	Female	0-6 days	95+ years	2	1	Tobacco (cigarettes per capita)
Leukemia	Female	0-6 days	95+ years	2	1	Cumulative cigarettes (10 years)
Leukemia	Female	0-6 days	95+ years	2	1	Cumulative cigarettes (20 years)
Leukemia	Female	0-6 days	95+ years	3	-1	Education (years per capita)
Leukemia	Female	0-6 days	95+ years	3	1	LDI (I$ per capita)
Leukemia	Female	0-6 days	95+ years	2	1	Mean BMI
Leukemia	Female	0-6 days	95+ years	1	1	Log-transformed SEV scalar: Leukemia
Leukemia	Female	0-6 days	95+ years	1	1	Log-transformed age-standardized SEV scalar: Leukemia

Leukemia	Female	0-6 days	95+ years	3	-1	Socio-demographic Index
Leukemia	Female	0-6 days	95+ years	2	-1	Healthcare access and quality index
Leukemia	Female	0-6 days	95+ years	2	1	Liters of alcohol consumed per capita
Lip and oral cavity cancer	Male	5-9 years	95+ years	1	1	Tobacco (cigarettes per capita)
Lip and oral cavity cancer	Male	5-9 years	95+ years	1	1	Cumulative cigarettes (10 years)
Lip and oral cavity cancer	Male	5-9 years	95+ years	1	1	Cumulative cigarettes (20 years)
Lip and oral cavity cancer	Male	5-9 years	95+ years	3	-1	Education (years per capita)
Lip and oral cavity cancer	Male	5-9 years	95+ years	3	1	LDI (I$ per capita)
Lip and oral cavity cancer	Male	5-9 years	95+ years	1	1	Log-transformed SEV scalar: Lip oral C
Lip and oral cavity cancer	Male	5-9 years	95+ years	3	1	Socio-demographic Index
Lip and oral cavity cancer	Male	5-9 years	95+ years	2	-1	Healthcare access and quality index
Lip and oral cavity cancer	Male	5-9 years	95+ years	2	1	Age- and sex- specific SEV for low fruit
Lip and oral cavity cancer	Male	5-9 years	95+ years	2	1	Age- and sex- specific SEV for Low vegetables
Lip and oral cavity cancer	Male	5-9 years	95+ years	2	1	Age- and sex- specific SEV for High red meat
Lip and oral cavity cancer	Male	5-9 years	95+ years	1	1	Liters of alcohol consumed per capita
Lip and oral cavity cancer	Female	5-9 years	95+ years	1	1	Tobacco (cigarettes per capita)
Lip and oral cavity cancer	Female	5-9 years	95+ years	1	1	Cumulative cigarettes (10 years)
Lip and oral cavity cancer	Female	5-9 years	95+ years	1	1	Cumulative cigarettes (20 years)
Lip and oral cavity cancer	Female	5-9 years	95+ years	3	-1	Education (years per capita)
Lip and oral cavity cancer	Female	5-9 years	95+ years	3	1	LDI (I$ per capita)
Lip and oral cavity cancer	Female	5-9 years	95+ years	1	1	Log-transformed SEV scalar: Lip oral C
Lip and oral cavity cancer	Female	5-9 years	95+ years	3	1	Socio-demographic Index
Lip and oral cavity cancer	Female	5-9 years	95+ years	2	-1	Healthcare access and quality index
Lip and oral cavity cancer	Female	5-9 years	95+ years	2	1	Age- and sex- specific SEV for low fruit
Lip and oral cavity cancer	Female	5-9 years	95+ years	2	1	Age- and sex- specific SEV for Low vegetables
Lip and oral cavity cancer	Female	5-9 years	95+ years	2	1	Age- and sex- specific SEV for High red meat
Lip and oral cavity cancer	Female	5-9 years	95+ years	1	1	Liters of alcohol consumed per capita
Liver cancer	Male	0-6 days	95+ years	2	1	Tobacco (cigarettes per capita)
Liver cancer	Male	0-6 days	95+ years	2	1	Cumulative cigarettes (20 years)

Liver cancer	Male	0-6 days	95+ years	2	1	Diabetes Fasting Plasma Glucose (mmol/L), age-standardized 25+
Liver cancer	Male	0-6 days	95+ years	3	-1	Education (years per capita)
Liver cancer	Male	0-6 days	95+ years	3	-1	LDI (I$ per capita)
Liver cancer	Male	0-6 days	95+ years	2	1	Mean BMI
Liver cancer	Male	0-6 days	95+ years	1	1	Log-transformed SEV scalar: Liver C
Liver cancer	Male	0-6 days	95+ years	3	-1	Socio-demographic Index
Liver cancer	Male	0-6 days	95+ years	1	1	HIV age-standardized prevalence
Liver cancer	Male	0-6 days	95+ years	2	-1	Healthcare access and quality index
Liver cancer	Male	0-6 days	95+ years	2	-1	Hepatitis B 3-dose coverage (proportion)
Liver cancer	Male	0-6 days	95+ years	2	1	Intravenous drug use (age-standardized proportion)
Liver cancer	Male	0-6 days	95+ years	2	-1	Hepatitis B vaccine coverage (proportion), aged through time
Liver cancer	Male	0-6 days	95+ years	3	1	Age- and sex-specific SEV for High red meat
Liver cancer	Male	0-6 days	95+ years	1	1	Hepatitis B Seroprevalence (HBsAg) age standardized
Liver cancer	Male	0-6 days	95+ years	1	1	Hepatitis C Seroprevalence (anti-HCV) age standardized
Liver cancer	Male	0-6 days	95+ years	1	1	Liters of alcohol consumed per capita
Liver cancer	Female	0-6 days	95+ years	2	1	Tobacco (cigarettes per capita)
Liver cancer	Female	0-6 days	95+ years	2	1	Cumulative cigarettes (20 years)
Liver cancer	Female	0-6 days	95+ years	2	1	Diabetes Fasting Plasma Glucose (mmol/L), age-standardized 25+
Liver cancer	Female	0-6 days	95+ years	3	-1	Education (years per capita)
Liver cancer	Female	0-6 days	95+ years	3	-1	LDI (I$ per capita)
Liver cancer	Female	0-6 days	95+ years	2	1	Mean BMI
Liver cancer	Female	0-6 days	95+ years	1	1	Log-transformed SEV scalar: Liver C
Liver cancer	Female	0-6 days	95+ years	3	-1	Socio-demographic Index
Liver cancer	Female	0-6 days	95+ years	1	1	HIV age-standardized prevalence
Liver cancer	Female	0-6 days	95+ years	2	-1	Healthcare access and quality index
Liver cancer	Female	0-6 days	95+ years	2	-1	Hepatitis B 3-dose coverage (proportion)
Liver cancer	Female	0-6 days	95+ years	2	1	Intravenous drug use (age-standardized proportion)

Liver cancer	Female	0-6 days	95+ years	2	-1	Hepatitis B vaccine coverage (proportion), aged through time
Liver cancer	Female	0-6 days	95+ years	3	1	Age- and sex-specific SEV for High red meat
Liver cancer	Female	0-6 days	95+ years	1	1	Hepatitis B Seroprevalence (HBsAg) age standardized
Liver cancer	Female	0-6 days	95+ years	1	1	Hepatitis C Seroprevalence (anti-HCV) age standardized
Liver cancer	Female	0-6 days	95+ years	1	1	Liters of alcohol consumed per capita
Malignant skin melanoma	Male	0-6 days	95+ years	3	-1	Education (years per capita)
Malignant skin melanoma	Male	0-6 days	95+ years	3	-1	LDI (I$ per capita)
Malignant skin melanoma	Male	0-6 days	95+ years	2	-1	Latitude Under 15 (proportion)
Malignant skin melanoma	Male	0-6 days	95+ years	2	-1	Latitude 15 to 30 (proportion)
Malignant skin melanoma	Male	0-6 days	95+ years	2	-1	Latitude 30 to 45 (proportion)
Malignant skin melanoma	Male	0-6 days	95+ years	2	-1	Latitude Over 45 (proportion)
Malignant skin melanoma	Male	0-6 days	95+ years	3	1	Socio-demographic Index
Malignant skin melanoma	Male	0-6 days	95+ years	2	-1	Healthcare access and quality index
Malignant skin melanoma	Male	0-6 days	95+ years	1	1	Liters of alcohol consumed per capita
Malignant skin melanoma	Female	0-6 days	95+ years	3	-1	Education (years per capita)
Malignant skin melanoma	Female	0-6 days	95+ years	3	-1	LDI (I$ per capita)
Malignant skin melanoma	Female	0-6 days	95+ years	2	-1	Latitude Under 15 (proportion)
Malignant skin melanoma	Female	0-6 days	95+ years	2	-1	Latitude 15 to 30 (proportion)
Malignant skin melanoma	Female	0-6 days	95+ years	2	-1	Latitude 30 to 45 (proportion)
Malignant skin melanoma	Female	0-6 days	95+ years	2	-1	Latitude Over 45 (proportion)
Malignant skin melanoma	Female	0-6 days	95+ years	3	1	Socio-demographic Index
Malignant skin melanoma	Female	0-6 days	95+ years	2	-1	Healthcare access and quality index
Malignant skin melanoma	Female	0-6 days	95+ years	1	1	Liters of alcohol consumed per capita
Mesothelioma	Male	20-24 years	95+ years	2	1	Cumulative Cigarettes (5 Years)
Mesothelioma	Male	20-24 years	95+ years	3	-1	Education (years per capita)
Mesothelioma	Male	20-24 years	95+ years	2	1	Gold production (binary)
Mesothelioma	Male	20-24 years	95+ years	3	-1	LDI (I$ per capita)
Mesothelioma	Male	20-24 years	95+ years	2	1	Indoor Air Pollution (All Cooking Fuels)
Mesothelioma	Male	20-24 years	95+ years	2	1	Population Density (over 1000 ppl/sqkm, proportion)

Mesothelioma	Male	20-24 years	95+ years	1	1	Smoking Prevalence
Mesothelioma	Male	20-24 years	95+ years	1	1	Log-transformed SEV scalar: Mesothel
Mesothelioma	Male	20-24 years	95+ years	1	1	Log-transformed age-standardized SEV scalar: Mesothel
Mesothelioma	Male	20-24 years	95+ years	3	1	Socio-demographic Index
Mesothelioma	Male	20-24 years	95+ years	2	-1	Healthcare access and quality index
Mesothelioma	Male	20-24 years	95+ years	1	1	Asbestos consumption (metric tons per year per capita)
Mesothelioma	Female	20-24 years	95+ years	2	1	Cumulative Cigarettes (5 Years)
Mesothelioma	Female	20-24 years	95+ years	3	-1	Education (years per capita)
Mesothelioma	Female	20-24 years	95+ years	2	1	Gold production (binary)
Mesothelioma	Female	20-24 years	95+ years	3	-1	LDI (I$ per capita)
Mesothelioma	Female	20-24 years	95+ years	2	1	Indoor Air Pollution (All Cooking Fuels)
Mesothelioma	Female	20-24 years	95+ years	2	1	Population Density (over 1000 ppl/sqkm, proportion)
Mesothelioma	Female	20-24 years	95+ years	1	1	Smoking Prevalence
Mesothelioma	Female	20-24 years	95+ years	3	1	Socio-demographic Index
Mesothelioma	Female	20-24 years	95+ years	2	-1	Healthcare access and quality index
Mesothelioma	Female	20-24 years	95+ years	1	1	Asbestos consumption (metric tons per year per capita)
Multiple myeloma	Male	20-24 years	95+ years	1	1	Tobacco (cigarettes per capita)
Multiple myeloma	Male	20-24 years	95+ years	3	-1	Education (years per capita)
Multiple myeloma	Male	20-24 years	95+ years	3	1	LDI (I$ per capita)
Multiple myeloma	Male	20-24 years	95+ years	2	1	Mean BMI
Multiple myeloma	Male	20-24 years	95+ years	2	-1	Sanitation (proportion with access)
Multiple myeloma	Male	20-24 years	95+ years	1	1	Smoking Prevalence
Multiple myeloma	Male	20-24 years	95+ years	2	-1	Improved Water Source (proportion with access)
Multiple myeloma	Male	20-24 years	95+ years	3	1	Socio-demographic Index
Multiple myeloma	Male	20-24 years	95+ years	2	-1	Healthcare access and quality index
Multiple myeloma	Male	20-24 years	95+ years	2	1	Age- and sex- specific SEV for low fruit
Multiple myeloma	Male	20-24 years	95+ years	2	1	Age- and sex- specific SEV for Low vegetables
Multiple myeloma	Male	20-24 years	95+ years	2	1	Age- and sex-specific SEV for High red meat
Multiple myeloma	Male	20-24 years	95+ years	1	1	Liters of alcohol consumed per capita
Multiple myeloma	Female	20-24 years	95+ years	1	1	Tobacco (cigarettes per capita)

Multiple myeloma	Female	20-24 years	95+ years	3	-1	Education (years per capita)
Multiple myeloma	Female	20-24 years	95+ years	3	1	LDI (I$ per capita)
Multiple myeloma	Female	20-24 years	95+ years	2	1	Mean BMI
Multiple myeloma	Female	20-24 years	95+ years	2	-1	Sanitation (proportion with access)
Multiple myeloma	Female	20-24 years	95+ years	1	1	Smoking Prevalence
Multiple myeloma	Female	20-24 years	95+ years	2	-1	Improved Water Source (proportion with access)
Multiple myeloma	Female	20-24 years	95+ years	3	1	Socio-demographic Index
Multiple myeloma	Female	20-24 years	95+ years	2	-1	Healthcare access and quality index
Multiple myeloma	Female	20-24 years	95+ years	2	1	Age- and sex- specific SEV for low fruit
Multiple myeloma	Female	20-24 years	95+ years	2	1	Age- and sex- specific SEV for Low vegetables
Multiple myeloma	Female	20-24 years	95+ years	2	1	Age- and sex-specific SEV for High red meat
Multiple myeloma	Female	20-24 years	95+ years	1	1	Liters of alcohol consumed per capita
Nasopharynx cancer	Male	5-9 years	95+ years	1	1	Tobacco (cigarettes per capita)
Nasopharynx cancer	Male	5-9 years	95+ years	1	1	Cumulative cigarettes (10 years)
Nasopharynx cancer	Male	5-9 years	95+ years	1	1	Cumulative cigarettes (20 years)
Nasopharynx cancer	Male	5-9 years	95+ years	3	-1	Education (years per capita)
Nasopharynx cancer	Male	5-9 years	95+ years	3	-1	LDI (I$ per capita)
Nasopharynx cancer	Male	5-9 years	95+ years	2	1	Population Density (over 1000 ppl/sqkm, proportion)
Nasopharynx cancer	Male	5-9 years	95+ years	1	1	Log-transformed SEV scalar: Nasoph C
Nasopharynx cancer	Male	5-9 years	95+ years	3	1	Socio-demographic Index
Nasopharynx cancer	Male	5-9 years	95+ years	2	-1	Healthcare access and quality index
Nasopharynx cancer	Male	5-9 years	95+ years	3	1	Age- and sex- specific SEV for low fruit
Nasopharynx cancer	Male	5-9 years	95+ years	2	1	Age- and sex- specific SEV for Low vegetables
Nasopharynx cancer	Male	5-9 years	95+ years	1	1	Liters of alcohol consumed per capita
Nasopharynx cancer	Female	5-9 years	95+ years	1	1	Tobacco (cigarettes per capita)
Nasopharynx cancer	Female	5-9 years	95+ years	1	1	Cumulative cigarettes (10 years)
Nasopharynx cancer	Female	5-9 years	95+ years	1	1	Cumulative cigarettes (20 years)
Nasopharynx cancer	Female	5-9 years	95+ years	3	-1	Education (years per capita)
Nasopharynx cancer	Female	5-9 years	95+ years	3	-1	LDI (I$ per capita)
Nasopharynx cancer	Female	5-9 years	95+ years	2	1	Population Density (over 1000 ppl/sqkm, proportion)

Nasopharynx cancer	Female	5-9 years	95+ years	1	1	Log-transformed SEV scalar: Nasoph C
Nasopharynx cancer	Female	5-9 years	95+ years	3	1	Socio-demographic Index
Nasopharynx cancer	Female	5-9 years	95+ years	2	-1	Healthcare access and quality index
Nasopharynx cancer	Female	5-9 years	95+ years	3	1	Age- and sex- specific SEV for low fruit
Nasopharynx cancer	Female	5-9 years	95+ years	2	1	Age- and sex- specific SEV for Low vegetables
Nasopharynx cancer	Female	5-9 years	95+ years	1	1	Liters of alcohol consumed per capita
Non-Hodgkin lymphoma	Male	1-4 years	95+ years	2	1	Cumulative cigarettes (10 years)
Non-Hodgkin lymphoma	Male	1-4 years	95+ years	2	1	Cumulative cigarettes (15 years)
Non-Hodgkin lymphoma	Male	1-4 years	95+ years	2	1	Cumulative cigarettes (20 years)
Non-Hodgkin lymphoma	Male	1-4 years	95+ years	2	1	Cumulative Cigarettes (5 Years)
Non-Hodgkin lymphoma	Male	1-4 years	95+ years	3	1	LDI (I$ per capita)
Non-Hodgkin lymphoma	Male	1-4 years	95+ years	2	1	Mean BMI
Non-Hodgkin lymphoma	Male	1-4 years	95+ years	2	1	Smoking Prevalence
Non-Hodgkin lymphoma	Male	1-4 years	95+ years	3	1	Socio-demographic Index
Non-Hodgkin lymphoma	Male	1-4 years	95+ years	2	-1	Healthcare access and quality index
Non-Hodgkin lymphoma	Male	1-4 years	95+ years	2	1	Liters of alcohol consumed per capita
Non-Hodgkin lymphoma	Female	1-4 years	95+ years	2	1	Cumulative cigarettes (10 years)
Non-Hodgkin lymphoma	Female	1-4 years	95+ years	2	1	Cumulative cigarettes (15 years)
Non-Hodgkin lymphoma	Female	1-4 years	95+ years	2	1	Cumulative cigarettes (20 years)
Non-Hodgkin lymphoma	Female	1-4 years	95+ years	2	1	Cumulative Cigarettes (5 Years)
Non-Hodgkin lymphoma	Female	1-4 years	95+ years	3	1	LDI (I$ per capita)
Non-Hodgkin lymphoma	Female	1-4 years	95+ years	2	1	Mean BMI
Non-Hodgkin lymphoma	Female	1-4 years	95+ years	2	1	Smoking Prevalence
Non-Hodgkin lymphoma	Female	1-4 years	95+ years	3	-1	Total Fertility Rate
Non-Hodgkin lymphoma	Female	1-4 years	95+ years	3	1	Socio-demographic Index
Non-Hodgkin lymphoma	Female	1-4 years	95+ years	2	-1	Healthcare access and quality index
Non-Hodgkin lymphoma	Female	1-4 years	95+ years	2	1	Liters of alcohol consumed per capita
Non-melanoma skin cancer	Male	20-24 years	95+ years	1	1	Cumulative cigarettes (10 years)
Non-melanoma skin cancer	Male	20-24 years	95+ years	1	1	Cumulative cigarettes (15 years)
Non-melanoma skin cancer	Male	20-24 years	95+ years	1	1	Cumulative cigarettes (5 years)

Non-melanoma skin cancer	Male	20-24 years	95+ years	3	-1	Education (years per capita)
Non-melanoma skin cancer	Male	20-24 years	95+ years	2	-1	Average latitude
Non-melanoma skin cancer	Male	20-24 years	95+ years	3	-1	LDI (I$ per capita)
Non-melanoma skin cancer	Male	20-24 years	95+ years	1	1	Smoking Prevalence
Non-melanoma skin cancer	Male	20-24 years	95+ years	3	1	Socio-demographic Index
Non-melanoma skin cancer	Male	20-24 years	95+ years	2	-1	Healthcare access and quality index
Non-melanoma skin cancer	Female	20-24 years	95+ years	1	1	Cumulative cigarettes (10 years)
Non-melanoma skin cancer	Female	20-24 years	95+ years	1	1	Cumulative cigarettes (15 years)
Non-melanoma skin cancer	Female	20-24 years	95+ years	1	1	Cumulative cigarettes (5 years)
Non-melanoma skin cancer	Female	20-24 years	95+ years	3	-1	Education (years per capita)
Non-melanoma skin cancer	Female	20-24 years	95+ years	2	-1	Average latitude
Non-melanoma skin cancer	Female	20-24 years	95+ years	3	-1	LDI (I$ per capita)
Non-melanoma skin cancer	Female	20-24 years	95+ years	1	1	Smoking Prevalence
Non-melanoma skin cancer	Female	20-24 years	95+ years	3	1	Socio-demographic index
Non-melanoma skin cancer	Female	20-24 years	95+ years	2	-1	Healthcare access and quality index
Other leukemia	Male	0-6 days	95+ years	2	1	Tobacco (cigarettes per capita)
Other leukemia	Male	0-6 days	95+ years	2	1	Cumulative cigarettes (10 years)
Other leukemia	Male	0-6 days	95+ years	2	1	Cumulative cigarettes (20 years)
Other leukemia	Male	0-6 days	95+ years	3	-1	Education (years per capita)
Other leukemia	Male	0-6 days	95+ years	3	1	LDI (I$ per capita)
Other leukemia	Male	0-6 days	95+ years	2	1	Mean BMI
Other leukemia	Male	0-6 days	95+ years	1	1	Log-transformed SEV scalar: Leukemia
Other leukemia	Male	0-6 days	95+ years	1	1	Log-transformed age-standardized SEV scalar: Leukemia
Other leukemia	Male	0-6 days	95+ years	3	-1	Socio-demographic Index
Other leukemia	Male	0-6 days	95+ years	2	-1	Healthcare access and quality index
Other leukemia	Male	0-6 days	95+ years	2	1	Liters of alcohol consumed per capita
Other leukemia	Female	0-6 days	95+ years	2	1	Tobacco (cigarettes per capita)
Other leukemia	Female	0-6 days	95+ years	2	1	Cumulative cigarettes (10 years)
Other leukemia	Female	0-6 days	95+ years	2	1	Cumulative cigarettes (20 years)
Other leukemia	Female	0-6 days	95+ years	3	-1	Education (years per capita)

Other leukemia	Female	0-6 days	95+ years	3	1	LDI (I$ per capita)
Other leukemia	Female	0-6 days	95+ years	2	1	Mean BMI
Other leukemia	Female	0-6 days	95+ years	1	1	Log-transformed SEV scalar: Leukemia
Other leukemia	Female	0-6 days	95+ years	1	1	Log-transformed age-standardized SEV scalar: Leukemia
Other leukemia	Female	0-6 days	95+ years	3	-1	Socio-demographic Index
Other leukemia	Female	0-6 days	95+ years	2	-1	Healthcare access and quality index
Other leukemia (dr)	Female	0-6 days	95+ years	1	1	Liters of alcohol consumed per capita
Other leukemia (glb)	Female	0-6 days	95+ years	2	1	Liters of alcohol consumed per capita
Other malignant neoplasms	Male	0-6 days	95+ years	1	1	Tobacco (cigarettes per capita)
Other malignant neoplasms	Male	0-6 days	95+ years	3	-1	Education (years per capita)
Other malignant neoplasms	Male	0-6 days	95+ years	3	1	LDI (I$ per capita)
Other malignant neoplasms	Male	0-6 days	95+ years	1	1	Smoking Prevalence
Other malignant neoplasms	Male	0-6 days	95+ years	3	1	Socio-demographic Index
Other malignant neoplasms	Male	0-6 days	95+ years	2	-1	PUFA adjusted(percent)
Other malignant neoplasms	Male	0-6 days	95+ years	2	-1	Healthcare access and quality index
Other malignant neoplasms	Male	0-6 days	95+ years	2	1	Age- and sex- specific SEV for low fruit
Other malignant neoplasms	Male	0-6 days	95+ years	2	1	Age- and sex- specific SEV for Low vegetables
Other malignant neoplasms	Male	0-6 days	95+ years	2	1	Age- and sex-specific SEV for Low nuts and seeds
Other malignant neoplasms	Female	0-6 days	95+ years	1	1	Tobacco (cigarettes per capita)
Other malignant neoplasms	Female	0-6 days	95+ years	3	-1	Education (years per capita)
Other malignant neoplasms	Female	0-6 days	95+ years	3	1	LDI (I$ per capita)
Other malignant neoplasms	Female	0-6 days	95+ years	1	1	Smoking Prevalence
Other malignant neoplasms	Female	0-6 days	95+ years	3	1	Socio-demographic Index
Other malignant neoplasms	Female	0-6 days	95+ years	2	-1	PUFA adjusted(percent)
Other malignant neoplasms	Female	0-6 days	95+ years	2	-1	Healthcare access and quality index
Other malignant neoplasms	Female	0-6 days	95+ years	2	1	Age- and sex- specific SEV for low fruit
Other malignant neoplasms	Female	0-6 days	95+ years	2	1	Age- and sex- specific SEV for Low vegetables
Other malignant neoplasms	Female	0-6 days	95+ years	2	1	Age- and sex-specific SEV for Low nuts and seeds
Other pharynx cancer	Male	20-24 years	95+ years	2	1	Cumulative cigarettes (5 years)
Other pharynx cancer	Male	20-24 years	95+ years	3	-1	Education (years per capita)

Other pharynx cancer	Male	20-24 years	95+ years	3	1	LDI (I$ per capita)
Other pharynx cancer	Male	20-24 years	95+ years	2	1	Population Density (over 1000 ppl/sqkm, proportion)
Other pharynx cancer	Male	20-24 years	95+ years	2	1	Population Density (under 150 ppl/sqkm, proportion)
Other pharynx cancer	Male	20-24 years	95+ years	1	1	Smoking Prevalence
Other pharynx cancer	Male	20-24 years	95+ years	1	1	Log-transformed SEV scalar: Oth Phar C
Other pharynx cancer	Male	20-24 years	95+ years	3	1	Socio-demographic Index
Other pharynx cancer	Male	20-24 years	95+ years	2	-1	Healthcare access and quality index
Other pharynx cancer	Male	20-24 years	95+ years	2	1	Age- and sex- specific SEV for low fruit
Other pharynx cancer	Male	20-24 years	95+ years	2	1	Age- and sex- specific SEV for Low vegetables
Other pharynx cancer	Male	20-24 years	95+ years	1	1	Liters of alcohol consumed per capita
Other pharynx cancer	Female	20-24 years	95+ years	2	1	Cumulative cigarettes (5 years)
Other pharynx cancer	Female	20-24 years	95+ years	3	-1	Education (years per capita)
Other pharynx cancer	Female	20-24 years	95+ years	3	1	LDI (I$ per capita)
Other pharynx cancer	Female	20-24 years	95+ years	2	1	Population Density (over 1000 ppl/sqkm, proportion)
Other pharynx cancer	Female	20-24 years	95+ years	2	1	Population Density (under 150 ppl/sqkm, proportion)
Other pharynx cancer	Female	20-24 years	95+ years	1	1	Smoking Prevalence
Other pharynx cancer	Female	20-24 years	95+ years	1	1	Log-transformed SEV scalar: Oth Phar C
Other pharynx cancer	Female	20-24 years	95+ years	3	1	Socio-demographic Index
Other pharynx cancer	Female	20-24 years	95+ years	2	-1	Healthcare access and quality index
Other pharynx cancer	Female	20-24 years	95+ years	2	1	Age- and sex- specific SEV for low fruit
Other pharynx cancer	Female	20-24 years	95+ years	2	1	Age- and sex- specific SEV for Low vegetables
Other pharynx cancer	Female	20-24 years	95+ years	1	1	Liters of alcohol consumed per capita
Ovarian cancer	Female	5-9 years	95+ years	2	-1	Contraception (Modern) Prevalence (proportion)
Ovarian cancer	Female	5-9 years	95+ years	2	1	Cumulative cigarettes (10 years)
Ovarian cancer	Female	5-9 years	95+ years	2	1	Cumulative cigarettes (20 years)
Ovarian cancer	Female	5-9 years	95+ years	2	1	Diabetes Age-Standardized Prevalence (proportion)
Ovarian cancer	Female	5-9 years	95+ years	3	-1	Education (years per capita)
Ovarian cancer	Female	5-9 years	95+ years	3	-1	LDI (I$ per capita)
Ovarian cancer	Female	5-9 years	95+ years	2	1	Mean BMI
Ovarian cancer	Female	5-9 years	95+ years	2	1	Smoking Prevalence

Ovarian cancer	Female	5-9 years	95+ years	2	-1	Total Fertility Rate
Ovarian cancer	Female	5-9 years	95+ years	1	1	Log-transformed SEV scalar: Ovary C
Ovarian cancer	Female	5-9 years	95+ years	3	1	Socio-demographic Index
Ovarian cancer	Female	5-9 years	95+ years	2	1	energy unadjusted(kcal)
Ovarian cancer	Female	5-9 years	95+ years	2	-1	Healthcare access and quality index
Ovarian cancer	Female	5-9 years	95+ years	2	1	Asbestos consumption (metric tons per year per capita)
Ovarian cancer	Female	5-9 years	95+ years	3	1	Age- and sex- specific SEV for low fruit
Ovarian cancer	Female	5-9 years	95+ years	3	1	Age- and sex- specific SEV for Low vegetables
Ovarian cancer	Female	5-9 years	95+ years	1	1	Liters of alcohol consumed per capita
Pancreatic cancer	Male	15-19 years	95+ years	1	1	Tobacco (cigarettes per capita)
Pancreatic cancer	Male	15-19 years	95+ years	1	1	Cumulative cigarettes (10 years)
Pancreatic cancer	Male	15-19 years	95+ years	1	1	Cumulative cigarettes (20 years)
Pancreatic cancer	Male	15-19 years	95+ years	2	1	Diabetes Fasting Plasma Glucose (mmol/L), age-standardized 25+
Pancreatic cancer	Male	15-19 years	95+ years	2	1	Diabetes Age-Standardized Prevalence (proportion)
Pancreatic cancer	Male	15-19 years	95+ years	3	-1	Education (years per capita)
Pancreatic cancer	Male	15-19 years	95+ years	3	1	LDI (I$ per capita)
Pancreatic cancer	Male	15-19 years	95+ years	1	1	Mean BMI
Pancreatic cancer	Male	15-19 years	95+ years	1	1	Log-transformed SEV scalar: Pancreas C
Pancreatic cancer	Male	15-19 years	95+ years	3	1	Socio-demographic Index
Pancreatic cancer	Male	15-19 years	95+ years	2	1	energy unadjusted(kcal)
Pancreatic cancer	Male	15-19 years	95+ years	2	-1	Healthcare access and quality index
Pancreatic cancer	Male	15-19 years	95+ years	3	1	Age- and sex- specific SEV for low fruit
Pancreatic cancer	Male	15-19 years	95+ years	3	1	Age- and sex- specific SEV for Low vegetables
Pancreatic cancer	Male	15-19 years	95+ years	2	1	Age- and sex-specific SEV for High red meat
Pancreatic cancer	Male	15-19 years	95+ years	2	1	Liters of alcohol consumed per capita
Pancreatic cancer	Female	15-19 years	95+ years	1	1	Tobacco (cigarettes per capita)
Pancreatic cancer	Female	15-19 years	95+ years	1	1	Cumulative cigarettes (10 years)
Pancreatic cancer	Female	15-19 years	95+ years	1	1	Cumulative cigarettes (20 years)

Pancreatic cancer	Female	15-19 years	95+ years	2	1	Diabetes Fasting Plasma Glucose (mmol/L), age-standardized 25+
Pancreatic cancer	Female	15-19 years	95+ years	2	1	Diabetes Age-Standardized Prevalence (proportion)
Pancreatic cancer	Female	15-19 years	95+ years	3	-1	Education (years per capita)
Pancreatic cancer	Female	15-19 years	95+ years	3	1	LDI (I$ per capita)
Pancreatic cancer	Female	15-19 years	95+ years	1	1	Mean BMI
Pancreatic cancer	Female	15-19 years	95+ years	1	1	Log-transformed SEV scalar: Pancreas C
Pancreatic cancer	Female	15-19 years	95+ years	3	1	Socio-demographic Index
Pancreatic cancer	Female	15-19 years	95+ years	2	1	energy unadjusted(kcal)
Pancreatic cancer	Female	15-19 years	95+ years	2	-1	Healthcare access and quality index
Pancreatic cancer	Female	15-19 years	95+ years	3	1	Age- and sex- specific SEV for low fruit
Pancreatic cancer	Female	15-19 years	95+ years	3	1	Age- and sex- specific SEV for Low vegetables
Pancreatic cancer	Female	15-19 years	95+ years	2	1	Age- and sex-specific SEV for High red meat
Pancreatic cancer	Female	15-19 years	95+ years	2	1	Liters of alcohol consumed per capita
Prostate cancer	Male	20-24 years	95+ years	3	-1	Education (years per capita)
Prostate cancer	Male	20-24 years	95+ years	3	-1	LDI (I$ per capita)
Prostate cancer	Male	20-24 years	95+ years	2	1	Smoking Prevalence
Prostate cancer	Male	20-24 years	95+ years	1	1	Log-transformed SEV scalar: Prostate C
Prostate cancer	Male	20-24 years	95+ years	3	1	Socio-demographic Index
Prostate cancer	Male	20-24 years	95+ years	2	-1	Healthcare access and quality index
Stomach cancer	Male	15-19 years	95+ years	1	1	Tobacco (cigarettes per capita)
Stomach cancer	Male	15-19 years	95+ years	2	1	Cumulative cigarettes (20 years)
Stomach cancer	Male	15-19 years	95+ years	3	-1	Education (years per capita)
Stomach cancer	Male	15-19 years	95+ years	3	1	LDI (I$ per capita)
Stomach cancer	Male	15-19 years	95+ years	2	1	Mean BMI
Stomach cancer	Male	15-19 years	95+ years	2	-1	Sanitation (proportion with access)
Stomach cancer	Male	15-19 years	95+ years	2	-1	Improved Water Source (proportion with access)
Stomach cancer	Male	15-19 years	95+ years	1	1	Log-transformed SEV scalar: Stomach C
Stomach cancer	Male	15-19 years	95+ years	2	1	Age- and sex-specific SEV for Unsafe water
Stomach cancer	Male	15-19 years	95+ years	2	1	Age- and sex-specific SEV for Unsafe sanitation

Stomach cancer	Male	15-19 years	95+ years	3	-1	Socio-demographic Index
Stomach cancer	Male	15-19 years	95+ years	2	-1	Healthcare access and quality index
Stomach cancer	Male	15-19 years	95+ years	1	1	Diet high in sodium
Stomach cancer	Male	15-19 years	95+ years	3	1	Age- and sex- specific SEV for low fruit
Stomach cancer	Male	15-19 years	95+ years	3	1	Age- and sex- specific SEV for Low vegetables
Stomach cancer	Female	15-19 years	95+ years	1	1	Tobacco (cigarettes per capita)
Stomach cancer	Female	15-19 years	95+ years	2	1	Cumulative cigarettes (20 years)
Stomach cancer	Female	15-19 years	95+ years	3	-1	Education (years per capita)
Stomach cancer	Female	15-19 years	95+ years	3	1	LDI (I$ per capita)
Stomach cancer	Female	15-19 years	95+ years	2	1	Mean BMI
Stomach cancer	Female	15-19 years	95+ years	2	-1	Sanitation (proportion with access)
Stomach cancer	Female	15-19 years	95+ years	2	-1	Improved Water Source (proportion with access)
Stomach cancer	Female	15-19 years	95+ years	1	1	Log-transformed SEV scalar: Stomach C
Stomach cancer	Female	15-19 years	95+ years	2	1	Age- and sex-specific SEV for Unsafe water
Stomach cancer	Female	15-19 years	95+ years	2	1	Age- and sex-specific SEV for Unsafe sanitation
Stomach cancer	Female	15-19 years	95+ years	3	-1	Socio-demographic Index
Stomach cancer	Female	15-19 years	95+ years	2	-1	Healthcare access and quality index
Stomach cancer	Female	15-19 years	95+ years	1	1	Diet high in sodium
Stomach cancer	Female	15-19 years	95+ years	3	1	Age- and sex- specific SEV for low fruit
Stomach cancer	Female	15-19 years	95+ years	3	1	Age- and sex- specific SEV for Low vegetables
Testicular cancer	Male	0-6 days	95+ years	2	1	Tobacco (cigarettes per capita)
Testicular cancer	Male	0-6 days	95+ years	2	1	Cumulative cigarettes (10 years)
Testicular cancer	Male	0-6 days	95+ years	2	1	Cumulative Cigarettes (15 Years)
Testicular cancer	Male	0-6 days	95+ years	2	1	Cumulative cigarettes (20 years)
Testicular cancer	Male	0-6 days	95+ years	2	1	Cumulative Cigarettes (5 Years)
Testicular cancer	Male	0-6 days	95+ years	3	-1	Education (years per capita)
Testicular cancer	Male	0-6 days	95+ years	3	1	LDI (I$ per capita)
Testicular cancer	Male	0-6 days	95+ years	2	1	Smoking Prevalence
Testicular cancer	Male	0-6 days	95+ years	3	1	Socio-demographic Index
Testicular cancer	Male	0-6 days	95+ years	2	-1	Healthcare access and quality index

Testicular cancer	Male	0-6 days	95+ years	2	1	Age- and sex- specific SEV for low fruit
Testicular cancer	Male	0-6 days	95+ years	2	1	Age- and sex- specific SEV for Low vegetables
Thyroid cancer	Male	5-9 years	95+ years	2	1	Tobacco (cigarettes per capita)
Thyroid cancer	Male	5-9 years	95+ years	3	-1	Education (years per capita)
Thyroid cancer	Male	5-9 years	95+ years	3	1	LDI (I$ per capita)
Thyroid cancer	Male	5-9 years	95+ years	2	1	Mean BMI
Thyroid cancer	Male	5-9 years	95+ years	3	-1	Sanitation (proportion with access)
Thyroid cancer	Male	5-9 years	95+ years	3	-1	Improved Water Source (proportion with access)
Thyroid cancer	Male	5-9 years	95+ years	1	1	Log-transformed SEV scalar: Thyroid C
Thyroid cancer	Male	5-9 years	95+ years	3	1	Socio-demographic Index
Thyroid cancer	Male	5-9 years	95+ years	2	-1	Healthcare access and quality index
Thyroid cancer	Male	5-9 years	95+ years	3	1	Age- and sex- specific SEV for low fruit
Thyroid cancer	Male	5-9 years	95+ years	2	1	Age- and sex- specific SEV for Low vegetables
Thyroid cancer	Male	5-9 years	95+ years	2	1	Age- and sex-specific SEV for High red meat
Thyroid cancer	Male	5-9 years	95+ years	1	1	Liters of alcohol consumed per capita
Thyroid cancer	Female	5-9 years	95+ years	2	1	Tobacco (cigarettes per capita)
Thyroid cancer	Female	5-9 years	95+ years	3	-1	Education (years per capita)
Thyroid cancer	Female	5-9 years	95+ years	3	1	LDI (I$ per capita)
Thyroid cancer	Female	5-9 years	95+ years	2	1	Mean BMI
Thyroid cancer	Female	5-9 years	95+ years	3	-1	Sanitation (proportion with access)
Thyroid cancer	Female	5-9 years	95+ years	3	-1	Improved Water Source (proportion with access)
Thyroid cancer	Female	5-9 years	95+ years	1	1	Log-transformed SEV scalar: Thyroid C
Thyroid cancer	Female	5-9 years	95+ years	3	1	Socio-demographic Index
Thyroid cancer	Female	5-9 years	95+ years	2	-1	Healthcare access and quality index
Thyroid cancer	Female	5-9 years	95+ years	3	1	Age- and sex- specific SEV for low fruit
Thyroid cancer	Female	5-9 years	95+ years	2	1	Age- and sex- specific SEV for Low vegetables
Thyroid cancer	Female	5-9 years	95+ years	2	1	Age- and sex-specific SEV for High red meat
Thyroid cancer	Female	5-9 years	95+ years	1	1	Liters of alcohol consumed per capita
Tracheal, bronchus, and lung cancer	Male	10-14 year	95+ years	2	1	Cumulative cigarettes (10 years)
Tracheal, bronchus, and lung cancer	Male	10-14 year	95+ years	2	1	Cumulative cigarettes (20 years)

Tracheal, bronchus, and lung cancer	Male	10-14 year	95+ years	2	1	Diabetes Fasting Plasma Glucose (mmol/L), age-standardized 25+
Tracheal, bronchus, and lung cancer	Male	10-14 year	95+ years	3	-1	Education (years per capita)
Tracheal, bronchus, and lung cancer	Male	10-14 year	95+ years	3	1	LDI (I$ per capita)
Tracheal, bronchus, and lung cancer	Male	10-14 year	95+ years	2	1	Indoor Air Pollution (All Cooking Fuels)
Tracheal, bronchus, and lung cancer	Male	10-14 year	95+ years	2	1	Outdoor Air Pollution (PM2.5)
Tracheal, bronchus, and lung cancer	Male	10-14 year	95+ years	1	1	Smoking Prevalence
Tracheal, bronchus, and lung cancer	Male	10-14 year	95+ years	1	1	Log-transformed SEV scalar: Lung C
Tracheal, bronchus, and lung cancer	Male	10-14 year	95+ years	1	1	Log-transformed age-standardized SEV scalar: Lung C
Tracheal, bronchus, and lung cancer	Male	10-14 year	95+ years	3	1	Socio-demographic Index
Tracheal, bronchus, and lung cancer	Male	10-14 year	95+ years	2	-1	Healthcare access and quality index
Tracheal, bronchus, and lung cancer	Male	10-14 year	95+ years	2	1	Residential radon
Tracheal, bronchus, and lung cancer	Male	10-14 year	95+ years	2	1	Secondhand smoke
Tracheal, bronchus, and lung cancer	Male	10-14 year	95+ years	1	1	Asbestos consumption (metric tons per year per capita)
Tracheal, bronchus, and lung cancer	Female	10-14 year	95+ years	2	1	Cumulative cigarettes (10 years)
Tracheal, bronchus, and lung cancer	Female	10-14 year	95+ years	2	1	Cumulative cigarettes (20 years)
Tracheal, bronchus, and lung cancer	Female	10-14 year	95+ years	2	1	Diabetes Fasting Plasma Glucose (mmol/L), age-standardized 25+
Tracheal, bronchus, and lung cancer	Female	10-14 year	95+ years	3	-1	Education (years per capita)
Tracheal, bronchus, and lung cancer	Female	10-14 year	95+ years	3	1	LDI (I$ per capita)
Tracheal, bronchus, and lung cancer	Female	10-14 year	95+ years	2	1	Indoor Air Pollution (All Cooking Fuels)
Tracheal, bronchus, and lung cancer	Female	10-14 year	95+ years	2	1	Outdoor Air Pollution (PM2.5)
Tracheal, bronchus, and lung cancer	Female	10-14 year	95+ years	1	1	Smoking Prevalence
Tracheal, bronchus, and lung cancer	Female	10-14 year	95+ years	1	1	Log-transformed SEV scalar: Lung C
Tracheal, bronchus, and lung cancer	Female	10-14 year	95+ years	1	1	Log-transformed age-standardized SEV scalar: Lung C
Tracheal, bronchus, and lung cancer	Female	10-14 year	95+ years	3	1	Socio-demographic Index
Tracheal, bronchus, and lung cancer	Female	10-14 year	95+ years	2	-1	Healthcare access and quality index
Tracheal, bronchus, and lung cancer	Female	10-14 year	95+ years	2	1	Residential radon
Tracheal, bronchus, and lung cancer	Female	10-14 year	95+ years	2	1	Secondhand smoke
Tracheal, bronchus, and lung cancer	Female	10-14 year	95+ years	1	1	Asbestos consumption (metric tons per year per capita)

Uterine cancer	Female	20-24 years	95+ years	2	1	Tobacco (cigarettes per capita)
Uterine cancer	Female	20-24 years	95+ years	2	1	Cumulative cigarettes (10 years)
Uterine cancer	Female	20-24 years	95+ years	2	1	Cumulative Cigarettes (5 Years)
Uterine cancer	Female	20-24 years	95+ years	2	1	Diabetes Age-Standardized Prevalence (proportion)
Uterine cancer	Female	20-24 years	95+ years	3	-1	Education (years per capita)
Uterine cancer	Female	20-24 years	95+ years	3	1	LDI (I$ per capita)
Uterine cancer	Female	20-24 years	95+ years	1	1	Mean BMI
Uterine cancer	Female	20-24 years	95+ years	2	1	Smoking Prevalence
Uterine cancer	Female	20-24 years	95+ years	2	-1	Total Fertility Rate
Uterine cancer	Female	20-24 years	95+ years	1	1	Log-transformed SEV scalar: Uterus C
Uterine cancer	Female	20-24 years	95+ years	3	1	Socio-demographic Index
Uterine cancer	Female	20-24 years	95+ years	2	-1	Healthcare access and quality index
Uterine cancer	Female	20-24 years	95+ years	2	1	Age- and sex- specific SEV for low fruit
Uterine cancer	Female	20-24 years	95+ years	2	1	Age- and sex- specific SEV for Low vegetables

Abbreviations: BMI, body mass index; DR, data rich; GBD, Global Burden of Disease Study; GLB, global; HBsAg, hepatitis B surface antigen; HCV, hepatitis C virus; LDI, lag-distributed income; MET, metabolic equivalent of task; NA, not applicable; PM2.5, particulate matter ≤ 2.5 micrometers; PUFA, polyunsaturated fatty acid; SEV, summary exposure value.

Footnote: The “Direction” column refers to the direction of the association for that covariate.

eTable 8: Comparison of GBD 2017 and GBD 2019 covariates used and level of covariates

Cause	Covariate	Sex	Age start	Age end	Level in GBD 2017	Level in GBD 2019
Acute lymphoid leukemia	Cumulative Cigarettes (10 Years)	Female	0-6 days	95+ years	2	2
Acute lymphoid leukemia	Cumulative Cigarettes (10 Years)	Male	0-6 days	95+ years	2	2
Acute lymphoid leukemia	Cumulative Cigarettes (20 Years)	Female	0-6 days	95+ years	2	2
Acute lymphoid leukemia	Cumulative Cigarettes (20 Years)	Male	0-6 days	95+ years	2	2
Acute lymphoid leukemia	Cumulative Cigarettes (5 Years)	Female	0-6 days	95+ years	2	NA
Acute lymphoid leukemia	Cumulative Cigarettes (5 Years)	Male	0-6 days	95+ years	2	NA
Acute lymphoid leukemia	Cumulative Cigarettes (15 Years)	Female	0-6 days	95+ years	2	NA
Acute lymphoid leukemia	Cumulative Cigarettes (15 Years)	Male	0-6 days	95+ years	2	NA
Acute lymphoid leukemia	Smoking prevalence	Female	0-6 days	95+ years	2	NA
Acute lymphoid leukemia	Smoking prevalence	Male	0-6 days	95+ years	2	NA
Acute lymphoid leukemia	Education (years per capita)	Female	0-6 days	95+ years	3	3
Acute lymphoid leukemia	Education (years per capita)	Male	0-6 days	95+ years	3	3
Acute lymphoid leukemia	Healthcare access and quality index	Female	0-6 days	95+ years	NA	2
Acute lymphoid leukemia	Healthcare access and quality index	Male	0-6 days	95+ years	NA	2
Acute lymphoid leukemia	LDI (I$ per capita)	Female	0-6 days	95+ years	3	3
Acute lymphoid leukemia	LDI (I$ per capita)	Male	0-6 days	95+ years	3	3
Acute lymphoid leukemia	Liters of alcohol consumed per capita	Female	0-6 days	95+ years	2	2
Acute lymphoid leukemia	Liters of alcohol consumed per capita	Male	0-6 days	95+ years	2	2
Acute lymphoid leukemia	Log-transformed SEV scalar: Leukemia	Female	0-6 days	95+ years	1	1
Acute lymphoid leukemia	Log-transformed SEV scalar: Leukemia	Male	0-6 days	95+ years	1	1
Acute lymphoid leukemia	Log-transformed age-standardized SEV scalar: Leukemia	Female	0-6 days	95+ years	1	1
Acute lymphoid leukemia	Log-transformed age-standardized SEV scalar: Leukemia	Male	0-6 days	95+ years	1	1
Acute lymphoid leukemia	Mean BMI	Female	0-6 days	95+ years	NA	2
Acute lymphoid leukemia	Mean BMI	Male	0-6 days	95+ years	NA	2
Acute lymphoid leukemia	Socio-demographic Index	Female	0-6 days	95+ years	3	3

Acute lymphoid leukemia	Socio-demographic Index	Male	0-6 days	95+ years	3	3
Acute lymphoid leukemia	Tobacco (cigarettes per capita)	Female	0-6 days	95+ years	2	2
Acute lymphoid leukemia	Tobacco (cigarettes per capita)	Male	0-6 days	95+ years	2	2
Acute myeloid leukemia	Cumulative Cigarettes (10 Years)	Female	0-6 days	95+ years	2	2
Acute myeloid leukemia	Cumulative Cigarettes (10 Years)	Male	0-6 days	95+ years	2	2
Acute myeloid leukemia	Cumulative Cigarettes (20 Years)	Female	0-6 days	95+ years	2	2
Acute myeloid leukemia	Cumulative Cigarettes (20 Years)	Male	0-6 days	95+ years	2	2
Acute myeloid leukemia	Cumulative Cigarettes (5 Years)	Female	0-6 days	95+ years	2	NA
Acute myeloid leukemia	Cumulative Cigarettes (5 Years)	Male	0-6 days	95+ years	2	NA
Acute myeloid leukemia	Cumulative Cigarettes (15 Years)	Female	0-6 days	95+ years	2	NA
Acute myeloid leukemia	Cumulative Cigarettes (15 Years)	Male	0-6 days	95+ years	2	NA
Acute myeloid leukemia	Tobacco (cigarettes per capita)	Female	0-6 days	95+ years	2	NA
Acute myeloid leukemia	Tobacco (cigarettes per capita)	Male	0-6 days	95+ years	2	NA
Acute myeloid leukemia	Education (years per capita)	Female	0-6 days	95+ years	3	3
Acute myeloid leukemia	Education (years per capita)	Male	0-6 days	95+ years	3	3
Acute myeloid leukemia	Healthcare access and quality index	Female	0-6 days	95+ years	2	2
Acute myeloid leukemia	Healthcare access and quality index	Male	0-6 days	95+ years	2	2
Acute myeloid leukemia	LDI (I$ per capita)	Female	0-6 days	95+ years	3	3
Acute myeloid leukemia	LDI (I$ per capita)	Male	0-6 days	95+ years	3	3
Acute myeloid leukemia	Liters of alcohol consumed per capita	Female	0-6 days	95+ years	2	2
Acute myeloid leukemia	Liters of alcohol consumed per capita	Male	0-6 days	95+ years	2	2
Acute myeloid leukemia	Log-transformed SEV scalar: Leukemia	Female	0-6 days	95+ years	1	1
Acute myeloid leukemia	Log-transformed SEV scalar: Leukemia	Male	0-6 days	95+ years	1	1
Acute myeloid leukemia	Log-transformed age-standardized SEV scalar: Leukemia	Female	0-6 days	95+ years	1	1
Acute myeloid leukemia	Log-transformed age-standardized SEV scalar: Leukemia	Male	0-6 days	95+ years	1	1
Acute myeloid leukemia	Mean BMI	Female	0-6 days	95+ years	NA	2
Acute myeloid leukemia	Mean BMI	Male	0-6 days	95+ years	NA	2
Acute myeloid leukemia	Smoking Prevalence	Female	0-6 days	95+ years	2	2

Acute myeloid leukemia	Smoking Prevalence	Male	0-6 days	95+ years	2	2
Acute myeloid leukemia	Socio-demographic Index	Female	0-6 days	95+ years	3	3
Acute myeloid leukemia	Socio-demographic Index	Male	0-6 days	95+ years	3	3
Bladder cancer	Age- and sex-specific SEV for Low fruit	Female	15-19 years	95+ years	NA	3
Bladder cancer	Age- and sex-specific SEV for Low fruit	Male	15-19 years	95+ years	NA	3
Bladder cancer	Age- and sex-specific SEV for Low vegetables	Female	15-19 years	95+ years	NA	2
Bladder cancer	Age- and sex-specific SEV for Low vegetables	Male	15-19 years	95+ years	NA	2
Bladder cancer	Fruits adjusted (g)	Female	15-19 years	95+ years	2	NA
Bladder cancer	Fruits adjusted (g)	Male	15-19 years	95+ years	2	NA
Bladder cancer	Vegetables adjusted (g)	Female	15-19 years	95+ years	2	NA
Bladder cancer	Vegetables adjusted (g)	Male	15-19 years	95+ years	2	NA
Bladder cancer	Cumulative Cigarettes (10 Years)	Female	15-19 years	95+ years	1	2
Bladder cancer	Cumulative Cigarettes (10 Years)	Male	15-19 years	95+ years	1	2
Bladder cancer	Cumulative Cigarettes (5 Years)	Female	15-19 years	95+ years	1	NA
Bladder cancer	Cumulative Cigarettes (5 Years)	Male	15-19 years	95+ years	1	NA
Bladder cancer	Cumulative Cigarettes (15 Years)	Female	15-19 years	95+ years	1	NA
Bladder cancer	Cumulative Cigarettes (15 Years)	Male	15-19 years	95+ years	1	NA
Bladder cancer	Education (years per capita)	Female	15-19 years	95+ years	3	NA
Bladder cancer	Education (years per capita)	Male	15-19 years	95+ years	3	NA
Bladder cancer	Diabetes Fasting Plasma Glucose (mmol/L), age-standardized 25+	Female	15-19 years	95+ years	NA	2
Bladder cancer	Diabetes Fasting Plasma Glucose (mmol/L), age-standardized 25+	Male	15-19 years	95+ years	NA	2
Bladder cancer	Healthcare access and quality index	Female	15-19 years	95+ years	2	2
Bladder cancer	Healthcare access and quality index	Male	15-19 years	95+ years	2	2
Bladder cancer	LDI (I$ per capita)	Female	15-19 years	95+ years	3	3
Bladder cancer	LDI (I$ per capita)	Male	15-19 years	95+ years	3	3
Bladder cancer	Liters of alcohol consumed per capita	Female	15-19 years	95+ years	2	2
Bladder cancer	Liters of alcohol consumed per capita	Male	15-19 years	95+ years	2	2
Bladder cancer	Log-transformed SEV scalar: Bladder C	Female	15-19 years	95+ years	1	1

Bladder cancer	Log-transformed SEV scalar: Bladder C	Male	15-19 years	95+ years	1	1
Bladder cancer	Schistosomiasis Prevalence Results	Female	15-19 years	95+ years	NA	1
Bladder cancer	Schistosomiasis Prevalence Results	Male	15-19 years	95+ years	NA	1
Bladder cancer	Schistosomiasis Prevalence (proportion)	Female	15-19 years	95+ years	1	NA
Bladder cancer	Schistosomiasis Prevalence (proportion)	Male	15-19 years	95+ years	1	NA
Bladder cancer	Smoking Prevalence	Female	15-19 years	95+ years	1	1
Bladder cancer	Smoking Prevalence	Male	15-19 years	95+ years	1	1
Bladder cancer	Socio-demographic Index	Female	15-19 years	95+ years	3	3
Bladder cancer	Socio-demographic Index	Male	15-19 years	95+ years	3	3
Brain and nervous system cancer	Age- and sex- specific SEV for low fruit	Female	0-6 days	95+ years	NA	2
Brain and nervous system cancer	Age- and sex- specific SEV for low fruit	Male	0-6 days	95+ years	NA	2
Brain and nervous system cancer	Age- and sex-specific SEV for High red meat	Female	0-6 days	95+ years	NA	2
Brain and nervous system cancer	Age- and sex-specific SEV for High red meat	Male	0-6 days	95+ years	NA	2
Brain and nervous system cancer	Age- and sex-specific SEV for Low vegetables	Female	0-6 days	95+ years	NA	2
Brain and nervous system cancer	Age- and sex-specific SEV for Low vegetables	Male	0-6 days	95+ years	NA	2
Brain and nervous system cancer	Fruits adjusted (g)	Female	0-6 days	95+ years	2	NA
Brain and nervous system cancer	Fruits adjusted (g)	Male	0-6 days	95+ years	2	NA
Brain and nervous system cancer	Red meats adjusted (g)	Female	0-6 days	95+ years	2	NA
Brain and nervous system cancer	Red meats adjusted (g)	Male	0-6 days	95+ years	2	NA
Brain and nervous system cancer	Vegetables adjusted (g)	Female	0-6 days	95+ years	2	NA
Brain and nervous system cancer	Vegetables adjusted (g)	Male	0-6 days	95+ years	2	NA
Brain and nervous system cancer	Cholesterol (total, mean per capita)	Female	0-6 days	95+ years	2	2
Brain and nervous system cancer	Cholesterol (total, mean per capita)	Male	0-6 days	95+ years	2	2
Brain and nervous system cancer	Cumulative Cigarettes (10 Years)	Female	0-6 days	95+ years	1	1
Brain and nervous system cancer	Cumulative Cigarettes (10 Years)	Male	0-6 days	95+ years	1	1
Brain and nervous system cancer	Cumulative Cigarettes (15 Years)	Female	0-6 days	95+ years	1	NA
Brain and nervous system cancer	Cumulative Cigarettes (15 Years)	Male	0-6 days	95+ years	1	NA
Brain and nervous system cancer	Education (years per capita)	Female	0-6 days	95+ years	3	3
Brain and nervous system cancer	Education (years per capita)	Male	0-6 days	95+ years	3	3
Brain and nervous system cancer	Healthcare access and quality index	Female	0-6 days	95+ years	2	2

Brain and nervous system cancer	Healthcare access and quality index	Male	0-6 days	95+ years	2	2
Brain and nervous system cancer	LDI (I$ per capita)	Female	0-6 days	95+ years	3	3
Brain and nervous system cancer	LDI (I$ per capita)	Male	0-6 days	95+ years	3	3
Brain and nervous system cancer	Liters of alcohol consumed per capita	Female	0-6 days	95+ years	1	1
Brain and nervous system cancer	Liters of alcohol consumed per capita	Male	0-6 days	95+ years	1	1
Brain and nervous system cancer	Smoking Prevalence	Female	0-6 days	95+ years	1	1
Brain and nervous system cancer	Smoking Prevalence	Male	0-6 days	95+ years	1	1
Brain and nervous system cancer	Socio-demographic Index	Female	0-6 days	95+ years	3	3
Brain and nervous system cancer	Socio-demographic Index	Male	0-6 days	95+ years	3	3
Brain and nervous system cancer	Systolic Blood Pressure (mmHg)	Female	0-6 days	95+ years	2	2
Brain and nervous system cancer	Systolic Blood Pressure (mmHg)	Male	0-6 days	95+ years	2	2
Breast cancer	Age- and sex- specific SEV for low fruit	Female	15-19 years	95+ years	2	NA
Breast cancer	Age- and sex- specific SEV for low fruit	Male	15-19 years	95+ years	2	NA
Breast cancer	Age- and sex-specific SEV for Low vegetables	Female	15-19 years	95+ years	2	NA
Breast cancer	Age- and sex-specific SEV for Low vegetables	Male	15-19 years	95+ years	2	NA
Breast cancer	Fruits adjusted (g)	Female	15-19 years	95+ years	NA	2
Breast cancer	Fruits adjusted (g)	Male	15-19 years	95+ years	NA	2
Breast cancer	Vegetables adjusted (g)	Female	15-19 years	95+ years	NA	2
Breast cancer	Vegetables adjusted (g)	Male	15-19 years	95+ years	NA	2
Breast cancer	Age-specific fertility rate	Female	15-19 years	95+ years	2	2
Breast cancer	Cumulative Cigarettes (10 years)	Female	15-19 years	95+ years	2	2
Breast cancer	Cumulative Cigarettes (10 years)	Male	15-19 years	95+ years	2	2
Breast cancer	Cumulative Cigarettes (20 years)	Female	15-19 years	95+ years	2	2
Breast cancer	Cumulative Cigarettes (20 years)	Male	15-19 years	95+ years	2	2
Breast cancer	Cumulative Cigarettes (5 Years)	Female	15-19 years	95+ years	2	NA
Breast cancer	Cumulative Cigarettes (5 Years)	Male	15-19 years	95+ years	2	NA
Breast cancer	Cumulative Cigarettes (15 Years)	Female	15-19 years	95+ years	2	NA
Breast cancer	Cumulative Cigarettes (15 Years)	Male	15-19 years	95+ years	2	NA
Breast cancer	Education (years per capita)	Female	15-19 years	95+ years	3	NA
Breast cancer	Education (years per capita)	Male	15-19 years	95+ years	3	NA

Breast cancer	Diabetes Fasting Plasma Glucose (mmol/L), age-standardized 25+	Female	15-19 years	95+ years	NA	2
Breast cancer	Diabetes Fasting Plasma Glucose (mmol/L), age-standardized 25+	Male	15-19 years	95+ years	NA	2
Breast cancer	Healthcare access and quality index	Female	15-19 years	95+ years	2	2
Breast cancer	Healthcare access and quality index	Male	15-19 years	95+ years	2	2
Breast cancer	LDI (I$ per capita)	Female	15-19 years	95+ years	3	3
Breast cancer	LDI (I$ per capita)	Male	15-19 years	95+ years	3	NA
Breast cancer	Liters of alcohol consumed per capita	Female	15-19 years	95+ years	1	1
Breast cancer	Liters of alcohol consumed per capita	Male	15-19 years	95+ years	1	3
Breast cancer	Log-transformed SEV scalar: Breast C	Female	15-19 years	95+ years	1	1
Breast cancer	Log-transformed SEV scalar: Breast C	Male	15-19 years	95+ years	1	NA
Breast cancer	Mean BMI	Female	15-19 years	95+ years	1	1
Breast cancer	Mean BMI	Male	15-19 years	95+ years	1	3
Breast cancer	Smoking Prevalence	Female	15-19 years	95+ years	2	2
Breast cancer	Smoking Prevalence	Male	15-19 years	95+ years	2	NA
Breast cancer	Socio-demographic Index	Female	15-19 years	95+ years	3	3
Breast cancer	Socio-demographic Index	Male	15-19 years	95+ years	3	2
Breast cancer	Total Fertility Rate	Female	15-19 years	95+ years	2	2
Cervical cancer	Age- and sex- specific SEV for low fruit	Female	15-19 years	95+ years	NA	2
Cervical cancer	Age- and sex-specific SEV for Low vegetables	Female	15-19 years	95+ years	NA	2
Cervical cancer	Fruits adjusted (g)	Female	15-19 years	95+ years	2	NA
Cervical cancer	Vegetables adjusted (g)	Female	15-19 years	95+ years	2	NA
Cervical cancer	Age-specific fertility rate	Female	15-19 years	95+ years	2	2
Cervical cancer	Cumulative Cigarettes (5 Years)	Female	15-19 years	95+ years	1	1
Cervical cancer	Cumulative Cigarettes (10 Years)	Female	15-19 years	95+ years	1	NA
Cervical cancer	Cumulative Cigarettes (15 Years)	Female	15-19 years	95+ years	1	NA
Cervical cancer	Education (years per capita)	Female	15-19 years	95+ years	3	3
Cervical cancer	HIV age-standardized prevalence	Female	15-19 years	95+ years	1	1
Cervical cancer	Healthcare access and quality index	Female	15-19 years	95+ years	2	2

Cervical cancer	LDI (I$ per capita)	Female	15-19 years	95+ years	3	3
Cervical cancer	Smoking Prevalence	Female	15-19 years	95+ years	2	2
Cervical cancer	Socio-demographic Index	Female	15-19 years	95+ years	3	3
Cervical cancer	Total Fertility Rate	Female	15-19 years	95+ years	2	2
Chronic lymphoid leukemia	Cumulative Cigarettes (10 years)	Female	20-24 years	95+ years	2	2
Chronic lymphoid leukemia	Cumulative Cigarettes (10 years)	Male	20-24 years	95+ years	2	2
Chronic lymphoid leukemia	Cumulative Cigarettes (15 years)	Female	20-24 years	95+ years	2	2
Chronic lymphoid leukemia	Cumulative Cigarettes (15 years)	Male	20-24 years	95+ years	2	2
Chronic lymphoid leukemia	Cumulative Cigarettes (20 years)	Female	20-24 years	95+ years	2	2
Chronic lymphoid leukemia	Cumulative Cigarettes (20 years)	Male	20-24 years	95+ years	2	2
Chronic lymphoid leukemia	Cumulative Cigarettes (5 years)	Female	20-24 years	95+ years	2	2
Chronic lymphoid leukemia	Cumulative Cigarettes (5 years)	Male	20-24 years	95+ years	2	2
Chronic lymphoid leukemia	Education (years per capita)	Female	20-24 years	95+ years	3	3
Chronic lymphoid leukemia	Education (years per capita)	Male	20-24 years	95+ years	3	3
Chronic lymphoid leukemia	Healthcare access and quality index	Female	20-24 years	95+ years	1	1
Chronic lymphoid leukemia	Healthcare access and quality index	Male	20-24 years	95+ years	2	2
Chronic lymphoid leukemia	LDI (I$ per capita)	Female	20-24 years	95+ years	3	3
Chronic lymphoid leukemia	LDI (I$ per capita)	Male	20-24 years	95+ years	3	3
Chronic lymphoid leukemia	Liters of alcohol consumed per capita	Female	20-24 years	95+ years	2	2
Chronic lymphoid leukemia	Liters of alcohol consumed per capita	Male	20-24 years	95+ years	2	2
Chronic lymphoid leukemia	Log-transformed SEV scalar: Leukemia	Female	20-24 years	95+ years	1	1
Chronic lymphoid leukemia	Log-transformed SEV scalar: Leukemia	Male	20-24 years	95+ years	1	1
Chronic lymphoid leukemia	Log-transformed age-standardized SEV scalar: Leukemia	Female	20-24 years	95+ years	1	1
Chronic lymphoid leukemia	Log-transformed age-standardized SEV scalar: Leukemia	Male	20-24 years	95+ years	1	1
Chronic lymphoid leukemia	Mean BMI	Female	20-24 years	95+ years	NA	2
Chronic lymphoid leukemia	Mean BMI	Male	20-24 years	95+ years	NA	2
Chronic lymphoid leukemia	Smoking Prevalence	Female	20-24 years	95+ years	2	2
Chronic lymphoid leukemia	Smoking Prevalence	Male	20-24 years	95+ years	2	2

Chronic lymphoid leukemia	Socio-demographic Index	Female	20-24 years	95+ years	3	3
Chronic lymphoid leukemia	Socio-demographic Index	Male	20-24 years	95+ years	3	3
Chronic lymphoid leukemia	Tobacco (cigarettes per capita)	Female	20-24 years	95+ years	2	2
Chronic lymphoid leukemia	Tobacco (cigarettes per capita)	Male	20-24 years	95+ years	2	2
Chronic myeloid leukemia	Cumulative Cigarettes (10 years)	Female	0-6 days	95+ years	2	2
Chronic myeloid leukemia	Cumulative Cigarettes (10 years)	Male	0-6 days	95+ years	2	2
Chronic myeloid leukemia	Cumulative Cigarettes (15 years)	Female	0-6 days	95+ years	2	2
Chronic myeloid leukemia	Cumulative Cigarettes (15 years)	Male	0-6 days	95+ years	2	2
Chronic myeloid leukemia	Cumulative Cigarettes (20 years)	Female	0-6 days	95+ years	2	2
Chronic myeloid leukemia	Cumulative Cigarettes (20 years)	Male	0-6 days	95+ years	2	2
Chronic myeloid leukemia	Cumulative Cigarettes (5 years)	Female	0-6 days	95+ years	2	2
Chronic myeloid leukemia	Cumulative Cigarettes (5 years)	Male	0-6 days	95+ years	2	2
Chronic myeloid leukemia	Education (years per capita)	Female	0-6 days	95+ years	3	3
Chronic myeloid leukemia	Education (years per capita)	Male	0-6 days	95+ years	3	3
Chronic myeloid leukemia	Healthcare access and quality index	Female	0-6 days	95+ years	2	2
Chronic myeloid leukemia	Healthcare access and quality index	Male	0-6 days	95+ years	2	2
Chronic myeloid leukemia	LDI (I$ per capita)	Female	0-6 days	95+ years	3	3
Chronic myeloid leukemia	LDI (I$ per capita)	Male	0-6 days	95+ years	3	3
Chronic myeloid leukemia	Liters of alcohol consumed per capita	Female	0-6 days	95+ years	2	2
Chronic myeloid leukemia	Liters of alcohol consumed per capita	Male	0-6 days	95+ years	2	2
Chronic myeloid leukemia	Log-transformed SEV scalar: Leukemia	Female	0-6 days	95+ years	1	1
Chronic myeloid leukemia	Log-transformed SEV scalar: Leukemia	Male	0-6 days	95+ years	1	1
Chronic myeloid leukemia	Log-transformed age-standardized SEV scalar: Leukemia	Female	0-6 days	95+ years	1	1
Chronic myeloid leukemia	Log-transformed age-standardized SEV scalar: Leukemia	Male	0-6 days	95+ years	1	1
Chronic myeloid leukemia	Mean BMI	Female	0-6 days	95+ years	NA	2
Chronic myeloid leukemia	Mean BMI	Male	0-6 days	95+ years	NA	2
Chronic myeloid leukemia	Smoking Prevalence	Female	0-6 days	95+ years	2	2
Chronic myeloid leukemia	Smoking Prevalence	Male	0-6 days	95+ years	2	2

Chronic myeloid leukemia	Socio-demographic Index	Female	0-6 days	95+ years	3	3
Chronic myeloid leukemia	Socio-demographic Index	Male	0-6 days	95+ years	3	3
Chronic myeloid leukemia	Tobacco (cigarettes per capita)	Female	0-6 days	95+ years	2	2
Chronic myeloid leukemia	Tobacco (cigarettes per capita)	Male	0-6 days	95+ years	2	2
Colon and rectum cancer	Age- and sex- specific SEV for low fruit	Female	5-9 years	95+ years	NA	3
Colon and rectum cancer	Age- and sex- specific SEV for low fruit	Male	5-9 years	95+ years	NA	3
Colon and rectum cancer	Age- and sex-specific SEV for High red meat	Female	5-9 years	95+ years	NA	1
Colon and rectum cancer	Age- and sex-specific SEV for High red meat	Male	5-9 years	95+ years	NA	1
Colon and rectum cancer	Fruits adjusted (g)	Female	5-9 years	95+ years	2	NA
Colon and rectum cancer	Fruits adjusted (g)	Male	5-9 years	95+ years	2	NA
Colon and rectum cancer	Red meats adjusted (g)	Female	5-9 years	95+ years	1	NA
Colon and rectum cancer	Red meats adjusted (g)	Male	5-9 years	95+ years	1	NA
Colon and rectum cancer	Age- and sex-specific SEV for Low calcium	Female	5-9 years	95+ years	NA	2
Colon and rectum cancer	Age- and sex-specific SEV for Low calcium	Male	5-9 years	95+ years	NA	2
Colon and rectum cancer	Age- and sex-specific SEV for Low fiber	Female	5-9 years	95+ years	NA	2
Colon and rectum cancer	Age- and sex-specific SEV for Low fiber	Male	5-9 years	95+ years	NA	2
Colon and rectum cancer	Age- and sex-specific SEV for Low milk	Female	5-9 years	95+ years	NA	3
Colon and rectum cancer	Age- and sex-specific SEV for Low milk	Male	5-9 years	95+ years	NA	3
Colon and rectum cancer	Age- and sex-specific SEV for Low nuts and seeds	Female	5-9 years	95+ years	NA	3
Colon and rectum cancer	Age- and sex-specific SEV for Low nuts and seeds	Male	5-9 years	95+ years	NA	3
Colon and rectum cancer	Age- and sex-specific SEV for Low vegetables	Female	5-9 years	95+ years	NA	2
Colon and rectum cancer	Age- and sex-specific SEV for Low vegetables	Male	5-9 years	95+ years	NA	2
Colon and rectum cancer	Milk adjusted (g)	Female	5-9 years	95+ years	2	NA
Colon and rectum cancer	Milk adjusted (g)	Male	5-9 years	95+ years	2	NA
Colon and rectum cancer	Nuts seeds adjusted (g)	Female	5-9 years	95+ years	2	NA
Colon and rectum cancer	Nuts seeds adjusted (g)	Male	5-9 years	95+ years	2	NA
Colon and rectum cancer	Vegetables adjusted (g)	Female	5-9 years	95+ years	2	NA
Colon and rectum cancer	Vegetables adjusted (g)	Male	5-9 years	95+ years	2	NA

Colon and rectum cancer	Cumulative Cigarettes (5 Years)	Female	5-9 years	95+ years	2	2
Colon and rectum cancer	Cumulative Cigarettes (5 Years)	Male	5-9 years	95+ years	2	2
Colon and rectum cancer	Cumulative Cigarettes (10 Years)	Female	5-9 years	95+ years	2	NA
Colon and rectum cancer	Cumulative Cigarettes (10 Years)	Male	5-9 years	95+ years	2	NA
Colon and rectum cancer	Cumulative Cigarettes (15 Years)	Female	5-9 years	95+ years	2	NA
Colon and rectum cancer	Cumulative Cigarettes (15 years)	Male	5-9 years	95+ years	2	NA
Colon and rectum cancer	Cumulative Cigarettes (20 Years)	Female	5-9 years	95+ years	2	NA
Colon and rectum cancer	Cumulative Cigarettes (20 Years)	Male	5-9 years	95+ years	2	2
Colon and rectum cancer	Smoking Prevalence	Female	5-9 years	95+ years	1	NA
Colon and rectum cancer	Smoking Prevalence	Male	5-9 years	95+ years	1	NA
Colon and rectum cancer	Diabetes Fasting Plasma Glucose (mmol/L), age-standardized 25+	Female	5-9 years	95+ years	NA	2
Colon and rectum cancer	Diabetes Fasting Plasma Glucose (mmol/L), age-standardized 25+	Male	5-9 years	95+ years	NA	2
Colon and rectum cancer	Diabetes Age-Specific Prevalence (proportion)	Female	5-9 years	95+ years	2	NA
Colon and rectum cancer	Diabetes Age-Specific Prevalence (proportion)	Male	5-9 years	95+ years	2	NA
Colon and rectum cancer	Education (years per capita)	Female	5-9 years	95+ years	3	3
Colon and rectum cancer	Education (years per capita)	Male	5-9 years	95+ years	3	3
Colon and rectum cancer	Healthcare access and quality index	Female	5-9 years	95+ years	2	3
Colon and rectum cancer	Healthcare access and quality index	Male	5-9 years	95+ years	2	3
Colon and rectum cancer	LDI (I$ per capita)	Female	5-9 years	95+ years	3	3
Colon and rectum cancer	LDI (I$ per capita)	Male	5-9 years	95+ years	3	3
Colon and rectum cancer	Liters of alcohol consumed per capita	Female	5-9 years	95+ years	1	2
Colon and rectum cancer	Liters of alcohol consumed per capita	Male	5-9 years	95+ years	1	2
Colon and rectum cancer	Log-transformed SEV scalar: Colorect C	Female	5-9 years	95+ years	1	1
Colon and rectum cancer	Log-transformed SEV scalar: Colorect C	Male	5-9 years	95+ years	1	1
Colon and rectum cancer	Mean BMI	Female	5-9 years	95+ years	1	1
Colon and rectum cancer	Mean BMI	Male	5-9 years	95+ years	1	1
Colon and rectum cancer	Socio-demographic Index	Female	5-9 years	95+ years	3	3

Colon and rectum cancer	Socio-demographic Index	Male	5-9 years	95+ years	3	3
Colon and rectum cancer	Tobacco (cigarettes per capita)	Female	5-9 years	95+ years	1	1
Colon and rectum cancer	Tobacco (cigarettes per capita)	Male	5-9 years	95+ years	1	1
Colon and rectum cancer	Total Physical Activity (MET-min/week), Age-specific	Female	5-9 years	95+ years	NA	1
Colon and rectum cancer	Total Physical Activity (MET-min/week), Age-specific	Male	5-9 years	95+ years	NA	1
Colon and rectum cancer	PUFA adjusted(percent)	Female	5-9 years	95+ years	2	2
Colon and rectum cancer	PUFA adjusted(percent)	Male	5-9 years	95+ years	2	2
Esophageal cancer	Age- and sex- specific SEV for low fruit	Female	20-24 years	95+ years	NA	2
Esophageal cancer	Age- and sex- specific SEV for low fruit	Male	20-24 years	95+ years	NA	2
Esophageal cancer	Age- and sex-specific SEV for Low vegetables	Female	20-24 years	95+ years	NA	2
Esophageal cancer	Age- and sex-specific SEV for Low vegetables	Male	20-24 years	95+ years	NA	2
Esophageal cancer	Fruits adjusted (g)	Female	20-24 years	95+ years	1	NA
Esophageal cancer	Fruits adjusted (g)	Male	20-24 years	95+ years	1	NA
Esophageal cancer	Vegetables adjusted (g)	Female	20-24 years	95+ years	2	NA
Esophageal cancer	Vegetables adjusted (g)	Male	20-24 years	95+ years	2	NA
Esophageal cancer	Education (years per capita)	Female	20-24 years	95+ years	3	3
Esophageal cancer	Education (years per capita)	Male	20-24 years	95+ years	3	3
Esophageal cancer	Healthcare access and quality index	Female	20-24 years	95+ years	2	2
Esophageal cancer	Healthcare access and quality index	Male	20-24 years	95+ years	2	2
Esophageal cancer	Improved Water Source (proportion with access)	Female	20-24 years	95+ years	2	3
Esophageal cancer	Improved Water Source (proportion with access)	Male	20-24 years	95+ years	2	3
Esophageal cancer	Indoor Air Pollution (All Cooking Fuels)	Female	20-24 years	95+ years	2	2
Esophageal cancer	Indoor Air Pollution (All Cooking Fuels)	Male	20-24 years	95+ years	2	2
Esophageal cancer	LDI (I$ per capita)	Female	20-24 years	95+ years	3	3
Esophageal cancer	LDI (I$ per capita)	Male	20-24 years	95+ years	3	3
Esophageal cancer	Liters of alcohol consumed per capita	Female	20-24 years	95+ years	1	1
Esophageal cancer	Liters of alcohol consumed per capita	Male	20-24 years	95+ years	1	1

Esophageal cancer	Log-transformed SEV scalar: Esophag C	Female	20-24 years	95+ years	1	NA
Esophageal cancer	Log-transformed SEV scalar: Esophag C	Male	20-24 years	95+ years	1	NA
Esophageal cancer	Log-transformed age-standardized SEV scalar: Esophag C	Female	20-24 years	95+ years	1	1
Esophageal cancer	Log-transformed age-standardized SEV scalar: Esophag C	Male	20-24 years	95+ years	1	1
Esophageal cancer	Mean BMI	Female	20-24 years	95+ years	1	1
Esophageal cancer	Mean BMI	Male	20-24 years	95+ years	1	1
Esophageal cancer	Sanitation (proportion with access)	Female	20-24 years	95+ years	2	3
Esophageal cancer	Sanitation (proportion with access)	Male	20-24 years	95+ years	2	3
Esophageal cancer	Smoking Prevalence	Female	20-24 years	95+ years	1	1
Esophageal cancer	Smoking Prevalence	Male	20-24 years	95+ years	1	1
Esophageal cancer	Socio-demographic Index	Female	20-24 years	95+ years	3	3
Esophageal cancer	Socio-demographic Index	Male	20-24 years	95+ years	3	3
Esophageal cancer	Tobacco (cigarettes per capita)	Female	20-24 years	95+ years	1	2
Esophageal cancer	Tobacco (cigarettes per capita)	Male	20-24 years	95+ years	1	2
Gallbladder and biliary tract cancer	Age- and sex- specific SEV for low fruit	Female	20-24 years	95+ years	NA	2
Gallbladder and biliary tract cancer	Age- and sex- specific SEV for low fruit	Male	20-24 years	95+ years	NA	2
Gallbladder and biliary tract cancer	Age- and sex-specific SEV for Low vegetables	Female	20-24 years	95+ years	NA	2
Gallbladder and biliary tract cancer	Age- and sex-specific SEV for Low vegetables	Male	20-24 years	95+ years	NA	2
Gallbladder and biliary tract cancer	Fruits adjusted (g)	Female	20-24 years	95+ years	2	NA
Gallbladder and biliary tract cancer	Fruits adjusted (g)	Male	20-24 years	95+ years	2	NA
Gallbladder and biliary tract cancer	Vegetables adjusted (g)	Female	20-24 years	95+ years	2	NA
Gallbladder and biliary tract cancer	Vegetables adjusted (g)	Male	20-24 years	95+ years	2	NA
Gallbladder and biliary tract cancer	Cumulative Cigarettes (10 years)	Female	20-24 years	95+ years	2	2
Gallbladder and biliary tract cancer	Cumulative Cigarettes (10 years)	Male	20-24 years	95+ years	2	2
Gallbladder and biliary tract cancer	Cumulative Cigarettes (5 Years)	Female	20-24 years	95+ years	2	2
Gallbladder and biliary tract cancer	Cumulative Cigarettes (5 Years)	Male	20-24 years	95+ years	2	2
Gallbladder and biliary tract cancer	Diabetes Age-Standardized Prevalence (proportion)	Female	20-24 years	95+ years	2	2

Gallbladder and biliary tract cancer	Diabetes Age-Standardized Prevalence (proportion)	Male	20-24 years	95+ years	2	2
Gallbladder and biliary tract cancer	Education (years per capita)	Female	20-24 years	95+ years	3	3
Gallbladder and biliary tract cancer	Education (years per capita)	Male	20-24 years	95+ years	3	3
Gallbladder and biliary tract cancer	Healthcare access and quality index	Female	20-24 years	95+ years	2	2
Gallbladder and biliary tract cancer	Healthcare access and quality index	Male	20-24 years	95+ years	2	2
Gallbladder and biliary tract cancer	LDI (I$ per capita)	Female	20-24 years	95+ years	3	3
Gallbladder and biliary tract cancer	LDI (I$ per capita)	Male	20-24 years	95+ years	3	3
Gallbladder and biliary tract cancer	Liters of alcohol consumed per capita	Female	20-24 years	95+ years	2	2
Gallbladder and biliary tract cancer	Liters of alcohol consumed per capita	Male	20-24 years	95+ years	2	2
Gallbladder and biliary tract cancer	Log-transformed SEV scalar: Gallblad C	Female	20-24 years	95+ years	1	1
Gallbladder and biliary tract cancer	Log-transformed SEV scalar: Gallblad C	Male	20-24 years	95+ years	1	1
Gallbladder and biliary tract cancer	Mean BMI	Female	20-24 years	95+ years	1	1
Gallbladder and biliary tract cancer	Mean BMI	Male	20-24 years	95+ years	1	1
Gallbladder and biliary tract cancer	Smoking Prevalence	Female	20-24 years	95+ years	2	2
Gallbladder and biliary tract cancer	Smoking Prevalence	Male	20-24 years	95+ years	2	2
Gallbladder and biliary tract cancer	Socio-demographic Index	Female	20-24 years	95+ years	3	3
Gallbladder and biliary tract cancer	Socio-demographic Index	Male	20-24 years	95+ years	3	3
Gallbladder and biliary tract cancer	Tobacco (cigarettes per capita)	Female	20-24 years	95+ years	2	2
Gallbladder and biliary tract cancer	Tobacco (cigarettes per capita)	Male	20-24 years	95+ years	2	2
Hodgkin lymphoma	Education (years per capita)	Female	1-4 years	95+ years	3	3
Hodgkin lymphoma	Education (years per capita)	Male	1-4 years	95+ years	3	3
Hodgkin lymphoma	Healthcare access and quality index	Female	1-4 years	95+ years	2	2
Hodgkin lymphoma	Healthcare access and quality index	Male	1-4 years	95+ years	2	2
Hodgkin lymphoma	LDI (I$ per capita)	Female	1-4 years	95+ years	3	3
Hodgkin lymphoma	LDI (I$ per capita)	Male	1-4 years	95+ years	3	3
Hodgkin lymphoma	Socio-demographic Index	Female	1-4 years	95+ years	3	3
Hodgkin lymphoma	Socio-demographic Index	Male	1-4 years	95+ years	3	3
Kidney cancer	Cumulative Cigarettes (10 years)	Female	0-6 days	95+ years	2	1
Kidney cancer	Cumulative Cigarettes (10 years)	Male	0-6 days	95+ years	2	1

Kidney cancer	Cumulative Cigarettes (5 years)	Female	0-6 days	95+ years	1	NA
Kidney cancer	Cumulative Cigarettes (5 years)	Male	0-6 days	95+ years	1	NA
Kidney cancer	Cumulative Cigarettes (15 years)	Female	0-6 days	95+ years	1	NA
Kidney cancer	Cumulative Cigarettes (15 years)	Male	0-6 days	95+ years	1	NA
Kidney cancer	Smoking Prevalence	Female	0-6 days	95+ years	2	NA
Kidney cancer	Smoking Prevalence	Male	0-6 days	95+ years	2	NA
Kidney cancer	Diabetes Age-Standardized Prevalence (proportion)	Female	0-6 days	95+ years	2	2
Kidney cancer	Diabetes Age-Standardized Prevalence (proportion)	Male	0-6 days	95+ years	2	2
Kidney cancer	Education (years per capita)	Female	0-6 days	95+ years	3	3
Kidney cancer	Education (years per capita)	Male	0-6 days	95+ years	3	3
Kidney cancer	Healthcare access and quality index	Female	0-6 days	95+ years	2	2
Kidney cancer	Healthcare access and quality index	Male	0-6 days	95+ years	2	2
Kidney cancer	LDI (I$ per capita)	Female	0-6 days	95+ years	3	3
Kidney cancer	LDI (I$ per capita)	Male	0-6 days	95+ years	3	3
Kidney cancer	Liters of alcohol consumed per capita	Female	0-6 days	95+ years	2	2
Kidney cancer	Liters of alcohol consumed per capita	Male	0-6 days	95+ years	2	2
Kidney cancer	Log-transformed SEV scalar: Kidney C	Female	0-6 days	95+ years	1	1
Kidney cancer	Log-transformed SEV scalar: Kidney C	Male	0-6 days	95+ years	1	1
Kidney cancer	Mean BMI	Female	0-6 days	95+ years	1	1
Kidney cancer	Mean BMI	Male	0-6 days	95+ years	1	1
Kidney cancer	Socio-demographic Index	Female	0-6 days	95+ years	3	3
Kidney cancer	Socio-demographic Index	Male	0-6 days	95+ years	3	3
Kidney cancer	Systolic Blood Pressure (mmHg)	Female	0-6 days	95+ years	2	2
Kidney cancer	Systolic Blood Pressure (mmHg)	Male	0-6 days	95+ years	2	2
Kidney cancer	Tobacco (cigarettes per capita)	Female	0-6 days	95+ years	2	1
Kidney cancer	Tobacco (cigarettes per capita)	Male	0-6 days	95+ years	2	1
Larynx cancer	Age- and sex- specific SEV for low fruit	Female	20-24 years	95+ years	NA	3
Larynx cancer	Age- and sex- specific SEV for low fruit	Male	20-24 years	95+ years	NA	3

Larynx cancer	Age- and sex-specific SEV for Low vegetables	Female	20-24 years	95+ years	NA	2
Larynx cancer	Age- and sex-specific SEV for Low vegetables	Male	20-24 years	95+ years	NA	2
Larynx cancer	Fruits adjusted (g)	Female	20-24 years	95+ years	2	NA
Larynx cancer	Fruits adjusted (g)	Male	20-24 years	95+ years	2	NA
Larynx cancer	Vegetables adjusted (g)	Female	20-24 years	95+ years	2	NA
Larynx cancer	Vegetables adjusted (g)	Male	20-24 years	95+ years	2	NA
Larynx cancer	Asbestos consumption (metric tons per year per capita)	Female	20-24 years	95+ years	NA	2
Larynx cancer	Asbestos consumption (metric tons per year per capita)	Male	20-24 years	95+ years	NA	2
Larynx cancer	Cumulative Cigarettes (10 years)	Female	20-24 years	95+ years	2	2
Larynx cancer	Cumulative Cigarettes (10 years)	Male	20-24 years	95+ years	2	2
Larynx cancer	Cumulative Cigarettes (20 years)	Female	20-24 years	95+ years	2	2
Larynx cancer	Cumulative Cigarettes (20 years)	Male	20-24 years	95+ years	2	2
Larynx cancer	Cumulative Cigarettes (5 years)	Female	20-24 years	95+ years	2	NA
Larynx cancer	Cumulative Cigarettes (5 years)	Male	20-24 years	95+ years	2	NA
Larynx cancer	Cumulative Cigarettes (15 years)	Female	20-24 years	95+ years	2	NA
Larynx cancer	Cumulative Cigarettes (15 years)	Male	20-24 years	95+ years	2	NA
Larynx cancer	Education (years per capita)	Female	20-24 years	95+ years	3	NA
Larynx cancer	Education (years per capita)	Male	20-24 years	95+ years	3	NA
Larynx cancer	Population Density (under 150 ppl/sqkm, proportion)	Female	20-24 years	95+ years	2	NA
Larynx cancer	Population Density (under 150 ppl/sqkm, proportion)	Male	20-24 years	95+ years	2	NA
Larynx cancer	Healthcare access and quality index	Female	20-24 years	95+ years	2	2
Larynx cancer	Healthcare access and quality index	Male	20-24 years	95+ years	2	2
Larynx cancer	LDI (I$ per capita)	Female	20-24 years	95+ years	3	3
Larynx cancer	LDI (I$ per capita)	Male	20-24 years	95+ years	3	3
Larynx cancer	Liters of alcohol consumed per capita	Female	20-24 years	95+ years	1	1
Larynx cancer	Liters of alcohol consumed per capita	Male	20-24 years	95+ years	1	1
Larynx cancer	Log-transformed SEV scalar: Larynx C	Female	20-24 years	95+ years	1	1

Larynx cancer	Log-transformed SEV scalar: Larynx C	Male	20-24 years	95+ years	1	1
Larynx cancer	Population Density (over 1000 ppl/sqkm, proportion)	Female	20-24 years	95+ years	2	2
Larynx cancer	Population Density (over 1000 ppl/sqkm, proportion)	Male	20-24 years	95+ years	2	2
Larynx cancer	Smoking Prevalence	Female	20-24 years	95+ years	2	2
Larynx cancer	Smoking Prevalence	Male	20-24 years	95+ years	2	2
Larynx cancer	Socio-demographic Index	Female	20-24 years	95+ years	3	3
Larynx cancer	Socio-demographic Index	Male	20-24 years	95+ years	3	3
Leukemia	Cumulative Cigarettes (10 years)	Female	0-6 days	95+ years	2	2
Leukemia	Cumulative Cigarettes (10 years)	Male	0-6 days	95+ years	2	2
Leukemia	Cumulative Cigarettes (20 years)	Female	0-6 days	95+ years	2	2
Leukemia	Cumulative Cigarettes (20 years)	Male	0-6 days	95+ years	2	2
Leukemia	Cumulative Cigarettes (5 years)	Female	0-6 days	95+ years	2	NA
Leukemia	Cumulative Cigarettes (5 years)	Male	0-6 days	95+ years	2	NA
Leukemia	Cumulative Cigarettes (15 years)	Female	0-6 days	95+ years	2	NA
Leukemia	Cumulative Cigarettes (15 years)	Male	0-6 days	95+ years	2	NA
Leukemia	Smoking Prevalence	Female	0-6 days	95+ years	2	NA
Leukemia	Smoking Prevalence	Male	0-6 days	95+ years	2	NA
Leukemia	Education (years per capita)	Female	0-6 days	95+ years	3	3
Leukemia	Education (years per capita)	Male	0-6 days	95+ years	3	3
Leukemia	Healthcare access and quality index	Female	0-6 days	95+ years	2	2
Leukemia	Healthcare access and quality index	Male	0-6 days	95+ years	2	2
Leukemia	LDI (I$ per capita)	Female	0-6 days	95+ years	3	3
Leukemia	LDI (I$ per capita)	Male	0-6 days	95+ years	3	3
Leukemia	Liters of alcohol consumed per capita	Female	0-6 days	95+ years	2	2
Leukemia	Liters of alcohol consumed per capita	Male	0-6 days	95+ years	2	2
Leukemia	Log-transformed SEV scalar: Leukemia	Female	0-6 days	95+ years	1	1
Leukemia	Log-transformed SEV scalar: Leukemia	Male	0-6 days	95+ years	1	1
Leukemia	Log-transformed age-standardized SEV scalar: Leukemia	Female	0-6 days	95+ years	1	1

Leukemia	Log-transformed age-standardized SEV scalar: Leukemia	Male	0-6 days	95+ years	1	1
Leukemia	Mean BMI	Female	0-6 days	95+ years	NA	2
Leukemia	Mean BMI	Male	0-6 days	95+ years	NA	2
Leukemia	Socio-demographic Index	Female	0-6 days	95+ years	3	3
Leukemia	Socio-demographic Index	Male	0-6 days	95+ years	3	3
Leukemia	Tobacco (cigarettes per capita)	Female	0-6 days	95+ years	2	2
Leukemia	Tobacco (cigarettes per capita)	Male	0-6 days	95+ years	2	2
Lip and oral cavity cancer	Age- and sex- specific SEV for low fruit	Female	5-9 years	95+ years	NA	2
Lip and oral cavity cancer	Age- and sex- specific SEV for low fruit	Male	5-9 years	95+ years	NA	2
Lip and oral cavity cancer	Age- and sex-specific SEV for High red meat	Female	5-9 years	95+ years	NA	2
Lip and oral cavity cancer	Age- and sex-specific SEV for High red meat	Male	5-9 years	95+ years	NA	2
Lip and oral cavity cancer	Age- and sex-specific SEV for Low vegetables	Female	5-9 years	95+ years	NA	2
Lip and oral cavity cancer	Age- and sex-specific SEV for Low vegetables	Male	5-9 years	95+ years	NA	2
Lip and oral cavity cancer	Fruits adjusted (g)	Female	5-9 years	95+ years	2	NA
Lip and oral cavity cancer	Fruits adjusted (g)	Male	5-9 years	95+ years	2	NA
Lip and oral cavity cancer	Red meats adjusted (g)	Female	5-9 years	95+ years	2	NA
Lip and oral cavity cancer	Red meats adjusted (g)	Male	5-9 years	95+ years	2	NA
Lip and oral cavity cancer	Vegetables adjusted (g)	Female	5-9 years	95+ years	1	NA
Lip and oral cavity cancer	Vegetables adjusted (g)	Male	5-9 years	95+ years	1	NA
Lip and oral cavity cancer	Cumulative Cigarettes (10 years)	Female	5-9 years	95+ years	1	1
Lip and oral cavity cancer	Cumulative Cigarettes (10 years)	Male	5-9 years	95+ years	1	1
Lip and oral cavity cancer	Cumulative Cigarettes (20 years)	Female	5-9 years	95+ years	1	1
Lip and oral cavity cancer	Cumulative Cigarettes (20 years)	Male	5-9 years	95+ years	1	1
Lip and oral cavity cancer	Cumulative Cigarettes (5 years)	Female	5-9 years	95+ years	1	NA
Lip and oral cavity cancer	Cumulative Cigarettes (5 years)	Male	5-9 years	95+ years	1	NA
Lip and oral cavity cancer	Cumulative Cigarettes (15 years)	Female	5-9 years	95+ years	1	NA
Lip and oral cavity cancer	Cumulative Cigarettes (15 years)	Male	5-9 years	95+ years	1	NA
Lip and oral cavity cancer	Smoking Prevalence	Female	5-9 years	95+ years	1	NA
Lip and oral cavity cancer	Smoking Prevalence	Male	5-9 years	95+ years	1	NA

Lip and oral cavity cancer	Health System Access 2 (unitless)	Female	5-9 years	95+ years	2	NA
Lip and oral cavity cancer	Education (years per capita)	Female	5-9 years	95+ years	3	3
Lip and oral cavity cancer	Education (years per capita)	Male	5-9 years	95+ years	3	3
Lip and oral cavity cancer	Healthcare access and quality index	Female	5-9 years	95+ years	2	2
Lip and oral cavity cancer	Healthcare access and quality index	Male	5-9 years	95+ years	2	2
Lip and oral cavity cancer	LDI (I$ per capita)	Female	5-9 years	95+ years	3	3
Lip and oral cavity cancer	LDI (I$ per capita)	Male	5-9 years	95+ years	3	3
Lip and oral cavity cancer	Liters of alcohol consumed per capita	Female	5-9 years	95+ years	1	1
Lip and oral cavity cancer	Liters of alcohol consumed per capita	Male	5-9 years	95+ years	1	1
Lip and oral cavity cancer	Log-transformed SEV scalar: Lip oral C	Female	5-9 years	95+ years	1	1
Lip and oral cavity cancer	Log-transformed SEV scalar: Lip oral C	Male	5-9 years	95+ years	1	1
Lip and oral cavity cancer	Socio-demographic Index	Female	5-9 years	95+ years	3	3
Lip and oral cavity cancer	Socio-demographic Index	Male	5-9 years	95+ years	3	3
Lip and oral cavity cancer	Tobacco (cigarettes per capita)	Female	5-9 years	95+ years	1	1
Lip and oral cavity cancer	Tobacco (cigarettes per capita)	Male	5-9 years	95+ years	1	1
Liver cancer	Age- and sex-specific SEV for High red meat	Female	0-6 days	95+ years	NA	3
Liver cancer	Age- and sex-specific SEV for High red meat	Male	0-6 days	95+ years	NA	3
Liver cancer	Red meats adjusted (g)	Female	0-6 days	95+ years	2	NA
Liver cancer	Red meats adjusted (g)	Male	0-6 days	95+ years	2	NA
Liver cancer	Cumulative Cigarettes (20 years)	Female	0-6 days	95+ years	2	2
Liver cancer	Cumulative Cigarettes (20 years)	Male	0-6 days	95+ years	2	2
Liver cancer	Cumulative Cigarettes (10 years)	Female	0-6 days	95+ years	2	NA
Liver cancer	Cumulative Cigarettes (10 years)	Male	0-6 days	95+ years	2	NA
Liver cancer	Cumulative Cigarettes (5 years)	Female	0-6 days	95+ years	2	NA
Liver cancer	Cumulative Cigarettes (5 years)	Male	0-6 days	95+ years	2	NA
Liver cancer	Cumulative Cigarettes (15 years)	Female	0-6 days	95+ years	2	NA
Liver cancer	Cumulative Cigarettes (15 years)	Male	0-6 days	95+ years	2	NA
Liver cancer	Diabetes Fasting Plasma Glucose (mmol/L)	Female	0-6 days	95+ years	2	NA
Liver cancer	Diabetes Fasting Plasma Glucose (mmol/L)	Male	0-6 days	95+ years	2	NA

Liver cancer	Diabetes Age-Standardized Prevalence (proportion)	Female	0-6 days	95+ years	2	NA
Liver cancer	Diabetes Age-Standardized Prevalence (proportion)	Male	0-6 days	95+ years	2	NA
Liver cancer	Diabetes Fasting Plasma Glucose (mmol/L), age-standardized 25+	Female	0-6 days	95+ years	NA	2
Liver cancer	Diabetes Fasting Plasma Glucose (mmol/L), age-standardized 25+	Male	0-6 days	95+ years	NA	2
Liver cancer	Education (years per capita)	Female	0-6 days	95+ years	3	3
Liver cancer	Education (years per capita)	Male	0-6 days	95+ years	3	3
Liver cancer	HIV age-standardized prevalence	Female	0-6 days	95+ years	1	1
Liver cancer	HIV age-standardized prevalence	Male	0-6 days	95+ years	1	1
Liver cancer	Healthcare access and quality index	Female	0-6 days	95+ years	2	2
Liver cancer	Healthcare access and quality index	Male	0-6 days	95+ years	2	2
Liver cancer	Hepatitis B 3-dose coverage (proportion)	Female	0-6 days	95+ years	2	2
Liver cancer	Hepatitis B 3-dose coverage (proportion)	Male	0-6 days	95+ years	2	2
Liver cancer	Hepatitis B Seroprevalence (HBsAg) age standardized	Female	0-6 days	95+ years	NA	1
Liver cancer	Hepatitis B Seroprevalence (HBsAg) age standardized	Male	0-6 days	95+ years	NA	1
Liver cancer	Hepatitis B (HBsAg) Seroprevalence	Female	0-6 days	95+ years	1	NA
Liver cancer	Hepatitis B (HBsAg) Seroprevalence	Male	0-6 days	95+ years	1	NA
Liver cancer	Hepatitis B vaccine coverage (proportion), aged through time	Female	0-6 days	95+ years	NA	2
Liver cancer	Hepatitis B vaccine coverage (proportion), aged through time	Male	0-6 days	95+ years	NA	2
Liver cancer	Hepatitis B 3-dose coverage (proportion), lagged 5 years	Female	0-6 days	95+ years	2	NA
Liver cancer	Hepatitis B 3-dose coverage (proportion), lagged 5 years	Male	0-6 days	95+ years	2	NA
Liver cancer	Hepatitis B 3-dose coverage (proportion), lagged 10 years	Female	0-6 days	95+ years	2	NA

Liver cancer	Hepatitis B 3-dose coverage (proportion), lagged 10 years	Male	0-6 days	95+ years	2	NA
Liver cancer	Hepatitis C Seroprevalence (anti-HCV) age standardized	Female	0-6 days	95+ years	NA	1
Liver cancer	Hepatitis C Seroprevalence (anti-HCV) age standardized	Male	0-6 days	95+ years	NA	1
Liver cancer	Hepatitis C (IgG) Seroprevalence	Female	0-6 days	95+ years	1	NA
Liver cancer	Hepatitis C (IgG) Seroprevalence	Male	0-6 days	95+ years	1	NA
Liver cancer	Intravenous drug use (age-standardized proportion)	Female	0-6 days	95+ years	NA	2
Liver cancer	Intravenous drug use (age-standardized proportion)	Male	0-6 days	95+ years	NA	2
Liver cancer	LDI (I$ per capita)	Female	0-6 days	95+ years	3	3
Liver cancer	LDI (I$ per capita)	Male	0-6 days	95+ years	3	3
Liver cancer	Liters of alcohol consumed per capita	Female	0-6 days	95+ years	1	1
Liver cancer	Liters of alcohol consumed per capita	Male	0-6 days	95+ years	1	1
Liver cancer	Log-transformed SEV scalar: Liver C	Female	0-6 days	95+ years	1	1
Liver cancer	Log-transformed SEV scalar: Liver C	Male	0-6 days	95+ years	1	1
Liver cancer	Mean BMI	Female	0-6 days	95+ years	2	2
Liver cancer	Mean BMI	Male	0-6 days	95+ years	2	2
Liver cancer	Socio-demographic Index	Female	0-6 days	95+ years	3	3
Liver cancer	Socio-demographic Index	Male	0-6 days	95+ years	3	3
Liver cancer	Tobacco (cigarettes per capita)	Female	0-6 days	95+ years	2	2
Liver cancer	Tobacco (cigarettes per capita)	Male	0-6 days	95+ years	2	2
Malignant skin melanoma	Education (years per capita)	Female	0-6 days	95+ years	3	3
Malignant skin melanoma	Education (years per capita)	Male	0-6 days	95+ years	3	3
Malignant skin melanoma	Healthcare access and quality index	Female	0-6 days	95+ years	2	2
Malignant skin melanoma	Healthcare access and quality index	Male	0-6 days	95+ years	2	2
Malignant skin melanoma	LDI (I$ per capita)	Female	0-6 days	95+ years	3	3
Malignant skin melanoma	LDI (I$ per capita)	Male	0-6 days	95+ years	3	3
Malignant skin melanoma	Latitude 15 to 30 (proportion)	Female	0-6 days	95+ years	2	2

Malignant skin melanoma	Latitude 15 to 30 (proportion)	Male	0-6 days	95+ years	2	2
Malignant skin melanoma	Latitude 30 to 45 (proportion)	Female	0-6 days	95+ years	2	2
Malignant skin melanoma	Latitude 30 to 45 (proportion)	Male	0-6 days	95+ years	2	2
Malignant skin melanoma	Latitude Over 45 (proportion)	Female	0-6 days	95+ years	2	2
Malignant skin melanoma	Latitude Over 45 (proportion)	Male	0-6 days	95+ years	2	2
Malignant skin melanoma	Latitude Under 15 (proportion)	Female	0-6 days	95+ years	2	2
Malignant skin melanoma	Latitude Under 15 (proportion)	Male	0-6 days	95+ years	2	2
Malignant skin melanoma	Liters of alcohol consumed per capita	Female	0-6 days	95+ years	1	1
Malignant skin melanoma	Liters of alcohol consumed per capita	Male	0-6 days	95+ years	1	1
Malignant skin melanoma	Socio-demographic Index	Female	0-6 days	95+ years	3	3
Malignant skin melanoma	Socio-demographic Index	Male	0-6 days	95+ years	3	3
Malignant skin melanoma	Fruits adjusted (g)	Female	0-6 days	95+ years	2	NA
Malignant skin melanoma	Fruits adjusted (g)	Male	0-6 days	95+ years	2	NA
Malignant skin melanoma	Vegetables adjusted (g)	Female	0-6 days	95+ years	2	NA
Malignant skin melanoma	Vegetables adjusted (g)	Male	0-6 days	95+ years	2	NA
Mesothelioma	Asbestos consumption (metric tons per year per capita)	Female	20-24 years	95+ years	1	1
Mesothelioma	Asbestos consumption (metric tons per year per capita)	Male	20-24 years	95+ years	1	1
Mesothelioma	Asbestos production (binary)	Female	20-24 years	95+ years	1	NA
Mesothelioma	Asbestos production (binary)	Male	20-24 years	95+ years	1	NA
Mesothelioma	Asbestos production (kg) per capita	Female	20-24 years	95+ years	2	NA
Mesothelioma	Asbestos production (kg) per capita	Male	20-24 years	95+ years	2	NA
Mesothelioma	Cumulative Cigarettes (5 Years)	Female	20-24 years	95+ years	1	2
Mesothelioma	Cumulative Cigarettes (5 Years)	Male	20-24 years	95+ years	1	2
Mesothelioma	Education (years per capita)	Female	20-24 years	95+ years	3	3
Mesothelioma	Education (years per capita)	Male	20-24 years	95+ years	3	3
Mesothelioma	Gold production (binary)	Female	20-24 years	95+ years	2	2
Mesothelioma	Gold production (binary)	Male	20-24 years	95+ years	2	2
Mesothelioma	Gold production (kg) per capita	Female	20-24 years	95+ years	2	NA

Mesothelioma	Gold production (kg) per capita	Male	20-24 years	95+ years	2	NA
Mesothelioma	Healthcare access and quality index	Female	20-24 years	95+ years	2	2
Mesothelioma	Healthcare access and quality index	Male	20-24 years	95+ years	2	2
Mesothelioma	Indoor Air Pollution (All Cooking Fuels)	Female	20-24 years	95+ years	1	2
Mesothelioma	Indoor Air Pollution (All Cooking Fuels)	Male	20-24 years	95+ years	1	2
Mesothelioma	LDI (I$ per capita)	Female	20-24 years	95+ years	3	3
Mesothelioma	LDI (I$ per capita)	Male	20-24 years	95+ years	3	3
Mesothelioma	Log-transformed SEV scalar: Mesothel	Male	20-24 years	95+ years	NA	1
Mesothelioma	Log-transformed age-standardized SEV scalar: Mesothel	Male	20-24 years	95+ years	NA	1
Mesothelioma	Population Density (over 1000 ppl/sqkm, proportion)	Female	20-24 years	95+ years	2	2
Mesothelioma	Population Density (over 1000 ppl/sqkm, proportion)	Male	20-24 years	95+ years	2	2
Mesothelioma	Smoking Prevalence	Female	20-24 years	95+ years	1	1
Mesothelioma	Smoking Prevalence	Male	20-24 years	95+ years	1	1
Mesothelioma	Socio-demographic Index	Female	20-24 years	95+ years	3	3
Mesothelioma	Socio-demographic Index	Male	20-24 years	95+ years	3	3
Multiple myeloma	Age- and sex- specific SEV for low fruit	Female	20-24 years	95+ years	NA	2
Multiple myeloma	Age- and sex- specific SEV for low fruit	Male	20-24 years	95+ years	NA	2
Multiple myeloma	Age- and sex-specific SEV for High red meat	Female	20-24 years	95+ years	NA	2
Multiple myeloma	Age- and sex-specific SEV for High red meat	Male	20-24 years	95+ years	NA	2
Multiple myeloma	Age- and sex-specific SEV for Low vegetables	Female	20-24 years	95+ years	NA	2
Multiple myeloma	Age- and sex-specific SEV for Low vegetables	Male	20-24 years	95+ years	NA	2
Multiple myeloma	Fruits adjusted (g)	Female	20-24 years	95+ years	2	NA
Multiple myeloma	Fruits adjusted (g)	Male	20-24 years	95+ years	2	NA
Multiple myeloma	Red meats adjusted (g)	Female	20-24 years	95+ years	2	NA
Multiple myeloma	Red meats adjusted (g)	Male	20-24 years	95+ years	2	NA
Multiple myeloma	Vegetables adjusted (g)	Female	20-24 years	95+ years	2	NA
Multiple myeloma	Vegetables adjusted (g)	Male	20-24 years	95+ years	2	NA
Multiple myeloma	Education (years per capita)	Female	20-24 years	95+ years	3	3

Multiple myeloma	Education (years per capita)	Male	20-24 years	95+ years	3	3
Multiple myeloma	Healthcare access and quality index	Female	20-24 years	95+ years	2	2
Multiple myeloma	Healthcare access and quality index	Male	20-24 years	95+ years	2	2
Multiple myeloma	Improved Water Source (proportion with access)	Female	20-24 years	95+ years	2	2
Multiple myeloma	Improved Water Source (proportion with access)	Male	20-24 years	95+ years	2	2
Multiple myeloma	LDI (I$ per capita)	Female	20-24 years	95+ years	3	3
Multiple myeloma	LDI (I$ per capita)	Male	20-24 years	95+ years	3	3
Multiple myeloma	Liters of alcohol consumed per capita	Female	20-24 years	95+ years	1	1
Multiple myeloma	Liters of alcohol consumed per capita	Male	20-24 years	95+ years	1	1
Multiple myeloma	Mean BMI	Female	20-24 years	95+ years	2	2
Multiple myeloma	Mean BMI	Male	20-24 years	95+ years	2	2
Multiple myeloma	Sanitation (proportion with access)	Female	20-24 years	95+ years	2	2
Multiple myeloma	Sanitation (proportion with access)	Male	20-24 years	95+ years	2	2
Multiple myeloma	Smoking Prevalence	Female	20-24 years	95+ years	1	1
Multiple myeloma	Smoking Prevalence	Male	20-24 years	95+ years	1	1
Multiple myeloma	Socio-demographic Index	Female	20-24 years	95+ years	3	3
Multiple myeloma	Socio-demographic Index	Male	20-24 years	95+ years	3	3
Multiple myeloma	Tobacco (cigarettes per capita)	Female	20-24 years	95+ years	1	1
Multiple myeloma	Tobacco (cigarettes per capita)	Male	20-24 years	95+ years	1	1
Nasopharynx cancer	Age- and sex- specific SEV for low fruit	Female	5-9 years	95+ years	NA	3
Nasopharynx cancer	Age- and sex- specific SEV for low fruit	Male	5-9 years	95+ years	NA	3
Nasopharynx cancer	Age- and sex-specific SEV for Low vegetables	Female	5-9 years	95+ years	NA	2
Nasopharynx cancer	Age- and sex-specific SEV for Low vegetables	Male	5-9 years	95+ years	NA	2
Nasopharynx cancer	Fruits adjusted (g)	Female	5-9 years	95+ years	2	NA
Nasopharynx cancer	Fruits adjusted (g)	Male	5-9 years	95+ years	2	NA
Nasopharynx cancer	Vegetables adjusted (g)	Female	5-9 years	95+ years	2	NA
Nasopharynx cancer	Vegetables adjusted (g)	Male	5-9 years	95+ years	2	NA
Nasopharynx cancer	Cumulative Cigarettes (10 years)	Female	5-9 years	95+ years	1	1

Nasopharynx cancer	Cumulative Cigarettes (10 years)	Male	5-9 years	95+ years	1	1
Nasopharynx cancer	Cumulative Cigarettes (20 years)	Female	5-9 years	95+ years	1	1
Nasopharynx cancer	Cumulative Cigarettes (20 years)	Male	5-9 years	95+ years	1	1
Nasopharynx cancer	Cumulative Cigarettes (5 years)	Female	5-9 years	95+ years	1	NA
Nasopharynx cancer	Cumulative Cigarettes (5 years)	Male	5-9 years	95+ years	1	NA
Nasopharynx cancer	Cumulative Cigarettes (15 years)	Female	5-9 years	95+ years	1	NA
Nasopharynx cancer	Cumulative Cigarettes (15 years)	Male	5-9 years	95+ years	1	NA
Nasopharynx cancer	Education (years per capita)	Female	5-9 years	95+ years	3	3
Nasopharynx cancer	Education (years per capita)	Male	5-9 years	95+ years	3	3
Nasopharynx cancer	Healthcare access and quality index	Female	5-9 years	95+ years	2	2
Nasopharynx cancer	Healthcare access and quality index	Male	5-9 years	95+ years	2	2
Nasopharynx cancer	LDI (I$ per capita)	Female	5-9 years	95+ years	3	3
Nasopharynx cancer	LDI (I$ per capita)	Male	5-9 years	95+ years	3	3
Nasopharynx cancer	Liters of alcohol consumed per capita	Female	5-9 years	95+ years	1	1
Nasopharynx cancer	Liters of alcohol consumed per capita	Male	5-9 years	95+ years	1	1
Nasopharynx cancer	Log-transformed SEV scalar: Nasoph C	Female	5-9 years	95+ years	1	1
Nasopharynx cancer	Log-transformed SEV scalar: Nasoph C	Male	5-9 years	95+ years	1	1
Nasopharynx cancer	Smoking Prevalence	Female	5-9 years	95+ years	1	NA
Nasopharynx cancer	Smoking Prevalence	Male	5-9 years	95+ years	1	NA
Nasopharynx cancer	Population Density (under 150 ppl/sqkm, proportion)	Female	5-9 years	95+ years	2	NA
Nasopharynx cancer	Population Density (under 150 ppl/sqkm, proportion)	Male	5-9 years	95+ years	2	NA
Nasopharynx cancer	Population Density (over 1000 ppl/sqkm, proportion)	Female	5-9 years	95+ years	2	2
Nasopharynx cancer	Population Density (over 1000 ppl/sqkm, proportion)	Male	5-9 years	95+ years	2	2
Nasopharynx cancer	Socio-demographic Index	Female	5-9 years	95+ years	3	3
Nasopharynx cancer	Socio-demographic Index	Male	5-9 years	95+ years	3	3
Nasopharynx cancer	Tobacco (cigarettes per capita)	Female	5-9 years	95+ years	1	1
Nasopharynx cancer	Tobacco (cigarettes per capita)	Male	5-9 years	95+ years	1	1

Non-Hodgkin lymphoma	Cumulative Cigarettes (10 years)	Female	1-4 years	95+ years	2	2
Non-Hodgkin lymphoma	Cumulative Cigarettes (10 years)	Male	1-4 years	95+ years	2	2
Non-Hodgkin lymphoma	Cumulative Cigarettes (15 years)	Female	1-4 years	95+ years	2	2
Non-Hodgkin lymphoma	Cumulative Cigarettes (15 years)	Male	1-4 years	95+ years	2	2
Non-Hodgkin lymphoma	Cumulative Cigarettes (20 years)	Female	1-4 years	95+ years	2	2
Non-Hodgkin lymphoma	Cumulative Cigarettes (20 years)	Male	1-4 years	95+ years	2	2
Non-Hodgkin lymphoma	Cumulative Cigarettes (5 Years)	Female	1-4 years	95+ years	2	2
Non-Hodgkin lymphoma	Cumulative Cigarettes (5 Years)	Male	1-4 years	95+ years	2	2
Non-Hodgkin lymphoma	Healthcare access and quality index	Female	1-4 years	95+ years	2	2
Non-Hodgkin lymphoma	Healthcare access and quality index	Male	1-4 years	95+ years	2	2
Non-Hodgkin lymphoma	LDI (I$ per capita)	Female	1-4 years	95+ years	3	3
Non-Hodgkin lymphoma	LDI (I$ per capita)	Male	1-4 years	95+ years	3	3
Non-Hodgkin lymphoma	Liters of alcohol consumed per capita	Female	1-4 years	95+ years	2	2
Non-Hodgkin lymphoma	Liters of alcohol consumed per capita	Male	1-4 years	95+ years	2	2
Non-Hodgkin lymphoma	Mean BMI	Female	1-4 years	95+ years	NA	2
Non-Hodgkin lymphoma	Mean BMI	Male	1-4 years	95+ years	NA	2
Non-Hodgkin lymphoma	Smoking Prevalence	Female	1-4 years	95+ years	2	2
Non-Hodgkin lymphoma	Smoking Prevalence	Male	1-4 years	95+ years	2	2
Non-Hodgkin lymphoma	Socio-demographic Index	Female	1-4 years	95+ years	3	3
Non-Hodgkin lymphoma	Socio-demographic Index	Male	1-4 years	95+ years	3	3
Non-Hodgkin lymphoma	Total Fertility Rate	Female	1-4 years	95+ years	3	3
Non-melanoma skin cancer	Average latitude	Female	20-24 years	95+ years	2	2
Non-melanoma skin cancer	Average latitude	Male	20-24 years	95+ years	2	2
Non-melanoma skin cancer	Cumulative Cigarettes (10 years)	Female	20-24 years	95+ years	1	1
Non-melanoma skin cancer	Cumulative Cigarettes (10 years)	Male	20-24 years	95+ years	1	1
Non-melanoma skin cancer	Cumulative Cigarettes (15 years)	Female	20-24 years	95+ years	1	1
Non-melanoma skin cancer	Cumulative Cigarettes (15 years)	Male	20-24 years	95+ years	1	1
Non-melanoma skin cancer	Cumulative Cigarettes (5 years)	Female	20-24 years	95+ years	1	1
Non-melanoma skin cancer	Cumulative Cigarettes (5 years)	Male	20-24 years	95+ years	1	1
Non-melanoma skin cancer	Education (years per capita)	Female	20-24 years	95+ years	3	3

Non-melanoma skin cancer	Education (years per capita)	Male	20-24 years	95+ years	3	3
Non-melanoma skin cancer	Healthcare access and quality index	Female	20-24 years	95+ years	2	2
Non-melanoma skin cancer	Healthcare access and quality index	Male	20-24 years	95+ years	2	2
Non-melanoma skin cancer	LDI (I$ per capita)	Female	20-24 years	95+ years	3	3
Non-melanoma skin cancer	LDI (I$ per capita)	Male	20-24 years	95+ years	3	3
Non-melanoma skin cancer	Smoking Prevalence	Female	20-24 years	95+ years	1	1
Non-melanoma skin cancer	Smoking Prevalence	Male	20-24 years	95+ years	1	1
Non-melanoma skin cancer	Socio-demographic Index	Female	20-24 years	95+ years	3	3
Non-melanoma skin cancer	Socio-demographic Index	Male	20-24 years	95+ years	3	3
Non-melanoma skin cancer (squamous-cell carcinoma)	Average latitude	Female	20-24 years	95+ years	2	2
Non-melanoma skin cancer (squamous-cell carcinoma)	Average latitude	Male	20-24 years	95+ years	2	2
Non-melanoma skin cancer (squamous-cell carcinoma)	Cumulative Cigarettes (10 years)	Female	20-24 years	95+ years	1	1
Non-melanoma skin cancer (squamous-cell carcinoma)	Cumulative Cigarettes (10 years)	Male	20-24 years	95+ years	1	1
Non-melanoma skin cancer (squamous-cell carcinoma)	Cumulative Cigarettes (15 years)	Female	20-24 years	95+ years	1	1
Non-melanoma skin cancer (squamous-cell carcinoma)	Cumulative Cigarettes (15 years)	Male	20-24 years	95+ years	1	1
Non-melanoma skin cancer (squamous-cell carcinoma)	Cumulative Cigarettes (5 years)	Female	20-24 years	95+ years	1	1
Non-melanoma skin cancer (squamous-cell carcinoma)	Cumulative Cigarettes (5 years)	Male	20-24 years	95+ years	1	1
Non-melanoma skin cancer (squamous-cell carcinoma)	Education (years per capita)	Female	20-24 years	95+ years	3	3
Non-melanoma skin cancer (squamous-cell carcinoma)	Education (years per capita)	Male	20-24 years	95+ years	3	3
Non-melanoma skin cancer (squamous-cell carcinoma)	Healthcare access and quality index	Female	20-24 years	95+ years	2	2

Non-melanoma skin cancer (squamous-cell carcinoma)	Healthcare access and quality index	Male	20-24 years	95+ years	2	2
Non-melanoma skin cancer (squamous-cell carcinoma)	LDI (I$ per capita)	Female	20-24 years	95+ years	3	3
Non-melanoma skin cancer (squamous-cell carcinoma)	LDI (I$ per capita)	Male	20-24 years	95+ years	3	3
Non-melanoma skin cancer (squamous-cell carcinoma)	Smoking Prevalence	Female	20-24 years	95+ years	1	1
Non-melanoma skin cancer (squamous-cell carcinoma)	Smoking Prevalence	Male	20-24 years	95+ years	1	1
Non-melanoma skin cancer (squamous-cell carcinoma)	Socio-demographic Index	Female	20-24 years	95+ years	3	3
Non-melanoma skin cancer (squamous-cell carcinoma)	Socio-demographic Index	Male	20-24 years	95+ years	3	3
Other leukemia	Cumulative Cigarettes (10 years)	Female	0-6 days	95+ years	2	2
Other leukemia	Cumulative Cigarettes (10 years)	Male	0-6 days	95+ years	2	2
Other leukemia	Cumulative Cigarettes (20 years)	Female	0-6 days	95+ years	2	2
Other leukemia	Cumulative Cigarettes (20 years)	Male	0-6 days	95+ years	2	2
Other leukemia	Cumulative Cigarettes (15 years)	Female	0-6 days	95+ years	2	NA
Other leukemia	Cumulative Cigarettes (15 years)	Male	0-6 days	95+ years	2	NA
Other leukemia	Cumulative Cigarettes (5 years)	Female	0-6 days	95+ years	2	NA
Other leukemia	Cumulative Cigarettes (5 years)	Male	0-6 days	95+ years	2	NA
Other leukemia	Smoking Prevalence	Female	0-6 days	95+ years	2	NA
Other leukemia	Smoking Prevalence	Male	0-6 days	95+ years	2	NA
Other leukemia	Education (years per capita)	Female	0-6 days	95+ years	3	3
Other leukemia	Education (years per capita)	Male	0-6 days	95+ years	3	3
Other leukemia	Healthcare access and quality index	Female	0-6 days	95+ years	2	2
Other leukemia	Healthcare access and quality index	Male	0-6 days	95+ years	2	2
Other leukemia	LDI (I$ per capita)	Female	0-6 days	95+ years	3	3
Other leukemia	LDI (I$ per capita)	Male	0-6 days	95+ years	3	3
Other leukemia	Liters of alcohol consumed per capita	Female	0-6 days	95+ years	2	2

Other leukemia	Liters of alcohol consumed per capita	Male	0-6 days	95+ years	2	2
Other leukemia	Log-transformed SEV scalar: Leukemia	Female	0-6 days	95+ years	1	1
Other leukemia	Log-transformed SEV scalar: Leukemia	Male	0-6 days	95+ years	1	1
Other leukemia	Log-transformed age-standardized SEV scalar: Leukemia	Female	0-6 days	95+ years	1	1
Other leukemia	Log-transformed age-standardized SEV scalar: Leukemia	Male	0-6 days	95+ years	1	1
Other leukemia	Mean BMI	Female	0-6 days	95+ years	NA	2
Other leukemia	Mean BMI	Male	0-6 days	95+ years	NA	2
Other leukemia	Socio-demographic Index	Female	0-6 days	95+ years	3	3
Other leukemia	Socio-demographic Index	Male	0-6 days	95+ years	3	3
Other leukemia	Tobacco (cigarettes per capita)	Female	0-6 days	95+ years	2	2
Other leukemia	Tobacco (cigarettes per capita)	Male	0-6 days	95+ years	2	2
Other malignant neoplasms	Age- and sex- specific SEV for low fruit	Female	0-6 days	95+ years	NA	2
Other malignant neoplasms	Age- and sex- specific SEV for low fruit	Male	0-6 days	95+ years	NA	2
Other malignant neoplasms	Age- and sex-specific SEV for Low nuts and seeds	Female	0-6 days	95+ years	NA	2
Other malignant neoplasms	Age- and sex-specific SEV for Low nuts and seeds	Male	0-6 days	95+ years	NA	2
Other malignant neoplasms	Age- and sex-specific SEV for Low vegetables	Female	0-6 days	95+ years	NA	2
Other malignant neoplasms	Age- and sex-specific SEV for Low vegetables	Male	0-6 days	95+ years	NA	2
Other malignant neoplasms	Fruits adjusted (g)	Female	0-6 days	95+ years	2	NA
Other malignant neoplasms	Fruits adjusted (g)	Male	0-6 days	95+ years	2	NA
Other malignant neoplasms	Nuts seeds adjusted (g)	Female	0-6 days	95+ years	2	NA
Other malignant neoplasms	Nuts seeds adjusted (g)	Male	0-6 days	95+ years	2	NA
Other malignant neoplasms	Vegetables adjusted (g)	Female	0-6 days	95+ years	2	NA
Other malignant neoplasms	Vegetables adjusted (g)	Male	0-6 days	95+ years	2	NA
Other malignant neoplasms	Education (years per capita)	Female	0-6 days	95+ years	3	3
Other malignant neoplasms	Education (years per capita)	Male	0-6 days	95+ years	3	3
Other malignant neoplasms	Healthcare access and quality index	Female	0-6 days	95+ years	2	2
Other malignant neoplasms	Healthcare access and quality index	Male	0-6 days	95+ years	2	2

Other malignant neoplasms	LDI (I$ per capita)	Female	0-6 days	95+ years	3	3
Other malignant neoplasms	LDI (I$ per capita)	Male	0-6 days	95+ years	3	3
Other malignant neoplasms	Smoking Prevalence	Female	0-6 days	95+ years	1	1
Other malignant neoplasms	Smoking Prevalence	Male	0-6 days	95+ years	1	1
Other malignant neoplasms	Socio-demographic Index	Female	0-6 days	95+ years	3	3
Other malignant neoplasms	Socio-demographic Index	Male	0-6 days	95+ years	3	3
Other malignant neoplasms	Tobacco (cigarettes per capita)	Female	0-6 days	95+ years	1	1
Other malignant neoplasms	Tobacco (cigarettes per capita)	Male	0-6 days	95+ years	1	1
Other malignant neoplasms	PUFA adjusted(percent)	Female	0-6 days	95+ years	2	2
Other malignant neoplasms	PUFA adjusted(percent)	Male	0-6 days	95+ years	2	2
Other pharynx cancer	Age- and sex- specific SEV for low fruit	Female	20-24 years	95+ years	NA	2
Other pharynx cancer	Age- and sex- specific SEV for low fruit	Male	20-24 years	95+ years	NA	2
Other pharynx cancer	Age- and sex-specific SEV for Low vegetables	Female	20-24 years	95+ years	NA	2
Other pharynx cancer	Age- and sex-specific SEV for Low vegetables	Male	20-24 years	95+ years	NA	2
Other pharynx cancer	Fruits adjusted (g)	Female	20-24 years	95+ years	2	NA
Other pharynx cancer	Fruits adjusted (g)	Male	20-24 years	95+ years	2	NA
Other pharynx cancer	Vegetables adjusted (g)	Female	20-24 years	95+ years	2	NA
Other pharynx cancer	Vegetables adjusted (g)	Male	20-24 years	95+ years	2	NA
Other pharynx cancer	Cumulative Cigarettes (5 years)	Female	20-24 years	95+ years	2	2
Other pharynx cancer	Cumulative Cigarettes (5 years)	Male	20-24 years	95+ years	2	2
Other pharynx cancer	Education (years per capita)	Female	20-24 years	95+ years	3	3
Other pharynx cancer	Education (years per capita)	Male	20-24 years	95+ years	3	3
Other pharynx cancer	Healthcare access and quality index	Female	20-24 years	95+ years	2	2
Other pharynx cancer	Healthcare access and quality index	Male	20-24 years	95+ years	2	2
Other pharynx cancer	LDI (I$ per capita)	Female	20-24 years	95+ years	3	3
Other pharynx cancer	LDI (I$ per capita)	Male	20-24 years	95+ years	3	3
Other pharynx cancer	Liters of alcohol consumed per capita	Female	20-24 years	95+ years	1	1
Other pharynx cancer	Liters of alcohol consumed per capita	Male	20-24 years	95+ years	1	1
Other pharynx cancer	Log-transformed SEV scalar: Oth Phar C	Female	20-24 years	95+ years	1	1
Other pharynx cancer	Log-transformed SEV scalar: Oth Phar C	Male	20-24 years	95+ years	1	1

Other pharynx cancer	Population Density (over 1000 ppl/sqkm, proportion)	Female	20-24 years	95+ years	2	2
Other pharynx cancer	Population Density (over 1000 ppl/sqkm, proportion)	Male	20-24 years	95+ years	2	2
Other pharynx cancer	Population Density (under 150 ppl/sqkm, proportion)	Female	20-24 years	95+ years	2	2
Other pharynx cancer	Population Density (under 150 ppl/sqkm, proportion)	Male	20-24 years	95+ years	2	2
Other pharynx cancer	Smoking Prevalence	Female	20-24 years	95+ years	1	1
Other pharynx cancer	Smoking Prevalence	Male	20-24 years	95+ years	1	1
Other pharynx cancer	Socio-demographic Index	Female	20-24 years	95+ years	3	3
Other pharynx cancer	Socio-demographic Index	Male	20-24 years	95+ years	3	3
Ovarian cancer	Age- and sex- specific SEV for low fruit	Female	5-9 years	95+ years	NA	3
Ovarian cancer	Age- and sex-specific SEV for Low vegetables	Female	5-9 years	95+ years	NA	3
Ovarian cancer	Fruits adjusted (g)	Female	5-9 years	95+ years	2	NA
Ovarian cancer	Vegetables adjusted (g)	Female	5-9 years	95+ years	2	NA
Ovarian cancer	Asbestos consumption (metric tons per year per capita)	Female	5-9 years	95+ years	NA	2
Ovarian cancer	Contraception (Modern) Prevalence (proportion)	Female	5-9 years	95+ years	1	2
Ovarian cancer	Cumulative Cigarettes (10 years)	Female	5-9 years	95+ years	1	2
Ovarian cancer	Cumulative Cigarettes (20 years)	Female	5-9 years	95+ years	1	2
Ovarian cancer	Cumulative Cigarettes (5 years)	Female	5-9 years	95+ years	1	NA
Ovarian cancer	Cumulative Cigarettes (15 years)	Female	5-9 years	95+ years	1	NA
Ovarian cancer	Tobacco (cigarettes per capita)	Female	5-9 years	95+ years	1	NA
Ovarian cancer	Diabetes Age-Standardized Prevalence (proportion)	Female	5-9 years	95+ years	2	2
Ovarian cancer	Education (years per capita)	Female	5-9 years	95+ years	3	3
Ovarian cancer	Healthcare access and quality index	Female	5-9 years	95+ years	2	2
Ovarian cancer	LDI (I$ per capita)	Female	5-9 years	95+ years	3	3
Ovarian cancer	Liters of alcohol consumed per capita	Female	5-9 years	95+ years	1	1

Ovarian cancer	Log-transformed SEV scalar: Ovary C	Female	5-9 years	95+ years	1	1
Ovarian cancer	Mean BMI	Female	5-9 years	95+ years	2	2
Ovarian cancer	Smoking Prevalence	Female	5-9 years	95+ years	2	2
Ovarian cancer	Socio-demographic Index	Female	5-9 years	95+ years	3	3
Ovarian cancer	Total Fertility Rate	Female	5-9 years	95+ years	2	2
Ovarian cancer	energy unadjusted(kcal)	Female	5-9 years	95+ years	2	2
Pancreatic cancer	Age- and sex- specific SEV for low fruit	Female	15-19 years	95+ years	NA	3
Pancreatic cancer	Age- and sex- specific SEV for low fruit	Male	15-19 years	95+ years	NA	3
Pancreatic cancer	Age- and sex-specific SEV for High red meat	Female	15-19 years	95+ years	NA	2
Pancreatic cancer	Age- and sex-specific SEV for High red meat	Male	15-19 years	95+ years	NA	2
Pancreatic cancer	Age- and sex-specific SEV for Low vegetables	Female	15-19 years	95+ years	NA	3
Pancreatic cancer	Age- and sex-specific SEV for Low vegetables	Male	15-19 years	95+ years	NA	3
Pancreatic cancer	Fruits adjusted (g)	Female	15-19 years	95+ years	2	NA
Pancreatic cancer	Fruits adjusted (g)	Male	15-19 years	95+ years	2	NA
Pancreatic cancer	Red meats adjusted (g)	Female	15-19 years	95+ years	2	NA
Pancreatic cancer	Red meats adjusted (g)	Male	15-19 years	95+ years	2	NA
Pancreatic cancer	Vegetables adjusted (g)	Female	15-19 years	95+ years	2	NA
Pancreatic cancer	Vegetables adjusted (g)	Male	15-19 years	95+ years	2	NA
Pancreatic cancer	Cumulative Cigarettes (10 years)	Female	15-19 years	95+ years	1	1
Pancreatic cancer	Cumulative Cigarettes (10 years)	Male	15-19 years	95+ years	1	1
Pancreatic cancer	Cumulative Cigarettes (20 years)	Female	15-19 years	95+ years	1	1
Pancreatic cancer	Cumulative Cigarettes (20 years)	Male	15-19 years	95+ years	1	1
Pancreatic cancer	Cumulative Cigarettes (15 years)	Female	15-19 years	95+ years	1	NA
Pancreatic cancer	Cumulative Cigarettes (15 years)	Male	15-19 years	95+ years	1	NA
Pancreatic cancer	Cumulative Cigarettes (5 years)	Female	15-19 years	95+ years	1	NA
Pancreatic cancer	Cumulative Cigarettes (5 years)	Male	15-19 years	95+ years	1	NA
Pancreatic cancer	Smoking Prevalence	Female	15-19 years	95+ years	1	NA
Pancreatic cancer	Smoking Prevalence	Male	15-19 years	95+ years	1	NA
Pancreatic cancer	Diabetes Age-Standardized Prevalence (proportion)	Female	15-19 years	95+ years	2	2

Pancreatic cancer	Diabetes Age-Standardized Prevalence (proportion)	Male	15-19 years	95+ years	2	2
Pancreatic cancer	Diabetes Fasting Plasma Glucose (mmol/L), age-standardized 25+	Female	15-19 years	95+ years	NA	2
Pancreatic cancer	Diabetes Fasting Plasma Glucose (mmol/L), age-standardized 25+	Male	15-19 years	95+ years	NA	2
Pancreatic cancer	Education (years per capita)	Female	15-19 years	95+ years	3	3
Pancreatic cancer	Education (years per capita)	Male	15-19 years	95+ years	3	3
Pancreatic cancer	Healthcare access and quality index	Female	15-19 years	95+ years	2	2
Pancreatic cancer	Healthcare access and quality index	Male	15-19 years	95+ years	2	2
Pancreatic cancer	LDI (I$ per capita)	Female	15-19 years	95+ years	3	3
Pancreatic cancer	LDI (I$ per capita)	Male	15-19 years	95+ years	3	3
Pancreatic cancer	Liters of alcohol consumed per capita	Female	15-19 years	95+ years	1	2
Pancreatic cancer	Liters of alcohol consumed per capita	Male	15-19 years	95+ years	1	2
Pancreatic cancer	Log-transformed SEV scalar: Pancreas C	Female	15-19 years	95+ years	1	1
Pancreatic cancer	Log-transformed SEV scalar: Pancreas C	Male	15-19 years	95+ years	1	1
Pancreatic cancer	Mean BMI	Female	15-19 years	95+ years	1	1
Pancreatic cancer	Mean BMI	Male	15-19 years	95+ years	1	1
Pancreatic cancer	Socio-demographic Index	Female	15-19 years	95+ years	3	3
Pancreatic cancer	Socio-demographic Index	Male	15-19 years	95+ years	3	3
Pancreatic cancer	Tobacco (cigarettes per capita)	Female	15-19 years	95+ years	1	1
Pancreatic cancer	Tobacco (cigarettes per capita)	Male	15-19 years	95+ years	1	1
Pancreatic cancer	energy unadjusted(kcal)	Female	15-19 years	95+ years	2	2
Pancreatic cancer	energy unadjusted(kcal)	Male	15-19 years	95+ years	2	2
Prostate cancer	Education (years per capita)	Male	20-24 years	95+ years	3	3
Prostate cancer	Healthcare access and quality index	Male	20-24 years	95+ years	2	2
Prostate cancer	LDI (I$ per capita)	Male	20-24 years	95+ years	3	3
Prostate cancer	Log-transformed SEV scalar: Prostate C	Male	20-24 years	95+ years	1	1
Prostate cancer	Smoking Prevalence	Male	20-24 years	95+ years	NA	2
Prostate cancer	Socio-demographic Index	Male	20-24 years	95+ years	3	3
Stomach cancer	Age- and sex- specific SEV for low fruit	Female	15-19 years	95+ years	NA	3

Stomach cancer	Age- and sex- specific SEV for low fruit	Male	15-19 years	95+ years	NA	3
Stomach cancer	Age- and sex-specific SEV for Low vegetables	Female	15-19 years	95+ years	NA	3
Stomach cancer	Age- and sex-specific SEV for Low vegetables	Male	15-19 years	95+ years	NA	3
Stomach cancer	Fruits adjusted (g)	Female	15-19 years	95+ years	2	NA
Stomach cancer	Fruits adjusted (g)	Male	15-19 years	95+ years	2	NA
Stomach cancer	Vegetables adjusted (g)	Female	15-19 years	95+ years	2	NA
Stomach cancer	Vegetables adjusted (g)	Male	15-19 years	95+ years	2	NA
Stomach cancer	Age- and sex-specific SEV for Unsafe sanitation	Female	15-19 years	95+ years	NA	2
Stomach cancer	Age- and sex-specific SEV for Unsafe sanitation	Male	15-19 years	95+ years	NA	2
Stomach cancer	Age- and sex-specific SEV for Unsafe water	Female	15-19 years	95+ years	NA	2
Stomach cancer	Age- and sex-specific SEV for Unsafe water	Male	15-19 years	95+ years	NA	2
Stomach cancer	SEV unsafe water	Female	15-19 years	95+ years	1	NA
Stomach cancer	SEV unsafe water	Male	15-19 years	95+ years	1	NA
Stomach cancer	SEV unsafe sanitation	Female	15-19 years	95+ years	1	NA
Stomach cancer	SEV unsafe sanitation	Male	15-19 years	95+ years	1	NA
Stomach cancer	Smoking Prevalence	Female	15-19 years	95+ years	1	NA
Stomach cancer	Smoking Prevalence	Male	15-19 years	95+ years	1	NA
Stomach cancer	Cumulative Cigarettes (15 years)	Female	15-19 years	95+ years	1	NA
Stomach cancer	Cumulative Cigarettes (15 years)	Male	15-19 years	95+ years	1	NA
Stomach cancer	Cumulative Cigarettes (5 years)	Female	15-19 years	95+ years	1	NA
Stomach cancer	Cumulative Cigarettes (5 years)	Male	15-19 years	95+ years	1	NA
Stomach cancer	Cumulative Cigarettes (10 years)	Female	15-19 years	95+ years	1	NA
Stomach cancer	Cumulative Cigarettes (10 years)	Male	15-19 years	95+ years	1	NA
Stomach cancer	Cumulative Cigarettes (20 years)	Female	15-19 years	95+ years	1	2
Stomach cancer	Cumulative Cigarettes (20 years)	Male	15-19 years	95+ years	1	2
Stomach cancer	Diet high in sodium	Female	15-19 years	95+ years	1	1
Stomach cancer	Diet high in sodium	Male	15-19 years	95+ years	1	1
Stomach cancer	Education (years per capita)	Female	15-19 years	95+ years	3	3

Stomach cancer	Education (years per capita)	Male	15-19 years	95+ years	3	3
Stomach cancer	Healthcare access and quality index	Female	15-19 years	95+ years	2	2
Stomach cancer	Healthcare access and quality index	Male	15-19 years	95+ years	2	2
Stomach cancer	Improved Water Source (proportion with access)	Female	15-19 years	95+ years	2	2
Stomach cancer	Improved Water Source (proportion with access)	Male	15-19 years	95+ years	2	2
Stomach cancer	LDI (I$ per capita)	Female	15-19 years	95+ years	3	3
Stomach cancer	LDI (I$ per capita)	Male	15-19 years	95+ years	3	3
Stomach cancer	Log-transformed SEV scalar: Stomach C	Female	15-19 years	95+ years	1	1
Stomach cancer	Log-transformed SEV scalar: Stomach C	Male	15-19 years	95+ years	1	1
Stomach cancer	Mean BMI	Female	15-19 years	95+ years	2	2
Stomach cancer	Mean BMI	Male	15-19 years	95+ years	2	2
Stomach cancer	Sanitation (proportion with access)	Female	15-19 years	95+ years	2	2
Stomach cancer	Sanitation (proportion with access)	Male	15-19 years	95+ years	2	2
Stomach cancer	Socio-demographic Index	Female	15-19 years	95+ years	3	3
Stomach cancer	Socio-demographic Index	Male	15-19 years	95+ years	3	3
Stomach cancer	Tobacco (cigarettes per capita)	Female	15-19 years	95+ years	1	1
Stomach cancer	Tobacco (cigarettes per capita)	Male	15-19 years	95+ years	1	1
Testicular cancer	Age- and sex- specific SEV for low fruit	Male	0-6 days	95+ years	NA	2
Testicular cancer	Age- and sex-specific SEV for Low vegetables	Male	0-6 days	95+ years	NA	2
Testicular cancer	Fruits adjusted (g)	Male	0-6 days	95+ years	2	NA
Testicular cancer	Vegetables adjusted (g)	Male	0-6 days	95+ years	2	NA
Testicular cancer	Cumulative Cigarettes (10 years)	Male	0-6 days	95+ years	2	2
Testicular cancer	Cumulative Cigarettes (15 Years)	Male	0-6 days	95+ years	2	2
Testicular cancer	Cumulative Cigarettes (20 years)	Male	0-6 days	95+ years	2	2
Testicular cancer	Cumulative Cigarettes (5 Years)	Male	0-6 days	95+ years	2	2
Testicular cancer	Education (years per capita)	Male	0-6 days	95+ years	3	3
Testicular cancer	Healthcare access and quality index	Male	0-6 days	95+ years	2	2
Testicular cancer	LDI (I$ per capita)	Male	0-6 days	95+ years	3	3

Testicular cancer	Smoking Prevalence	Male	0-6 days	95+ years	2	2
Testicular cancer	Socio-demographic Index	Male	0-6 days	95+ years	3	3
Testicular cancer	Tobacco (cigarettes per capita)	Male	0-6 days	95+ years	2	2
Thyroid cancer	Age- and sex- specific SEV for low fruit	Female	5-9 years	95+ years	NA	3
Thyroid cancer	Age- and sex- specific SEV for low fruit	Male	5-9 years	95+ years	NA	3
Thyroid cancer	Age- and sex-specific SEV for High red meat	Female	5-9 years	95+ years	NA	2
Thyroid cancer	Age- and sex-specific SEV for High red meat	Male	5-9 years	95+ years	NA	2
Thyroid cancer	Age- and sex-specific SEV for Low vegetables	Female	5-9 years	95+ years	NA	2
Thyroid cancer	Age- and sex-specific SEV for Low vegetables	Male	5-9 years	95+ years	NA	2
Thyroid cancer	Fruits adjusted (g)	Female	5-9 years	95+ years	2	NA
Thyroid cancer	Fruits adjusted (g)	Male	5-9 years	95+ years	2	NA
Thyroid cancer	Red meats adjusted (g)	Female	5-9 years	95+ years	2	NA
Thyroid cancer	Red meats adjusted (g)	Male	5-9 years	95+ years	2	NA
Thyroid cancer	Vegetables adjusted (g)	Female	5-9 years	95+ years	2	NA
Thyroid cancer	Vegetables adjusted (g)	Male	5-9 years	95+ years	2	NA
Thyroid cancer	Education (years per capita)	Female	5-9 years	95+ years	3	3
Thyroid cancer	Education (years per capita)	Male	5-9 years	95+ years	3	3
Thyroid cancer	Healthcare access and quality index	Female	5-9 years	95+ years	2	2
Thyroid cancer	Healthcare access and quality index	Male	5-9 years	95+ years	2	2
Thyroid cancer	Improved Water Source (proportion with access)	Female	5-9 years	95+ years	2	3
Thyroid cancer	Improved Water Source (proportion with access)	Male	5-9 years	95+ years	2	3
Thyroid cancer	LDI (I$ per capita)	Female	5-9 years	95+ years	3	3
Thyroid cancer	LDI (I$ per capita)	Male	5-9 years	95+ years	3	3
Thyroid cancer	Liters of alcohol consumed per capita	Female	5-9 years	95+ years	1	1
Thyroid cancer	Liters of alcohol consumed per capita	Male	5-9 years	95+ years	1	1
Thyroid cancer	Log-transformed SEV scalar: Thyroid C	Female	5-9 years	95+ years	1	1
Thyroid cancer	Log-transformed SEV scalar: Thyroid C	Male	5-9 years	95+ years	1	1
Thyroid cancer	Mean BMI	Female	5-9 years	95+ years	2	2

Thyroid cancer	Mean BMI	Male	5-9 years	95+ years	2	2
Thyroid cancer	Sanitation (proportion with access)	Female	5-9 years	95+ years	2	3
Thyroid cancer	Sanitation (proportion with access)	Male	5-9 years	95+ years	2	3
Thyroid cancer	Socio-demographic Index	Female	5-9 years	95+ years	3	3
Thyroid cancer	Socio-demographic Index	Male	5-9 years	95+ years	3	3
Thyroid cancer	Smoking Prevalence	Female	5-9 years	95+ years	1	NA
Thyroid cancer	Smoking Prevalence	Male	5-9 years	95+ years	1	NA
Thyroid cancer	Tobacco (cigarettes per capita)	Female	5-9 years	95+ years	2	2
Thyroid cancer	Tobacco (cigarettes per capita)	Male	5-9 years	95+ years	2	2
Tracheal, bronchus, and lung cancer	Asbestos consumption (metric tons per year per capita)	Female	10-14 year	95+ years	NA	1
Tracheal, bronchus, and lung cancer	Asbestos consumption (metric tons per year per capita)	Male	10-14 year	95+ years	NA	1
Tracheal, bronchus, and lung cancer	Cumulative Cigarettes (10 years)	Female	10-14 year	95+ years	1	2
Tracheal, bronchus, and lung cancer	Cumulative Cigarettes (10 years)	Male	10-14 year	95+ years	1	2
Tracheal, bronchus, and lung cancer	Cumulative Cigarettes (20 years)	Female	10-14 year	95+ years	1	2
Tracheal, bronchus, and lung cancer	Cumulative Cigarettes (20 years)	Male	10-14 year	95+ years	1	2
Tracheal, bronchus, and lung cancer	Cumulative Cigarettes (15 years)	Female	10-14 year	95+ years	1	NA
Tracheal, bronchus, and lung cancer	Cumulative Cigarettes (15 years)	Male	10-14 year	95+ years	1	NA
Tracheal, bronchus, and lung cancer	Cumulative Cigarettes (5 years)	Female	10-14 year	95+ years	1	NA
Tracheal, bronchus, and lung cancer	Cumulative Cigarettes (5 years)	Male	10-14 year	95+ years	1	NA
Tracheal, bronchus, and lung cancer	Tobacco (cigarettes per capita)	Female	10-14 year	95+ years	1	NA
Tracheal, bronchus, and lung cancer	Tobacco (cigarettes per capita)	Male	10-14 year	95+ years	1	NA
Tracheal, bronchus, and lung cancer	Diabetes Fasting Plasma Glucose (mmol/L), age-standardized 25+	Female	10-14 year	95+ years	NA	2
Tracheal, bronchus, and lung cancer	Diabetes Fasting Plasma Glucose (mmol/L), age-standardized 25+	Male	10-14 year	95+ years	NA	2
Tracheal, bronchus, and lung cancer	Education (years per capita)	Female	10-14 year	95+ years	3	3
Tracheal, bronchus, and lung cancer	Education (years per capita)	Male	10-14 year	95+ years	3	3
Tracheal, bronchus, and lung cancer	Healthcare access and quality index	Female	10-14 year	95+ years	2	2
Tracheal, bronchus, and lung cancer	Healthcare access and quality index	Male	10-14 year	95+ years	2	2

Tracheal, bronchus, and lung cancer	Indoor Air Pollution (All Cooking Fuels)	Female	10-14 year	95+ years	2	2
Tracheal, bronchus, and lung cancer	Indoor Air Pollution (All Cooking Fuels)	Male	10-14 year	95+ years	2	2
Tracheal, bronchus, and lung cancer	LDI (I$ per capita)	Female	10-14 year	95+ years	3	3
Tracheal, bronchus, and lung cancer	LDI (I$ per capita)	Male	10-14 year	95+ years	3	3
Tracheal, bronchus, and lung cancer	Log-transformed SEV scalar: Lung C	Female	10-14 year	95+ years	1	1
Tracheal, bronchus, and lung cancer	Log-transformed SEV scalar: Lung C	Male	10-14 year	95+ years	1	1
Tracheal, bronchus, and lung cancer	Log-transformed age-standardized SEV scalar: Lung C	Female	10-14 year	95+ years	1	1
Tracheal, bronchus, and lung cancer	Log-transformed age-standardized SEV scalar: Lung C	Male	10-14 year	95+ years	1	1
Tracheal, bronchus, and lung cancer	Outdoor Air Pollution (PM2.5)	Female	10-14 year	95+ years	2	2
Tracheal, bronchus, and lung cancer	Outdoor Air Pollution (PM2.5)	Male	10-14 year	95+ years	2	2
Tracheal, bronchus, and lung cancer	Residential radon	Female	10-14 year	95+ years	NA	2
Tracheal, bronchus, and lung cancer	Residential radon	Male	10-14 year	95+ years	NA	2
Tracheal, bronchus, and lung cancer	Secondhand smoke	Female	10-14 year	95+ years	1	2
Tracheal, bronchus, and lung cancer	Secondhand smoke	Male	10-14 year	95+ years	1	2
Tracheal, bronchus, and lung cancer	Smoking Prevalence	Female	10-14 year	95+ years	1	1
Tracheal, bronchus, and lung cancer	Smoking Prevalence	Male	10-14 year	95+ years	1	1
Tracheal, bronchus, and lung cancer	Socio-demographic Index	Female	10-14 year	95+ years	3	3
Tracheal, bronchus, and lung cancer	Socio-demographic Index	Male	10-14 year	95+ years	3	3
Uterine cancer	Age- and sex- specific SEV for low fruit	Female	20-24 years	95+ years	NA	2
Uterine cancer	Age- and sex-specific SEV for Low vegetables	Female	20-24 years	95+ years	NA	2
Uterine cancer	Fruits adjusted (g)	Female	20-24 years	95+ years	2	NA
Uterine cancer	Vegetables adjusted (g)	Female	20-24 years	95+ years	2	NA
Uterine cancer	Cumulative Cigarettes (10 years)	Female	20-24 years	95+ years	2	2
Uterine cancer	Cumulative Cigarettes (5 Years)	Female	20-24 years	95+ years	2	2
Uterine cancer	Diabetes Age-Standardized Prevalence (proportion)	Female	20-24 years	95+ years	2	2
Uterine cancer	Education (years per capita)	Female	20-24 years	95+ years	3	3
Uterine cancer	Healthcare access and quality index	Female	20-24 years	95+ years	2	2
Uterine cancer	LDI (I$ per capita)	Female	20-24 years	95+ years	3	3

Uterine cancer	Log-transformed SEV scalar: Uterus C	Female	20-24 years	95+ years	1	1
Uterine cancer	Mean BMI	Female	20-24 years	95+ years	1	1
Uterine cancer	Smoking Prevalence	Female	20-24 years	95+ years	2	2
Uterine cancer	Socio-demographic Index	Female	20-24 years	95+ years	3	3
Uterine cancer	Tobacco (cigarettes per capita)	Female	20-24 years	95+ years	2	2
Uterine cancer	Total Fertility Rate	Female	20-24 years	95+ years	2	2
Uterine cancer	Tobacco (cigarettes per capita)	Female	20-24 years	95+ years	2	NA

Abbreviations: BMI, body mass index; DR, data rich; GBD, Global Burden of Disease Study; GLB, global; HBsAg, hepatitis B surface antigen; HCV, hepatitis C virus; LDI, lag-distributed income; MET, metabolic equivalent of task; NA, not applicable; PM2.5, particulate matter ≤ 2.5 micrometers; PUFA, polyunsaturated fatty acid; SEV, summary exposure value.

eTable 9: Results for CODEm model performance testing

Cause	Sex	RMSE in	RMSE out	Trend in	Trend out	Coverage in	Coverage out
Acute lymphoid leukemia [Data Rich]	Male	0.310748	0.421052	0.257037	0.250826	0.999775	0.999201
Acute lymphoid leukemia [Global]	Male	0.340238	0.481488	0.269561	0.266842	0.999598	0.996258
Acute lymphoid leukemia [Data Rich]	Female	0.330709	0.469462	0.291051	0.295814	0.999639	0.99753
Acute lymphoid leukemia [Global]	Female	0.387468	0.532984	0.309633	0.305578	0.999247	0.995595
Acute myeloid leukemia [Data Rich]	Male	0.41862	0.467416	0.371762	0.271103	0.998507	0.997028
Acute myeloid leukemia [Global]	Male	0.442375	0.54861	0.372251	0.356678	0.998107	0.996117
Acute myeloid leukemia [Data Rich]	Female	0.394568	0.448862	0.352312	0.249893	0.998964	0.997453
Acute myeloid leukemia [Global]	Female	0.407571	0.523704	0.353383	0.33313	0.998587	0.995357
Bladder cancer [Data Rich]	Male	0.240898	0.285527	0.199469	0.213802	0.998131	0.996401
Bladder cancer [Global]	Male	0.274484	0.368138	0.225794	0.224493	0.998225	0.990777
Bladder cancer [Data Rich]	Female	0.219065	0.27044	0.179335	0.199428	0.99817	0.997553
Bladder cancer [Global]	Female	0.253852	0.361089	0.201613	0.206813	0.998074	0.995339
Brain and central nervous system cancer [Data Rich]	Male	0.256468	0.339441	0.198232	0.243717	0.998414	0.997352
Brain and central nervous system cancer [Global]	Male	0.317265	0.455977	0.221404	0.227775	0.998775	0.992953
Brain and central nervous system cancer [Data Rich]	Female	0.275224	0.35502	0.190857	0.22379	0.99882	0.997871
Brain and central nervous system cancer [Global]	Female	0.301083	0.44498	0.2191	0.22137	0.99905	0.994921
Breast cancer [Data Rich]	Male	0.706904	0.839615	0.646192	0.755323	0.928699	0.92097
Breast cancer [Global]	Male	0.769967	0.895063	0.67604	0.665045	0.939103	0.925064
Breast cancer [Data Rich]	Female	0.190097	0.238463	0.156457	0.180158	0.995296	0.992005
Breast cancer [Global]	Female	0.213415	0.292349	0.168595	0.171745	0.995641	0.986556
Cervical cancer [Data Rich]	Female	0.205345	0.259868	0.169338	0.203447	0.998678	0.997579
Cervical cancer [Global]	Female	0.242419	0.360121	0.195079	0.201005	0.999068	0.991885
Chronic lymphoid leukemia [Data Rich]	Male	0.281179	0.549864	0.215486	0.224895	0.993027	0.986267
Chronic lymphoid leukemia [Global]	Male	0.320083	0.610391	0.242085	0.241267	0.992505	0.982641
Chronic lymphoid leukemia [Data Rich]	Female	0.39135	0.586716	0.370043	0.380051	0.988081	0.980056
Chronic lymphoid leukemia [Global]	Female	0.412761	0.641749	0.374616	0.418784	0.989395	0.977703
Chronic myeloid leukemia [Data Rich]	Male	0.337209	0.602014	0.292333	0.283856	0.985279	0.98508

Chronic myeloid leukemia [Global]	Male	0.367785	0.691518	0.303339	0.313485	0.988074	0.985079
Chronic myeloid leukemia [Data Rich]	Female	0.350665	0.590275	0.289082	0.278623	0.984769	0.985895
Chronic myeloid leukemia [Global]	Female	0.398875	0.739473	0.313448	0.315678	0.987321	0.984064
Colon and rectum cancer [Data Rich]	Male	0.190946	0.24147	0.157788	0.159771	0.995286	0.992178
Colon and rectum cancer [Global]	Male	0.227892	0.304014	0.184744	0.177172	0.996427	0.989429
Colon and rectum cancer [Data Rich]	Female	0.18306	0.231029	0.151894	0.158584	0.99588	0.993197
Colon and rectum cancer [Global]	Female	0.206598	0.280277	0.168597	0.167246	0.996496	0.991145
Esophageal cancer [Data Rich]	Male	0.197786	0.243235	0.162392	0.178707	0.998139	0.996859
Esophageal cancer [Global]	Male	0.240983	0.396882	0.188189	0.190213	0.998341	0.981691
Esophageal cancer [Data Rich]	Female	0.216536	0.272917	0.181223	0.22027	0.99904	0.998262
Esophageal cancer [Global]	Female	0.262599	0.439	0.209188	0.207226	0.998757	0.984349
Gallbladder and biliary tract cancer [Data Rich]	Male	0.210762	0.257952	0.170754	0.179093	0.99896	0.997991
Gallbladder and biliary tract cancer [Global]	Male	0.251943	0.357674	0.200561	0.195415	0.999264	0.992854
Gallbladder and biliary tract cancer [Data Rich]	Female	0.213419	0.254766	0.168672	0.176916	0.998906	0.997939
Gallbladder and biliary tract cancer [Global]	Female	0.239816	0.352588	0.187002	0.183674	0.998964	0.988385
Hodgkin lymphoma [Data Rich]	Male	0.338556	0.440404	0.288515	0.347947	0.997264	0.997151
Hodgkin lymphoma [Global]	Male	0.403778	0.527964	0.308644	0.308939	0.999444	0.995815
Hodgkin lymphoma [Data Rich]	Female	0.425215	0.529984	0.29507	0.366626	0.990635	0.991235
Hodgkin lymphoma [Global]	Female	0.460638	0.612558	0.325305	0.337065	0.992827	0.987712
Kidney cancer [Data Rich]	Male	0.270148	0.374426	0.231028	0.292066	0.997861	0.996453
Kidney cancer [Global]	Male	0.28997	0.414506	0.23986	0.255253	0.998188	0.99382
Kidney cancer [Data Rich]	Female	0.282258	0.435576	0.234894	0.322233	0.99821	0.996758
Kidney cancer [Global]	Female	0.305091	0.46851	0.244577	0.267581	0.99812	0.993281
Larynx cancer [Data Rich]	Male	0.220774	0.265656	0.184647	0.198589	0.999327	0.998863
Larynx cancer [Global]	Male	0.267476	0.391878	0.214871	0.213209	0.999372	0.990982
Larynx cancer [Data Rich]	Female	0.290484	0.349996	0.246702	0.27534	0.993277	0.993692
Larynx cancer [Global]	Female	0.359559	0.514848	0.290491	0.292231	0.994598	0.989664
Leukemia [Data Rich]	Male	0.214077	0.263591	0.186937	0.222432	0.999145	0.99826
Leukemia [Global]	Male	0.271541	0.335096	0.211088	0.213378	0.999578	0.99719
Leukemia [Data Rich]	Female	0.198555	0.244279	0.170536	0.197341	0.999169	0.998474

Leukemia [Global]	Female	0.253005	0.323649	0.211247	0.20947	0.999523	0.997907
Lip and oral cavity cancer [Data Rich]	Male	0.254061	0.321112	0.216056	0.205439	0.997306	0.995437
Lip and oral cavity cancer [Global]	Male	0.268411	0.385903	0.22366	0.222025	0.997854	0.993797
Lip and oral cavity cancer [Data Rich]	Female	0.217157	0.297755	0.177057	0.17384	0.998593	0.997568
Lip and oral cavity cancer [Global]	Female	0.245299	0.35369	0.191169	0.192107	0.998795	0.996958
Liver cancer [Data Rich]	Male	0.277096	0.371486	0.229624	0.264623	0.996156	0.993083
Liver cancer [Global]	Male	0.32687	0.631061	0.266922	0.270088	0.995476	0.972126
Liver cancer [Data Rich]	Female	0.316171	0.408322	0.22516	0.260284	0.99793	0.995804
Liver cancer [Global]	Female	0.32009	0.590435	0.259578	0.266162	0.997457	0.981469
Malignant skin melanoma [Data Rich]	Male	0.282741	0.405971	0.221329	0.232564	0.998476	0.997567
Malignant skin melanoma [Global]	Male	0.341866	0.461807	0.244628	0.239671	0.99866	0.993613
Malignant skin melanoma [Data Rich]	Female	0.248595	0.333716	0.193576	0.193995	0.999166	0.998469
Malignant skin melanoma [Global]	Female	0.323326	0.441126	0.225913	0.220791	0.99899	0.995255
Mesothelioma [Data Rich]	Male	0.200856	0.360612	0.175292	0.234068	0.999987	0.999943
Mesothelioma [Global]	Male	0.264438	0.437326	0.19753	0.214515	0.999683	0.996791
Mesothelioma [Data Rich]	Female	0.455723	0.910995	0.454968	0.689974	0.999968	0.991245
Mesothelioma [Global]	Female	0.440471	0.744811	0.415638	0.499273	0.998004	0.987963
Multiple myeloma [Data Rich]	Male	0.232883	0.275872	0.189341	0.190352	0.999453	0.999132
Multiple myeloma [Global]	Male	0.299219	0.383876	0.223369	0.218695	0.999491	0.995722
Multiple myeloma [Data Rich]	Female	0.242432	0.287812	0.200849	0.200434	0.999643	0.999242
Multiple myeloma [Global]	Female	0.304571	0.388807	0.226423	0.228057	0.999523	0.995996
Nasopharynx cancer [Data Rich]	Male	0.277888	0.328338	0.243308	0.246639	0.999822	0.999556
Nasopharynx cancer [Global]	Male	0.303888	0.463655	0.255738	0.252325	0.999663	0.990558
Nasopharynx cancer [Data Rich]	Female	0.301697	0.369424	0.259233	0.289024	0.995143	0.994079
Nasopharynx cancer [Global]	Female	0.33418	0.503304	0.273791	0.274855	0.994869	0.987136
Non-Hodgkin lymphoma [Data Rich]	Male	0.190296	0.26584	0.155396	0.176023	0.998955	0.997787
Non-Hodgkin lymphoma [Global]	Male	0.212483	0.326477	0.164107	0.162797	0.99916	0.995587
Non-Hodgkin lymphoma [Data Rich]	Female	0.1921	0.275211	0.158491	0.182009	0.99926	0.998202
Non-Hodgkin lymphoma [Global]	Female	0.218556	0.335889	0.168391	0.166175	0.999419	0.996138

Non-melanoma skin cancer (squamous-cell carcinoma) [Data Rich]	Male	0.187014	0.271342	0.132362	0.153868	0.999799	0.999579
Non-melanoma skin cancer (squamous-cell carcinoma) [Global]	Male	0.215805	0.354613	0.150469	0.152022	0.999798	0.997394
Non-melanoma skin cancer (squamous-cell carcinoma) [Data Rich]	Female	0.213103	0.305569	0.15533	0.168031	0.999125	0.998895
Non-melanoma skin cancer (squamous-cell carcinoma) [Global]	Female	0.267942	0.451328	0.188854	0.185703	0.998427	0.992207
Other leukemia [Data Rich]	Male	0.282494	0.381942	0.241361	0.229578	0.999734	0.999253
Other leukemia [Global]	Male	0.309665	0.463243	0.251833	0.245941	0.99957	0.993564
Other leukemia [Data Rich]	Female	0.279342	0.386578	0.238006	0.240633	0.999753	0.999095
Other leukemia [Global]	Female	0.337468	0.46428	0.252373	0.245634	0.999625	0.994124
Other malignant neoplasms [Data Rich]	Male	0.209241	0.256764	0.172726	0.190063	0.996091	0.993792
Other malignant neoplasms [Global]	Male	0.249786	0.354058	0.189328	0.193065	0.997607	0.991761
Other malignant neoplasms [Data Rich]	Female	0.198916	0.238695	0.166856	0.173074	0.99734	0.994796
Other malignant neoplasms [Global]	Female	0.220775	0.335985	0.175327	0.179505	0.997653	0.993175
Other pharynx cancer [Data Rich]	Male	0.244485	0.307088	0.20426	0.213738	0.999408	0.998881
Other pharynx cancer [Global]	Male	0.277714	0.443553	0.222129	0.217025	0.999432	0.991696
Other pharynx cancer [Data Rich]	Female	0.280274	0.3384	0.238221	0.250264	0.998726	0.998526
Other pharynx cancer [Global]	Female	0.309356	0.441873	0.253513	0.247686	0.999056	0.996806
Ovarian cancer [Data Rich]	Female	0.207516	0.273279	0.168154	0.184435	0.998087	0.996715
Ovarian cancer [Global]	Female	0.240434	0.348621	0.180684	0.182016	0.998618	0.994472
Pancreatic cancer [Data Rich]	Male	0.208363	0.263915	0.171769	0.200205	0.99645	0.994616
Pancreatic cancer [Global]	Male	0.243467	0.353046	0.195591	0.196526	0.99724	0.992451
Pancreatic cancer [Data Rich]	Female	0.211322	0.264799	0.179225	0.205886	0.997031	0.995275
Pancreatic cancer [Global]	Female	0.229988	0.327964	0.18741	0.191431	0.997558	0.994161
Prostate cancer [Data Rich]	Male	0.1923	0.229505	0.147543	0.160288	0.997635	0.995545
Prostate cancer [Global]	Male	0.219589	0.279819	0.155811	0.152698	0.997501	0.991061
Stomach cancer [Data Rich]	Male	0.174823	0.216585	0.143368	0.163153	0.996825	0.994387
Stomach cancer [Global]	Male	0.221437	0.342098	0.175544	0.175098	0.997261	0.97773
Stomach cancer [Data Rich]	Female	0.172379	0.213413	0.142051	0.160748	0.997414	0.995481

Stomach cancer [Global]	Female	0.224046	0.327492	0.176793	0.173164	0.997996	0.984052
Testicular cancer [Data Rich]	Male	0.338756	0.466129	0.286761	0.281068	0.999256	0.998529
Testicular cancer [Global]	Male	0.452611	0.643414	0.381982	0.385017	0.995095	0.989925
Thyroid cancer [Data Rich]	Male	0.301986	0.364563	0.25634	0.275841	0.999081	0.998499
Thyroid cancer [Global]	Male	0.325978	0.451676	0.268093	0.275032	0.999028	0.997434
Thyroid cancer [Data Rich]	Female	0.374735	0.424526	0.329318	0.307338	0.994602	0.992091
Thyroid cancer [Global]	Female	0.395627	0.504645	0.330969	0.329624	0.995041	0.991576
Tracheal, bronchus, and lung cancer [Data Rich]	Male	0.20052	0.252085	0.164035	0.168916	0.995802	0.992387
Tracheal, bronchus, and lung cancer [Global]	Male	0.256649	0.350376	0.202454	0.193446	0.997257	0.979902
Tracheal, bronchus, and lung cancer [Data Rich]	Female	0.211586	0.278726	0.1735	0.187087	0.997126	0.994813
Tracheal, bronchus, and lung cancer [Global]	Female	0.259003	0.380143	0.20628	0.196712	0.998099	0.984358
Uterine cancer [Data Rich]	Female	0.228228	0.279147	0.190758	0.20872	0.999054	0.998101
Uterine cancer [Global]	Female	0.31705	0.413482	0.255661	0.25166	0.9992	0.994406

Abbreviations: RMSE, root mean squared error.

Footnote: RMSE measures the deviation between the data and the model estimate. Trend refers to the percent of predictions that correctly predict the direction of the time trend from adjacent points. Coverage refers to the percent of data that are included within the ensemble uncertainty interval. For performance testing, data are split into training and out-of-sample testing sets. Columns with “in” refer to the performance within the training sample data, while “out” refer to the performance of the ensemble within the withheld out-of-sample data. “Global” models include all locations, while “Data Rich” models include a subset of locations with high-quality data.

eTable 10: Percent change before and after CoDCorrect by cancer for all ages, both sexes combined, 2019

Cause	CoDCorrect level	Percent change (95% uncertainty interval)
Neoplasms	2	1.74 (-3.24 to 4.88)
Bladder cancer	3	1.56 (-5.03 to 5.33)
Brain and central nervous system cancer	3	1.41 (-1.74 to 4.93)
Breast cancer	3	2.12 (-3.22 to 5.63)
Cervical cancer	3	3.36 (-0.98 to 7.20)
Colon and rectum cancer	3	1.4 (-4.72 to 4.84)
Esophageal cancer	3	1.66 (-2.51 to 4.87)
Gallbladder and biliary tract cancer	3	1.96 (-5.22 to 6.36)
Hodgkin lymphoma	3	5.82 (1.76 to 9.73)
Kidney cancer	3	0.89 (-4.18 to 3.85)
Larynx cancer	3	4.03 (0.75 to 7.15)
Leukemia	3	2.29 (-2.23 to 5.78)
Lip and oral cavity cancer	3	4.93 (0.68 to 8.65)
Liver cancer	3	1.19 (-3.38 to 4.41)
Malignant skin melanoma	3	0.86 (-4.06 to 3.72)
Mesothelioma	3	0.99 (-4.19 to 3.99)
Multiple myeloma	3	1.23 (-4.67 to 4.60)
Nasopharynx cancer	3	3.53 (0.64 to 7.06)
Non-Hodgkin lymphoma	3	2.06 (-3.12 to 5.64)
Non-melanoma skin cancer	3	1.56 (-6.01 to 5.78)

Other malignant neoplasms	3	3.71<br>(-0.73 to 7.64)
Other pharynx cancer	3	5.54<br>(2.32 to 9.49)
Ovarian cancer	3	1.46<br>(-3.95 to 5.04)
Pancreatic cancer	3	0.85<br>(-5.11 to 4.06)
Prostate cancer	3	2.13<br>(-4.09 to 5.99)
Stomach cancer	3	1.88<br>(-3.22 to 5.18)
Testicular cancer	3	5.8<br>(2.71 to 10.87)
Thyroid cancer	3	3.33<br>(-2.39 to 7.08)
Tracheal, bronchus, and lung cancer	3	0.72<br>(-3.95 to 3.60)
Uterine cancer	3	1.41<br>(-4.77 to 4.97)

eTable 11: Duration of four prevalence phases by cancer

Cancer	Diagnosis/ Treatment (months)	Remission (months)	Disseminated/ metastatic (months)	Terminal (months)	Note for metastatic phase
Esophageal cancer	5.0 [33]	*	4.6 [34]	1	SEER Summary Stage 1977 (Distant site/node involved)
Stomach cancer	5.2 [33]	*	3.9 [34]	1	SEER Summary Stage 1977 (Distant site/node involved) 1995-2000
Liver cancer	4.0	*	2.5 [34]	1	SEER Summary Stage 1977 (Distant site/node involved)
Larynx cancer	5.3 [33]	*	8.8 [34]	1	SEER Stage IVc
Tracheal, bronchus, and lung cancer	3.3 [35]	*	4.5 [34]	1	SEER Summary Stage 1977 (Distant site/node involved) 1995-2000
Breast cancer	3.0 [35]	*	17.7 [34]	1	SEER Summary Stage 1977 (Distant site/node involved) 1995-2000
Cervical cancer	4.8 [33]	*	9.2 [34]	1	SEER Summary Stage 1977 (Distant site/node involved) 1995-2000
Uterine cancer	4.6 [33]	*	11.6 [34]	1	SEER Summary Stage 1977 (Distant site/node involved) 1995-2000
Prostate cancer	4.0 [35]	*	30.4 [34]	1	SEER Summary Stage 1977 (Distant site/node involved) 1995-2000
Colon and rectum cancer	4.0 [35]	*	9.7 [34]	1	SEER Summary Stage 1977 (Distant site/node involved)
Lip and oral cavity cancers	5.3 [33]	*	9.3 [34]	1	SEER Stage IVc
Nasopharynx cancer	5.3 [33]	*	9.3 [34]	1	SEER Stage IVc
Other phyarnx cancer	5.3 [33]	*	13.2 [34]	1	SEER Stage IVc
Gallbladder and biliary tract cancer	4.0	*	3.5 [34]	1	SEER Summary Stage 1977 (Distant site/node involved) 1995-2000
Pancreatic cancer	4.1 [33]	*	2.5 [34]	1	SEER Summary Stage 1977 (Distant site/node involved) 1995-2000
Malignant skin melanoma	2.9 [36]	*	7.2 [34]	1	SEER Summary Stage 1977 (Distant site/node involved) 1995-2000
Ovarian cancer	3.2 [35]	*	25.6 [34]	1	SEER Summary Stage 1977 (Distant site/node involved) 1995-2000
Testicular cancer	3.7 [33]	*	19.5 [34]	1	SEER Stage III

Kidney cancer	5.3 [33]	*	5.4 [34]	1	SEER Summary Stage 1977 (Distant site/node involved) 1995-2000
Bladder cancer	5.1 [33]	*	5.8 [34]	1	SEER Summary Stage 1977 (Distant site/node involved) 1995-2000
Brain and central nervous system cancer	5.0	*	6.9 [34]	1	SEER median age standardized survival all patients, all years
Thyroid cancer	3.0	*	19.4 [34]	1	SEER Stage IVc
Mesothelioma	4.0	*	7.8 [34]	1	SEER Summary Stage 1977 (Distant site/node involved) 1995-2000
Hodgkin lymphoma	3.7 [35]	*	26.0 [37]	1	
Non-Hodgkin lymphoma	3.7 [35]	*	7.7 [37]	1	
Multiple myeloma	7.0 [33]	*	36.8 [34]	1	SEER median age standardized survival all patients, all years
Leukemia	5.0 [33]	*	43.7 [34]	1	SEER median age standardized survival all patients, all years
Acute lymphoid leukemia	12.0	*	7.0 [34]	1	SEER median age standardized survival all patients, all years
Acute myeloid leukemia	6.0	*	4.6 [34]	1	SEER median age standardized survival all patients, all years
Chronic lymphoid leukemia	6.0	*	48.0 [38]	1	SEER median age standardized survival all patients, all years
Chronic myeloid leukemia	6.0	*	4.6 [34]	1	SEER median age standardized survival for AML (patients wit CML die in blast crisis, which is treated like AML) all patients, all years
Other leukemia	6.0	*	48.0 [38]	1	SEER median age standardized survival all patients, all years
Other malignant neoplasms	4.4 (mean of other cancer durations)	*	15.8 [34]	1	SEER median age standardized survival all patients, all years

Footnote: The remission phase duration is calculated based on the remaining time after attributing other sequelae durations. Superscripts refer to references used to inform these values.

eTable 12: Disability weights

**Health state**	**Lay description**	**Disability weight (95% uncertainty interval)**
Cancer, diagnosis and primary therapy *All cancers except non-melanoma skin cancer*	This person has pain, nausea, fatigue, weight loss and high anxiety.	0.288 (0.193 to 0.399)
Cancer, controlled phase *All cancers except non-melanoma skin cancer*	This person has a chronic disease that requires medication every day and causes some worry but minimal interference with daily activities.	0.049 (0.031 to 0.072)
Cancer, metastatic *All cancers except non-melanoma skin cancer*	This person has severe pain, extreme fatigue, weight loss and high anxiety.	0.451 (0.307 to 0.600)
Terminal phase, with medication *All cancers except non-melanoma skin cancer*	This person has lost a lot of weight and regularly uses strong medication to avoid constant pain. The person has no appetite, feels nauseous, and needs to spend most of the day in bed.	0.540 (0.377 to 0.687)
Generic uncomplicated disease: worry and daily medication *Myelodysplastic, myeloproliferative, and other hematopoietic neoplasms*	This person has a chronic disease that requires medication every day and causes some worry but minimal interference with daily activities.	0.049 (0.031 to 0.072)
Mastectomy *Breast cancer*	This person had one of her breasts removed and sometimes has pain or swelling in the arms.	0.036 (0.020 to 0.057)
Stoma *Colon and rectum cancer*	This person has a pouch attached to an opening in the belly to collect and empty stools.	0.095 (0.063 to 0.131)
Laryngectomy *Larynx cancer*	This person has difficulty speaking, and others find it difficult to understand.	0.051 (0.032 to 0.078)
Urinary incontinence *Bladder cancer; Prostate cancer*	This person cannot control urinating.	0.139 (0.094 to 0.198)

Impotence<br>*Prostate cancer*	This person has difficulty in obtaining or maintaining an erection.	0.017<br>(0.009 to 0.030)
Disfigurement,<br>level 1<br>*Cutaneous squamous cell carcinoma; Basal cell carcinoma*	This person has a slight, visible physical deformity that others notice, which causes some worry and discomfort.	0.011<br>(0.005 to 0.021)
Disfigurement,<br>level 2<br>*Cutaneous squamous cell carcinoma*	This person has a visible physical deformity that causes others to stare and comment. As a result, the person is worried and has trouble sleeping and concentrating.	0.067<br>(0.044 to 0.096)
Disfigurement,<br>level 3, with itch/pain<br>*Cutaneous squamous cell carcinoma*	This person has an obvious physical deformity that is very painful and itchy. The physical deformity makes others uncomfortable, which causes the person to avoid social contact, feel worried, sleep poorly, and think about suicide.	0.576<br>(0.401 to 0.731)

eTable 13: GATHER[5] guidelines checklist

Item #	Checklist item	Reported on page #
**Objectives and funding**		
1	Define the indicator(s), populations (including age, sex, and geographic entities), and time period(s) for which estimates were made.	Appendix pg. 5
2	List the funding sources for the work.	See main manuscript
**Data Inputs**		
For all data inputs from multiple sources that are synthesized as part of the study:		
3	Describe how the data were identified and how the data were accessed.	Appendix pg. 6
4	Specify the inclusion and exclusion criteria. Identify all ad-hoc exclusions.	Appendix pg. 6
5	Provide information on all included data sources and their main characteristics. For each data source used, report reference information or contact name/institution, population represented, data collection method, year(s) of data collection, sex and age range, diagnostic criteria or measurement method, and sample size, as relevant.	http://ghdx.healthdata.org/gbd-2019
6	Identify and describe any categories of input data that have potentially important biases (e.g., based on characteristics listed in item 5).	Appendix pg. 7
For data inputs that contribute to the analysis but were not synthesized as part of the study:		
7	Describe and give sources for any other data inputs.	http://ghdx.healthdata.org/gbd-2019
For all data inputs:		
8	Provide all data inputs in a file format from which data can be efficiently extracted (e.g., a spreadsheet rather than a PDF), including all relevant meta-data listed in item 5. For any data inputs that cannot be shared because of ethical or legal reasons, such as third-party ownership, provide a contact name or the name of the institution that retains the right to the data.	http://ghdx.healthdata.org/gbd-2019
**Data analysis**		
9	Provide a conceptual overview of the data analysis method. A diagram may be helpful.	Appendix pg. 201-202 (eFigures 2 & 3)
10	Provide a detailed description of all steps of the analysis, including mathematical formulae. This description should cover, as relevant, data cleaning, data pre-processing, data adjustments and weighting of data sources, and mathematical or statistical model(s).	Appendix pg. 7-17
11	Describe how candidate models were evaluated and how the final model(s) were selected.	Found in *Section 3: Causes of death modelling methods* of the Supplementary appendix 1 to "GBD 2019 Diseases and Injuries Collaborators. Global burden of 369 diseases and injuries in 204 countries and territories, 1990–2019: a systematic

		analysis for the Global Burden of Disease Study 2019."[2] Details of covariate selection for cancer models can be found in: Appendix pg. 84-110 (eTable7)
12	Provide the results of an evaluation of model performance, if done, as well as the results of any relevant sensitivity analysis.	Appendix pg. 149-153 (eTable 9)
13	Describe methods for calculating uncertainty of the estimates. State which sources of uncertainty were, and were not, accounted for in the uncertainty analysis.	Appendix pg. 12
14	State how analytic or statistical source code used to generate estimates can be accessed.	http://ghdx.healthdata.org/gbd-2019/code
Results and Discussion		
15	Provide published estimates in a file format from which data can be efficiently extracted.	GBD 2019 estimates are available online (https://vizhub.healthdata.org/gbd-compare/ and http://ghdx.healthdata.org/gbd-results-tool)
16	Report a quantitative measure of the uncertainty of the estimates (e.g. uncertainty intervals).	See main manuscript, "Results"
17	Interpret results in light of existing evidence. If updating a previous set of estimates, describe the reasons for changes in estimates.	See main manuscript, "Discussion"
18	Discuss limitations of the estimates. Include a discussion of any modelling assumptions or data limitations that affect interpretation of the estimates.	See main manuscript, "Limitations"

eTable 14: Contribution of YLDs and YLLs to DALYs by cancer, global, both sexes, 2019

Cause	Contribution YLDs, % (95% UI)	Contribution YLLs, % (95% UI)
Total cancers	3.1 (2.3 - 4.0)	96.9 (96.0 - 97.7)
Lip and oral cavity cancer	2.6 (1.9 - 3.4)	97.4 (96.6 - 98.1)
Nasopharynx cancer	4.1 (2.9 - 5.6)	95.9 (94.4 - 97.1)
Other pharynx cancer	1.5 (1.0 - 1.9)	98.5 (98.1 - 99.0)
Esophageal cancer	1.3 (0.9 - 1.7)	98.7 (98.3 - 99.1)
Stomach cancer	1.6 (1.1 - 2.0)	98.4 (98.0 - 98.9)
Colon and rectum cancer	4.4 (3.2 - 5.6)	95.6 (94.4 - 96.8)
Liver cancer	1.0 (0.7 - 1.4)	99.0 (98.6 - 99.3)
Gallbladder and biliary tract cancer	1.3 (1.0 - 1.7)	98.7 (98.3 - 99.0)
Pancreatic cancer	0.9 (0.7 - 1.2)	99.1 (98.8 - 99.3)
Larynx cancer	3.6 (2.6 - 4.6)	96.4 (95.4 - 97.4)
Tracheal, bronchus, and lung cancer	1.2 (0.9 - 1.5)	98.8 (98.5 - 99.1)
Malignant skin melanoma	7.7 (5.5 - 10.1)	92.3 (89.9 - 94.5)
Non-melanoma skin cancer	10.6 (7.2 - 14.8)	89.4 (85.2 - 92.8)
Breast cancer	6.7 (4.8 - 8.9)	93.3 (91.1 - 95.2)
Cervical cancer	2.7 (2.0 - 3.6)	97.3 (96.4 - 98.0)
Uterine cancer	9.4 (6.8 - 12.2)	90.6 (87.8 - 93.2)
Ovarian cancer	2.9 (2.1 - 3.6)	97.1 (96.4 - 97.9)
Prostate cancer	10.4 (7.8 - 13.1)	89.6 (86.9 - 92.2)
Testicular cancer	9.5 (6.4 - 13.4)	90.5 (86.6 - 93.6)
Kidney cancer	3.7 (2.7 - 4.9)	96.3 (95.1 - 97.3)
Bladder cancer	6.2 (4.6 - 8.0)	93.8 (92.0 - 95.4)
Brain and central nervous system cancer	1.5 (1.1 - 2.0)	98.5 (98.0 - 98.9)
Thyroid cancer	9.2 (6.6 - 12.3)	90.8 (87.7 - 93.4)
Mesothelioma	1.9 (1.3 - 2.4)	98.1 (97.6 - 98.7)
Hodgkin lymphoma	4.1 (3.0 - 5.4)	95.9 (94.6 - 97.0)
Non-Hodgkin lymphoma	4.0 (2.9 - 5.1)	96.0 (94.9 - 97.1)
Multiple myeloma	3.8 (2.7 - 4.8)	96.2 (95.2 - 97.3)
Leukemia	3.1 (2.3 - 4.0)	96.9 (96.0 - 97.7)
Acute lymphoid leukemia	3.5 (2.6 - 4.7)	96.5 (95.3 - 97.4)
Chronic lymphoid leukemia	8.1 (6.0 - 10.3)	91.9 (89.7 - 94.0)
Acute myeloid leukemia	1.1 (0.8 - 1.5)	98.9 (98.5 - 99.2)
Chronic myeloid leukemia	2.4 (1.7 - 3.2)	97.6 (96.8 - 98.3)
Other leukemia	3.2 (2.4 - 4.2)	96.8 (95.8 - 97.6)
Other malignant neoplasms	2.8 (2.0 - 3.7)	97.2 (96.3 - 98.0)

Abbreviations: 95% UI, 95% uncertainty interval; DALY, disability-adjusted life year; YLD, years lived with disability; YLL, years of life lost.

Footnote: "Total cancers" in this table includes non-melanoma skin cancer, and excludes the "Other neoplasms" category of benign and in situ neoplasms.

eTable 15: Global number of incidence, prevalence, YLDs, deaths, YLLs, DALYs for both sexes, 2010 and 2019 for all level 2 GBD causes

Measure	Cause Name	Number, 2010 (95% UI)	Number, 2019 (95% UI)
**DALYs**	**Total cancers**	**216,000,000 (208,000,000 - 223,000,000)**	**250,000,000 (235,000,000 - 264,000,000)**
DALYs	Cardiovascular diseases	350,000,000 (335,000,000 - 363,000,000)	393,000,000 (368,000,000 - 417,000,000)
DALYs	Chronic respiratory diseases	93,600,000 (86,200,000 - 100,000,000)	104,000,000 (94,800,000 - 112,000,000)
DALYs	Diabetes and kidney diseases	87,700,000 (77,700,000 - 98,400,000)	113,000,000 (99,300,000 - 128,000,000)
DALYs	Digestive diseases	83,600,000 (78,400,000 - 89,700,000)	89,000,000 (81,400,000 - 97,600,000)
DALYs	Enteric infections	132,000,000 (113,000,000 - 156,000,000)	96,800,000 (79,200,000 - 120,000,000)
DALYs	HIV/AIDS and sexually transmitted infections	86,000,000 (74,200,000 - 103,000,000)	56,200,000 (48,400,000 - 67,000,000)
DALYs	Maternal and neonatal disorders	244,000,000 (224,000,000 - 266,000,000)	199,000,000 (172,000,000 - 232,000,000)
DALYs	Mental disorders	111,000,000 (82,300,000 - 146,000,000)	125,000,000 (93,000,000 - 163,000,000)
DALYs	Musculoskeletal disorders	125,000,000 (90,800,000 - 165,000,000)	150,000,000 (109,000,000 - 198,000,000)
DALYs	Neglected tropical diseases and malaria	87,200,000 (55,700,000 - 124,000,000)	62,900,000 (38,600,000 - 96,000,000)
DALYs	Neurological disorders	82,900,000 (46,500,000 - 135,000,000)	97,700,000 (55,900,000 - 159,000,000)

DALYs	Nutritional deficiencies	55,400,000<br>(43,600,000 - 71,500,000)	49,800,000<br>(36,900,000 - 65,800,000)
DALYs	Other infectious diseases	78,700,000<br>(61,800,000 - 103,000,000)	51,400,000<br>(40,700,000 - 66,000,000)
DALYs	Other non-communicable diseases	151,000,000<br>(121,000,000 - 184,000,000)	153,000,000<br>(124,000,000 - 187,000,000)
DALYs	Respiratory infections and tuberculosis	193,000,000<br>(177,000,000 - 211,000,000)	153,000,000<br>(137,000,000 - 172,000,000)
DALYs	Self-harm and interpersonal violence	69,600,000<br>(66,100,000 - 73,300,000)	67,900,000<br>(63,400,000 - 72,900,000)
DALYs	Sense organ diseases	54,800,000<br>(37,600,000 - 77,000,000)	66,100,000<br>(45,100,000 - 93,000,000)
DALYs	Skin and subcutaneous diseases	39,100,000<br>(26,100,000 - 57,800,000)	42,900,000<br>(28,600,000 - 63,400,000)
DALYs	Substance use disorders	30,900,000<br>(24,900,000 - 37,500,000)	35,100,000<br>(28,200,000 - 43,000,000)
DALYs	Transport injuries	83,600,000<br>(74,100,000 - 90,400,000)	77,600,000<br>(69,200,000 - 85,500,000)
DALYs	Unintentional injuries	120,000,000<br>(107,000,000 - 133,000,000)	104,000,000<br>(88,900,000 - 120,000,000)
**Deaths**	**Total cancers**	**8,290,000**<br>**(7,890,000 - 8,570,000)**	**10,000,000**<br>**(9,360,000 - 10,600,000)**
Deaths	Cardiovascular diseases	15,800,000<br>(14,900,000 - 16,400,000)	18,600,000<br>(17,100,000 - 19,700,000)
Deaths	Chronic respiratory diseases	3,510,000<br>(3,200,000 - 3,690,000)	3,970,000<br>(3,580,000 - 4,300,000)
Deaths	Diabetes and kidney diseases	2,290,000<br>(2,150,000 - 2,370,000)	2,990,000<br>(2,770,000 - 3,180,000)

Deaths	Digestive diseases	2,300,000<br>(2,190,000 - 2,390,000)	2,560,000<br>(2,390,000 - 2,720,000)
Deaths	Enteric infections	2,190,000<br>(1,750,000 - 2,830,000)	1,750,000<br>(1,290,000 - 2,420,000)
Deaths	HIV/AIDS and sexually transmitted infections	1,460,000<br>(1,270,000 - 1,730,000)	954,000<br>(849,000 - 1,110,000)
Deaths	Maternal and neonatal disorders	2,670,000<br>(2,440,000 - 2,910,000)	2,080,000<br>(1,780,000 - 2,450,000)
Deaths	Mental disorders	287<br>(262 - 344)	318<br>(286 - 386)
Deaths	Musculoskeletal disorders	90,700<br>(72,200 - 104,000)	118,000<br>(94,800 - 136,000)
Deaths	Neglected tropical diseases and malaria	1,030,000<br>(590,000 - 1,560,000)	747,000<br>(407,000 - 1,250,000)
Deaths	Neurological disorders	1,640,000<br>(783,000 - 3,530,000)	2,220,000<br>(1,030,000 - 4,760,000)
Deaths	Nutritional deficiencies	322,000<br>(290,000 - 358,000)	252,000<br>(221,000 - 289,000)
Deaths	Other infectious diseases	1,040,000<br>(843,000 - 1,320,000)	730,000<br>(605,000 - 905,000)
Deaths	Other non-communicable diseases	1,150,000<br>(985,000 - 1,320,000)	1,140,000<br>(1,010,000 - 1,300,000)
Deaths	Respiratory infections and tuberculosis	3,930,000<br>(3,690,000 - 4,170,000)	3,680,000<br>(3,380,000 - 4,010,000)
Deaths	Self-harm and interpersonal violence	1,260,000<br>(1,190,000 - 1,320,000)	1,250,000<br>(1,160,000 - 1,330,000)
Deaths	Sense organ diseases	NA	NA
Deaths	Skin and subcutaneous diseases	76,300	98,500

		(58,700 - 96,400)	(75,100 - 124,000)
Deaths	Substance use disorders	252,000<br>(236,000 - 259,000)	296,000<br>(273,000 - 311,000)
Deaths	Transport injuries	1,370,000<br>(1,190,000 -1,440,000)	1,280,000<br>(1,130,000 - 1,390,000)
Deaths	Unintentional injuries	1,970,000<br>(1,780,000 - 2,090,000)	1,770,000<br>(1,550,000 - 1,950,000)
**Incidence**	**Total cancers**	**18,700,000**<br>**(18,000,000 - 19,300,000)**	**23,600,000**<br>**(22,200,000 - 24,900,000)**
Incidence	Cardiovascular diseases	44,500,000<br>(42,100,000 - 47,000,000)	55,500,000<br>(52,300,000 - 58,900,000)
Incidence	Chronic respiratory diseases	64,600,000<br>(57,800,000 - 72,700,000)	77,600,000<br>(68,900,000 - 87,900,000)
Incidence	Diabetes and kidney diseases	32,000,000<br>(30,500,000 - 33,500,000)	41,900,000<br>(39,800,000 - 44,300,000)
Incidence	Digestive diseases	371,000,000<br>(339,000,000 - 405,000,000)	444,000,000<br>(406,000,000 - 484,000,000)
Incidence	Enteric infections	5,350,000,000<br>(4,960,000,000 - 5,780,000,000)	6,600,000,000<br>(6,070,000,000 - 7,160,000,000)
Incidence	HIV/AIDS and sexually transmitted infections	684,000,000<br>(586,000,000 - 794,000,000)	772,000,000<br>(661,000,000 - 895,000,000)
Incidence	Maternal and neonatal disorders	141,000,000<br>(128,000,000 - 155,000,000)	136,000,000<br>(123,000,000 - 150,000,000)
Incidence	Mental disorders	325,000,000<br>(296,000,000 - 357,000,000)	371,000,000<br>(334,000,000 - 412,000,000)
Incidence	Musculoskeletal disorders	277,000,000<br>(251,000,000 - 304,000,000)	323,000,000<br>(293,000,000 - 354,000,000)
Incidence	Neglected tropical diseases and malaria	319,000,000	290,000,000

		(274,000,000 - 378,000,000)	(231,000,000 - 365,000,000)
Incidence	Neurological disorders	720,000,000 (647,000,000 - 795,000,000)	805,000,000 (726,000,000 - 889,000,000)
Incidence	Nutritional deficiencies	146,000,000 (123,000,000 - 171,000,000)	162,000,000 (137,000,000 - 191,000,000)
Incidence	Other infectious diseases	409,000,000 (376,000,000 - 461,000,000)	384,000,000 (361,000,000 - 410,000,000)
Incidence	Other non-communicable diseases	5,160,000,000 (4,720,000,000 - 5,600,000,000)	5,710,000,000 (5,240,000,000 - 6,190,000,000)
Incidence	Respiratory infections and tuberculosis	16,400,000,000 (14,700,000,000 - 18,400,000,000)	18,100,000,000 (16,300,000,000 - 20,200,000,000)
Incidence	Self-harm and interpersonal violence	46,900,000 (39,800,000 - 54,400,000)	47,400,000 (39,500,000 - 55,600,000)
Incidence	Sense organ diseases	0 (0 - 0)	0 (0 - 0)
Incidence	Skin and subcutaneous diseases	4,260,000,000 (4,100,000,000 - 4,440,000,000)	4,860,000,000 (4,680,000,000 - 5,060,000,000)
Incidence	Substance use disorders	64,100,000 (55,600,000 - 73,400,000)	69,700,000 (60,200,000 - 80,100,000)
Incidence	Transport injuries	99,900,000 (85,600,000 - 115,000,000)	107,000,000 (90,000,000 - 125,000,000)
Incidence	Unintentional injuries	488,000,000 (455,000,000 - 524,000,000)	560,000,000 (515,000,000 - 610,000,000)
**Prevalence**	**Total cancers**	**66,700,000 (64,600,000 - 69,200,000)**	**85,800,000 (80,300,000 - 92,100,000)**
Prevalence	Cardiovascular diseases	413,000,000 (393,000,000 - 434,000,000)	523,000,000 (497,000,000 - 550,000,000)
Prevalence	Chronic respiratory diseases	386,000,000	455,000,000

		(359,000,000 - 422,000,000)	(417,000,000 - 499,000,000)
Prevalence	Diabetes and kidney diseases	775,000,000<br>(739,000,000 - 811,000,000)	994,000,000<br>(947,000,000 - 1,040,000,000)
Prevalence	Digestive diseases	1,920,000,000<br>(1,820,000,000 -2,020,000,000)	2,280,000,000<br>(2,150,000,000 - 2,400,000,000)
Prevalence	Enteric infections	80,600,000<br>(75,600,000 - 86,100,000)	98,800,000<br>(92,000,000 - 106,000,000)
Prevalence	HIV/AIDS and sexually transmitted infections	1,110,000,000<br>(997,000,000 - 1,240,000,000)	1,310,000,000<br>(1,170,000,000 - 1,460,000,000)
Prevalence	Maternal and neonatal disorders	91,500,000<br>(84,900,000 - 99,500,000)	112,000,000<br>(104,000,000 - 121,000,000)
Prevalence	Mental disorders	876,000,000<br>(813,000,000 - 943,000,000)	970,000,000<br>(901,000,000 - 1,040,000,000)
Prevalence	Musculoskeletal disorders	1,250,000,000<br>(1,180,000,000 - 1,320,000,000)	1,510,000,000<br>(1,430,000,000 - 1,600,000,000)
Prevalence	Neglected tropical diseases and malaria	1,460,000,000<br>(1,390,000,000 - 1,540,000,000)	1,310,000,000<br>(1,240,000,000 - 1,380,000,000)
Prevalence	Neurological disorders	2,360,000,000<br>(2,170,000,000 - 2,540,000,000)	2,660,000,000<br>(2,450,000,000 - 2,860,000,000)
Prevalence	Nutritional deficiencies	1,190,000,000<br>(1,150,000,000 - 1,220,000,000)	1,280,000,000<br>(1,240,000,000 - 1,320,000,000)
Prevalence	Other infectious diseases	87,100,000<br>(84,400,000 - 90,100,000)	88,600,000<br>(86,100,000 - 91,400,000)
Prevalence	Other non-communicable diseases	4,770,000,000<br>(4,620,000,000 - 4,920,000,000)	5,370,000,000<br>(5,200,000,000 - 5,540,000,000)
Prevalence	Respiratory infections and tuberculosis	2,010,000,000<br>(1,860,000,000 - 2,170,000,000)	2,110,000,000<br>(1,940,000,000 - 2,280,000,000)
Prevalence	Self-harm and interpersonal violence	351,000,000	393,000,000

		(312,000,000 - 398,000,000)	(349,000,000 - 445,000,000)
Prevalence	Sense organ diseases	1,450,000,000 (1,380,000,000 - 1,520,000,000)	1,760,000,000 (1,680,000,000 - 1,850,000,000)
Prevalence	Skin and subcutaneous diseases	1,790,000,000 (1,750,000,000 - 1,850,000,000)	2,020,000,000 (1,970,000,000 - 2,080,000,000)
Prevalence	Substance use disorders	146,000,000 (132,000,000 - 163,000,000)	161,000,000 (145,000,000 - 181,000,000)
Prevalence	Transport injuries	260,000,000 (242,000,000 - 277,000,000)	300,000,000 (280,000,000 - 321,000,000)
Prevalence	Unintentional injuries	923,000,000 (862,000,000 - 990,000,000)	1,150,000,000 (1,070,000,000 - 1,230,000,000)
**YLDs**	**Total cancers**	**6,190,000 (4,560,000 - 7,980,000)**	**7,840,000 (5,760,000 - 10,100,000)**
YLDs	Cardiovascular diseases	26,900,000 (19,600,000 - 34,300,000)	34,400,000 (24,900,000 - 43,600,000)
YLDs	Chronic respiratory diseases	27,400,000 (22,100,000 - 32,600,000)	32,400,000 (26,100,000 - 38,500,000)
YLDs	Diabetes and kidney diseases	33,400,000 (23,600,000 - 44,100,000)	45,400,000 (31,900,000 - 60,100,000)
YLDs	Digestive diseases	14,500,000 (10,100,000 - 20,200,000)	17,000,000 (11,900,000 - 23,900,000)
YLDs	Enteric infections	9,150,000 (6,330,000 - 12,500,000)	11,100,000 (7,710,000 - 15,300,000)
YLDs	HIV/AIDS and sexually transmitted infections	4,960,000 (3,490,000 - 6,960,000)	5,010,000 (3,550,000 - 6,730,000)
YLDs	Maternal and neonatal disorders	14,400,000 (11,400,000 - 17,900,000)	20,000,000 (15,900,000 - 24,200,000)
YLDs	Mental disorders	111,000,000	125,000,000

		(82,300,000 - 146,000,000)	(93,000,000 - 163,000,000)
YLDs	Musculoskeletal disorders	123,000,000<br>(88,400,000 - 163,000,000)	147,000,000<br>(106,000,000 - 195,000,000)
YLDs	Neglected tropical diseases and malaria	14,800,000<br>(10,400,000 - 20,600,000)	13,700,000<br>(9,720,000 - 19,100,000)
YLDs	Neurological disorders	56,800,000<br>(23,900,000 - 108,000,000)	65,600,000<br>(28,600,000 - 123,000,000)
YLDs	Nutritional deficiencies	36,200,000<br>(24,200,000 - 51,600,000)	37,600,000<br>(25,200,000 - 53,500,000)
YLDs	Other infectious diseases	3,600,000<br>(2,520,000 - 4,910,000)	3,650,000<br>(2,570,000 - 5,010,000)
YLDs	Other non-communicable diseases	76,000,000<br>(52,600,000 - 106,000,000)	87,500,000<br>(60,500,000 - 123,000,000)
YLDs	Respiratory infections and tuberculosis	12,600,000<br>(8,610,000 - 17,600,000)	13,300,000<br>(9,080,000 - 18,600,000)
YLDs	Self-harm and interpersonal violence	7,540,000<br>(5,680,000 - 9,780,000)	8,300,000<br>(6,310,000 - 10,700,000)
YLDs	Sense organ diseases	54,800,000<br>(37,600,000 - 77,000,000)	66,100,000<br>(45,100,000 - 93,000,000)
YLDs	Skin and subcutaneous diseases	37,100,000<br>(24,200,000 - 55,900,000)	40,600,000<br>(26,400,000 - 61,300,000)
YLDs	Substance use disorders	20,400,000<br>(14,400,000 - 27,100,000)	23,000,000<br>(16,200,000 - 30,500,000)
YLDs	Transport injuries	15,800,000<br>(11,500,000 - 20,900,000)	17,800,000<br>(13,000,000 - 23,700,000)
YLDs	Unintentional injuries	30,400,000<br>(21,500,000 - 42,200,000)	37,800,000<br>(26,700,000 - 52,200,000)
**YLLs (Years of Life Lost)**	**Total cancers**	**209,000,000**	**242,000,000**

		**(202,000,000 - 216,000,000)**	**(227,000,000 - 256,000,000)**
YLLs (Years of Life Lost)	Cardiovascular diseases	323,000,000<br>(309,000,000 - 334,000,000)	359,000,000<br>(335,000,000 - 380,000,000)
YLLs (Years of Life Lost)	Chronic respiratory diseases	66,100,000<br>(61,100,000 - 69,500,000)	71,100,000<br>(64,700,000 - 77,000,000)
YLLs (Years of Life Lost)	Diabetes and kidney diseases	54,200,000<br>(52,000,000 - 56,300,000)	67,300,000<br>(63,000,000 - 71,700,000)
YLLs (Years of Life Lost)	Digestive diseases	69,100,000<br>(66,400,000 - 72,000,000)	72,000,000<br>(67,000,000 - 77,100,000)
YLLs (Years of Life Lost)	Enteric infections	123,000,000<br>(104,000,000 - 146,000,000)	85,700,000<br>(68,000,000 - 108,000,000)
YLLs (Years of Life Lost)	HIV/AIDS and sexually transmitted infections	81,000,000<br>(68,400,000 - 98,000,000)	51,200,000<br>(43,500,000 - 62,000,000)
YLLs (Years of Life Lost)	Maternal and neonatal disorders	230,000,000<br>(210,000,000 - 251,000,000)	179,000,000<br>(153,000,000 - 211,000,000)
YLLs (Years of Life Lost)	Mental disorders	15,800<br>(14,400 - 19,200)	17,400<br>(15,500 - 21,500)
YLLs (Years of Life Lost)	Musculoskeletal disorders	2,350,000<br>(1,920,000 - 2,750,000)	2,790,000<br>(2,290,000 - 3,310,000)
YLLs (Years of Life Lost)	Neglected tropical diseases and malaria	72,400,000<br>(41,300,000 - 111,000,000)	49,200,000<br>(26,400,000 - 83,100,000)
YLLs (Years of Life Lost)	Neurological disorders	26,100,000<br>(16,300,000 - 48,100,000)	32,100,000<br>(18,800,000 - 60,800,000)
YLLs (Years of Life Lost)	Nutritional deficiencies	19,300,000<br>(16,500,000 - 22,100,000)	12,200,000<br>(10,000,000 - 15,000,000)
YLLs (Years of Life Lost)	Other infectious diseases	75,200,000<br>(58,600,000 - 99,100,000)	47,800,000<br>(37,300,000 - 62,400,000)
YLLs (Years of Life Lost)	Other non-communicable diseases	75,400,000	65,700,000

		(60,700,000 - 91,600,000)	(55,000,000 - 78,900,000)
YLLs (Years of Life Lost)	Respiratory infections and tuberculosis	181,000,000 (165,000,000 - 198,000,000)	140,000,000 (125,000,000 - 159,000,000)
YLLs (Years of Life Lost)	Self-harm and interpersonal violence	62,000,000 (58,700,000 - 65,000,000)	59,600,000 (55,400,000 - 63,700,000)
YLLs (Years of Life Lost)	Sense organ diseases	NA	NA
YLLs (Years of Life Lost)	Skin and subcutaneous diseases	1,950,000 (1,480,000 - 2,340,000)	2,260,000 (1,690,000 - 2,680,000)
YLLs (Years of Life Lost)	Substance use disorders	10,500,000 (9,850,000 - 10,800,000)	12,200,000 (11,200,000 - 12,800,000)
YLLs (Years of Life Lost)	Transport injuries	67,900,000 (59,500,000 - 71,900,000)	59,800,000 (52,900,000 - 65,300,000)
YLLs (Years of Life Lost)	Unintentional injuries	89,300,000 (80,700,000 - 95,400,000)	65,800,000 (57,400,000 - 73,300,000)

Abbreviations: 95% UI, 95% uncertainty interval; DALYs, disability-adjusted life years; GBD, Global Burden of Disease; NA, not applicable; YLDs, years lived with disability; YLLs, years of life lost.

Footnote: “Total cancers” in this table includes non-melanoma skin cancer, and excludes the “Other neoplasms” category of benign and in situ neoplasms. For “sense organ diseases”, incidence, death, and YLLs were not estimated for GBD 2019.

eTable 16: Trends in incidence globally, and by SDI quintile, both sexes, 2010 to 2019

Cause	Location	Incidence 2010, thousands (95% UI)	Incidence 2019, thousands (95% UI)	Annualized Rate of Change 2010-2019, Counts (95% UI)	Age-standardized Incidence 2010, per 100,000 (95% UI)	Age-standardized Incidence 2019, per 100,000 (95% UI)	Annualized Rate of Change 2010-2019, Age-standardized rate (95% UI)
Total cancers	Global	18 700 (18 000 to 19 300)	23 600 (22 200 to 24 900)	2.6 (2.1 to 3.1)	293.8 (283.4 to 303.4)	290.5 (274.0 to 307.1)	-0.1 (-0.7 to 0.4)
Total cancers	High SDI	8 930 (8 540 to 9 310)	10 900 (10 200 to 11 600)	2.2 (1.8 to 2.7)	598.2 (572.6 to 622.6)	605.3 (568.0 to 645.0)	0.1 (-0.4 to 0.6)
Total cancers	High-middle SDI	4 280 (4 130 to 4 440)	5 220 (4 810 to 5 620)	2.2 (1.3 to 3.1)	267.1 (257.2 to 276.9)	264.5 (243.4 to 284.4)	-0.1 (-1.0 to 0.7)
Total cancers	Middle SDI	3 550 (3 380 to 3 720)	4 810 (4 380 to 5 230)	3.4 (2.3 to 4.3)	186.6 (177.5 to 195.0)	193.3 (176.6 to 209.9)	0.4 (-0.6 to 1.3)
Total cancers	Low-middle SDI	1 370 (1 300 to 1 450)	1 920 (1 780 to 2 070)	3.7 (3.0 to 4.5)	125.0 (118.4 to 132.2)	135.6 (125.5 to 146.0)	0.9 (0.2 to 1.6)
Total cancers	Low SDI	513 (462 to 563)	700 (627 to 778)	3.5 (2.6 to 4.4)	114.0 (102.9 to 124.0)	118.2 (106.1 to 130.6)	0.4 (-0.3 to 1.1)
Total Cancers (excl. NMSC)	Global	13 800 (13 300 to 14 300)	17 200 (15 900 to 18 500)	2.4 (1.7 to 3.1)	214.8 (205.4 to 221.9)	211.4 (195.4 to 226.8)	-0.2 (-0.9 to 0.5)
Total Cancers (excl. NMSC)	High SDI	4 830 (4 580 to 5 040)	5 560 (5 020 to 6 090)	1.6 (0.7 to 2.4)	329.6 (314.5 to 343.3)	316.7 (287.4 to 346.7)	-0.4 (-1.3 to 0.4)
Total Cancers (excl. NMSC)	High-middle SDI	3 860 (3 710 to 4 010)	4 690 (4 290 to 5 090)	2.2 (1.2 to 3.1)	240.7 (230.4 to 249.8)	238.2 (217.7 to 258.3)	-0.1 (-1.1 to 0.8)
Total Cancers (excl. NMSC)	Middle SDI	3 320 (3 150 to 3 480)	4 470 (4 050 to 4 890)	3.3 (2.1 to 4.3)	173.8 (164.6 to 182.3)	179.3 (162.7 to 196.1)	0.3 (-0.8 to 1.4)
Total Cancers (excl. NMSC)	Low-middle SDI	1 300 (1 220 to 1 380)	1 810 (1 670 to 1 960)	3.7 (2.9 to 4.5)	117.3 (110.8 to 124.5)	127.2 (117.3 to 137.8)	0.9 (0.1 to 1.7)
Total Cancers (excl. NMSC)	Low SDI	496 (444 to 546)	678 (604 to 755)	3.5 (2.6 to 4.4)	109.6 (98.8 to 119.5)	114.1 (102.1 to 126.2)	0.4 (-0.3 to 1.2)
Lip and oral cavity cancer	Global	290 (280 to 302)	373 (341 to 404)	2.8 (1.8 to 3.7)	4.4 (4.2 to 4.6)	4.5 (4.1 to 4.9)	0.2 (-0.7 to 1.1)

Lip and oral cavity cancer	High SDI	71.7 (68.6 to 73.6)	80.5 (72.6 to 88.7)	1.3 (0.2 to 2.3)	5.0 (4.8 to 5.1)	4.7 (4.3 to 5.2)	-0.6 (-1.7 to 0.4)
Lip and oral cavity cancer	High-middle SDI	60.9 (58.7 to 63.0)	71.6 (64.8 to 78.1)	1.8 (0.8 to 2.8)	3.7 (3.6 to 3.8)	3.6 (3.2 to 3.9)	-0.5 (-1.5 to 0.5)
Lip and oral cavity cancer	Middle SDI	68.8 (65.0 to 74.3)	94.4 (82.9 to 106)	3.5 (2.3 to 4.7)	3.5 (3.3 to 3.8)	3.7 (3.3 to 4.2)	0.6 (-0.6 to 1.8)
Lip and oral cavity cancer	Low-middle SDI	66.9 (62.6 to 71.6)	95.6 (83.3 to 108)	4.0 (2.4 to 5.4)	6.0 (5.6 to 6.4)	6.7 (5.8 to 7.5)	1.1 (-0.4 to 2.6)
Lip and oral cavity cancer	Low SDI	21.8 (19.4 to 24.3)	30.8 (27.2 to 34.7)	3.8 (2.2 to 5.4)	5.0 (4.5 to 5.6)	5.4 (4.8 to 6.0)	0.7 (-0.9 to 2.2)
Nasopharynx cancer	Global	128 (120 to 138)	177 (156 to 200)	3.6 (2.0 to 5.1)	1.9 (1.7 to 2.0)	2.1 (1.9 to 2.4)	1.4 (-0.1 to 3.0)
Nasopharynx cancer	High SDI	17.0 (16.3 to 17.6)	19.3 (17.3 to 21.5)	1.4 (0.2 to 2.5)	1.3 (1.2 to 1.3)	1.3 (1.2 to 1.4)	-0.0 (-1.2 to 1.2)
Nasopharynx cancer	High-middle SDI	48.8 (43.9 to 54.7)	68.1 (56.5 to 81.6)	3.7 (1.3 to 6.1)	3.0 (2.7 to 3.3)	3.6 (3.0 to 4.3)	2.1 (-0.3 to 4.5)
Nasopharynx cancer	Middle SDI	44.7 (41.0 to 49.2)	66.5 (57.4 to 76.9)	4.4 (2.5 to 6.4)	2.1 (1.9 to 2.3)	2.5 (2.2 to 2.9)	2.0 (0.1 to 3.9)
Nasopharynx cancer	Low-middle SDI	12.9 (11.9 to 14.1)	16.7 (15.1 to 18.5)	2.8 (1.7 to 3.9)	1.1 (1.0 to 1.2)	1.1 (1.0 to 1.2)	0.1 (-1.0 to 1.2)
Nasopharynx cancer	Low SDI	4.62 (4.12 to 5.13)	5.90 (5.20 to 6.59)	2.7 (1.4 to 4.1)	1.0 (0.9 to 1.1)	0.9 (0.8 to 1.0)	-0.4 (-1.6 to 0.9)
Other pharynx cancer	Global	126 (122 to 131)	167 (153 to 180)	3.1 (2.2 to 3.9)	1.9 (1.8 to 2.0)	2.0 (1.8 to 2.2)	0.5 (-0.4 to 1.3)
Other pharynx cancer	High SDI	38.8 (37.7 to 39.7)	45.8 (41.1 to 51.3)	1.8 (0.7 to 3.0)	2.7 (2.7 to 2.8)	2.8 (2.5 to 3.1)	0.0 (-1.2 to 1.3)
Other pharynx cancer	High-middle SDI	25.3 (24.7 to 25.9)	32.1 (29.3 to 34.8)	2.6 (1.6 to 3.6)	1.5 (1.5 to 1.6)	1.6 (1.4 to 1.7)	0.3 (-0.7 to 1.3)
Other pharynx cancer	Middle SDI	21.6 (20.3 to 23.1)	31.4 (27.4 to 35.2)	4.1 (2.8 to 5.3)	1.1 (1.0 to 1.2)	1.2 (1.1 to 1.3)	1.1 (-0.2 to 2.3)
Other pharynx cancer	Low-middle SDI	33.7 (30.9 to 36.4)	47.7 (40.9 to 54.9)	3.9 (2.1 to 5.5)	3.1 (2.8 to 3.3)	3.3 (2.9 to 3.8)	1.0 (-0.8 to 2.6)
Other pharynx cancer	Low SDI	6.99 (6.10 to 7.84)	9.91 (8.44 to 11.6)	3.9 (2.2 to 5.6)	1.7 (1.5 to 1.9)	1.8 (1.5 to 2.1)	0.8 (-0.8 to 2.4)

Esophageal cancer	Global	479 (391 to 513)	535 (467 to 595)	1.2 (-0.2 to 2.6)	7.5 (6.1 to 8.0)	6.5 (5.7 to 7.2)	-1.6 (-2.9 to -0.2)
Esophageal cancer	High SDI	84.2 (80.1 to 87.1)	95.9 (86.7 to 105)	1.4 (0.5 to 2.4)	5.5 (5.3 to 5.7)	5.2 (4.7 to 5.7)	-0.7 (-1.7 to 0.3)
Esophageal cancer	High-middle SDI	127 (95.6 to 139)	145 (113 to 169)	1.5 (-0.3 to 3.2)	7.7 (5.8 to 8.5)	7.1 (5.5 to 8.2)	-1.0 (-2.9 to 0.6)
Esophageal cancer	Middle SDI	200 (132 to 222)	205 (165 to 238)	0.3 (-1.7 to 3.0)	11.1 (7.2 to 12.3)	8.4 (6.7 to 9.7)	-3.0 (-4.9 to -0.3)
Esophageal cancer	Low-middle SDI	46.8 (42.7 to 64.8)	59.9 (52.7 to 84.1)	2.7 (1.6 to 3.7)	4.5 (4.1 to 6.3)	4.4 (3.9 to 6.2)	-0.4 (-1.4 to 0.6)
Esophageal cancer	Low SDI	21.5 (18.5 to 24.8)	28.1 (23.2 to 33.4)	3.0 (1.8 to 4.1)	5.6 (4.8 to 6.4)	5.4 (4.5 to 6.4)	-0.3 (-1.4 to 0.7)
Stomach cancer	Global	1 160 (1 090 to 1 220)	1 270 (1 150 to 1 400)	1.0 (-0.1 to 2.1)	18.3 (17.1 to 19.2)	15.6 (14.1 to 17.2)	-1.8 (-2.8 to -0.7)
Stomach cancer	High SDI	234 (215 to 243)	238 (210 to 261)	0.2 (-0.7 to 1.1)	15.0 (14.0 to 15.6)	12.4 (11.1 to 13.6)	-2.1 (-3.0 to -1.2)
Stomach cancer	High-middle SDI	356 (332 to 378)	381 (337 to 427)	0.8 (-0.7 to 2.1)	21.9 (20.4 to 23.2)	18.8 (16.6 to 21.0)	-1.7 (-3.1 to -0.3)
Stomach cancer	Middle SDI	411 (381 to 443)	458 (399 to 527)	1.2 (-0.4 to 2.8)	22.4 (20.8 to 24.1)	18.7 (16.4 to 21.4)	-2.0 (-3.6 to -0.5)
Stomach cancer	Low-middle SDI	124 (117 to 132)	149 (136 to 163)	2.0 (1.1 to 2.9)	12.0 (11.3 to 12.7)	11.0 (10.0 to 12.0)	-1.0 (-1.9 to -0.1)
Stomach cancer	Low SDI	35.6 (32.0 to 39.2)	43.2 (38.6 to 48.1)	2.1 (1.4 to 2.9)	9.3 (8.4 to 10.2)	8.4 (7.6 to 9.3)	-1.1 (-1.8 to -0.4)
Colon and rectum cancer	Global	1 640 (1 560 to 1 700)	2 170 (2 000 to 2 340)	3.1 (2.4 to 3.8)	26.1 (24.7 to 27.1)	26.7 (24.6 to 28.9)	0.2 (-0.5 to 1.0)
Colon and rectum cancer	High SDI	678 (632 to 701)	799 (716 to 873)	1.8 (1.0 to 2.7)	43.9 (41.4 to 45.3)	42.8 (38.7 to 46.6)	-0.3 (-1.2 to 0.6)
Colon and rectum cancer	High-middle SDI	502 (480 to 522)	657 (596 to 718)	3.0 (2.0 to 4.0)	31.1 (29.6 to 32.3)	32.5 (29.5 to 35.4)	0.5 (-0.5 to 1.5)
Colon and rectum cancer	Middle SDI	338 (316 to 360)	518 (464 to 578)	4.7 (3.5 to 5.9)	18.2 (17.0 to 19.3)	20.9 (18.8 to 23.3)	1.6 (0.4 to 2.7)
Colon and rectum cancer	Low-middle SDI	95.8 (90.3 to 102)	153 (139 to 168)	5.2 (4.2 to 6.1)	9.4 (8.8 to 10.0)	11.4 (10.3 to 12.4)	2.1 (1.1 to 3.0)

Colon and rectum cancer	Low SDI	25.7 (22.9 to 29.1)	39.0 (34.9 to 43.4)	4.7 (3.6 to 5.7)	6.8 (6.1 to 7.8)	7.7 (6.9 to 8.6)	1.4 (0.3 to 2.4)
Liver cancer	Global	420 (399 to 441)	534 (487 to 589)	2.7 (1.6 to 3.7)	6.5 (6.2 to 6.8)	6.5 (5.9 to 7.2)	0.0 (-1.0 to 1.1)
Liver cancer	High SDI	121 (113 to 126)	140 (126 to 154)	1.6 (0.6 to 2.6)	8.0 (7.5 to 8.3)	7.6 (6.9 to 8.4)	-0.6 (-1.6 to 0.5)
Liver cancer	High-middle SDI	89.1 (83.2 to 95.4)	107 (94.2 to 121)	2.0 (0.5 to 3.6)	5.5 (5.1 to 5.8)	5.3 (4.7 to 6.0)	-0.2 (-1.8 to 1.3)
Liver cancer	Middle SDI	152 (142 to 165)	211 (184 to 242)	3.6 (2.0 to 5.3)	7.8 (7.3 to 8.5)	8.3 (7.2 to 9.5)	0.6 (-1.0 to 2.2)
Liver cancer	Low-middle SDI	41.4 (38.1 to 44.6)	56.3 (51.0 to 62.6)	3.4 (2.4 to 4.5)	3.9 (3.6 to 4.2)	4.1 (3.7 to 4.5)	0.5 (-0.6 to 1.5)
Liver cancer	Low SDI	16.0 (14.4 to 17.6)	20.3 (17.8 to 22.8)	2.6 (1.6 to 3.6)	3.9 (3.5 to 4.3)	3.7 (3.3 to 4.1)	-0.6 (-1.6 to 0.3)
Gallbladder and biliary tract cancer	Global	165 (141 to 174)	199 (167 to 220)	2.1 (1.3 to 2.9)	2.7 (2.3 to 2.8)	2.5 (2.1 to 2.7)	-0.8 (-1.7 to -0.0)
Gallbladder and biliary tract cancer	High SDI	56.4 (45.5 to 60.5)	63.8 (52.1 to 72.3)	1.4 (0.5 to 2.2)	3.5 (2.8 to 3.7)	3.2 (2.6 to 3.6)	-1.1 (-1.9 to -0.2)
Gallbladder and biliary tract cancer	High-middle SDI	44.6 (33.8 to 47.8)	50.8 (37.7 to 57.5)	1.4 (0.3 to 2.6)	2.8 (2.1 to 3.0)	2.5 (1.9 to 2.8)	-1.2 (-2.4 to -0.1)
Gallbladder and biliary tract cancer	Middle SDI	35.8 (32.1 to 39.7)	45.8 (39.6 to 53.4)	2.7 (1.5 to 4.0)	2.0 (1.8 to 2.3)	1.9 (1.7 to 2.2)	-0.6 (-1.9 to 0.7)
Gallbladder and biliary tract cancer	Low-middle SDI	21.4 (19.0 to 24.9)	29.6 (26.0 to 33.9)	3.6 (2.4 to 4.8)	2.1 (1.9 to 2.5)	2.2 (2.0 to 2.6)	0.5 (-0.7 to 1.6)
Gallbladder and biliary tract cancer	Low SDI	6.75 (5.50 to 7.90)	9.11 (7.68 to 10.5)	3.3 (2.2 to 4.8)	1.8 (1.5 to 2.1)	1.9 (1.6 to 2.1)	0.2 (-0.9 to 1.6)
Pancreatic cancer	Global	391 (369 to 406)	530 (486 to 574)	3.4 (2.7 to 4.1)	6.3 (5.9 to 6.5)	6.6 (6.0 to 7.1)	0.5 (-0.3 to 1.2)
Pancreatic cancer	High SDI	159 (147 to 165)	197 (175 to 216)	2.4 (1.5 to 3.2)	10.1 (9.4 to 10.4)	10.2 (9.1 to 11.1)	0.1 (-0.8 to 1.0)
Pancreatic cancer	High-middle SDI	121 (115 to 126)	157 (143 to 170)	2.9 (1.9 to 3.8)	7.5 (7.1 to 7.8)	7.7 (7.0 to 8.3)	0.3 (-0.7 to 1.2)
Pancreatic cancer	Middle SDI	75.3 (70.4 to 80.8)	117 (105 to 131)	4.9 (3.9 to 5.9)	4.2 (3.9 to 4.4)	4.8 (4.3 to 5.3)	1.6 (0.6 to 2.6)

Pancreatic cancer	Low-middle SDI	27.9<br>(26.0 to 29.8)	46.9<br>(43.0 to 51.0)	5.8<br>(4.7 to 6.8)	2.8<br>(2.6 to 3.0)	3.5<br>(3.2 to 3.8)	2.6<br>(1.6 to 3.6)
Pancreatic cancer	Low SDI	7.80<br>(6.61 to 8.97)	12.6<br>(11.0 to 14.2)	5.3<br>(4.1 to 6.5)	2.1<br>(1.8 to 2.4)	2.5<br>(2.2 to 2.9)	2.0<br>(0.9 to 3.2)
Larynx cancer	Global	168<br>(162 to 173)	209<br>(194 to 225)	2.5<br>(1.6 to 3.3)	2.6<br>(2.5 to 2.7)	2.5<br>(2.3 to 2.7)	-0.3<br>(-1.1 to 0.5)
Larynx cancer	High SDI	38.6<br>(37.2 to 39.5)	43.7<br>(39.3 to 48.5)	1.4<br>(0.2 to 2.5)	2.6<br>(2.5 to 2.7)	2.5<br>(2.2 to 2.8)	-0.6<br>(-1.8 to 0.5)
Larynx cancer	High-middle SDI	50.2<br>(48.5 to 51.7)	57.9<br>(52.6 to 63.5)	1.6<br>(0.5 to 2.6)	3.0<br>(2.9 to 3.1)	2.8<br>(2.5 to 3.1)	-0.9<br>(-1.9 to 0.1)
Larynx cancer	Middle SDI	40.4<br>(38.2 to 42.9)	57.1<br>(51.5 to 63.5)	3.9<br>(2.6 to 5.1)	2.1<br>(2.0 to 2.2)	2.2<br>(2.0 to 2.5)	0.6<br>(-0.6 to 1.8)
Larynx cancer	Low-middle SDI	28.7<br>(26.5 to 31.1)	38.0<br>(33.9 to 42.5)	3.1<br>(1.8 to 4.4)	2.7<br>(2.5 to 2.9)	2.7<br>(2.4 to 3.0)	0.1<br>(-1.2 to 1.4)
Larynx cancer	Low SDI	9.74<br>(8.58 to 11.0)	12.3<br>(10.7 to 14.1)	2.6<br>(1.1 to 4.2)	2.4<br>(2.1 to 2.7)	2.3<br>(2.0 to 2.6)	-0.6<br>(-2.0 to 0.9)
Tracheal, bronchus, and lung cancer	Global	1 830<br>(1 750 to 1 910)	2 260<br>(2 070 to 2 450)	2.3<br>(1.3 to 3.2)	29.0<br>(27.6 to 30.2)	27.7<br>(25.3 to 30.0)	-0.5<br>(-1.5 to 0.4)
Tracheal, bronchus, and lung cancer	High SDI	634<br>(599 to 653)	709<br>(637 to 773)	1.2<br>(0.4 to 2.2)	40.9<br>(38.9 to 42.0)	37.4<br>(33.9 to 40.8)	-1.0<br>(-1.9 to -0.1)
Tracheal, bronchus, and lung cancer	High-middle SDI	561<br>(533 to 587)	672<br>(604 to 736)	2.0<br>(0.8 to 3.2)	34.3<br>(32.6 to 35.9)	32.7<br>(29.3 to 35.8)	-0.6<br>(-1.8 to 0.6)
Tracheal, bronchus, and lung cancer	Middle SDI	482<br>(448 to 516)	662<br>(579 to 750)	3.5<br>(1.9 to 5.0)	26.5<br>(24.6 to 28.4)	27.0<br>(23.7 to 30.6)	0.2<br>(-1.3 to 1.6)
Tracheal, bronchus, and lung cancer	Low-middle SDI	124<br>(115 to 133)	171<br>(154 to 188)	3.6<br>(2.4 to 4.7)	12.1<br>(11.3 to 13.0)	12.7<br>(11.4 to 13.9)	0.5<br>(-0.7 to 1.5)
Tracheal, bronchus, and lung cancer	Low SDI	32.2<br>(27.4 to 38.2)	44.6<br>(38.3 to 52.4)	3.6<br>(2.3 to 4.8)	8.5<br>(7.3 to 10.1)	8.8<br>(7.6 to 10.3)	0.4<br>(-0.9 to 1.5)
Malignant skin melanoma	Global	241<br>(172 to 274)	290<br>(214 to 342)	2.0<br>(1.0 to 3.1)	3.7<br>(2.6 to 4.2)	3.6<br>(2.6 to 4.2)	-0.4<br>(-1.5 to 0.7)
Malignant skin melanoma	High SDI	168<br>(116 to 194)	194<br>(140 to 238)	1.6<br>(0.4 to 3.0)	12.4<br>(8.8 to 14.8)	12.4<br>(9.2 to 15.5)	-0.0<br>(-1.3 to 1.5)
Malignant skin melanoma	High-middle SDI	54.7<br>(38.6 to 59.9)	67.6<br>(47.6 to 78.7)	2.4<br>(1.3 to 3.5)	3.4<br>(2.4 to 3.7)	3.5<br>(2.5 to 4.1)	0.4<br>(-0.6 to 1.6)

Malignant skin melanoma	Middle SDI	12.3 (9.82 to 14.4)	18.1 (14.3 to 21.7)	4.3 (3.0 to 5.5)	0.6 (0.5 to 0.7)	0.7 (0.6 to 0.9)	1.7 (0.5 to 2.8)
Malignant skin melanoma	Low-middle SDI	4.28 (3.32 to 5.08)	6.17 (4.82 to 7.30)	4.1 (2.9 to 5.1)	0.4 (0.3 to 0.5)	0.4 (0.3 to 0.5)	1.2 (0.2 to 2.2)
Malignant skin melanoma	Low SDI	2.43 (1.79 to 3.17)	3.42 (2.59 to 4.39)	3.8 (2.1 to 5.5)	0.5 (0.4 to 0.6)	0.5 (0.4 to 0.7)	0.5 (-0.9 to 1.9)
Non-melanoma skin cancer	Global	4 850 (4 440 to 5 250)	6 350 (5 810 to 6 950)	3.0 (2.8 to 3.2)	79.0 (72.6 to 85.8)	79.1 (72.3 to 86.6)	0.0 (-0.2 to 0.2)
Non-melanoma skin cancer	High SDI	4 100 (3 770 to 4 430)	5 340 (4 890 to 5 830)	2.9 (2.7 to 3.2)	268.6 (247.3 to 289.9)	288.7 (264.7 to 313.7)	0.8 (0.6 to 1.0)
Non-melanoma skin cancer	High-middle SDI	421 (375 to 471)	530 (459 to 607)	2.6 (2.2 to 2.9)	26.4 (23.5 to 29.5)	26.3 (22.9 to 30.0)	-0.0 (-0.4 to 0.3)
Non-melanoma skin cancer	Middle SDI	231 (201 to 262)	343 (291 to 397)	4.4 (4.0 to 4.7)	12.8 (11.2 to 14.4)	14.0 (12.0 to 16.1)	1.0 (0.7 to 1.3)
Non-melanoma skin cancer	Low-middle SDI	74.8 (64.3 to 86.0)	113 (93.9 to 133)	4.6 (4.2 to 4.9)	7.7 (6.6 to 8.7)	8.4 (7.0 to 9.8)	1.0 (0.5 to 1.4)
Non-melanoma skin cancer	Low SDI	17.2 (14.0 to 21.1)	22.6 (18.4 to 27.9)	3.1 (2.9 to 3.3)	4.4 (3.6 to 5.2)	4.2 (3.4 to 5.0)	-0.6 (-0.8 to -0.3)
Breast cancer	Global	1 580 (1 520 to 1 640)	2 000 (1 830 to 2 170)	2.6 (1.7 to 3.5)	23.8 (22.8 to 24.6)	24.2 (22.1 to 26.2)	0.2 (-0.7 to 1.0)
Breast cancer	High SDI	622 (593 to 641)	679 (607 to 754)	1.0 (-0.2 to 2.1)	43.9 (42.2 to 45.1)	41.2 (36.9 to 45.6)	-0.7 (-1.9 to 0.4)
Breast cancer	High-middle SDI	425 (407 to 441)	517 (464 to 574)	2.2 (1.1 to 3.3)	25.7 (24.6 to 26.7)	26.0 (23.3 to 28.9)	0.1 (-1.0 to 1.3)
Breast cancer	Middle SDI	337 (319 to 357)	493 (437 to 553)	4.2 (2.9 to 5.5)	16.0 (15.1 to 16.8)	18.5 (16.4 to 20.8)	1.7 (0.4 to 2.9)
Breast cancer	Low-middle SDI	147 (136 to 158)	231 (203 to 259)	5.0 (3.7 to 6.3)	12.6 (11.7 to 13.5)	15.4 (13.5 to 17.3)	2.2 (0.9 to 3.5)
Breast cancer	Low SDI	51.5 (45.8 to 57.7)	81.7 (71.3 to 93.3)	5.1 (3.6 to 6.5)	11.5 (10.1 to 12.8)	13.5 (11.9 to 15.2)	1.8 (0.5 to 3.1)
Cervical cancer	Global	477 (423 to 521)	566 (482 to 636)	1.9 (0.9 to 2.9)	6.9 (6.1 to 7.5)	6.8 (5.8 to 7.7)	-0.1 (-1.1 to 0.8)
Cervical cancer	High SDI	60.8 (56.4 to 63.2)	63.9 (55.7 to 71.5)	0.5 (-0.6 to 1.7)	4.8 (4.4 to 5.0)	4.5 (3.9 to 5.0)	-0.7 (-1.9 to 0.5)

Cervical cancer	High-middle SDI	103 (89.2 to 110)	113 (89.8 to 129)	1.0 (-0.4 to 2.4)	6.3 (5.4 to 6.6)	5.9 (4.7 to 6.8)	-0.6 (-2.1 to 0.8)
Cervical cancer	Middle SDI	154 (128 to 166)	183 (144 to 209)	1.9 (0.6 to 3.2)	7.1 (5.9 to 7.7)	6.9 (5.4 to 7.8)	-0.4 (-1.7 to 0.8)
Cervical cancer	Low-middle SDI	97.4 (86.7 to 116)	126 (108 to 150)	2.9 (1.6 to 4.2)	7.8 (7.0 to 9.5)	8.0 (6.9 to 9.6)	0.3 (-0.9 to 1.6)
Cervical cancer	Low SDI	60.3 (47.6 to 72.0)	78.8 (61.6 to 97.9)	3.0 (1.7 to 4.4)	12.0 (9.5 to 14.4)	11.7 (9.3 to 14.5)	-0.2 (-1.4 to 1.1)
Uterine cancer	Global	359 (317 to 375)	435 (397 to 480)	2.1 (1.1 to 3.4)	5.4 (4.8 to 5.7)	5.2 (4.8 to 5.7)	-0.5 (-1.4 to 0.7)
Uterine cancer	High SDI	134 (128 to 138)	168 (149 to 189)	2.5 (1.2 to 3.7)	9.3 (8.9 to 9.6)	9.9 (8.8 to 11.1)	0.7 (-0.6 to 1.9)
Uterine cancer	High-middle SDI	129 (115 to 136)	149 (133 to 165)	1.6 (0.4 to 3.0)	7.8 (6.9 to 8.2)	7.3 (6.5 to 8.1)	-0.7 (-1.8 to 0.7)
Uterine cancer	Middle SDI	69.1 (46.5 to 77.6)	78.6 (64.7 to 92.0)	1.4 (-0.5 to 4.8)	3.3 (2.3 to 3.7)	2.9 (2.4 to 3.4)	-1.4 (-3.2 to 1.8)
Uterine cancer	Low-middle SDI	19.7 (16.7 to 22.9)	29.4 (25.0 to 35.6)	4.4 (3.3 to 5.6)	1.8 (1.5 to 2.1)	2.0 (1.7 to 2.5)	1.5 (0.4 to 2.6)
Uterine cancer	Low SDI	6.23 (5.13 to 7.65)	9.53 (7.77 to 11.7)	4.7 (3.4 to 6.2)	1.5 (1.3 to 1.9)	1.7 (1.4 to 2.1)	1.4 (0.2 to 2.7)
Ovarian cancer	Global	230 (214 to 248)	294 (261 to 330)	2.7 (1.7 to 3.7)	3.5 (3.2 to 3.7)	3.6 (3.2 to 4.0)	0.3 (-0.8 to 1.3)
Ovarian cancer	High SDI	74.2 (69.3 to 77.4)	80.5 (70.5 to 91.5)	0.9 (-0.2 to 2.1)	5.2 (4.9 to 5.4)	4.8 (4.3 to 5.5)	-0.9 (-2.0 to 0.3)
Ovarian cancer	High-middle SDI	66.4 (61.9 to 70.0)	77.3 (65.9 to 86.5)	1.7 (0.5 to 2.8)	4.1 (3.8 to 4.3)	4.0 (3.4 to 4.4)	-0.3 (-1.5 to 0.8)
Ovarian cancer	Middle SDI	52.6 (47.0 to 60.5)	76.5 (63.2 to 89.0)	4.2 (2.5 to 5.5)	2.5 (2.3 to 2.9)	2.9 (2.4 to 3.4)	1.7 (0.0 to 3.0)
Ovarian cancer	Low-middle SDI	27.0 (23.3 to 33.0)	43.6 (35.3 to 54.7)	5.3 (3.2 to 7.0)	2.3 (2.0 to 2.8)	2.9 (2.4 to 3.6)	2.6 (0.5 to 4.2)
Ovarian cancer	Low SDI	9.65 (7.75 to 14.3)	16.4 (13.5 to 20.3)	5.9 (3.1 to 8.3)	2.1 (1.6 to 3.0)	2.6 (2.2 to 3.2)	2.6 (0.0 to 4.9)
Prostate cancer	Global	1 070 (927 to 1 360)	1 410 (1 230 to 1 830)	3.1 (2.3 to 4.0)	17.3 (14.9 to 22.0)	17.4 (15.1 to 22.5)	0.1 (-0.8 to 1.0)

Prostate cancer	High SDI	568<br>(477 to 766)	696<br>(588 to 994)	2.3<br>(1.0 to 3.5)	36.7<br>(31.0 to 49.5)	36.6<br>(30.8 to 52.4)	-0.0<br>(-1.3 to 1.3)
Prostate cancer	High-middle SDI	240<br>(209 to 304)	318<br>(275 to 404)	3.2<br>(2.2 to 4.1)	14.9<br>(13.0 to 18.8)	15.4<br>(13.3 to 19.5)	0.4<br>(-0.6 to 1.3)
Prostate cancer	Middle SDI	160<br>(138 to 184)	249<br>(215 to 298)	4.9<br>(3.9 to 5.9)	9.7<br>(8.3 to 11.2)	10.8<br>(9.3 to 12.8)	1.2<br>(0.2 to 2.1)
Prostate cancer	Low-middle SDI	68.9<br>(58.2 to 78.6)	105<br>(89.7 to 122)	4.6<br>(3.6 to 5.6)	7.9<br>(6.7 to 9.0)	8.5<br>(7.3 to 10.0)	0.9<br>(-0.1 to 1.9)
Prostate cancer	Low SDI	28.3<br>(20.8 to 33.3)	40.6<br>(30.4 to 47.9)	4.0<br>(3.1 to 4.9)	9.2<br>(6.7 to 10.8)	9.6<br>(7.2 to 11.3)	0.5<br>(-0.4 to 1.3)
Testicular cancer	Global	88.4<br>(75.0 to 101)	109<br>(93.4 to 129)	2.4<br>(1.2 to 3.6)	1.2<br>(1.0 to 1.4)	1.4<br>(1.2 to 1.7)	1.3<br>(0.1 to 2.5)
Testicular cancer	High SDI	36.2<br>(29.1 to 43.4)	36.6<br>(28.5 to 45.9)	0.1<br>(-1.5 to 1.8)	4.1<br>(3.0 to 5.3)	4.0<br>(2.8 to 5.4)	-0.2<br>(-2.0 to 1.4)
Testicular cancer	High-middle SDI	29.3<br>(24.4 to 34.7)	37.8<br>(30.9 to 46.3)	2.8<br>(1.2 to 4.5)	2.2<br>(1.7 to 2.7)	2.7<br>(2.1 to 3.5)	2.4<br>(0.5 to 4.3)
Testicular cancer	Middle SDI	15.3<br>(13.1 to 17.5)	23.6<br>(20.6 to 27.5)	4.9<br>(3.3 to 6.5)	0.7<br>(0.6 to 0.8)	1.0<br>(0.9 to 1.2)	3.9<br>(2.4 to 5.6)
Testicular cancer	Low-middle SDI	5.28<br>(4.32 to 6.93)	7.82<br>(6.57 to 9.83)	4.4<br>(2.4 to 6.4)	0.3<br>(0.3 to 0.4)	0.4<br>(0.4 to 0.6)	3.1<br>(1.3 to 5.0)
Testicular cancer	Low SDI	2.33<br>(1.60 to 4.45)	3.41<br>(2.47 to 5.76)	4.2<br>(0.9 to 7.5)	0.2<br>(0.2 to 0.4)	0.3<br>(0.2 to 0.4)	2.0<br>(-0.6 to 4.8)
Kidney cancer	Global	295<br>(285 to 303)	372<br>(345 to 402)	2.6<br>(1.8 to 3.4)	4.6<br>(4.4 to 4.7)	4.6<br>(4.2 to 4.9)	0.0<br>(-0.8 to 0.8)
Kidney cancer	High SDI	132<br>(126 to 135)	155<br>(141 to 171)	1.8<br>(0.8 to 2.9)	9.1<br>(8.8 to 9.3)	9.0<br>(8.2 to 10.0)	-0.1<br>(-1.1 to 1.0)
Kidney cancer	High-middle SDI	96.8<br>(93.4 to 99.8)	119<br>(110 to 128)	2.3<br>(1.4 to 3.1)	6.1<br>(5.8 to 6.3)	6.1<br>(5.6 to 6.6)	0.1<br>(-0.8 to 0.9)
Kidney cancer	Middle SDI	45.6<br>(43.0 to 49.1)	66.5<br>(59.2 to 75.2)	4.2<br>(3.0 to 5.3)	2.3<br>(2.2 to 2.5)	2.6<br>(2.4 to 3.0)	1.5<br>(0.4 to 2.6)
Kidney cancer	Low-middle SDI	14.9<br>(13.9 to 16.1)	22.6<br>(20.5 to 25.1)	4.6<br>(3.5 to 5.8)	1.3<br>(1.2 to 1.4)	1.5<br>(1.4 to 1.7)	2.1<br>(1.0 to 3.3)
Kidney cancer	Low SDI	5.73<br>(4.84 to 6.67)	8.37<br>(7.27 to 9.54)	4.2<br>(2.8 to 5.7)	1.1<br>(0.9 to 1.2)	1.2<br>(1.0 to 1.4)	1.7<br>(0.5 to 3.0)

Bladder cancer	Global	405 (386 to 419)	524 (476 to 569)	2.9 (2.0 to 3.7)	6.6 (6.2 to 6.8)	6.5 (5.9 to 7.1)	-0.1 (-0.9 to 0.8)
Bladder cancer	High SDI	156 (147 to 162)	192 (170 to 214)	2.3 (1.2 to 3.3)	9.9 (9.4 to 10.2)	9.9 (8.9 to 11.0)	-0.0 (-1.1 to 1.1)
Bladder cancer	High-middle SDI	145 (139 to 150)	178 (162 to 195)	2.3 (1.2 to 3.3)	9.0 (8.6 to 9.4)	8.8 (8.0 to 9.6)	-0.4 (-1.4 to 0.6)
Bladder cancer	Middle SDI	69.0 (64.5 to 73.2)	104 (91.9 to 119)	4.6 (3.2 to 5.9)	3.8 (3.6 to 4.1)	4.3 (3.8 to 4.9)	1.2 (-0.0 to 2.5)
Bladder cancer	Low-middle SDI	23.7 (21.8 to 25.8)	34.9 (32.0 to 38.0)	4.3 (3.2 to 5.5)	2.4 (2.2 to 2.7)	2.7 (2.5 to 2.9)	1.0 (-0.1 to 2.2)
Bladder cancer	Low SDI	11.1 (9.32 to 12.7)	15.5 (13.5 to 17.7)	3.7 (2.1 to 5.1)	3.2 (2.7 to 3.6)	3.3 (2.8 to 3.7)	0.2 (-1.3 to 1.7)
Brain and central nervous system cancer	Global	284 (226 to 307)	348 (262 to 389)	2.2 (1.0 to 3.2)	4.3 (3.4 to 4.6)	4.3 (3.3 to 4.9)	0.2 (-1.0 to 1.1)
Brain and central nervous system cancer	High SDI	77.5 (56.6 to 82.9)	91.7 (64.0 to 106)	1.9 (0.8 to 3.0)	6.3 (4.7 to 6.7)	6.5 (4.6 to 7.4)	0.3 (-0.9 to 1.4)
Brain and central nervous system cancer	High-middle SDI	81.5 (63.2 to 87.7)	97.8 (70.8 to 111)	2.0 (0.7 to 3.0)	5.4 (4.2 to 5.8)	5.6 (4.1 to 6.4)	0.4 (-1.0 to 1.4)
Brain and central nervous system cancer	Middle SDI	77.3 (60.6 to 84.0)	98.9 (73.3 to 113)	2.7 (1.2 to 3.9)	3.8 (2.9 to 4.1)	4.0 (3.0 to 4.6)	0.7 (-0.8 to 1.8)
Brain and central nervous system cancer	Low-middle SDI	33.8 (27.3 to 40.9)	41.6 (32.9 to 48.8)	2.3 (0.6 to 3.6)	2.5 (2.0 to 3.1)	2.6 (2.1 to 3.1)	0.5 (-1.0 to 1.6)
Brain and central nervous system cancer	Low SDI	14.0 (10.2 to 19.5)	17.8 (13.1 to 22.0)	2.6 (0.4 to 4.6)	1.9 (1.4 to 2.5)	2.0 (1.5 to 2.4)	0.4 (-1.4 to 1.9)
Thyroid cancer	Global	191 (171 to 198)	234 (212 to 253)	2.2 (1.5 to 3.1)	2.8 (2.5 to 2.9)	2.8 (2.6 to 3.1)	0.1 (-0.6 to 1.0)
Thyroid cancer	High SDI	67.2 (59.0 to 69.8)	68.4 (62.1 to 74.9)	0.2 (-0.8 to 1.5)	5.2 (4.5 to 5.4)	4.6 (4.2 to 5.0)	-1.3 (-2.4 to 0.1)
Thyroid cancer	High-middle SDI	49.8 (46.7 to 51.9)	58.0 (52.4 to 64.0)	1.7 (0.7 to 2.7)	3.0 (2.9 to 3.2)	3.1 (2.8 to 3.4)	0.0 (-1.0 to 1.1)
Thyroid cancer	Middle SDI	46.0 (41.6 to 48.9)	65.6 (57.6 to 73.1)	3.9 (2.9 to 4.9)	2.1 (1.9 to 2.3)	2.5 (2.2 to 2.8)	1.7 (0.7 to 2.7)
Thyroid cancer	Low-middle SDI	20.4 (17.7 to 22.3)	30.1 (26.0 to 33.7)	4.3 (3.3 to 5.4)	1.6 (1.4 to 1.8)	1.9 (1.7 to 2.1)	1.9 (0.8 to 2.9)

Thyroid cancer	Low SDI	7.79<br>(6.25 to 8.91)	11.7<br>(9.59 to 13.7)	4.5<br>(3.0 to 6.1)	1.4<br>(1.2 to 1.7)	1.6<br>(1.3 to 1.9)	1.3<br>(0.1 to 2.6)
Mesothelioma	Global	29.3<br>(26.9 to 30.9)	34.5<br>(31.2 to 37.8)	1.8<br>(1.1 to 2.6)	0.5<br>(0.4 to 0.5)	0.4<br>(0.4 to 0.5)	-0.9<br>(-1.7 to -0.1)
Mesothelioma	High SDI	15.1<br>(14.1 to 15.7)	17.5<br>(15.6 to 19.6)	1.7<br>(0.6 to 2.8)	1.0<br>(0.9 to 1.0)	0.9<br>(0.8 to 1.0)	-0.7<br>(-1.8 to 0.5)
Mesothelioma	High-middle SDI	6.64<br>(5.93 to 7.06)	7.24<br>(6.39 to 8.01)	1.0<br>(-0.1 to 2.0)	0.4<br>(0.4 to 0.4)	0.4<br>(0.3 to 0.4)	-1.4<br>(-2.5 to -0.3)
Mesothelioma	Middle SDI	4.13<br>(3.71 to 4.49)	4.97<br>(4.45 to 5.47)	2.1<br>(1.1 to 3.0)	0.2<br>(0.2 to 0.2)	0.2<br>(0.2 to 0.2)	-0.8<br>(-1.7 to 0.1)
Mesothelioma	Low-middle SDI	2.51<br>(2.00 to 3.19)	3.52<br>(2.87 to 4.37)	3.8<br>(2.5 to 5.1)	0.2<br>(0.2 to 0.3)	0.3<br>(0.2 to 0.3)	0.8<br>(-0.4 to 2.1)
Mesothelioma	Low SDI	0.862<br>(0.490 to 1.61)	1.20<br>(0.703 to 2.23)	3.7<br>(2.1 to 5.3)	0.2<br>(0.1 to 0.4)	0.2<br>(0.1 to 0.4)	0.6<br>(-0.9 to 2.0)
Hodgkin lymphoma	Global	77.3<br>(69.1 to 89.1)	87.5<br>(77.9 to 101)	1.4<br>(0.7 to 2.0)	1.1<br>(1.0 to 1.3)	1.1<br>(1.0 to 1.3)	-0.3<br>(-1.0 to 0.3)
Hodgkin lymphoma	High SDI	30.5<br>(27.0 to 36.2)	33.0<br>(28.6 to 39.9)	0.9<br>(-0.0 to 1.8)	2.6<br>(2.3 to 3.2)	2.5<br>(2.2 to 3.2)	-0.5<br>(-1.4 to 0.5)
Hodgkin lymphoma	High-middle SDI	20.0<br>(17.4 to 22.7)	22.0<br>(19.1 to 25.4)	1.0<br>(0.1 to 1.9)	1.3<br>(1.1 to 1.5)	1.4<br>(1.2 to 1.6)	0.3<br>(-0.6 to 1.2)
Hodgkin lymphoma	Middle SDI	12.7<br>(10.8 to 14.9)	15.8<br>(13.3 to 18.5)	2.4<br>(1.6 to 3.2)	0.6<br>(0.5 to 0.7)	0.6<br>(0.5 to 0.7)	0.9<br>(0.1 to 1.7)
Hodgkin lymphoma	Low-middle SDI	8.58<br>(7.49 to 11.1)	10.0<br>(8.61 to 12.8)	1.7<br>(0.7 to 2.8)	0.6<br>(0.5 to 0.8)	0.6<br>(0.5 to 0.8)	-0.2<br>(-1.2 to 0.7)
Hodgkin lymphoma	Low SDI	5.36<br>(4.02 to 6.24)	6.63<br>(5.06 to 7.84)	2.4<br>(1.2 to 3.5)	0.8<br>(0.6 to 0.9)	0.7<br>(0.6 to 0.9)	-0.7<br>(-1.8 to 0.4)
Non-Hodgkin lymphoma	Global	355<br>(331 to 377)	457<br>(417 to 499)	2.8<br>(2.1 to 3.5)	5.6<br>(5.2 to 6.0)	5.7<br>(5.2 to 6.3)	0.2<br>(-0.5 to 0.9)
Non-Hodgkin lymphoma	High SDI	152<br>(138 to 165)	184<br>(162 to 207)	2.1<br>(1.2 to 3.0)	10.1<br>(9.2 to 10.8)	9.9<br>(8.8 to 11.1)	-0.2<br>(-1.0 to 0.7)
Non-Hodgkin lymphoma	High-middle SDI	88.3<br>(82.8 to 94.2)	110<br>(98.4 to 123)	2.4<br>(1.5 to 3.4)	5.7<br>(5.3 to 6.1)	5.9<br>(5.3 to 6.5)	0.3<br>(-0.6 to 1.3)
Non-Hodgkin lymphoma	Middle SDI	70.9<br>(65.8 to 76.6)	102<br>(91.2 to 114)	4.0<br>(2.9 to 5.1)	3.6<br>(3.3 to 3.9)	4.1<br>(3.7 to 4.6)	1.6<br>(0.6 to 2.6)

Non-Hodgkin lymphoma	Low-middle SDI	31.8 (28.4 to 36.4)	45.4 (40.3 to 51.6)	3.9 (3.0 to 4.9)	2.6 (2.4 to 2.9)	3.0 (2.7 to 3.4)	1.6 (0.6 to 2.5)
Non-Hodgkin lymphoma	Low SDI	11.2 (9.52 to 13.3)	15.6 (12.9 to 18.8)	3.7 (2.4 to 4.9)	2.2 (1.9 to 2.6)	2.4 (2.0 to 2.8)	0.6 (-0.4 to 1.6)
Multiple myeloma	Global	121 (108 to 129)	156 (137 to 173)	2.8 (2.0 to 3.6)	1.9 (1.7 to 2.1)	1.9 (1.7 to 2.1)	-0.1 (-0.9 to 0.7)
Multiple myeloma	High SDI	59.3 (52.7 to 63.0)	72.2 (62.6 to 82.5)	2.2 (1.2 to 3.2)	3.8 (3.4 to 4.1)	3.8 (3.3 to 4.3)	-0.1 (-1.0 to 1.0)
Multiple myeloma	High-middle SDI	29.5 (25.6 to 31.1)	37.4 (31.0 to 41.8)	2.6 (1.5 to 3.6)	1.8 (1.6 to 1.9)	1.8 (1.5 to 2.1)	0.0 (-1.1 to 1.0)
Multiple myeloma	Middle SDI	18.5 (16.1 to 20.8)	26.7 (22.0 to 30.4)	4.1 (2.6 to 5.2)	1.0 (0.9 to 1.1)	1.1 (0.9 to 1.2)	0.8 (-0.7 to 1.9)
Multiple myeloma	Low-middle SDI	9.58 (8.38 to 11.2)	13.8 (11.9 to 15.9)	4.0 (2.8 to 5.0)	0.9 (0.8 to 1.1)	1.0 (0.9 to 1.2)	0.9 (-0.3 to 1.9)
Multiple myeloma	Low SDI	3.90 (3.10 to 4.74)	5.48 (4.32 to 6.39)	3.8 (2.5 to 4.9)	1.0 (0.8 to 1.2)	1.1 (0.8 to 1.2)	0.5 (-0.7 to 1.6)
Leukemia	Global	562 (522 to 601)	644 (587 to 700)	1.5 (0.6 to 2.4)	8.6 (8.0 to 9.2)	8.2 (7.5 to 8.9)	-0.5 (-1.4 to 0.4)
Leukemia	High SDI	165 (155 to 174)	189 (169 to 208)	1.5 (0.5 to 2.4)	12.4 (11.8 to 13.1)	12.0 (10.9 to 13.2)	-0.4 (-1.4 to 0.6)
Leukemia	High-middle SDI	141 (131 to 147)	168 (151 to 183)	2.0 (0.9 to 2.9)	10.0 (9.3 to 10.5)	10.1 (9.1 to 11.1)	0.1 (-0.9 to 1.1)
Leukemia	Middle SDI	143 (131 to 156)	164 (144 to 184)	1.5 (0.3 to 2.6)	7.1 (6.5 to 7.7)	6.9 (6.1 to 7.7)	-0.3 (-1.5 to 0.8)
Leukemia	Low-middle SDI	68.7 (57.3 to 82.2)	74.2 (65.0 to 86.0)	0.9 (-0.6 to 2.3)	5.0 (4.3 to 5.8)	4.7 (4.2 to 5.5)	-0.6 (-1.9 to 0.7)
Leukemia	Low SDI	44.8 (32.7 to 61.0)	49.0 (37.9 to 60.8)	1.0 (-1.3 to 3.4)	5.4 (4.2 to 6.7)	5.1 (4.1 to 6.0)	-0.6 (-2.2 to 0.9)
Acute lymphoid leukemia	Global	114 (101 to 125)	153 (129 to 171)	3.3 (2.1 to 4.4)	1.7 (1.5 to 1.8)	2.0 (1.6 to 2.2)	1.8 (0.5 to 2.9)
Acute lymphoid leukemia	High SDI	34.9 (32.5 to 39.9)	40.1 (34.9 to 45.8)	1.6 (0.2 to 2.8)	3.4 (3.2 to 3.8)	3.5 (3.1 to 4.0)	0.5 (-0.8 to 1.8)
Acute lymphoid leukemia	High-middle SDI	33.7 (29.9 to 37.2)	52.1 (43.2 to 59.7)	4.8 (3.4 to 6.1)	2.5 (2.2 to 2.7)	3.4 (2.8 to 3.9)	3.5 (2.0 to 4.8)

Acute lymphoid leukemia	Middle SDI	25.9 (20.5 to 28.4)	40.6 (29.1 to 46.9)	5.0 (3.3 to 6.4)	1.2 (1.0 to 1.4)	1.7 (1.2 to 2.0)	3.6 (2.0 to 5.0)
Acute lymphoid leukemia	Low-middle SDI	11.3 (9.29 to 14.4)	11.8 (9.96 to 14.1)	0.4 (-1.1 to 2.1)	0.7 (0.6 to 0.9)	0.7 (0.6 to 0.8)	-0.3 (-1.7 to 1.2)
Acute lymphoid leukemia	Low SDI	7.80 (5.38 to 11.7)	8.65 (6.37 to 11.0)	1.1 (-1.7 to 4.0)	0.7 (0.5 to 0.9)	0.6 (0.5 to 0.8)	-0.6 (-3.0 to 1.7)
Chronic lymphoid leukemia	Global	80.3 (74.9 to 90.7)	103 (93.5 to 119)	2.8 (2.0 to 3.6)	1.3 (1.2 to 1.5)	1.3 (1.2 to 1.5)	-0.1 (-0.9 to 0.7)
Chronic lymphoid leukemia	High SDI	39.0 (35.9 to 46.1)	44.4 (38.4 to 54.6)	1.4 (0.2 to 2.6)	2.5 (2.3 to 2.9)	2.3 (2.0 to 2.8)	-0.9 (-2.1 to 0.3)
Chronic lymphoid leukemia	High-middle SDI	24.1 (22.6 to 26.5)	30.7 (27.6 to 34.4)	2.7 (1.8 to 3.5)	1.5 (1.4 to 1.7)	1.5 (1.4 to 1.7)	0.2 (-0.7 to 1.0)
Chronic lymphoid leukemia	Middle SDI	10.0 (9.01 to 11.3)	16.7 (14.6 to 19.3)	5.7 (4.4 to 7.0)	0.5 (0.5 to 0.6)	0.7 (0.6 to 0.8)	2.7 (1.4 to 3.9)
Chronic lymphoid leukemia	Low-middle SDI	4.90 (4.40 to 5.59)	8.30 (7.22 to 9.55)	5.9 (4.4 to 7.2)	0.5 (0.4 to 0.6)	0.6 (0.5 to 0.7)	2.7 (1.2 to 4.0)
Chronic lymphoid leukemia	Low SDI	2.17 (1.79 to 2.54)	3.35 (2.82 to 3.95)	4.8 (3.7 to 6.0)	0.6 (0.5 to 0.7)	0.7 (0.6 to 0.8)	1.6 (0.5 to 2.7)
Acute myeloid leukemia	Global	104 (91.9 to 111)	124 (109 to 136)	1.9 (1.1 to 2.8)	1.6 (1.4 to 1.7)	1.6 (1.4 to 1.7)	-0.2 (-1.1 to 0.6)
Acute myeloid leukemia	High SDI	40.6 (33.2 to 42.9)	47.8 (38.5 to 53.5)	1.8 (0.9 to 2.8)	2.9 (2.4 to 3.0)	2.8 (2.3 to 3.1)	-0.4 (-1.4 to 0.6)
Acute myeloid leukemia	High-middle SDI	22.9 (19.3 to 24.0)	26.1 (21.8 to 28.9)	1.5 (0.5 to 2.4)	1.6 (1.3 to 1.6)	1.5 (1.2 to 1.7)	-0.4 (-1.4 to 0.5)
Acute myeloid leukemia	Middle SDI	21.2 (19.2 to 24.5)	25.4 (22.6 to 29.9)	2.0 (0.9 to 3.0)	1.0 (0.9 to 1.2)	1.1 (0.9 to 1.2)	0.2 (-0.8 to 1.2)
Acute myeloid leukemia	Low-middle SDI	13.0 (11.4 to 15.8)	16.2 (14.3 to 19.7)	2.4 (1.1 to 3.7)	1.0 (0.9 to 1.2)	1.0 (0.9 to 1.2)	0.7 (-0.5 to 1.8)
Acute myeloid leukemia	Low SDI	6.76 (4.91 to 8.66)	8.77 (6.82 to 10.7)	2.9 (0.9 to 4.9)	0.9 (0.7 to 1.1)	0.9 (0.7 to 1.1)	0.7 (-0.8 to 2.1)
Chronic myeloid leukemia	Global	58.5 (53.4 to 64.5)	65.8 (59.4 to 74.1)	1.3 (0.3 to 2.3)	0.9 (0.8 to 1.0)	0.8 (0.7 to 0.9)	-1.1 (-2.1 to -0.1)
Chronic myeloid leukemia	High SDI	26.3 (23.9 to 30.6)	29.6 (25.7 to 35.3)	1.3 (0.0 to 2.6)	1.8 (1.7 to 2.1)	1.7 (1.5 to 2.0)	-0.7 (-2.0 to 0.6)

Chronic myeloid leukemia	High-middle SDI	11.7 (10.9 to 12.8)	13.7 (12.1 to 15.4)	1.7 (0.6 to 2.8)	0.8 (0.7 to 0.8)	0.7 (0.6 to 0.8)	-0.5 (-1.5 to 0.6)
Chronic myeloid leukemia	Middle SDI	6.57 (5.98 to 7.24)	7.64 (6.76 to 8.76)	1.7 (0.5 to 2.8)	0.3 (0.3 to 0.4)	0.3 (0.3 to 0.4)	-0.6 (-1.8 to 0.5)
Chronic myeloid leukemia	Low-middle SDI	7.67 (6.23 to 9.36)	8.54 (7.06 to 10.3)	1.2 (-0.4 to 2.7)	0.6 (0.5 to 0.7)	0.6 (0.5 to 0.7)	-0.9 (-2.3 to 0.5)
Chronic myeloid leukemia	Low SDI	6.16 (4.24 to 9.62)	6.29 (4.61 to 8.15)	0.2 (-3.0 to 3.1)	0.9 (0.7 to 1.2)	0.8 (0.6 to 1.0)	-1.5 (-3.6 to 0.3)
Other leukemia	Global	205 (174 to 236)	197 (171 to 224)	-0.5 (-1.8 to 1.0)	3.1 (2.6 to 3.6)	2.6 (2.2 to 2.9)	-2.1 (-3.4 to -0.6)
Other leukemia	High SDI	24.0 (22.0 to 26.8)	26.7 (23.3 to 30.3)	1.2 (0.2 to 2.1)	1.9 (1.8 to 2.1)	1.7 (1.5 to 1.9)	-1.0 (-2.0 to -0.0)
Other leukemia	High-middle SDI	48.3 (41.7 to 52.6)	45.2 (37.9 to 52.1)	-0.7 (-2.2 to 0.7)	3.7 (3.2 to 4.1)	3.0 (2.4 to 3.5)	-2.5 (-4.1 to -0.9)
Other leukemia	Middle SDI	79.3 (68.1 to 93.0)	73.3 (62.3 to 86.8)	-0.9 (-2.3 to 0.7)	4.0 (3.4 to 4.7)	3.2 (2.7 to 3.8)	-2.5 (-4.0 to -0.9)
Other leukemia	Low-middle SDI	31.7 (23.0 to 41.2)	29.3 (24.6 to 36.0)	-0.9 (-2.9 to 1.5)	2.2 (1.7 to 2.8)	1.8 (1.6 to 2.3)	-2.1 (-3.8 to -0.0)
Other leukemia	Low SDI	21.9 (12.0 to 31.7)	22.0 (14.4 to 29.2)	0.0 (-2.7 to 3.5)	2.3 (1.5 to 3.1)	2.0 (1.4 to 2.6)	-1.4 (-3.5 to 1.0)
Other malignant neoplasms	Global	652 (594 to 686)	831 (741 to 906)	2.7 (2.0 to 3.4)	9.9 (9.0 to 10.5)	10.4 (9.3 to 11.4)	0.5 (-0.2 to 1.2)
Other malignant neoplasms	High SDI	182 (163 to 190)	209 (184 to 230)	1.5 (0.6 to 2.4)	14.3 (13.0 to 14.9)	14.2 (12.8 to 15.6)	-0.1 (-1.1 to 0.8)
Other malignant neoplasms	High-middle SDI	173 (158 to 181)	215 (193 to 237)	2.4 (1.5 to 3.4)	11.6 (10.7 to 12.1)	12.3 (11.1 to 13.4)	0.7 (-0.3 to 1.6)
Other malignant neoplasms	Middle SDI	165 (148 to 175)	230 (201 to 256)	3.7 (2.6 to 4.7)	8.4 (7.5 to 8.9)	9.5 (8.3 to 10.6)	1.5 (0.5 to 2.4)
Other malignant neoplasms	Low-middle SDI	90.8 (76.6 to 102)	121 (100 to 139)	3.2 (2.3 to 4.2)	7.3 (6.1 to 8.2)	8.1 (6.6 to 9.3)	1.0 (0.1 to 2.0)
Other malignant neoplasms	Low SDI	41.4 (36.0 to 47.0)	56.6 (48.0 to 65.2)	3.5 (2.5 to 4.4)	7.3 (6.2 to 8.2)	7.6 (6.4 to 8.7)	0.6 (-0.4 to 1.4)

Abbreviations: 95% UI, 95% uncertainty interval; NMSC, non-melanoma skin cancer; SDI, sociodemographic index.

Footnote: Sociodemographic index (SDI) quintiles do not sum exactly to the global total because several territories are included in the global total but not in any SDI quintile totals. “Total cancers” in this table excludes the “Other neoplasms” category of benign and in situ neoplasms.

eTable 17: Trends in mortality globally, and by SDI quintile, both sexes, 2010 to 2019

Cause	Location	Mortality 2010, thousands (95% UI)	Mortality 2019, thousands (95% UI)	Annualized rate of change 2010-2019, counts (95% UI)	Age-standardized mortality 2010, per 100,000 (95% UI)	Age-standardized mortality 2019, per 100,000 (95% UI)	Annualized rate of change 2010-2019, age-standardized rate (95% UI)
Total cancers	Global	8 290 (7 890 to 8 570)	10 000 (9 360 to 10 600)	2.1 (1.5 to 2.7)	132.5 (125.5 to 137.1)	124.7 (116.4 to 132.0)	-0.7 (-1.3 to -0.1)
Total cancers	High SDI	2 200 (2 040 to 2 280)	2 540 (2 320 to 2 650)	1.6 (1.4 to 1.8)	139.7 (130.8 to 144.0)	130.8 (121.3 to 135.9)	-0.7 (-0.9 to -0.5)
Total cancers	High-middle SDI	2 320 (2 210 to 2 420)	2 660 (2 440 to 2 860)	1.5 (0.7 to 2.3)	145.6 (138.1 to 151.6)	132.7 (121.5 to 142.5)	-1.0 (-1.9 to -0.2)
Total cancers	Middle SDI	2 340 (2 210 to 2 460)	2 900 (2 630 to 3 160)	2.4 (1.3 to 3.5)	130.3 (123.0 to 137.0)	121.4 (110.1 to 132.3)	-0.8 (-1.9 to 0.3)
Total cancers	Low-middle SDI	1 020 (968 to 1 080)	1 380 (1 270 to 1 490)	3.3 (2.4 to 4.1)	99.7 (93.8 to 105.2)	102.4 (94.3 to 110.8)	0.3 (-0.5 to 1.1)
Total cancers	Low SDI	403 (364 to 440)	538 (483 to 598)	3.2 (2.4 to 4.0)	99.7 (89.4 to 108.9)	100.7 (90.0 to 111.5)	0.1 (-0.6 to 0.8)
Total Cancers (excl. NMSC)	Global	8 250 (7 850 to 8 520)	9 970 (9 310 to 10 500)	2.1 (1.5 to 2.7)	131.8 (124.8 to 136.3)	123.9 (115.7 to 131.2)	-0.7 (-1.3 to -0.1)
Total Cancers (excl. NMSC)	High SDI	2 190 (2 030 to 2 270)	2 530 (2 310 to 2 640)	1.6 (1.4 to 1.8)	139.1 (130.3 to 143.4)	130.2 (120.8 to 135.3)	-0.7 (-0.9 to -0.5)
Total Cancers (excl. NMSC)	High-middle SDI	2 310 (2 200 to 2 400)	2 650 (2 420 to 2 850)	1.5 (0.7 to 2.3)	144.8 (137.3 to 150.7)	131.9 (120.7 to 141.7)	-1.0 (-1.9 to -0.2)
Total Cancers (excl. NMSC)	Middle SDI	2 320 (2 190 to 2 440)	2 880 (2 620 to 3 150)	2.4 (1.3 to 3.5)	129.4 (122.1 to 136.1)	120.5 (109.3 to 131.4)	-0.8 (-1.9 to 0.3)
Total Cancers (excl. NMSC)	Low-middle SDI	1 020 (962 to 1 080)	1 370 (1 260 to 1 490)	3.3 (2.4 to 4.1)	99.0 (93.1 to 104.6)	101.7 (93.7 to 110.1)	0.3 (-0.5 to 1.1)
Total Cancers (excl. NMSC)	Low SDI	402 (364 to 438)	536 (481 to 596)	3.2 (2.4 to 4.0)	99.3 (89.2 to 108.4)	100.3 (89.6 to 110.9)	0.1 (-0.6 to 0.8)
Lip and oral cavity cancer	Global	157 (151 to 164)	199 (182 to 218)	2.6 (1.7 to 3.5)	2.4 (2.3 to 2.6)	2.4 (2.2 to 2.7)	-0.0 (-0.9 to 0.9)

Lip and oral cavity cancer	High SDI	26.2 (24.5 to 27.1)	29.4 (27.1 to 31.0)	1.3 (0.9 to 1.7)	1.7 (1.6 to 1.8)	1.6 (1.5 to 1.7)	-0.9 (-1.3 to -0.5)
Lip and oral cavity cancer	High-middle SDI	32.9 (31.7 to 34.0)	37.5 (34.6 to 40.5)	1.4 (0.5 to 2.3)	2.0 (2.0 to 2.1)	1.9 (1.7 to 2.0)	-1.0 (-1.9 to -0.2)
Lip and oral cavity cancer	Middle SDI	39.6 (37.4 to 42.7)	52.1 (46.1 to 58.8)	3.1 (1.8 to 4.3)	2.2 (2.0 to 2.3)	2.2 (1.9 to 2.4)	-0.0 (-1.2 to 1.1)
Lip and oral cavity cancer	Low-middle SDI	43.8 (40.9 to 46.8)	60.3 (53.1 to 69.0)	3.5 (2.0 to 5.1)	4.2 (3.9 to 4.5)	4.4 (3.9 to 5.0)	0.6 (-0.9 to 2.1)
Lip and oral cavity cancer	Low SDI	14.7 (13.1 to 16.2)	20.0 (17.7 to 22.4)	3.4 (1.8 to 5.0)	3.7 (3.3 to 4.1)	3.8 (3.3 to 4.2)	0.3 (-1.3 to 1.8)
Nasopharynx cancer	Global	61.7 (58.4 to 65.2)	71.6 (65.4 to 77.6)	1.7 (0.6 to 2.6)	0.9 (0.9 to 1.0)	0.9 (0.8 to 0.9)	-0.7 (-1.8 to 0.2)
Nasopharynx cancer	High SDI	4.99 (4.78 to 5.13)	5.55 (5.14 to 5.98)	1.2 (0.5 to 1.9)	0.4 (0.3 to 0.4)	0.3 (0.3 to 0.4)	-0.6 (-1.3 to 0.2)
Nasopharynx cancer	High-middle SDI	15.4 (14.2 to 16.7)	17.2 (14.9 to 19.7)	1.2 (-0.4 to 2.9)	0.9 (0.9 to 1.0)	0.9 (0.8 to 1.0)	-0.8 (-2.5 to 0.8)
Nasopharynx cancer	Middle SDI	25.1 (23.5 to 27.1)	28.7 (25.6 to 32.1)	1.5 (0.2 to 2.9)	1.3 (1.2 to 1.4)	1.1 (1.0 to 1.2)	-1.3 (-2.6 to 0.0)
Nasopharynx cancer	Low-middle SDI	11.8 (10.9 to 12.8)	14.5 (13.2 to 16.1)	2.3 (1.2 to 3.3)	1.0 (0.9 to 1.1)	1.0 (0.9 to 1.1)	-0.5 (-1.5 to 0.6)
Nasopharynx cancer	Low SDI	4.36 (3.93 to 4.84)	5.54 (4.90 to 6.23)	2.7 (1.4 to 3.9)	0.9 (0.9 to 1.0)	0.9 (0.8 to 1.0)	-0.4 (-1.6 to 0.8)
Other pharynx cancer	Global	87.5 (83.3 to 91.7)	114 (103 to 126)	3.0 (1.9 to 4.0)	1.3 (1.3 to 1.4)	1.4 (1.2 to 1.5)	0.3 (-0.8 to 1.4)
Other pharynx cancer	High SDI	14.4 (13.9 to 14.8)	16.4 (15.4 to 17.4)	1.4 (0.9 to 2.1)	1.0 (1.0 to 1.0)	0.9 (0.9 to 1.0)	-0.6 (-1.2 to 0.0)
Other pharynx cancer	High-middle SDI	15.4 (15.0 to 15.8)	18.0 (16.7 to 19.3)	1.7 (0.9 to 2.5)	0.9 (0.9 to 1.0)	0.9 (0.8 to 0.9)	-0.7 (-1.5 to 0.1)
Other pharynx cancer	Middle SDI	18.7 (17.6 to 20.1)	25.6 (22.3 to 28.8)	3.5 (2.1 to 4.7)	1.0 (0.9 to 1.0)	1.0 (0.9 to 1.1)	0.4 (-1.0 to 1.6)
Other pharynx cancer	Low-middle SDI	32.1 (29.5 to 34.6)	44.7 (38.6 to 51.8)	3.7 (1.9 to 5.4)	3.0 (2.8 to 3.3)	3.2 (2.8 to 3.7)	0.7 (-1.0 to 2.5)
Other pharynx cancer	Low SDI	6.76 (5.87 to 7.52)	9.46 (8.11 to 11.0)	3.7 (2.1 to 5.4)	1.7 (1.4 to 1.9)	1.8 (1.5 to 2.1)	0.7 (-1.0 to 2.3)

Esophageal cancer	Global	453 (366 to 485)	498 (438 to 551)	1.1 (-0.2 to 2.6)	7.2 (5.8 to 7.7)	6.1 (5.4 to 6.8)	-1.8 (-3.0 to -0.3)
Esophageal cancer	High SDI	68.4 (64.6 to 70.9)	79.1 (73.6 to 83.1)	1.6 (1.3 to 2.0)	4.4 (4.2 to 4.6)	4.2 (3.9 to 4.4)	-0.6 (-1.0 to -0.3)
Esophageal cancer	High-middle SDI	121 (92.2 to 133)	136 (108 to 157)	1.3 (-0.5 to 2.9)	7.5 (5.7 to 8.2)	6.6 (5.3 to 7.6)	-1.3 (-3.1 to 0.3)
Esophageal cancer	Middle SDI	194 (128 to 214)	194 (158 to 224)	-0.0 (-1.8 to 2.9)	11.1 (7.2 to 12.2)	8.1 (6.5 to 9.4)	-3.4 (-5.1 to -0.5)
Esophageal cancer	Low-middle SDI	47.6 (43.4 to 65.6)	60.7 (54.0 to 85.6)	2.7 (1.7 to 3.6)	4.7 (4.3 to 6.5)	4.5 (4.0 to 6.4)	-0.5 (-1.4 to 0.5)
Esophageal cancer	Low SDI	22.0 (18.8 to 25.2)	28.7 (23.8 to 34.3)	3.0 (1.9 to 4.1)	5.9 (5.0 to 6.7)	5.7 (4.8 to 6.8)	-0.4 (-1.4 to 0.7)
Stomach cancer	Global	922 (870 to 970)	957 (871 to 1 030)	0.4 (-0.5 to 1.4)	14.7 (13.8 to 15.5)	11.9 (10.8 to 12.8)	-2.4 (-3.3 to -1.4)
Stomach cancer	High SDI	140 (127 to 146)	145 (129 to 154)	0.4 (0.1 to 0.7)	8.7 (8.0 to 9.0)	7.2 (6.5 to 7.6)	-2.1 (-2.4 to -1.8)
Stomach cancer	High-middle SDI	284 (266 to 301)	281 (251 to 309)	-0.1 (-1.3 to 1.0)	17.6 (16.4 to 18.7)	13.8 (12.4 to 15.2)	-2.7 (-3.9 to -1.5)
Stomach cancer	Middle SDI	342 (317 to 367)	345 (305 to 388)	0.1 (-1.3 to 1.6)	19.5 (18.1 to 20.8)	14.6 (13.0 to 16.3)	-3.2 (-4.6 to -1.8)
Stomach cancer	Low-middle SDI	121 (114 to 128)	142 (130 to 155)	1.8 (0.8 to 2.6)	12.2 (11.4 to 12.9)	10.8 (9.9 to 11.8)	-1.3 (-2.2 to -0.5)
Stomach cancer	Low SDI	36.3 (32.6 to 39.7)	43.9 (39.3 to 48.9)	2.1 (1.4 to 2.9)	9.9 (9.0 to 10.8)	8.9 (8.1 to 9.9)	-1.2 (-1.8 to -0.5)
Colon and rectum cancer	Global	858 (806 to 892)	1 090 (1 000 to 1 150)	2.6 (2.0 to 3.2)	14.1 (13.2 to 14.7)	13.7 (12.6 to 14.5)	-0.3 (-0.9 to 0.2)
Colon and rectum cancer	High SDI	281 (257 to 294)	328 (295 to 346)	1.7 (1.4 to 1.9)	17.3 (16.0 to 18.0)	16.3 (14.9 to 17.1)	-0.7 (-0.9 to -0.5)
Colon and rectum cancer	High-middle SDI	271 (257 to 281)	327 (300 to 350)	2.1 (1.4 to 2.8)	17.1 (16.1 to 17.8)	16.2 (14.9 to 17.4)	-0.6 (-1.3 to 0.1)
Colon and rectum cancer	Middle SDI	205 (193 to 217)	280 (251 to 306)	3.5 (2.4 to 4.5)	11.8 (11.0 to 12.5)	12.0 (10.8 to 13.1)	0.2 (-0.8 to 1.1)
Colon and rectum cancer	Low-middle SDI	77.1 (72.5 to 81.8)	117 (106 to 128)	4.6 (3.6 to 5.5)	8.0 (7.5 to 8.5)	9.1 (8.2 to 9.9)	1.4 (0.4 to 2.4)

Colon and rectum cancer	Low SDI	23.4 (20.9 to 26.5)	34.7 (31.0 to 38.6)	4.4 (3.2 to 5.4)	6.6 (5.9 to 7.6)	7.3 (6.5 to 8.1)	1.1 (-0.0 to 2.1)
Liver cancer	Global	386 (366 to 405)	485 (444 to 526)	2.5 (1.6 to 3.4)	6.0 (5.7 to 6.3)	5.9 (5.4 to 6.4)	-0.2 (-1.1 to 0.7)
Liver cancer	High SDI	97.0 (89.7 to 101)	112 (102 to 119)	1.6 (1.0 to 2.0)	6.2 (5.8 to 6.5)	5.9 (5.4 to 6.2)	-0.6 (-1.3 to -0.2)
Liver cancer	High-middle SDI	83.4 (78.2 to 88.6)	97.2 (87.2 to 108)	1.7 (0.3 to 3.0)	5.1 (4.8 to 5.5)	4.8 (4.3 to 5.4)	-0.7 (-2.1 to 0.6)
Liver cancer	Middle SDI	147 (138 to 159)	197 (173 to 223)	3.2 (1.7 to 4.7)	7.8 (7.3 to 8.4)	7.9 (7.0 to 8.9)	0.1 (-1.4 to 1.5)
Liver cancer	Low-middle SDI	42.1 (38.8 to 45.2)	57.2 (52.1 to 63.5)	3.4 (2.4 to 4.4)	4.1 (3.7 to 4.4)	4.2 (3.9 to 4.7)	0.4 (-0.6 to 1.4)
Liver cancer	Low SDI	16.4 (14.8 to 18.1)	20.8 (18.2 to 23.3)	2.6 (1.6 to 3.6)	4.2 (3.8 to 4.6)	3.9 (3.5 to 4.4)	-0.7 (-1.6 to 0.2)
Gallbladder and biliary tract cancer	Global	143 (121 to 152)	172 (145 to 189)	2.1 (1.4 to 2.9)	2.4 (2.0 to 2.5)	2.2 (1.8 to 2.4)	-0.9 (-1.6 to -0.1)
Gallbladder and biliary tract cancer	High SDI	42.5 (33.5 to 45.8)	48.4 (38.3 to 53.4)	1.5 (1.0 to 1.9)	2.6 (2.1 to 2.8)	2.3 (1.9 to 2.5)	-1.1 (-1.5 to -0.6)
Gallbladder and biliary tract cancer	High-middle SDI	39.2 (30.2 to 42.0)	44.0 (33.5 to 49.4)	1.3 (0.4 to 2.3)	2.5 (1.9 to 2.7)	2.2 (1.7 to 2.4)	-1.4 (-2.3 to -0.5)
Gallbladder and biliary tract cancer	Middle SDI	33.6 (30.1 to 36.9)	42.3 (36.9 to 49.2)	2.6 (1.4 to 3.9)	2.0 (1.8 to 2.2)	1.8 (1.6 to 2.1)	-0.9 (-2.0 to 0.4)
Gallbladder and biliary tract cancer	Low-middle SDI	20.8 (18.3 to 24.6)	28.7 (25.3 to 33.1)	3.6 (2.4 to 4.8)	2.2 (1.9 to 2.6)	2.2 (2.0 to 2.6)	0.4 (-0.8 to 1.5)
Gallbladder and biliary tract cancer	Low SDI	6.65 (5.53 to 7.72)	8.95 (7.45 to 10.3)	3.3 (2.1 to 4.6)	1.9 (1.6 to 2.2)	1.9 (1.6 to 2.2)	0.1 (-1.0 to 1.4)
Pancreatic cancer	Global	389 (367 to 404)	531 (492 to 567)	3.5 (2.9 to 4.0)	6.3 (5.9 to 6.6)	6.6 (6.1 to 7.1)	0.5 (-0.1 to 1.0)
Pancreatic cancer	High SDI	152 (140 to 158)	190 (171 to 201)	2.5 (2.1 to 2.8)	9.5 (8.9 to 9.9)	9.6 (8.8 to 10.2)	0.1 (-0.2 to 0.5)
Pancreatic cancer	High-middle SDI	123 (117 to 128)	160 (146 to 171)	2.9 (2.1 to 3.7)	7.7 (7.3 to 8.0)	7.8 (7.2 to 8.4)	0.2 (-0.6 to 1.0)
Pancreatic cancer	Middle SDI	77.2 (72.2 to 82.4)	120 (107 to 135)	4.9 (3.9 to 5.9)	4.4 (4.1 to 4.7)	5.0 (4.5 to 5.6)	1.5 (0.6 to 2.5)

Pancreatic cancer	Low-middle SDI	28.7 (26.9 to 30.8)	48.5 (44.3 to 53.1)	5.8 (4.8 to 6.8)	3.0 (2.8 to 3.2)	3.8 (3.4 to 4.1)	2.6 (1.6 to 3.6)
Pancreatic cancer	Low SDI	8.04 (6.84 to 9.28)	12.9 (11.3 to 14.7)	5.3 (4.1 to 6.6)	2.3 (1.9 to 2.6)	2.7 (2.4 to 3.1)	2.0 (0.9 to 3.2)
Larynx cancer	Global	105 (101 to 109)	123 (115 to 133)	1.8 (1.0 to 2.5)	1.6 (1.6 to 1.7)	1.5 (1.4 to 1.6)	-1.0 (-1.7 to -0.3)
Larynx cancer	High SDI	13.2 (12.6 to 13.5)	14.6 (13.7 to 15.3)	1.1 (0.8 to 1.5)	0.9 (0.8 to 0.9)	0.8 (0.7 to 0.8)	-1.0 (-1.4 to -0.6)
Larynx cancer	High-middle SDI	29.1 (28.1 to 29.9)	30.4 (28.1 to 32.5)	0.5 (-0.3 to 1.2)	1.8 (1.7 to 1.8)	1.5 (1.4 to 1.6)	-2.1 (-2.9 to -1.3)
Larynx cancer	Middle SDI	28.1 (26.8 to 29.6)	34.9 (31.6 to 38.2)	2.4 (1.4 to 3.4)	1.5 (1.5 to 1.6)	1.4 (1.3 to 1.6)	-0.9 (-1.9 to 0.0)
Larynx cancer	Low-middle SDI	25.5 (23.5 to 27.7)	32.1 (28.7 to 36.2)	2.6 (1.2 to 3.9)	2.4 (2.3 to 2.7)	2.3 (2.1 to 2.6)	-0.5 (-1.8 to 0.7)
Larynx cancer	Low SDI	9.10 (7.99 to 10.1)	11.2 (9.79 to 12.8)	2.3 (0.9 to 3.8)	2.3 (2.1 to 2.6)	2.2 (1.9 to 2.5)	-0.9 (-2.2 to 0.5)
Tracheal, bronchus, and lung cancer	Global	1 670 (1 590 to 1 740)	2 040 (1 880 to 2 190)	2.2 (1.4 to 3.1)	26.7 (25.3 to 27.8)	25.2 (23.2 to 27.0)	-0.7 (-1.5 to 0.2)
Tracheal, bronchus, and lung cancer	High SDI	515 (482 to 532)	578 (534 to 603)	1.3 (1.0 to 1.5)	32.7 (30.9 to 33.7)	29.8 (27.8 to 31.0)	-1.0 (-1.3 to -0.8)
Tracheal, bronchus, and lung cancer	High-middle SDI	523 (497 to 547)	614 (559 to 670)	1.8 (0.7 to 2.9)	32.2 (30.5 to 33.6)	29.9 (27.2 to 32.6)	-0.8 (-1.9 to 0.2)
Tracheal, bronchus, and lung cancer	Middle SDI	473 (442 to 507)	630 (551 to 712)	3.2 (1.7 to 4.6)	26.8 (25.0 to 28.7)	26.3 (23.0 to 29.7)	-0.2 (-1.6 to 1.2)
Tracheal, bronchus, and lung cancer	Low-middle SDI	126 (118 to 135)	174 (158 to 190)	3.6 (2.4 to 4.6)	12.7 (11.9 to 13.6)	13.2 (12.0 to 14.3)	0.4 (-0.7 to 1.3)
Tracheal, bronchus, and lung cancer	Low SDI	33.2 (28.2 to 39.5)	46.0 (39.6 to 53.9)	3.6 (2.4 to 4.7)	9.1 (7.7 to 10.8)	9.4 (8.1 to 10.9)	0.3 (-0.8 to 1.4)
Malignant skin melanoma	Global	53.4 (39.3 to 58.9)	62.8 (46.3 to 71.0)	1.8 (1.3 to 2.4)	0.9 (0.6 to 0.9)	0.8 (0.6 to 0.9)	-0.9 (-1.4 to -0.4)
Malignant skin melanoma	High SDI	25.4 (16.5 to 28.2)	29.4 (19.5 to 34.2)	1.7 (1.1 to 2.3)	1.7 (1.1 to 1.9)	1.6 (1.1 to 1.9)	-0.5 (-1.1 to 0.3)
Malignant skin melanoma	High-middle SDI	16.1 (11.7 to 18.0)	18.0 (12.9 to 20.2)	1.2 (0.5 to 1.9)	1.0 (0.7 to 1.1)	0.9 (0.7 to 1.0)	-1.1 (-1.9 to -0.4)

Malignant skin melanoma	Middle SDI	6.73 (5.28 to 7.93)	8.56 (6.75 to 10.2)	2.7 (1.5 to 3.7)	0.4 (0.3 to 0.4)	0.4 (0.3 to 0.4)	-0.3 (-1.4 to 0.7)
Malignant skin melanoma	Low-middle SDI	3.16 (2.42 to 3.82)	4.16 (3.26 to 5.00)	3.1 (2.0 to 4.0)	0.3 (0.2 to 0.4)	0.3 (0.2 to 0.4)	0.0 (-0.9 to 0.9)
Malignant skin melanoma	Low SDI	2.02 (1.49 to 2.57)	2.71 (2.01 to 3.44)	3.3 (1.7 to 4.7)	0.5 (0.4 to 0.6)	0.5 (0.4 to 0.6)	0.0 (-1.4 to 1.2)
Non-melanoma skin cancer	Global	43.3 (39.3 to 45.3)	56.1 (50.4 to 59.8)	2.9 (2.3 to 3.5)	0.7 (0.7 to 0.8)	0.7 (0.7 to 0.8)	-0.3 (-0.9 to 0.3)
Non-melanoma skin cancer	High SDI	9.84 (8.65 to 10.4)	13.0 (11.1 to 13.9)	3.1 (2.7 to 3.4)	0.6 (0.5 to 0.6)	0.6 (0.5 to 0.6)	0.4 (0.1 to 0.7)
Non-melanoma skin cancer	High-middle SDI	12.5 (11.6 to 13.1)	15.5 (13.9 to 16.6)	2.3 (1.6 to 3.0)	0.9 (0.8 to 0.9)	0.8 (0.7 to 0.9)	-0.8 (-1.5 to -0.2)
Non-melanoma skin cancer	Middle SDI	13.7 (12.7 to 14.5)	17.7 (15.8 to 19.4)	2.8 (1.8 to 3.8)	0.9 (0.8 to 1.0)	0.8 (0.7 to 0.9)	-0.8 (-1.7 to 0.1)
Non-melanoma skin cancer	Low-middle SDI	5.78 (5.06 to 6.23)	7.86 (6.86 to 8.61)	3.4 (2.5 to 4.2)	0.7 (0.6 to 0.8)	0.7 (0.6 to 0.8)	-0.2 (-1.0 to 0.5)
Non-melanoma skin cancer	Low SDI	1.36 (0.904 to 1.61)	1.97 (1.31 to 2.35)	4.1 (3.2 to 4.9)	0.5 (0.3 to 0.5)	0.5 (0.3 to 0.6)	0.5 (-0.3 to 1.3)
Breast cancer	Global	565 (536 to 586)	701 (647 to 752)	2.4 (1.7 to 3.1)	8.8 (8.3 to 9.2)	8.6 (7.9 to 9.2)	-0.3 (-1.0 to 0.4)
Breast cancer	High SDI	152 (140 to 159)	168 (152 to 177)	1.1 (0.7 to 1.4)	10.0 (9.3 to 10.3)	9.0 (8.4 to 9.5)	-1.1 (-1.4 to -0.7)
Breast cancer	High-middle SDI	147 (139 to 152)	166 (153 to 180)	1.4 (0.5 to 2.2)	9.2 (8.7 to 9.5)	8.3 (7.6 to 9.0)	-1.1 (-1.9 to -0.2)
Breast cancer	Middle SDI	141 (134 to 149)	185 (166 to 205)	3.0 (1.8 to 4.1)	7.2 (6.8 to 7.6)	7.3 (6.6 to 8.1)	0.1 (-1.0 to 1.2)
Breast cancer	Low-middle SDI	87.6 (80.5 to 95.3)	127 (110 to 145)	4.2 (2.7 to 5.5)	8.0 (7.3 to 8.7)	8.9 (7.8 to 10.2)	1.2 (-0.2 to 2.6)
Breast cancer	Low SDI	36.5 (32.3 to 40.8)	54.5 (47.5 to 62.1)	4.4 (3.1 to 5.8)	8.8 (7.9 to 9.9)	9.8 (8.6 to 11.1)	1.2 (-0.1 to 2.4)
Cervical cancer	Global	238 (211 to 269)	280 (239 to 314)	1.8 (0.9 to 2.8)	3.6 (3.2 to 4.1)	3.4 (2.9 to 3.8)	-0.6 (-1.5 to 0.4)
Cervical cancer	High SDI	24.2 (21.5 to 25.5)	26.2 (22.8 to 28.1)	0.9 (0.5 to 1.4)	1.7 (1.5 to 1.7)	1.5 (1.4 to 1.6)	-0.9 (-1.4 to -0.5)

Cervical cancer	High-middle SDI	47.5<br>(42.2 to 50.5)	51.8<br>(41.7 to 57.9)	1.0<br>(-0.4 to 2.2)	2.9<br>(2.6 to 3.1)	2.6<br>(2.1 to 2.9)	-1.2<br>(-2.5 to 0.0)
Cervical cancer	Middle SDI	77.0<br>(63.9 to 84.0)	90.1<br>(71.3 to 103)	1.8<br>(0.4 to 3.1)	3.9<br>(3.3 to 4.3)	3.5<br>(2.8 to 4.1)	-1.1<br>(-2.5 to 0.2)
Cervical cancer	Low-middle SDI	53.1<br>(47.1 to 65.2)	66.7<br>(57.3 to 81.2)	2.5<br>(1.2 to 3.8)	4.7<br>(4.2 to 5.8)	4.6<br>(3.9 to 5.6)	-0.3<br>(-1.6 to 0.9)
Cervical cancer	Low SDI	36.0<br>(28.6 to 42.9)	45.5<br>(35.8 to 56.3)	2.6<br>(1.4 to 3.9)	8.1<br>(6.5 to 9.7)	7.7<br>(6.1 to 9.4)	-0.6<br>(-1.7 to 0.6)
Uterine cancer	Global	79.6<br>(69.2 to 84.1)	91.6<br>(82.4 to 102)	1.6<br>(0.8 to 2.7)	1.3<br>(1.1 to 1.4)	1.1<br>(1.0 to 1.3)	-1.3<br>(-2.0 to -0.3)
Uterine cancer	High SDI	22.2<br>(20.1 to 23.2)	26.6<br>(24.0 to 28.1)	2.0<br>(1.8 to 2.3)	1.4<br>(1.3 to 1.4)	1.4<br>(1.2 to 1.4)	-0.2<br>(-0.5 to 0.1)
Uterine cancer	High-middle SDI	24.4<br>(22.0 to 25.8)	26.4<br>(24.0 to 28.8)	0.9<br>(-0.0 to 2.0)	1.5<br>(1.4 to 1.6)	1.3<br>(1.2 to 1.4)	-1.8<br>(-2.7 to -0.7)
Uterine cancer	Middle SDI	20.0<br>(14.1 to 22.0)	21.0<br>(17.5 to 24.3)	0.5<br>(-1.1 to 3.3)	1.1<br>(0.8 to 1.2)	0.9<br>(0.7 to 1.0)	-2.6<br>(-4.1 to -0.1)
Uterine cancer	Low-middle SDI	9.12<br>(7.92 to 11.1)	12.2<br>(10.4 to 15.3)	3.3<br>(2.1 to 4.5)	0.9<br>(0.8 to 1.1)	0.9<br>(0.8 to 1.2)	0.1<br>(-1.0 to 1.3)
Uterine cancer	Low SDI	3.80<br>(3.10 to 4.69)	5.30<br>(4.32 to 6.64)	3.7<br>(2.5 to 5.0)	1.0<br>(0.8 to 1.3)	1.1<br>(0.9 to 1.4)	0.4<br>(-0.8 to 1.6)
Ovarian cancer	Global	153<br>(142 to 165)	198<br>(175 to 218)	2.9<br>(1.9 to 3.6)	2.4<br>(2.2 to 2.6)	2.4<br>(2.1 to 2.7)	0.1<br>(-0.8 to 0.8)
Ovarian cancer	High SDI	50.9<br>(46.5 to 53.4)	56.6<br>(50.4 to 61.3)	1.2<br>(0.7 to 1.6)	3.3<br>(3.1 to 3.4)	3.0<br>(2.7 to 3.3)	-0.9<br>(-1.4 to -0.5)
Ovarian cancer	High-middle SDI	44.4<br>(41.3 to 46.6)	52.0<br>(45.0 to 57.2)	1.8<br>(0.6 to 2.7)	2.7<br>(2.5 to 2.9)	2.6<br>(2.2 to 2.8)	-0.7<br>(-1.8 to 0.3)
Ovarian cancer	Middle SDI	32.7<br>(29.1 to 37.0)	48.5<br>(39.9 to 56.5)	4.4<br>(2.7 to 5.6)	1.7<br>(1.5 to 1.9)	1.9<br>(1.6 to 2.2)	1.4<br>(-0.2 to 2.6)
Ovarian cancer	Low-middle SDI	18.5<br>(16.1 to 22.4)	29.9<br>(24.4 to 37.6)	5.3<br>(3.2 to 6.8)	1.7<br>(1.5 to 2.1)	2.1<br>(1.7 to 2.7)	2.3<br>(0.3 to 3.8)
Ovarian cancer	Low SDI	6.84<br>(5.48 to 9.99)	11.3<br>(9.55 to 13.9)	5.6<br>(3.0 to 7.8)	1.7<br>(1.3 to 2.4)	2.0<br>(1.7 to 2.5)	2.4<br>(-0.1 to 4.5)
Prostate cancer	Global	378<br>(319 to 449)	487<br>(420 to 594)	2.8<br>(2.3 to 3.3)	6.5<br>(5.5 to 7.8)	6.3<br>(5.4 to 7.7)	-0.4<br>(-0.9 to 0.1)

Prostate cancer	High SDI	127 (103 to 167)	158 (131 to 215)	2.4 (2.1 to 2.9)	7.4 (6.1 to 9.6)	7.2 (6.1 to 9.9)	-0.2 (-0.5 to 0.3)
Prostate cancer	High-middle SDI	93.2 (78.3 to 113)	114 (97.0 to 142)	2.3 (1.6 to 2.9)	6.1 (5.1 to 7.4)	5.7 (4.8 to 7.1)	-0.7 (-1.3 to -0.1)
Prostate cancer	Middle SDI	83.5 (70.1 to 95.8)	112 (94.0 to 133)	3.3 (2.4 to 4.1)	5.8 (4.8 to 6.6)	5.5 (4.6 to 6.5)	-0.6 (-1.4 to 0.3)
Prostate cancer	Low-middle SDI	48.1 (40.6 to 56.0)	66.6 (56.3 to 78.8)	3.6 (2.7 to 4.5)	6.2 (5.2 to 7.3)	6.0 (5.1 to 7.2)	-0.3 (-1.2 to 0.6)
Prostate cancer	Low SDI	26.0 (18.8 to 30.8)	35.2 (25.9 to 41.6)	3.4 (2.5 to 4.3)	9.2 (6.6 to 10.9)	9.1 (6.6 to 10.7)	-0.1 (-0.9 to 0.7)
Testicular cancer	Global	9.13 (8.69 to 10.1)	10.8 (9.96 to 11.9)	1.9 (1.1 to 2.7)	0.1 (0.1 to 0.1)	0.1 (0.1 to 0.2)	0.4 (-0.3 to 1.2)
Testicular cancer	High SDI	1.38 (1.34 to 1.43)	1.44 (1.36 to 1.53)	0.5 (-0.0 to 1.0)	0.1 (0.1 to 0.1)	0.1 (0.1 to 0.1)	-0.8 (-1.3 to -0.2)
Testicular cancer	High-middle SDI	2.52 (2.42 to 2.69)	2.80 (2.56 to 3.05)	1.2 (0.3 to 2.0)	0.2 (0.2 to 0.2)	0.2 (0.2 to 0.2)	0.2 (-0.7 to 1.1)
Testicular cancer	Middle SDI	2.72 (2.56 to 3.11)	3.38 (3.03 to 3.82)	2.4 (1.3 to 3.5)	0.1 (0.1 to 0.1)	0.1 (0.1 to 0.2)	1.0 (-0.1 to 2.0)
Testicular cancer	Low-middle SDI	1.73 (1.56 to 2.00)	2.17 (1.89 to 2.52)	2.5 (1.1 to 4.0)	0.1 (0.1 to 0.1)	0.1 (0.1 to 0.1)	0.7 (-0.7 to 2.1)
Testicular cancer	Low SDI	0.778 (0.654 to 0.915)	1.05 (0.886 to 1.25)	3.3 (1.4 to 5.3)	0.1 (0.1 to 0.1)	0.1 (0.1 to 0.1)	0.5 (-1.3 to 2.5)
Kidney cancer	Global	132 (125 to 136)	166 (155 to 176)	2.6 (2.0 to 3.1)	2.1 (2.0 to 2.2)	2.1 (1.9 to 2.2)	-0.3 (-0.8 to 0.2)
Kidney cancer	High SDI	52.4 (48.9 to 54.2)	63.3 (57.9 to 66.7)	2.1 (1.7 to 2.5)	3.3 (3.1 to 3.4)	3.3 (3.0 to 3.4)	-0.2 (-0.6 to 0.2)
Kidney cancer	High-middle SDI	45.0 (43.1 to 46.4)	53.7 (49.6 to 57.1)	2.0 (1.3 to 2.6)	2.8 (2.7 to 2.9)	2.7 (2.5 to 2.8)	-0.6 (-1.2 to 0.0)
Kidney cancer	Middle SDI	22.1 (20.9 to 23.6)	31.0 (27.6 to 35.5)	3.8 (2.7 to 4.9)	1.2 (1.1 to 1.3)	1.3 (1.1 to 1.5)	0.6 (-0.4 to 1.7)
Kidney cancer	Low-middle SDI	8.81 (8.14 to 9.45)	13.1 (11.8 to 14.5)	4.4 (3.2 to 5.8)	0.8 (0.8 to 0.9)	1.0 (0.9 to 1.1)	1.5 (0.3 to 2.9)
Kidney cancer	Low SDI	3.61 (3.01 to 4.17)	5.18 (4.47 to 5.94)	4.0 (2.6 to 5.4)	0.8 (0.7 to 0.9)	0.9 (0.8 to 1.0)	1.3 (-0.0 to 2.7)

Bladder cancer	Global	182 (171 to 189)	229 (211 to 243)	2.5 (2.0 to 3.1)	3.1 (2.9 to 3.2)	2.9 (2.7 to 3.1)	-0.6 (-1.1 to -0.0)
Bladder cancer	High SDI	61.8 (56.5 to 64.6)	77.0 (68.5 to 82.5)	2.4 (2.0 to 2.9)	3.7 (3.4 to 3.8)	3.6 (3.2 to 3.8)	-0.1 (-0.6 to 0.3)
Bladder cancer	High-middle SDI	59.4 (56.0 to 61.6)	69.9 (63.9 to 74.8)	1.8 (1.1 to 2.5)	3.8 (3.6 to 4.0)	3.5 (3.2 to 3.7)	-1.1 (-1.7 to -0.4)
Bladder cancer	Middle SDI	35.1 (32.8 to 37.1)	47.0 (41.9 to 53.6)	3.3 (2.1 to 4.4)	2.2 (2.0 to 2.3)	2.1 (1.9 to 2.4)	-0.3 (-1.3 to 0.8)
Bladder cancer	Low-middle SDI	16.7 (15.3 to 18.2)	22.9 (20.9 to 25.0)	3.5 (2.4 to 4.7)	1.9 (1.7 to 2.1)	1.9 (1.7 to 2.1)	-0.0 (-1.1 to 1.1)
Bladder cancer	Low SDI	8.91 (7.41 to 10.1)	11.8 (10.3 to 13.4)	3.1 (1.6 to 4.6)	2.8 (2.3 to 3.2)	2.7 (2.4 to 3.1)	-0.4 (-1.8 to 1.1)
Brain and central nervous system cancer	Global	206 (162 to 224)	246 (186 to 271)	2.0 (0.8 to 2.7)	3.1 (2.4 to 3.4)	3.0 (2.3 to 3.4)	-0.3 (-1.4 to 0.4)
Brain and central nervous system cancer	High SDI	47.2 (34.2 to 50.5)	54.8 (38.3 to 60.0)	1.7 (1.0 to 2.1)	3.5 (2.6 to 3.7)	3.4 (2.5 to 3.7)	-0.1 (-0.7 to 0.2)
Brain and central nervous system cancer	High-middle SDI	59.8 (45.4 to 64.5)	68.8 (49.1 to 77.1)	1.6 (0.4 to 2.5)	3.9 (2.9 to 4.2)	3.7 (2.7 to 4.1)	-0.5 (-1.7 to 0.4)
Brain and central nervous system cancer	Middle SDI	60.1 (46.3 to 65.3)	73.8 (53.5 to 83.9)	2.3 (0.9 to 3.4)	3.0 (2.3 to 3.3)	3.0 (2.2 to 3.4)	-0.2 (-1.6 to 0.9)
Brain and central nervous system cancer	Low-middle SDI	28.1 (22.7 to 34.0)	34.5 (27.3 to 40.1)	2.3 (0.7 to 3.4)	2.2 (1.8 to 2.7)	2.3 (1.8 to 2.6)	0.2 (-1.2 to 1.2)
Brain and central nervous system cancer	Low SDI	11.2 (8.28 to 15.3)	14.3 (10.6 to 17.5)	2.7 (0.7 to 4.4)	1.7 (1.3 to 2.2)	1.8 (1.3 to 2.1)	0.3 (-1.2 to 1.6)
Thyroid cancer	Global	37.3 (33.8 to 39.0)	45.6 (41.3 to 48.8)	2.2 (1.6 to 2.8)	0.6 (0.5 to 0.6)	0.6 (0.5 to 0.6)	-0.5 (-1.1 to 0.1)
Thyroid cancer	High SDI	8.28 (7.14 to 8.74)	9.38 (8.00 to 10.0)	1.4 (1.0 to 1.8)	0.5 (0.5 to 0.5)	0.5 (0.4 to 0.5)	-1.0 (-1.4 to -0.5)
Thyroid cancer	High-middle SDI	8.47 (7.81 to 8.82)	9.45 (8.56 to 10.2)	1.2 (0.5 to 2.0)	0.5 (0.5 to 0.6)	0.5 (0.4 to 0.5)	-1.3 (-2.0 to -0.5)
Thyroid cancer	Middle SDI	10.8 (9.92 to 11.5)	14.0 (12.5 to 15.5)	2.8 (1.9 to 3.7)	0.6 (0.6 to 0.7)	0.6 (0.5 to 0.7)	-0.4 (-1.3 to 0.5)
Thyroid cancer	Low-middle SDI	6.71 (5.96 to 7.29)	8.87 (7.80 to 9.84)	3.1 (2.1 to 4.1)	0.6 (0.6 to 0.7)	0.7 (0.6 to 0.7)	0.1 (-0.9 to 1.1)

Thyroid cancer	Low SDI	2.95 (2.39 to 3.37)	3.88 (3.18 to 4.50)	3.0 (2.1 to 4.0)	0.7 (0.6 to 0.8)	0.7 (0.6 to 0.8)	0.0 (-0.9 to 0.8)
Mesothelioma	Global	24.5 (22.5 to 25.9)	29.3 (26.7 to 31.0)	2.0 (1.6 to 2.3)	0.4 (0.4 to 0.4)	0.4 (0.3 to 0.4)	-0.8 (-1.2 to -0.5)
Mesothelioma	High SDI	12.5 (11.6 to 13.0)	14.8 (13.6 to 15.6)	1.9 (1.5 to 2.2)	0.8 (0.7 to 0.8)	0.7 (0.7 to 0.8)	-0.5 (-0.8 to -0.2)
Mesothelioma	High-middle SDI	5.81 (5.21 to 6.15)	6.43 (5.72 to 6.88)	1.1 (0.4 to 1.8)	0.4 (0.3 to 0.4)	0.3 (0.3 to 0.3)	-1.3 (-2.1 to -0.6)
Mesothelioma	Middle SDI	3.50 (3.13 to 3.80)	4.24 (3.83 to 4.65)	2.1 (1.1 to 3.0)	0.2 (0.2 to 0.2)	0.2 (0.2 to 0.2)	-0.8 (-1.8 to 0.1)
Mesothelioma	Low-middle SDI	2.03 (1.63 to 2.56)	2.86 (2.35 to 3.49)	3.8 (2.6 to 5.1)	0.2 (0.2 to 0.2)	0.2 (0.2 to 0.3)	0.8 (-0.4 to 2.0)
Mesothelioma	Low SDI	0.670 (0.380 to 1.27)	0.944 (0.549 to 1.78)	3.8 (2.2 to 5.3)	0.2 (0.1 to 0.3)	0.2 (0.1 to 0.3)	0.7 (-0.8 to 2.1)
Hodgkin lymphoma	Global	26.0 (22.6 to 30.3)	27.6 (23.7 to 31.8)	0.6 (0.0 to 1.2)	0.4 (0.3 to 0.4)	0.3 (0.3 to 0.4)	-1.3 (-1.9 to -0.7)
Hodgkin lymphoma	High SDI	3.64 (3.18 to 4.31)	3.88 (3.42 to 4.65)	0.7 (0.4 to 1.0)	0.3 (0.2 to 0.3)	0.2 (0.2 to 0.3)	-1.1 (-1.4 to -0.7)
Hodgkin lymphoma	High-middle SDI	5.85 (5.07 to 6.57)	5.57 (4.81 to 6.38)	-0.5 (-1.2 to 0.1)	0.4 (0.3 to 0.4)	0.3 (0.3 to 0.3)	-2.2 (-2.8 to -1.6)
Hodgkin lymphoma	Middle SDI	6.31 (5.37 to 7.42)	6.60 (5.53 to 7.67)	0.5 (-0.3 to 1.2)	0.3 (0.3 to 0.4)	0.3 (0.2 to 0.3)	-1.6 (-2.4 to -0.9)
Hodgkin lymphoma	Low-middle SDI	6.07 (5.28 to 7.87)	6.56 (5.57 to 8.37)	0.9 (-0.2 to 2.0)	0.5 (0.4 to 0.6)	0.4 (0.3 to 0.5)	-1.3 (-2.4 to -0.2)
Hodgkin lymphoma	Low SDI	4.14 (3.11 to 4.84)	4.91 (3.67 to 5.84)	1.9 (0.7 to 3.0)	0.7 (0.5 to 0.8)	0.6 (0.5 to 0.7)	-1.1 (-2.3 to -0.1)
Non-Hodgkin lymphoma	Global	204 (194 to 212)	255 (238 to 270)	2.5 (2.0 to 3.0)	3.3 (3.1 to 3.4)	3.2 (3.0 to 3.4)	-0.2 (-0.7 to 0.3)
Non-Hodgkin lymphoma	High SDI	70.7 (65.0 to 73.6)	84.5 (76.3 to 89.3)	2.0 (1.7 to 2.3)	4.5 (4.2 to 4.6)	4.3 (4.0 to 4.5)	-0.4 (-0.7 to -0.1)
Non-Hodgkin lymphoma	High-middle SDI	46.8 (44.4 to 48.6)	54.7 (50.5 to 58.5)	1.7 (1.0 to 2.4)	3.0 (2.8 to 3.1)	2.8 (2.6 to 3.0)	-0.6 (-1.4 to 0.1)
Non-Hodgkin lymphoma	Middle SDI	47.6 (45.3 to 50.4)	62.2 (56.9 to 68.3)	3.0 (2.1 to 3.9)	2.5 (2.4 to 2.6)	2.6 (2.3 to 2.8)	0.2 (-0.6 to 1.1)

Non-Hodgkin lymphoma	Low-middle SDI	28.0 (26.0 to 30.3)	38.2 (34.7 to 42.4)	3.5 (2.5 to 4.3)	2.5 (2.4 to 2.7)	2.7 (2.5 to 3.0)	0.8 (-0.2 to 1.7)
Non-Hodgkin lymphoma	Low SDI	11.0 (9.86 to 12.4)	14.8 (12.7 to 17.1)	3.3 (2.1 to 4.5)	2.4 (2.1 to 2.8)	2.5 (2.1 to 2.9)	0.5 (-0.5 to 1.3)
Multiple myeloma	Global	88.1 (77.5 to 94.1)	113 (99.5 to 122)	2.8 (2.2 to 3.3)	1.4 (1.3 to 1.5)	1.4 (1.2 to 1.5)	-0.2 (-0.8 to 0.3)
Multiple myeloma	High SDI	39.0 (33.8 to 41.1)	48.1 (41.3 to 51.2)	2.3 (2.0 to 2.6)	2.4 (2.1 to 2.5)	2.4 (2.1 to 2.6)	-0.0 (-0.4 to 0.3)
Multiple myeloma	High-middle SDI	21.8 (18.8 to 22.9)	27.2 (22.9 to 29.6)	2.5 (1.6 to 3.1)	1.4 (1.2 to 1.4)	1.3 (1.1 to 1.5)	-0.2 (-1.1 to 0.4)
Multiple myeloma	Middle SDI	15.1 (13.2 to 17.0)	21.1 (17.5 to 24.1)	3.7 (2.3 to 4.7)	0.8 (0.7 to 0.9)	0.9 (0.7 to 1.0)	0.4 (-1.0 to 1.4)
Multiple myeloma	Low-middle SDI	8.50 (7.41 to 9.95)	11.9 (10.2 to 14.0)	3.8 (2.7 to 4.8)	0.8 (0.7 to 1.0)	0.9 (0.8 to 1.0)	0.6 (-0.5 to 1.6)
Multiple myeloma	Low SDI	3.59 (2.84 to 4.36)	4.97 (3.94 to 5.77)	3.6 (2.3 to 4.7)	1.0 (0.8 to 1.2)	1.0 (0.8 to 1.2)	0.3 (-0.9 to 1.3)
Leukemia	Global	300 (275 to 318)	335 (307 to 360)	1.2 (0.5 to 1.9)	4.7 (4.3 to 5.0)	4.3 (3.9 to 4.6)	-1.0 (-1.7 to -0.4)
Leukemia	High SDI	72.8 (66.8 to 75.6)	85.3 (77.0 to 89.7)	1.7 (1.5 to 2.0)	4.9 (4.5 to 5.0)	4.6 (4.3 to 4.8)	-0.6 (-0.8 to -0.3)
Leukemia	High-middle SDI	71.1 (65.3 to 74.2)	75.8 (68.7 to 81.3)	0.7 (-0.0 to 1.4)	4.8 (4.4 to 5.0)	4.2 (3.8 to 4.5)	-1.4 (-2.1 to -0.7)
Leukemia	Middle SDI	84.6 (76.9 to 91.0)	92.2 (81.5 to 104)	1.0 (-0.0 to 2.0)	4.3 (3.9 to 4.7)	3.9 (3.4 to 4.4)	-1.2 (-2.1 to -0.2)
Leukemia	Low-middle SDI	44.9 (39.4 to 51.7)	51.1 (45.3 to 59.1)	1.4 (0.3 to 2.5)	3.6 (3.2 to 4.1)	3.5 (3.1 to 4.0)	-0.5 (-1.5 to 0.5)
Leukemia	Low SDI	26.0 (20.8 to 33.3)	29.9 (24.1 to 35.5)	1.6 (-0.3 to 3.4)	4.1 (3.3 to 4.8)	3.9 (3.1 to 4.6)	-0.5 (-1.7 to 0.6)
Acute lymphoid leukemia	Global	45.5 (38.1 to 52.1)	47.6 (39.4 to 53.0)	0.5 (-0.6 to 1.6)	0.7 (0.6 to 0.8)	0.6 (0.5 to 0.7)	-0.8 (-1.9 to 0.3)
Acute lymphoid leukemia	High SDI	4.90 (4.47 to 5.46)	5.32 (4.72 to 5.80)	0.9 (0.3 to 1.3)	0.4 (0.4 to 0.5)	0.4 (0.4 to 0.5)	-0.7 (-1.2 to -0.2)
Acute lymphoid leukemia	High-middle SDI	10.0 (8.73 to 10.7)	10.3 (8.57 to 11.3)	0.3 (-0.7 to 1.1)	0.8 (0.7 to 0.8)	0.7 (0.6 to 0.8)	-1.0 (-1.9 to -0.1)

Acute lymphoid leukemia	Middle SDI	16.3 (13.0 to 17.8)	17.1 (13.1 to 19.2)	0.5 (-0.6 to 1.6)	0.8 (0.6 to 0.9)	0.7 (0.6 to 0.8)	-0.7 (-1.8 to 0.4)
Acute lymphoid leukemia	Low-middle SDI	8.56 (7.04 to 10.7)	8.63 (7.37 to 10.4)	0.1 (-1.4 to 1.7)	0.5 (0.5 to 0.7)	0.5 (0.4 to 0.6)	-0.8 (-2.1 to 0.6)
Acute lymphoid leukemia	Low SDI	5.67 (3.98 to 8.35)	6.32 (4.70 to 8.00)	1.2 (-1.7 to 3.9)	0.5 (0.4 to 0.7)	0.5 (0.4 to 0.6)	-0.6 (-2.9 to 1.6)
Chronic lymphoid leukemia	Global	35.3 (32.4 to 39.1)	44.6 (40.4 to 50.1)	2.6 (2.1 to 3.1)	0.6 (0.5 to 0.7)	0.6 (0.5 to 0.6)	-0.5 (-1.0 to 0.0)
Chronic lymphoid leukemia	High SDI	13.3 (11.8 to 15.5)	15.3 (13.4 to 18.6)	1.6 (1.1 to 2.2)	0.8 (0.7 to 0.9)	0.7 (0.6 to 0.9)	-1.0 (-1.6 to -0.4)
Chronic lymphoid leukemia	High-middle SDI	10.2 (9.42 to 11.1)	11.9 (10.7 to 13.1)	1.7 (1.0 to 2.3)	0.7 (0.6 to 0.7)	0.6 (0.5 to 0.7)	-1.1 (-1.7 to -0.5)
Chronic lymphoid leukemia	Middle SDI	5.63 (5.09 to 6.34)	7.91 (7.00 to 9.11)	3.8 (2.7 to 4.9)	0.3 (0.3 to 0.4)	0.3 (0.3 to 0.4)	0.5 (-0.5 to 1.5)
Chronic lymphoid leukemia	Low-middle SDI	4.13 (3.66 to 4.80)	6.51 (5.60 to 7.61)	5.1 (3.6 to 6.5)	0.4 (0.4 to 0.5)	0.5 (0.4 to 0.6)	1.9 (0.4 to 3.3)
Chronic lymphoid leukemia	Low SDI	2.02 (1.65 to 2.38)	2.99 (2.48 to 3.52)	4.4 (3.2 to 5.5)	0.6 (0.5 to 0.7)	0.7 (0.6 to 0.8)	1.2 (0.1 to 2.3)
Acute myeloid leukemia	Global	77.6 (68.0 to 81.6)	94.1 (82.6 to 101)	2.1 (1.6 to 2.7)	1.2 (1.1 to 1.3)	1.2 (1.0 to 1.3)	-0.2 (-0.8 to 0.4)
Acute myeloid leukemia	High SDI	30.9 (25.0 to 32.6)	36.8 (29.8 to 39.5)	2.0 (1.6 to 2.3)	2.1 (1.7 to 2.2)	2.0 (1.7 to 2.2)	-0.2 (-0.6 to 0.1)
Acute myeloid leukemia	High-middle SDI	18.0 (15.1 to 18.9)	20.9 (17.4 to 22.6)	1.7 (1.0 to 2.3)	1.2 (1.0 to 1.2)	1.1 (1.0 to 1.2)	-0.4 (-1.0 to 0.2)
Acute myeloid leukemia	Middle SDI	15.3 (13.8 to 17.6)	18.9 (16.9 to 22.4)	2.4 (1.4 to 3.4)	0.8 (0.7 to 0.9)	0.8 (0.7 to 0.9)	0.3 (-0.7 to 1.3)
Acute myeloid leukemia	Low-middle SDI	9.05 (7.99 to 11.0)	11.6 (10.2 to 14.1)	2.7 (1.5 to 3.9)	0.7 (0.6 to 0.9)	0.8 (0.7 to 0.9)	0.7 (-0.4 to 1.8)
Acute myeloid leukemia	Low SDI	4.40 (3.27 to 5.51)	5.79 (4.53 to 7.00)	3.1 (1.3 to 4.9)	0.6 (0.5 to 0.8)	0.7 (0.5 to 0.8)	0.8 (-0.5 to 2.0)
Chronic myeloid leukemia	Global	28.0 (24.9 to 31.7)	29.9 (27.0 to 33.5)	0.8 (-0.4 to 1.7)	0.4 (0.4 to 0.5)	0.4 (0.3 to 0.4)	-1.5 (-2.6 to -0.6)
Chronic myeloid leukemia	High SDI	4.50 (4.09 to 5.23)	4.81 (4.29 to 5.63)	0.7 (0.3 to 1.2)	0.3 (0.3 to 0.3)	0.3 (0.2 to 0.3)	-1.5 (-2.0 to -1.0)

Chronic myeloid leukemia	High-middle SDI	5.27 (4.97 to 5.66)	4.97 (4.55 to 5.51)	-0.6 (-1.4 to 0.1)	0.3 (0.3 to 0.4)	0.3 (0.2 to 0.3)	-2.9 (-3.6 to -2.2)
Chronic myeloid leukemia	Middle SDI	6.01 (5.50 to 6.64)	6.65 (5.92 to 7.65)	1.1 (0.0 to 2.2)	0.3 (0.3 to 0.3)	0.3 (0.2 to 0.3)	-1.3 (-2.4 to -0.3)
Chronic myeloid leukemia	Low-middle SDI	6.96 (5.79 to 8.32)	7.96 (6.62 to 9.75)	1.5 (-0.0 to 2.9)	0.6 (0.5 to 0.7)	0.5 (0.4 to 0.6)	-0.8 (-2.2 to 0.5)
Chronic myeloid leukemia	Low SDI	5.19 (3.68 to 7.72)	5.51 (4.07 to 6.95)	0.7 (-2.1 to 3.1)	0.9 (0.7 to 1.1)	0.8 (0.6 to 1.0)	-1.3 (-3.2 to 0.3)
Other leukemia	Global	113 (99.0 to 127)	118 (103 to 132)	0.5 (-0.4 to 1.5)	1.8 (1.5 to 2.0)	1.5 (1.3 to 1.7)	-1.8 (-2.7 to -0.8)
Other leukemia	High SDI	19.3 (17.2 to 22.0)	23.0 (19.8 to 26.2)	1.9 (1.4 to 2.3)	1.2 (1.1 to 1.4)	1.2 (1.0 to 1.3)	-0.6 (-1.0 to -0.2)
Other leukemia	High-middle SDI	27.6 (24.0 to 29.5)	27.8 (23.9 to 31.0)	0.1 (-1.0 to 1.2)	1.8 (1.6 to 2.0)	1.5 (1.3 to 1.7)	-2.2 (-3.2 to -1.1)
Other leukemia	Middle SDI	41.4 (35.5 to 46.6)	41.7 (35.4 to 48.7)	0.1 (-1.2 to 1.4)	2.2 (1.9 to 2.4)	1.8 (1.5 to 2.1)	-2.2 (-3.4 to -0.9)
Other leukemia	Low-middle SDI	16.2 (12.9 to 19.9)	16.5 (14.1 to 19.7)	0.2 (-1.1 to 1.9)	1.4 (1.1 to 1.6)	1.1 (1.0 to 1.4)	-1.9 (-3.0 to -0.4)
Other leukemia	Low SDI	8.75 (5.60 to 11.6)	9.31 (6.73 to 11.7)	0.7 (-1.4 to 3.2)	1.4 (1.0 to 1.8)	1.2 (0.9 to 1.5)	-1.3 (-2.7 to 0.3)
Other malignant neoplasms	Global	338 (300 to 359)	408 (355 to 444)	2.1 (1.5 to 2.7)	5.3 (4.6 to 5.6)	5.1 (4.5 to 5.6)	-0.3 (-0.9 to 0.3)
Other malignant neoplasms	High SDI	64.8 (55.7 to 68.4)	73.6 (63.4 to 78.7)	1.4 (1.1 to 1.7)	4.4 (3.9 to 4.7)	4.2 (3.7 to 4.4)	-0.6 (-0.9 to -0.3)
Other malignant neoplasms	High-middle SDI	76.5 (68.4 to 80.6)	84.9 (75.5 to 91.6)	1.2 (0.4 to 1.9)	5.0 (4.5 to 5.2)	4.5 (4.0 to 4.8)	-1.1 (-1.8 to -0.4)
Other malignant neoplasms	Middle SDI	89.5 (77.3 to 95.3)	111 (93.9 to 123)	2.4 (1.5 to 3.2)	4.8 (4.1 to 5.1)	4.7 (4.0 to 5.2)	-0.3 (-1.2 to 0.6)
Other malignant neoplasms	Low-middle SDI	70.0 (57.2 to 77.9)	91.0 (73.3 to 105)	2.9 (1.9 to 3.9)	6.2 (4.9 to 7.0)	6.5 (5.1 to 7.5)	0.4 (-0.6 to 1.4)
Other malignant neoplasms	Low SDI	36.9 (32.6 to 41.4)	47.9 (41.5 to 54.5)	2.9 (1.8 to 4.0)	7.0 (6.0 to 7.8)	7.1 (6.0 to 8.0)	0.2 (-0.6 to 1.1)

Abbreviations: 95% UI, 95% uncertainty interval; NMSC, non-melanoma skin cancer; SDI, socio-demographic index.

Footnote: Socio-demographic index (SDI) quintiles do not sum exactly to the global total because several territories are included in the global total but not in any SDI quintile totals. “Total cancers” in this table excludes the “Other neoplasms” category of benign and in situ neoplasms.

eTable 18: Global age-standardized cancer estimates in 2019 and ranking among 22 level 2 categories of diseases and injuries in the Global Burden of Disease Study, overall and by quintile of Socio-demographic Index

	DALYs (Disability-Adjusted Life Years)		Deaths		YLLs (Years of Life Lost)		Incident cases		YLDs (Years Lived with Disability)	
	Age-standardized rate, per 100,000 (95% UI)	Cancer rank	Age-standardized rate, per 100,000 (95% UI)	Cancer rank	Age-standardized rate, per 100,000 (95% UI)	Cancer rank	Age-standardized rate, per 100,000 (95% UI)	Cancer rank	Age-standardized rate, per 100,000 (95% UI)	Cancer rank
Global	3 032.4 (2 843.3 - 3 207.9)	2	123.9 (115.7 - 131.2)	2	2 938.0 (2 756.6 - 3 109.1)	2	211.4 (195.4 - 226.8)	21	94.3 (69.4 - 121.7)	20
Low SDI	2 648.1 (2 363.1 - 2 951.2)	7	100.3 (89.6 - 110.9)	3	2 607.0 (2 329.3 - 2 903.7)	5	114.1 (102.1 - 126.2)	21	41.1 (29.7 - 54.0)	22
Low-middle SDI	2 680.4 (2 458.5 - 2 913.5)	4	101.7 (93.7 - 110.1)	3	2 632.4 (2 414.9 - 2 863.5)	4	127.2 (117.3 - 137.8)	21	48.0 (35.6 - 62.7)	22
Middle SDI	2 979.9 (2 721.8 - 3 240.9)	2	120.5 (109.3 - 131.4)	2	2 907.0 (2 648.7 - 3 168.8)	2	179.3 (162.7 - 196.1)	21	72.9 (53.7 - 96.3)	20
High-middle SDI	3 236.3 (2 988.5 - 3 468.4)	2	131.9 (120.7 - 141.7)	2	3 128.3 (2 891.9 - 3 359.8)	2	238.2 (217.7 - 258.3)	21	108.0 (78.9 - 141.0)	18
High SDI	2 991.9 (2 853.9 - 3 097.9)	1	130.2 (120.8 - 135.3)	1	2 829.5 (2 707.2 - 2 914.4)	1	316.7 (287.4 - 346.7)	20	162.4 (118.1 - 209.9)	14

Abbreviations: DALYs, disability-adjusted life years; YLLs, years of life lost; YLDs, years lived with disability; SDI, Socio-demographic Index; UI, 95% uncertainty interval.

Totals and rankings exclude non-melanoma skin cancer. All estimates refer to estimates in 2019. Rank refers to the relative ranking of the total cancer estimate for a given measure (e.g., DALYs) and SDI quintile (e.g., high SDI) compared among the 22 Level 2 categories of diseases and injuries in the Global Burden of Disease Study 2019. More details on SDI quintiles, including population, are in the eAppendix.

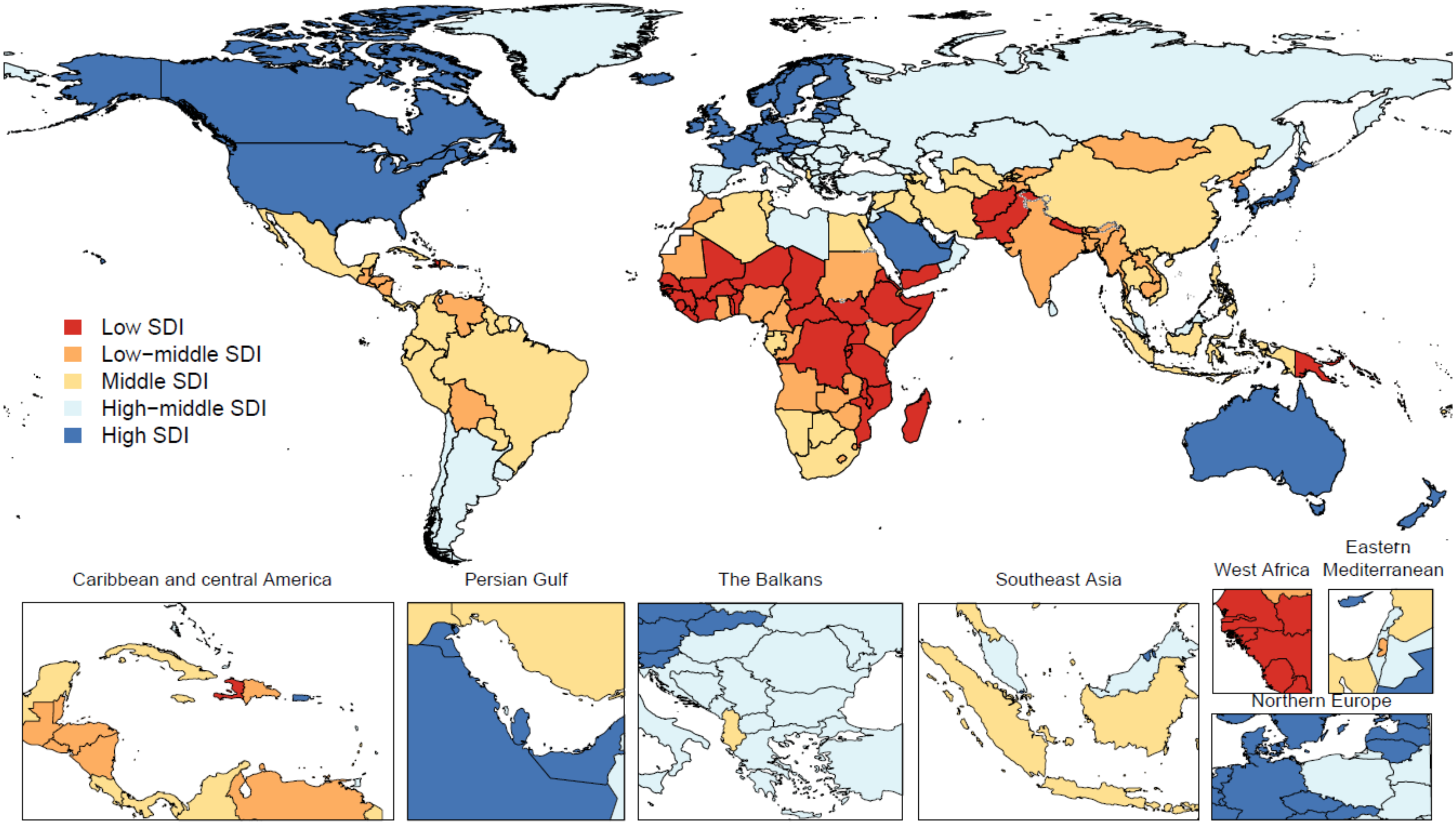

eFigure 1: Socio-demographic Index quintiles for the Global Burden of Disease Study 2019

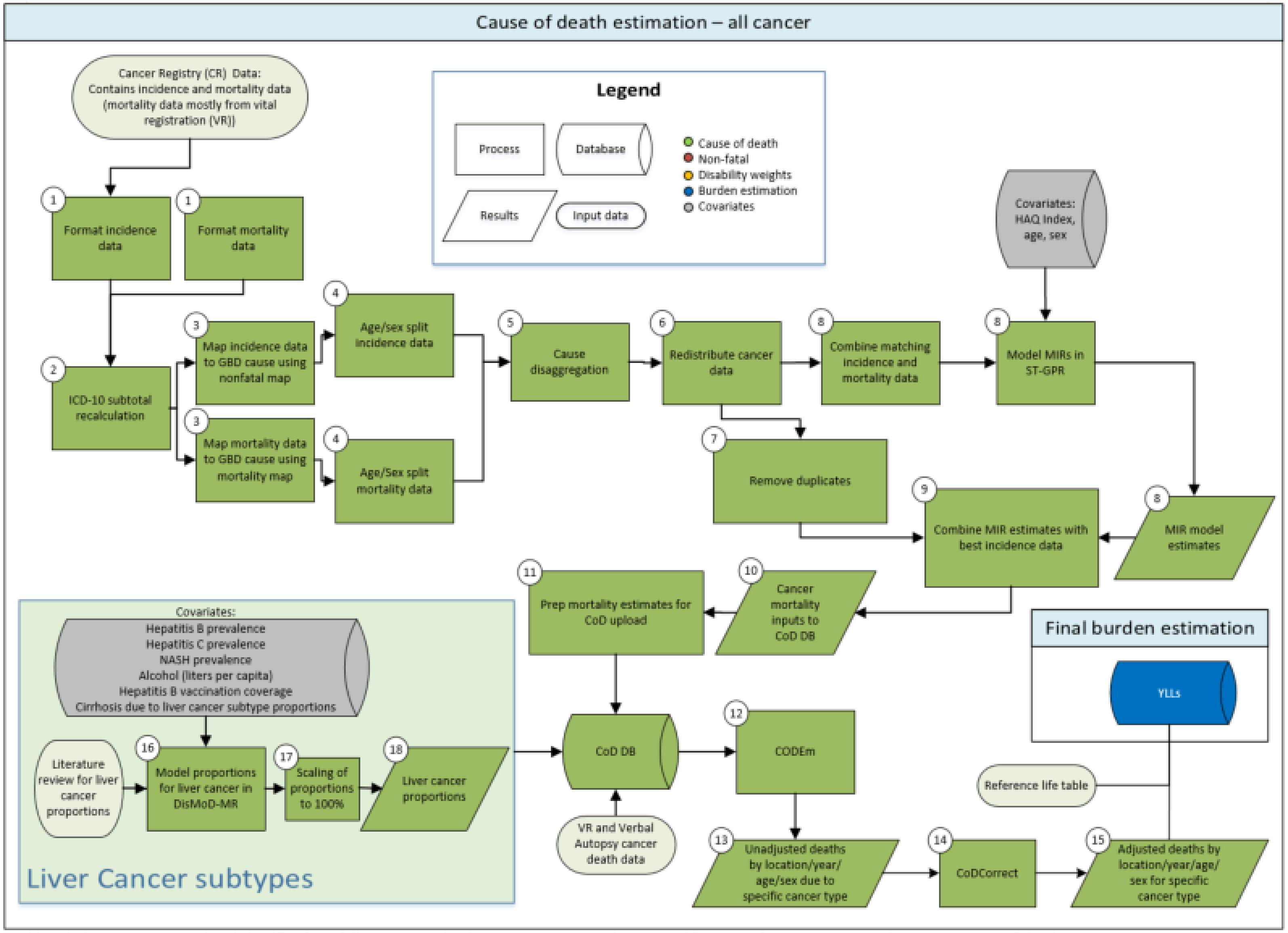

eFigure 2: Flowchart GBD cancer mortality, YLL estimation

Abbreviations: COD, causes of death; CODEm, cause of death ensemble model; DB, database; DisMod-MR, disease model - Bayesian meta-regression; HAQ Index, Healthcare Access and Quality Index; ICD, International Classifictaion of Diseases; ST-GPR, spaciotemporal Gaussian process regression; MIR, mortality to incidence ratio; NASH, nonalcoholic steatohepatitis; VR, vital registration; YLL, years of life lost.

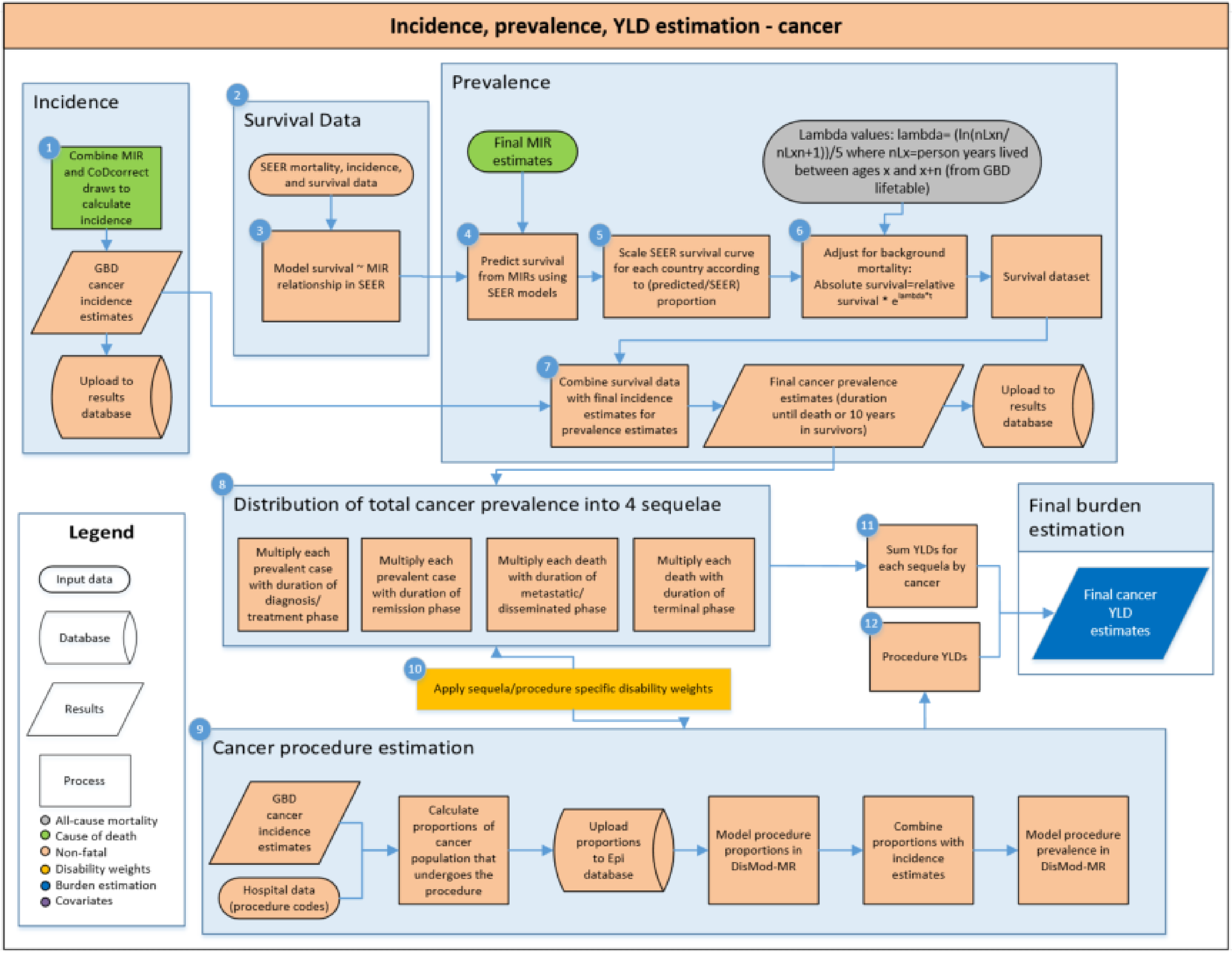

eFigure 3: Flowchart GBD cancer incidence, prevalence, YLD estimation

Abbreviations: GBD, Global Burden of Disease Study; MIR, mortality to incidence ratio; SEER, Surveillance, Epidemiology and End Results Program; YLD, years lived with disability.

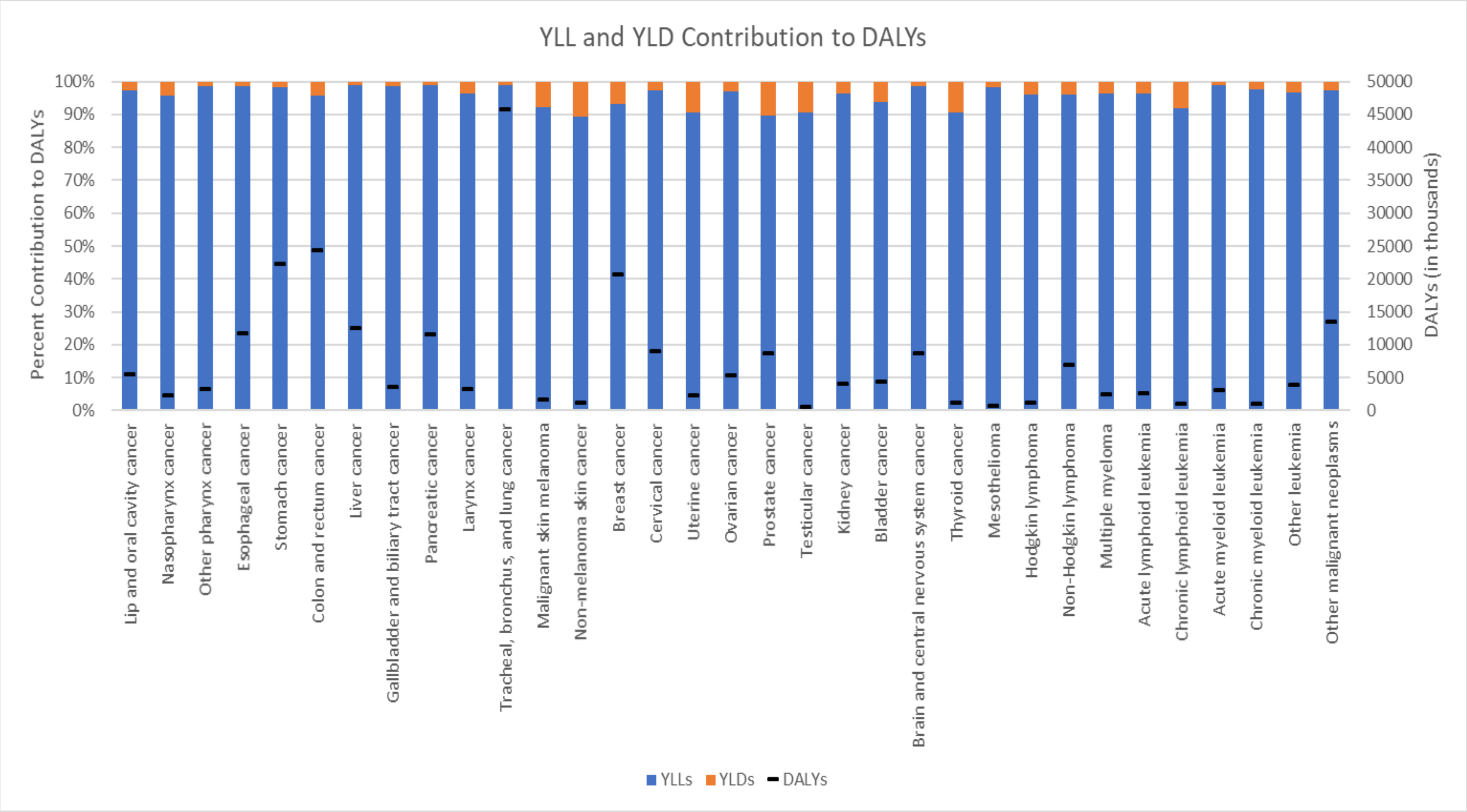

eFigure 4: Contribution of YLDs and YLLs to DALYs by cancer, global, both sexes, 2019

Abbreviations: YLL, years of life lost; YLD, years lived with disability; DALY, disability adjusted life year

Footnote: Black horizontal rectangle provides total DALYs, according to right-side y-axis. Blue shading provides the proportion of the total DALYs that are due to YLLs, according to left-side y-axis, with the orange shading the proportion due to YLDs.

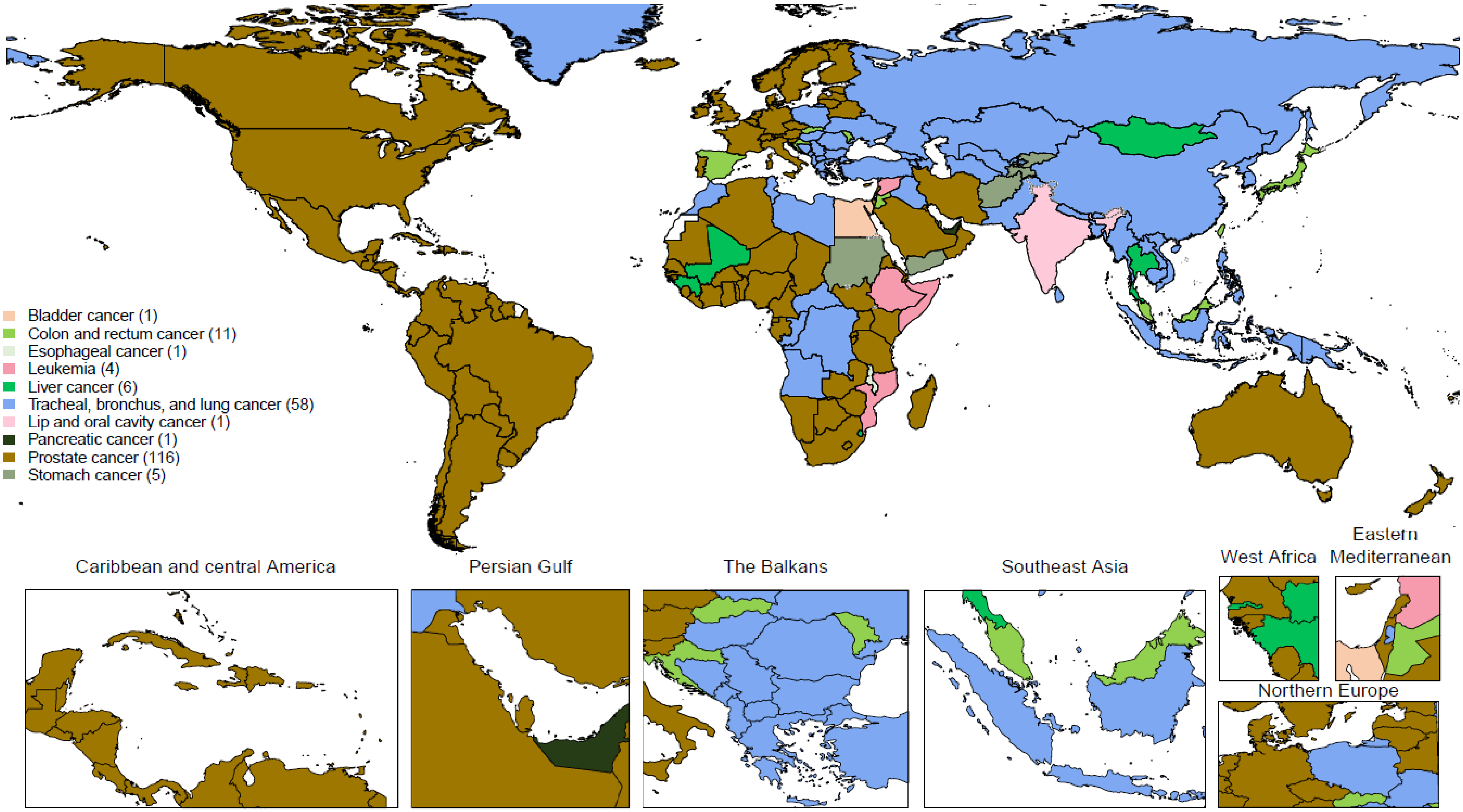

eFigure 5: Top-ranked cancers by absolute number of incident cases for all ages in males, 2019

Footnote: Number of countries and territories represented is in parentheses.

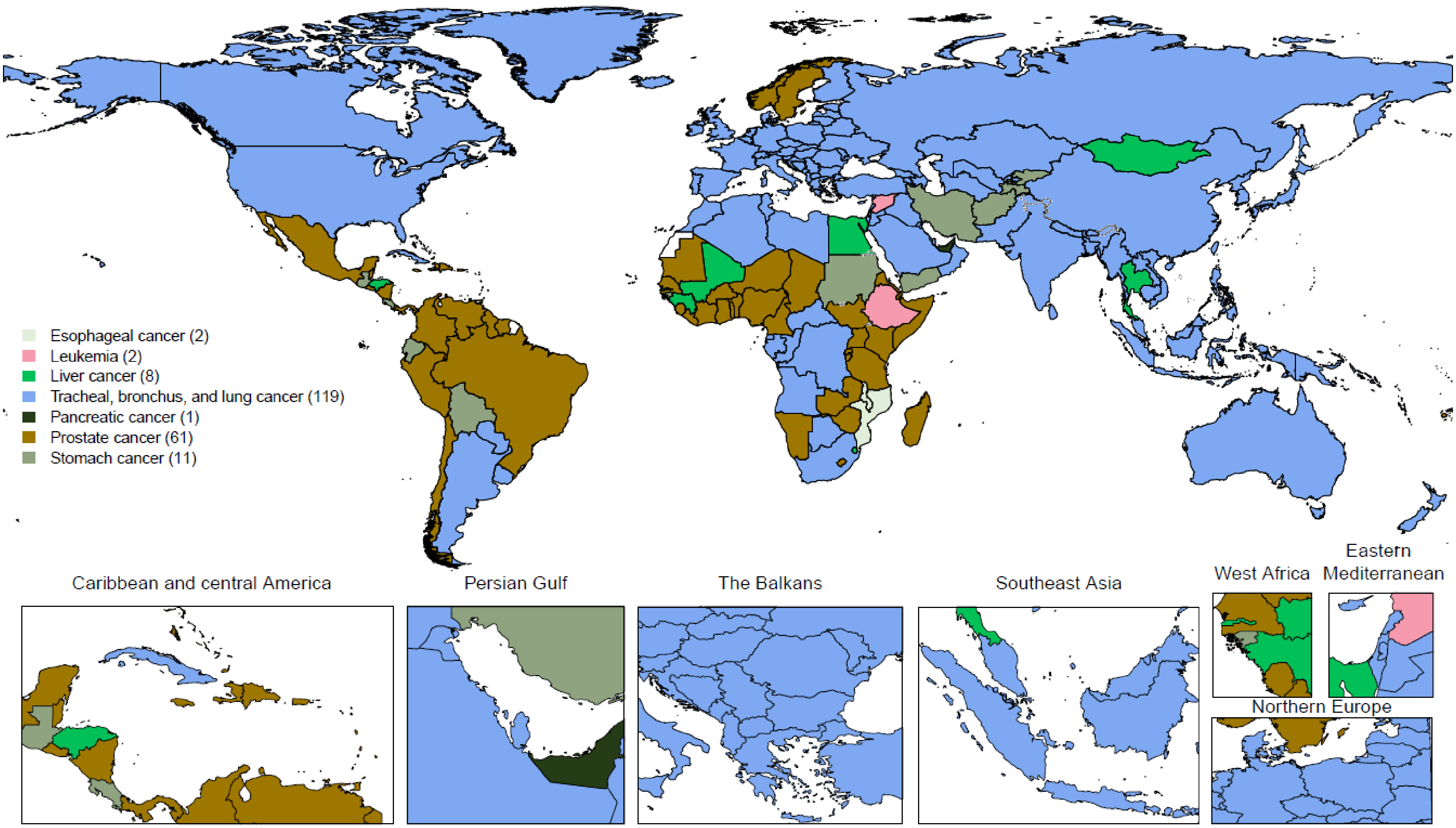

eFigure 6: Top-ranked cancers by absolute number of deaths for all ages in males, 2019

Footnote: Number of countries and territories represented is in parentheses.

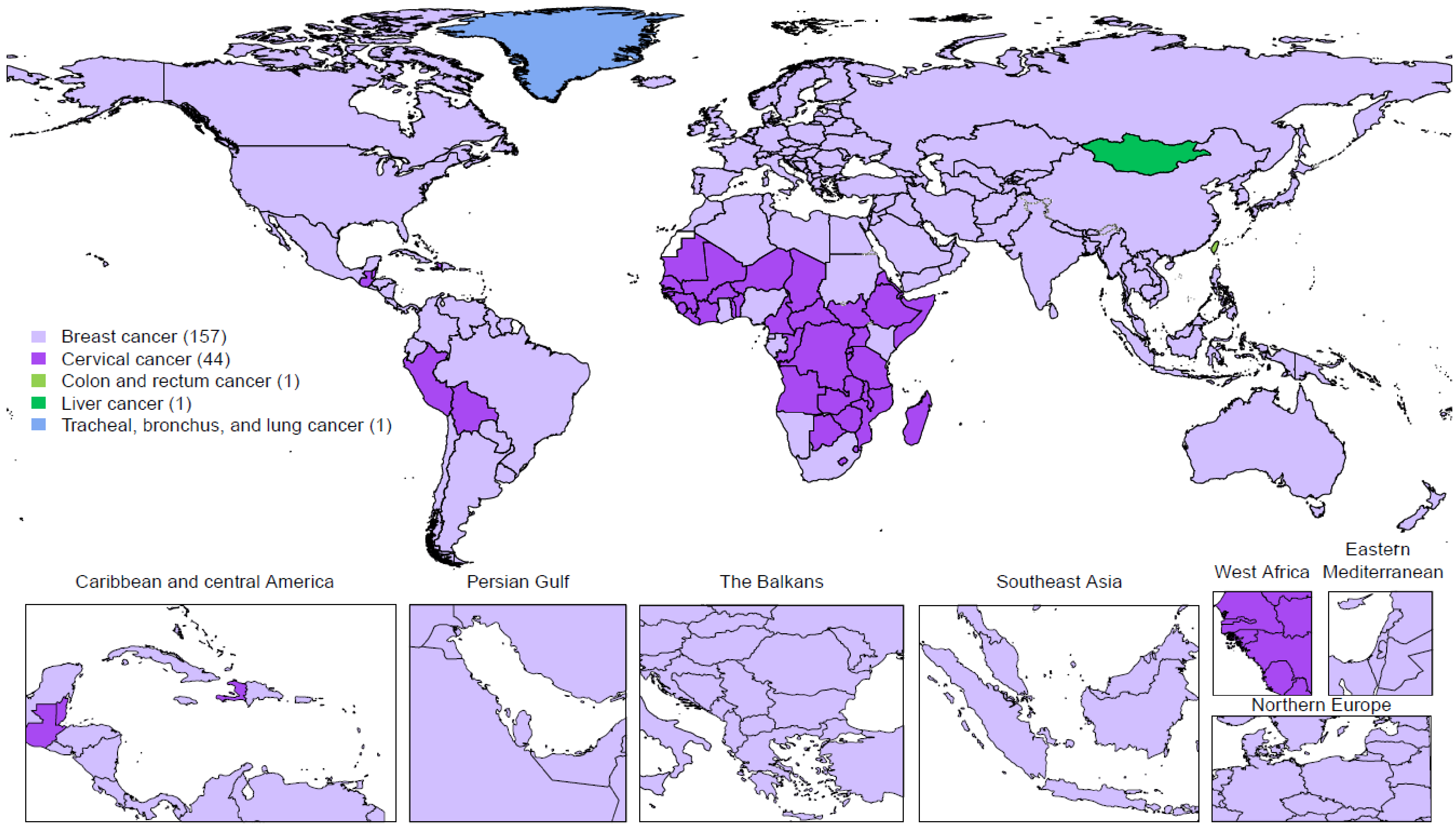

eFigure 7: Top-ranked cancers by absolute number of incident cases for all ages in females, 2019

Footnote: Number of countries and territories represented is in parentheses.

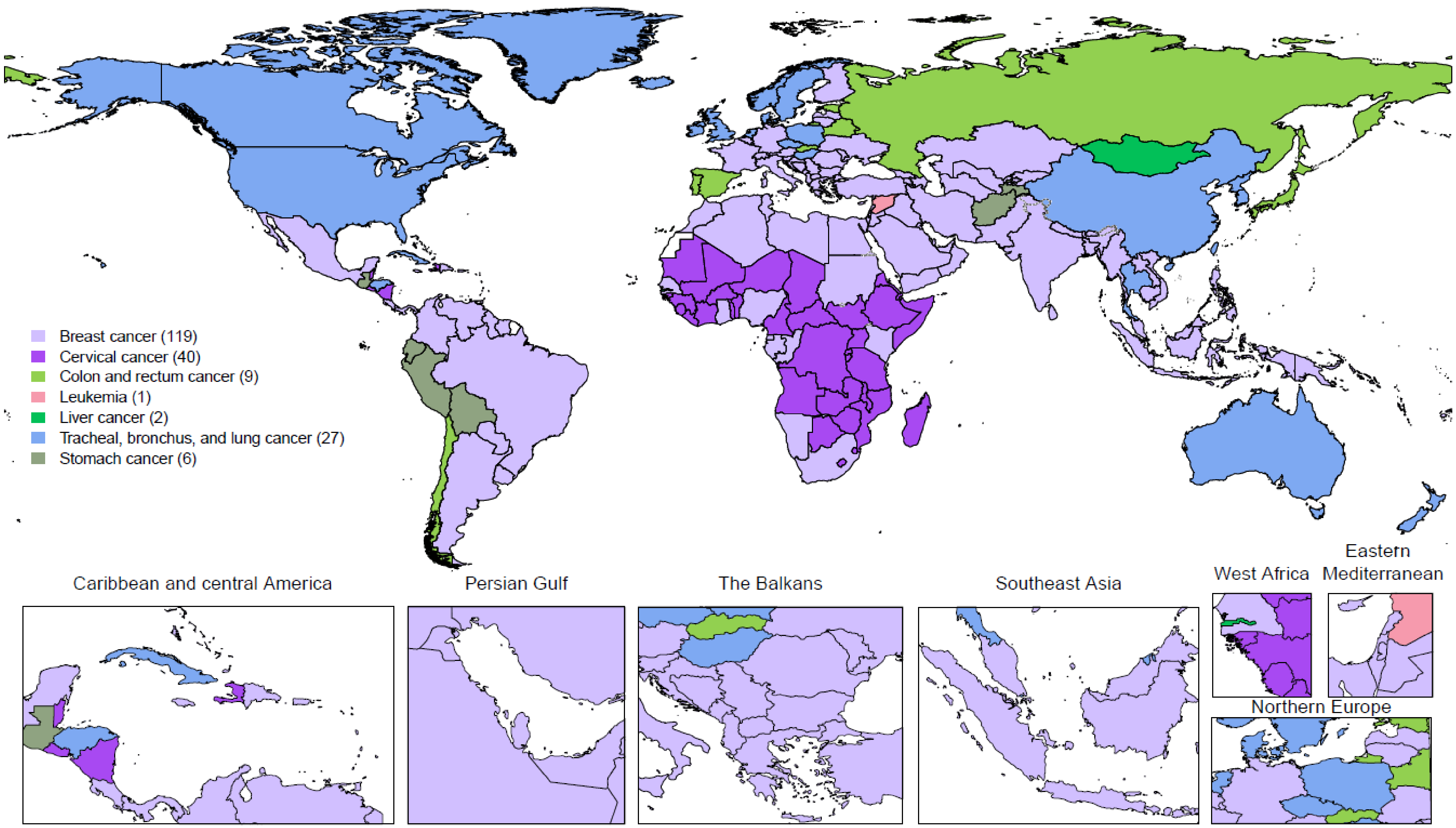

eFigure 8: Top-ranked cancers by absolute number of deaths for all ages in females, 2019

Footnote: Number of countries and territories represented is in parentheses.

Rank 2010	Rank 2019	Cancer	Absolute DALYs in 2019, millions (95% UI)	Percent change in absolute DALYs, 2010-2019 (95% UI)	Age-standardized DALY rate in 2019, per 100,000 (95% UI)	Percent change in age-standardized DALY rate, 2010-2019 (95% UI)
1	1	Tracheal, bronchus, and lung cancer	31.6 (28.6, 34.7)	15.1 (3.8, 27.7)	802.9 (727.6, 879.8)	-10.0 (-18.6, -0.4)
2	2	Stomach cancer	14.5 (12.9, 16.2)	-1.6 (-13.1, 11.2)	368.9 (328.2, 410.3)	-21.9 (-30.8, -12.0)
3	3	Colon and rectum cancer	14.0 (12.9, 15.0)	24.0 (14.4, 34.0)	360.1 (333.1, 387.8)	-2.2 (-9.6, 5.4)
5	4	Liver cancer	9.05 (8.02, 10.1)	22.2 (8.0, 37.9)	225.3 (200.4, 250.2)	-1.2 (-12.3, 11.0)
4	5	Esophageal cancer	8.82 (7.63, 10.1)	6.9 (-7.5, 25.8)	221.4 (191.2, 252.2)	-15.6 (-26.8, -0.9)
6	6	Prostate cancer	8.64 (7.55, 10.6)	26.2 (20.4, 32.8)	244.1 (211.8, 297.7)	-5.3 (-9.6, -0.6)
7	7	Leukemia	6.67 (5.70, 7.46)	1.9 (-7.4, 12.7)	175.4 (149.6, 196.5)	-10.8 (-18.6, -1.5)
8	8	Pancreatic cancer	6.48 (5.98, 7.00)	30.7 (20.6, 41.5)	164.7 (151.9, 177.4)	2.9 (-4.9, 11.1)
9	9	Brain and central nervous system cancer	5.01 (3.66, 5.74)	10.5 (-3.2, 23.7)	127.6 (92.9, 146.2)	-4.6 (-16.0, 6.4)
10	10	Non-Hodgkin lymphoma	4.22 (3.93, 4.53)	16.6 (8.5, 24.9)	108.2 (100.9, 116.2)	-3.3 (-9.7, 3.3)
11	11	Lip and oral cavity cancer	3.76 (3.33, 4.18)	18.2 (4.8, 31.9)	92.8 (82.4, 103.1)	-4.1 (-15.0, 6.7)
12	12	Bladder cancer	3.33 (3.09, 3.57)	22.1 (14.2, 30.2)	90.2 (83.6, 96.6)	-6.1 (-11.7, -0.2)
13	13	Larynx cancer	2.80 (2.59, 3.03)	13.6 (4.8, 22.3)	69.3 (64.1, 75.2)	-10.0 (-16.9, -3.2)
14	14	Kidney cancer	2.74 (2.54, 2.93)	22.3 (14.6, 30.5)	70.1 (65.1, 75.1)	-2.1 (-8.2, 4.4)
15	15	Other pharynx cancer	2.50 (2.22, 2.82)	25.5 (9.9, 40.5)	61.2 (54.2, 68.8)	1.0 (-11.4, 13.1)
16	16	Nasopharynx cancer	1.68 (1.51, 1.86)	13.7 (0.4, 28.3)	41.1 (37.0, 45.4)	-5.7 (-16.7, 6.2)
17	17	Gallbladder and biliary tract cancer	1.58 (1.30, 1.76)	17.2 (6.8, 30.7)	41.0 (33.7, 45.3)	-8.2 (-16.2, 1.9)
18	18	Multiple myeloma	1.38 (1.15, 1.57)	27.5 (17.9, 35.8)	35.5 (29.8, 40.0)	-0.4 (-7.6, 5.7)
19	19	Malignant skin melanoma	0.974 (0.642, 1.21)	13.0 (4.7, 22.3)	24.8 (16.2, 30.8)	-8.1 (-14.9, -0.3)
21	20	Non-melanoma skin cancer	0.743 (0.682, 0.802)	26.9 (18.7, 34.5)	20.0 (18.4, 21.6)	-0.9 (-7.0, 4.8)
20	21	Hodgkin lymphoma	0.726 (0.579, 0.894)	0.6 (-6.1, 7.7)	18.4 (14.7, 22.6)	-11.2 (-17.1, -5.0)
22	22	Testicular cancer	0.562 (0.511, 0.628)	14.7 (6.3, 23.7)	14.2 (12.9, 16.0)	4.1 (-3.3, 12.3)
23	23	Thyroid cancer	0.510 (0.462, 0.553)	21.2 (10.3, 31.4)	12.9 (11.7, 14.0)	-1.6 (-10.3, 6.6)
24	24	Mesothelioma	0.471 (0.443, 0.510)	14.4 (8.8, 19.8)	12.2 (11.5, 13.1)	-9.7 (-13.8, -5.7)
25	25	Breast cancer	0.315 (0.279, 0.349)	17.8 (6.7, 29.2)	8.0 (7.0, 8.8)	-6.5 (-15.0, 2.3)

eFigure 9: Cancers ranked by disability-adjusted life years (DALYs) for males between 2010 and 2019

Abbreviations: DALY, disability-adjusted life year; UI, 95% uncertainty interval

Footnote: Rankings are by absolute DALYs, and exclude the "Other malignant neoplasms" cancer group. Cancers are ordered by rank in 2019, with lines connecting to rank in 2010. Absolute DALYs and age-standardized DALY rates for 2010 can be found online at https://vizhub.healthdata.org/gbd-compare/. Colors refer to the directional change in cancer rank from 2010 to 2019: red signifies an increase in rank; blue signifies no change in rank; and green signifies a decrease in rank.

Rank 2010	Rank 2019	Cancer	Absolute DALYs in 2019, millions (95% UI)	Percent change in absolute DALYs, 2010-2019 (95% UI)	Age-standardized DALY rate in 2019, per 100,000 (95% UI)	Percent change in age-standardized DALY rate, 2010-2019 (95% UI)
1	1	Breast cancer	20.3 (18.7, 21.9)	21.3 (13.1, 29.6)	473.8 (437.3, 510.5)	-1.0 (-7.6, 5.8)
2	2	Tracheal, bronchus, and lung cancer	14.3 (13.0, 15.7)	25.5 (14.5, 37.0)	327.6 (298.5, 360.4)	-1.3 (-10.0, 7.7)
3	3	Colon and rectum cancer	10.3 (9.49, 11.1)	22.1 (14.0, 30.6)	237.9 (218.7, 257.1)	-3.5 (-9.9, 3.2)
4	4	Cervical cancer	8.96 (7.55, 9.98)	14.6 (5.0, 25.0)	210.6 (177.7, 234.9)	-4.8 (-12.8, 3.7)
5	5	Stomach cancer	7.70 (6.93, 8.50)	1.7 (-8.1, 12.5)	178.2 (160.5, 196.9)	-18.7 (-26.6, -10.1)
6	6	Ovarian cancer	5.36 (4.69, 5.95)	27.5 (16.3, 37.7)	124.7 (109.1, 138.7)	2.9 (-6.2, 11.1)
7	7	Pancreatic cancer	5.06 (4.63, 5.46)	35.7 (26.5, 44.4)	116.0 (106.0, 125.1)	5.9 (-1.3, 12.7)
8	8	Leukemia	4.99 (4.52, 5.44)	-0.6 (-9.9, 10.0)	127.2 (115.0, 138.9)	-13.4 (-21.9, -4.3)
9	9	Brain and central nervous system cancer	3.65 (2.70, 4.10)	13.2 (-2.8, 26.9)	90.9 (67.8, 102.0)	-3.1 (-17.1, 8.9)
10	10	Liver cancer	3.48 (3.11, 3.87)	17.7 (5.8, 31.0)	81.3 (72.7, 90.3)	-5.6 (-15.3, 5.0)
11	11	Esophageal cancer	2.84 (2.43, 3.20)	3.5 (-8.8, 21.5)	65.3 (55.8, 73.3)	-18.7 (-28.3, -4.0)
12	12	Non-Hodgkin lymphoma	2.77 (2.56, 2.98)	20.7 (13.3, 28.6)	66.1 (61.1, 71.2)	-0.7 (-6.8, 5.7)
13	13	Uterine cancer	2.33 (2.09, 2.56)	11.6 (3.5, 26.1)	53.5 (48.1, 58.8)	-11.7 (-18.2, -0.7)
14	14	Gallbladder and biliary tract cancer	2.04 (1.69, 2.35)	17.6 (7.8, 29.7)	46.8 (38.7, 54.0)	-8.0 (-15.6, 1.5)
15	15	Lip and oral cavity cancer	1.75 (1.56, 1.95)	31.9 (18.0, 46.6)	40.8 (36.5, 45.6)	6.7 (-4.4, 18.7)
16	16	Kidney cancer	1.32 (1.23, 1.41)	18.7 (12.4, 25.0)	31.1 (29.0, 33.4)	-4.7 (-9.8, 0.4)
17	17	Multiple myeloma	1.12 (0.968, 1.24)	24.7 (16.3, 32.1)	25.7 (22.1, 28.5)	-2.6 (-9.3, 3.2)
18	18	Bladder cancer	1.07 (0.963, 1.15)	20.2 (11.7, 29.7)	24.4 (22.1, 26.4)	-6.5 (-13.1, 0.8)
19	19	Malignant skin melanoma	0.734 (0.525, 0.882)	11.0 (2.4, 18.0)	17.3 (12.4, 20.8)	-9.1 (-16.1, -3.4)
20	20	Other pharynx cancer	0.731 (0.627, 0.857)	29.7 (9.8, 51.8)	17.0 (14.6, 19.9)	5.0 (-11.0, 22.7)
21	21	Thyroid cancer	0.722 (0.628, 0.792)	15.5 (7.6, 23.6)	16.9 (14.7, 18.6)	-5.9 (-12.5, 0.5)
22	22	Nasopharynx cancer	0.651 (0.580, 0.726)	10.3 (-1.8, 23.3)	15.4 (13.7, 17.2)	-8.2 (-18.4, 2.6)
23	23	Larynx cancer	0.464 (0.421, 0.512)	18.6 (8.4, 30.2)	10.7 (9.7, 11.8)	-5.2 (-13.3, 4.0)
24	24	Non-melanoma skin cancer	0.441 (0.387, 0.486)	20.2 (8.7, 32.2)	10.2 (8.9, 11.2)	-5.7 (-14.6, 3.5)
25	25	Hodgkin lymphoma	0.420 (0.329, 0.508)	5.3 (-2.1, 14.2)	10.5 (8.2, 12.7)	-6.5 (-13.3, 1.4)
26	26	Mesothelioma	0.197 (0.144, 0.225)	15.6 (5.2, 27.5)	4.6 (3.4, 5.3)	-6.4 (-14.8, 3.5)

eFigure 10: Cancers ranked by disability-adjusted life years (DALYs) for females between 2010 and 2019

Abbreviations: DALY, disability-adjusted life year; UI, 95% uncertainty interval

Footnote: Rankings are by absolute DALYs, and exclude the “Other malignant neoplasms” cancer group. Cancers are ordered by rank in 2019, with lines connecting to rank in 2010. Absolute DALYs and age-standardized DALY rates for 2010 can be found online at https://vizhub.healthdata.org/gbd-compare/. Colors refer to the directional change in cancer rank from 2010 to 2019: red signifies an increase in rank; blue signifies no change in rank; and green signifies a decrease in rank.

Location/grouping: *Global,* SDI quintile, ***Super Region,*** **Region,** Country or Territory	Tracheal, bronchus, and lung cancer	Colon and rectum cancer	Stomach cancer	Breast cancer	Pancreatic cancer	Esophageal cancer	Prostate cancer	Liver cancer	Other malignant neoplasms	Leukemia	Cervical cancer	Non-Hodgkin lymphoma	Brain and central nervous system cancer	Bladder cancer	Lip and oral cavity cancer	Ovarian cancer	Gallbladder and biliary tract cancer	Kidney cancer	Larynx cancer	Other pharynx cancer	Multiple myeloma	Uterine cancer	Nasopharynx cancer	Malignant skin melanoma	Non-melanoma skin cancer	Thyroid cancer	Mesothelioma	Hodgkin lymphoma	Testicular cancer
Global	1	2	3	4	5	6	7	8	9	10	11	12	13	14	15	16	17	18	19	20	21	22	23	24	25	26	27	28	29
High SDI	1	2	6	4	3	10	5	7	12	8	21	9	15	11	19	14	16	13	24	22	17	20	27	18	25	26	23	28	29
High-middle SDI	1	2	3	4	5	6	7	8	9	10	16	13	12	11	18	15	17	14	19	22	20	21	24	23	25	26	27	28	29
Middle SDI	1	3	2	6	7	5	8	4	9	10	11	13	12	16	14	15	17	19	18	21	22	23	20	26	24	25	28	27	29
Low-middle SDI	1	4	2	3	12	8	7	10	5	11	6	14	15	19	9	17	18	21	16	13	23	22	20	27	25	24	28	26	29
Low SDI	3	7	5	1	14	9	6	10	2	8	4	12	13	15	11	16	19	22	17	18	23	21	20	26	27	25	29	24	28
Central Europe, Eastern Europe, and Central Asia	1	2	3	4	5	14	6	13	7	10	15	17	9	11	16	12	20	8	19	22	23	18	27	21	24	25	28	26	29
**Central Asia**	1	4	2	3	7	6	12	5	8	11	10	16	9	15	17	14	20	13	19	21	22	18	26	23	24	27	29	25	28
Armenia	1	2	4	3	5	17	7	6	10	12	11	18	8	9	20	13	19	14	15	26	22	16	27	25	21	24	23	28	29
Azerbaijan	1	3	2	4	6	5	9	11	10	8	13	17	7	15	20	16	19	12	14	26	22	18	27	25	23	24	29	21	28
Georgia	1	4	3	2	6	19	5	12	7	10	11	17	9	8	18	13	21	16	14	23	25	15	29	20	22	27	28	24	26
Kazakhstan	1	3	2	4	6	5	12	7	8	11	9	19	14	16	15	10	20	13	18	22	23	17	27	21	24	25	28	26	29
Kyrgyzstan	2	3	1	4	6	8	13	12	7	9	5	17	10	18	15	11	19	14	21	22	24	16	25	23	20	26	29	27	28
Mongolia	3	5	2	8	9	4	17	1	7	11	6	13	10	20	16	14	12	15	18	23	21	19	24	28	25	22	27	26	29
Tajikistan	2	4	1	3	9	6	11	10	7	8	12	13	5	17	19	15	23	14	18	21	20	16	22	25	24	28	27	26	29
Turkmenistan	1	5	3	4	12	2	13	7	6	11	9	16	8	17	14	15	21	10	19	18	20	24	27	25	23	28	29	22	26
Uzbekistan	2	4	1	3	11	7	16	6	8	10	9	12	5	19	13	14	24	15	17	20	23	18	21	26	25	28	29	22	27
**Central Europe**	1	2	5	3	4	17	6	13	8	11	14	15	9	7	18	12	16	10	19	23	22	20	26	21	24	25	28	27	29
Albania	1	4	2	8	7	15	3	5	6	10	13	14	9	19	17	16	18	11	12	24	22	20	26	23	21	25	29	27	28
Bosnia and Herzegovina	1	2	4	3	5	18	7	6	10	12	15	17	8	9	20	11	14	13	16	24	22	19	28	21	23	26	29	25	27

Bulgaria	1	2	5	3	4	19	6	9	10	11	13	17	7	8	18	12	20	14	15	23	24	16	27	21	22	25	29	26	28
Croatia	1	2	5	3	6	16	4	14	11	9	22	12	7	8	21	13	15	10	19	23	18	20	28	17	24	26	25	27	29
Czechia	1	2	7	4	3	16	5	15	10	8	19	14	13	9	21	12	11	6	23	22	17	18	28	20	24	25	27	26	29
Hungary	1	2	5	4	3	15	6	18	10	8	20	16	14	7	12	11	13	9	19	17	23	22	26	21	24	25	29	28	27
Montenegro	1	2	6	3	4	15	5	8	14	11	16	17	7	10	18	13	20	12	9	23	22	19	29	21	26	25	28	24	27
North Macedonia	1	2	3	4	5	20	6	7	9	11	14	19	8	10	21	13	18	17	12	24	23	16	28	15	22	26	29	25	27
Poland	1	2	5	3	4	14	6	22	11	9	16	15	12	7	17	10	13	8	18	23	20	19	28	21	24	25	26	27	29
Romania	1	2	3	4	5	18	6	15	7	11	8	19	10	9	13	12	21	14	16	17	24	20	25	23	22	26	29	27	28
Serbia	1	2	6	3	4	19	5	9	10	11	13	15	7	8	18	12	17	14	16	23	22	20	27	21	24	25	29	26	28
Slovakia	1	2	5	3	4	18	6	15	8	11	20	12	10	9	17	14	13	7	23	16	21	19	27	22	24	25	29	26	28
Slovenia	1	2	6	4	5	18	3	7	12	10	22	9	14	8	21	13	15	11	23	19	16	20	28	17	24	26	25	27	29
**Eastern Europe**	1	2	3	4	5	13	6	15	7	10	14	18	12	11	16	9	22	8	19	21	23	17	27	20	24	25	28	26	29
Belarus	1	2	3	4	5	12	6	16	7	9	11	18	14	15	13	10	23	8	17	19	21	22	28	20	24	25	27	26	29
Estonia	1	2	3	6	5	15	4	12	9	8	16	13	14	11	20	10	21	7	23	22	17	18	27	19	24	25	28	26	29
Latvia	1	2	3	4	5	13	6	16	11	10	17	15	12	7	18	9	24	8	20	22	19	14	27	21	23	25	28	26	29
Lithuania	1	2	3	5	6	13	4	14	11	9	15	16	12	10	18	8	23	7	20	21	19	17	27	22	24	25	28	26	29
Republic of Moldova	1	2	3	4	5	19	6	16	7	15	8	18	10	9	17	13	24	11	14	12	22	20	25	21	23	27	29	26	28
Russian Federation	1	2	3	4	5	10	6	15	7	11	14	18	13	12	17	9	22	8	19	21	23	16	28	20	24	25	27	26	29
Ukraine	1	2	3	4	5	15	6	16	7	9	14	19	10	12	13	11	22	8	18	20	23	17	27	21	24	26	28	25	29
High-income	1	2	6	4	3	12	5	7	11	8	20	10	15	9	19	14	16	13	22	24	17	21	27	18	25	26	23	28	29
**Australasia**	1	2	9	4	5	10	3	11	12	6	23	7	13	15	18	17	22	14	25	24	16	21	27	8	19	26	20	28	29
Australia	1	2	11	4	5	9	3	8	12	6	23	7	13	15	18	17	21	14	25	24	16	22	27	10	20	26	19	28	29
New Zealand	1	2	8	4	5	11	3	12	10	7	23	9	13	15	22	17	21	14	26	25	16	19	28	6	18	24	20	27	29
**High-income Asia-Pacific**	1	2	3	7	5	9	8	4	11	13	17	10	19	12	18	15	6	14	25	21	16	20	26	27	23	22	24	28	29
Brunei Darussalam	1	2	4	3	7	19	13	5	8	9	10	6	12	20	16	11	17	15	24	21	18	22	14	25	27	23	28	26	29
Japan	1	2	3	8	4	9	7	5	11	13	18	10	19	12	17	15	6	14	25	21	16	20	26	27	24	22	23	28	29
Republic of Korea	1	4	3	7	5	11	8	2	9	12	17	10	15	13	19	16	6	14	21	22	18	24	26	25	23	20	27	28	29
Singapore	1	2	6	4	5	14	7	3	8	10	15	9	17	16	21	11	18	12	23	22	20	19	13	27	26	24	25	28	29
**High-income North America**	1	2	13	4	3	10	5	8	11	6	20	7	12	9	19	15	21	14	22	25	16	18	28	17	23	26	24	27	29
Canada	1	2	8	3	5	11	4	10	13	7	21	6	12	9	18	15	20	14	24	25	16	19	28	17	23	26	22	27	29
Greenland	1	2	5	6	3	4	10	7	9	19	15	14	13	16	17	12	21	8	22	18	20	25	11	23	27	26	24	28	29
United States of America	1	2	15	4	3	10	5	8	11	6	19	7	12	9	20	14	21	13	22	25	16	18	28	17	23	26	24	27	29
**Southern Latin America**	1	2	5	3	6	11	4	17	8	12	10	13	16	14	20	15	7	9	19	26	18	21	29	22	23	24	28	27	25
Argentina	1	2	6	3	4	10	5	17	8	11	7	12	15	14	20	16	13	9	18	26	19	21	29	22	23	24	27	28	25

Chile	2	3	1	7	6	10	4	14	9	12	11	13	18	15	22	17	5	8	23	26	16	20	29	21	19	24	27	28	25
Uruguay	1	2	6	3	5	8	4	19	12	11	14	10	16	9	20	15	13	7	18	23	17	22	27	21	24	25	29	26	28
**Western Europe**	1	2	6	3	5	12	4	10	9	8	21	11	14	7	19	15	17	13	23	24	16	20	28	18	25	26	22	27	29
Andorra	1	2	7	6	3	14	4	5	10	9	18	11	8	12	20	17	16	13	23	25	15	21	28	22	24	26	19	27	29
Austria	1	2	6	4	3	16	5	8	9	7	20	11	14	10	19	13	18	12	23	22	15	21	28	17	24	26	25	27	29
Belgium	1	2	7	3	5	10	4	12	9	8	20	11	15	6	17	14	22	13	23	24	16	19	27	18	25	26	21	28	29
Cyprus	1	2	6	3	5	19	4	12	10	7	17	9	11	8	21	13	18	15	23	27	14	16	28	20	22	25	24	26	29
Denmark	1	2	9	4	5	8	3	15	11	7	20	14	10	6	18	12	21	13	24	22	16	19	28	17	25	26	23	27	29
Finland	1	2	6	5	3	16	4	9	8	12	21	7	13	14	20	11	17	10	26	25	15	19	28	18	24	23	22	27	29
France	1	2	9	3	5	13	4	6	10	8	21	11	15	7	17	14	24	12	22	20	16	19	27	18	25	26	23	28	29
Germany	1	2	6	3	4	14	5	12	7	8	21	11	13	9	19	15	17	10	23	20	16	22	28	18	26	25	24	27	29
Greece	1	2	6	3	5	20	4	12	10	8	19	15	9	7	21	13	17	11	16	28	14	18	26	22	23	25	27	24	29
Iceland	1	2	7	5	4	10	3	14	9	12	21	13	8	11	18	16	20	6	24	26	15	22	28	17	25	19	23	27	29
Ireland	1	2	7	3	5	6	4	15	14	9	20	8	11	12	19	10	22	13	23	24	16	18	28	17	21	26	25	27	29
Israel	1	2	7	3	4	17	5	15	11	6	19	8	10	9	22	12	21	14	23	27	13	18	28	16	20	24	26	25	29
Italy	1	2	5	3	4	17	6	9	10	8	23	11	14	7	19	13	15	12	21	25	16	20	28	18	24	26	22	27	29
Luxembourg	1	2	7	3	4	14	5	12	10	6	22	13	9	8	19	11	21	17	23	20	15	18	27	16	25	26	24	28	29
Malta	1	2	6	3	4	15	5	14	8	11	22	10	13	7	18	9	23	12	20	27	16	17	24	19	25	26	21	28	29
Monaco	1	2	11	4	3	13	6	10	14	9	19	5	16	7	23	12	24	15	17	27	8	22	29	18	26	21	20	25	28
Netherlands	1	2	6	3	5	7	4	16	11	9	22	10	15	8	21	13	19	12	23	24	14	20	28	17	25	26	18	27	29
Norway	1	2	6	5	4	16	3	17	8	15	19	12	11	7	21	10	20	9	26	25	13	18	28	14	23	24	22	27	29
Portugal	2	1	3	5	6	13	4	9	11	8	21	10	12	7	17	16	18	15	19	22	14	20	26	23	24	25	27	28	29
San Marino	1	2	3	6	5	19	4	13	9	10	21	7	11	8	18	17	14	16	20	27	12	28	26	15	25	22	23	24	29
Spain	1	2	6	4	5	16	3	8	10	9	21	11	12	7	18	14	19	13	20	23	15	17	27	22	24	26	25	28	29
Sweden	1	2	11	5	4	17	3	16	8	7	20	9	13	6	21	12	18	10	26	23	14	19	28	15	24	25	22	27	29
Switzerland	1	2	7	4	5	12	3	6	10	11	21	9	14	8	18	13	19	15	25	23	16	22	27	17	24	26	20	28	29
United Kingdom	1	2	7	4	5	6	3	13	12	10	21	9	15	8	20	11	22	14	25	24	16	19	28	18	23	26	17	27	29
Latin America and Caribbean	1	3	2	5	6	10	4	12	8	9	7	13	11	17	19	15	14	16	18	23	20	21	29	24	22	25	28	26	27
**Andean Latin America**	3	4	1	6	8	17	2	13	9	7	5	10	12	18	22	14	11	15	24	27	19	16	29	23	21	20	28	26	25
Bolivia (Plurnational State of)	3	5	1	6	11	16	2	12	9	7	4	10	13	17	23	15	8	18	24	27	19	14	29	21	22	20	28	25	26
Ecuador	4	3	1	6	8	17	2	11	9	7	5	10	13	18	22	14	12	15	24	27	20	16	28	23	21	19	29	25	26
Peru	2	4	1	6	7	17	3	13	10	8	5	9	12	18	22	14	11	15	24	27	19	16	29	23	20	21	28	26	25
**Caribbean**	1	3	5	4	7	10	2	12	8	9	6	11	18	15	16	19	21	20	14	23	17	13	25	26	22	24	28	27	29
Antigua and Barbuda	5	3	4	2	6	12	1	15	8	9	7	11	17	16	19	10	21	18	20	22	13	14	26	23	24	25	29	28	27

Bahamas	4	3	5	2	10	7	1	14	8	13	6	12	18	21	15	11	20	19	17	22	9	16	26	23	25	24	28	27	29
Barbados	5	2	4	3	6	11	1	16	10	12	7	9	19	15	18	14	20	17	21	22	8	13	24	25	26	23	28	27	29
Belize	2	4	5	6	7	11	1	10	8	9	3	14	12	16	20	18	19	15	17	23	22	13	27	26	21	28	25	24	29
Bermuda	1	3	6	4	5	8	2	17	11	12	20	9	15	7	16	10	24	14	21	22	13	18	27	19	23	25	26	28	29
Cuba	1	2	6	4	5	7	3	17	9	12	11	14	16	10	15	21	23	20	8	22	19	13	27	25	18	24	28	26	29
Dominica	3	5	2	4	6	12	1	14	8	11	7	9	22	13	15	21	19	16	17	20	10	18	25	26	23	24	28	27	29
Dominican Republic	2	3	5	4	10	15	1	8	7	9	6	13	22	21	12	23	14	16	17	18	20	11	25	26	19	24	28	27	29
Grenada	4	2	5	3	6	9	1	14	11	12	7	8	18	15	17	13	20	19	22	21	16	10	26	25	24	23	28	27	29
Guyana	5	3	6	2	7	14	1	12	8	10	4	13	18	17	16	9	19	15	20	23	21	11	28	26	24	22	27	25	29
Haiti	5	6	4	3	16	12	1	10	8	7	2	9	11	15	18	20	19	21	17	22	14	13	24	26	27	25	28	23	29
Jamaica	2	3	5	4	8	15	1	14	10	9	6	7	18	16	21	13	17	20	19	23	12	11	24	25	26	22	28	27	29
Puerto Rico	3	1	6	4	5	13	2	11	9	7	16	8	17	12	19	14	22	15	21	23	10	18	27	24	20	25	28	26	29
Saint Kitts and Nevis	4	2	5	3	6	10	1	11	8	13	7	9	19	16	18	12	21	17	20	23	14	15	25	27	22	24	26	29	28
Saint Lucia	2	5	4	3	6	10	1	17	9	12	7	8	19	13	15	14	22	20	18	21	11	16	25	23	27	24	29	26	28
Saint Vincent and the Grenadines	5	3	4	2	8	17	1	13	9	10	6	7	18	16	12	14	22	20	15	21	19	11	26	25	23	24	28	27	29
Suriname	3	2	7	4	6	18	1	13	8	10	5	9	11	15	17	12	20	16	22	24	14	19	21	27	26	23	29	25	28
Trinidad and Tobago	4	2	7	3	5	17	1	13	8	11	6	12	19	16	18	10	20	15	21	22	14	9	25	26	24	23	28	27	29
United States Virgin Islands	3	2	6	4	5	11	1	16	10	13	12	9	15	19	17	8	21	14	20	23	7	18	24	22	27	26	25	28	29
**Central Latin America**	2	3	1	5	7	16	4	10	9	8	6	11	13	17	21	12	15	14	20	27	18	22	29	24	19	23	28	25	26
Colombia	2	3	1	5	6	15	4	11	9	8	7	10	13	17	21	12	14	16	20	27	18	23	29	22	19	24	28	25	26
Costa Rica	4	2	1	5	6	18	3	7	10	8	11	9	12	16	22	15	17	14	23	26	13	19	27	21	20	25	29	24	28
El Salvador	2	4	1	6	7	15	3	14	9	8	5	12	10	18	19	13	11	16	20	25	22	17	27	26	21	23	29	24	28
Guatemala	5	4	1	7	10	13	2	8	9	6	3	12	11	19	20	14	15	16	21	27	23	17	28	26	18	22	29	25	24
Honduras	1	7	3	5	10	18	4	2	8	6	9	13	12	21	20	14	15	17	22	24	19	16	28	26	23	11	25	27	29
Mexico	1	2	3	5	6	16	4	10	9	8	7	12	15	17	21	13	14	11	20	28	18	23	29	24	19	22	27	26	25
Nicaragua	5	3	1	6	7	16	2	10	9	8	4	12	13	18	21	14	11	15	17	25	22	20	28	24	19	23	29	26	27
Panama	4	2	3	5	7	15	1	10	9	8	6	11	12	18	20	13	19	14	21	23	16	17	26	25	24	22	29	27	28
Venezuela (Bolivarian Republic of)	1	4	3	5	7	15	2	13	8	9	6	10	16	18	22	12	17	11	14	25	20	21	27	24	19	23	29	26	28
**Tropical Latin America**	1	2	4	5	6	7	3	13	8	11	9	12	10	15	14	17	18	19	16	21	20	22	28	24	23	25	26	27	29
Brazil	1	2	4	5	6	7	3	13	8	11	9	12	10	14	15	17	18	19	16	21	20	22	28	24	23	25	26	27	29
Paraguay	1	2	6	4	7	10	3	12	8	9	5	11	13	20	18	14	16	15	19	22	23	17	29	25	21	24	28	27	26
North Africa and Middle East	1	3	2	4	7	13	9	5	8	6	16	12	10	11	20	14	17	18	15	27	19	21	22	26	25	23	28	24	29
**North Africa and Middle East**	1	3	2	4	7	13	9	5	8	6	16	12	10	11	20	14	17	18	15	27	19	21	22	26	25	23	28	24	29
Afghanistan	5	8	1	7	15	10	12	6	2	3	11	4	9	14	21	17	16	20	13	26	19	22	24	25	27	23	28	18	29

Algeria	1	3	4	2	7	19	8	12	5	10	11	6	15	14	21	16	9	20	17	23	18	26	13	27	25	24	29	22	28
Bahrain	1	3	6	2	4	15	8	7	9	5	17	10	12	13	18	11	19	14	21	27	16	20	24	28	25	23	26	22	29
Egypt	2	5	7	4	9	13	11	1	6	8	19	12	10	3	21	15	14	17	18	24	16	20	27	26	25	22	29	23	28
Iran (Islamic Republic of)	2	3	1	4	8	10	6	11	9	5	18	13	7	12	20	15	17	16	14	26	19	21	27	23	24	22	29	25	28
Iraq	1	5	9	2	8	17	11	7	6	3	16	12	4	10	19	14	20	15	13	24	21	18	26	28	25	22	27	23	29
Jordan	1	2	7	3	5	16	9	12	8	4	17	6	10	11	18	13	15	14	21	25	19	20	23	28	24	22	26	29	27
Kuwait	1	2	9	3	4	15	5	12	8	6	18	7	11	10	20	13	16	14	21	26	17	19	24	28	23	22	25	27	29
Lebanon	1	3	6	2	7	21	4	13	9	8	19	12	11	5	20	10	17	16	14	27	15	18	25	24	26	23	29	22	28
Libya	1	2	6	3	4	20	11	7	8	5	17	12	10	9	22	14	16	18	13	26	19	23	15	27	25	24	28	21	29
Morocco	1	3	4	2	8	16	7	14	9	12	5	6	17	15	19	11	18	22	10	24	20	21	13	27	26	23	28	25	29
Oman	1	2	5	3	8	13	11	9	4	7	16	6	10	14	18	12	19	17	21	25	15	27	26	28	22	24	23	20	29
Palestine	1	2	6	3	7	19	9	8	10	5	18	11	4	12	22	13	15	16	20	26	17	14	25	27	24	23	28	21	29
Qatar	1	4	8	3	6	13	11	2	9	5	18	7	10	15	20	14	19	12	16	22	17	24	21	27	26	25	28	23	29
Saudi Arabia	2	1	9	3	5	14	11	8	6	7	18	4	10	19	17	12	15	13	22	25	20	23	16	27	26	21	29	24	28
Sudan	3	5	1	4	11	8	9	10	6	2	15	13	7	12	21	18	17	16	14	27	19	23	26	24	25	22	29	20	28
Syrian Arab Republic	2	4	6	3	9	14	8	7	10	1	15	12	5	11	21	13	19	17	16	23	18	20	26	25	22	24	28	27	29
Tunisia	1	2	4	3	6	21	7	14	8	9	15	16	20	5	17	12	11	18	10	23	19	22	13	27	25	24	28	26	29
Turkey	1	2	3	5	4	17	6	12	9	7	19	11	8	10	23	13	18	14	15	29	16	20	26	24	22	25	21	27	28
United Arab Emirates	2	4	10	3	1	5	15	12	9	8	18	7	6	13	19	17	20	11	14	24	16	27	23	25	26	22	29	21	28
Yemen	2	5	1	4	15	8	9	10	6	3	13	14	7	12	20	17	16	21	11	27	18	23	24	25	26	22	29	19	28
South Asia	2	5	3	1	12	8	11	14	4	10	9	16	18	19	6	17	15	22	13	7	21	23	20	29	26	24	27	25	28
**South Asia**	2	5	3	1	12	8	11	14	4	10	9	16	18	19	6	17	15	22	13	7	21	23	20	29	26	24	27	25	28
Bangladesh	2	5	4	3	14	7	8	11	1	12	10	18	15	19	6	17	16	22	13	9	21	24	20	27	26	23	28	25	29
Bhutan	2	3	1	4	13	6	8	12	5	11	10	16	17	19	7	15	14	22	18	9	20	24	21	27	26	23	28	25	29
India	1	4	3	2	11	9	12	13	6	10	8	16	17	19	5	18	15	22	14	7	21	24	20	28	26	23	27	25	29
Nepal	4	6	3	2	12	5	10	16	1	11	7	17	18	19	8	15	13	22	14	9	21	24	20	27	26	23	28	25	29
Pakistan	2	6	7	1	17	5	12	15	3	10	18	19	14	11	4	8	16	25	9	13	23	20	22	27	28	24	29	21	26
Southeast Asia, East Asia, and Oceania	1	3	2	6	7	4	10	5	8	9	11	13	12	14	18	15	16	19	20	25	23	21	17	26	22	24	28	27	29
**East Asia**	1	3	2	7	6	4	11	5	8	10	12	13	9	14	19	16	15	18	20	25	22	23	17	26	21	24	27	28	29
China	1	3	2	7	6	4	11	5	8	10	12	13	9	14	19	16	15	18	20	25	22	23	17	26	21	24	27	28	29
Democratic People’s Republic of Korea	1	3	2	6	10	5	12	4	8	9	7	13	11	15	18	17	14	21	20	26	23	19	16	25	22	24	28	27	29
Taiwan (Province of China)	1	2	3	7	4	5	9	6	10	13	15	12	20	11	8	19	16	14	23	17	21	22	18	26	25	24	27	28	29
**Oceania**	2	5	3	1	11	12	6	9	8	7	4	15	10	17	14	16	20	23	21	27	19	13	18	25	24	22	28	26	29
American Samoa	1	4	5	3	11	17	2	6	9	12	10	13	15	14	22	8	23	21	24	26	18	7	16	25	20	19	27	28	29

Cook Islands	1	5	6	3	7	10	2	4	9	11	12	13	15	8	16	14	18	19	22	25	20	17	26	24	23	21	27	28	29
Fiji	4	3	7	1	10	11	5	8	9	6	2	13	16	15	14	19	18	21	22	26	20	12	25	28	24	17	29	27	23
Guam	1	2	6	3	7	11	4	5	13	9	10	8	18	16	15	14	23	19	25	24	20	17	12	26	22	21	27	28	29
Kiribati	3	8	4	2	15	9	11	5	7	10	1	12	19	21	6	18	20	14	24	23	22	13	17	28	27	26	29	25	16
Marshall Islands	1	6	3	2	10	15	7	5	9	8	4	12	13	17	16	14	20	23	21	27	19	11	18	25	24	22	28	26	29
Micronesia (Federated States of)	1	4	3	2	10	14	7	6	9	8	5	13	15	17	16	12	20	21	22	26	19	11	18	25	24	23	27	28	29
Nauru	1	4	3	2	10	15	9	6	8	7	5	11	12	17	16	14	21	19	22	26	20	13	18	25	24	23	28	27	29
Niue	1	3	5	2	7	13	4	6	9	10	8	12	17	15	16	11	21	18	24	26	19	14	20	25	22	23	27	28	29
Northern Mariana Islands	1	2	4	3	8	15	6	5	10	12	7	14	16	18	9	17	24	20	25	21	19	11	13	26	22	23	27	28	29
Palau	1	5	9	2	7	11	10	8	6	12	3	13	14	18	4	15	21	20	23	26	22	25	24	19	17	16	27	28	29
Papua New Guinea	2	6	3	1	13	10	5	15	8	7	4	18	9	17	12	16	20	23	21	27	19	11	14	25	24	22	28	26	29
Samoa	3	4	2	1	11	17	7	8	5	9	6	12	15	14	19	10	21	22	26	28	24	13	16	18	23	25	29	20	27
Solomon Islands	2	5	3	1	14	13	8	10	9	6	4	7	12	18	15	17	19	23	21	27	20	11	16	25	24	22	28	26	29
Tokelau	1	3	5	2	9	14	4	7	8	10	6	12	16	15	17	13	20	21	24	26	18	11	19	25	22	23	27	28	29
Tonga	1	8	4	3	11	15	5	2	10	12	6	9	17	18	19	14	23	22	25	27	20	16	21	26	13	24	29	28	7
Tuvalu	1	5	3	2	10	14	4	7	8	9	6	12	15	16	17	13	20	22	21	26	19	11	18	25	24	23	28	27	29
Vanuatu	1	7	2	3	10	12	4	5	9	8	6	13	11	16	15	18	20	23	21	28	19	14	17	25	24	22	27	26	29
**Southeast Asia**	1	2	5	3	9	13	8	4	6	7	10	11	15	18	14	12	17	19	21	23	24	20	16	26	25	22	28	27	29
Cambodia	1	2	4	5	10	14	9	3	7	6	8	13	12	18	15	11	20	22	19	25	23	17	16	27	24	21	28	26	29
Indonesia	1	2	5	3	8	12	6	14	4	7	9	11	13	18	15	10	22	19	20	25	24	16	17	27	23	21	28	26	29
Lao People's Democratic Republic	1	2	4	3	10	14	9	5	8	6	7	13	11	18	15	12	21	20	19	25	23	17	16	27	24	22	28	26	29
Malaysia	1	2	4	3	12	14	10	5	11	6	9	8	17	13	15	16	21	18	20	23	24	19	7	26	25	22	29	27	28
Maldives	1	2	9	3	5	15	8	4	6	7	14	13	10	16	12	11	19	17	21	22	18	23	28	27	26	20	25	24	29
Mauritius	2	1	4	3	6	11	5	13	7	9	8	16	17	14	12	10	19	18	20	23	21	15	22	27	25	24	29	26	28
Myanmar	1	3	5	4	11	14	8	10	7	6	9	2	13	16	15	12	21	19	20	25	23	17	18	27	24	22	28	26	29
Philippines	1	2	8	3	9	18	6	4	5	7	10	12	13	20	14	11	22	17	21	25	23	16	15	26	24	19	29	28	27
Seychelles	3	1	8	4	6	12	2	11	14	9	5	10	16	17	7	15	23	20	13	18	19	22	21	24	25	27	29	26	28
Sri Lanka	1	3	5	2	9	6	12	10	4	8	16	13	14	19	7	17	15	11	24	18	21	20	23	28	22	25	27	26	29
Thailand	2	3	5	4	8	12	11	1	9	7	10	16	14	17	13	15	6	20	18	25	23	22	19	26	21	24	27	28	29
Timor-Leste	1	2	4	3	10	13	7	5	8	6	9	12	11	18	15	14	19	21	20	25	24	17	16	27	23	22	28	26	29
Viet Nam	1	2	4	3	7	12	10	14	5	9	6	16	18	19	8	15	20	22	17	13	25	24	11	27	23	21	28	26	29
Sub-Saharan Africa	4	6	7	1	11	8	3	10	5	9	2	12	13	15	16	14	21	18	17	26	19	20	22	24	27	25	28	23	29
**Central sub-Saharan Africa**	3	8	5	2	11	4	6	12	7	9	1	10	14	13	15	16	21	20	17	25	18	19	23	22	27	26	29	24	28
Angola	1	4	5	3	11	6	7	12	8	9	2	10	13	14	15	17	21	18	16	24	19	20	23	22	27	26	29	25	28

Central African Republic	2	9	3	4	15	5	7	10	6	8	1	11	12	14	13	17	20	21	16	25	18	19	23	24	27	26	28	22	29
Congo	2	4	7	1	9	5	6	13	8	10	3	11	15	12	14	16	21	18	17	23	20	19	24	22	26	25	28	27	29
Democratic Republic of the Congo	3	8	5	2	13	4	7	12	6	9	1	10	14	11	15	16	20	21	17	25	18	19	24	22	27	26	28	23	29
Equatorial Guinea	1	3	8	2	9	6	5	12	7	11	4	10	16	13	15	14	21	17	20	22	19	18	24	23	25	26	29	27	28
Gabon	1	3	7	2	8	5	4	13	9	12	6	10	17	11	14	15	21	16	18	22	19	20	24	23	25	26	28	27	29
**Eastern sub-Saharan Africa**	9	6	7	3	12	4	5	10	2	8	1	11	14	16	15	13	22	18	19	26	21	20	17	25	27	24	28	23	29
Burundi	7	8	6	4	14	2	5	10	3	9	1	11	13	16	12	15	23	22	18	25	20	21	17	24	27	26	28	19	29
Comoros	7	5	8	3	9	2	4	13	6	10	1	12	16	15	14	11	22	18	21	26	20	17	19	23	27	24	28	25	29
Djibouti	6	7	8	4	10	2	3	12	5	9	1	11	15	16	14	13	23	17	19	22	21	20	18	24	27	26	29	25	28
Eritrea	7	6	5	2	14	3	8	11	4	9	1	10	15	16	13	12	23	22	19	25	24	18	17	21	27	26	28	20	29
Ethiopia	7	5	6	3	16	11	8	9	2	1	4	20	10	13	15	12	17	18	22	27	24	23	19	25	26	14	28	21	29
Kenya	8	6	3	2	13	1	4	12	7	11	5	9	15	20	10	14	19	22	17	21	18	24	16	25	26	27	28	23	29
Madagascar	8	7	6	2	13	3	5	11	4	9	1	10	15	16	14	12	24	22	19	25	23	18	17	21	27	26	28	20	29
Malawi	6	8	12	4	13	2	7	11	1	10	3	9	16	5	18	15	23	14	24	29	22	21	26	17	25	20	28	19	27
Mozambique	5	7	8	3	12	6	11	10	2	4	1	9	13	15	17	14	21	23	16	25	20	19	29	22	26	24	27	18	28
Rwanda	6	7	8	2	13	3	5	10	4	9	1	12	15	16	14	11	22	21	19	25	20	18	17	23	27	26	28	24	29
Somalia	8	9	4	5	18	2	7	10	3	6	1	12	11	15	13	14	22	25	19	26	21	20	16	23	27	24	28	17	29
South Sudan	6	5	8	7	12	2	3	11	4	9	1	10	14	13	15	16	24	17	20	23	21	19	18	25	27	26	29	22	28
Uganda	8	6	7	4	11	2	1	9	5	10	3	13	16	17	14	12	25	21	22	20	19	18	15	24	27	26	29	23	28
United Republic of Tanzania	6	7	8	5	11	3	4	14	2	9	1	10	13	16	15	12	22	17	21	25	20	19	18	24	27	26	28	23	29
Zambia	6	5	8	4	11	2	7	14	3	9	1	10	15	16	13	12	24	17	19	23	21	20	18	25	27	26	29	22	28
**Southern sub-Saharan Africa**	1	6	8	3	9	5	2	7	10	12	4	11	17	14	15	13	22	20	18	24	16	19	26	21	23	27	25	28	29
Botswana	1	5	7	2	8	6	4	19	9	10	3	13	14	15	11	12	22	20	16	21	17	18	26	23	25	29	24	28	27
Eswatini	2	7	8	5	9	4	6	1	10	12	3	11	15	16	14	13	22	19	17	24	18	20	25	21	27	26	23	28	29
Lesotho	2	8	7	3	10	5	6	4	9	11	1	12	18	16	13	14	23	22	15	25	19	17	24	20	26	27	21	28	29
Namibia	5	4	11	1	9	15	2	10	6	13	3	8	17	18	7	16	24	19	14	20	22	21	26	12	25	28	23	27	29
South Africa	1	4	9	3	8	5	2	7	11	12	6	10	16	15	14	13	22	19	18	25	17	20	26	21	23	27	24	28	29
Zimbabwe	5	8	7	3	9	4	2	6	11	13	1	20	19	10	17	12	21	23	16	25	14	15	24	18	26	22	28	27	29
**Western sub-Saharan Africa**	4	6	5	2	10	12	1	8	7	9	3	11	13	14	17	15	20	18	16	26	21	22	23	24	25	27	28	19	29
Benin	3	6	2	5	11	10	4	9	7	8	1	12	13	14	17	15	20	16	18	26	21	19	23	24	25	27	28	22	29
Burkina Faso	4	8	2	3	11	9	5	13	6	7	1	10	12	14	15	16	20	18	17	25	21	19	23	24	26	27	28	22	29
Cabo Verde	3	6	2	8	7	4	1	5	10	11	9	13	15	14	12	18	20	16	19	22	21	17	24	26	23	25	29	27	28
Cameroon	1	6	3	4	7	9	5	22	10	8	2	11	13	12	15	14	19	21	16	23	20	17	18	26	25	28	27	24	29
Chad	3	8	1	5	13	10	4	9	6	7	2	11	12	14	15	20	18	17	16	26	21	19	23	24	25	27	29	22	28

Côte d'Ivoire	2	6	1	5	11	9	4	10	7	8	3	12	13	14	16	15	20	18	17	24	21	19	23	25	26	27	28	22	29
Gambia	3	5	7	4	10	11	8	1	6	9	2	16	15	13	14	12	17	18	21	25	20	19	27	24	23	26	29	22	28
Ghana	5	6	7	1	4	13	2	8	12	10	3	9	11	14	21	15	19	18	16	24	20	17	26	23	22	25	28	27	29
Guinea	6	8	3	5	14	13	4	1	7	10	2	20	15	9	11	12	21	22	16	24	23	18	27	19	26	25	29	17	28
Guinea-Bissau	4	6	2	3	11	10	5	8	7	9	1	12	13	14	16	15	19	20	17	25	21	18	23	24	27	26	28	22	29
Liberia	5	6	2	4	10	9	3	7	8	11	1	12	14	13	16	15	20	18	17	26	21	19	23	24	25	27	28	22	29
Mali	6	7	1	4	11	12	9	2	8	10	3	18	13	5	15	16	19	14	17	26	21	20	27	22	25	24	29	23	28
Mauritania	2	6	3	5	7	10	1	9	8	11	4	12	15	13	16	14	19	18	20	24	21	17	26	23	22	27	28	25	29
Niger	4	8	2	7	11	9	3	21	5	6	1	13	10	12	14	17	18	19	16	24	20	15	26	25	23	29	28	22	27
Nigeria	5	4	9	2	8	19	1	10	6	7	3	12	11	18	21	13	22	16	15	27	20	24	17	23	25	26	28	14	29
São Tomé and Príncipe	1	5	3	6	13	8	7	14	2	11	4	12	21	9	20	10	15	17	18	22	19	16	26	25	24	23	27	28	29
Senegal	3	6	2	4	10	8	1	13	7	9	5	11	14	12	16	15	20	19	18	26	21	17	25	24	23	27	28	22	29
Sierra Leone	4	6	2	5	11	10	3	9	7	8	1	12	13	14	16	15	20	18	17	26	21	19	23	25	24	27	28	22	29
Togo	3	6	2	4	11	9	5	8	7	10	1	12	14	13	16	15	20	19	17	25	21	18	23	24	26	27	28	22	29

eFigure 11: Ranking of 29 cancer groups by mortality in 2019, by country and location group

Footnote: Cancer rankings by location compare the absolute number of deaths for each cancer, with colors ranging from the highest ranking (red) to the lowest ranking (green) within that location. Socio-demographic index (SDI) quintiles are ordered from high to low SDI quintile. Countries and location groups are ordered alphabetically by the GBD location hierarchy of 7 super-regions (bold, italicized) and 21 regions (bold).

Location/grouping: *Global*, SDI quintile, ***Super Region***, **Region**, Country or Territory	Non-melanoma skin cancer	Tracheal, bronchus, and lung cancer	Colon and rectum cancer	Breast cancer	Prostate cancer	Stomach cancer	Other malignant neoplasms	Leukemia	Cervical cancer	Esophageal cancer	Liver cancer	Pancreatic cancer	Bladder cancer	Non-Hodgkin lymphoma	Uterine cancer	Lip and oral cavity cancer	Kidney cancer	Brain and central nervous system cancer	Ovarian cancer	Malignant skin melanoma	Thyroid cancer	Larynx cancer	Gallbladder and biliary tract cancer	Nasopharynx cancer	Other pharynx cancer	Multiple myeloma	Testicular cancer	Hodgkin lymphoma	Mesothelioma
Global	1	2	3	4	5	6	7	8	9	10	11	12	13	14	15	16	17	18	19	20	21	22	23	24	25	26	27	28	29
High SDI	1	3	2	5	4	6	7	11	22	16	15	8	10	12	13	18	14	17	19	9	21	25	23	28	24	20	26	27	29
High-middle SDI	3	1	2	4	6	5	7	9	14	12	16	10	8	15	11	19	13	17	18	21	22	23	24	20	27	26	25	28	29
Middle SDI	5	1	2	3	6	4	7	11	10	9	8	12	13	14	17	16	20	15	18	27	21	22	23	19	24	25	26	28	29
Low-middle SDI	7	2	3	1	8	4	6	10	5	11	12	14	19	15	22	9	23	17	16	28	20	18	21	24	13	25	27	26	29
Low SDI	11	5	8	1	7	6	3	4	2	10	12	17	16	15	21	9	23	13	14	27	19	18	22	25	20	26	28	24	29
Central Europe, Eastern Europe, and Central Asia	4	2	1	3	5	6	8	13	12	21	19	11	9	16	7	18	10	17	14	15	22	20	25	28	23	26	24	27	29
**Central Asia**	1	3	5	2	12	4	7	10	6	11	8	14	16	18	9	19	15	13	17	23	21	20	26	28	25	27	22	24	29
Armenia	3	2	4	1	5	6	11	13	10	22	12	7	8	18	9	20	15	14	16	24	19	17	23	29	28	25	21	27	26
Azerbaijan	2	1	5	3	8	4	12	6	9	7	16	11	15	19	13	21	10	14	17	25	20	18	23	28	27	26	24	22	29
Georgia	3	2	4	1	6	5	9	12	10	23	19	11	8	17	7	18	13	15	14	21	22	16	25	29	26	27	20	24	28
Kazakhstan	1	3	4	2	9	5	7	15	6	10	13	12	16	20	8	17	11	18	14	21	19	22	26	28	25	27	23	24	29
Kyrgyzstan	1	4	6	3	12	2	7	10	5	13	18	9	17	19	8	16	14	15	11	23	20	22	24	28	25	26	21	27	29
Mongolia	4	3	8	7	18	2	9	12	6	5	1	10	20	17	13	16	15	11	14	28	21	22	19	25	24	23	27	26	29
Tajikistan	1	4	5	3	14	2	8	7	11	10	16	12	18	17	9	19	13	6	15	24	26	20	25	23	22	21	29	27	28
Turkmenistan	1	4	8	2	13	7	3	10	5	6	11	15	18	19	22	14	9	12	16	26	20	24	27	28	23	25	17	21	29
Uzbekistan	2	4	6	1	18	3	7	8	5	12	11	15	19	13	10	16	14	9	17	25	24	20	28	26	22	27	21	23	29
**Central Europe**	3	2	1	4	5	9	12	11	14	24	21	7	6	15	8	18	10	17	16	13	25	19	22	28	23	26	20	27	29
Albania	1	2	5	4	3	6	7	10	14	23	8	9	18	17	12	20	13	11	21	22	19	15	25	28	27	26	16	24	29
Bosnia and Herzegovina	3	1	2	4	5	6	11	14	15	22	10	8	7	18	9	20	13	12	16	17	23	19	21	28	26	25	24	27	29
Bulgaria	1	4	2	3	5	8	11	12	10	22	19	9	7	18	6	17	15	13	14	20	23	16	24	28	25	26	21	27	29
Croatia	5	2	1	3	4	8	14	9	18	25	21	10	6	15	7	17	11	13	16	12	24	19	20	29	26	22	23	27	28

Czechia	1	3	2	5	4	13	12	11	17	22	25	9	7	14	10	19	6	18	15	8	21	26	16	28	23	24	20	27	29
Hungary	3	2	1	4	5	8	13	10	16	23	25	7	6	15	11	12	9	20	17	14	24	19	21	28	18	26	22	27	29
Montenegro	4	1	3	2	5	11	17	8	15	24	14	7	6	18	9	20	13	12	19	16	22	10	25	28	27	26	21	23	29
North Macedonia	4	1	3	2	5	6	10	13	15	25	11	9	7	20	8	21	18	14	17	12	22	16	23	28	27	26	19	24	29
Poland	4	1	2	3	5	9	12	10	17	21	26	8	6	14	7	18	11	16	13	15	24	19	22	29	23	25	20	27	28
Romania	3	2	1	4	5	6	10	12	8	22	21	9	7	18	11	14	13	16	17	19	24	15	25	28	20	26	23	27	29
Serbia	4	1	2	3	5	9	10	13	11	23	18	8	6	17	7	20	14	12	19	15	25	16	22	28	24	27	21	26	29
Slovakia	3	2	1	4	5	7	11	13	15	24	25	8	6	14	9	16	10	20	17	12	26	23	18	28	21	22	19	27	29
Slovenia	1	4	2	5	3	7	14	8	21	25	15	10	12	9	13	20	11	19	16	6	26	24	17	29	18	22	23	27	28
**Eastern Europe**	4	3	1	2	5	6	9	13	12	20	23	10	11	16	7	17	8	18	14	15	19	21	27	28	22	26	24	25	29
Belarus	5	4	1	3	2	6	8	10	12	21	23	11	14	17	9	16	7	20	15	13	19	18	27	28	22	24	26	25	29
Estonia	5	4	2	3	1	6	12	13	15	23	19	9	10	14	7	18	8	17	16	11	22	21	25	28	24	20	26	27	29
Latvia	5	4	1	3	2	7	12	11	17	19	23	10	9	15	6	18	8	16	14	13	20	21	26	28	24	22	25	27	29
Lithuania	3	4	2	5	1	6	11	12	15	18	21	8	10	16	9	19	7	17	13	14	23	22	25	28	24	20	26	27	29
Republic of Moldova	2	4	1	3	5	6	10	13	9	23	21	8	11	15	7	19	12	17	16	20	22	14	27	28	18	26	25	24	29
Russian Federation	4	3	1	2	5	7	8	13	12	18	23	10	11	17	6	21	9	20	14	15	16	22	26	28	19	27	24	25	29
Ukraine	3	2	1	4	6	5	10	12	13	22	23	9	11	18	8	14	7	17	15	16	20	19	26	28	21	25	27	24	29
High-income	1	3	2	5	4	6	8	10	21	17	15	9	7	12	13	19	14	16	18	11	22	24	23	29	25	20	26	27	28
**Australasia**	1	6	3	4	2	12	13	8	22	17	18	9	10	7	14	20	11	16	19	5	21	25	26	29	28	15	23	27	24
Australia	1	6	3	4	2	12	13	8	22	17	18	10	9	7	15	21	11	16	19	5	20	25	26	29	28	14	23	27	24
New Zealand	1	6	2	4	3	14	13	8	21	17	18	10	11	7	12	19	9	16	20	5	24	26	23	29	28	15	22	27	25
**High-income Asia Pacific**	14	2	1	4	5	3	11	12	19	13	6	7	9	8	15	23	16	17	20	27	18	24	10	26	25	22	28	21	29
Brunei Darussalam	19	3	2	1	11	5	8	9	4	24	7	12	17	6	18	20	15	13	10	28	16	27	22	14	25	23	26	21	29
Japan	14	2	1	4	5	3	11	12	18	13	6	7	9	8	15	23	16	17	20	26	19	25	10	27	24	22	28	21	29
Republic of Korea	14	2	1	5	6	3	10	11	16	18	4	7	9	12	21	25	17	15	19	24	13	20	8	27	26	22	28	23	29
Singapore	8	3	1	2	4	7	9	10	17	20	6	12	14	11	13	23	15	16	19	26	18	22	25	5	27	24	28	21	29
**High-income North America**	1	3	5	4	2	14	7	12	22	21	17	11	13	9	8	15	10	16	19	6	20	23	27	29	24	18	25	26	28
Canada	1	3	2	4	5	14	6	8	20	18	17	11	10	7	13	21	12	15	19	9	22	23	26	29	27	16	24	25	28
Greenland	1	2	3	4	8	9	11	20	10	6	14	5	12	17	26	15	7	18	13	25	23	22	24	16	19	21	27	29	28
United States of America	1	3	5	4	2	14	8	12	22	21	17	11	13	10	7	15	9	16	19	6	20	23	27	29	24	18	25	26	28
**Southern Latin America**	1	4	2	3	5	6	10	12	7	16	25	8	11	13	17	23	9	19	18	20	24	22	14	29	27	21	15	26	28
Argentina	3	4	1	2	5	8	10	12	6	14	25	7	11	13	17	22	9	19	16	21	24	20	18	29	27	23	15	26	28
Chile	1	6	3	4	2	5	11	15	9	17	23	12	13	14	16	24	10	21	18	20	22	25	7	29	27	19	8	26	28
Uruguay	3	4	1	2	5	7	12	13	10	14	24	6	9	11	17	19	8	21	16	20	25	18	15	28	26	22	23	27	29

**Western Europe**	2	5	1	3	4	12	8	7	21	19	16	10	6	11	13	18	14	15	17	9	22	23	25	29	24	20	26	27	28
Andorra	2	4	1	3	5	11	7	6	17	21	13	15	9	8	14	18	16	10	23	12	20	24	22	29	26	19	25	27	28
Austria	3	5	2	4	1	12	10	7	22	23	15	8	6	11	13	18	14	17	16	9	19	26	25	29	21	20	24	27	28
Belgium	4	3	2	1	5	13	8	7	21	16	20	11	6	10	12	17	14	15	18	9	24	22	28	29	23	19	25	27	26
Cyprus	4	5	3	1	2	11	8	7	19	26	17	13	6	10	9	20	16	14	15	12	21	22	23	28	29	18	24	25	27
Denmark	2	3	1	5	4	15	13	9	23	17	21	10	6	11	12	18	14	8	16	7	26	24	25	29	20	19	22	27	28
Finland	2	5	4	3	1	15	9	14	23	20	16	7	13	8	11	19	10	12	17	6	21	27	22	29	26	18	25	24	28
France	1	4	2	3	5	14	8	7	23	19	15	10	6	11	12	17	13	16	18	9	25	22	28	29	20	21	24	27	26
Germany	5	4	1	3	2	11	8	6	23	15	16	7	9	13	14	19	12	20	17	10	24	26	21	29	22	18	25	27	28
Greece	1	2	4	3	5	8	11	7	21	26	19	9	6	15	12	22	13	10	16	14	25	17	24	27	28	20	23	18	29
Iceland	2	3	5	4	1	14	9	11	22	17	21	12	7	13	16	20	8	10	19	6	15	24	25	29	26	18	28	23	27
Ireland	1	5	2	3	4	14	10	7	19	16	21	12	8	9	11	20	15	13	17	6	22	25	27	28	26	18	23	24	29
Israel	1	4	3	2	5	14	11	6	19	25	20	10	8	7	13	21	15	12	16	9	18	24	26	27	29	17	23	22	28
Italy	1	5	2	3	4	9	7	8	23	25	15	11	6	12	10	21	13	18	19	14	17	22	20	29	27	16	24	26	28
Luxembourg	1	4	3	2	5	14	9	8	25	19	17	12	6	13	10	16	18	11	15	7	21	24	28	27	23	20	22	26	29
Malta	1	5	3	2	4	12	8	9	26	22	21	7	6	10	11	17	13	15	16	14	18	23	29	24	28	20	19	25	27
Monaco	5	1	3	2	4	13	11	9	24	19	16	8	7	6	21	25	15	18	14	12	22	17	28	29	27	10	20	23	26
Netherlands	5	4	1	2	3	13	8	10	23	15	20	11	7	9	12	19	14	16	17	6	21	26	25	29	27	18	22	28	24
Norway	3	4	2	5	1	15	8	14	20	22	23	10	7	12	13	19	11	9	16	6	18	27	24	29	25	17	21	26	28
Portugal	3	5	1	4	2	7	9	8	18	20	17	12	6	11	10	13	15	16	23	14	19	25	24	28	21	22	26	27	29
San Marino	3	5	1	4	2	6	10	8	24	26	22	13	7	9	23	18	16	12	21	11	15	19	20	25	28	14	27	17	29
Spain	2	4	1	3	5	8	9	7	21	22	16	11	6	12	10	15	14	17	18	13	24	19	23	28	25	20	27	26	29
Sweden	1	5	3	4	2	15	9	8	22	19	20	11	7	10	12	18	14	13	16	6	24	26	21	29	25	17	23	27	28
Switzerland	1	5	4	3	2	12	7	8	24	20	16	10	9	11	13	18	15	14	19	6	21	26	25	29	22	17	23	28	27
United Kingdom	1	4	2	3	5	15	11	9	21	14	19	10	7	8	13	17	12	20	16	6	25	23	28	29	22	18	26	27	24
Latin America and Caribbean	1	5	4	3	2	6	8	9	7	17	18	10	16	11	12	19	14	13	15	24	20	22	21	29	26	23	25	27	28
**Andean Latin America**	4	8	3	6	2	1	7	9	5	23	18	12	19	10	11	22	17	14	15	24	16	25	13	29	27	21	20	26	28
Bolivia (Plurinational State of)	5	7	6	4	3	1	9	8	2	20	14	13	19	12	11	22	18	15	17	23	16	24	10	29	28	21	25	26	27
Ecuador	1	8	4	5	3	2	7	9	6	24	18	12	19	10	11	22	17	15	14	23	13	26	16	29	27	21	20	25	28
Peru	4	8	2	6	1	3	5	9	7	23	18	11	19	10	12	22	16	13	15	24	17	25	14	29	27	21	20	26	28
**Caribbean**	8	4	3	2	1	9	6	10	5	16	19	13	11	12	7	15	17	21	20	26	22	14	23	27	24	18	28	25	29
Antigua and Barbuda	9	7	3	2	1	5	6	11	4	18	21	10	14	12	8	19	16	20	13	23	17	22	24	27	25	15	26	28	29
Bahamas	11	4	3	2	1	7	6	15	5	13	19	16	20	12	8	14	17	22	9	25	21	18	24	27	23	10	29	26	28
Barbados	12	8	3	2	1	7	6	13	4	14	19	11	15	10	5	18	16	21	17	25	20	22	23	26	24	9	28	27	29

Belize	8	5	4	3	1	6	7	10	2	18	15	11	14	13	9	19	12	16	17	25	21	20	22	29	26	24	27	23	28
Bermuda	11	4	2	3	1	10	7	13	19	16	23	6	5	9	8	18	14	20	15	12	22	21	26	27	24	17	29	25	28
Cuba	5	2	3	4	1	11	7	15	10	16	22	12	8	13	6	14	17	18	20	25	21	9	26	28	24	19	27	23	29
Dominica	12	5	3	2	1	4	7	11	6	15	18	9	13	8	14	16	17	24	20	26	23	19	22	27	21	10	29	25	28
Dominican Republic	9	4	3	2	1	7	6	10	5	19	11	15	20	14	8	13	12	24	23	26	17	18	16	25	22	21	28	27	29
Grenada	12	6	3	2	1	8	9	14	4	11	20	10	15	7	5	16	18	21	13	25	19	24	22	26	23	17	29	27	28
Guyana	10	5	4	2	1	6	8	12	3	19	15	11	17	14	7	16	13	20	9	26	18	21	23	29	25	22	27	24	28
Haiti	9	6	7	2	3	5	8	4	1	14	13	20	16	11	10	15	21	12	18	26	23	19	22	27	25	17	29	24	28
Jamaica	10	4	3	2	1	6	8	11	5	18	17	12	15	9	7	22	19	20	14	25	16	21	23	26	24	13	28	27	29
Puerto Rico	7	4	2	3	1	10	5	12	14	18	17	8	11	6	9	19	15	20	16	23	21	22	27	28	26	13	25	24	29
Saint Kitts and Nevis	11	6	3	2	1	8	5	13	4	17	20	9	12	10	7	18	14	22	15	28	19	21	23	26	24	16	25	29	27
Saint Lucia	11	5	3	2	1	6	7	14	4	17	21	8	12	9	10	16	19	22	15	23	18	20	25	28	24	13	27	26	29
Saint Vincent and the Grenadines	10	6	4	2	1	5	8	13	3	20	18	12	15	9	7	11	19	22	14	25	17	16	24	27	23	21	28	26	29
Suriname	9	5	3	2	1	7	6	10	4	21	17	8	16	12	14	19	15	13	11	26	20	23	22	24	27	18	28	25	29
Trinidad and Tobago	9	5	3	2	1	11	6	13	4	20	17	8	16	12	7	18	15	21	10	27	19	23	22	28	24	14	25	26	29
United States Virgin Islands	14	4	2	3	1	6	7	15	9	16	22	5	17	11	12	18	13	19	10	21	25	20	24	26	23	8	29	28	27
**Central Latin America**	1	7	4	3	2	5	8	9	6	23	15	10	18	12	14	21	11	17	13	25	16	24	19	29	27	22	20	26	28
Colombia	1	6	4	3	2	5	7	9	8	21	19	11	18	10	14	23	16	13	12	20	15	25	17	28	27	22	24	26	29
Costa Rica	2	7	4	3	1	5	6	10	8	25	13	11	15	9	12	22	14	17	18	19	16	26	24	28	27	20	23	21	29
El Salvador	1	7	6	4	2	3	8	9	5	21	18	10	19	13	11	20	16	12	14	26	17	23	15	28	27	24	25	22	29
Guatemala	1	8	6	5	3	2	9	7	4	18	10	11	22	16	12	21	15	13	14	26	17	23	19	28	27	24	20	25	29
Honduras	1	2	10	4	3	5	8	9	7	22	6	13	20	17	12	18	16	15	14	26	11	23	19	29	24	21	27	28	25
Mexico	1	8	4	3	2	6	7	9	5	24	14	10	20	13	15	21	11	18	12	23	16	25	19	29	28	22	17	26	27
Nicaragua	2	8	5	3	1	6	7	9	4	23	12	10	19	11	17	21	14	15	13	26	18	20	16	28	27	24	22	25	29
Panama	3	7	4	2	1	5	8	9	6	21	16	12	18	11	10	19	13	14	15	25	17	22	23	28	26	20	24	27	29
Venezuela (Bolivarian Republic of)	2	4	6	3	1	7	8	9	5	23	20	10	15	13	11	18	12	19	14	25	17	16	22	28	27	21	26	24	29
**Tropical Latin America**	1	5	4	3	2	6	8	12	7	11	21	9	14	16	15	13	17	10	18	23	25	19	20	29	24	22	26	27	28
Brazil	1	5	4	3	2	6	8	12	7	11	21	9	13	16	15	14	17	10	18	23	25	19	20	29	24	22	26	27	28
Paraguay	24	5	4	1	2	7	6	8	3	12	21	9	18	11	10	16	13	17	14	23	15	20	22	29	26	25	19	27	28
North Africa and Middle East	9	2	3	1	5	6	4	8	16	20	10	13	7	12	18	23	15	11	17	27	14	19	22	24	28	25	21	26	29
**North Africa and Middle East**	9	2	3	1	5	6	4	8	16	20	10	13	7	12	18	23	15	11	17	27	14	19	22	24	28	25	21	26	29
Afghanistan	12	6	9	4	13	1	2	3	10	11	7	18	14	5	22	23	19	8	17	26	16	15	20	25	27	24	28	21	29
Algeria	6	4	3	1	5	11	2	12	8	25	19	14	13	7	24	22	17	16	18	28	9	21	15	10	27	23	26	20	29
Bahrain	10	4	3	1	2	13	5	7	18	22	16	9	6	8	17	19	11	14	12	26	15	23	24	25	28	21	27	20	29

Egypt	9	6	5	3	8	10	4	7	19	18	1	12	2	14	16	23	15	11	17	27	13	20	22	28	26	21	24	25	29
Iran (Islamic Republic of)	7	6	5	1	4	2	3	8	20	13	15	11	10	14	21	23	16	9	18	26	12	17	25	27	28	24	19	22	29
Iraq	14	2	7	1	8	10	3	4	19	22	9	11	5	15	16	20	13	6	17	28	12	18	25	26	27	24	21	23	29
Jordan	8	3	2	1	6	11	4	5	18	25	19	13	9	7	14	20	15	10	17	27	12	24	21	23	28	22	16	26	29
Kuwait	10	6	2	1	4	15	3	7	18	21	17	14	5	8	13	20	12	11	16	27	9	24	25	26	28	23	19	22	29
Lebanon	8	5	4	1	2	10	6	7	22	27	19	13	3	15	11	23	18	12	14	25	9	17	24	26	28	20	21	16	29
Libya	19	2	3	1	6	11	4	7	12	25	13	8	5	16	20	23	15	10	17	27	14	18	22	9	28	24	26	21	29
Morocco	13	2	3	1	6	9	5	15	4	22	20	14	11	7	17	18	21	19	10	27	8	12	24	16	26	23	28	25	29
Oman	8	7	3	2	4	9	1	6	18	20	12	14	13	5	23	19	15	10	16	26	11	25	27	24	29	21	22	17	28
Palestine	13	3	2	1	7	9	6	5	18	24	12	11	10	14	8	22	17	4	16	27	15	23	21	25	28	20	26	19	29
Qatar	10	6	4	1	3	14	2	5	18	21	7	15	12	8	22	23	11	9	16	27	13	19	26	25	28	24	17	20	29
Saudi Arabia	14	7	3	1	4	15	2	8	19	23	12	11	13	6	18	20	10	9	16	27	5	26	24	17	28	25	21	22	29
Sudan	9	6	5	1	8	3	4	2	13	11	12	14	10	15	24	22	16	7	19	26	17	18	21	27	28	23	25	20	29
Syrian Arab Republic	8	3	5	2	4	9	6	1	16	21	11	12	10	13	14	20	17	7	15	25	19	18	23	26	27	22	24	28	29
Tunisia	7	2	3	1	5	8	6	10	14	25	21	13	4	19	18	17	16	22	15	26	12	11	20	9	27	23	28	24	29
Turkey	5	1	2	3	4	6	7	10	20	23	18	9	8	12	14	25	13	11	17	22	16	19	24	26	29	21	15	27	28
United Arab Emirates	14	8	3	1	5	15	2	12	18	13	17	4	7	9	24	20	6	10	19	26	11	16	25	27	28	22	21	23	29
Yemen	9	5	6	2	8	1	4	3	10	11	13	17	12	16	23	21	18	7	19	25	15	14	20	26	28	24	27	22	29
South Asia	21	4	5	1	10	7	3	9	6	11	16	15	19	14	22	2	23	17	13	28	20	12	18	24	8	25	27	26	29
**South Asia**	21	4	5	1	10	7	3	9	6	11	16	15	19	14	22	2	23	17	13	28	20	12	18	24	8	25	27	26	29
Bangladesh	27	3	7	2	8	5	1	11	6	9	13	17	20	16	22	4	23	15	14	28	19	12	18	21	10	24	26	25	29
Bhutan	27	5	3	1	8	2	6	9	7	10	13	15	20	14	21	4	22	16	12	28	18	19	17	24	11	23	26	25	29
India	20	4	3	1	10	6	7	9	5	11	16	13	21	14	22	2	23	18	15	29	19	12	17	24	8	25	27	26	28
Nepal	26	5	7	2	10	4	1	9	3	8	19	14	20	16	21	6	22	17	12	28	18	13	15	23	11	24	27	25	29
Pakistan	27	4	7	1	12	11	3	5	13	8	18	22	9	21	15	2	24	14	6	28	17	10	19	25	16	26	23	20	29
Southeast Asia, East Asia, and Oceania	5	1	2	4	9	3	7	10	11	6	8	12	14	15	17	18	19	16	20	27	21	22	23	13	26	24	25	28	29
**East Asia**	5	1	2	4	9	3	8	10	12	6	7	11	14	16	17	19	18	15	20	26	22	21	23	13	27	24	25	28	29
China	5	1	3	4	10	2	8	9	13	6	7	11	14	16	17	21	18	15	19	26	22	20	23	12	27	24	25	28	29
Democratic People's Republic of Korea	23	1	3	4	10	2	9	8	6	7	5	12	14	16	11	18	19	13	15	26	17	22	20	21	27	24	28	25	29
Taiwan (Province of China)	26	2	1	3	4	5	8	15	16	9	12	11	7	13	14	6	10	22	20	28	19	23	21	17	18	24	25	27	29
**Oceania**	25	2	6	1	7	4	8	5	3	17	11	15	14	18	9	10	20	12	13	27	16	23	22	19	28	21	24	26	29
American Samoa	22	2	4	1	3	6	9	12	7	21	10	11	13	15	5	19	18	16	8	25	14	24	23	17	26	20	29	28	27
Cook Islands	23	3	4	2	1	6	8	14	10	15	5	9	7	12	11	13	18	19	16	25	17	22	20	28	27	21	24	26	29
Fiji	25	4	3	1	5	8	7	6	2	16	10	12	14	15	9	13	20	17	18	28	11	23	21	27	26	22	19	24	29

Guam	22	1	2	3	4	6	12	11	5	19	7	9	13	10	8	14	17	20	15	27	16	23	24	18	25	21	28	26	29
Kiribati	27	4	8	2	14	5	7	9	1	10	6	16	18	15	12	3	13	19	17	28	24	25	21	20	23	22	11	26	29
Marshall Islands	24	2	5	1	7	4	9	8	3	18	6	13	14	15	10	12	20	16	11	26	17	23	22	19	28	21	29	25	27
Micronesia (Federated States of)	24	2	5	1	6	4	10	9	3	17	7	12	14	16	8	13	19	18	11	25	15	23	22	20	28	21	29	26	27
Nauru	27	2	4	1	9	5	8	6	3	19	10	16	17	11	7	13	18	15	14	26	12	22	23	20	28	21	24	25	29
Niue	24	2	4	1	3	6	9	11	5	18	8	10	12	15	7	14	17	19	13	25	16	22	23	21	27	20	26	28	29
Northern Mariana Islands	24	1	3	2	4	8	10	13	6	20	9	11	12	14	5	7	16	19	17	26	15	23	25	18	22	21	29	27	28
Palau	20	1	6	2	5	8	7	12	3	15	9	10	16	13	19	4	18	17	14	21	11	22	24	25	26	23	29	27	28
Papua New Guinea	24	2	8	1	7	4	6	5	3	14	18	16	13	20	9	11	19	10	12	26	15	23	22	17	27	21	29	25	28
Samoa	26	5	3	1	7	4	6	9	2	23	11	12	14	15	10	16	21	18	8	22	13	27	24	19	28	25	20	17	29
Solomon Islands	25	3	6	1	10	4	7	5	2	17	11	18	16	8	9	12	20	14	15	26	13	23	22	19	27	21	29	24	28
Tokelau	23	2	5	1	4	6	9	10	3	18	8	11	13	16	7	14	19	17	12	25	15	24	22	21	26	20	28	27	29
Tonga	20	2	8	1	5	7	9	12	6	17	3	13	16	10	11	15	21	18	14	26	19	25	24	23	28	22	4	27	29
Tuvalu	24	2	6	1	4	5	9	10	3	18	7	11	14	15	8	13	19	17	12	25	16	23	22	20	27	21	29	26	28
Vanuatu	24	1	6	2	5	4	9	8	3	16	7	13	15	14	10	11	20	12	17	25	18	23	22	19	28	21	29	26	27
**Southeast Asia**	20	2	3	1	6	9	5	8	4	19	7	13	18	14	15	10	17	16	11	28	12	23	22	21	24	25	26	27	29
Cambodia	22	1	2	3	9	7	8	5	4	17	6	15	18	16	12	11	19	14	10	28	13	21	23	20	25	24	27	26	29
Indonesia	20	2	3	1	6	8	5	7	4	18	19	10	17	14	11	12	15	16	9	28	13	22	23	21	25	24	26	27	29
Lao People's Democratic Republic	21	1	3	2	9	6	8	4	5	17	7	14	18	16	11	13	19	12	10	28	15	22	23	20	25	24	27	26	29
Malaysia	19	3	2	1	5	10	8	6	4	20	11	18	13	7	15	12	17	21	16	28	14	22	24	9	25	26	23	27	29
Maldives	21	3	2	1	5	15	4	6	10	20	8	13	16	11	18	9	17	14	7	26	12	22	25	29	24	23	28	19	27
Mauritius	21	3	2	1	4	5	8	12	7	16	17	9	13	15	6	10	14	20	11	28	18	19	23	24	25	22	27	26	29
Myanmar	20	1	3	2	9	7	8	5	6	18	11	13	17	4	12	14	19	16	10	28	15	22	23	21	25	24	27	26	29
Philippines	18	3	2	1	6	11	7	4	5	21	8	13	20	15	12	14	17	16	9	28	10	22	25	19	26	24	23	27	29
Seychelles	23	5	2	3	1	13	12	7	4	15	16	14	10	8	17	6	20	18	9	28	24	11	25	22	19	21	26	27	29
Sri Lanka	22	3	2	1	7	8	4	9	11	10	17	16	18	12	13	5	6	19	15	29	14	23	20	26	21	24	27	25	28
Thailand	13	2	4	3	6	9	7	8	5	16	1	12	15	18	19	10	21	17	14	28	20	22	11	23	25	24	26	27	29
Timor-Leste	20	1	2	3	6	7	8	4	5	17	9	14	18	15	12	13	19	10	11	28	16	22	23	21	25	24	27	26	29
Viet Nam	23	2	3	1	9	5	4	10	6	18	20	12	16	17	19	8	22	21	11	28	7	15	24	13	14	26	25	27	29
Sub-Saharan Africa	9	4	7	2	3	8	5	6	1	10	11	12	16	13	19	17	18	15	14	26	20	21	25	24	27	23	28	22	29
**Central sub-Saharan Africa**	10	3	9	2	5	7	4	8	1	6	16	15	11	12	18	13	19	14	17	24	22	20	23	26	28	21	27	25	29
Angola	10	3	5	2	6	8	7	4	1	9	16	15	13	11	19	12	18	14	17	24	22	20	23	27	28	21	25	26	29
Central African Republic	10	3	9	2	8	4	7	5	1	6	11	16	15	13	19	12	21	14	18	26	24	17	22	25	27	20	28	23	29
Congo	10	3	4	2	5	7	8	9	1	6	16	11	12	13	17	14	19	18	15	24	22	20	23	26	25	21	28	27	29

Democratic Republic of the Congo	10	3	9	2	6	7	4	8	1	5	15	16	11	12	18	13	20	14	17	25	22	19	23	26	28	21	27	24	29
Equatorial Guinea	10	3	4	1	5	11	6	8	2	7	16	12	13	9	19	15	17	18	14	25	20	22	24	28	26	21	23	27	29
Gabon	10	2	4	1	5	7	8	12	3	6	16	9	11	13	18	14	17	19	15	24	22	20	23	27	25	21	26	28	29
**Eastern sub-Saharan Africa**	10	9	7	2	6	8	4	3	1	5	13	18	17	14	20	12	19	16	11	25	15	23	26	21	27	24	28	22	29
Burundi	10	9	8	2	6	7	3	5	1	4	13	18	16	14	20	11	23	12	15	25	17	22	26	19	27	24	28	21	29
Comoros	11	7	6	2	3	8	5	9	1	4	15	12	16	14	17	13	20	19	10	25	18	23	24	21	27	22	28	26	29
Djibouti	10	7	5	2	3	9	4	8	1	6	15	13	16	14	20	12	18	17	11	25	19	22	27	21	26	23	28	24	29
Eritrea	10	8	6	2	9	5	4	7	1	3	14	18	17	13	16	11	21	15	12	24	19	23	26	20	27	25	28	22	29
Ethiopia	10	9	5	3	7	6	4	1	2	15	14	20	16	23	19	13	17	11	12	26	8	24	22	21	28	25	27	18	29
Kenya	11	10	7	1	4	5	6	9	2	3	14	15	18	12	21	8	23	16	13	27	24	19	22	17	25	20	29	26	28
Madagascar	10	9	7	2	5	8	4	6	1	3	14	16	18	13	19	12	21	15	11	23	17	24	26	20	27	25	28	22	29
Malawi	12	9	8	4	7	15	2	6	1	3	14	17	5	10	22	18	11	16	13	19	20	26	25	27	29	23	24	21	28
Mozambique	9	6	5	3	11	8	4	2	1	7	13	15	16	10	19	18	22	14	12	23	17	21	25	29	27	24	26	20	28
Rwanda	12	8	6	2	4	9	3	7	1	5	11	15	17	14	19	13	20	16	10	25	18	22	26	21	27	23	28	24	29
Somalia	10	8	9	5	7	6	4	2	1	3	11	21	17	15	19	13	23	12	14	25	18	22	26	20	27	24	28	16	29
South Sudan	10	8	6	5	3	9	7	2	1	4	13	15	17	11	19	12	18	16	14	27	20	23	26	21	25	24	28	22	29
Uganda	10	9	6	2	3	8	5	7	1	4	11	13	19	16	15	14	21	17	12	25	18	26	27	20	24	23	28	22	29
United Republic of Tanzania	11	8	7	2	4	9	5	3	1	6	20	15	16	10	19	13	18	14	12	25	17	23	26	21	27	22	28	24	29
Zambia	13	8	5	2	6	9	3	7	1	4	19	14	16	10	20	12	18	17	11	24	15	22	27	21	26	25	28	23	29
**Southern sub-Saharan Africa**	1	4	6	3	5	9	11	12	2	7	8	10	16	13	17	15	20	19	14	22	23	21	24	28	26	18	27	29	25
Botswana	5	4	6	2	3	8	12	9	1	7	21	10	15	16	14	11	19	17	13	22	27	18	24	26	23	20	28	29	25
Eswatini	4	5	8	2	6	9	10	12	1	7	3	11	16	13	17	15	18	19	14	22	23	20	25	27	26	21	29	28	24
Lesotho	4	3	8	2	5	9	11	10	1	7	6	13	18	15	16	12	21	19	14	23	24	17	25	26	27	20	29	28	22
Namibia	2	8	6	1	3	14	5	11	4	17	13	12	19	9	20	7	18	21	16	10	23	15	28	29	22	24	26	27	25
South Africa	1	3	6	2	5	10	11	12	4	7	8	9	16	13	17	15	19	18	14	22	23	21	25	28	26	20	27	29	24
Zimbabwe	7	6	8	2	3	9	11	14	1	4	5	12	10	22	15	17	23	21	13	20	18	19	24	25	27	16	28	26	29
**Western sub-Saharan Africa**	12	4	5	1	3	6	8	7	2	15	9	10	14	11	19	18	17	13	16	26	25	21	23	24	28	22	27	20	29
Benin	13	6	8	2	4	3	7	5	1	10	9	11	16	12	19	18	15	14	17	26	23	20	21	27	28	22	24	25	29
Burkina Faso	11	6	8	2	5	3	7	4	1	9	14	13	15	10	19	17	16	12	18	26	23	20	21	27	28	22	25	24	29
Cabo Verde	18	3	7	4	1	2	10	11	6	5	8	9	13	14	16	12	15	17	19	25	21	20	22	26	24	23	27	28	29
Cameroon	12	4	6	2	3	5	10	7	1	11	23	8	13	9	17	16	19	15	14	27	24	18	21	20	26	22	28	25	29
Chad	12	5	8	6	3	2	7	4	1	10	9	15	14	11	18	16	17	13	19	27	24	20	21	26	28	22	25	23	29
Côte d'Ivoire	16	4	6	2	5	3	8	7	1	10	9	11	13	12	19	17	18	14	15	27	23	20	21	25	28	22	26	24	29
Gambia	10	4	6	3	9	8	5	7	2	15	1	11	14	19	17	13	18	16	12	26	21	23	20	28	27	22	25	24	29

Ghana	14	6	4	1	3	8	11	10	2	16	9	5	13	7	17	20	18	12	15	25	24	19	21	28	27	22	23	26	29
Guinea	12	6	8	5	3	4	7	11	1	14	2	17	9	22	15	10	16	18	13	20	24	21	23	28	27	26	25	19	29
Guinea-Bissau	13	4	7	2	5	3	8	6	1	10	9	12	14	11	18	17	19	15	16	26	23	20	21	25	27	22	28	24	29
Liberia	12	5	7	2	3	4	8	6	1	10	9	11	14	13	18	17	19	16	15	25	23	20	22	26	27	21	28	24	29
Mali	9	7	6	4	11	2	10	8	1	13	3	12	5	20	18	16	14	15	17	24	21	19	22	28	27	25	23	26	29
Mauritania	12	4	6	2	3	5	8	9	1	11	10	7	14	13	16	17	19	18	15	24	23	20	21	27	26	22	28	25	29
Niger	9	7	8	6	4	3	5	2	1	10	22	13	12	18	15	14	16	11	17	25	28	19	20	27	26	21	24	23	29
Nigeria	13	6	4	1	2	9	5	7	3	22	11	8	18	12	23	20	16	10	14	25	26	17	24	19	28	21	27	15	29
São Tomé and Príncipe	14	4	5	3	7	6	2	10	1	11	15	16	8	13	12	19	18	23	9	25	22	20	17	27	26	21	24	29	28
Senegal	8	5	6	2	3	4	7	9	1	10	16	11	13	12	18	17	19	15	14	25	23	20	21	26	27	22	28	24	29
Sierra Leone	11	5	8	2	3	4	7	6	1	10	9	13	15	12	19	18	17	14	16	26	23	20	21	27	28	22	25	24	29
Togo	12	4	6	2	5	3	8	7	1	10	9	11	14	13	18	17	19	16	15	25	23	20	21	26	27	22	28	24	29

eFigure 12: Ranking of 29 cancer groups by incidence in 2019, by country and location group

Footnote: Cancer rankings by location compare the absolute number of incident cases for each cancer, with colors ranging from the highest ranking (red) to the lowest ranking (green) within that location. Socio-demographic index (SDI) quintiles are ordered from high to low SDI quintile. Countries and location groups are ordered alphabetically by the GBD location hierarchy of 7 super-regions (bold, italicized) and 21 regions (bold).

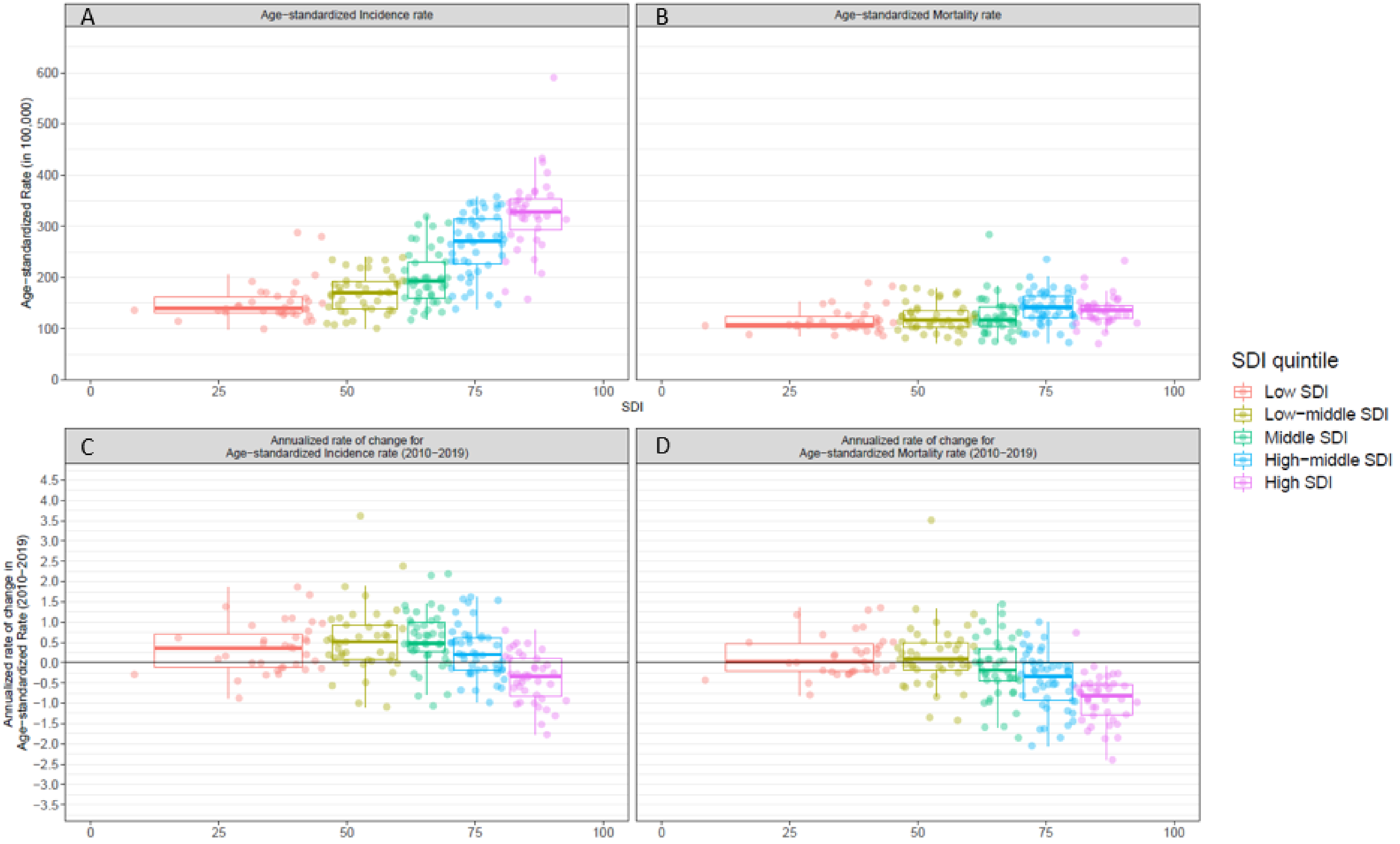

eFigure 13: Age-standardized rates and Annualized rate of change for age-standardized rates for Total Cancers excluding Non-Melanoma Skin Cancer, All Ages, Both Sexes

Footnote: Panels provide global estimates for total cancers except non-melanoma skin cancer, stratified by quintile of Socio-demographic Index. Annualized rate of change from 2010 to 2019 represents the average percentage change per year over this period. Dots represent individual countries and territories. Box plots show the 25th, 50th, and 75th percentile of each SDI quintile, with vertical bars representing bounds for outliers (bars extend to the furthest observation that is within a distance of 1.5 times the interquartile range from the 25th or 75th percentiles). The x-axis provides SDI values, quintile cutoffs are described in the eAppendix. Panels provide: A) age-standardized incidence and mortality rates per 100,000 persons in 2019; B) age-standardized incidence rates per 100,000 persons in 2019; C) annualized rate of change in the age-standardized incidence rates from 2010 to 2019; and D) annualized rate of change in the number of age-standardized mortality rates from 2010 to 2019.

A) Both sexes combined

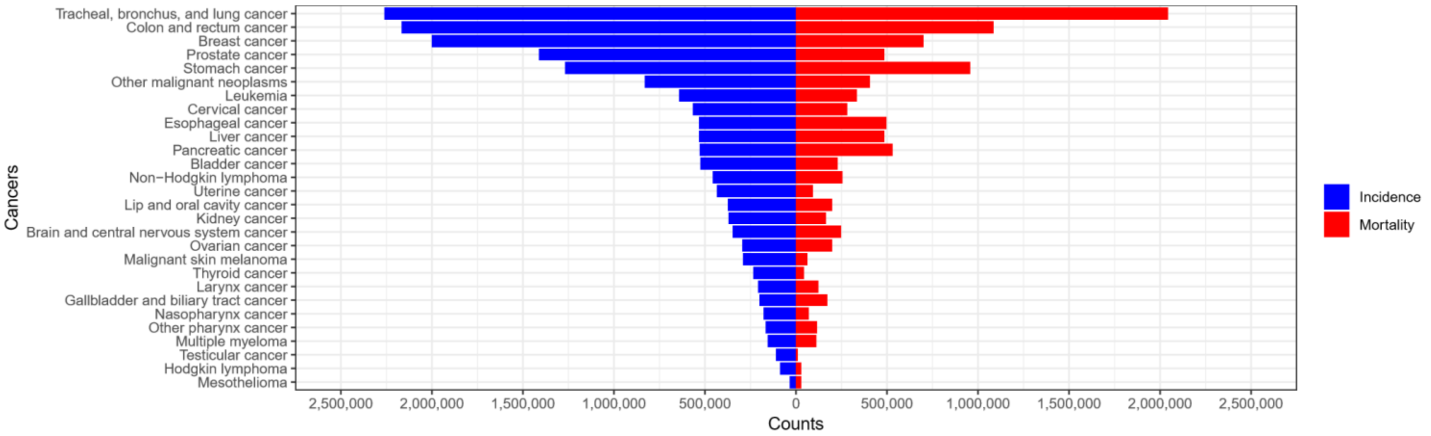

B) Male only

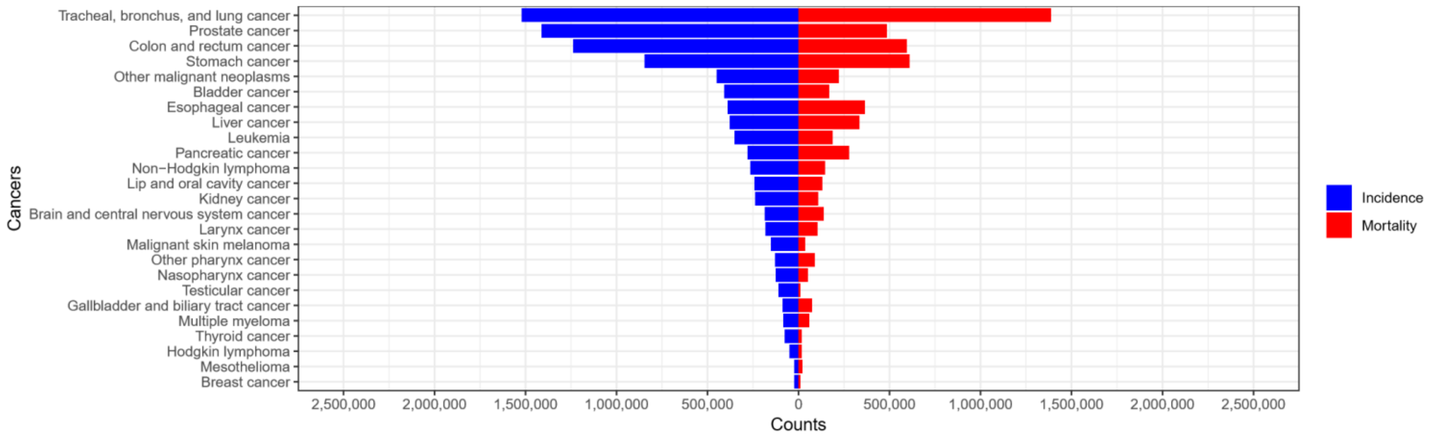

C) Female only

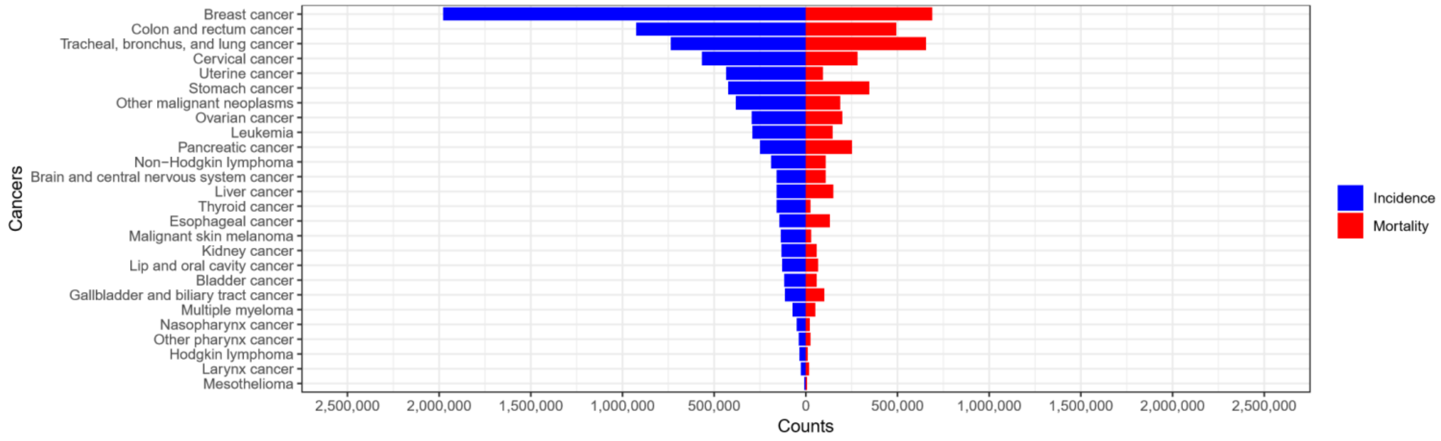

eFigure 14: Global counts of cancer-specific incidence and deaths in 2019, overall and by sex

Footnote: Panels provide the global absolute counts of cancer incidence and mortality in 2019, in order of decreasing incidence, for A) both sexes combined, B) males only, and C) females only. Values and associated 95% uncertainty intervals are reported in Table 1.

A) Both sexes combined

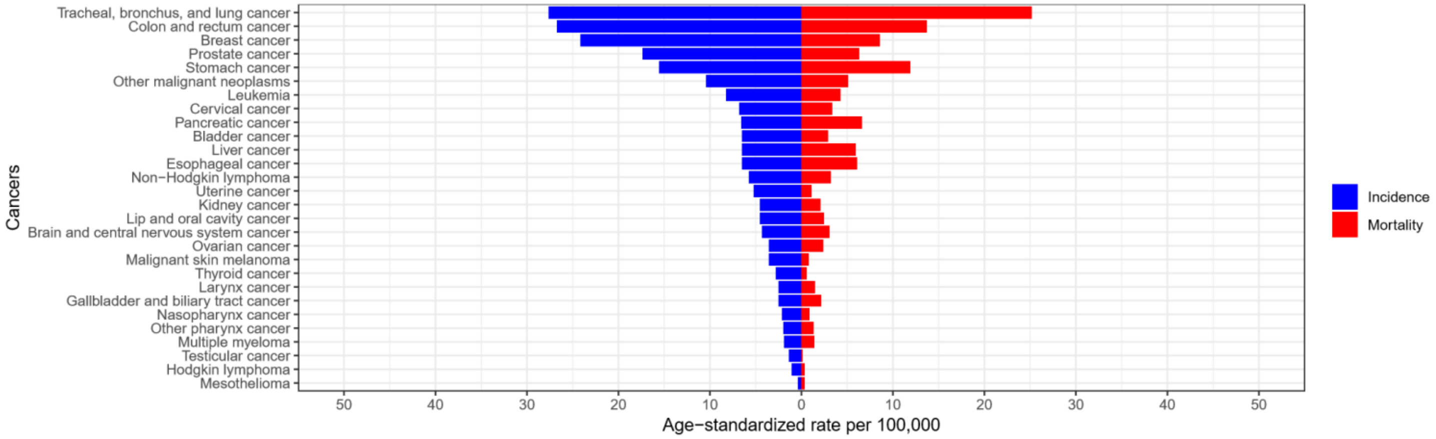

B) Male only

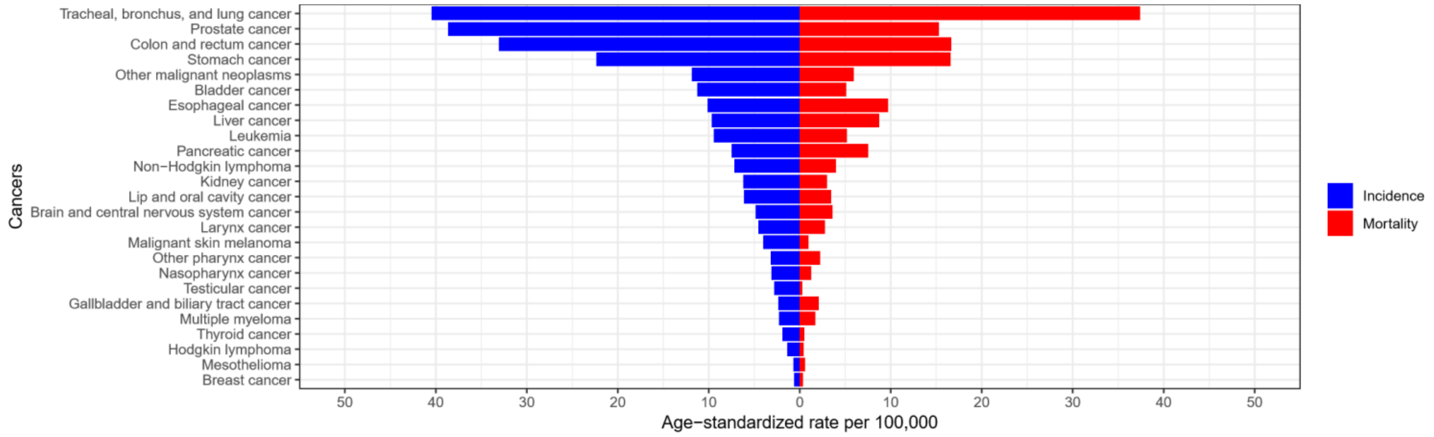

C) Female only

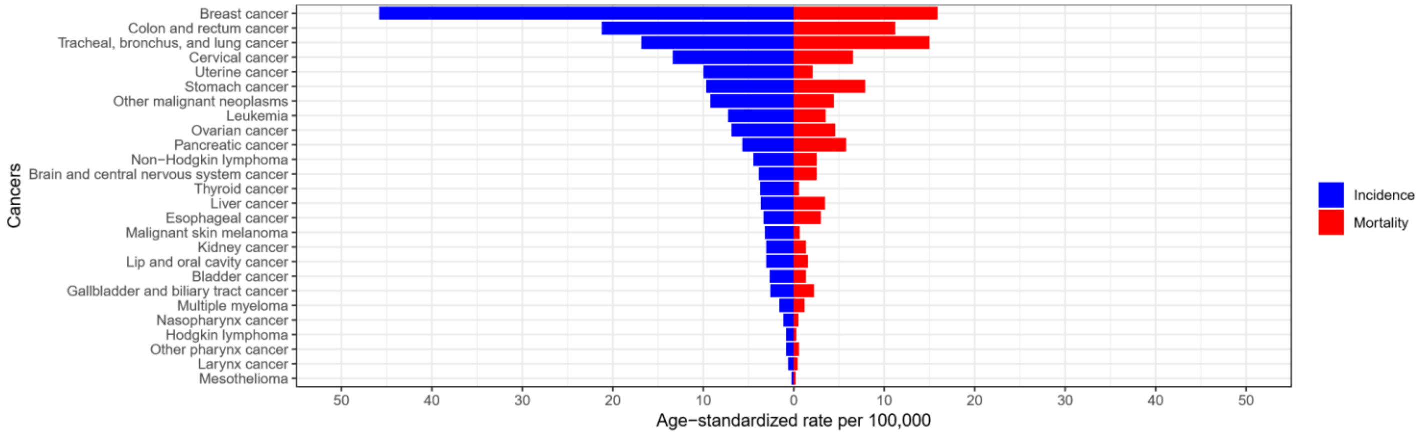

eFigure 15: Global age-standardized cancer-specific incidence and mortality rates in 2019, overall and by sex

Footnote: Panels provide the global age-standardized rates per 100,000 of cancer incidence and mortality in 2019, in order of decreasing incidence, for A) both sexes combined, B) males only, and C) females only. Values and associated 95% uncertainty intervals are reported in Table 1.
